# Asymmetric synthesis of alkyl fluorides *via* biocatalytic reduction of α-fluoroenones and α-fluoroenoates†

**DOI:** 10.1039/d6gc00545d

**Published:** 2026-05-13

**Authors:** Helen Allan, Yu Wang, Bethan Winterson, Alexandra King, Abil E. Aliev, Rachel Szpara, Victor Laserna, Charlotte E. Coomber, John M. Ward, Jack W. E. Jeffries, Helen C. Hailes, Tom D. Sheppard

**Affiliations:** a Department of Chemistry, University College London 20 Gordon Street London WC1H 0AJ UK h.c.hailes@ucl.ac.uk tom.sheppard@ucl.ac.uk; b Department of Biochemical Engineering, University College London Bernard Katz Building Gower Street London WC1E 6B UK

## Abstract

Here we report the first biocatalytic asymmetric synthesis of alkyl fluorides *via* reduction of α-fluoroenones and α-fluoroenoates using ene reductase enzymes. The reduction of a wide range of (*Z*) or (*E*)-α-fluoroenones was shown to proceed in high yield and selectivity using ene reductases. Importantly, the different alkene geometries led to opposite enantiomers of the chiral fluoroalkane. The reaction could also be successfully extended to α-fluoroenoates to access enantioenriched α-fluoroesters with only the *E*-alkene isomers undergoing reduction, enabling mixtures of alkene geometries to be employed. The selectivity and substrate scope were rationalized using *in silico* substrate-enzyme molecular docking studies. The enantioenriched alkyl fluorides were elaborated *via* chemical transformations to access further functionalized building blocks for synthesis.

## Abstract 2

(*E*) or (*Z*) α-Fluoroenones and (*E*) α-fluoroenoates undergo asymmetric reduction with ene reductase enzymes to give sp^3^-fluorides with high levels of enantioselectivity.

## Graphical abstract

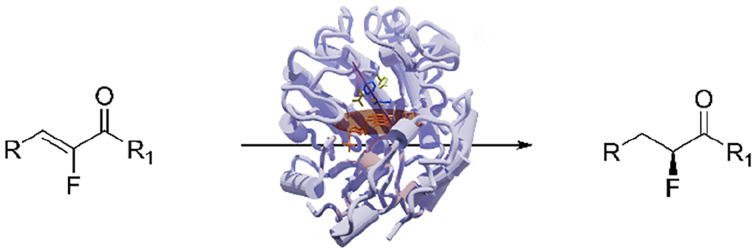

**Green foundation**

1. This work describes a greener and more sustainable approach to chiral fluorinated compounds with significant environmental benefits over existing methods which require expensive and/or hazardous (and non-renewable) transition metal catalysts.

2. This paper reports the first generally applicable method for asymmetric synthesis of alkyl fluorides using biocatalysis, by employing ene-reductase enzymes to reduce fluoroenones and related systems with high stereoselectivity. This biocatalytic method offers efficient and sustainable reaction conditions, *e.g.* mild ambient temperature/pressure, in aqueous solution, and has a wide substrate scope.

3. The sustainability of the process could be improved in the future by developing greener methods for preparing the required fluoroenone starting materials from sustainably sourced chemical building blocks.

Fluorinated molecules make up ∼20% of marketed drugs and an ever-increasing number of pharmaceutical candidates;^1–3^ an even larger proportion of agrochemicals in development contain fluorine.^4,5^ This reflects the significant utility of fluorine in biologically active molecules, and there is considerable interest in novel methods for the synthesis of fluorinated organic compounds. Importantly, the fluorine atom provides a largely inert functional group that can increase metabolic stability and compound permeability. Moreover, it can perturb lipophilicity and p*K*_a_ – impacting on binding affinities, and C(sp^3^)–F centres can provide control over the molecular shape through conformational interactions of the polar C–F bond.^3,6–9^ Compounds bearing a fluorine atom at a chiral centre are relatively unusual in bioactive molecules. However, examples describing the stereoselective replacement of an sp^3^ C–H with C–F and the resulting impact on biological activities highlight the importance of such compounds.^6,10^

Approaches reported for the enantioselective synthesis of chiral centres containing a C–F bond^11–14^ include electrophilic fluorination of carbonyl compounds^15–22^ or the synthesis of benzylic fluorides *via* asymmetric catalysis,^23,24^ the use of hydrogen-bonding phase transfer catalysts for catalytic asymmetric S_N_2 reactions of fluoride,^25,26^ and the use of a chiral fluorinating reagent.^27^ Direct fluorination of enantioenriched alcohols with reagents such as DAST is perhaps the most accessible approach, but it is potentially hazardous to scale-up and promotes competing elimination processes often resulting in low product yields,^28^ though newer reagents have been developed to address these issues, with some asymmetric examples reported.^29–32^

A versatile approach is the asymmetric reduction of fluorinated alkenes. This has been achieved with a range of homogeneous transition metal complexes for specific classes of alkene, though typically heavier/expensive transition metals are required.^33–37^ An attractive alternative strategy would be a biocatalytic fluoroalkene reduction. The use of biocatalysts is continuing to grow, playing an important role in the fine chemical and pharmaceutical industries by providing a sustainable and green synthetic strategy for the preparation of high-value chemicals. They have significant potential compared to traditional organic chemistry approaches, avoiding the use of expensive chiral catalysts, toxic or rare transition metals, toxic solvents or extreme temperatures and pressures. Biocatalysts are typically employed under mild reaction conditions in aqueous media, giving excellent chemo- and stereoselectivities.

The use of biocatalysts for the asymmetric reduction of fluorinated alkenes has received limited attention to date. The reduction of α-fluorocrotonic acid using a *Clostridium kluyveri* whole cell system under a hydrogen atmosphere was reported (Scheme 1a), although the enantioselectivity was not determined.^38^ A purified oxygen sensitive enoate reductase from the organism was subsequently studied for the same reaction.^39^ More recently, the asymmetric reduction of three fluorinated cinnamyl alcohols using Baker's yeast was reported (Scheme 1b), but the stereoselectivity was moderate.^40^ An alternative enzymatic photodecarboxylation approach to α-fluoroesters has also been described.^41^ We envisaged that ene reductase (ERED) enzymes could offer a generally applicable strategy for the asymmetric reduction of fluorinated alkenes bearing a carbonyl group. Ene reductases (EREDs) are flavin-containing enzymes that reduce alkenes activated with an electron-withdrawing group, *via* a *trans*-hydrogenation.^42–44^ The predominant family of EREDs is the old yellow enzyme (OYE) nicotinamide NAD(P)H/Flavin dependent oxidoreductases which catalyse the reduction of α,β-unsaturated compounds including ketones, aldehydes, and nitro compounds; esters and acids are less readily accepted.^42–44^ In recent years many EREDs have been described, including some that can accept sterically challenging enones, recently discovered in our laboratory *via* a sequence-based functional metagenomics strategy.^42–45^ EREDs have been applied in the synthesis of industrially useful compounds such as pregabalin precursors, flavour precursor molecules and other high value chiral building blocks on an industrially relevant scale.^46–48^ Interestingly, Hyster *et al.* have recently reported one example of a fluoroalkene photoenzymatic radical cyclisation using an ERED (Scheme 1c).^49^ However, the direct ERED mediated reduction of fluoroenones/enoates has not been described.^50^ Moreover, the successful reduction of α-haloenoates (Cl, Br, I) with generally high selectivity has been demonstrated,^51–55^ but the corresponding α-fluorocinnamate was reported to be unreactive (Scheme 1d).^52^ Here, a highly flexible novel biocatalytic approach to enantioenriched sp^3^ fluorides, *via* the reduction of α-fluoroenones and α-fluoroenoates, is described to provide access to an array of functionalized chiral fluorinated acylic/cyclic compounds, in good to excellent yields and stereoselectivities (Scheme 1e).

**Scheme 1:**
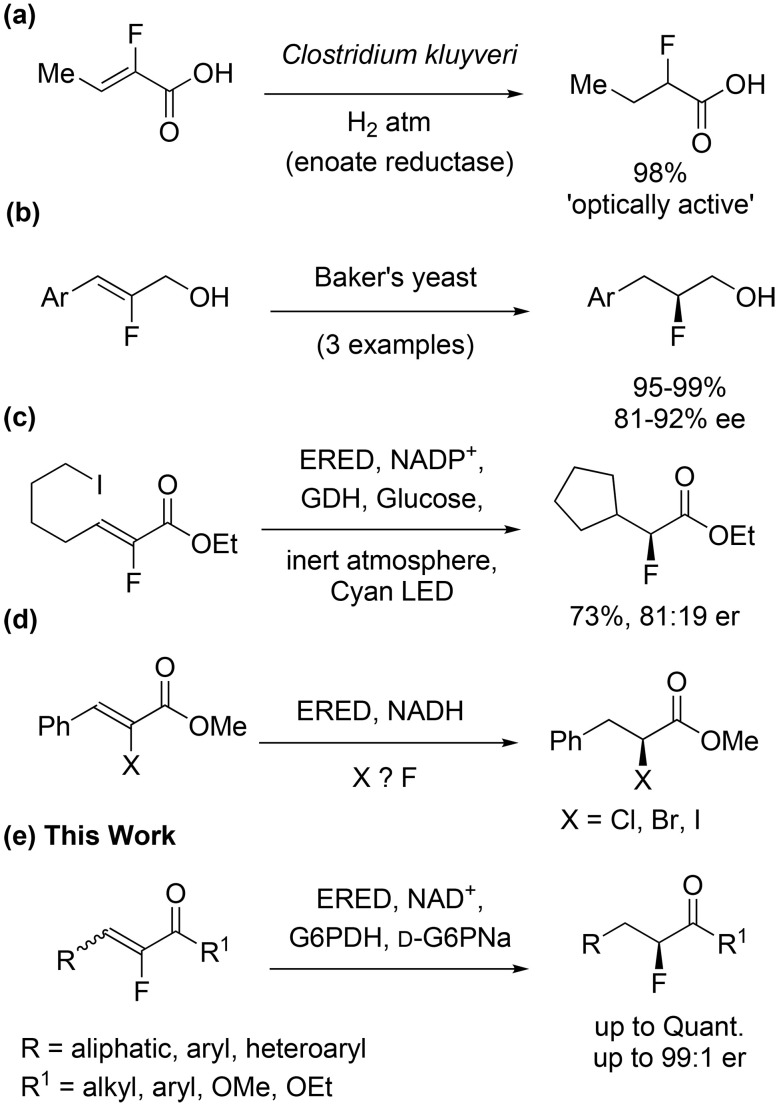
Biocatalytic routes to stereogenic sp^3^ fluorine centres. (a) Asymmetric reduction of α-fluorocrotonic acid by an air-sensitive enoate reductase from *clostridium kluyveri*.^38^ (b) Asymmetric reduction of fluorinated cinnamyl alcohols using Baker's yeast.^40^ (c) A fluoroalkene photoenzymatic radical cyclisation using an ERED.^49^ (d) Enantioselective reduction of α-halocinnamates (X ≠ F) using EREDs.^52^ (e) Asymmetric reduction of α-fluoroenones and α-fluoroenoates using EREDS (this work).

Initially we focused our attention on the reduction of α-fluoroenones which have not previously been reported to be reduced by EREDs. They were readily prepared *via* the Horner–Wadsworth–Emmons olefination of aldehydes using a fluorinated phosphonate under conditions developed by Coutrot *et al.*^56,57^ Samples of the racemic products for use as analytic standards were synthesised *via* the Pd-catalyzed decarboxylation of fluorinated β-ketoesters.^58^ Several EREDs, heterologously expressed in *E. coli* BL21 (DE3) were selected, including NCR from the bacterium *Zymomonas mobilis* which has been used successfully with a range of linear substrates, and our previously reported EREDs pQR1445 and pQR1907 from a drain metagenome which exhibited good organic solvent tolerance.^45^ The EREDs were co-expressed with glucose-6-phosphate dehydrogenase (G6PDH) to recycle the NADH co-factor *in situ*, utilizing d-glucose-6-phosphate sodium salt (d-G6PNa) as co-substrate. Enzyme lysates were employed in all reactions as these are typically used in industry, negating the need for costly enzyme purification. Initial screens explored the reduction of (*Z*)-3-fluoro-4-phenyl-3-buten-2-one (1a) and reactions were monitored by HPLC. The highest activity was seen with NCR, which gave the complete conversion of 1a to the desired fluoroalkane ((*S*)-7a) with excellent stereocontrol (99 : 1 er, chiral HPLC analysis) (Fig. 1a). A range of substrates were then screened against NCR, pQR1445^45^ and pQR1907.^45^ The highest activity was seen with NCR in all but one case (Fig. 2).

**Fig. 1:**
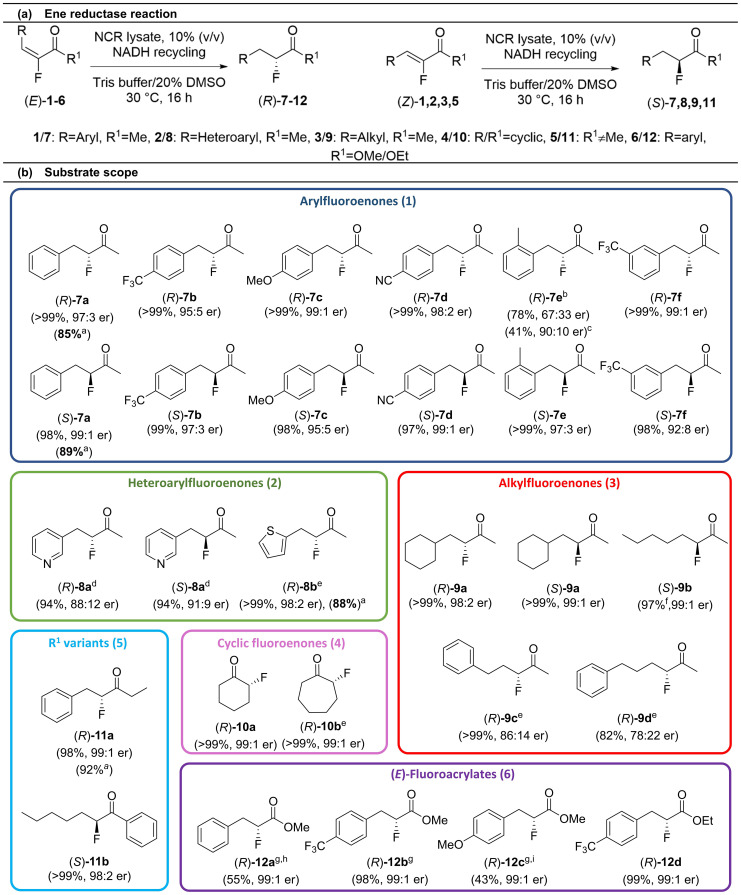
(a) Ene reductase reaction of fluoroenones and fluoroenoates; (b) Substrate scope. Substrate (10 mM), ERED and G6PDH lysates (10% (v/v), co-expression, total protein in the lysates 1 mg mL^−1^), NAD^+^ (1 mM), G6PNa (50 mM), in Tris-HCl (100 mM) and DMSO (20%) at pH 7.5, 30 °C, 16 h, 700 rpm. Reactions were performed in triplicate. Yields and enantiomeric ratios were determined by HPLC or GC analysis. ^*a*^ Isolated yield from preparative scale reaction. ^*b*^ 80% (v/v) enzyme lysate concentration. ^*c*^ NCR-G270Y used, 80% (v/v) enzyme lysate concentration. ^*d*^ pQR1445 used, 20% (v/v) enzyme lysate concentration. ^*e*^ 60% (v/v) enzyme lysate concentration. ^*f*^ Conversion; no starting material remaining by GC, but both product and starting material are volatile. ^*g*^ 40% (v/v) enzyme lysate concentration. ^*h*^ from 8 : 1 (*E* : *Z*); ^*i*^ from 5 : 1 (*E* : *Z*); where the SM was a mixture of isomers, yield is based upon the conversion of the *E* isomer to the product.

**Fig. 2:**
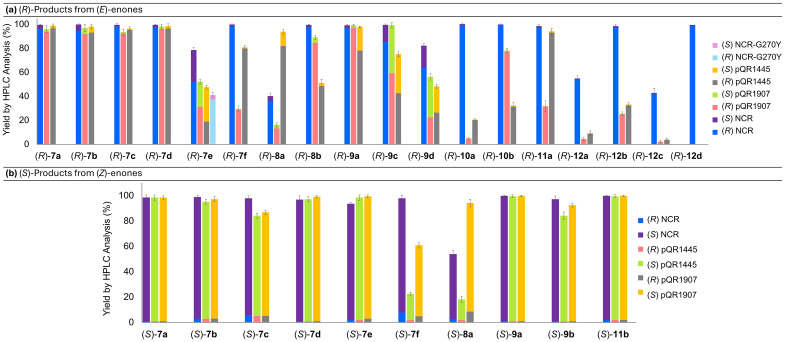
Enzyme screening results for substrates. All substrates were screened against at least 3 different EREDs (NCR/pQR1907/pQR1445) and the quantity of each product enantiomer obtained with each enzyme is shown by the indicated colours on the charts. Substrate (10 mM), ERED and G6PDH lysates (10% (v/v), co-expression, total protein in the lysates 1 mg mL^−1^), NAD^+^ (1 mM), G6PNa (50 mM), in Tris-HCl (100 mM) and DMSO (20%) at pH 7.5, 30 °C, 16 h, 700 rpm. Reactions were performed in triplicate. Yields and enantiomeric ratios were determined by HPLC or GC analysis. See SI Table S1 for further details.

The NCR ERED was found to accept a wide range of α-fluoroenones (substrates 1–5 to give compounds 7–11) and reductions occurred with excellent yields and stereoselectivities with both (*Z*) and (*E*)-α-fluoroenones accepted by the enzyme (Fig. 1a and b) which showed high activity. For example, with (*E*)-1a, (*R*)-7a was readily formed in >99% yield with a *k*_cat_ of 1.18 s^−1^ (100 μg mL^−1^ WT-NCR) and a turnover number (TON) of 3950. Interestingly the opposite stereochemistry was observed starting from the (*Z*)-alkene compared to when the (*E*)-alkene was used. The observed stereoselectivity and substrate acceptance is particularly notable as often these enzymes are unable to accept both alkene geometries. Substrates with a range of β-aryl substituents were accepted, yielding fluorides containing electron poor (7b, 7d) and electron rich (7c) aryl groups, and a range of aryl substitution patterns (7e, 7f). Enones bearing heteroaromatic groups (2 to give 8a, 8b) were also successfully reduced, as well as substrates with alkyl groups (3 to give 9a, 9b). Interestingly, for 9c and 9d with longer chains between the Ph and carbonyl group, the er decreased, most likely due to steric constraints affecting preferred conformations of the (*E*)-isomer in the active site. Moreover, 6- and 7-membered ring cyclic fluoroketones could be formed with high enantiopurity (10a, 10b).

While most products were obtained in high yield and selectivity, the substrates to produce the ((*R*)-*o*-tolyl product (*R*)-7e) and pyridyl products (8a) were less well accepted. Low conversions were seen for both (*Z*)- and (*E*)- pyridyl substrates (2a to give 8a) using NCR (<50% v/v enzyme lysate concentration) but product enantiopurity was improved at higher enzyme loadings (60% v/v). Changing the enzyme to pQR1445 (20% enzyme lysate concentration) gave excellent yields and moderate to good selectivities (>90%, (*S*)-8a: 9 : 91 er; (*R*)-8a: 88 : 12 er). For the preparation of (*R*)-7e a higher enzyme concentration was attempted but the reaction could not be pushed to completion and a low er was observed (67 : 33 er). Enzyme mutagenesis was therefore conducted to improve the stereoselectivity in the formation of (*R*)-7e. Using the reported X-ray crystallographic data for NCR (PDB database (4A3U)),^59^*in silico* molecular docking was carried out using AutoDock Vina (v.1.2.0).^60,61^ The docking study revealed two viable binding modes for (*E*)-7e (Fig. S10A and B). The slight energetic preference for the (*R*)-product could account for the WT-NCR selectivity observed. In an attempt to improve the (*R*)-selectivity, the residue Gly270 was substituted with a bulkier tyrosine (G270Y). Modelling studies indicated that the Tyr-phenolic ring occupied the space required for the (*S*)-productive binding mode, thereby blocking the formation of (*S*)-7e (Fig. S10C). When used in reactions, NCR-G270Y gave a higher stereoselectivity towards (*R*)-7e (90 : 10 er), but a lower yield of 41% (by GC, Fig. S10) compared to the WT-NCR (78% yield, 67 : 33 er). Indeed, molecular docking experiments indicated that the binding energy of (*E*)-7e with NCR-G270Y (Δ*G*_bind_ = −5.8 kcal mol^−1^, Fig. S10C) was higher than for the WT-NCR, supporting the lower conversion but enhanced *R*-selectivity. NCR was also found to accept bulkier ketone substituents. When the ethyl ketone (5a) was subjected to the reaction conditions the (*E*)-fluoroenone gave the corresponding fluoroalkane (*R*)-11a in 98% yield and 99 : 1 er; the (*Z*)-fluoroenone was poorly accepted, however, with <5% conversion to (*S*)-11a. A phenyl ketone was well accepted giving fluoride (*S*)-11b in excellent yield and enantiopurity from the (*Z*)-enone, though the corresponding (*E*)-enone substrate was not tested due to purification issues.

Less activated alkenes such as enoates are considered as borderline substrates for ene reductases. We reasoned that the electronegative fluorine atom may activate these substrates sufficiently to enable reduction to occur. A range of (*E*)- and (*Z*)-fluoroenoates were synthesized *via* a Horner–Wadsworth–Emmons reaction-(*E*) or TiCl_4_ mediated aldol condensation-(*Z*) and subjected to the reaction (Fig. 1c).^62^ Authentic samples of reaction products were synthesized *via* a one-pot substitution/Krapcho decarboxylation.^63^ Whilst aryl (*E*)-enoates (6) were accepted by the enzyme, the conversion was lower than with (*E*)-enones (1–5). The enzymes were most active towards electron poor enoates (6b to give 12b, 98% yield) compared to electron rich enoates (6c to give 12c, 55% yield); both methyl (6a–c) and ethyl (6d) esters were accepted. In all cases the stereocontrol was excellent. However, an (*E*)-alkylenoate (methyl-2-fluorohept-2-enoate) and all tested (*Z*)-fluoroenoates showed no conversion. This was in line with the ketone results above, where a more sterically demanding ketone substituent (Et), comparable in size to OMe/OEt, led to low reactivity of the (*Z*)-isomer. This enzyme selectivity is especially useful when preparing the (*E*)-enoates *via* a Horner–Wadsworth–Emmons reaction. The resultant mixture of (*E*)- and (*Z*)- enoates can be used in the ERED reaction without the need for prior separation with no decrease in the enantiopurity of the product.

The reactions were amenable to biocatalytic preparative scale reactions (20–130 mg) giving an isolated yield of 89% of (*S*)-7a from (*Z*)-1a; 86% of (*R*)-7a from (*E*)-1a; 88% of (*R*)-8b from (*E*)-2b and 92% of (*R*)-11a from (*E*)-5a (Fig. 1). Authentic reference standards of (*R*)-10a, (*R*)-11a and (*R*)-12a were synthesized in order to assign the absolute stereochemistry of the products (correlated by HPLC/GC on a chiral stationary phase) from the biocatalytic reactions (SI).

Further docking studies were conducted to rationalise the high reactivities and excellent stereoselectivities observed with NCR with substrates (*E*)-1a and (*E*)-6a and also (*Z*)-1a as ligands. As indicated in Fig. 3 the ligands were orientated in the catalytic pocket of NCR with the carbonyl group complexed to His172 (3.69–4.41 Å, van der Waals interaction) and Asn175 (2.91–3.17 Å, H-bond). Hydride transfer occurs from the reduced flavin to the β-C of the ligands while the α-C is protonated by Tyr177. Substrates (*E*/*Z*)-1a and (*E*)-6a can adopt productive conformations with hydride addition and then protonation in a *trans*-fashion to the alkene (Fig. 3A–C). While the (*E*)-fluoroenones and (*E*)-fluoroenoates bind in a ‘classical’ orientation,^64^ the alkene system in the (*Z*)-fluoroenone has ‘flipped’ relative to the flavin cofactor to maintain the required *trans*-reduction geometry, thereby giving the opposite stereoselectivity. This mirrors the ‘flipped’ substrate binding mode observed by Stewart *et al.*^64^ While they demonstrated that this ‘flip’ is controlled by steric gatekeeping, here the ‘flipped’ mode is likely in response to the (*Z*)-1a-alkene geometry as the enzyme can accommodate the substrate. However, for substrate (*Z*)-6a, docked conformations are not productive as they would require hydride delivery and subsequent protonation to occur from the same face of the alkene (Fig. 3D). Docking studies detailing the orientation of 5a,b in the enzyme pocket can be found in the SI.

**Fig. 3:**
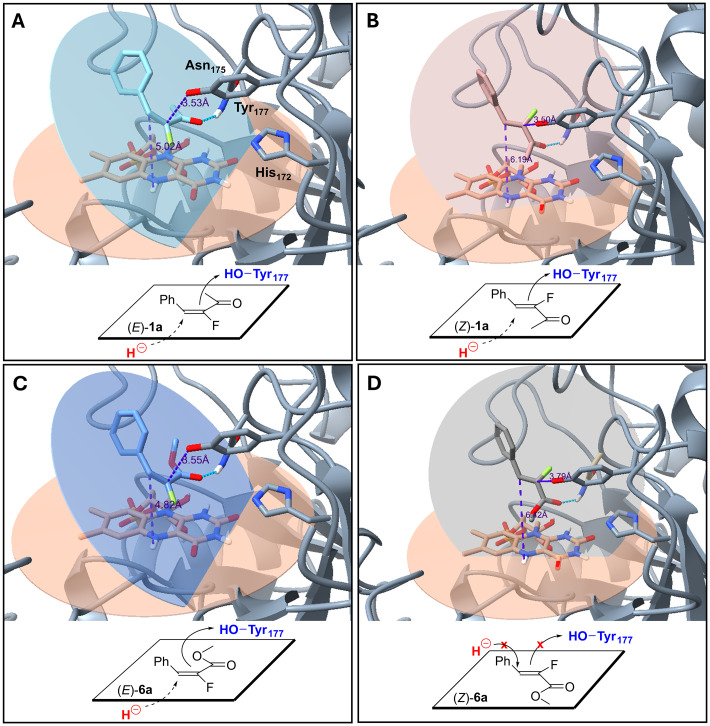
Docking studies of substrates (*E*/*Z*)-1a and 6a with NCR. (A) (*E*)-1a with NCR, giving product (*R*)-7a. (B) (*Z*)-1a with NCR, giving product (*S*)-7a. (C) (*E*)-6a with NCR, giving product (*R*)-12a. (D) (*Z*)-6a with NCR. No product was generated as the hydride delivery and the protonation from Tyr177 are required from different surfaces.

The selective formation of two contiguous stereocentres from a tetrasubstituted alkene was explored using deuterated alkenes (*E*)-[4-D_1_]-1a and (*Z*)-[4-D_1_]-1a, synthesized from benzaldehyde-α-d_1_. After reduction, the resulting diastereoisomers were analyzed by ^1^H, ^13^C and ^19^F NMR (see SI). Reduction of (*E*)-[4-D_1_]-1a gave the (3*R*,4*S*)-diasteroisomer (Scheme 2) whereas (*Z*)-[4-D_1_]-1a gave the (3*S*,4*S*)-stereochemistry (SI). This corroborated the reported *trans*-reduction mechanism whereby the hydride addition and protonation occur on opposite faces of the alkene,^45^ and exemplified stereoselective routes to isotopically labelled species.

**Scheme 2:**
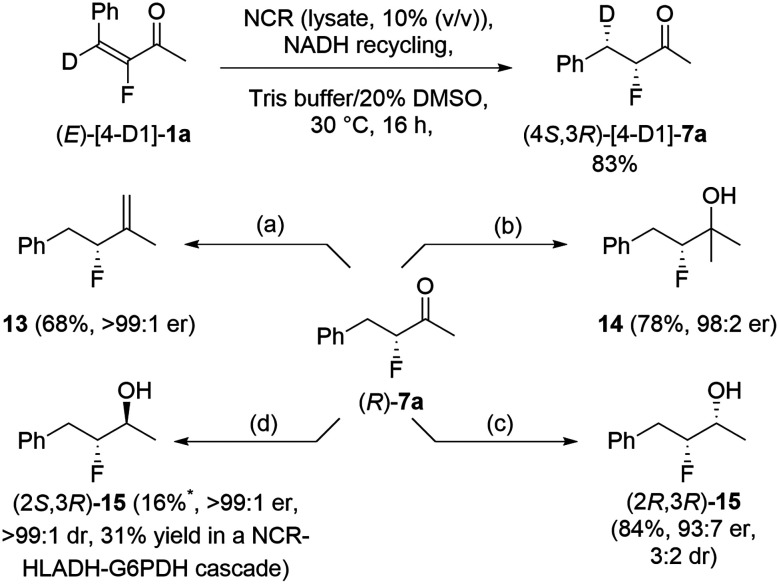
(a) Ph_3_PMeBr, ^*n*^BuLi, THF; (b) MeMgBr, THF, 0–25 °C; (c) NaBH_4_, MeOH, 0 °C; (d) HLADH (40% v/v lysate), KPi buffer, ZnCl_2_ (1 mM), 25 °C, 12 h; *NMR yield with ethyl fluoroacetate as internal standard. For the cascades reaction: HLADH (pure enzyme at 2 mg mL^−1^), Tris buffer (pH7.5, 100 mM), 30 °C, 48 h.

Subsequently, we explored transformations of enantioenriched reduction product (*R*)-7a to access other enantioenriched fluorinated building blocks (Scheme 2). Wittig olefination gave alkene 13 in 68% yield, Grignard addition gave tertiary alcohol 14 in 78% yield, and chemical reduction of the ketone using NaBH_4_ provided fluorohydrin 15 in 84% yield, 3 : 2 dr ((2*R*,3*R*):(2*S*:3*R*)). Enzymatic reduction of the ketone (unoptimized) using horse liver alcohol dehydrogenase (HLADH) proceeded with excellent diastereoselectivity (99 : 1) to give alcohol (2*S*,3*R*)-15. This could be conducted in a one-pot enzyme cascade to yield (2*S*,3*R*)-15 in 31% yield directly from enone (*E*)-1a.

In this work, we have demonstrated that an ERED-mediated asymmetric reduction of a fluoroenone provides a highly effective approach to the synthesis of enantioenriched alkyl fluorides, which does not require the use of expensive catalysts or low-abundance transition metals. In order to evaluate the efficiency of our method for the preparation of enantioenriched alkyl fluorides, we compared the reaction efficiency with alternative approaches using selected green metrics, with the caveat that comparison of such metrics for small scale unoptimized procedures should be considered as only indicative (Table 1).

**Table 1:** Comparison of three gre**e**n metrics for three representative asymmetric preparations of α-fluoroketones

	Organocatalysis^22^	Hydrogenation^34^	Biocatalysis (this work)
Fluoroketone (scale)	10a (2 mmol)	2a (0.05 mmol)	2a (0.79 mmol)
*E*-factor,^65^^a^	65	96	**59** b
(lowest value best)			
Process Mass Intensity (PMI),^66^^a^	**66**	97	714^b^
(lowest value best)			
Ecoscale^67^	42	68	**79**
(score out of 100)			
Comments	99 : 1 er, 55% yield; chromatography used for purification	95 : 5 er, 96% yield; CH_2_Cl_2_ used as solvent;^c^ silica gel purification used	99 : 1 er, 86% yield; pure after liquid–liquid extraction

a Mass-based metrics were calculated based on the reaction procedure only, excluding any workup or chromatography.

b The use of water as a solvent is generally not included in calculation of *E*-factor but is typically incorporated into PMI calculations; this accounts for the large difference in these values.

c The EcoScale metric incorporates penalties for the use of flammable organic solvents but does not appear to have any penalties for the use of halogenated solvents such as CH_2_Cl_2_, even though they would be considered unsuitable by most chemists for sustainable large-scale procedures.

Two established literature methods were selected for comparison, an asymmetric organocatalytic synthesis of ketone 10a,^22^ and an asymmetric hydrogenation used to prepare ketone 7a.^34^ Three green metrics were selected for comparison: *E*-factor, the total amount of waste (g) produced per g of product;^65^ Process Mass Intensity (PMI), the total material input (g) per g of product obtained;^66^ and EcoScale, a convenient scoring metric which assesses the reaction yield as well as the materials used and their safety/environmental profiles (with 100 indicating an ideal process, and lower scores indicative of the degree of safety/environmental hazards).^67^ Our method compares very favourably in terms of the *E*-factor and EcoScale metrics and the former value is comparable with processes typically employed in larger scale syntheses of pharmaceutical intermediates. The PMI value is very large in our biocatalytic reduction due to the large amount of water used as a solvent in the current process, though we anticipate that this could readily be reduced *via* further optimisation or reaction engineering (*e.g.* immobilisation of the enzyme, flow systems, and/or the use of mechanoenzymatic approaches). Finally, it should be noted that the fluoride product obtained from our scale-up reaction did not require silica gel purification after workup offering another advantage over the other methods. Overall, this preliminary assessment of metrics suggests that our method has the potential to offer considerable advantages in terms of lower environmental impact and greater efficiency over other approaches, though further optimisation and adaption of the procedures will be necessary for scale-up to multigram and ultimately kilo-scale syntheses.

## Conclusions

In conclusion, a novel approach to enantioenriched sp^3^ fluorides has been developed, *via* the bioreduction of α-fluoroenones and α-fluoroenoates. Notably, this provides access to an array of functionalized chiral fluorinated compounds in good to excellent yields and stereoselectivities. In the case of α-fluoroenones, the *Z* and *E* isomers of the enone give rise to the two enantiomeric products; with α-fluoroenoates only the *E* isomer is reduced by the enzyme, enabling alkene mixtures to be employed as substrates. The observed substrate behavior was rationalized by docking studies of a range of reactive and unreactive substrates into the active site, with only the former binding in a conformation suitable for productive hydride and proton transfer in a *trans* fashion across the fluoroalkene unit.

## Author contributions

Conceptualization: HCH, TDS. Formal analysis: All. Funding acquisition: HCH, TDS. Investigation: HA, YW, BW, AK, AEA, RS, VL, CEC. Methodology: HA, YW, BW, AK, AEA, JMW, JWEJ, HCH, TDS. Project Administration: HCH, TDS. Supervision: JMW, JWEJ, HCH, TDS. Validation: HA, YW, BW, AEA, HCH, TDS. Visualisation: HA, YW, HCH, TDS. Writing – original draft: HA, HCH, TDS. Writing – review & editing: HA, YW, BW, HCH, TDS.

## Conflicts of interest

There are no conflicts of interest to declare.

## Supplementary Material

GC-028-D6GC00545D-s001

## Data Availability

Supplementary information (SI): experimental details, materials and methods, co-solvent screening, docking studies, determination of stereoselectivity, HPLC traces and ^1^H, ^13^C, ^19^F, ^31^P NMR spectra for all compounds are available in the SI information (PDF). See DOI: https://doi.org/10.1039/d6gc00545d. The authors have cited additional references within the SI.^68–150^
