# Supplementary material for: Asymmetric synthesis of alkyl fluorides via biocatalytic reduction of α-fluoroenones and α-fluoroenoates
Source: Green Chem. 2026 May 13;28(21):9078–90. doi: 10.1039/d6gc00545d (PMC13182226; doi:10.1039/d6gc00545d)
Supplement: GC-028-D6GC00545D-s001 [file GC-028-D6GC00545D-s001.pdf]

Supplementary Information (SI) for Green Chemistry.
This journal is © The Royal Society of Chemistry 2026

# *Supporting Information*

# Asymmetric Synthesis of Alkyl Fluorides via Biocatalytic Reduction of α-Fluoroenones and α-Fluoroenoates

Helen Allan,[+1] Yu Wang,[+1] Bethan Winterson,[1] Alexandra King,[1] Abil E. Aliev,[1] Rachel Szpara,[1] Victor Laserna,[1] Charlotte E. Coomber,[1] John M. Ward,[2] Jack W. E. Jeffries,[2] Helen C. Hailes,*[1] Tom D. Sheppard*[1]

h.c.hailes@ucl.ac.uk; tom.sheppard@ucl.ac.uk

[1]Department of Chemistry, University College London, 20 Gordon Street, London, WC1H 0AJ, UK

[2]Department of Biochemical Engineering, University College London, London, WC1E 6BT, UK

Contents

General Experimental ........ 7
HPLC and GC Analysis ........ 7
HWE Reagent Synthesis ........ 8
α-Fluoroenones (*E* and *Z*) (1,2,3,4,5) ........ 11
General Procedure A:[56] ........ 11
α-Fluoroketone (Reference Standards, 7-12) ........ 22
General Procedure B:[87] ........ 22
General Procedure C:[88,83,58] ........ 22
(*Z*) α-Fluoroenoates (6) ........ 38
General Procedure D:[62] ........ 38
(*E*) α-Fluoroenoates (6) ........ 40
General Procedure E:[73] ........ 40
α-Fluoroesters (Reference Standards, 12) ........ 43
General Procedure F:[63] ........ 43
Biological details ........ 46
Enzyme expression ........ 47
Enzyme reactions ........ 48
Kinetics study ........ 48
Co-solvent screening ........ 50
Enzyme screening ........ 51
Compounds not accepted by the enzymes ........ 52
Preparative Scale Reactions & Green Metrics ........ 53
Post-Modification (13, 14, 15) ........ 61
Cascade Reaction ........ 64
NMR analysis of compound 15 diastereomers ........ 65
Docking Studies ........ 79
Preparing your protein ........ 79
Preparing the ligand ........ 79
Using Autodock Vina ........ 79
Viewing the results ........ 79
Additional Docking Studies ........ 82
Mutant design for (*E*)-1e ........ 83
Determination of Absolute Stereochemistry ........ 86
Confirmation of Bio-reduction Stereochemistry ........ 89
Synthesis ........ 89

Enzyme Reactions ........ 89
NMR Analysis ........ 91
References........ 99
HPLC and GC Traces ........ 107
3-Fluoro-4-phenylbutan-2-one (7a) ........ 107
3-Fluoro-4-(4-trifluoromethylphenyl)-butan-2-one (7b) ........ 109
3-Fluoro-4-(4-methoxyphenyl)-butan-2-one (7c) ........ 111
3-Fluoro-4-(4-cyanophenyl)-butan-2-one (7d) ........ 113
3-Fluoro-4-(o-tolyl)butan-2-one (7e) ........ 115
3-Fluoro-4-(3-trifluoromethylphenyl)-butan-2-one (7f) ........ 118
3-Fluoro-4-(pyridin-3-yl)butan-2-one (8a) ........ 120
3-Fluoro-4-(thiophen-2-yl)butan-2-one (8b)........ 122
4-Cyclohexyl-3-fluorobutan-2-one (9a) ........ 123
3-Fluoro-4-phenylbutan-2-one (9b) ........ 125
3-Fluoro-6-phenylpentan-2-one (9c) ........ 126
3-Fluoro-6-phenylhexan-2-one (9d)........ 127
2-Fluorocyclohexan-1-one (10a)........ 128
2-Fluorocyclohept-1-one (10b) ........ 129
2-Fluoro-1-phenylpentan-3-one (11a)........ 130
2-Fluoro-1-phenylheptan-1-one (11b)........ 132
Methyl 2-fluoro-3-phenylpropanoate (12a) ........ 133
Methyl 2-fluoro-3-(4-trifluoromethylphenyl)propanoate (12b)........ 135
Methyl 2-fluoro-3-(4-methoxyphenyl)propanoate (12c)........ 137
Ethyl 2-fluoro-3-(4-trifluoromethylphenyl)propanoate (12d) ........ 139
Methyl 2-fluoroheptanoate (12e)........ 141
(*R*)-(2-fluoro-3-methylbut-3-en-1-yl)benzene (13) ........ 142
(*R*)-3-fluoro-2-methyl-4-phenylbutan-2-ol (14) ........ 143
(2*R*,3*S*)-3-Fluoro-4-phenylbutan-2-ol (15) Enzyme reduction ........ 144
3-Fluoro-4-phenylbutan-2-ol (15) $NaBH_4$ reduction ........ 145
NMR Spectra ........ 146
Diethyl(1-fluoro-2-oxopropyl)phosphonate ........ 146
Diethyl(1-fluoro-2-oxobutyl)phosphonate........ 148
(Diethoxyphosphoryl)fluoroacetic acid........ 150
Diethyl(1-fluoro-2-oxo-2-phenylethyl)phosphonate ........ 152
Diethylmethyl-1-fluoromethylphosphonocarboxylate ........ 154
4-(2-Methylphenyl)butan-2-one ........ 156

(*E*)-4-Cyclohexylbut-3-en-2-one ........ 157
4-Cyclohexylbutan-2-one ........ 158
2-Chloro-2-fluorocycloheptan-1-one ........ 161
(*E*/*Z*)-5-Phenylpent-3-en-2-one ........ 163
5-Phenylpentan-2-one ........ 164
(*E*)-6-Phenylhex-3-en-2-one ........ 165
6-Phenylhexan-2-one ........ 166
Allyl 3-oxopentanoate ........ 167
Allyl 3-oxo-2-(4-(trifluoromethyl)benzyl)butanoate (S1-7b) ........ 168
Allyl 3-oxo-2-(4-methoxybenzyl)butanoate (S1-7c) ........ 169
Allyl 3-oxo-2-(4-cyanobenzyl)butanoate (S1-7d) ........ 170
Allyl 3-oxo-2-(3-(trifluoromethyl)benzyl)butanoate (S1-7f) ........ 171
Allyl 2-benzyl-3-oxopentanoate (S1-11a) ........ 172
Allyl 2-fluoro-3-oxo-2-(4-(trifluoromethyl)benzyl)butanoate (S2-7b) ........ 173
Allyl 2-fluoro-3-oxo-2-(4-methoxybenzyl)butanoate (S2-7c) ........ 174
Allyl 2-fluoro-3-oxo-2-(4-cyanobenzyl)butanoate (S2-7d) ........ 176
Allyl 2-fluoro-3-oxo-2-(3-(trifluoromethyl)benzyl)butanoate (S2-7f) ........ 177
Allyl 2-benzyl-2-fluoro-3-oxopentanoate (S2-11a) ........ 179
Ethyl (*Z*)-2-fluoro-3-phenylacrylate ((*Z*)-S3-6a) ........ 180
Ethyl (*Z*)-2-fluoro-3-(4-trifluoromethylphenyl)acrylate ((*Z*)-S3-6b) ........ 182
Ethyl (*Z*)-2-fluoro-3-(4-methoxyphenyl)acrylate ((*Z*)-S3-6c) ........ 183
(*Z*)-2-Fluoro-1-phenylbut-1-ene-3-one ((*Z*)-1a) ........ 185
(*E*)-2-Fluoro-1-phenylbut-1-ene-3-one ((*E*)-1a) ........ 186
(*Z*)-3-Fluoro-4-(4-trifluoromethylphenyl)-3-buten-2-one ((*Z*)-1b) ........ 188
(*E*)-3-Fluoro-4-(4-trifluoromethylphenyl)-3-buten-2-one ((*E*)-1b) ........ 189
(*Z*)-3-Fluoro-4-(4-methoxyphenyl)-3-buten-2-one ((*Z*)-1c) ........ 191
(*E*)-3-Fluoro-4-(4-methoxyphenyl)-3-buten-2-one ((*E*)-1c) ........ 192
(*Z*)-3-Fluoro-4-(4-cyanophenyl)-3-buten-2-one ((*Z*)-1d) ........ 194
(*E*)-3-Fluoro-4-(4-cyanophenyl)-3-buten-2-one ((*E*)-1d) ........ 195
(*Z*)-3-Fluoro-4-(2-methylphenyl)-3-buten-2-one ((*Z*)-1e) ........ 197
(*E*)-3-Fluoro-4-(2-methylphenyl)-3-buten-2-one ((*E*)-1e) ........ 198
(*Z*)-3-Fluoro-4-(3-trifluoromethylphenyl)-3-buten-2-one ((*Z*)-1f) ........ 200
(*E*)-3-Fluoro-4-(3-trifluoromethylphenyl)-3-buten-2-one ((*E*)-1f) ........ 201
(*Z*)-3-Fluoro-4(pyridine-3-yl)but-3-en-one ((*Z*)-2a) ........ 203
(*E*)-3-Fluoro-4-(pyridin-3-yl)but-3-en-2-one ((*E*)-2a) ........ 204
(*E*)-3-Fluoro-4-(thiophen-2-yl)but-3-en-2-one ((*E*)-2b) ........ 206

(*Z*)-3-Fluoro-4-cyclohexylbut-3-en-2-one ((*Z*)-3a) ... 207
(*E*)-3- Fluoro-4-cyclohexylbut-3-en-2-one ((*E*)-3a) ... 209
(*Z*)-3-Fluorooct-3-en-2-one ((*Z*)-3b) ... 210
(*E*)-3-Fluorooct-3-en-2-one ((*E*)-3b) ... 212
(*E*)-3-Fluoro-5-phenylpent-3-en-2-one ((*E*)-3c) ... 213
(*E*)- 3-Fluoro-6-phenylhex-3-en-2-one ((*E*)-3d) ... 215
(*E*)-2-Fluorocyclohex-2-en-1-one ((*E*)-4a) ... 216
(*E*)-2-Fluorocyclohept-2-en-1-one ((*E*)-4b) ... 218
(*Z*)-2-Fluoro-1-phenylpent-1-en-3-one ((*Z*)-5a) ... 220
(*E*)-2-Fluoro-1-phenylpent-1-en-3-one ((*E*)-5a) ... 221
(*Z*)-2-Fluoro-1-phenylhept-2-en-1-one ((*Z*)-5b) ... 223
(*E*)-2-Fluoro-1-phenylhept-2-en-1-one ((*E*)-5b) (impure) ... 224
(*E*)-3-Fluoro-4-phenylbut-3-en-2-one-4-d ((*E*)-4-D-1a) ... 227
3-Fluoro-4-phenylbutan-2-one (7a) ... 228
3-Fluoro-4-(4-trifluoromethylphenyl)-butan-2-one (7b) ... 230
3-Fluoro-4-(4-methoxyphenyl)-butan-2-one (7c) ... 231
3-Fluoro-4-(4-cyanophenyl)-butan-2-one (7d) ... 233
3-Fluoro-4-(2-methylphenyl)-butan-2-one (7e) ... 234
3-Fluoro-4-(3-trifluoromethylphenyl)-butan-2-one (7f) ... 236
3-Fluoro-4-(pyridin-3-yl)butan-2-one (8a) ... 237
3-Fluoro-4-(thiophen-2-yl)butan-2-one (8b) ... 239
4-Cyclohexyl-3-fluorobutan-2-one (9a) ... 240
3-Fluoro-4-phenylbutan-2-one (9b) ... 242
3-Fluoro-5-phenylpentan-2-one (9c) ... 243
3-Fluoro-6-phenylhexan-2-one (9d) ... 245
2-Fluorocyclohexan-1-one (10a) ... 246
2-Fluorocycloheptan-1-one (10b) ... 248
2-Fluoro-1-phenylpentan-3-one (11a) ... 249
2-Fluoro-1-phenylheptan-1-one (11b) ... 251
(3*S*,4*R*)-3-Fluoro-4-phenylbutan-2-one-4-d ((3*R*,4*S*)-7a) ... 252
(3*S*,4*S*)-3-Fluoro-4-phenylbutan-2-one-4-d ((3*S*,4*S*)-7a) ... 254
Methyl (*Z*)-2-fluoro-3-phenylacrylate ((*Z*)-6a) ... 256
Methyl (*E*)-2-fluoro-3-phenylacrylate ((*E*)-6a) (8:1 mix with (*Z*)-6a) ... 257
Methyl (*Z*)-2-fluoro-3-(4-trifluoromethylphenyl)acrylate ((*Z*)-6b) ... 259
Methyl (*E*)-2-fluoro-3-(4-trifluoromethylphenyl)acrylate ((*E*)-6b) ... 260
Methyl (*Z*)-2-fluoro-3-(4-methoxyphenyl)acrylate ((*Z*)-6c) ... 262

Methyl (*E*)-2-fluoro-3-(4-methoxyphenyl)acrylate ((*E*)-6c) (5:1 mix with (*Z*)-3c) .......... 264
Ethyl (*E*)-2-fluoro-3-(4-trifluoromethylphenyl)acrylate ((*E*)-6d) .......... 265
Methyl (*E*)-2-fluorohept-2-enoate ((*E*)-6e) (13:1 mix with (*Z*)-6e).......... 267
Methyl 2-fluoro-3-phenylpropanoate (12a) .......... 268
Methyl 2-fluoro-3-(4-trifluoromethylphenyl)propanoate (12b).......... 270
Methyl 2-fluoro-3-(4-methoxyphenyl)propanoate (12c).......... 271
Ethyl 2-fluoro-3-(4-trifluoromethylphenyl)propanoate (12d) .......... 273
Methyl 2-fluoroheptanoate (12e).......... 274
(2-Fluoro-3-methylbut-3-en-1-yl)benzene (13) .......... 276
3-Fluoro-2-methyl-4-phenylbutan-2-ol (14) .......... 277
(2*S*,3*R*),(2*R*,3*R*)-3-Fluoro-4-phenylbutan-2-ol (15).......... 279

# General Experimental

All commercially available chemicals and solvents were used as received without any further purification. Reactions were performed at room temperature using flame dried glassware under Ar unless otherwise stated and are unoptimized. Reactions were monitored by thin layer chromatography (TLC) on aluminium plates coated with silica gel 60 F254 (Merck KGaA). Detection was provided by short and long range UV lamp (254 and 365 nm respectively), as well as $KMnO_4$ or anisaldehyde staining. Flash column chromatography was performed using Geduran® silicagel 60 (40 – 63 µm). Melting points were noted with a Gallenkamp Melting Point apparatus and are uncorrected. Infrared spectra were obtained on a Bruker Alpha II compact FTIR Spectrometer operating in ATR mode. High and low resolution mass spectrometry were obtained using a Thermo Vanquish LC connected to Q Exactive Plus Hybrid Quadrupole-Orbitrap mass spectrometer operating in ESI mode or Thermo Scientific Trace 1310 GCMS connected to an ISQ single quadrupole mass spectrometer operating in EI mode as specified. NMR spectra were recorded on Bruker Avance Neo 700, Avance III 600, Avance Neo 500 or Avance III 400 spectrometers as stated. $^{1}H$ and $^{13}C$ spectra are expressed in parts per million (ppm) with known residual solvent peaks employed as reference: $CDCl_3$ (δ = 7.27 ($^{1}H$), 77.1 ppm ($^{13}C$)); DMSO (δ = 2.50 ($^{1}H$), 39.5 ppm ($^{13}C$)). Coupling constants (*J*) are quoted in Hertz and multiplicities for couplings are shown as s (singlet), d (doublet), t (triplet), q (quartet), m (multiplet) and combinations thereof.

## HPLC and GC Analysis

Chiral HPLC analysis was performed using an Agilent 1260 series HPLC equipped with G7167A Infinity II multisampler, G7112B binary pump, G7165A variable wavelength detector, OpenLab CDS Workstation, and a Chiralpak AD-H column, OD-H column or OD-J (5 µm particle size, 4.5 mm × 250 mm) as stated. Stock solutions of compounds at 5 mM concentration were made up, these were diluted to 0.5 mM and passed through a 0.45 µm PTFE syringe filter before use. Standard conditions were 1 $mL/min^{-1}$ flow rate, 1% IPA/hexane, 10 µL injection volume, detection at 214 nm, column temp. 25 °C.

Gas chromatography (GC) analysis was performed using Agilent Technologies 7820A (G4350) GC System with OpenLAB CDS Chemstation software, Supelco Beta Dex 225 capillary GC column (30 m × 250 µm, 0.25 µm) and flame ionization detector at 300 °C. The samples (1 µL) were injected with the autosampler tower G4567A at an injection temperature of 250 °C and applied by split injection (ratio 50:1) and a split flow of 36 mL/min.

## HWE Reagent Synthesis

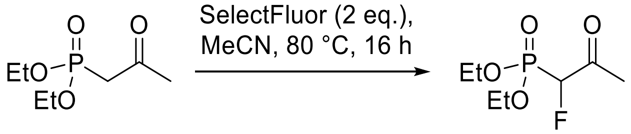


*Diethyl (1-fluoro-2-oxopropyl)phosphonate* was synthesised following the procedure of Radwan-Olszewska *et al.*[68] A solution of diethyl (2-oxopropyl)phosphonate (2.00 g, 10.3 mmol) in MeCN (172 mL) was treated with Selectfluor (7.27 g, 20.6 mmol) and stirred at rt for 5 min. After this time the solution was heated to 80 °C for 16 h. After cooling to rt the mixture was taken up in $Et_2O$ (200 mL, washed $NH_4Cl_{(sat.\ aq.)}$ (150 mL), brine (150 mL) and conc. *in vacuo*, azeotrope with PhMe (50 mL). Purification was achieved using flash column chromatography (50% EtOAc/cyclohexane) to give diethyl (1-fluoro-2-oxopropyl)phosphonate as a colourless oil (1.11 g, 51%; Lit.[68] 62%). $^1$H NMR (700 MHz, $CDCl_3$) δ 5.13 (1H, dd, *J* = 48.0, 14.2 Hz), 4.25-4.22 (4H, m), 2.37 (3H, d, *J* = 4.3 Hz), 1.38-1.35 (6H, m); $^{13}$C NMR (176 MHz, $CDCl_3$) δ 200.8 (C, d, *J* = 20.3 Hz), 91.8 (CH, dd, *J* = 197.7, 152.7 Hz), 64.3 ($CH_2$, t, *J* = 14.5 Hz), 26.9 ($CH_3$, s), 16.3 ($CH_3$, dd, *J* = 5.9, 2.6 Hz); $^{19}$F NMR (376 MHz, $CDCl_3$) δ −207.3 (d, *J* = 71.2 Hz); $^{31}$P (283 MHz, $CDCl_3$) δ 9.8-10.2 (m); m/z [HRMS, ESI] Calcd. $(C_7H_{14}FO_4P+H)^+$ 213.0686, found 213.0685. Data in agreement with that reported in the literature.[64]

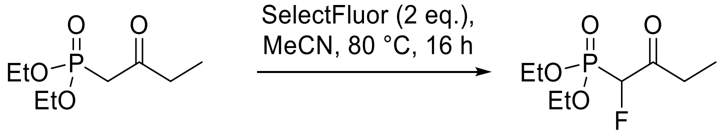


*Diethyl(1-fluoro-2-oxobutyll)phosphonate* was synthesised following an analogous procedure to that of Radwan-Olszewska *et al.*[68] using diethyl(2-oxobutyl)phosphonate (2.00 g, 9.61 mmol). Purification was achieved using flash column chromatography (40-100% EtOAc/pentane) to give diethyl (1-fluoro-2-oxobutyl)phosphonate as a pale yellow oil (775 mg, 36%); $^1$H NMR (700 MHz, $CDCl_3$) δ 5.16 (1H, dd, *J* = 47.8, 14.2 Hz), 4.26-4.20 (4H, m), 2.79-2.66 (2H, m), 1.36 (6H, dt, *J* = 10.3, 7.2 Hz), 1.10 (3H, *t*, *J* = 7.2 Hz); $^{13}$C NMR (176 MHz, $CDCl_3$) δ 203.7 (C, d, *J* = 19.4 Hz), 91.7 (CH, dd, *J* = 197.5, 153.0 Hz), 64.3 ($CH_2$, t, *J* = 6.3 Hz), 32.5 ($CH_2$, s), 16.4 ($CH_3$, dd, *J* = 4.4, 2.7 Hz), 6.8 ($CH_3$, d, *J* = 2.0 Hz); $^{19}$F NMR (659 MHz, $CDCl_3$) δ −209.8 (dd, *J* = 71.5, 47.8 Hz); $^{31}$P (283 MHz, $CDCl_3$) δ 10.3 (d, *J* = 71.5 Hz); m/z [HRMS, ESI] Calcd. $(C_8H_{16}FO_4P+H)^+$ 227.0843, found 227.0835. Data in agreement with that reported in the literature ($^{19}$F and $^{31}$P NMR not previously reported).[56]

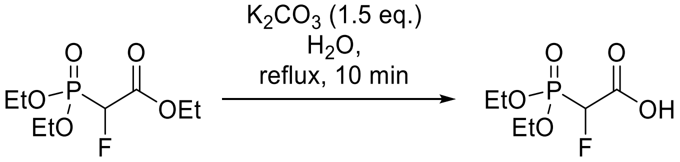


*(Diethoxyphosphoryl)fluoroacetic acid* was synthesised following the procedure of Coutrot *et al.*[69] A solution of 2-fluorotriethylphosphonoacetate (1.00 g, 4.12 mmol) in $H_2O$ (6 mL) was treated with $K_2CO_3$ (856 mg, 6.20 mmol) and the resultant solution heated to reflux for 10 min (reaction performed under air). After this time the solution was cooled to rt, extracted with $Et_2O$ (5 mL) and the aqueous layer acidified to pH 1 with HCl (4 M). The aqueous layer was saturated with NaCl, extracted with $CH_2Cl_2$ (5 x 20 mL) and conc. *in vacuo*. PhMe (10 mL) was added to azeotrope any residual water and conc. *in vacuo* to give the title compound as a white solid (752 mg, 85%; Lit.[69] 73%). m.p. 74-76 °C (Lit.[69] 70-74°C); $^1$H NMR (400 MHz, $CDCl_3$) δ 7.64 (1H, br s), 5.26 (1H, dd, *J* = 47.2, 13.6 Hz), 4.39-4.28 (4H, m), 1.43-1.38 (6H, m); $^{13}$C NMR (176 MHz, $CDCl_3$) δ 165.7 (C, dd, *J* = 21.8, 15.1 Hz), 84.6 (CH, dd, *J* = 194.7, 158.4 Hz), 65.2 ($CH_2$, dd, *J* = 42.4, 6.7 Hz), 16.3 ($CH_3$, d, *J* = 5.6 Hz); $^{19}$F NMR (659 MHz, $CDCl_3$) δ −210.0 (dd, *J* = 72.3, 47.2 Hz); $^{31}$P (162 MHz, $CDCl_3$) δ 11.5 (d, *J* = 72.3 Hz); m/z [HRMS, ESI] Calcd. $(C_6H_{12}FO_5+H)^+$ 215.0476, found 215.0479. Data in agreement with that reported in the literature.[70]

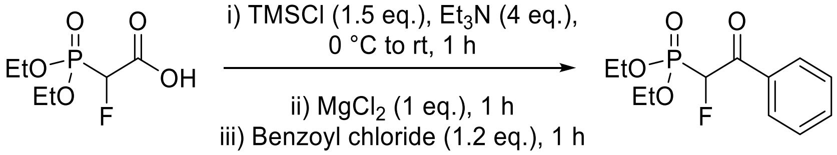


*Diethyl (1-fluoro-2-oxo-2-phenylethyl)phosphonate* was synthesised following the procedure of Kim *et al.*[71] A solution of (diethoxyphosphoryl)fluoroacetic acid (392 mg, 2.00 mmol) in PhMe (5 mL) was cooled to 0 °C and treated with TMSCl (0.380 mL, 3.00 mmol) followed by $Et_3N$ (1.12 mL, 8.00 mmol) and the resultant solution allowed to warm to rt over 1 h. After this time $MgCl_2$ (190 mg, 2.00 mmol) was added and stirring continued for 1 h. Finally benzoyl chloride (0.280 mL, 2.40 mmol) was added and the resultant solution stirred for 1 h, quenched with $NH_4Cl_{(sat.\ aq.)}$ (2 mL), extracted with $Et_2O$ (2 x 15 mL), dried ($MgSO_4$), filtered and conc. *in vacuo*. Purification *via* flash column chromatography (20-80% EtOAc/cyclohexane) gave the title compound as a yellow oil (164 mg, 30%; Lit.[71] 80%). $^1$H NMR (700 MHz, $CDCl_3$) δ 8.04 (2H, d, *J* = 7.8 Hz), 7.63 (1H, t, *J* = 7.4 Hz), 7.50 (2H, app t, *J* = 7.4 Hz), 6.00 (1H, dd, *J* = 47.2, 13.4 Hz), 4.29-4.14 (4H, m), 1.35 (3H, t, *J* = 7.1 Hz), 1.27-1.25 (3H, m); $^{13}$C NMR (176 MHz, $CDCl_3$) δ 191.3 (C, d, *J* = 16.5 Hz), 134.4 (CH, s), 129.5 (CH, d, *J* = 3.1 Hz), 128.9 (C, t, *J* = 66.3 Hz), 128.7 (CH, s), 90.4 (CH, dd, *J* = 196.1, 152.4 Hz), 64.41 ($CH_2$, d, *J* = 6.7 Hz), 64.37 ($CH_2$, d, *J* = 6.7 Hz), 16.4 ($CH_3$, d, *J* = 5.8 Hz), 16.3 ($CH_3$, d, *J* = 5.8 Hz); $^{19}$F NMR (659 MHz, $CDCl_3$) δ −207.7 (dd, *J* = 72.0, 47.2 Hz); $^{31}$P (283 MHz, $CDCl_3$) δ 10.0 (d, *J* = 72.0 Hz); m/z [HRMS, ESI] Calcd. $(C_{12}H_{16}FO_4+H)^+$ 275.0843, found 275.0839. Data in agreement with that reported in the literature.[72]

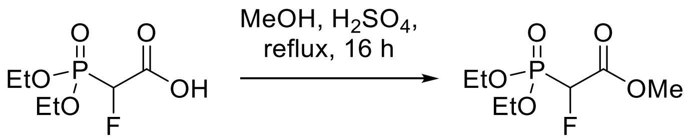


*Diethylmethyl-1-fluoromethylphosphonocarboxylate*:[73] A solution of (diethoxyphosphoryl)fluoroacetic acid (1.15 g, 5.37 mmol) in MeOH (6 mL) was treated with $H_2SO_4$ (29 µL, 5.50 mmol) and the resultant solution heated at reflux for 16 h (reaction performed under air). After this time, the reaction was cooled to rt, quenched with $H_2O$ (1 mL), extracted with $Et_2O$ (3x10 mL), dried ($Na_2SO_4$), filtered and conc. *in vacuo* to give the title compound as a colourless oil (1.12 mg, 92%). $^1H$ NMR (700 MHz, $CDCl_3$) δ 5.23 (1H, dd, *J* = 47.0, 12.6 Hz), 4.29-4.24 (4H, m), 3.89 (3H, s), 1.40-1.36 (6H, m); $^{13}C$ NMR (176 MHz, $CDCl_3$) δ 165.4 (C, dd, *J* = 22.2, 0.9 Hz), 85.1 (CH, dd, *J* = 195.4, 157.6 Hz), 64.5 ($CH_2$, dd, *J* = 15.2, 6.7 Hz), 53.2 ($CH_3$, s), 16.3 ($CH_3$, d, *J* = 5.7 Hz); $^{19}F$ NMR (659 MHz, $CDCl_3$) δ −210.5 (dd, *J* = 71.0, 47.0 Hz); $^{31}P$ (283 MHz, $CDCl_3$) δ 9.8 (d, *J* = 71.0 Hz); m/z [HRMS, ESI] Calcd. $(C_7H_{14}FO_5P+H)^+$ 229.0656, found 229.0627. Data in agreement with that reported in the literature.[74]

## α-Fluoroenones (*E* and *Z*) (1,2,3,4,5)

### General Procedure A:[56]

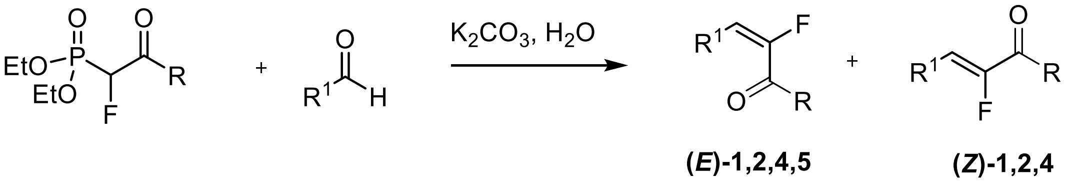


Following the procedure of Coutrot *et al.*:[56] In an Eppendorf tube, a mixture of phosphonate (1.2 eq.) and aldehyde (1 eq.) was treated with $K_2CO_3$ solution (50% wt solution, 2 eq.). An exotherm was observed and the solution shaken for 25 min. After this time the reaction was diluted with $H_2O$ (4 mL/mmol), extracted into EtOAc (2 x 25 mL/mmol), dried ($MgSO_4$), filtered and conc. *in vacuo*. Purification was achieved by flash column chromatography, *E* and *Z* isomers were separable and isolated as stated.

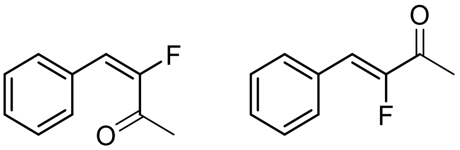


*3-Fluoro-4-phenyl-3-buten-2-one* (**1a**): General Procedure A. Reaction performed using benzaldehyde (40 µL, 0.39 mmol) and diethyl (1-fluoro-2-oxopropyl)phosphonate, subsequent purification *via* flash column chromatography (5% EtOAc/cyclohexane) gave *E*-**1a** as a pale-yellow oil (24 mg, 38%) and *Z*-**1a** as white solid (19 mg, 30%).

(*E*)-**1a**: $\upsilon_{max}$ (oil) 1704 (m), 1691 (m) cm$^{-1}$; $^1$H NMR (700 MHz, $CDCl_3$) δ 7.60-7.59 (2H, m), 7.38-7.35 (3H, m), 6.74 (1H, d, *J* = 24.6 Hz), 2.32 (3H, d, *J* = 4.9 Hz); $^{13}$C NMR (176 MHz, $CDCl_3$) δ 192.9 (C, d, *J* = 38.3 Hz), 153.2 (C, d, *J* = 257.2 Hz), 131.0 (C, d, *J* = 10.2 Hz), 130.1 (CH, d, *J* = 2.8 Hz), 129.4 (CH, s), 128.2 (CH, s), 120.1 (CH, d, *J* = 27.6 Hz), 28.4 ($CH_3$, s); $^{19}$F NMR (659 MHz, $CDCl_3$) δ −113.0 (dq, *J* = 24.6, 4.9 Hz); *m/z* [LRMS, ESI] (relative intensity, M) 165 (100, $[M+H]^+$); [HRMS, ESI] Calcd. $(C_{10}H_9FO+H)^+$ 165.0710, found 165.0713. Data in agreement with that reported in the literature where **1a** was isolated as a mix of isomers.[56]

(*Z*)-**1a**: m.p. 52-53 °C (Lit.[75] 54 °C); $^1$H NMR (700 MHz, $CDCl_3$) δ 7.69-7.67 (2H, m), 7.43-7.40 (3H, m), 6.83 (1H, d, *J* = 36.4 Hz), 2.43 (3H, d, *J* = 3.4 Hz); $^{13}$C NMR (176 MHz, $CDCl_3$) δ 192.5 (C, d, *J* = 33.3 Hz), 154.1 (C, d, *J* = 271.9 Hz), 131.2 (C, d, *J* = 4.0 Hz), 130.8 (CH, d, *J* = 8.4 Hz), 130.0 (CH, d, *J* = 2.7 Hz), 129.0 (CH, s), 115.7 (CH, d, *J* = 5.4 Hz), 25.8 ($CH_3$, s); $^{19}$F NMR (659 MHz, $CDCl_3$) δ −123.6 (dq, *J* = 36.4, 3.4 Hz); m/z [HRMS, ESI] Calcd. $(C_{10}H_9FO+H)^+$ 165.0710, found 165.0706. Data in agreement with that reported in the literature.[76]

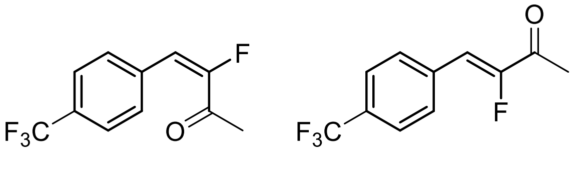


*3-Fluoro-4-(4-trifluoromethylphenyl)-3-buten-2-one* (**1b**): General Procedure A. Reaction performed using 4-(trifluoromethyl)benzaldehyde (53 µL, 0.39 mmol) and diethyl (1-fluoro-2-oxopropyl)phosphonate, subsequent

purification *via* flash column chromatography (10% EtOAc/cyclohexane) gave *E*-**1b** as a colourless semi-solid (28 mg, 35%) and *Z*-**1b** as a white solid (22 mg, 24%).

(*E*)-**1b**: $^1$H NMR (700 MHz, $CDCl_3$) $\delta$ 7.68 (2H, d, *J* = 8.2 Hz), 7.62 (2H, d, *J* = 8.2 Hz), 6.69 (1H, d, *J* = 23.5 Hz), 2.37 (3H, d, *J* = 5.0 Hz); $^{13}$C NMR (176 MHz, $CDCl_3$) $\delta$ 192.8 (C, d, *J* = 39.4 Hz), 153.8 (C, d, *J* = 262.5 Hz), 134.6 (C, d, *J* = 10.8 Hz), 131.0 (C, q, *J* = 32.7 Hz), 130.3 (CH, d, *J* = 2.9 Hz), 125.2 (CH, q, *J* = 3.8 Hz), 124.0 (C, q, *J* = 272.1 Hz), 118.2 (CH, d, *J* = 28.3 Hz), 28.1 ($CH_3$, d, *J* = 2.0 Hz); $^{19}$F NMR (659 MHz, $CDCl_3$) $\delta$ −62.8 (3F, s), −110.6 (1F, dq, *J* = 23.5, 5.0 Hz); m/z [LRMS, EI] Calcd. $(C_{11}H_8F_4O)^+$ 232, found 232. Data in agreement with that reported in the literature.[77]

(*Z*)-**1b**: m.p. 63-64 °C (no lit. m.p. reported); $^1$H NMR (700 MHz, $CDCl_3$) $\delta$ 7.78 (2H, d, *J* = 8.2 Hz), 7.67 (2H, d, *J* = 8.2 Hz), 6.83 (1H, d, *J* = 35.6 Hz), 2.45 (3H, d, *J* = 3.3 Hz); $^{13}$C NMR (176 MHz, $CDCl_3$) $\delta$ 192.3 (C, d, *J* = 34.4 Hz), 154.9 (C, d, *J* = 276.7 Hz), 134.6 (C, d, *J* = 3.6 Hz), 131.4 (C, qd, *J* = 32.5, 2.4 Hz), 130.8 (CH, d, *J* = 8.4 Hz), 125.9 (CH, q, *J* = 3.7 Hz), 123.9 (C, q, *J* = 272.0 Hz), 113.6 (CH, d, *J* = 5.1 Hz), 25.9 ($CH_3$, s); $^{19}$F NMR (659 MHz, $CDCl_3$) $\delta$ −63.0 (3F, s), −120.8 (1F, dq, *J* = 35.6, 3.3 Hz); m/z [LRMS, EI] Calcd. $(C_{11}H_8F_4O)^+$ 232, found 232. Data in agreement with that reported in the literature.[78]

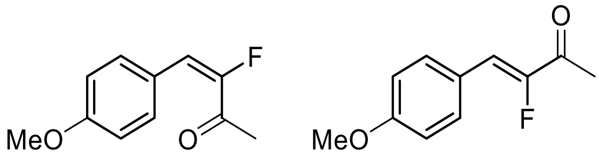


*3-Fluoro-4-(4-methoxyphenyl)-3-buten-2-one* (**1c**): General Procedure A. Reaction performed using 4-anisaldehyde (47 µL, 0.39 mmol) and diethyl (1-fluoro-2-oxopropyl)phosphonate, subsequent purification *via* flash column chromatography (10% EtOAc/cyclohexane) gave *E*-**1c** as a white solid (29 mg, 39%) and *Z*-**1c** as an off white solid (27 mg, 36%).

(*E*)-**1c**: m.p. 32-33 °C (Lit.[77] 41-43 °C); $^1$H NMR (700 MHz, $CDCl_3$) $\delta$ 7.68 (2H, d, *J* = 8.8 Hz), 6.89 (2H, d, *J* = 8.8 Hz), 6.65 (1H, d, *J* = 26.0 Hz), 3.84 (3H, s), 2.34 (3H, d, *J* = 5.2 Hz); $^{13}$C NMR (176 MHz, $CDCl_3$) $\delta$ 193.0 (C, d, *J* = 35.4 Hz), 160.8 (C, d, *J* = 1.7 Hz), 152.3 (C, d, *J* = 252.6 Hz), 132.3 (CH, d, *J* = 2.8 Hz), 123.3 (C, d, *J* = 10.2 Hz), 120.7 (CH, d, *J* = 28.8 Hz), 113.8 (CH, s), 55.4 ($CH_3$, s), 28.3 ($CH_3$, d, *J* = 1.8 Hz); $^{19}$F NMR (659 MHz, $CDCl_3$) $\delta$ −114.8 (dq, *J* = 26.0, 5.2 Hz); m/z [LRMS, EI] Calcd. $(C_{11}H_{11}FO_2)^+$ 194, found 194. Data in agreement with that reported in the literature.[77]

(*Z*)-**1c**: m.p. 67-68 °C (Lit.[77] 71-73 °C); $^1$H NMR (700 MHz, $CDCl_3$) $\delta$ 7.64 (2H, d, *J* = 8.8 Hz), 6.94 (2H, d, *J* = 8.8 Hz), 6.79 (1H, d, *J* = 36.8 Hz), 3.86 (3H, s), 2.41 (3H, d, *J* = 3.3 Hz); $^{13}$C NMR (176 MHz, $CDCl_3$) $\delta$ 192.3 (C, d, *J* = 32.5 Hz), 161.1 (C, d, *J* = 3.4 Hz), 153.3 (C, d, *J* = 267.7 Hz), 132.6 (CH, d, *J* = 8.5 Hz), 123.9 (C, d, *J* = 4.2 Hz), 115.9 (CH, d, *J* = 5.9 Hz), 114.5 (CH, s), 55.5 ($CH_3$, s), 25.7 ($CH_3$, s); $^{19}$F NMR (659 MHz, $CDCl_3$) $\delta$ −126.6 (dq, *J* = 36.8, 3.3 Hz); m/z [LRMS, EI] Calcd. $(C_{11}H_{11}FO_2)^+$ 194, found 194. Data in agreement with that reported in the literature.[77]

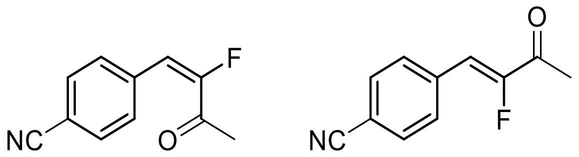

*3-Fluoro-4-(4-cyanophenyl)-3-buten-2-one* (**1d**): General Procedure A. Reaction performed using 4-cyanobenzaldehyde (51 mg, 0.39 mmol) and diethyl (1-fluoro-2-oxopropyl)phosphonate, subsequent purification *via* flash column chromatography (10% EtOAc/cyclohexane) gave *E*-**1d** as a white solid (31 mg, 42%) and *Z*-**1d** as a white solid (15 mg, 20%).

(*E*)-**1d**: m.p. 68-70 °C (Lit.[79] 85-87 °C); $^{1}$H NMR (700 MHz, $CDCl_3$) δ 7.67 (2H, d, *J* = 8.4 Hz), 7.64 (2H, d, *J* = 8.4 Hz), 6.64 (1H, d, *J* = 23.3 Hz), 2.38 (3H, d, *J* = 5.0 Hz); $^{13}$C NMR (176 MHz, $CDCl_3$) δ 192.9 (C, d, *J* = 39.7 Hz), 154.0 (C, d, *J* = 264.9 Hz), 135.7 (CH, d, *J* = 10.9 Hz), 132.0 (CH, s), 130.6 (C, d, *J* = 2.9 Hz), 118.6 (C, s), 113.0 (CH, d, *J* = 5.0), 112.6 (C, s), 28.1 ($CH_3$, d, *J* = 2.2 Hz); $^{19}$F NMR (659 MHz, $CDCl_3$) δ −109.2 (dq, *J* = 23.3, 5.0 Hz); m/z [LRMS, EI] Calcd. $(C_{11}H_8FNO)^+$ 189, found 189. Data in agreement with that reported in the literature.[79]

(*Z*)-**1d**: m.p. 118-120 °C (Lit.[78] 119-121 °C); $^{1}$H NMR (400 MHz, $CDCl_3$) δ 7.76 (2H, d, *J* = 8.3 Hz), 7.69 (2H, d, *J* = 8.3 Hz), 6.81 (1H, d, *J* = 35.2 Hz), 2.45 (3H, d, *J* = 3.5 Hz); $^{13}$C NMR (176 MHz, $CDCl_3$) δ 192.2 (C, d, *J* = 34.6 Hz), 155.2 (C, d, *J* = 279.0 Hz), 135.6 (C, d, *J* = 4.1 Hz), 132.6 (CH, s), 131.0 (CH, d, *J* = 8.6 Hz), 118.4 (C, s), 113.1 (C, d, *J* = 3.2), 113.0 (CH, d, *J* = 4.8 Hz), 27.9 ($CH_3$, s); $^{19}$F NMR (659 MHz, $CDCl_3$) δ −119.2 (dq, *J* = 35.2, 3.5 Hz); m/z [LRMS, EI] Calcd. $(C_{11}H_8FNO)^+$ 189, found 189. Data in agreement with that reported in the literature.[79]

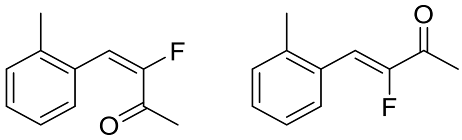

*3-Fluoro-4-(2-methylphenyl)-3-buten-2-one* (**1e**): General Procedure A. Reaction performed using 4-methylbenzaldehyde (164 µL, 1.41 mmol) and diethyl (1-fluoro-2-oxopropyl)phosphonate, subsequent purification *via* flash column chromatography (2-5% EtOAc/cyclohexane) gave *E*-**1e** as a colourless oil (40 mg, 16%, 9:1 E:Z) and *Z*-**1e** as a colourless oil (10 mg, 4%).

(*E*)-**1e**: ; $\upsilon_{max}$ (oil) 2925 (w), 1691 (s), 1632 (m) cm$^{-1}$; $^{1}$H NMR (700 MHz, $CDCl_3$) δ 7.30-7.27 (2H, m), 7.23 (1H, d, *J* = 7.6 Hz), 7.20 (1H, app t, *J* = 7.6 Hz), 6.87 (1H, d, *J* = 21.5 Hz), 2.30 (3H, s), 2.17 (3H, d, *J* = 3.8 Hz); $^{13}$C NMR (176 MHz, $CDCl_3$) δ 192.4 (C, d, *J* = 34.5 Hz), 153.4 (C, d, *J* = 258.7 Hz), 136.6 (C, d, *J* = 2.9 Hz), 130.8 (C, d, *J* = 9.6 Hz), 130.1 (CH, s), 129.8 (CH, d, *J* = 2.2 Hz), 129.1 (CH, s), 125.8 (CH, s), 118.8 (CH, d, *J* = 24.8 Hz), 28.3 ($CH_3$, s), 20.3 ($CH_3$, s); $^{19}$F NMR (659 MHz, $CDCl_3$) δ −114.5 (1F, dq, *J* = 21.5, 3.8 Hz); *m/z* [LRMS, ESI] (relative intensity, M) 179 (100, [M+H]+); m/z [HRMS, ESI] Calcd. $(C_{11}H_{11}FO+H)^+$ 179.0867, found 179.0865.

(*Z*)-**1e**: $\upsilon_{max}$ (oil) 2921 (w), 1688 (s), 1632 (s) cm$^{-1}$; $^{1}$H NMR (700 MHz, $CDCl_3$) δ 7.82 (1H, d, *J* = 7.5 Hz), 7.28-7.26 (2H, m), 7.25 (1H, app t, *J* = 7.5 Hz), 7.05 (1H, d, *J* = 35.8 Hz), 2.43 (3H, d, *J* = 3.5 Hz), 2.42 (3H, s); $^{13}$C NMR (176

MHz, $CDCl_3$) δ 192.7 (C, d, *J* = 34.5 Hz), 154.1 (C, d, *J* = 272.5 Hz), 138.2 (C, d, *J* = 2.9 Hz), 130.7 (C, s), 130.3 (CH, s), 130.2 (CH, s), 129.1 (CH, d, *J* = 3.5 Hz), 129.7 (CH, d, *J* = 4.0 Hz), 112.5 (CH, d, *J* = 5.2 Hz), 25.9 ($CH_3$, s), 20.3 ($CH_3$, s); $^{19}$F NMR (659 MHz, $CDCl_3$) δ −125.2 (dq, *J* = 35.8, 3.5 Hz); *m/z* [LRMS, ESI] (relative intensity, M) 179 (100, [M+H]+); m/z [HRMS, ESI] Calcd. $(C_{11}H_{11}FO+H)^+$ 179.0867, found 179.0865.

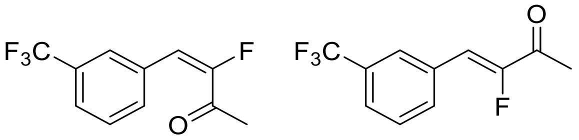


*3-Fluoro-4-(3-trifluoromethylphenyl)-3-buten-2-one* (**1f**): General Procedure A. Reaction performed using 3-(trifluoromethyl)benzaldehyde (79 µL, 0.71 mmol) and diethyl (1-fluoro-2-oxopropyl)phosphonate, subsequent purification *via* flash column chromatography (10% $Et_2O$/pentane) gave *E*-**1f** as a pale yellow oil (58 mg, 43%) and *Z*-**1f** as a colourless oil (42 mg, 31%).

(*E*)-**1f**: IR ; $\upsilon_{max}$ (oil) 1706 (m) cm$^{-1}$; $^{1}$H NMR (700 MHz, $CDCl_3$) δ 7.86 (1H, s), 7.78 (1H, d, *J* = 7.8 Hz), 7.61 (1H, d, *J* = 7.8 Hz), 7.49 (1H, app t, *J* = 7.8 Hz), 6.69 (1H, d, *J* = 23.7 Hz), 2.37 (3H, d, *J* = 5.1 Hz); $^{13}$C NMR (176 MHz, $CDCl_3$) δ 192.9 (C, d, *J* = 39.5 Hz), 153.7 (C, d, *J* = 261.7 Hz), 133.3 (CH, d, *J* = 1.2 Hz), 131.7 (C, d, *J* = 10.8 Hz), 130.8 (CH, q, *J* = 32.5 Hz), 128.8 (CH, s), 127.0 (CH, app quintet, *J* = 3.7 Hz), 126.0-125.9 (CH, m), 124.0 (C, q, *J* = 272.3 Hz), 118.4 (CH, d, *J* = 28.7 Hz), 28.2 ($CH_3$, d, *J* = 2.1 Hz); $^{19}$F NMR (659 MHz, $CDCl_3$) δ −62.8 (3F, s), −111.0 (1F, dq, *J* = 23.7, 5.1 Hz); *m/z* [LRMS, ESI] (relative intensity, M) 233 (100, [M+H]+), 191 (22, [M+H-COMe]+); m/z [HRMS, ESI] Calcd. $(C_{11}H_8F_4O+H)^+$ 233.0590, found 233.0579.

(*Z*)-**1f**: $^{1}$H NMR (700 MHz, $CDCl_3$) δ 7.93 (1H, s), 7.84 (1H, d, *J* = 7.8 Hz), 7.65 (1H, d, *J* = 7.8 Hz), 7.55 (1H, t, *J* = 7.8 Hz), 6.85 (1H, d, *J* = 35.5 Hz), 2.45 (3H, d, *J* = 3.6 Hz); $^{13}$C NMR (176 MHz, $CDCl_3$) δ 192.2 (C, d, *J* = 34.2 Hz), 154.7 (C, d, *J* = 275.6 Hz), 133.7 (CH, d, *J* = 8.6 Hz), 131.9 (C, d, *J* = 3.8 Hz), 131.5 (CH, q, *J* = 32.6 Hz), 129.5 (CH, s), 127.2 (CH, dq, *J* = 7.8, 3.9 Hz), 126.4 (CH, app quintet, *J* = 3.5 Hz), 123.8 (C, q, *J* = 272.5 Hz), 113.7 (CH, d, *J* = 5.1 Hz), 25.9 ($CH_3$, s); $^{19}$F NMR (659 MHz, $CDCl_3$) δ −63.0 (3F, s), −121.4 (1F, dq, *J* = 35.5, 3.6 Hz); m/z [HRMS, ESI] Calcd. $(C_{11}H_8F_4O+H)^+$ 233.0590, found 233.0576. Data in agreement with that reported in the literature.[69]

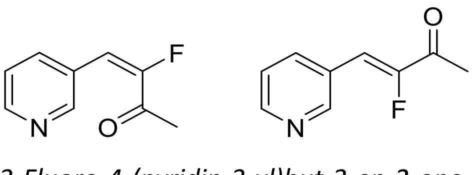


*3-Fluoro-4-(pyridin-3-yl)but-3-en-2-one* (**2a**): General Procedure A. Reaction performed using 3-pyridinecarboxaldehyde (74 µL, 0.78 mmol) and diethyl (1-fluoro-2-oxopropyl)phosphonate, subsequent purification *via* flash column chromatography (50-100% EtOAc/cyclohexane) gave *E*-**2a** as a colourless oil (44 mg, 34%) and *Z*-**2a** as an off white solid (36 mg, 28%).

(*E*)-**2a**: $\upsilon_{max}$ (oil) 1709 (m), 1624 (m) cm$^{-1}$; $^{1}$H NMR (700 MHz, $CDCl_3$) δ 8.65 (1H, s), 8.56-8.54 (1H, m), 8.16-8.13 (1H, m), 7.32-7.30 (1H, m), 6.62 (1H, d, *J* = 23.5 Hz), 2.39-2.37 (3H, m); $^{13}$C NMR (176 MHz, $CDCl_3$) δ 193.0 (C, d, *J*

= 39.8 Hz), 154.0 (C, d, *J* = 262.6 Hz), 150.9 (CH, s), 149.8 (CH, s), 137.2 (CH, d, *J* = 2.1 Hz), 127.3 (C, d, *J* = 10.0 Hz), 123.2 (CH, s), 116.2 (CH, d, *J* = 29.2 Hz), 28.1 ($CH_3$, d, *J* = 2.1 Hz); $^{19}$F NMR (659 MHz, $CDCl_3$) δ −109.6 to −109.5 (m); *m/z* [LRMS, ESI] (relative intensity, M) 166 (100, [M+H]+); m/z [HRMS, ESI] Calcd. $(C_9H_8FNO+H)^+$ 166.0663, found 166.0661.

(*Z*)-**2a**: m.p. 66-67 °C ; $υ_{max}$ (solid) 1707 (m), 1687 (s), 1638 (m) cm$^{-1}$; $^1$H NMR (700 MHz, $CDCl_3$) δ 8.83 (1H, s), 8.61-8.60 (1H, m), 8.08-8.06 (1H, m), 7.40-7.37 (1H, m), 6.82 (1H, d, *J* = 36.0 Hz), 2.45-2.44 (3H, m); $^{13}$C NMR (176 MHz, $CDCl_3$) δ 192.0 (C, d, *J* = 33.9 Hz), 155.3 (C, d, *J* = 275.7 Hz), 151.3 (CH, d, *J* = 7.1 Hz), 150.3 (CH, d, *J* = 2.9 Hz), 137.4 (CH, d, *J* = 10.2 Hz), 127.6 (C, d, *J* = 4.2 Hz), 124.0 (CH, s), 111.8 (CH, d, *J* = 6.1 Hz), 25.9 ($CH_3$, s); $^{19}$F NMR (659 MHz, $CDCl_3$) δ −120.3 to −120.5 (m); *m/z* [LRMS, ESI] (relative intensity, M) 166 (100, [M+H]+); m/z [HRMS, ESI] Calcd. $(C_9H_8FNO+H)^+$ 166.0663, found 166.0660.

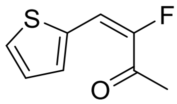


*3-Fluoro-4-(thiophen-2-yl)but-3-en-2-one* (**2b**): General Procedure A. Reaction performed using thiophene-2-carboxaldehyde (114 µL, 1.96 mmol) and diethyl (1-fluoro-2-oxopropyl)phosphonate, subsequent purification *via* flash column chromatography (0-20% EtOAc/hexane) gave *E*-**2b** as a pale yellow solid (116 mg, 35%). m.p. 67-70 °C ; $υ_{max}$ (oil) 2923 (m), 1698 (s), 1620 (s) cm$^{-1}$; $^1$H NMR (400 MHz, $CDCl_3$) δ 7.55 (1H, d, *J* = 5.1 Hz), 7.39 (1H, d, *J* = 3.6 Hz), 7.18 (1H, d, *J* = 35.0 Hz), 7.13-7.10 (1H, m), 2.41 (3H, d, *J* = 3.3 Hz); $^{13}$C NMR (100 MHz, $CDCl_3$) δ 191.5 (C, d, *J* = 31.5 Hz), 152.6 (C, d, *J* = 269.7 Hz), 133.7 (C, d, *J* = 5.4 Hz), 132.3 (CH, d, *J* = 4.5 Hz), 131.0 (CH, d, *J* = 10.2 Hz), 127.8 (CH, s), 110.4 (CH, d, *J* = 9.9 Hz), 25.7 ($CH_3$, s); $^{19}$F NMR (377 MHz, $CDCl_3$) δ −122.1 (d, *J* = 35.0 Hz); *m/z* [LRMS, ESI] (relative intensity, M) 171 (100, [M+H]$^+$; m/z [HRMS, ESI] Calcd. $(C_8H_7FO^{32}S+H)^+$ 171.0274, found 171.0272.

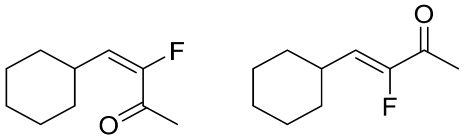


*3-Fluoro-4-cyclohexylbut-3-en-2-one* (**3a**): General Procedure A. Reaction performed using cyclohexanecarboxaldehyde (95 µL, 0.79 mmol) and diethyl (1-fluoro-2-oxopropyl)phosphonate, subsequent purification *via* flash column chromatography (5% $Et_2O$/pentane) gave *E*-**3a** as a colourless oil (45 mg, 33%) and *Z*-**3a** as a colourless oil (46 mg, 34%).

(*E*)-**3a**: $υ_{max}$ (oil) 2930 (s), 2855 (m), 1705 (s) cm$^{-1}$; $^1$H NMR (700 MHz, $CDCl_3$) δ 5.54 (1H, dd, *J* = 22.8, 10.3 Hz), 3.10-3.04 (1H, m), 2.37 (3H, d, *J* = 5.0 Hz), 1.74-1.64 (5H, m), 1.38-1.30 (2H, m), 1.22-1.15 (1H, m), 1.11-1.04 (2H, m); $^{13}$C NMR (176 MHz, $CDCl_3$) δ 194.6 (C, d, *J* = 40.3 Hz), 152.6 (C, d, *J* = 254.7 Hz), 127.7 (CH, d, *J* = 15.3 Hz), 34.2 (CH, d, *J* = 5.0 Hz), 32.9 ($CH_3$, d, *J* = 2.3 Hz), 28.0 ($CH_2$, d, *J* = 2.8 Hz), 25.9 ($CH_2$, s), 25.5 ($CH_2$, s); $^{19}$F NMR (659 MHz, $CDCl_3$) δ −120.9 (dd, *J* = 22.8, 3.0 Hz); m/z [HRMS, ESI] Calcd. $(C_{10}H_{15}FO+H)^+$ 171.1180, found 171.1181.

(*Z*)-**3a**: $^1$H NMR (700 MHz, $CDCl_3$) $\delta$ 5.90 (1H, dd, *J* = 34.8, 9.6 Hz), 2.60-2.53 (1H, m), 2.30 (3H, d, *J* = 2.8 Hz), 1.77-1.65 (5H, m), 1.37-1.15 (5H, m); $^{13}$C NMR (176 MHz, $CDCl_3$) $\delta$ 192.1 (C, d, *J* = 31.8 Hz), 153.9 (C, d, *J* = 259.2 Hz), 125.0 (CH, d, *J* = 12.4 Hz), 34.1 (CH, s), 32.1 ($CH_3$, d, *J* = 1.3 Hz), 25.9 ($CH_2$, s), 25.7 ($CH_2$, s), 25.5 ($CH_2$, s); $^{19}$F NMR (659 MHz, $CDCl_3$) $\delta$ −129.1 (d, *J* = 34.8 Hz); m/z [LRMS, EI] Calcd. $(C_{10}H_{15}FO)^+$ 170, found 170. Data in agreement with that reported in the literature.[76]

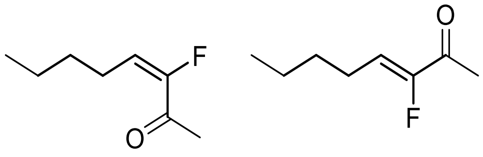

*3-Fluorooct-3-en-2-one* (**3b**): General Procedure A. Reaction performed using valeraldehyde (125 µL, 0.983 mmol) and diethyl (1-fluoro-2-oxopropyl)phosphonate, subsequent purification *via* flash column chromatography (5% $Et_2O$/pentane) gave *E*-**3b** as a pale yellow oil (87 mg containing 39 mg $Et_2O$ by NMR (1:1.5 ratio), 28%) and *Z*-**3b** as a pale yellow oil (89 mg containing 30 mg $Et_2O$ by NMR (1:1 ratio), 35%).

(*E*)-**3b**: $\upsilon_{max}$ (oil) 2960 (m), 2931 (m), 2873 (w), 1734 (s) $cm^{-1}$; $^1$H NMR (700 MHz, $CDCl_3$) $\delta$ 5.72 (1H, dt, *J* = 22.4, 8.1 Hz), 3.49 (q, residual $Et_2O$), 2.54-2.50 (2H, m), 2.30 (3H, d, *J* = 4.9 Hz), 1.44-1.33 (4H, m), 1.21 (t, residual $Et_2O$), 0.91 (3H, t, *J* = 7.2 Hz); $^{13}$C NMR (176 MHz, $CDCl_3$) $\delta$ 194.7 (C, d, *J* = 40.1 Hz), 153.4 (C, d, *J* = 253.9 Hz), 122.0 (CH, d, *J* = 17.8 Hz), 66.0 (residual $Et_2O$), 31.5 ($CH_2$, d, *J* = 1.9 Hz), 27.9 ($CH_3$, d, *J* = 2.8 Hz), 25.1 ($CH_2$, d, *J* = 5.5 Hz), 22.4 ($CH_2$, s), 15.4 (residual $Et_2O$), 13.9 ($CH_3$, s); $^{19}$F NMR (659 MHz, $CDCl_3$) $\delta$ −119.2 to −119.1 (m); *m/z* [LRMS, ESI] (relative intensity, M) 145 (26, $[M+H]^+$); [HRMS, ESI] Calcd. $(C_8H_{13}FO+H)^+$ 145.1023, found 145.1018.

(*Z*)-**3b**: $\delta$ $\upsilon_{max}$ (oil) 2959 (m), 2932 (m), 2873 (w), 1720 (s) $cm^{-1}$; $^1$H NMR (700 MHz, $CDCl_3$) $\delta$ 6.04 (1H, dt, *J* = 34.2, 7.9 Hz), 3.49 (q, residual $Et_2O$), 2.30 (3H, d, *J* = 2.9 Hz), 2.26 (2H, dq, *J* = 7.4, 2.2 Hz), 1.47-1.43 (2H, m), 1.39-1.35 (2H, m), 1.21 (t, residual $Et_2O$), 0.93 (3H, t, *J* = 7.4 Hz); $^{13}$C NMR (176 MHz, $CDCl_3$) $\delta$ 191.7 (C, d, *J* = 31.8 Hz), 155.3 (C, d, *J* = 259.3 Hz), 120.1 (CH, d, *J* = 13.1 Hz), 66.0 (residual $Et_2O$), 30.6 ($CH_3$, d, *J* = 1.8 Hz), 25.7 ($CH_2$, s), 24.1 ($CH_2$, d, *J* = 3.2 Hz), 22.4 ($CH_2$, s), 15.4 (residual $Et_2O$), 13.9 ($CH_3$, s); $^{19}$F NMR (659 MHz, $CDCl_3$) $\delta$ −128.7 (dq, *J* = 34.2, 2.9 Hz); *m/z* [LRMS, ESI] (relative intensity, M) 145 (100, $[M+H]^+$); [HRMS, ESI] Calcd. $(C_8H_{13}FO+H)^+$ 145.1023, found 145.1019.

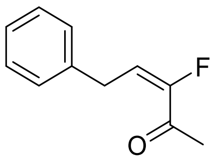

*(E)-3-Fluoro-5-phenylpent-3-en-2-one* (**3c**): General Procedure A. Reaction performed using phenylacetaldehyde (75 µL, 0.640 mmol) and diethyl (1-fluoro-2-oxopropyl)phosphonate, subsequent purification *via* flash column chromatography (0-10% EtOAc/hexane) gave *E*-**3c** as a colourless oil (49 mg, 43%). $^1$H NMR (400 MHz, $CDCl_3$) $\delta$ 7.31-7.19 (5H, m), 5.87 (1H, dt, *J* = 21.4, 8.1 Hz), 3.88 (2H, dd, *J* = 8.1, 1.1 Hz), 2.34 (3H, d, *J* = 4.8 Hz); $^{13}$C NMR

(100 MHz, $CDCl_3$) δ 194.8 (C, d, *J* = 39.8 Hz), 153.2 (C, d, *J* = 256.7 Hz), 139.0 (C, d, *J* = 2.0 Hz), 128.7 (CH, s), 128.4 (CH, s), 126.6 (CH, s), 119.9 (CH, d, *J* = 19.6 Hz), 31.3 ($CH_2$, d, *J* = 6.0 Hz), 27.8 ($CH_3$, d, *J* = 2.8 Hz); $^{19}F$ NMR (377 MHz, $CDCl_3$) δ −118.5 (dq, *J* = 21.4, 4.8 Hz); m/z [LRMS, EI] Calcd. $(C_{11}H_{11}FO)^+$ 178, found 178. Data in agreement with that reported in the literature.[80]

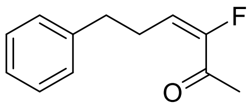


*(E)-3-Fluoro-6-phenylhex-3-en-2-one* (**3d**) – General Procedure A. Reaction performed using 3-phenylpropionaldehyde (85 µL, 0.640 mmol) and diethyl (1-fluoro-2-oxopropyl)phosphonate, subsequent purification *via* flash column chromatography (0-10% EtOAc/hexane) gave *E*-**3d** as a pale yellow oil (73 mg, 59%). $^{1}H$ NMR (400 MHz, $CDCl_3$) δ 7.37-7.25 (5H, m), 5.78 (1H, dt, *J* = 21.7, 7.9 Hz), 2.94-2.88 (2H, m), 2.81-2.78 (2H, m), 2.33 (3H, d, *J* = 4.7 Hz); $^{13}C$ NMR (100 MHz, $CDCl_3$) δ 194.6 (C, d, *J* = 39.9 Hz), 153.0 (C, d, *J* = 254.3 Hz), 140.8 (C, s), 128.5 (CH, s), 128.4 (CH, s), 126.2 (CH, s), 120.4 (CH, d, *J* = 18.6 Hz), 35.2 ($CH_2$, d, *J* = 2.0 Hz), 27.8 ($CH_3$, d, *J* = 2.7 Hz), 26.9 ($CH_2$, d, *J* = 5.5 Hz); $^{19}F$ NMR (377 MHz, $CDCl_3$) δ −118.4 (dq, *J* = 21.7, 4.7 Hz); m/z [LRMS, EI] Calcd. $(C_{12}H_{13}FO)^+$ 192, found 192. Data in agreement with that reported in the literature.[78]

2-Fluorocyclohex-2-en-1-one (**4a**):

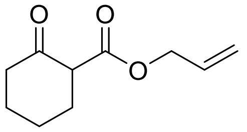


Step 1) *Allyl 2-oxocyclohexane-1-carboxylate*

Following the procedure of Driver *et al.*[81] A solution of ethyl-2-oxocylcohexane carboxylate (1.50 mL, 9.00 mmol) in anhydrous PhMe (20 mL) was treated with allyl alcohol (12.0 mL, 1.80 mol) and DMAP (330 mg, 2.70 mmol). The resultant solution was heated to reflux for 72 h. After this time the solution was cooled to rt and conc. *in vacuo*. Purification *via* flash column chromatography (0%-10% EtOAc/cyclohexane) gave allyl 2-oxocyclohexane-1-carboxylate as a colourless oil (1.45 g, containing 16 mol% ethyl ester, mix of keto-enol forms). The inseparable mixture was taken through to the next step. $^{1}H$ NMR (700 MHz, $CDCl_3$) keto form δ 6.00-5.90 (1H, m), 5.34 (1H, dq, *J* = 17.2, 1.4 Hz), 5.24 (1H, dq, *J* = 10.5, 1.4 Hz), 4.67-4.64 (2H, m), 2.40-2.35 (1H, m), 2.28-2.25 (3H, m), 2.22-1.59 (5H, m); enol form δ 12.15 (1H, s), 6.00-5.90 (1H, m), 5.33 (1H, dq, *J* = 17.2, 1.1 Hz), 5.25 (1H, dq, *J* = 10.4, 1.1 Hz), 4.66 (2H, dt, *J* = 5.5, 1.0 Hz), 2.29-2.24 (4H, m), 1.71-1.62 (2H, m), 1.61-1.58 (2H, m); $^{13}C$ NMR (176 MHz, $CDCl_3$) keto form δ 206.2 (C), 169.8 (C), 132.0 (CH), 118.9 ($CH_2$), 65.8 ($CH_2$), 57.4 (CH), 41.7 ($CH_2$), 30.1 ($CH_2$), 27.2 ($CH_2$), 23.4 ($CH_2$); enol form δ 172.6 (C), 172.4 (C), 132.4 (CH), 117.9 ($CH_2$), 97.7 (C), 64.8 ($CH_2$), 29.3 ($CH_2$), 27.0 ($CH_2$), 22.5 ($CH_2$), 22.0 ($CH_2$); [HRMS, ESI] Calcd. $(C_{10}H_{14}O_3+H)^+$ 183.1016, found 183.108. Data in agreement with that reported in the literature (Enol form,[81] Keto form[82]).

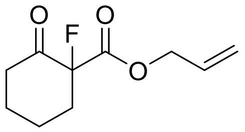

Step 2) *Allyl 1-fluoro-2-oxocyclohexanonecarboxylate*

Following the procedure of Stoltz *et al.*:[83] A solution of allyl 2-oxocyclohexane-1-carboxylate (1.00 g, 5.94 mmol) in anhydrous MeCN (52 mL) was treated with $TiCl_4$ (0.53 mL, 0.53 mmol, 1 M in $CH_2Cl_2$) followed by SelectFluor (2.50 g, 7.13 mmol, added portion-wise over 5 min). The resultant dark orange solution was allowed to stir at rt for 20 h. After this time $H_2O$ (40 mL) was added and the solution extracted with $Et_2O$ (2 x 40 mL). The organics were dried ($MgSO_4$), filtered and conc. *in vacuo.* Purification *via* flash column chromatography (5%-40% EtOAc/cyclohexane) gave allyl 1-fluoro-2-oxocyclohexanonecarboxylate as a colourless oil (814 mg, 68% yield, containing 16 mol% ethyl ester). The inseparable mixture was taken through to the next step. $^1$H NMR (700 MHz, $CDCl_3$) δ 5.92 (1H, ddt, *J* = 16.8, 10.6, 5.4 Hz), 5.37 (1H, d, *J* = 16.8 Hz), 5.29 (1H, d, *J* = 10.6 Hz), 4.76-4.69 (1H, m), 2.76-2.71 (1H, m), 2.63-2.58 (1H, m), 2.51-2.43 (1H, m), 2.20-2.14 (1H, m), 1.96-1.82 (4H, m); $^{13}$C NMR (176 MHz, $CDCl_3$) δ 201.9 (C, d, *J* = 20.1 Hz), 166.8 (C, d, *J* = 24.8 Hz), 131.1 ($CH_2$, s), 119.6 (CH, s), 96.5 (CH, d, *J* = 196.8 Hz), 66.8 ($CH_2$, s), 39.7 ($CH_2$, s), 36.2 ($CH_2$, d, *J* = 21.7 Hz), 26.7 ($CH_2$, s), 21.0 ($CH_2$, d, *J* = 5.6 Hz); $^{19}$F NMR (659 MHz, $CDCl_3$) δ −160.9 to −160.8 (m); [HRMS, ESI] Calcd. $(C_{10}H_{13}FO_3+H)^+$ 201.0922, found 201.0918. Data in agreement with that reported in the literature.[83]

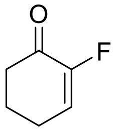

Step 3) *2-Fluorocyclohex-2-en-1-one* (**4a**): Following the procedure of Ishii *et al.*[58] A solution of triphenylphosphine (20 mg, 0.076 mmol) and $Pd_2(dba)_3$ (70 mg, 0.076 mmol) in anhydrous MeCN (6 mL) was heated to 80 °C, a solution of allyl 1-fluoro-2-oxocyclohexanonecarboxylate in MeCN (2 mL) was then added dropwise and the resultant solution stirred at 80 °C for 2 h. After this time the solution was cooled to rt and conc. *in vacuo*. The residue was taken up in $Et_2O$ (15 mL) and washed with brine (15 mL). The organics were dried ($MgSO_4$), filtered and conc. *in vacuo*. Purification *via* flash column chromatography (5% $Et_2O$/pentane) gave **4a** as a colourless oil (82 mg, 48%). $^1$H NMR (700 MHz, $CDCl_3$) δ 6.47 (1H, dt, *J* = 13.9, 4.5 Hz), 2.56-2.54 (2H, m), 2.47 (2H, app quintet, *J* = 5.2 Hz), 2.06-2.01 (2H, m); $^{13}$C NMR (176 MHz, $CDCl_3$) δ 191.6 (C, d, *J* = 19.3 Hz), 154.1 (C, d, *J* = 261.3 Hz), 125.5 (CH, d, *J* = 12.4 Hz), 38.5 ($CH_2$, d, *J* = 3.6 Hz), 24.0 ($CH_2$, d, *J* = 5.1 Hz), 22.7 ($CH_2$, s); $^{19}$F NMR (659 MHz, $CDCl_3$) δ −130.0 to −129.9 (m); m/z [LRMS, EI] Calcd. $(C_{10}H_7FO)^+$ 114, found 114. Data in agreement with that reported in the literature ($^{13}$C not previously reported).[58]

*2-Fluorocyclohept-2-en-1-one* (**4b**):

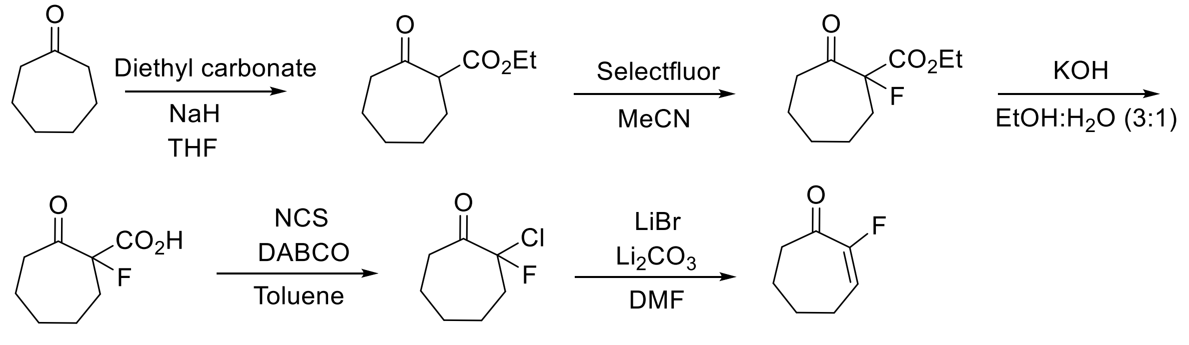


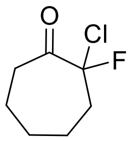


*2-Chloro-2-fluorocycloheptan-1-one*: A solution of cycloheptanone (2.40 mL, 20.5 mmol) in THF (20.5 mL) was added dropwise to a stirred suspension of diethyl carbonate (12.0 mL, 98.7 mmol) and NaH (60 % dispersion in mineral oil, 2.1 equiv.) in THF (41 mL). The mixture was heated to reflux for 12 h, cooled in an ice bath and then acidified with aqueous 10% HCl. The residue was extracted with $Et_2O$ (3 x 30 mL). The combined organic layers were dried over $MgSO_4$, filtered and the solvent was removed *in vacuo*. The crude product was subjected to the next reaction without further purification.

Following the procedure of Armstrong *et al.*[84] The crude product was resuspended in acetonitrile (255 mL) and Selectfluor (14.5 g, 41.0 mmol) was added. The mixture was heated to 80 °C for 18 h and then cooled to room temperature. $H_2O$ (255 mL) was added and the residue was extracted with $Et_2O$ (3 x 30 mL). The combined organic layers were dried over $MgSO_4$, filtered and the solvent was removed *in vacuo*. $CH_2Cl_2$ was added (30 mL) to the crude mixture, the mixture filtered through celite and the remaining solvent removed under reduced pressure. The crude product was subjected to the next reaction without further purification.

Following the procedure of Shibatomi *et al.*[85] Potassium hydroxide (3.45 g, 61.6 mmol) was added to a solution of crude α-fluoro-β-oxoester in EtOH and $H_2O$ (3:1, 0.21 M), and the mixture was stirred at –10 °C for 1 h. After the reaction completed, the reaction mixture was diluted with $H_2O$ and washed with $CH_2Cl_2$. The resulting aqueous layer was acidified with 1.2 N HCl, and then extracted with $CH_2Cl_2$. The organic layer was dried over $MgSO_4$ and concentrated to give *α*-fluoro-*β*-oxocarboxylic acid. The crude product was subjected to the next reaction without further purification. Product also contained significant quantities of the corresponding decarboxylated compound.

Following the procedure of Shibatomi *et al.*[85] DABCO (90 mg, 10 mol%) and *N*-chlorosuccinimide (3.22 g, 24.1 mmol) were added to a stirred solution of α-fluoro-β-oxocarboxylic acid (1.40 g, 8.04 mmol) in toluene (0.2 M), and the mixture was stirred at room temperature overnight. After the reaction completed, the reaction mixture

was directly subjected to silica-gel flash column chromatography (1:9 $Et_2O$:Hexanes) to give the corresponding α-chloro-α-fluoroketone as a colourless oil (601 mg, 45%). $^1$H NMR (300 MHz, $CDCl_3$) δ 2.85-2.73 (1H, m), 2.67-2.52 (2H, m), 2.39-2.19 (1H, m), 2.09-1.46 (6H, m); $^{13}$C NMR (101 MHz, $CDCl_3$) δ 199.8 (C, d, *J* = 21.8 Hz), 108.6 (C, d, *J* = 255.9 Hz), 40.1 ($CH_2$, d, *J* = 23.2 Hz), 38.4 ($CH_2$, s), 28.9 ($CH_2$, s), 25.4 ($CH_2$, s), 24.3 ($CH_2$, d, *J* = 2.2 Hz); $^{19}$F NMR (282 MHz, $CDCl_3$) δ −113.6 (dd, J = 29.0, 13.3 Hz). Data in agreement with that reported in the literature.[85]

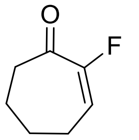

*2-Fluorocyclohept-2-en-1-one* (**4b**) – Following the procedure of Shibatomi *et al.*:[85] LiBr (127 mg, 1.46 mmol) and $Li_2CO_3$ (108 mg, 1.46 mmol) were added to a stirred solution of *α*-chloro-*α*-fluoroketone (100 mg, 0.61 mmol) in DMF (1 mL), and the mixture was stirred at 130 °C for 5 h. After the reaction completed, the reaction mixture was diluted with $H_2O$, and extracted with $Et_2O$. The organic layer was washed with brine, dried over $MgSO_4$, and concentrated. The resulting crude product was subjected to silica-gel flash (1:9 $Et_2O$:Hexanes) column chromatography to give **4b** as a colourless oil (36 mg, 46%). $^1$H NMR (300 MHz, $CDCl_3$) δ 6.37 (dt, *J* = 21.7, 6.3 Hz, 1H), 2.67–2.63 (m, 2H), 2.45–2.39 (m, 2H), 1.88–1.80 (m, 4H); $^{13}$C NMR (101 MHz, $CDCl_3$) δ 194.5 (d, *J* = 22.6 Hz), 155.7 (d, *J* = 248.4 Hz), 123.6 (d, *J* = 21.3 Hz), 41.7 (d, *J* = 3.7 Hz), 25.6, 24.8 (d, *J* = 7.8 Hz), 21.0; $^{19}$F NMR (282 MHz, $CDCl_3$) δ −118.8 (dd, *J* = 21.7, 2.3 Hz); *m/z* [LRMS, EI] Calcd. $(C_7H_9FO)^+$ 128, found 128. Data in agreement with that reported in the literature.[85]

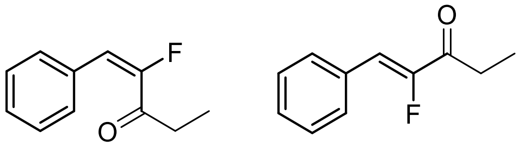

*2-Fluoro-1-phenylpent-1-en-3-one* (**5a**): General Procedure A. Reaction performed using benzaldehyde (151 µL, 1.48 mmol) and diethyl (1-fluoro-2-oxobutyl)phosphonate, subsequent purification *via* flash column chromatography (5 % EtOAc/cyclohexane) gave *E*-**5a** as a pale yellow oil (121 mg, 39%) and *Z*-**5a** as a white solid (45 mg, 17%).

(*E*)-**5a**: $^1$H NMR (700 MHz, $CDCl_3$) δ 7.61 (2H, d, *J* = 7.0 Hz), 7.38-7.35 (3H, m), 6.70 (1H, d, *J* = 25.2 Hz), 2.69 (2H, qd, *J* = 7.2, 3.2 Hz), 1.11 (3H, t, *J* = 7.2 Hz); $^{13}$C NMR (176 MHz, $CDCl_3$) δ 196.0 (C, d, *J* = 37.3 Hz), 153.1 (C, d, *J* = 257.9 Hz), 131.0 (C, d, *J* = 10.6 Hz), 130.2 (CH, s), 129.3 (CH, s), 128.3 (CH, s), 119.8 (CH, d, *J* = 27.3 Hz), 33.7 ($CH_2$, s), 7.3 ($CH_3$, s); $^{19}$F NMR (659 MHz, $CDCl_3$) δ −115.5 (d, *J* = 25.2 Hz); m/z [HRMS, ESI] Calcd. $(C_{11}H_{11}FO+H)^+$ 179.0867, found 179.0866. Data in agreement with that reported in the literature.[56]

(*Z*)-**5a**: m.p. 50-51 °C (Lit.[86] 56 °C); $^1$H NMR (700 MHz, $CDCl_3$) δ 7.67 (2H, d, *J* = 7.2 Hz), 7.43-7.38 (3H, m), 6.84 (1H, d, *J* = 36.9 Hz), 2.78 (2H, qd, *J* = 7.2, 2.0 Hz), 1.19 (3H, t, *J* = 7.2 Hz); $^{13}$C NMR (176 MHz, $CDCl_3$) δ 195.6 (C, d, *J* = 32.5 Hz), 154.2 (C, d, *J* = 273.2 Hz), 131.3 (C, d, *J* = 3.4 Hz), 130.8 (CH, d, *J* = 8.1 Hz), 129.9 (CH, d, *J* = 2.8 Hz), 129.0

(CH, s), 114.9 (CH, d, $J$ = 5.1 Hz), 31.5 ($CH_2$, s), 7.6 ($CH_3$, d, $J$ = 1.5 Hz); $^{19}$F NMR (659 MHz, $CDCl_3$) δ −125.4 (d, $J$ = 36.9 Hz); m/z [HRMS, ESI] Calcd. $(C_{11}H_1FO+H)^+$ 179.0867, found 179.0865. Data in agreement with that reported in the literature.[76]

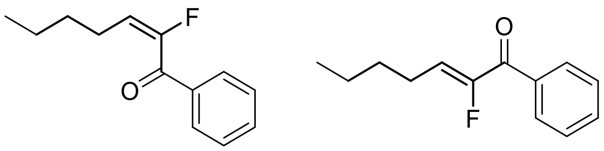

*2-Fluoro-1-phenylhept-2-en-1-one* (**5b**): General Procedure A. Reaction performed using valeraldehyde (62 µL, 0.58 mmol) and diethyl (1-fluoro-2-oxo-2-phenylethyl)phosphonate, subsequent purification *via* flash column chromatography (0-5% $Et_2O$/hexane) gave *Z*-**5b** as a pale yellow oil (31 mg, 26%), *E*-**5b** degraded under purification conditions.

(*E*)-**5b**: Compound degraded upon column chromatography and could not be isolated cleanly, further degradation was seen when stored at -20 °C. $^1$H NMR crude (700 MHz, $CDCl_3$) δ 7.89 (2H, d, $J$ = 7.7 Hz), 7.58 (1H, t, $J$ = 7.7 Hz), 7.47 (2H, app t, $J$ = 7.7 Hz), 5.91 (1H, dt, $J$ = 22.8, 8.3 Hz), 2.40 (2H, dq, $J$ = 8.3, 1.7 Hz), 1.47-1.42 (2H, m), 1.35 (2H, app sextet, $J$ = 7.6 Hz), 0.90 (3H, t, $J$ = 7.3 Hz); $^{19}$F NMR (659 MHz, $CDCl_3$) δ −113.1 (d, $J$ = 22.8 Hz).

(*Z*)-**5b**: $\upsilon_{max}$ (oil) 2958 (m), 2931 (m), 2862 (w), 1765 (s), 1745 (s) cm$^{-1}$; $^1$H NMR (700 MHz, $CDCl_3$) δ 7.81 (2H, d, $J$ = 7.5 Hz), 7.58 (1H, t, $J$ = 7.5 Hz), 7.47 (2H, app t, $J$ = 7.5 Hz), 6.04 (1H, dt, $J$ = 34.2, 7.7 Hz), 2.36 (2H, dq, $J$ = 7.7, 1.9 Hz), 1.49 (2H, app quintet, $J$ = 7.5 Hz), 1.38 (2H, app sextet, $J$ = 7.5 Hz), 0.93 (3H, t, $J$ = 7.3 Hz); $^{13}$C NMR (176 MHz, $CDCl_3$) δ 187.8 (C, d, $J$ = 28.2 Hz), 155.4 (C, d, $J$ = 260.1 Hz), 136.5 (C, s), 132.9 (CH, s), 129.4 (CH, d, $J$ = 3.5 Hz), 128.5 (CH, s), 124.6 (CH, d, $J$ = 13.1 Hz), 30.6 ($CH_2$, d, $J$ = 1.5 Hz), 25.5 ($CH_2$, d, $J$ = 3.3 Hz), 22.5 ($CH_2$, s); 13.9 ($CH_3$, s); $^{19}$F NMR (659 MHz, $CDCl_3$) δ −124.9 (d, $J$ = 34.2 Hz); *m/z* [LRMS, ESI] (relative intensity, M) 207 (100, $[M+H]^+$); m/z [HRMS, ESI] Calcd. $(C_{13}H_{15}FO+H)^+$ 207.1180, found 207.1180.

## α-Fluoroketone (Reference Standards, 7-12)

### General Procedure B:[87]

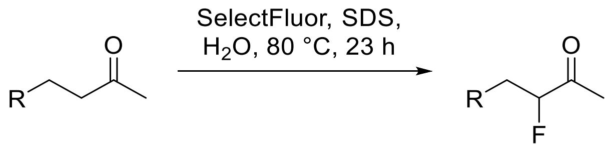


Following the procedure of Stavber *et al.*:[87] A suspension of ketone (1 eq.) in $H_2O$ (5 mL/mmol) was stirred vigorously for 2 min. After this time sodium lauryl sulfate (0.5 eq.) was added and the resultant solution heated to 80 °C. When the solution reached 80 °C a portion of SelectFluor (0.55 eq.) was added and heating continued for 1 h after which time an additional portion of SelectFluor (0.55 eq.) was added. The reaction mixture was heated at 80 °C for 23 h, cooled to rt and extracted into $Et_2O$ (2 x 15 mL/mmol). The combined organics were dried ($Na_2SO_4$), filtered and conc. *in vacuo*. Purification was achieved using flash column chromatography.

### General Procedure C:[88,83,58]

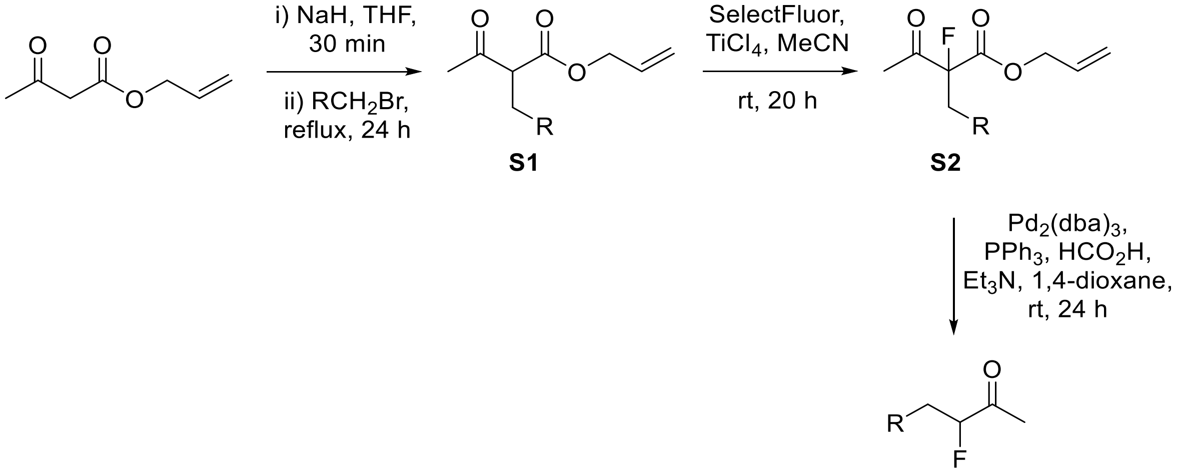


**Step 1 (S1):**[88] Following the analogous procedure of Feng *et al.* for allyl 3-oxo-2-benzylbutanoate: A suspension of NaH (95%, 1.1 eq.) in THF (3.3 mL/mmol) was treated with a solution of allyl acetoacetate (1.1 eq.) in THF (0.45 mL/mmol), with cooling in a rt water bath, and stirred for 30 min. After this time $ArCH_2Br$ (1 eq.) in THF (0.45 mL/mmol) was added dropwise and the resultant solution heated to reflux for 24 h. After cooling to rt $NH_4Cl_{(sat.\ aq.)}$ (4 mL/mmol) was added, extracted with $Et_2O$ (3 x 10 mL/mmol), the combined organics were dried ($Na_2SO_4$), filtered and conc. *in vacuo.* Purification was achieved using flash column chromatography.

**Step 2 (S2):**[83] Following a modified procedure of Stoltz *et al.* for allyl 2-fluoro-2-cyclohexanonecarboxylate: A solution of **S1** (1 eq.) in anhydrous MeCN (8.7 mL/mmol) was treated with $TiCl_4$ (0.09 eq., 1 M in $CH_2Cl_2$) followed by SelectFluor (1.2 eq., added portion-wise over 5 min). The resultant dark orange solution was allowed to stir at rt for 20 h. After this time $H_2O$ (9 mL/mmol) was added and the solution extracted with $Et_2O$ (2 x 9 mL/mmol). The organics were dried ($MgSO_4$), filtered and conc. *in vacuo.* Purification was achieved using flash column chromatography.

**Step 3 (2):**[58] Following the analogous procedure of Ishii *et al.* for allyl 1-fluoro-2-cyclododecanonecarboxylate: A solution of formic acid (4.4 eq.) and $Et_3N$ (2 eq.) in dioxane (6.5 mL/mmol) was added to a stirring solution of $Pd_2(dba)_3$ (0.01 eq.) and $PPh_3$ (0.01 eq.) in dioxane (13 mL/mmol). A solution of **S2** (1 eq.) in dioxane (6.5 mL/mmol) was then added and the reaction stirred at rt for 24 h. After this time the reaction was poured onto $NaHCO_{3(sat.\ aq.)}$ (13 mL/mmol), extracted with $Et_2O$ (2 x 30 mL/mmol), the combined organics were dried ($Na_2SO_4$), filtered and conc. *in vacuo.* Purification was achieved using flash column chromatography.

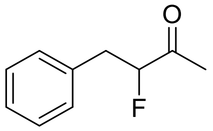


*3-Fluoro-4-phenylbutan-2-one* (**7a**): General Procedure B. Reaction performed using benzyl acetone (180 mg, 1.00 mmol), subsequent purification *via* flash column chromatography ($CH_2Cl_2$) gave **7a** as a colourless oil (42 mg, 25%; Lit.[48] yield 83%). $^1H$ NMR (700 MHz, $CDCl_3$) $\delta$ 7.33 (2H, app t, $J$ = 7.5 Hz), 7.29-7.27 (1H, m), 7.24 (2H, d, $J$ = 7.5 Hz), 4.94 (1H, ddd, $J$ = 49.8, 7.6, 3.7 Hz), 3.20 (1H, ddd, $J$ = 28.9, 14.5, 3.7 Hz), 3.06 (1H, ddd, $J$ = 26.2, 14.5, 7.6 Hz), 2.15 (3H, d, $J$ = 4.9 Hz); $^{13}C$ NMR (176 MHz, $CDCl_3$) $\delta$ 208.1 (C, d, $J$ = 26.2 Hz), 135.4 (C, s), 129.6 (CH, s), 128.7 (CH, s), 127.2 (CH, s), 96.0 (CH, d, $J$ = 187.4 Hz), 38.2 ($CH_2$, d, $J$ = 20.4 Hz), 26.5 ($CH_3$, s); $^{19}F$ NMR (376 MHz, $CDCl_3$, H decoupled) $\delta$ −188.2; m/z [LRMS, EI] Calcd. $(C_{10}H_{11}FO)^+$ 166, found 166. Data in agreement with that reported in the literature.[87]

*3-Fluoro-4-(4-trifluoromethylphenyl)-butan-2-one* (**7b**): General Procedure C.

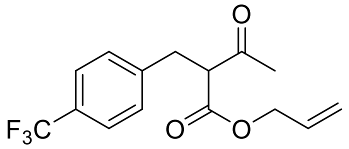


Step 1) *Allyl 3-oxo-2-(4-(trifluoromethyl)benzyl)butanoate* (**S1-7b**) Reaction performed using 4-trifluoromethylbenzyl bromide (950 mg, 3.99 mmol), subsequent purification *via* flash column chromatography (10% EtOAc/cyclohexane) gave **S1-7b** as a colourless oil (678 mg, 57%). $\upsilon_{max}$ (oil) 2942 (w), 1740 (s), 1716 (s) $cm^{-1}$; $^1H$ NMR (700 MHz, $CDCl_3$) $\delta$ 7.54 (2H, d, $J$ = 8.1 Hz), 7.31 (2H, d, $J$ = 8.1 Hz), 5.85 (1H, ddt, $J$ = 17.2, 10.4, 5.9 Hz), 5.26-5.22 (2H, m), 4.60-4.59 (2H, m), 3.82 (1H, t, $J$ = 15.2 Hz), 3.25 (1H, dd, $J$ = 14.2, 7.1 Hz), 3.21 (1H, dd, $J$ = 14.2, 6.7 Hz), 2.23 (3H, s); $^{13}C$ NMR (176 MHz, $CDCl_3$) $\delta$ 201.6 (C, s), 168.5 (C, s), 142.4 (C, s), 131.2 (CH, s), 129.3 (CH, s), 129.2 (C, q, $J$ = 32.5 Hz), 125.7 (CH, q, $J$ = 3.7 Hz), 124.3 (C, d, $J$ = 271.9 Hz), 119.4 ($CH_2$, s), 66.3 ($CH_2$, s), 61.0 (CH, s), 33.7 ($CH_2$, s), 29.8 ($CH_3$, s); *m/z* [LRMS, ESI] (relative intensity, M) 301 (100, [M+H]+), 283 (45, [M+H-F]+), 199 (32, [M+H-$CO_2$allyl-Me]+); [HRMS, ESI] Calcd. $(C_{15}H_{15}F_3O_3+H)^+$ 301.1046, found 301.1044.

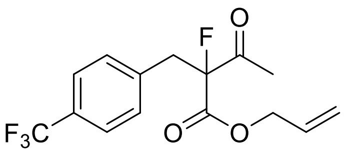

Step 2) *Allyl 2-fluoro-3-oxo-2-(4-(trifluoromethyl)benzyl)butanoate* (**S2-7b**) Reaction performed using **S1-7b** (300 mg, 1.00 mmol), subsequent purification *via* flash column chromatography (10% EtOAc/cyclohexane) gave **S2-7b** as a colourless oil (196 mg, 62%). $\upsilon_{max}$ (oil) 1756 (m), 1735 (m) $cm^{-1}$; $^{1}H$ NMR (700 MHz, $CDCl_3$) $\delta$ 7.56 (2H, d, *J* = 8.1 Hz), 7.36 (2H, d, *J* = 8.1 Hz), 5.83 (1H, ddt, *J* = 16.4, 11.5, 5.8 Hz), 5.32-5.27 (2H, m), 4.68-4.63 (2H, m), 3.49 (1H, dd, *J* = 35.0, 14.8 Hz), 3.46 (1H, dd, *J* = 36.3, 14.8 Hz), 2.19 (3H, d, *J* = 5.1 Hz); $^{13}C$ NMR (176 MHz, $CDCl_3$) $\delta$ 201.8 (C, d, *J* = 29.2 Hz), 165.2 (C, d, *J* = 25.4 Hz), 137.3 (C, s), 130.9 (CH, s), 130.6 (CH, s), 130.0 (C, q, *J* = 32.5 Hz), 125.4 (CH, q, *J* = 3.7 Hz), 124.2 (C, q, *J* = 272.1 Hz), 120.0 ($CH_2$, s), 99.8 (C, d, *J* = 201.3 Hz), 67.2 ($CH_2$, s), 39.4 ($CH_2$, d, *J* = 20.2), 26.2 ($CH_3$, s); $^{19}F$ NMR (282 MHz, $CDCl_3$, H decoupled) $\delta$ −62.6 (3H), −164.7 (1H); *m/z* [LRMS, ESI] (relative intensity, M) 319 (100, [M+H]+), 199 (22, [M+H-$CO_2$allyl-F-Me]+); [HRMS, ESI] Calcd. $(C_{15}H_{14}F_4O_3+H)^+$ 319.0952, found 319.0942.

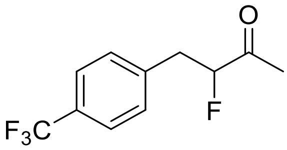

Step 3) *3-Fluoro-4-(4-trifluoromethylphenyl)-butan-2-one* (**7b**) Reaction performed using **S2-7b** (165 mg, 0.518 mmol), subsequent purification *via* flash column chromatography (20% $Et_2O$/pentane) gave **7b** as a colourless oil (50 mg, 41%). $^{1}H$ NMR (700 MHz, $CDCl_3$) $\delta$ 7.58 (2H, d, *J* = 8.1 Hz), 7.36 (2H, d, *J* = 8.1 Hz), 4.95 (1H, ddd, *J* = 49.5, 7.6, 3.7 Hz), 3.25 (1H, ddd, *J* = 28.8, 14.8, 3.7 Hz), 3.06 (1H, ddd, *J* = 25.8, 14.8, 7.7 Hz), 2.18 (3H, d, *J* = 5.0 Hz); $^{13}C$ NMR (176 MHz, $CDCl_3$) $\delta$ 207.6 (C, d, *J* = 26.3 Hz), 139.5 (C, s), 130.0 (CH, s), 129.6 (C, q, *J* = 32.4 Hz), 125.6 (CH, q, *J* = 3.7 Hz), 124.2 (C, q, *J* = 272.0 Hz), 95.5 (CH, d, *J* = 188.1 Hz), 37.8 ($CH_2$, d, *J* = 20.3 Hz), 26.6 ($CH_3$, s); $^{19}F$ NMR (377 MHz, $CDCl_3$) $\delta$ −62.6 (3H, s), −188.4 to −188.7 (1H, m); *m/z* [LRMS, EI] Calcd. $(C_{11}H_{10}F_4O)^+$ 234.07, found 234.09. Data in agreement with that reported in the literature.[34]

*3-Fluoro-4-(4-methoxyphenyl)-butan-2-one* (**7c**): General Procedure C.

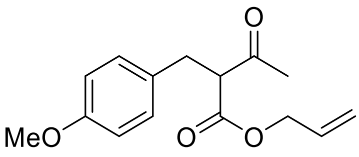

Step 1) *Allyl 3-oxo-2-(4-methoxybenzyl)butanoate* (**S1-7c**) Reaction performed using 4-methoxybenzyl bromide (810 mg, 4.02 mmol), subsequent purification *via* flash column chromatography (10% EtOAc/cyclohexane) gave **S1-7c** as a colourless oil (350 mg, 33%). $\upsilon_{max}$ (oil) 2958 (w), 1739 (s), 1718 (s), 1514 (m) $cm^{-1}$; $^{1}H$ NMR (700 MHz, $CDCl_3$) $\delta$ 7.10 (2H, d, *J* = 8.6 Hz), 6.82 (2H, d, *J* = 8.6 Hz), 5.85 (1H, ddt, *J* = 16.3, 11.4, 5.8 Hz), 5.28-5.23 (2H, m),

4.60-4.59 (2H, m), 3.78 (3H, s), 3.77 (1H, t, *J* = 7.6 Hz), 3.12 (2H, d, *J* = 7.9 Hz), 2.19 (3H, s); $^{13}$C NMR (176 MHz, $CDCl_3$) δ 202.5 (C), 168.8 (C), 158.4 (C), 131.4 (CH), 129.9 (C), 129.8 (CH), 118.9 ($CH_2$), 114.0 (CH), 66.0 ($CH_2$), 61.5 (CH), 55.2 ($CH_3$), 33.2 ($CH_2$), 29.7 ($CH_3$); *m/z* [LRMS, ESI] (relative intensity, M) 261 (100, [M]+), 221 (5, [M-allyl]+); [HRMS, ESI] Calcd. $(C_{15}H_{18}O_4\text{-}H)^-$ 261.1132, found 261.1131.

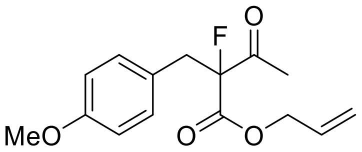


Step 2) *Allyl 2-fluoro-3-oxo-2-(4-methoxybenzyl)butanoate* (**S2-7c**) Reaction performed using **S1-7c** (300 mg, 1.00 mmol), subsequent purification *via* flash column chromatography (10% EtOAc/cyclohexane) gave **S2-7c** as a pale yellow oil (195 mg, 56%). $υ_{max}$ (oil) 2935 (w), 2838 (w), 1754 (s), 1732 (s), 1649 (m), 1513 (s) cm$^{-1}$; $^{1}$H NMR (400 MHz, $CDCl_3$) δ 7.14 (2H, d, *J* = 8.4 Hz), 6.83 (2H, d, *J* = 8.4 Hz), 5.86 (1H, ddt, *J* = 16.5, 10.9, 5.6 Hz), 5.34-5.26 (2H, m), 4.71-4.63 (2H, m), 3.79 (3H, s), 3.42-3.22 (2H, m), 2.13 (3H, d, *J* = 5.2 Hz); $^{13}$C NMR (176 MHz, $CDCl_3$) δ 202.6 (C, d, *J* = 29.5 Hz), 165.5 (C, d, *J* = 25.6 Hz), 159.0 (C, s), 131.5 (CH, s), 130.8 (CH, s), 124.9 (C, s), 119.6 ($CH_2$, s), 113.9 (CH, s), 100.2 (C, d, *J* = 199.9 Hz), 66.9 ($CH_2$, s), 55.3 ($CH_3$, s), 39.1 ($CH_2$, d, *J* = 20.3), 26.5 ($CH_3$, s); $^{19}$F NMR (282 MHz, $CDCl_3$) δ −164.8 (app tq, *J* = 19.3, 5.0 Hz); *m/z* [LRMS, ESI] (relative intensity, M) 281 (50, [M+H]+), 263 (15, [M-F+H]+), 161 (100, [M-F-Me-allyl]); [HRMS, ESI] Calcd. $(C_{15}H_{17}FO_4+H)^+$ 281.1184, found 281.1180.

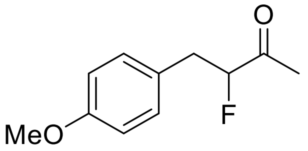


Step 3) *3-Fluoro-4-(4-methoxyphenyl)-butan-2-one* (**7c**) Reaction performed using **S2-7c** (190 mg, 0.678 mmol), subsequent purification *via* flash column chromatography (20% $Et_2O$/pentane) gave **7c** as a colourless oil (98 mg, 74%). $υ_{max}$ (oil) 2934 (w), 2837 (w), 1722 (s), 1612 (m) cm$^{-1}$; $^{1}$H NMR (700 MHz, $CDCl_3$) δ 7.16-7.14 (2H, m), 6.87-6.84 (2H, m), 4.89 (1H, ddd, *J* = 50.0, 7.3, 3.9 Hz), 3.80 (3H, s), 3.13 (1H, ddd, *J* = 27.9, 14.0, 3.9 Hz), 3.00 (1H, ddd, *J* = 22.2, 14.0, 7.3 Hz), 2.13 (3H, d, *J* = 4.9 Hz); $^{13}$C NMR (176 MHz, $CDCl_3$) δ 208.4 (C, d, *J* = 26.3 Hz), 158.8 (C, s), 130.7 (CH, s), 127.3 (CH, s), 114.1 (C, s), 96.2 (CH, d, *J* = 187.0 Hz), 55.4 ($CH_3$, s), 37.4 ($CH_2$, d, *J* = 20.5 Hz), 26.7 ($CH_3$, s); $^{19}$F NMR (282 MHz, $CDCl_3$) δ −188.4 to −188.1 (m); *m/z* [LRMS, ESI] (relative intensity, M) 235 (100, [M+K]+), 197 (4, [M+H]+), 179 (16, [M+H-$H_2O$]+); [HRMS, ESI] Calcd. $(C_{11}H_{13}FO_2+H)^+$ 197.0972, found 197.0970.

*3-Fluoro-4-(4-cyanophenyl)-butan-2-one* (**7d**): General Procedure C.

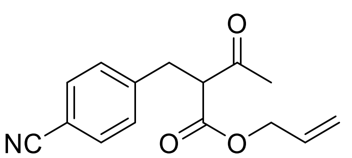

Step 1) *Allyl 3-oxo-2-(4-(cyano)benzyl)butanoate* (**S1-7d**) Reaction performed using 4-(bromomethyl)benzonitrile (980 mg, 5.00 mmol), subsequent purification *via* flash column chromatography (20% EtOAc/cyclohexane) gave **S1-7d** as a colourless oil (503 mg, 39%). $\upsilon_{max}$ (oil) 2942 (w), 2228 (m), 1740 (s), 1716 (s) $cm^{-1}$; $^1$H NMR (700 MHz, $CDCl_3$) δ 7.58 (2H, d, *J* = 8.1 Hz), 7.31 (2H, d, *J* = 8.1 Hz), 5.83 (1H, ddt, *J* = 17.1, 10.7, 6.1 Hz), 5.28-5.24 (2H, m), 4.62-4.57 (2H, m), 3.80 (1H, t, *J* = 7.5 Hz), 3.25 (1H, dd, *J* = 14.2, 7.0 Hz), 3.20 (1H, dd, *J* = 14.2, 8.1 Hz), 2.23 (3H, s); $^{13}$C NMR (176 MHz, $CDCl_3$) δ 201.3 (C), 168.3 (C), 143.9 (C), 132.5 (CH), 131.2 (C), 129.9 (CH), 119.6 ($CH_2$), 118.9 (C), 110.9 (CH), 66.5 ($CH_2$), 60.7 (CH), 33.8 ($CH_2$), 29.8 ($CH_3$); *m/z* [LRMS, ESI] (relative intensity, M) 258 (24, [M+H]+); [HRMS, ESI] Calcd. $(C_{15}H_{15}O_3N+H)^+$ 258.1125, found 258.1122.

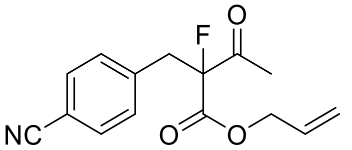


Step 2) *Allyl 2-fluoro-3-oxo-2-(4-cyanobenzyl)butanoate* (**S2-7d**) Reaction performed using **S1-7d** (429 mg, 1.67 mmol), subsequent purification *via* flash column chromatography (25% EtOAc/cyclohexane) gave **S2-7d** as a colourless oil (221 mg, 55%). $\upsilon_{max}$ (oil) 2229 (m), 1754 (m), 1733 (m) $cm^{-1}$; $^1$H NMR (700 MHz, $CDCl_3$) δ 7.59 (2H, d, *J* = 8.0 Hz), 7.34 (2H, d, *J* = 8.0 Hz), 5.83 (1H, ddt, *J* = 16.4, 10.8, 5.9 Hz), 5.32-5.28 (2H, m), 4.68-4.62 (2H, m), 3.52-3.41 (2H, m), 2.19 (3H, d, *J* = 5.0 Hz); $^{13}$C NMR (176 MHz, $CDCl_3$) δ 201.5 (C, d, *J* = 29.3 Hz), 165.0 (C, d, *J* = 25.4 Hz), 138.7 (C, s), 132.3 (CH, s), 131.4 (CH, s), 130.5 (CH, s), 120.1 ($CH_2$, s), 118.7 (C, s), 111.8 (C, s), 99.6 (C, d, *J* = 201.6 Hz), 67.3 ($CH_2$, s), 39.6 ($CH_2$, d, *J* = 20.1), 26.2 ($CH_3$, s); $^{19}$F NMR (659 MHz, $CDCl_3$) δ −164.6 to −164.7 (m); *m/z* [LRMS, ESI] (relative intensity, M) 276 (85, [M+H]+), 232 (21, [M+H-CN-CF(COMe)$CO_2$allyl)]+); [HRMS, ESI] Calcd. $(C_{15}H_{14}FNO_3+H)^+$ 276.1030, found 276.1028.

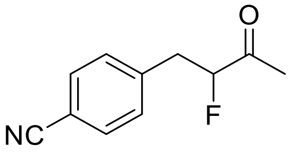


Step 3) *3-Fluoro-4-(4-cyanophenyl)-butan-2-one* (**7d**) Reaction performed using **S2-7d** (255 mg, 0.926 mmol), subsequent purification *via* flash column chromatography (20% $Et_2O$/cyclohexane) gave **7d** as a white solid (132 mg, 75%). m.p. 50-52 °C; $\upsilon_{max}$ (solid) 2928 (w), 2228 (m), 1722 (s), 1609 (m), 1507 (m) $cm^{-1}$; $^1$H NMR (700 MHz, $CDCl_3$) δ 7.62 (2H, d, *J* = 8.2 Hz), 7.36 (2H, d, *J* = 8.2 Hz), 4.92 (1H, ddd, *J* = 49.4, 7.6, 3.7 Hz), 3.26 (1H, ddd, *J* = 28.5, 14.9, 3.7 Hz), 3.12 (1H, ddd, *J* = 25.5, 14.9, 7.6 Hz), 2.19 (3H, d, *J* = 5.0 Hz); $^{13}$C NMR (176 MHz, $CDCl_3$) δ 207.3 (C, d, *J* = 26.2 Hz), 141.0 (C, s), 132.5 (CH, s), 130.5 (CH, s), 118.8 (C, s), 111.4 (C, s), 95.3 (CH, d, *J* = 189.0 Hz), 38.0 ($CH_2$, d, *J* = 20.3 Hz), 26.6 ($CH_3$, s); $^{19}$F NMR (659 MHz, $CDCl_3$) δ −188.7 to −188.5 (m); *m/z* [LRMS, ESI] (relative intensity, M) 171 (100, [M-F]+), 170 (14, [M-HF]+); [HRMS, ESI] Calcd. $(C_{11}H_{10}FNO+H)^+$ 192.0746, loss of HF seen under ESI and EI conditions, Calcd. $(C_{11}H_9NO+H)^+$ 171.0684 found 171.0680.

*3-Fluoro-4-(2-methylphenyl)-butan-2-one* (**7e**):

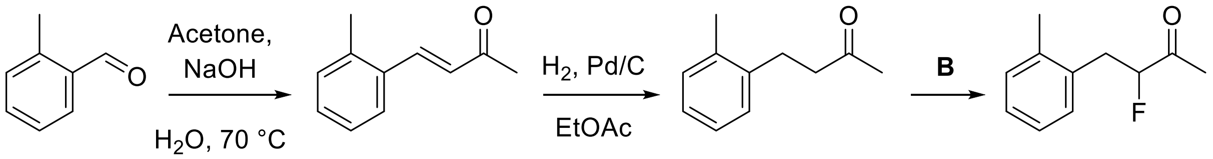


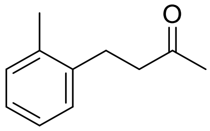


Step 1 and 2) *4-(o-tolyl)butan-2-one* Following the procedures of Cheng *et al.*[89] and Lin[90] A solution of *o*-tolualdehyde (936 µL, 8.06 mmol) and acetone (10 mL) was treated with NaOH solution (1.93 mL, 10% solution in $H_2O$) and stirred at rt for 2 h. After this time the reaction was conc. *in vacuo*, taken up in EtOAc (15 mL), washed $H_2O$ (25 mL), brine (15 mL), dried ($MgSO_4$), filtered and conc. *in vacuo.* The residue was dissolved in EtOAc (9 mL), treated with Pd/C (10%, 100 mg) and back flushed with $H_2$ gas (balloon). The reaction mixture was stirred at rt for 16 h, filtered through celite, washed EtOAc (5 mL) and conc. *in vacuo.* Purification *via* flash column chromatography (5-10% EtOAc/cyclohexane) gave 4-(o-tolyl)butan-2-one as a colourless oil (554 mg, 42%). $^{1}H$ NMR (700 MHz, $CDCl_3$) $\delta$ 7.16-7.12 (4H, m), 2.89 (2H, t, *J* = 8.2 Hz), 2.73 (2H, t, *J* = 8.2 Hz), 2.32 (3H, s), 2.17 (3H, s); $^{13}C$ NMR (176 MHz, $CDCl_3$) $\delta$ 208.2 (C), 139.2 (C), 136.0 (C), 130.4 (CH), 128.7 (CH), 126.4 (CH), 126.3 (CH), 44.0 ($CH_2$), 30.2 ($CH_2$), 19.4 ($CH_3$); [HRMS, ESI] Calcd. $(C_{11}H_{14}O_3+H)^+$ 163.1117, found 163.1114. Data in agreement with that reported in the literature.[90]

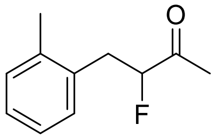


Step 3) *3-Fluoro-4-(o-tolyl)butan-2-one* (**7e**): General Procedure B. Reaction performed using 4-(*o*-tolyl)butan-2-one (217 mg, 1.34 mmol), subsequent purification *via* flash column chromatography (2-5% EtOAc/cyclohexane) gave **7e** as a colourless oil (42 mg, 17%). $\upsilon_{max}$ (oil) 2926 (w), 1722 (s) $cm^{-1}$; $^{1}H$ NMR (400 MHz, $CDCl_3$) $\delta$ 7.22-7.16 (4H, m), 4.92 (1H, ddd, *J* = 50.1, 8.5, 3.5 Hz), 3.21 (1H, ddd, *J* = 31.7, 15.0, 3.5 Hz), 3.05 (1H, ddd, *J* = 22.6, 15.0, 8.5 Hz), 2.19 (3H, d, *J* = 5.0 Hz); $^{13}C$ NMR (176 MHz, $CDCl_3$) $\delta$ 208.2 (C, d, *J* = 26.3 Hz), 136.9 (C, s), 134.0 (C, s), 130.7 (CH, s), 130.2 (CH,s), 127.4 (CH, s), 126.4 (CH, s), 95.9 (CH, d, *J* = 187.3 Hz), 35.3 ($CH_2$, d, *J* = 20.8 Hz), 26.3 ($CH_3$, s); $^{19}F$ NMR (659 MHz, $CDCl_3$) $\delta$ −187.8 to −188.9 (m); *m/z* [LRMS, ESI] (relative intensity, M) 181 (3, [M+H]+), 163 (100, [M+H-F]+), 143 (14); [HRMS, ESI] Calcd. $(C_{11}H_{13}FO+H)^+$ 181.1023, Calcd. found 181.1020.

*3-Fluoro-4-(3-trifluoromethylphenyl)-butan-2-one* (**7f**): General Procedure C.

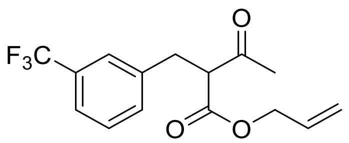

Step 1) *Allyl 3-oxo-2-(3-(trifluoromethyl)benzyl)butanoate* (**S1-7f**) Reaction performed using 3-trifluoromethylbenzyl bromide (1.63 mL, 10.0 mmol), subsequent purification *via* flash column chromatography (0-15% EtOAc/cyclohexane) gave **S1-7f** as a colourless oil as a 24:1 mixture of keto:enol forms (2.63 g, 88%). $\upsilon_{max}$ (oil) 1740 (m), 1716 (m) $cm^{-1}$; *m/z* [LRMS, ESI] (relative intensity, M) 301 (100, [M+H]+), 283 (16, [M+H-F]+), 199 (7, [M+H-$CO_2$allyl-Me]+); [HRMS, ESI] Calcd. $(C_{15}H_{15}F_3O_3+H)^+$ 301.1046, found 301.1042.

Keto Tautomer: $^1$H NMR (700 MHz, $CDCl_3$) $\delta$ 7.48 (1H, d, *J* = 7.2 Hz), 7.45 (1H, s), 7.41-7.37 (2H, m), 5.82 (1H, ddt, *J* = 16.5, 11.6, 5.8 Hz), 5.27-5.23 (2H, m), 4.59 (2H, d, *J* = 5.8 Hz), 3.81 (1H, app t, *J* = 7.6 Hz), 3.24 (1H, dd, *J* = 14.2, 7.0 Hz), 3.21 (1H, dd, *J* = 14.2, 8.2 Hz), 2.23 (3H, s); $^{13}$C NMR (176 MHz, $CDCl_3$) $\delta$ 201.6 (C, s), 168.5 (C, s), 139.2 (C, s), 132.5 (CH, d, *J* = 0.8 Hz), 131.2 (CH, s), 131.0 (C, q, *J* = 32.2 Hz), 129.2 (CH, s), 125.6 (CH, q, *J* = 3.7 Hz), 124.1 (C, q, *J* = 272.2 Hz), 123.7 (CH, d, *J* = 3.7 Hz), 119.4 ($CH_2$, s), 66.4 ($CH_2$, s), 61.1 (CH, s), 33.6 ($CH_2$, s), 29.7 ($CH_3$, s); $^{19}$F NMR (659 MHz, $CDCl_3$) $\delta$ −62.7 (s).

Enol Tautomer: $^1$H NMR (700 MHz, $CDCl_3$) $\delta$ 12.89 (1H, br), 7.55-7.30 (4H, m), 5.85-5.79 (1H, m), 5.18-5.16 (2H, m), 4.63-4.62 (2H, m), 3.65 (2H, s), 2.08 (3H, s); $^{13}$C NMR (176 MHz, $CDCl_3$) $\delta$ 174.4 (C, s), 172.6 (C, s), 142.0 (C, s), 131.8 (CH, s), 131.2 (CH, s), 128.8 (CH, s), 124.7 (C, q, *J* = 3.8 Hz), 123.0 (CH, q, *J* = 3.8 Hz), 118.3 ($CH_2$, s), 98.7 (C, s), 65.2 ($CH_2$, s), 31.7 ($CH_2$, s), 19.3 ($CH_3$, s), 2 x C not seen due to low intensity of enol tautomer; $^{19}$F NMR (659 MHz, $CDCl_3$) $\delta$ −62.6 (s).

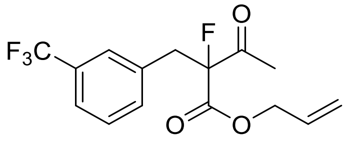

Step 2) *Allyl 2-fluoro-3-oxo-2-(3-(trifluoromethyl)benzyl)butanoate* (**S2-7f**) Reaction performed using **S1-7f** (1.00 g, 3.33 mmol), subsequent purification *via* flash column chromatography (0-10% EtOAc/cyclohexane) gave **S2-7f** as a colourless oil (868 mg, 82%). $\upsilon_{max}$ (oil) 1756 (m), 1735 (m) $cm^{-1}$; $^1$H NMR (700 MHz, $CDCl_3$) $\delta$ 7.55 (1H, dd, *J* = 3.5, 4.3 Hz), 7.50 (1H, s), 7.42 (2H, d, *J* = 5.0 Hz), 5.83 (1H, ddt, *J* = 16.9, 11.7, 5.9 Hz), 5.31 (1H, dd, *J* = 16.9, 1.1 Hz), 5.28 (1H, dd, *J* = 11.7, 5.9), 4.68-4.62 (2H, m), 3.49 (1H, dd, *J* = 63.2, 25.4 Hz), 3.46 (1H, dd, *J* = 62.2, 24.6 Hz), 2.20 (3H, d, *J* = 5.1 Hz); $^{13}$C NMR (176 MHz, $CDCl_3$) $\delta$ 201.7 (C, d, *J* = 29.1 Hz), 165.2 (C, d, *J* = 25.5 Hz), 134.2 (C, s), 130.5 (CH, s), 129.0 (CH, s), 130.9 (C, q, *J* = 32.3 Hz), 127.3 (CH, q, *J* = 3.5 Hz), 124.6 (CH, q, *J* = 3.7 Hz), 124.1 (C, q, *J* = 272.3 Hz), 120.1 ($CH_2$, s), 99.8 (C, d, *J* = 200.9 Hz), 67.2 ($CH_2$, s), 39.4 ($CH_2$, d, *J* = 20.2), 26.2 ($CH_3$, s); $^{19}$F NMR (282 MHz, $CDCl_3$) $\delta$ −62.7 (3F, s), −164.8 (1F, tq, *J* =25.0, 4.9 Hz); *m/z* [LRMS, ESI] (relative intensity, M) 319 (44, [M+H]+), 199 (63, [M+H-$CO_2$allyl-F-Me]+); [HRMS, ESI] Calcd. $(C_{11}H_{10}F_4O_3+H)^+$ 319.0952, found 319.0949.

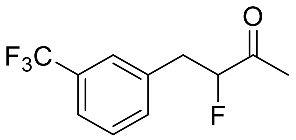


Step 3) *3-Fluoro-4-(3-trifluoromethylphenyl)-butan-2-one* (**7f**) Reaction performed using **S2-7f** (255 mg, 0.800 mmol), subsequent purification *via* flash column chromatography (30% $CH_2Cl_2$/pentane) gave **7f** as a colourless oil (40 mg, 21%). $\upsilon_{max}$ (oil) 1725 (m) $cm^{-1}$; $^1H$ NMR (700 MHz, $CDCl_3$) δ 7.54 (1H, d, *J* = 7.5 Hz), 7.51 (1H, s), 7.46-7.42 (2H, m), 4.95 (1H, ddd, *J* = 49.6, 7.8, 3.6 Hz), 3.26 (1H, ddd, *J* = 29.2, 14.5, 3.6 Hz), 3.12 (1H, ddd, *J* = 25.2, 14.5, 7.8 Hz), 2.19 (3H, d, *J* = 5.0 Hz); $^{13}C$ NMR (176 MHz, $CDCl_3$) δ 207.6 (C, d, *J* = 26.2 Hz), 136.5 (C, s), 133.0 (CH, s), 131.1 (C, q, *J* = 32.3 Hz), 129.2 (CH, s), 126.4 (CH, q, *J* = 3.7 Hz), 124.2 (CH, q, *J* = 3.7 Hz), 124.1 (C, q, *J* = 272.3 Hz), 95.5 (CH, d, *J* = 188.0 Hz), 37.8 ($CH_2$, d, *J* = 20.5 Hz), 26.5 ($CH_3$, s); $^{19}F$ NMR (659 MHz, $CDCl_3$) δ −62.7 (3F, s), −188.6 to −188.7 (1F, m); *m/z* [LRMS, EI] (relative intensity, M) 235 (2, [M+H]+), 215 (52, M-F]+), 214 (100, [M-HF]+), 199 (50, [M-F,Me]+), 171 (31, [M-CHFC(O)Me]+), 159 (29, HFC(O)Me); [HRMS, ESI] Calcd. $(C_{11}H_{10}F_4O+H)^+$ 235.0741, found 235.0735.

*3-Fluoro-4-(pyridin-3-yl)butan-2-one* (**8a**):

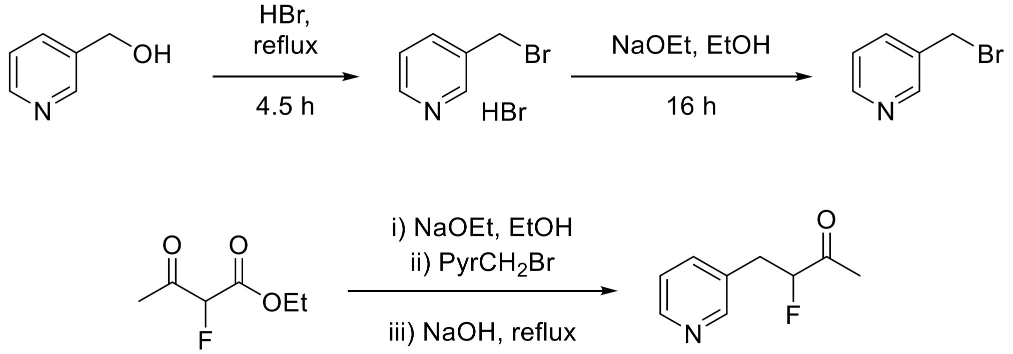


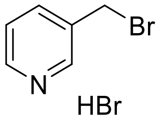


Step 1) *3-(Bromomethyl)pyridine hydrobromide*: Following the procedure of Wittenberger *et al.*:[91] A solution of 3-pyridinecarbinol (1.00 g, 9.16 mmol) in HBr (9 mL, 48% solution) was heated to 125 °C for 4.5 h. After this time the reaction was cooled and conc. *in vacuo*. The resultant solid was washed with EtOH until the filtrate was colourless. 3-(Bromomethyl)pyridine hydrobromide was isolated as a white solid (1.41 g, 61%, Lit.[20] 55%) and used immediately in the next step. $^1H$ NMR (400 MHz, DMSO) δ 8.98 (1H, s), 8.80 (1H, d, *J* = 5.5 Hz), 7.92 (1H, d, *J* = 8.0 Hz), 7.50 (1H, dd, *J* = 8.0, 5.5 Hz), 4.86 (2H, s). Data in agreement with that reported in the literature.[92]

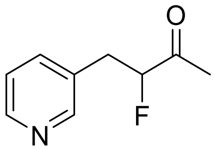

Step 2) *3-Fluoro-4-(pyridin-3-yl)butan-2-one* (**8a**): Following an analogous procedure to that of Sanford *et al.* for the synthesis of ethyl 2-benzyl-2-fluoro-3-oxobutanoate.[93] A solution of ethyl α-fluoroacetate (480 mg, 3.20 mmol) in EtOH (1 mL) was treated with NaOEt (2.40 mL, 21% solution in EtOH) and stirred for 10 min. In a separate flask, 3-(bromomethyl)pyridine hydrobromide (1.50 g, 5393 mmol) was suspended in EtOH (1 mL) and treated with NaOEt (1.30 mL, 21% solution in EtOH) followed by the deprotonated ethyl α-fluoroacetate solution. The resultant suspension was stirred at rt for 16 h. After this time, a solution of NaOH (0.16 g) in $H_2O$ (2.4 mL) was added and the resultant solution heated to reflux for 3 h. After cooling to rt $H_2O$ (5 mL) was added and the solution extracted with $CH_2Cl_2$ (2 x 10 mL). The organics were dried ($MgSO_4$), filtered and conc. *in vacuo*. Purification *via* flash column chromatography (5%-50% EtOAc/cyclohexane) gave **8a** as a colourless oil (67 mg, 13%). $υ_{max}$ (oil) 2927 (w), 1724 (s), 1576 (m) cm$^{-1}$; $^{1}$H NMR (700 MHz, $CDCl_3$) δ 8.52 (1H, dd, *J* = 4.8, 1.7 Hz), 8.49 (1H, d, *J* = 1.7 Hz), 7.57-7.54 (1H, m), 7.24 (1H, dd, *J* = 7.8, 4.8 Hz), 4.92 (1H, ddd, *J* = 49.4, 7.6, 3.8 Hz), 3.18 (1H, ddd, *J* = 28.5, 15.0, 3.8 Hz), 3.05 (1H, ddd, *J* = 25.8, 15.0, 7.6 Hz), 2.17 (3H, d, *J* = 5.0 Hz); $^{13}$C NMR (176 MHz, $CDCl_3$) δ 207.5 (C, d, *J* = 26.1 Hz), 150.8 (CH, s), 148.8 (CH, s), 137.2 (CH, s), 131.0 (C, s), 123.5 (CH, s), 95.4 (CH, d, *J* = 188.0 Hz), 35.2 ($CH_2$, d, *J* = 20.5 Hz), 26.6 ($CH_3$, s); $^{19}$F NMR (659 MHz, $CDCl_3$) δ −189.0 to −188.8 (m); *m/z* [LRMS, ESI] (relative intensity, M) 168 (100, [M+H]+); [HRMS, ESI] Calcd. $(C_9H_{10}FON+H)^+$ 168.0819, found 168.0817.

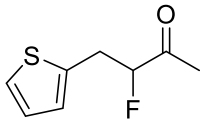

*3-Fluoro-4-(thiophen-2-yl)butan-2-one* (**8b**): Synthetic routes used for other reference compounds were unsuccessful for the thiophene. Large scale enzyme reaction gave 16 mg of **8b** (see Preparative Scale section for conditions). $υ_{max}$ (oil) 2923 (m), 1723 (s), 1421 (m) cm$^{-1}$; $^{1}$H NMR (600 MHz, $CDCl_3$) δ 7.19 (1H, dd, *J* = 5.1, 1.2 Hz), 6.94 (1H, dd, *J* = 5.2, 3.4 Hz), 6.91 – 6.83 (1H, m), 4.91 (1H, ddd, *J* = 49.5, 6.8, 3.8 Hz), 3.43-3.24 (2H, m), 2.16 (3H, d, *J* = 4.9 Hz); $^{13}$C NMR (151 MHz, $CDCl_3$) δ 207.8 (C, d, *J* = 26.2 Hz), 136.5 (C, s), 127.2 (CH, s), 127.1 (CH, s), 125.1 (CH, s), 95.3 (CH, d, *J* = 188.2 Hz), 32.3 ($CH_2$, d, *J* = 21.1 Hz), 26.7 ($CH_3$, s). $^{19}$F NMR (376 MHz, $CDCl_3$) δ -187.4 to -187.8 (m). *m/z* [HRMS, ESI] Calcd. $(C_8H_9FOS+H)^+$ 173.0431, found 173.0428.

*4-Cyclohexyl-3-fluorobutan-2-one* (**9a**): General Procedure B.

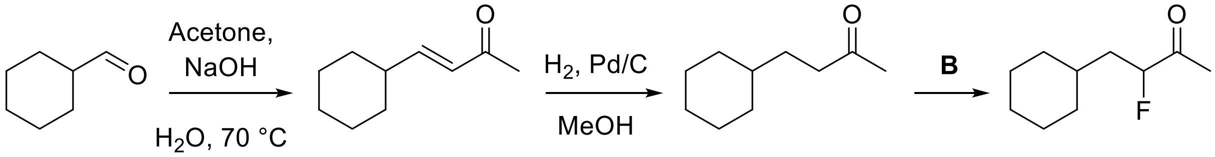

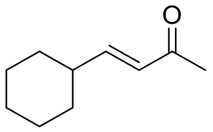

Step 1) *(E)-4-Cyclohexylbut-3-en-2-one* Following the procedure of Goldfarb *et al.*:[94] A solution of cyclohexane carboxaldeyhde (1.00 g, 8.92 mmol) and acetone (1.36 mL, 29.8 mmol) in $H_2O$ (1.80 mL) was treated with NaOH (2.20 mL, 1% solution in $H_2O$) and heated to 70 °C for 4 h. After this time the reaction was cooled to rt, $H_2O$ (1.5 mL) added, extracted into $Et_2O$ (15 mL), dried ($MgSO_4$), filtered and conc. *in vacuo.* Purification *via* flash column chromatography (10% $Et_2O$/pentane) gave (*E*)-4-cyclohexylbut-3-en-2-one as a colourless oil (863 mg, 64%). $^1H$ NMR (700 MHz, $CDCl_3$) δ 6.73 (1H, dd, *J* = 16.1, 6.8 Hz), 6.02 (1H, d, *J* = 16.1 Hz), 2.25 (3H, s), 2.18-2.11 (1H, m), 1.80-1.75 (4H, m), 1.72-1.65 (1H, m), 1.35-1.27 (2H, m), 1.23-1.11 (3H, m); $^{13}C$ NMR (176 MHz, $CDCl_3$) δ 199.3(C), 153.6 (CH), 129.0 (CH), 40.7 (CH), 31.9 ($CH_3$), 27.0 ($CH_2$), 26.0 ($CH_2$), 25.8 ($CH_2$); [HRMS, ESI] Calcd. $(C_{10}H_{16}O+H)^+$ 153.1274, found 153.1273. Data in agreement with that reported in the literature.[95]

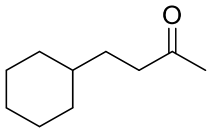

Step 2) *4-Cyclohexylbutan-2-one* Following the procedure of Imoto *et al.*:[96] A solution of (*E*)-4-cyclohexylbut-3-en-2-one (800 mg, 5.25 mmol) in MeOH (8 mL) was treated with Pd/C (10%, 120 mg) and back flushed with $H_2$ gas (balloon). The reaction mixture was stirred at rt for 16 h, filtered through celite, washed MeOH (5 mL) and conc. *in vacuo.* Purification *via* flash column chromatography (10% $Et_2O$/pentane) gave 4-cyclohexylbutan-2-one as a colourless oil (782 mg, 96%). $^1H$ NMR (500 MHz, $CDCl_3$) δ 2.43 (2H, t, *J* = 15.6 Hz), 2.14 (3H, s), 1.71-1.61 (5H, m), 1.49-1.30 (2H, m), 1.26-1.11 (4H, m), 0.93-0.85 (2H, m); $^{13}C$ NMR (125 MHz, $CDCl_3$) δ 209.8 (C), 41.5 ($CH_2$), 37.4 (CH), 33.2 ($CH_2$), 31.4 ($CH_2$), 30.0 ($CH_3$), 26.7 ($CH_2$), 36.4 ($CH_2$); [HRMS, ESI] Calcd. $(C_{10}H_{18}O+H)^+$ 155.1430, found 155.1427. Data in agreement with that reported in the literature.[97]

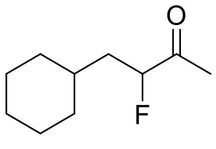

Step 3) *4-Cyclohexyl-3-fluorobutan-2-one* (**9a**): General Procedure B. Reaction performed using 4-cyclohexylbutan-2-one (270 mg, 1.75 mmol), subsequent purification *via* flash column chromatography (5% $Et_2O$/pentane) gave **9a** as a colourless oil (155 mg, 52%). $^1H$ NMR (700 MHz, $CDCl_3$) δ 4.85 (1H, ddd, *J* = 51.1, 9.9, 34 Hz), 2.26 (3H, d, *J* = 4.7 Hz), 1.82-1.51 (8H, m), 1.31-1.13 (3H, m), 1.01-0.87 (2H, m); $^{13}C$ NMR (176 MHz, $CDCl_3$) δ 208.9 (C, d, *J* = 25.5 Hz), 94.6 (CH, d, *J* = 183.4 Hz), 39.3 ($CH_2$, d, *J* = 20.4 Hz), 34.0 ($CH_2$, s), 33.8 (CH, s), 32.3 ($CH_2$, s), 26.4 ($CH_2$, s), 26.3 ($CH_2$, s), 26.1 ($CH_2$, s), 25.7 ($CH_3$, s); $^{19}F$ NMR (659 MHz, $CDCl_3$) δ −188.3 to −188.1 (m); [HRMS, ESI] Calcd. $(C_{10}H_{17}FO\text{-}H)^+$ 171.1191, found 171.1176. Data in agreement with that reported in the literature.[34]

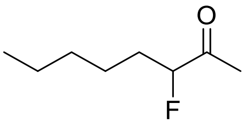

*3-Fluoro-4-phenylbutan-2-one* (**9b**): General Procedure B. Reaction performed using 2-octanone (321 µL, 2.05 mmol), subsequent purification *via* flash column chromatography (10% $Et_2O$/pentane) gave **9b** as a colourless oil (65 mg, 22% (61%, brsm)). $^1$H NMR (700 MHz, $CDCl_3$) δ 4.80-4.64 (1H, m), 2.26 (3H, d, *J* = 4.8 Hz), 1.84-1.72 (2H, m), 1.49-1.42 (2H, m), 1.33-1.21 (4H, m), 0.91-0.87 (3H, m); $^{13}$C NMR (176 MHz, $CDCl_3$) δ 208.6 (C, d, *J* = 25.7 Hz), 96.1 (CH, d, *J* = 183.7 Hz), 31.9 ($CH_2$, d, *J* = 20.6 Hz), 31.4 ($CH_2$, s), 26.0 ($CH_2$, s), 24.2 ($CH_2$, d, *J* = 2.9Hz), 22.5 ($CH_2$, s), 14.0 ($CH_3$, s); $^{19}$F NMR (659 MHz, $CDCl_3$) δ −189.4 to −189.6 (m); *m/z* [LRMS, EI] Calcd. $(C_8H_{15}FO)^+$ 146, found 146. Data in agreement with that reported in the literature.[98]

*3-Fluoro-5-phenylpentan-2-one* (**9c**): General Procedure B.

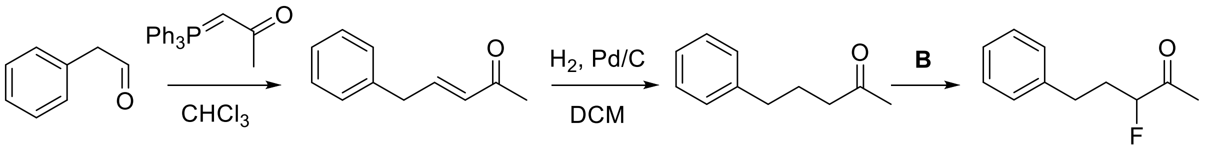


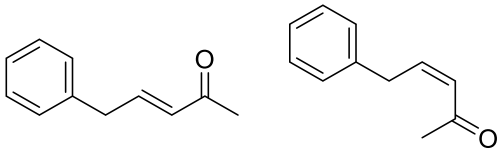

Step 1) *(E/Z)-5-Phenylpent-3-en-2-one* A solution of 2-phenylacetaldehyde (768 µL, 6.90 mmol) in $CHCl_3$ (30 mL) was treated with 1-triphenylphosphoranylidene-2-propanone (1.91 g, 6.00 mmol) and heated to reflux for 4 h. After this time the reaction was cooled to rt, $H_2O$ extracted with $CHCl_3$, washed with brine, dried ($MgSO_4$), filtered and conc. *in vacuo.* Purification *via* flash column chromatography (0-10% EtOAc/Pet. Ether) gave 5-phenylpent-3-en-2-one as a colourless oil (827 mg, 86%) as a 2:1 mixture of *E*:*Z* isomers. (*E*) Isomer: $^1$H NMR (400 MHz, $CDCl_3$) δ 7.39-7.16 (5H, m), 6.92 (1H, dt, *J* = 15.8, 6.8 Hz), 6.09 (1H, d, *J* = 15.8 Hz), 3.54 (2H, d, *J* = 6.8 Hz), 2.24 (3H, s); $^{13}$C NMR (101 MHz, $CDCl_3$) δ 198.7 (C), 146.5 (CH), 137.8 (C), 132.2 (CH), 128.94 (CH), 128.90 (CH), 126.9 (CH), 38.9 ($CH_2$), 27.1 ($CH_3$); (*Z*) Isomer: $^1$H NMR (400 MHz, $CDCl_3$) δ 7.39-7.16 (5H, m), 6.48 (1H, d, *J* = 15.9 Hz), 6.31 (1H, dt, *J* = 15.9, 7.1 Hz), 3.34 (2H, d, *J* = 7.1 Hz), 2.22 (3H, s); $^{13}$C NMR (101 MHz, $CDCl_3$) δ 206.8 (C), 137.0 (C), 134.0 (CH), 128.7 (CH), 127.7 (CH), 126.4 (CH), 122.0 (CH), 38.9 ($CH_2$), 27.1 ($CH_3$); m/z [LRMS, EI] Calcd. $(C_{11}H_{12}O)^+$ 160, found 160. Data in agreement with that reported in the literature.[99]

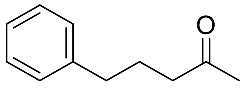

Step 2) *5-phenylpentan-2-one* A solution of (*E/Z*)- 5-phenylpent-3-en-2-one (410 mg, 2.56 mmol) in $CH_2Cl_2$ (20 mL) was treated with Pd/C (10%, 40 mg) and back flushed with $H_2$ gas (balloon). The reaction mixture was stirred at

rt for 16 h, filtered, washed $CH_2Cl_2$ and conc. *in vacuo.* Purification *via* flash column chromatography (0-10% EtOAc/pet. ether) gave 5-phenylpentan-2-one as a colourless oil (357 mg, 86%). $^1$H NMR (400 MHz, $CDCl_3$) δ 7.31-7.27 (2H,m), 7.22-7.17 (3H, m), 2.63 (2H, *t*, *J* = 7.5 Hz), 2.45 (2H, t, *J* = 7.5 Hz), 2.13 (3H, s), 1.92 (2H, app quin, *J* = 7.6 Hz); $^{13}$C NMR (101 MHz, $CDCl_3$) δ 208.9 (C), 141.7 (C), 128.6 (CH), 128.5 (CH), 126.1 (CH), 43.0 ($CH_2$), 35.2 ($CH_2$), 30.1 ($CH_2$), 25.3 ($CH_3$); [LRMS, EI] Calcd. $(C_{11}H_{14}O)^+$ 162, found 162. Data in agreement with that reported in the literature.[100]

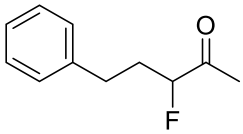


*3-Fluoro-5-phenylpentan-2-*one (**9c**): General Procedure B. Reaction performed using 5-phenylpentan-2-one (100 mg, 0.62 mmol), subsequent purification *via* flash column chromatography (5% EtOAc/pet. ether) gave **9c** as a colourless oil (63 mg, 56%). $^1$H NMR (400 MHz, $CDCl_3$) δ 7.34-7.28 (2H, m), 7.25-7.19 (3H, m), 4.67 (1H, ddd, *J* = 50.1, 8.2, 4.6 Hz), 2.88-2.71 (2H, m), 2.27 (3H, d, *J* = 4.6 Hz), 2.21-2.03 (2H, m); $^{13}$C NMR (101 MHz, $CDCl_3$) δ 208.3 (C, d, *J* = 25.6 Hz), 140.3 (C, s), 128.71 (CH, s), 128.67 (CH, s), 126.5 (CH, s), 95.1 (CH, d, *J* = 184.3 Hz), 33.5 ($CH_2$, d, *J* = 20.7 Hz), 30.7 ($CH_2$, d, *J* = 3.4 Hz), 26.0 ($CH_3$, s); $^{19}$F NMR (377 MHz, $CDCl_3$) δ −190.9 to −190.6 (m). Data in agreement with that reported in the literature.[101]

*3-Fluoro-6-phenylhexan-2-one* (**9d**): General Procedure B.

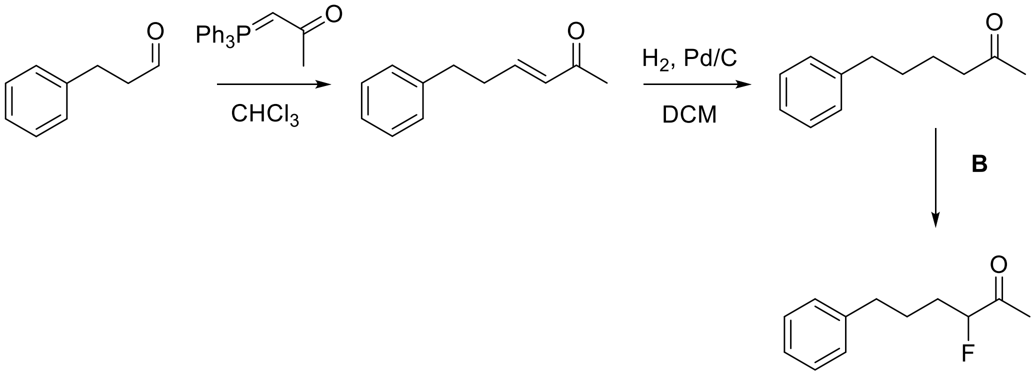


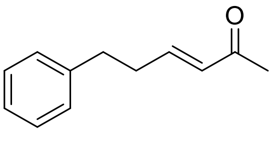


Step 1) *(E)-6-Phenylhex-3-en-2-one* A solution of 3-phenylpropanal (917 µL, 6.90 mmol) in $CHCl_3$ (30 mL) was treated with 1-triphenylphosphoranylidene-2-propanone (1.91 g, 6.00 mmol) and heated to reflux for 4 h. After this time the reaction was cooled to rt, $H_2O$ extracted with $CHCl_3$, washed with brine, dried ($MgSO_4$), filtered and conc. *in vacuo.* Purification *via* flash column chromatography (0-10% EtOAc/Pet. Ether) gave 5-phenylpent-3-en-2-one as a colourless oil (858 mg, 82%) as a single isomer. $^1$H NMR (400 MHz, $CDCl_3$) δ 7.33-7.29 (2H, m), 7.24-

7.18 (3H, m), 6.83 (1H, dt, *J* = 16.0, 6.8 Hz), 6.10 (1H, d, *J* = 16.0 Hz), 2.79 (2H, t, *J* = 8.0 Hz), 2.56 (2H, app q, *J* = 7.5 Hz), 2.24 (3H, s); $^{13}$C NMR (101 MHz, $CDCl_3$) δ 198.7 (C), 147.2 (CH), 140.8 (C), 131.8 (CH), 128.5 (CH), 126.4 (CH), 34.6 ($CH_2$), 34.2 ($CH_2$), 27.0 ($CH_3$); m/z [LRMS, EI] Calcd. $(C_{12}H_{14}O)^+$ 174, found 174. Data in agreement with that reported in the literature.[102]

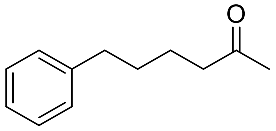

Step 2) *6-Phenylhexan-2-one* A solution of (*E/Z*)- 5-phenylpent-3-en-2-one (719 mg, 4.08 mmol) in $CH_2Cl_2$ (20 mL) was treated with Pd/C (10%, 70 mg) and back flushed with $H_2$ gas (balloon). The reaction mixture was stirred at rt for 16 h, filtered, washed $CH_2Cl_2$ and conc. *in vacuo.* Purification *via* flash column chromatography (0-10% EtOAc/pet. ether) gave 6-phenylhexan-2-one as a colourless oil (662 mg, 90%). $^1$H NMR (400 MHz, $CDCl_3$) δ 7.30-7.27 (2H, m), 7.20-7.17 (3H, m), 2.66-2.60 (2H, m), 2.49-2.42 (2H, m), 2.13 (3H, s), 1.66-1.59 (4H, m); $^{13}$C NMR (125 MHz, $CDCl_3$) δ 209.1 (C), 142.3 (C), 128.5 (CH), 128.4 (CH), 125.9 (CH), 43.7 ($CH_2$), 35.9 ($CH_2$), 31.0 ($CH_2$), 30.0 ($CH_2$), 25.6 ($CH_3$); m/z [LRMS, EI] Calcd. $(C_{12}H_{16}FO)^+$ 176, found 176. Data in agreement with that reported in the literature.[103]

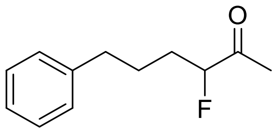

*3-Fluoro-6-phenylhexan-2-one* (**9d**): General Procedure B. Reaction performed using 6-phenylhexan-2-one (109 mg, 0.62 mmol), subsequent purification *via* flash column chromatography (5% EtOAc/pet. ether) gave **9d** as a colourless oil (89 mg, 74%). $^1$H NMR (400 MHz, $CDCl_3$) δ 7.32-7.27 (2H, m), 7.22-7.17 (3H, m), 4.75 (dt, *J* = 54.5, 7.5 Hz), 2.68-2.65 (2H, m), 2.25 (3H, d, *J* = 4.8 Hz), 1.91-1.76 (4H, m);$^{13}$C NMR (176 MHz, $CDCl_3$) δ 208.4 (C, d, *J* = 25.8 Hz), 141.6 (C), 128.6 (CH), 128.5 (CH), 126.1 (CH), 95.9 (CH, d, *J* = 184.1 Hz), 35.5 ($CH_2$, s), 31.5 ($CH_2$, d, *J* = 20.8 Hz), 26.3 ($CH_3$, d, *J* = 3.0 Hz), 26.0 ($CH_2$, s); $^{19}$F NMR (377 MHz, $CDCl_3$) δ −189.6 to −188.4 (m); m/z [LRMS, EI] Calcd. $(C_{12}H_{15}FO)^+$ 194, found 194. Data in agreement with that reported in the literature for the (*S*)-enantiomer.[34]

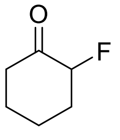

*2-Fluorocyclohexan-1-one* (**10a**): A solution of cyclohexanone (1.03 mL, 10.0 mmol) in MeCN (120 mL) was treated with Selectfluor (4.25 g, 12.0 mmol) and heated to reflux for 24 h. After this time the solution was cooled to rt, conc. *in vacuo*. The residue was taken up in $CH_2Cl_2$ (20 mL), filtered, washed with $NaHCO_{3\,(sat.aq)}$ (20 mL) and brine (20 mL). The organics were dried ($MgSO_4$), filtered and conc. *in vacuo*. Purification *via* flash column chromatography (10%-50% $Et_2O$/pentane) gave **10a** as a colourless oil (759 mg, 65%). $^1$H NMR (600 MHz, $CDCl_3$) δ 4.90 (1H, ddd, *J* = 48.9, 11.5, 6.3 Hz), 2.60-2.54 (1H, m), 2.47-2.40 (1H, m), 2.36-2.30 (1H, m), 2.07-1.96 (2H, m),

1.92-1.83 (1H, m), 1.76-1.64 (2H, m); $^{13}C$ NMR (151 MHz, $CDCl_3$) δ 205.8 (C, d, *J* = 14.5 Hz), 92.8 (CH, d, *J* = 190.4 Hz), 40.3 ($CH_2$, s), 34.3 ($CH_2$, d, *J* = 18.5 Hz), 27.0 ($CH_2$, d, *J* = 0.8 Hz), 22.8 ($CH_2$, d, *J* = 9.7 Hz); $^{19}F$ NMR (377 MHz, $CDCl_3$) δ −188.3 to −188.1 (m); [HRMS, ESI] Calcd. $(C_6H_9FO+H)^+$ 117.0710, found 117.0713. Data in agreement with that reported in the literature.[104]

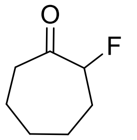


*2-Fluorocycloheptan-1-one* (**10b**): A solution of cycloheptanone (849 mg, 7.50 mmol) in MeCN (90 mL) was treated with Selectfluor (3.19 g, 9.00 mmol) and heated to reflux for 15 h. After this time the solution was conc. *in vacuo* and redissolved in $CH_2Cl_2$ (40 mL), filtered, solid washed with $CH_2Cl_2$ (20 mL) and the combined organics washed with $NaHCO_{3(aq)}$ (30 mL), dried ($MgSO_4$), filtered and conc. *in vacuo*. Purification *via* flash column chromatography (30% $Et_2O$/hexane) gave **10b** as a colourless oil (976 mg, 63%). $^1H$ NMR (400 MHz, $CDCl_3$) δ 5.11-4.96 (1H, m), 2.57-2.50 (2H, m), 2.06-1.47 (8H, m); $^{13}C$ NMR (101 MHz, $CDCl_3$) δ 208.7 (C, d, *J* = 18.4 Hz), 95.3 (CH, d, *J* = 184.8 Hz), 39.9 ($CH_2$, s), 31.3 ($CH_2$, d, *J* = 21.9 Hz), 28.2 ($CH_2$), 25.1 ($CH_2$, d, *J* = 6.7 Hz), 23.0 ($CH_2$); $^{19}F$ NMR (377 MHz, $CDCl_3$) δ −184.53 to −184.2 (m); m/z [HRMS, ESI] Calcd. $(C_7H_{11}FO+H)^+$ 131.0867, found 131.0870. Data in agreement with that reported in the literature.[105]

*2-Fluoro-1-phenylpentan-3-one* (**11a**): General Procedure C.

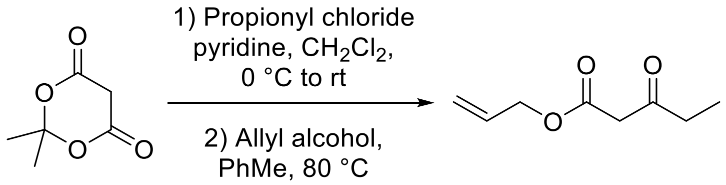


*Allyl 3-oxopentanoate*: Following the procedure of Spreitzer *et al.*:[106] A solution of 2,2-dimethyl-1,3-dioxane-4,6-dione(2.00 g, 13.9 mmol) in anhydrous $CH_2Cl_2$ (28 mL) was treated with propionyl chloride (1.42 g, 15.3 mmol) and cooled to 0 °C. Pyridine (2.24 mL, 27.8 mmol) was added dropwise and the resultant orange solution stirred at 0 °C for 1 h and then warmed to rt for 1 h. After this time the reaction mixture was washed 1 M $HCl_{(aq.)}$ (30 mL), $H_2O$ (30 mL), dried ($Na_2SO_4$), filtered and conc. *in vacuo*. The crude residue was taken up in anhydrous PhMe (40 mL), treated with allyl alcohol (2.84 mL, 41.5 mmol) and heated to 80 °C for 19 h. After cooling to rt the solution was conc. *in vacuo*. Purification *via* flash column chromatography (0-20% EtOAc/cyclohexane) gave allyl 3-oxopentanoate as a colourless oil (1.25 g, 58%). $^1H$ NMR (700 MHz, $CDCl_3$) δ 5.95-5.88 (1H, m), 5.34 (1H, d, *J* = 17.2 Hz), 5.26 (1H, d, *J* = 10.4 Hz), 4.63 (2H, d, *J* = 5.8 Hz), 3.48 (2H, s), 2.57 (2H, q, *J* = 7.3 Hz), 1.09 (3H, t, *J* = 7.3 Hz); $^{13}C$ NMR (176 MHz, $CDCl_3$) δ 203.2 (C), 167.2 (C), 131.7 (CH), 118.9 ($CH_2$), 66.1 ($CH_2$), 49.0 ($CH_2$), 36.5 ($CH_2$), 7.7 ($CH_3$); [HRMS, ESI] Calcd. $(C_8H_{12}O_3-H)^-$ 155.0714 found 155.0704. Data in agreement with that reported in the literature.[107]

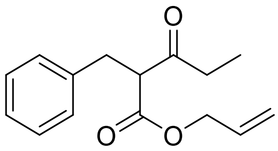

Step 1) *Allyl 2-benzyl-3-oxopentanoate* (**S1-11a**) Reaction performed using benzyl bromide (0.560 mL, 5.82 mmol) and allyl 3-oxopentanoate (1.00 g, 6.40 mmol), subsequent purification *via* flash column chromatography (1-10% EtOAc/cyclohexane) gave **S1-11a** as a colourless oil (633 mg, 44%), trace enol form present. $\upsilon_{max}$ (oil) 1740 (s), 1713 (s) $cm^{-1}$; $^{1}H$ NMR (700 MHz, $CDCl_3$) $\delta$ 7.29-7.26 (2H, m), 7.23-7.20 (1H, m), 7.18 (2H, d, *J* = 7.2 Hz), 5.84 (1H, ddt, *J* = 16.5, 10.9, 5.7 Hz), 5.28-5.20 (2H, m), 4.59 (2H, d , *J* = 5.8 Hz), 3.84 (1H, t, *J* = 7.6 Hz), 3.20 (1H, dd, *J* = 14.1, 7.7 Hz), 3.18 (1H, dd, *J* = 14.1, 7.7 Hz), 2.58 (1H, dq, *J* = 18.3, 7.2 Hz), 2.34 (1H, dq, *J* = 18.3, 7.2 Hz), 1.00 (3H, t, *J* = 7.2 Hz); $^{13}C$ NMR (176 MHz, $CDCl_3$) $\delta$ 205.3 (C), 169.0 (C), 138.3 (C), 131.6 (CH), 128.9 (CH), 128.7 (CH), 126.8 (CH), 118.9 ($CH_2$), 66.1 ($CH_2$), 60.4 (CH), 36.4 ($CH_2$), 34.3 ($CH_2$), 7.6 ($CH_3$); *m/z* [LRMS, ESI] (relative intensity, M) 247 (100, [M+H]+), 229 (34, [M+H-$CH_3$]+); [HRMS, ESI] Calcd. $(C_{15}H_{18}O_3+H)^+$ 247.1329 found 247.1323.

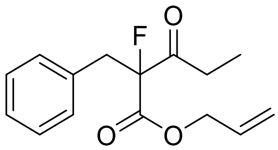

Step 2) *Allyl 2-benzyl-2-fluoro-3-oxopentanoate* (**S2-11a**) Reaction performed using **S1-11a** (500 mg, 2.03 mmol), subsequent purification *via* flash column chromatography (0-10% EtOAc/cyclohexane) gave **S2-11a** as a colourless oil (336 mg, 63%). $\upsilon_{max}$ (oil) 1756 (s), 1731 (s) $cm^{-1}$; $^{1}H$ NMR (400 MHz, $CDCl_3$) $\delta$ 7.30-7.25 (3H, m), 7.21 (2H, d, *J* = 7.4 Hz), 5.85 (1H, ddt, *J* = 16.3, 10.8, 4.2 Hz), 5.33-5.29 (1H, m), 5.28-5.26 (1H, m), 4.69-4.62 (2H, m), 3.43 (2H, d, *J* = 25.9 Hz), 2.60-2.53 (1H, m), 2.36-2.38 (1H, m), 0.95 (3H, t, *J* = 7.2 Hz); $^{13}C$ NMR (176 MHz, $CDCl_3$) $\delta$ 205.3 (C, d, *J* = 28.2 Hz), 165.7 (C, d, *J* = 25.4 Hz), 133.2 (C, s), 130.9 (CH, s), 130.5 (CH, s), 128.5 (CH, s), 127.6 (CH, s), 119.6 ($CH_2$, s), 100.3 (C, d, *J* = 200.4 Hz), 67.0 ($CH_2$, s), 40.2 ($CH_2$, d, *J* = 20.2), 32.1 ($CH_2$, s), 6.86 ($CH_3$, d, *J* = 2.2 Hz); $^{19}F$ NMR (659 MHz, $CDCl_3$) $\delta$ −167.2 (t, *J* = 25.9 Hz); *m/z* [LRMS, ESI] (relative intensity, M) 265 (78, [M+H]+), 247 (100), 227 (98); [HRMS, ESI] Calcd. $(C_{15}H_{17}FO_3+H)^+$ 265.1235, found 265.1230.

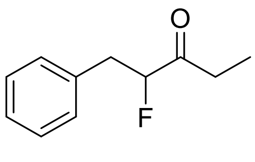

Step 3) *2-Fluoro-1-phenylpentan-3-one* (**11a**) Reaction performed using **S2-11a** (200 mg, 0.756 mmol), subsequent purification *via* flash column chromatography (0-10% EtOAc/cyclohexane) gave **11a** as a colourless oil (109 mg, 80%). $^{1}H$ NMR (600 MHz, $CDCl_3$) $\delta$ 7.33-7.26 (3H, m), 7.22 (2H, d, *J* = 7.3 Hz), 4.98 (1H, ddd, *J* = 49.8, 7.5, 3.8 Hz), 3.20 (1H, ddd, *J* = 28.9, 14.5, 3.8 Hz), 3.06 (1H, ddd, *J* = 26.6, 14.5, 7.5 Hz), 2.58 (1H, dqd, *J* = 19.0, 7.2, 3.4 Hz), 2.36 (1H, dqd, *J* = 19.1, 7.2, 2.6 Hz), 1.00 (3H, t, *J* = 7.2 Hz); $^{13}C$ NMR (150 MHz, $CDCl_3$) $\delta$ 210.6 (C, d, *J* = 24.9 Hz), 135.6 (C, s), 129.6 (CH, s), 128.6 (CH, s), 127.1 (CH, s), 96.0 (CH, d, *J* = 187.2 Hz), 38.4 ($CH_2$, d, *J* = 20.4 Hz), 32.1

($CH_2$, s), 6.6 ($CH_3$, d, $J$ = 2.4 Hz); $^{19}F$ NMR (659 MHz, $CDCl_3$) δ −191.0 to −191.2 (m); *m/z* [LRMS, EI] Calcd. $(C_{11}H_{13}FO)^+$ 180, found 180. Data in agreement with that reported in the literature.[108]

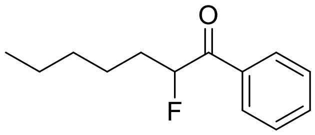


*2-Fluoro-1-phenylheptan-1-one* (**11b**): Following the procedure of Zhang *et al.* as reported for 2-fluoro-1-phenylbutan-1-one.[105] A solution of diisopropylamine (0.47 ml, 3.36 mmol) in THF (5 mL) was cooled to -78 °C, treated with $^n$BuLi (1.91 mL, 3.06 mmol, 1.6 M in hexanes) and the resultant solution stirred for 15 min. The reaction vessel was removed from the cooling bath and allowed to warm to rt for 30 min. After cooling to -78 °C, heptanophenone (0.56 mL, 2.78 mmol) was added dropwise and stirred for 1.5 h, NFSI (1.05 g, 3.34 mmol, solution in 3 mL THF) was added and the reaction allowed to warm to rt and stirred for 16 h. $NH_4Cl_{(aq.\ sat.)}$ (5 mL) was added, followed by HCl (1 M, 5 mL) and the mixture extracted with $CH_2Cl_2$ (3 x 20 mL), dried ($Na_2SO_4$), filtered and conc. *in vacuo.* Purification *via* flash column chromatography (5-20% $CH_2Cl_2$/hexane) gave **11b** as a colourless oil (196 mg, 34%). $^1H$ NMR (700 MHz, $CDCl_3$) δ 7.97 (2H, d, $J$ = 7.6 Hz), 7.61 (1H, t, $J$ = 7.6 Hz), 7.50 (2H, app t, $J$ = 7.6 Hz), 5.62-5.52 (1H, m), 2.00-1.93 (2H, m), 1.57-1.52 (2H, m), 1.38-1.31 (4H, m), 0.90 (3H, t, $J$ = 6.9 Hz); $^{13}C$ NMR (176 MHz, $CDCl_3$) δ 197.1 (C, d, $J$ = 19.5 Hz), 134.5 (C, s), 133.8 (CH, s), 129.0 (CH, d, $J$ = 3.7 Hz), 128.9 (CH, s), 94.0 (CH, d, $J$ = 183.0 Hz), 32.9 ($CH_2$, d, $J$ = 21.3 Hz), 31.5 ($CH_2$, s), 24.6 ($CH_2$, d, $J$ = 3.0 Hz), 22.5 ($CH_2$, s), 14.1 ($CH_3$, s); $^{19}F$ NMR (659 MHz, $CDCl_3$) δ −189.6 to −189.7 (m); *m/z* [HRMS, ESI] Calcd. $(C_{13}H_{17}FO+H)^+$ 209.1336, found 209.1333. Data in agreement with that reported in the literature.[110]

## (*Z*) α-Fluoroenoates (6)

### General Procedure D:[62]

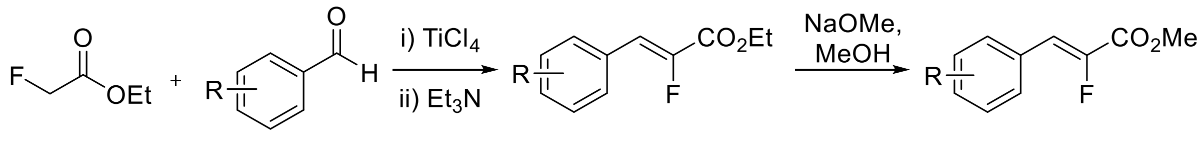


**S3** **6**

i) Using the procedure of Jothib *et al.*[62] A solution of aldehyde (1 eq.) and ethyl fluoroacetate (1.1 eq.) in anhydrous $CH_2Cl_2$ (1.2 mL/100 mg) was treated with $TiCl_4$ (1 M solution in $CH_2Cl_2$, 1.2 eq.) added dropwise over 10 min. The resultant solution was stirred at rt for 1 h. After this time $Et_3N$ (2 eq.) was added dropwise over 10 min, ensuring that the reaction temperature remained below 30 °C (ice bath used). The reaction was stirred for 2 h, diluted with $CH_2Cl_2$ (10 mL), washed 1 M HCl (15 mL), $H_2O$ (15 mL) and brine (15 mL). The organics were dried ($MgSO_4$), filtered and conc. *in vacuo*. Purification was achieved by flash column chromatography to give **S3**.

ii) NaOMe (1.2 eq.) was added to a solution of **S3** (1 eq.) in anhydrous MeOH (5 mL/100 mg). The reaction mixture was stirred for 1 h. Celite™ was then added, the reaction filtered, and the solvent removed *in vacuo*. The resultant residue was taken up in $Et_2O$, filtered through a silica plug and conc. *in vacuo* to afford pure (*Z*)-**6**.

*Methyl (Z)-2-fluoro-3-phenylacrylate* ((*Z*)-**6a**): General Procedure D.

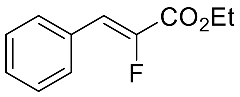


*Ethyl (Z)-2-fluoro-3-phenylacrylate* ((*Z*)-**S3-6a**): $TiCl_4$ mediated aldol condensation of benzaldehyde (500 mg, 4.71 mmol) and subsequent purification *via* flash column chromatography (10% EtOAc/pet. ether) gave (*Z*)-**S3-6a** as a dark orange oil (336 mg, 37%). $^1$H NMR (400 MHz, $CDCl_3$) δ 7.67-7.65 (2H, m), 7.48-7.36 (3H, m), 6.93 (1H, d, *J* = 35.3 Hz), 4.36 (2H, q, *J* = 7.1 Hz), 1.40 (3H, t, *J* = 7.1 Hz); $^{13}$C NMR (100 MHz, $CDCl_3$) δ 161.4 (C, d, *J* = 34.0 Hz), 147.0 (C, d, *J* = 266.1 Hz), 131.1 (C, d, *J* = 4.4 Hz), 130.3 (CH, d, *J* = 8.1 Hz), 129.6 (CH, d, *J* = 2.7 Hz), 128.8 (CH, s), 117.4 (CH, d, *J* = 4.7 Hz), 61.9 ($CH_2$, s), 14.2 ($CH_3$, s); $^{19}$F NMR (377 MHz, $CDCl_3$) δ −125.3 (d, *J* = 35.3 Hz); *m/z* [LRMS, EI] Calcd. $(C_{11}H_{11}FO_2)^+$ 194, found 194. Data in agreement with that reported in the literature.[111]

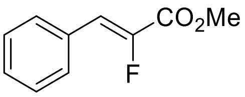


*Methyl (Z)-2-fluoro-3-phenylacrylate* ((*Z*)-**6a**): Transesterification of (*Z*)-**S3-6a** (100 mg, 0.515 mmol) gave (*Z*)-**6a** as a yellow oil (63 mg, 68%). $^1$H NMR (700 MHz, $CDCl_3$) δ 7.67-7.65 (2H, m), 7.43-7.39 (2H, m), 6.95 (1H, d, *J* = 36.0 Hz), 3.91 (3H, s); $^{13}$C NMR (176 MHz, $CDCl_3$) δ 162.0 (C, d, *J* = 31.0 Hz), 147.7 (C, d, *J* = 266.0 Hz), 131.2 (C, d, *J* = 4.4 Hz), 130.4 (CH, d, *J* = 2.6 Hz), 129.9 (CH, d, *J* = 8.1 Hz), 128.7 (CH, s), 117.9 (CH, d, *J* = 4.6 Hz), 52.8 ($CH_3$, s); $^{19}$F NMR

(659 MHz, $CDCl_3$) δ −125.6 (d, *J* = 36.0 Hz); *m/z* [LRMS, EI] Calcd. $(C_{10}H_9FO_2)^+$ 180, found 180. Data in agreement with that reported in the literature.[112]

*Methyl (Z)-2-fluoro-3-(4-trifluoromethylphenyl)acrylate* ((*Z*)-**6b**): General Procedure D.

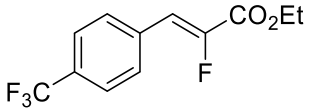

*Ethyl (Z)-2-fluoro-3-(4-trifluoromethylphenyl)acrylate* ((*Z*)-**S3-6b**): $TiCl_4$ mediated aldol condensation of 2-(trifluoromethyl)benzaldehyde (392 µL, 2.87 mmol) and subsequent purification *via* flash column chromatography (2-8% EtOAc/pet. ether) gave (*Z*)-**S3-6b** as a fine white solid (354 mg, 47%). m.p. 50-52 °C (no lit. m.p. reported); $^1$H NMR (700 MHz, $CDCl_3$) δ 7.75 (2H, d, *J* = 8.4 Hz), 7.66 (2H, d, *J* = 8.4 Hz), 6.95 (1H, d, *J* = 34.9 Hz), 4.38 (2H, q, *J* = 7.2 Hz), 1.40 (3H, t, *J* = 7.2 Hz); $^{13}$C NMR (176 MHz, $CDCl_3$) δ 160.8 (C, d, *J* = 38.5 Hz), 148.4 (C, d, *J* = 266.3 Hz), 134.5 (C, d, *J* = 4.6 Hz), 131.0 (C, s), 130.3 (CH, d, *J* = 8.4 Hz), 125.7 (CH, q, *J* = 3.8 Hz), 123.8 (C, q, *J* = 271.6 Hz), 115.8 (CH, d, *J* = 4.2 Hz), 62.2 ($CH_2$, s), 14.2 ($CH_3$, s); $^{19}$F NMR (659 MHz, $CDCl_3$) δ −63.0 (3F, s), −122.0 (1F, d, *J* = 34.9 Hz); *m/z* [HRMS, ESI] Calcd. $(C_{12}H_{10}F_4O_2+H)^+$ 263.0690, found 263.0685. Data in agreement with that reported in the literature.[111]

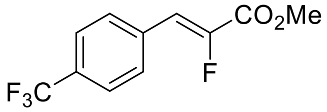

*Methyl (Z)-2-fluoro-3-(4-trifluoromethylphenyl)acrylate* ((*Z*)-**6b**): Transesterification of (*Z*)-**S3-6b** (100 mg, 0.381 mmol) gave (*Z*)-**6b** (52 mg, 55%) as a fine white solid. m.p. 84-86 °C (Lit.[109] 90-92 °C). $^1$H NMR (600 MHz, $CDCl_3$) δ 7.76 (2H, d, *J* = 8.1 Hz), 7.67 (2H, d, *J* = 8.1 Hz), 6.97 (1H, d, *J* = 34.0 Hz), 3.93 (3H, d, *J* = 0.9 Hz); $^{13}$C NMR (176 MHz, $CDCl_3$) δ 161.4 (C, d, *J* = 34.9 Hz), 148.0 (C, d, *J* = 271.0 Hz), 134.4 (C, d, *J* = 3.6 Hz), 131.2 (C, qd, *J* = 32.5, 2.4 Hz), 130.4 (CH, d, *J* = 7.2 Hz), 125.7 (CH, dq, *J* = 7.2, 3.6 Hz), 123.8 (C, q, *J* = 271.0 Hz), 116.1 (CH, d, *J* = 3.6 Hz), 52.9 ($CH_3$, s); $^{19}$F NMR (659 MHz, $CDCl_3$) δ −63.0 (3H, s), −122.4 (1H, d, *J* = 34.0 Hz); *m/z* [LRMS, EI] Calcd. $(C_{11}H_8F_4O_2)^+$ 248, found 248. Data in agreement with that reported in the literature.[114]

*Methyl (Z)-2-fluoro-3-(4-methoxyphenyl)acrylate* ((*Z*)-**6c**): General Procedure D.

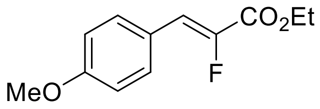

*Ethyl (Z)-2-fluoro-3-(4methoxyphenyl)acrylate* ((*Z*)-**S3-6c**): $TiCl_4$ mediated aldol condensation of 4-anisaldehyde (0.57 mL, 4.71 mmol) and subsequent purification *via* flash column chromatography (20% EtOAc/pet. ether) gave (*Z*)-**S3-6c** as an off white solid (576 mg, 54%). m.p. 39-40 °C (Lit.[111] 43 °C); $^1$H NMR (400 MHz, $CDCl_3$) δ 7.62 (1H, d, *J* = 8.4 Hz), 6.93 (1H, d, *J* = 8.4 Hz), 6.88 (1H, d, *J* = 35.5 Hz), 4.35 (2H, q, *J* = 7.1 Hz), 3.85 (3H, s), 1.39 (3H, t, *J* = 7.1 Hz); $^{13}$C NMR (100 MHz, $CDCl_3$) δ 161.8 (C, d, *J* = 33.9 Hz), 160.7 (C, d, *J* = 3.3 Hz), 147.9 (C, d, *J* = 263.8 Hz), 132.1

(CH, d, $J$ = 8.1 Hz), 123.9 (C, d, $J$ = 4.3 Hz), 117.4 (CH, d, $J$ = 4.9 Hz), 114.4 (CH, s), 61.8 ($CH_2$, s), 55.4 ($CH_2$, s), 14.3 ($CH_3$, s); $^{19}F$ NMR (377 MHz, $CDCl_3$) δ −128.8 (d, $J$ = 35.5 Hz); *m/z* [HRMS, ESI] Calcd. $(C_{12}H_{13}FO_3+H)^+$ 225.0922, found 225.0917. Data in agreement with that reported in the literature.[111]

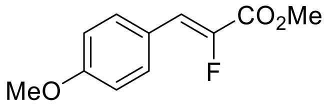


*Methyl (Z)-2-fluoro-3-(4-methoxyphenyl)acrylate* ((*Z*)-**6c**): Transesterification of (*Z*)-**S3-6c** (100 mg, 0.450 mmol) gave (*Z*)-**6c** as a white solid (76 mg, 80%). m.p. 53-54 °C (Lit.[109]57-59 °C); $^1H$ NMR (700 MHz, $CDCl_3$) δ 7.62 (2H, d, $J$ = 8.8 Hz), 6.94 (2H, d, $J$ = 8.8 Hz), 6.90 (1H, d, $J$ = 35.7 Hz), 3.89 (3H, s), 3.85 (3H, s); $^{13}C$ NMR (176 MHz, $CDCl_3$) δ 162.4 (C, d, $J$ = 33.8 Hz), 160.9 (C, d, $J$ = 3.3 Hz), 145.7 (C, d, $J$ = 262.2 Hz), 132.2 (CH, d, $J$ = 8.1 Hz), 123.9 (C, d, $J$ = 4.3 Hz), 117.8 (CH, d, $J$ = 4.7 Hz), 114.4 (CH, s), 55.5 ($CH_3$, s), 52.6 ($CH_3$, s); $^{19}F$ NMR (659 MHz, $CDCl_3$) δ −129.1 (d, $J$ = 35.7 Hz); *m/z* [LRMS, EI] Calcd. $(C_{11}H_{11}FO_3)^+$ 210, found 210. Data in agreement with that reported in the literature.[116]

## (*E*) α-Fluoroenoates (6)

### General Procedure E:[73]

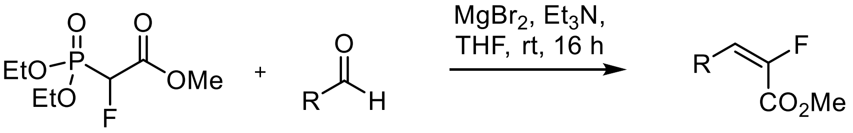


**6a-e**

Synthesis of fluoroalkenes **6a-e** utilising conditions analogous to those of Andersson whereby the ethyl ester is used.[34,73] N/B - The E alkene was predominantly formed when $MgBr_2$ was utilised in the HWE reaction. This is the opposite selectivity to that seen by Andersson *et al.*

A solution of methyl diethylmethyl-1-fluorophosphonoacetate (1 eq.) in anhydrous THF (5 mL/mmol) was treated with $Et_3N$ (2 eq.) followed by $MgBr_2$ (1 eq.). Aldehyde (1 eq.) was added and the solution stirred at rt for 16 h. After this time the reaction was diluted with $Et_2O$ (5 mL/mmol), filtered, solid washed with $Et_2O$ (5 mL/mmol) and the organics combined. Washed $NH_4Cl_{(sat.\ aq.)}$ (10 mL/mmol), brine (10 mL/mmol), dried ($Na_2SO_4$), filtered and conc. *in vacuo*. Purification was achieved by flash column chromatography, where stated, to give **6** as a mixture of *E*/*Z* isomers as stated (predominantly *E* isomer).

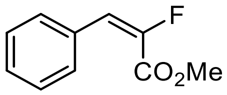


*Methyl (E/Z)-2-fluoro-3-phenylacrylate* (**6a**): General Procedure E. Reaction performed using benzaldehyde (106 µL, 1.04 mmol), no purification was necessary, giving **6a** as colourless oil (161 mg, 86%, 8:1 *E*:*Z*).

(*E*)-**6a**: $^{1}$H NMR (700 MHz, $CDCl_3$) δ 7.49-7.47 (2H, m), 7.39-7.33 (2H, m), 6.92 (1H, d, *J* = 22.5 Hz), 3.81 (3H, s); $^{13}$C NMR (176 MHz, $CDCl_3$) δ 161.0 (C, d, *J* = 35.5 Hz), 146.8 (C, d, *J* = 254.0 Hz), 130.9 (d, *J* = 9.3 Hz), 129.8 (CH, d, *J* = 11.1 Hz), 129.0 (CH, s), 128.2 (CH, d, *J* = 4.0 Hz), 122.0 (CH, d, *J* = 25.4 Hz), 52.4 ($CH_3$, s); $^{19}$F NMR (659 MHz, $CDCl_3$) δ −117.5 (d, *J* = 22.5 Hz); *m/z* [LRMS, ESI] Calcd. $(C_{10}H_9FO_2)^+$ 180, found 180. (*E*)-Data in agreement with that reported in the literature (no $^{13}$C NMR Spectra reported).[74]

(*Z*)-**6a**: NMR data as reported above.

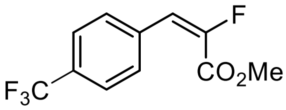


*Methyl (E)-2-fluoro-3-(4-trifluoromethylphenyl)acrylate* (**6b**): General Procedure E. Reaction performed using 4-(trifluoromethyl)benzaldehyde (120 µL, 0.877 mmol), and subsequent purification *via* flash column chromatography (10 % EtOAc/cyclohexane) gave **6b** as pale yellow oil (48 mg, 22%, as 22 mg pure *E*; 17 mg 19:1 *E*:*Z* and 9 mg 1:15 *E*:*Z*).

(*E*)-**6b**: $\upsilon_{max}$ (oil) 2955 (w), 1740 (s) cm$^{-1}$; $^{1}$H NMR (700 MHz, $CDCl_3$) δ 7.63 (2H, d, *J* = 8.3 Hz), 7.57 (2H, d, *J* = 8.3 Hz), 6.92 (1H, d, *J* = 21.2 Hz), 3.81 (3H, s); $^{13}$C NMR (176 MHz, $CDCl_3$) δ 160.6 (C, d, *J* = 35.3 Hz), 147.7 (C, d, *J* = 258.9 Hz), 134.7 (C, d, *J* = 9.9 Hz), 130.7 (C, q, *J* = 32.5 Hz), 130.0 (CH, d, *J* = 2.8 Hz), 125.0 (CH, q, *J* = 3.7 Hz), 123.9 (C, q, *J* = 272.4 Hz), 120.3 (CH, d, *J* = 26.3 Hz), 52.6 ($CH_3$, s); $^{19}$F NMR (659 MHz, $CDCl_3$) δ −62.8 (3F, s), −114.9 (1F, d, *J* = 21.2 Hz); *m/z* [LRMS, ESI] (relative intensity, M) 249 (92, [M+H]+), 229 (35, [M-F]+), 218 (11, [M-OMe+H]+), 217 (100, [M-OMe]+), 197 (19, [M-OMe-F-H]+); [HRMS, ESI] Calcd. $(C_{11}H_8F_4O_2H)^+$ 249.0533, found 249.0524.

(*Z*)-**6b**: NMR data as reported above.

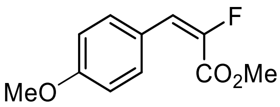


*Methyl (E/Z)-2-fluoro-3-(4-methoxyphenyl)acrylate* (**6c**): General Procedure E. Reaction performed using 4-anisaldehyde (106 µL, 0.877 mmol) and subsequent purification *via* flash column chromatography ($CH_2Cl_2$) gave **6c** as colourless oil (109 mg, 54%, 5:1 *E*:*Z*).

(*E*)-**6c**: $^{1}$H NMR (700 MHz, $CDCl_3$) δ 7.55-7.53 (2H, m), 6.91-6.89 (2H, m), 6.87 (1H, d, *J* = 24.0 Hz), 3.84 (3H, s), 3.86 (3H, s); $^{13}$C NMR (176 MHz, $CDCl_3$) δ 161.4 (C, d, *J* = 35.2 Hz), 160.4 (C, d, *J* = 1.0 Hz), 145.6 (C, d, *J* = 250.8 Hz), 132.0 (CH, d, *J* = 2.8 Hz), 123.0 (C, d, *J* = 9.2 Hz), 122.5 (CH, d, *J* = 26.7 Hz), 113.8 (CH, s), 55.4 ($CH_3$, s), 52.3 ($CH_3$, s); $^{19}$F NMR (659 MHz, $CDCl_3$) δ −119.7 (d, *J* = 24.0 Hz). *m/z* [LRMS, EI] Calcd. $(C_{11}H_{11}FO_3)^+$ 210, found 210.

(*Z*)-**6c**: NMR data as reported above.

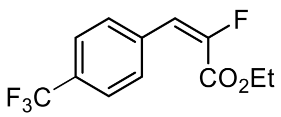

*Ethyl (E)-2-fluoro-3-(4-trifluoromethylphenyl)acrylate* (**6d**): General Procedure E. Reaction performed using 4-(trifluoromethyl)benzaldehyde (250 mg, 1.44 mmol) and diethylphosphoryl-2-fluoroacetate (291 µL, 1.58 mmol), and subsequent purification *via* flash column chromatography (1-4% EtOAc/cyclohexane) gave **6d** as a colourless oil (78 mg, 21% pure *E*).

(*E*)-**6d**: $^1$H NMR (700 MHz, $CDCl_3$) δ 7.62 (2H, d, *J* = 8.3 Hz), 7.55 (2H, d, *J* = 8.3 Hz), 6.93 (1H, d, *J* = 21.0 Hz), 4.26 (2H, q, *J* = 7.1 Hz), 1.25 (3H, t, *J* = 7.1 Hz); $^{13}$C NMR (176 MHz, $CDCl_3$) δ 160.0 (C, d, *J* = 36.1 Hz), 148.0 (C, d, *J* = 257.8 Hz), 134.8 (C, d, *J* = 9.6 Hz), 130.4 (C, q, *J* = 32.5 Hz), 129.8 (CH, d, *J* = 2.4 Hz), 124.9 (CH, q, *J* = 3.6 Hz), 123.9 (C, q, *J* = 273.5 Hz), 119.9 (CH, d, *J* = 26.5 Hz), 51.9 ($CH_2$, s), 13.8 ($CH_3$, s); $^{19}$F NMR (659 MHz, $CDCl_3$) δ −62.8 (3F, s), −114.6 (1F, d, *J* = 21.0 Hz); *m/z* [LRMS, EI] Calcd. $(C_{12}H_{10}F_4O_2)^+$ 262, found 262. (*E*)-Data in agreement with that reported in the literature.[117]

(*Z*)-NMR data as reported above.

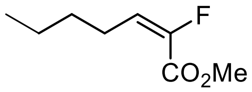

*Methyl (E/Z)-2-fluorohept-2-enoate* (**6e**): General Procedure E. Reaction performed using valeraldehyde (93 µL, 0.88 mmol), no purification needed, gave **6e** as a yellow oil (82 mg, 54%, 13:1 *E*:*Z*).

(*E*)-**6e**: $^1$H NMR (700 MHz, $CDCl_3$) δ 5.94 (1H, dt, *J* = 21.8, 7.9 Hz), 3.84 (3H, s), 2.52 (2H, dq, *J* = 7.9, 1.5 Hz), 1.46-1.38 (4H, m), 0.94 (3H, t, *J* = 7.6 Hz); $^{13}$C NMR (176 MHz, $CDCl_3$) δ 161.6 (C, d, *J* = 35.7 Hz), 147.0 (C, d, *J* = 249.0 Hz), 124.3 (CH, d, *J* = 17.3 Hz), 52.1 ($CH_3$, s), 31.4 ($CH_2$, d, *J* = 2.0 Hz), 25.2 ($CH_2$, d, *J* = 4.9 Hz), 22.3 ($CH_2$, s), 13.9 ($CH_3$, s); $^{19}$F NMR (659 MHz, $CDCl_3$) δ −123.2 (d, *J* = 21.7 Hz). *m/z* [LRMS, ES+] Calcd. $(C_8H_{13}FO_2)^+$ 160.09, found 160.11 (Does not fly by ESI).

(*Z*)-**6e**: $^1$H NMR (700 MHz, $CDCl_3$) δ 6.13 (1H, dt, *J* = 33.3, 7.7 Hz), 3.82 (3H, s), 2.26 (2H, qd, *J* = 7.7, 2.1 Hz), 1.46-1.38 (4H, m), 0.94 (3H, t, *J* = 7.3 Hz); $^{19}$F NMR (659 MHz, $CDCl_3$) δ −131.3 (d, *J* = 33.3 Hz). $^{13}$C NMR too weak to assign. (*Z*)-Data in agreement with that reported in the literature.[118]

## α-Fluoroesters (Reference Standards, 12)

### General Procedure F:[63]

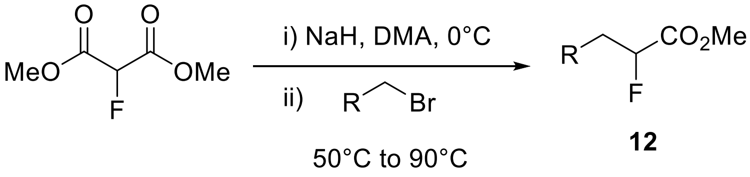


**12**

Synthesis of reference standards **12a-e** applying conditions developed by Zhou to the corresponding alkyl or aryl halide.[63]

A solution of dimethyl-2-fluoromalonate (1.2 eq.) in anhydrous DMA (0.8 mL/mmol) was cooled to 0 °C and treated with NaH (60% in mineral oil, 1.2 eq.) added portion-wise (effervescence). The resultant solution was stirred for 10 min and after this time the desired aryl bromide or alkyl bromide (1 eq.) was added. The reaction was heated to 50 °C for 2 h, until no more bromide remained and then heated to 90 °C for 16 h. After this time the reaction was cooled to rt, quenched $NH_4Cl_{(sat.\ aq.)}$ (0.8 mL/mmol) and extracted with EtOAc (3 x 8 mL/mmol). The organics were dried ($Na_2SO_4$), filtered and conc. *in vacuo*. Purification was achieved by flash column chromatography to give **12**.

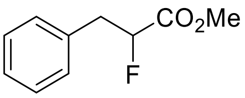


*Methyl 2-fluoro-3-phenylpropanoate* (**12a**): General Procedure F. Substitution/Krapcho reaction using benzyl bromide (59 µL, 0.50 mmol) and subsequent purification *via* flash column chromatography (0-40% $Et_2O$/pentane) gave **12a** as a colourless oil (40 mg, 37%). $^1$H NMR (700 MHz, $CDCl_3$) δ 7.33 (2H, d, *J* = 7.6 Hz), 7.29-7.24 (3H, m), 5.12 (1H, ddd, *J* = 48.7, 7.9, 3.9 Hz), 3.78 (3H, s), 3.25 (1H, ddd, *J* = 28.9, 14.7, 3.9 Hz), 3.18 (1H, ddd, *J* = 31.5, 14.7, 7.9 Hz); $^{13}$C NMR (176 MHz, $CDCl_3$) δ 169.8 (C, d, *J* = 23.7 Hz), 135.2 (C, d, *J* = 1.6 Hz), 129.5 (CH, s), 128.7 (CH, s), 127.4 (CH, s), 89.5 (CH, d, *J* = 186.4 Hz), 52.5 ($CH_3$, s), 38.8 ($CH_2$, d, *J* = 20.9 Hz); $^{19}$F NMR (659 MHz, $CDCl_3$) δ −190.1 (ddd, *J* = 48.8, 28.9, 23.7 Hz); *m/z* [LRMS, EI] Calcd. $(C_{10}H_{11}FO_2)^+$ 182, found 182. Data in agreement with that reported in the literature.[119]

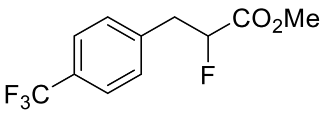


*Methyl 2-fluoro-3-(4-trifluoromethylphenyl)propanoate* (**12b**): General Procedure F. Substitution/Krapcho reaction using 4-bromomethyltrifluoromethylbenzene (77 µL, 0.50 mmol) gave a 8:5 ratio of product and non-decarboxylated intermediate, subsequent purification *via* flash column chromatography (0-40% $Et_2O$/pentane) gave **12b** as a colourless oil (42 mg, 34%). $\upsilon_{max}$ (oil) 2956 (w), 2929 (w), 1762 (m), 1742 (m) cm$^{-1}$; $^1$H NMR (700 MHz, $CDCl_3$) δ 7.59 (2H, d, *J* = 8.1 Hz), 7.38 (2H, d, *J* = 8.1 Hz), 5.13 (1H, ddd, *J* = 48.5, 7.8, 3.9 Hz), 3.79 (3H, s), 3.35-3.20 (2H, m); $^{13}$C NMR (176 MHz, $CDCl_3$) δ 169.3 (C, d, *J* = 23.5 Hz), 139.3 (C, s), 129.9 (CH, s), 129.8 (C, q, *J* =

97.4 Hz), 125.7 (CH, q, *J* = 3.7 Hz), 124.2 (C, q, *J* = 272.0 Hz), 88.9 (CH, d, *J* = 188.6 Hz), 52.6 ($CH_3$, s), 38.5 ($CH_2$, d, *J* = 21.0 Hz); $^{19}F$ NMR (659 MHz, $CDCl_3$) δ −62.6 (3F, s), −190.5 (1F, ddd, *J* = 52.1, 27.9, 24.3 Hz); *m/z* [LRMS, ESI] (relative intensity, M) 251 (100, [M+H]+), 231 (31, [M-F]+), 191 (44, [M-$CO_2$Me]+); [HRMS, ESI] Calcd. $(C_{11}H_{10}F_4O_2+H)^+$ 251.0690, found 251.0687.

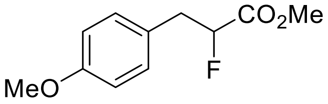


*Methyl 2-fluoro-3-(4-methoxyphenyl)propanoate* (**12c**): General Procedure F. Substitution/Krapcho reaction using 4-methoxybenzyl bromide (70 µL, 0.50 mmol) and subsequent purification *via* flash column chromatography (0-20% $Et_2O$/pentane) gave **12c** as a colourless oil (48 mg, 45%). $\upsilon_{max}$ (oil) 2952 (w), 2930 (w), 2835 (w), 1760 (s), 1741 (m), 1610 (w), 1512 (s) cm$^{-1}$; $^1H$ NMR (500 MHz, $CDCl_3$) δ 7.17-7.16 (2H, m), 6.88-6.85 (2H, m), 5.07 (1H, ddd, *J* = 48.9, 7.6, 4.1 Hz), 3.80 (3H, s), 3.77 (3H, s), 3.23-3.06 (2H, m); $^{13}C$ NMR (125 MHz, $CDCl_3$) δ 169.9 (C, d, *J* = 23.7 Hz), 158.9 (C, s), 130.6 (CH, s), 127.2 (C, d, *J* = 1.7 Hz), 114.1 (CH, s), 89.6 (CH, d, *J* = 187.1 Hz), 55.4 ($CH_3$, s), 52.5 ($CH_3$, s), 38.0 ($CH_2$, d, *J* = 21.0 Hz); $^{19}F$ NMR (377 MHz, $CDCl_3$) δ −190.4 (ddd, *J* = 48.7, 28.0, 24.7 Hz); *m/z* [LRMS, ESI] (relative intensity, M) 213 (31, [M+H]+), 193 (32, [M-F]+), 181 (100, [M-OMe]+), 153 (50, [M-$CO_2$Me]+); [HRMS, ESI] Calcd. $(C_{11}H_{13}FO_3+H)^+$ 213.0922, found 213.0921.

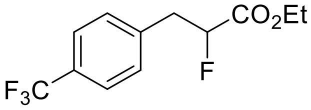


*Ethyl 2-fluoro-3-(4-trifluoromethylphenyl)propanoate* (**12d**): General Procedure F. Substitution/Krapcho reaction using 4-bromomethyltrifluoromethylbenzene (287 mg, 1.20 mmol) and diethyl-2-fluoromalonate (189 µL, 1.2 mmol) gave a 2:3 ratio of product and non-decarboxylated intermediate, subsequent purification *via* flash column chromatography (0-5% EtOAc/cyclohexane) gave **12d** as a colourless oil (82 mg, 26%). $\upsilon_{max}$ (oil) 2986 (w), 1755 (m) cm$^{-1}$; $^1H$ NMR (400 MHz, $CDCl_3$) δ 7.59 (2H, d, *J* = 8.0 Hz), 7.38 (2H, d, *J* = 8.0 Hz), 5.13 (1H, ddd, *J* = 48.7, 7.3, 4.0 Hz), 4.24 (2H, app q, *J* = 7.3 Hz), 3.37-3.17 (2H, m), 1.27 (3H, t, *J* = 7.1 Hz); $^{13}C$ NMR (176 MHz, $CDCl_3$) δ 168.9 (C, d, *J* = 23.5 Hz), 139.3 (C, s), 130.0 (CH, s), 129.7 (C, q, *J* = 32.5 Hz), 125.6 (CH, q, *J* = 3.7 Hz), 124.2 (C, q, *J* = 272.0 Hz), 88.8 (CH, d, *J* = 188.5 Hz), 61.9 ($CH_2$, s), 38.5 ($CH_2$, d, *J* = 20.9 Hz), 14.2 ($CH_3$, s); $^{19}F$ NMR (659 MHz, $CDCl_3$) δ −62.6 (3F, s), −190.3 (1F, ddd, *J* = 52.1, 27.6, 24.6 Hz); *m/z* [LRMS, ESI] (relative intensity, M) 265 (42, [M+H]+), 199 (100); [HRMS, ESI] Calcd. $(C_{12}H_{12}F_4O_2+H)^+$ 265.0846, found 265.0843.

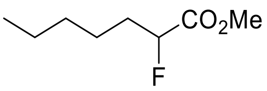


*Methyl 2-fluoroheptanoate* (**12e**): General Procedure F. Substitution/Krapcho reaction using bromopentane (124 µL, 1.0 mmol) and subsequent purification *via* flash column chromatography (15% $Et_2O$/pentane) gave **12e** as a colourless oil (81 mg, 50%). $\upsilon_{max}$ (oil) 2956 (w), 2931 (w), 2862 (w), 1765 (s), 1745 (m) cm$^{-1}$; $^1H$ NMR (700 MHz,

$CDCl_3$) δ 4.92 (1H, ddd, *J* = 49.1, 7.1, 4.6 Hz), 3.81 (3H, s), 1.92-1.85 (2H, m), 1.52-1.43 (2H, m), 1.36-1.29 (4H, m), 0.92-0.89 (3H, m); $^{13}C$ NMR (176 MHz, $CDCl_3$) δ 170.7 (C, d, *J* = 23.8 Hz), 89.2 (CH, d, *J* = 184.0 Hz), 52.4 ($CH_3$, s), 32.5 ($CH_2$, d, *J* = 21.0 Hz), 31.3 ($CH_2$, s), 24.2 ($CH_2$, d, *J* = 2.9 Hz), 22.5 ($CH_2$, s), 14.1 ($CH_3$, s); $^{19}F$ NMR (377 MHz, $CDCl_3$) −192.1 (ddd, *J* = 49.6, 27.0, 24.4 Hz); *m/z* [LRMS, EI] Calcd. $(C_8H_{15}FO_2)^+$ 162, found 162 (compound not detected by ESI).

## β-Fluoroenones

β-Fluoro alkenes and the corresponding reference compounds were synthesised according to the published literature procedures:

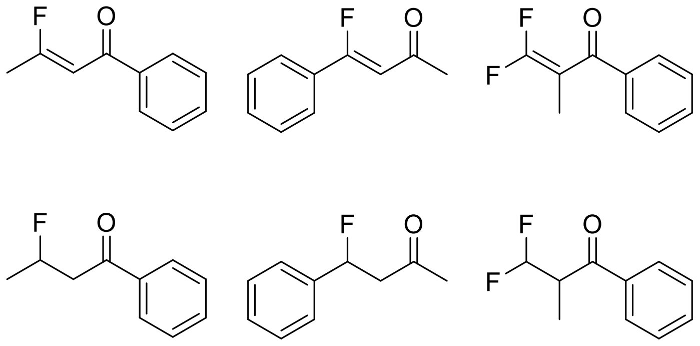


(*Z*)-3-Fluoro-1-phenylbut-2-en-1-one[120]

4-Fluoro-4-phenylbutan-2-one[121]

(*Z*)-4-Fluoro-4-phenylbut-3-en-2-one[122]

4-Fluoro-4-phenylbutan-2-one[123]

3,3-Difluoro-2-methyl-1-phenylprop-2-en-1-one[124]

3,3-Difluoro-2-methyl-1-phenylpropan-1-one[125]

# Biological details

Three ene-reductases (EREDs) were used in this study, NCR, and enzyme from plasmids pQR1907 and pQR1445 (which are referred to by their UCL plasmid number), together with co-expressed glucose-6-phosphate dehydrogenase (G6PDH) pQR1181 for $NAD^+$ recycling. Plasmid constructions were described in our previous publication.[45] The amino acid and DNA sequences were listed below:

**>NCR**
```
MPSLFDPIRFGAFTAKNRIWMAPLTRGRATRDHVPTEIMAEYYAQRASAGLIISEATGISQEGLGWPYAPGIWSDAQVEAWLP
ITQAVHDAGGLIFAQLWHMGRMVPSNVSGMQPVAPSASQAPGLGHTYDGKKPYDVARALRLDEIPRLLDDYEKAARHALKAGF
DGVQIHAANGYLIDEFIRDSTNHRHDEYGGAVENRIRLLKDVTERVIATIGKERTAVRLSPNGEIQGTVDSHPEQVFIPAAKM
LSDLDIAFLGMREGAVDGTFGKTDQPKLSPEIRKVFKPPLVLNQDYTFETAQAALDSGVADAISFGRPFIGNPDLPRRFFEKA
PLTKDVIETWYTQTPKGYTDYPLLGDHHHHHH
```

**>pQR1445**
```
MSNLFTPLQVGAWQLPNRIIMAPLTRCRASEGRVPNALMAEYYAQRASAGLIISEATSVTPMGVGYPNTPGIWSDAQVDGWKL
ITDAVHQAGGRIVLQLWHVGRISDPVYLDGQLPVAPSAIAPQGHVSLVRPTKAFETPRALDTAELVDVVAAYRLGAENAKKAG
FDGVEIHGANGYLLDQFLQSSTNQRTDQYGGSLENRARLLLEVVDACIEVWGADRVGVHLAPRGDAHDMGDANPAETFGYVAE
QLGARQVAFICTREYLADDSLAGLIKAKFGGVYIANEKYDQAQADAAIASGAADAVAFGVKFIANPDLPARFAQGAVLNAPDP
STFYGAGSKGYTDYLTLHHHHHH
```

**>pQR1907**
```
MSGKLFTPFSSGSFTFPNRVIMAPLTRMRASQPGDIPNELMQTYYVQRASAGLIIAEATQISPQGKGYMDTPGIYSAEQVQGW
RKITQAVHEAGGHIALQLWHVGRVSHHSLQPDQQLPVSASAIPYQNRTTVRGEDGKPTRVDCDTPRALELSEIPGVIEDYRRA
TVNSREAGFDMVEVHAAHGYLLHQFQSAESNKREDAYGGSLENRARLTLEALDAVIGAWDAKHVGIRISPLGTFNGLDDKDGL
EMALYLTREFTKRGIAYLHLSEPDWAGGPAHGDEFRQALRDAFPGTIIGAGNYTVEKSEMLLAKGFIDAAAFGRPFIANPDLP
VRLQKGAELNNVVAATLYGGGAEGYTDYPALAHHHHHH
```

**>pQR1181**
```
MGSSHHHHHHSQDPSEGPVKFEKNTVISVFGASGDLAKKKTFPALFGLFREGYLDPSTKIFGYARSKLSMEEDLKSRVLPHLK
KPHGEADDSKVEQFFKMVSYISGNYDTDEGFDELRTQIEKFEKSANVDVPHRLFYLALPPSVFLTVAKQIKSRVYAENGITRV
IVEKPFGHDLASARELQKNLGPLFKEEELYRIDHYLGKELVKNLLVLRFGNQFLNASWNRDNIQSVQISFKERFGTEGRGGYF
DSIGIIRDVMQNHLLQIMTLLTMERPVSFDPESIRDEKVKVLKAVAPIDTDDVLLGQYGKSEDGSKPAYVDDDTVDKDSKCVT
FAAMTFNIENERWEGVPIMMRAGKALNESKVEIRLQYKAVASGVFKDIPNNELVIRVQPDAAVYLKFNAKTPGLSNATQVTDL
NLTYASRYQDFWIPEAYEVLIRDALLGDHSNFVRDDELDISWGIFTPLLKHIERPDGPTPEIYPYGSRGPKGLKEYMQKHKYV
MPEKHPYAWPVTKPEDTKDN
```

**>NCR**
```
ATGCCATCTTTGTTCGATCCAATTCGTTTCGGTGCCTTTACCGCAAAGAACCGTATTTGGATGGCGCCTCTCACCCGTGGTCG
CGCCACGCGCGATCACGTCCCGACCGAGATCATGGCAGAGTACTATGCGCAGCGTGCCAGCGCAGGCCTGATCATTAGCGAAG
CGACCGGTATTAGCCAAGAGGGTCTGGGTTGGCCGTATGCTCCGGGTATCTGGAGCGACGCCCAGGTAGAGGCGTGGCTGCCG
ATCACGCAAGCTGTGCATGACGCGGGTGGCCTGATCTTCGCGCAGCTGTGGCACATGGGTCGCATGGTCCCTTCTAATGTTAG
CGGTATGCAACCGGTGGCGCCGAGCGCATCCCAGGCACCGGGCCTGGGTCACACTTATGACGGCAAAAAGCCGTACGATGTTG
CACGCGCGCTGCGTCTGGATGAAATTCCGCGCTTGCTGGATGACTACGAGAAAGCTGCGCGTCATGCGCTGAAGGCTGGCTTT
GATGGTGTTCAGATCCACGCTGCGAACGGTTACCTGATTGACGAATTCATTCGTGACAGCACCAATCACCGTCATGACGAGTA
TGGTGGCGCAGTTGAGAATCGCATCCGCTTGCTGAAAGATGTGACCGAGCGTGTGATTGCGACGATTGGCAAGGAACGTACCG
CGGTCCGCCTGAGCCCGAACGGTGAGATCCAGGGTACCGTGGATTCGCACCCGGAACAAGTGTTTATTCCAGCCGCAAAAATG
CTGAGCGATTTAGACATCGCGTTCCTGGGTATGCGTGAGGGTGCCGTTGATGGTACGTTTGGCAAGACTGACCAGCCGAAGCT
GAGCCCGGAAATCCGTAAGGTCTTTAAACCGCCGCTGGTCCTGAACCAAGATTACACGTTTGAAACCGCGCAAGCCGCGTTGG
ACAGCGGCGTTGCAGACGCGATCTCCTTCGGCCGTCCGTTCATTGGTAATCCGGATCTGCCGCGTCGTTTCTTTGAGAAAGCA
CCGCTGACCAAAGACGTTATCGAAACCTGGTATACGCAGACGCCGAAAGGTTACACTGACTACCCGCTTCTGGGCGACCACCA
CCACCACCACCACTGA
```

**>pQR1445**
```
ATGTCCAATTTATTCACCCCCCTACAGGTCGGCGCGTGGCAGTTGCCCAATCGGATCATCATGGCACCCCTCACCCGTTGCCG
TGCCAGCGAAGGCCGCGTGCCCAATGCGCTGATGGCAGAATACTACGCGCAGCGTGCCAGTGCCGGTTTGATCATCAGTGAAG
CCACCTCAGTTACACCAATGGGCGTGGGATATCCCAACACACCGGGTATTTGGTCGGATGCGCAAGTGGACGGCTGGAAGCTG
ATCACCGATGCGGTGCATCAAGCCGGTGGGCGGATTGTGCTGCAACTGTGGCATGTGGGGCGGATTTCTGATCCGGTGTATTT
```

GGATGGTCAATTGCCGGTTGCCCCTAGCGCGATTGCCCCACAAGGCCATGTCAGTTTGGTACGCCCGACCAAAGCGTTTGAAA
CCCCACGTGCACTCGACACCGCAGAGCTAGTCGACGTGGTCGCTGCCTATCGTTTGGGTGCAGAGAATGCGAAAAAGGCTGGT
TTTGATGGCGTTGAGATTCATGGAGCCAATGGCTATTTGCTCGATCAATTCTTGCAAAGCTCAACCAATCAACGCACCGATCA
ATATGGCGGCAGCTTAGAGAACCGTGCACGCTTGCTGCTTGAAGTGGTCGATGCTTGTATTGAGGTATGGGGTGCAGATCGGG
TGGGGGTGCATCTGGCGCCGCGTGGCGATGCGCATGATATGGGTGATGCCAACCCTGCCGAAACCTTTGGCTATGTGGCGGAG
CAATTGGGTGCGCGCCAAGTGGCGTTTATTTGTACCCGTGAATATCTGGCCGACGACAGCCTTGCCGGTTTGATCAAAGCCAA
ATTTGGCGGCGTGTATATCGCCAATGAAAAATACGACCAAGCCCAAGCCGATGCAGCCATTGCCAGCGGCGCTGCCGATGCGG
TGGCGTTTGGGGTGAAGTTTATTGCCAACCCCGATTTGCCTGCGCGTTTTGCTCAAGGTGCAGTGCTGAATGCACCTGATCCG
AGCACCTTTTATGGGGCAGGCAGTAAAGGCTATACCGACTATCTGACCTTGCACCACCACCACCACCACTGA

**>pQR1907**
ATGTCCGGCAAGTTGTTCACCCCGTTCAGCTCGGGTTCCTTCACCTTCCCCAACCGCGTTATCATGGCGCCGCTGACGCGTAT
GCGCGCTTCGCAGCCGGGTGACATTCCCAACGAGCTGATGCAGACCTATTACGTGCAGCGCGCCAGCGCCGGCCTCATCATCG
CCGAGGCCACGCAGATCTCCCCGCAGGGCAAGGGCTATATGGACACTCCGGGGATTTATTCCGCGGAGCAGGTGCAGGGCTGG
CGCAAGATCACCCAGGCCGTGCATGAGGCCGGTGGCCATATCGCCCTGCAGCTCTGGCATGTGGGTCGTGTTTCGCATCACAG
CCTGCAGCCCGACCAGCAACTGCCGGTGTCCGCTTCTGCCATTCCCTACCAGAACCGCACCACGGTCCGTGGTGAAGACGGCA
AGCCCACGCGCGTGGATTGCGATACCCCACGTGCGCTGGAACTGTCCGAAATCCCCGGTGTGATCGAAGACTACCGCCGCGCC
ACCGTGAATTCGCGCGAAGCCGGTTTCGACATGGTGGAAGTGCATGCCGCGCATGGCTATCTGCTGCACCAGTTCCAGTCCGC
CGAAAGCAACAAGCGTGAAGACGCCTATGGTGGTTCGCTGGAAAACCGTGCCCGCCTGACGCTGGAAGCCCTGGATGCCGTGA
TCGGTGCCTGGGATGCCAAGCATGTAGGTATCCGCATTTCCCCGCTGGGCACCTTCAACGGCCTGGACGACAAGGACGGCCTG
GAAATGGCGCTGTATCTCACGCGTGAATTCACCAAGCGCGGTATCGCCTACCTGCATCTGTCCGAGCCGGACTGGGCCGGCGG
TCCGGCGCATGGCGACGAATTCCGCCAGGCCCTGCGCGACGCTTTCCCGGGCACCATCATCGGTGCCGGCAACTACACGGTGG
AAAAATCGGAGATGCTGCTGGCCAAGGGCTTTATCGATGCCGCCGCGTTTGGTCGTCCCTTTATTGCCAATCCGGACCTGCCG
GTGCGTCTGCAGAAGGGCGCTGAGTTGAACAATGTGGTGGCGGCTACGCTGTATGGCGGTGGCGCCGAAGGCTATACGGATTA
TCCGGCGCTGGCCCACCACCACCACCACCACTGA

**>pQR1181**
ATGGGCAGCAGCCATCACCATCATCACCACAGCCAGGATCCGAGTGAAGGCCCCGTCAAATTCGAAAAAAATACCGTCATATC
TGTCTTTGGTGCGTCAGGTGATCTGGCAAAGAAGAAGACTTTTCCCGCCTTATTTGGGCTTTTCAGAGAAGGTTACCTTGATC
CATCTACCAAGATCTTCGGTTATGCCCGGTCCAAATTGTCCATGGAGGAGGACCTGAAGTCCCGTGTCCTACCCCACTTGAAA
AAACCTCACGGTGAAGCCGATGACTCTAAGGTCGAACAGTTCTTCAAGATGGTCAGCTACATTTCGGGAAATTACGACACAGA
TGAAGGCTTCGACGAATTAAGAACGCAGATCGAGAAATTCGAGAAAAGTGCCAACGTCGATGTCCCACACCGTCTCTTCTATC
TGGCCTTGCCGCCAAGCGTTTTTTTGACGGTGGCCAAGCAGATCAAGAGTCGTGTGTACGCAGAGAATGGCATCACCCGTGTA
ATCGTAGAGAAACCTTTCGGCCACGACCTGGCCTCTGCCAGGGAGCTGCAAAAAAACCTGGGGCCCCTCTTTAAAGAAGAAGA
GTTGTACAGAATTGACCATTACTTGGGTAAAGAGTTGGTCAAGAATCTTTTAGTCTTGAGGTTCGGTAACCAGTTTTTGAATG
CCTCGTGGAATAGAGACAACATTCAAAGCGTTCAGATTTCGTTTAAAGAGAGGTTCGGCACCGAAGGCCGTGGCGGCTATTTC
GACTCTATAGGCATAATCAGAGACGTGATGCAGAACCATCTGTTACAAATCATGACTCTCTTGACTATGGAAAGACCGGTGTC
TTTTGACCCGGAATCTATTCGTGACGAAAAGGTTAAGGTTCTAAAGGCCGTGGCCCCCATCGACACGGACGACGTCCTCTTGG
GCCAGTACGGTAAATCTGAGGACGGGTCTAAGCCCGCCTACGTGGATGATGACACTGTAGACAAGGACTCTAAATGTGTCACT
TTTGCAGCAATGACTTTCAACATCGAAAACGAGCGTTGGGAGGGCGTCCCCATCATGATGCGTGCCGGTAAGGCTTTGAATGA
GTCCAAGGTGGAGATCAGACTGCAGTACAAAGCGGTCGCATCGGGTGTCTTCAAAGACATTCCAAATAACGAACTGGTCATCA
GAGTGCAGCCCGATGCCGCTGTGTACCTAAAGTTTAATGCTAAGACCCCTGGTCTGTCAAATGCTACCCAAGTCACAGATCTG
AATCTAACTTACGCAAGCAGGTACCAAGACTTTTGGATTCCAGAGGCTTACGAGGTGTTGATAAGAGACGCCCTACTGGGTGA
CCATTCCAACTTTGTCAGAGATGACGAATTGGATATCAGTTGGGGCATATTCACCCCATTACTGAAGCACATAGAGCGTCCGG
ACGGTCCAACACCGGAAATTTACCCCTACGGATCAAGAGGTCCAAAGGGATTGAAGGAATATATGCAAAAACACAAGTATGTT
ATGCCCGAAAAGCACCCTTACGCTTGGCCCGTGACTAAGCCAGAAGATACGAAGGATAATTAG

## Enzyme expression

All ERED plasmids were co-transformed with pQR1181 into *E. coli* BL21 (DE3) and grown overnight at 37 °C in LB broth containing kanamycin (50 µg/mL) and chloramphenicol (34 µg/mL). The preculture were then used to inoculate fresh TB media (1% v/v). Cells were grown at 37 °C, 250 rpm to an $OD_{600}$ of 0.6 and IPTG (final concentration 500 uM) was added for inducing protein expression. Cells were harvested by centrifugation after 72 h at 18 °C. Pellets were suspended in Tris-buffer (100 mM, pH 7.5) and disrupted by sonication (15 s on, 15 s off, 15 cycles, 12 watts output) in an ice bath. Cell debris was removed by centrifugation (12,000 rpm, 15 min, 4

°C) and the supernatant was filtered through a 0.45 mm PES filter. The clarified cell lysates were adjusted to 10 mg/mL (measured using Bradford assay[126]) and stored in small batches at −20 °C.

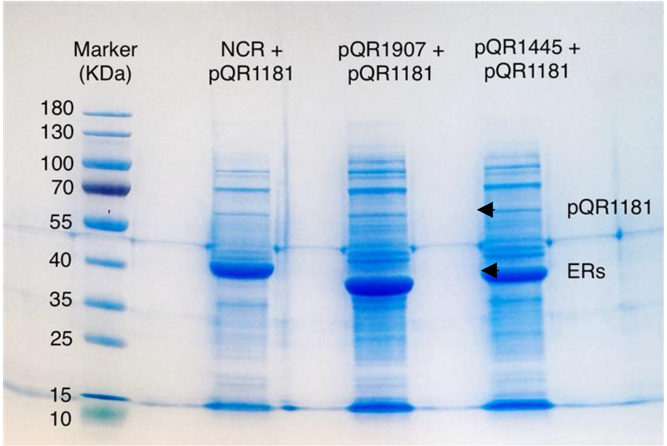


**Figure S1 SDS-Page to monitor enzyme expression.** Lane 1: Protein ladder (Thermo Scientific™ PageRuler™ Prestained Protein Ladder, 10 to 180 kDa). Lane 2: NCR+pQR1181. Lane 3: pQR1907+pQR1181. Lane 4: pQR1445+pQR1181.

## Enzyme reactions

Final concentrations of 10 mM alkene starting materials, 50 mM D-glucose 6-phosphate and 1 mM $NAD^+$ were added to Tris-HCl buffer (100 mM, pH 7.5) with 20% DMSO. Reactions were initiated by adding 10% (v/v) enzyme mixtures (40% (v/v) enzyme mixtures for (*Z*)-esters starting materials, total concentrations of 10 mg/mL) of ERED (NCR/pQR1907/pQR1445) and G6PDH (pQR1181). Reactions were performed with a Thermo mixer at 30 °C, 700 rpm for 16 h. Afterwards, the reaction mixture was extracted with EtOAc, and washed with ice cold $H_2O$, brine and dried over $MgSO_4$. Solvents were then removed under vacuum, and the samples were store at –20 °C until analysed by HPLC.

## Kinetics study

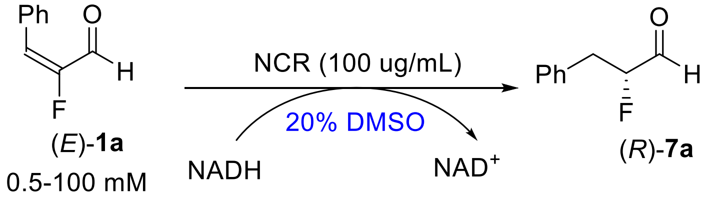


Enzymes NCR was purified and adjusted to 1 mg/mL. As the NADH has high absorbance under UV, UV absorbance was not suitable for measurement of the enzyme activity (initial enzyme rate). Instead, this was measured using HPLC analysis as described in SI ‘HPLC and GC Traces’ (against standard curve). Substrates (*E*)-**1a** were measured at the final concentration of 0, 0.5, 1, 2, 5, 10, 20, 50 and 100 mM (1 equivalent) and co-factor NADH at 1.2 equivalent. Reactions were initialised by adding 10% v/v of the enzyme (at a final concentration of 100 µg/mL) and performed in Eppendorf® Safe-Lock micro test tubes (1.5 mL, Eppendorf, UK) at a total volume of 1 mL in KPi

buffer (100 mM, pH 7.5). 200 µL Samples were taken from each reaction at 5 min, 10 min, 20 min, 30 min and 1h. These samples were extracted immediately with 200 µL ethyl acetate and the ethyl acetate layer were analysed with GC. Each reaction was performed in triplite. The $K_m$ and the $k_{cat}$ were calculated using Origin Software. The $K_m$ and the $k_{cat}$ were calculated using Origin Software (Figure S2).

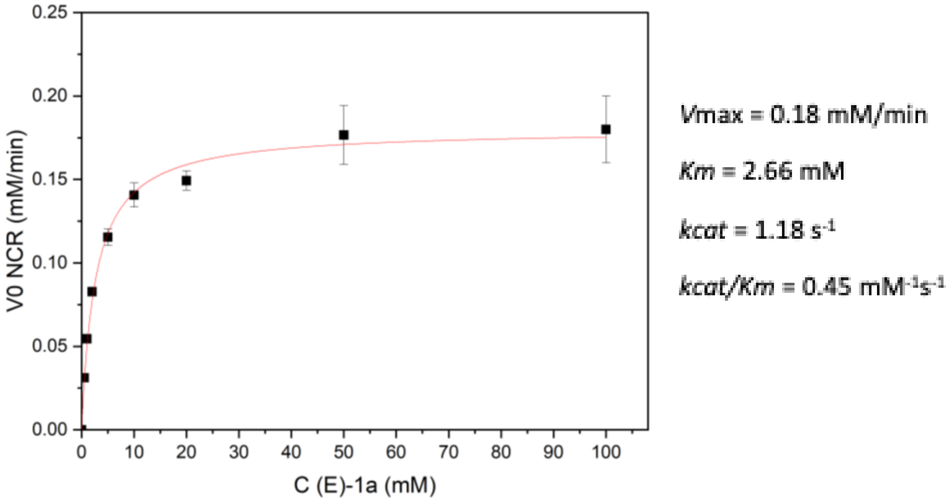


**Figure S2 Fitting of the Michaelis-Menten Function for the NCR with (*E*)-1a using Origin Software.**

# Co-solvent screening

Different co-solvents were screened for enzyme reactions as the alkene starting materials do not dissolve well in aqueous buffer. Enzyme reactions were carried out with (*E*)-**6b** and 40% (v/v) enzyme mixtures. MeOH, EtOH, IPA, *t*BuOH, MeCN, DMSO, THF, DMF, PEG500, PEG200, ethylene glycerol and triethylene glycerol at 10-40% (v/v) were tested. Biphasic reactions were also tested using hexane, CPME, MTBE, toluene and isooctane as the organic layer. As shown in **Figure S3**, co-solvents affected enzyme reactions significantly, and NCR had low tolerance to some organic solvents. The 20% (v/v) DMSO gave the best performance among the co-solvents.

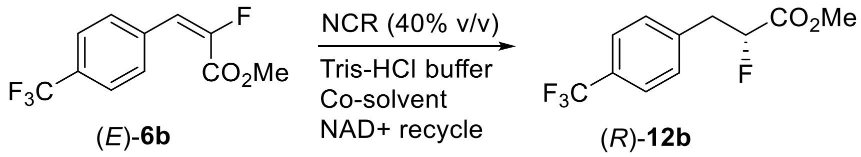


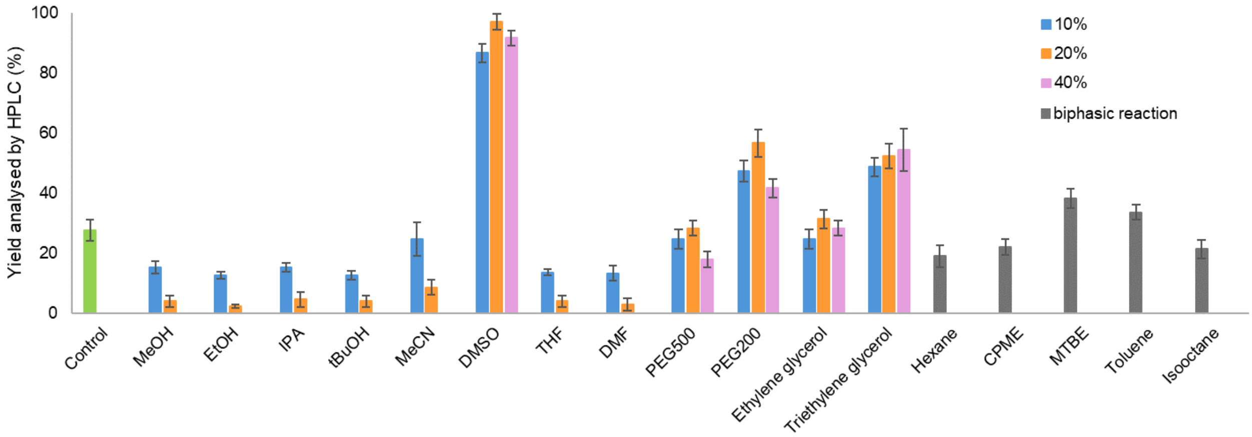


**Figure S3 Co-solvents screening for enzyme reactions.**

# Enzyme screening

All substrates were screened against 3 different EREDs (NCR/pQR1907/pQR1445). Results can be seen in Table S1.

**Table S1:** Enzyme screen. Substrate (10 mM), ERED and G6PDH lysates (10% (v/v), co-expression, total protein in the lysates 1 mg mL$^{-1}$), NAD$^+$ (1 mM), G6PNa (50 mM), in Tris–HCl (100 mM) and DMSO (20%) at pH 7.5, 30 °C, 16 h, 700 rpm. Reactions were performed in triplicate. Yields and enantiomeric ratios were determined by HPLC or GC analysis.

| | | **NCR** | | **pQR1907** | | **pQR1445** | |
|---|---|---|---|---|---|---|---|
| Substrates | Products | Yield (%) | *e.r.*(*R*:*S*) | Yield (%) | *e.r.* (*R*:*S*) | Yield (%) | *e.r.* (*R*:*S*) |
| (*Z*)-**1a** | (*S*)-**7a** | 98.3 | 1:99 | 98.3 | 2:98 | 98.3 | 1:99 |
| (*Z*)-**1b** | (*S*)-**7b** | 98.7 | 3:97 | 95 | 2:98 | 97 | 2:98 |
| (*Z*)-**1c** | (*S*)-**7c** | 97.7 | 6:94 | 84 | 6:94 | 86.7 | 6:94 |
| (*Z*)-**1d** | (*S*)-**7d** | 96.7 | 1:99 | 97 | 1:99 | 99 | 1:99 |
| (*Z*)-**1e** | (*S*)-**7e** | 93.3 | 2:98 | 98.3 | 2:98 | 99.3 | 3:97 |
| (*Z*)-**1f** | (*S*)-**7f** | 97.7 | 8:92 | 22.3 | 7:93 | 60.7 | 2:98 |
| (*Z*)-**2a** | (*S*)-**8a** | 53.7 | 5:95 | 18 | 10:90 | 94 | 9:91 |
| (*Z*)-**3a** | (*S*)-**9a** | 99.7 | 1:99 | 99.3 | 1:99 | 99.7 | 1:99 |
| (Z)-**3b** | (*S*)-**9b** | 97 | 1:99 | 84 | 1:99 | 92.3 | 1:99 |
| (Z)-**5a** | (*S*)-**11a** | 2 | - | 0 | - | 0 | - |
| (Z)-**5b** | (*S*)-**11b** | 99.7 | 2:98 | 99.3 | 2:98 | 99.7 | 2:98 |
| (*E*)-**1a** | (*R*)-**7a** | 99.3 | 97:3 | 95.7 | 98:2 | 98.7 | 98:2 |
| (*E*)-**1b** | (*R*)-**7b** | 99.7 | 95:5 | 96.7 | 94:6 | 98 | 96:4 |
| (*E*)-**1c** | (*R*)-**7c** | 99.3 | 99:1 | 93.3 | 99:1 | 96.3 | 98:2 |
| (*E*)-**1d** | (*R*)-**7d** | 99.3 | 98:2 | 98 | 98:2 | 98.3 | 99:1 |
| (*E*)-**1e** | (*R*)-**7e** | 78.3 | 67:33 (WT-NCR) 90:10 (NCR-G270Y) | 52 | 60:40 | 47.3 | 40:60 |
| (*E*)-**1f** | (*R*)-**7f** | 99.7 | 99:1 | 29.3 | 99:1 | 80.7 | 99:1 |
| (*E*)-**2a** | (*R*)-**8a** | 40 | 90:10 | 16 | 83:17 | 93.7 | 88:12 |
| (*E*)-**2b** | (*R*)-**8b** | 99.3 | 98:2 | 89.3 | 95.5 | 51.4 | 95:5 |
| (*E*)-**3a** | (*R*)-**9a** | 99 | 98:2 | 99 | 98:2 | 93.7 | 80:20 |
| (*E*)-**3c** | (*R*)-**3c** | 99.3 | 86:14 | 99.3 | 60:40 | 72.4 | 55:45 |
| (*E*)-**3d** | (*R*)-**3d** | 82.3 | 78:22 | 56.4 | 40:60 | 48.3 | 56:44 |
| (*E*)-**4a** | (*R*)-**10a** | 99.7 | 99:1 | 4.8 | 93:7 | 20.3 | 99:1 |
| (*E*)-**4b** | (*R*)-**10b** | 99.7 | 99:1 | 78.2 | 99:1 | 31.8 | 98:2 |
| (*E*)-**5a** | (*R*)-**11a** | 98.3 | 99:1 | 98.3 | 99:1 | 98.3 | 99:1 |
| (*E*)-**3a** | (*R*)-**12a** | 54.7 | 99:1 | 4.3 | 98:2 | 9 | 99:1 |
| (*E*)-**3b** | (*R*)-**12b** | 98.3 | 99:1 | 25.3 | 99:1 | 33 | 98:2 |
| (*E*)-**3c** | (*R*)-**12c** | 42.7 | 99:1 | 3 | 99:1 | 3.7 | 99:1 |
| (*E*)-**3d** | (*R*)-**12d** | 98.7 | 99:1 | 0 | - | 0 | - |
| (*E*)-**3e** | (*R*)-**12e** | 0 | - | 0 | - | 0 | - |

# Compounds not accepted by the enzymes

The following compounds were tested in with the three enzymes reported above. No desired product was seen (HPLC or GC analysis, by comparison with the reduced reference standards):

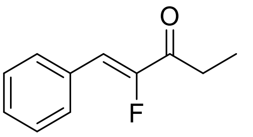

(*Z*)-**5a**

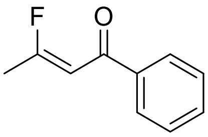

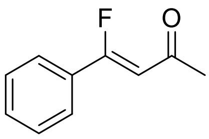

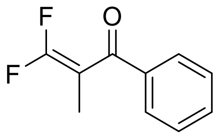

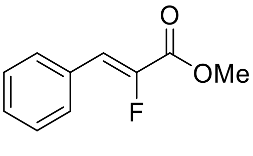

(*Z*)-**6a**

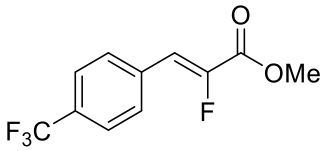

(*Z*)-**6b**

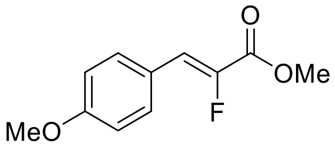

(*Z*)-**6c**

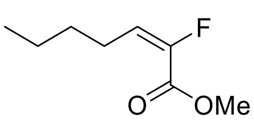

(*E*)-**6e**

Starting material was observed in all cases except in the case of 3,3-difluoro-2-methyl-1-phenylprop-2-en-1-one, where complete consumption of the starting material was seen. However, analysis by GC indicated that the product was not the desired 3,3-difluoro-2-methyl-1-phenylpropan-1-one.

# Preparative Scale Reactions & Green Metrics

*3-Fluoro-4-phenylbutan-2-one* (**7a**)

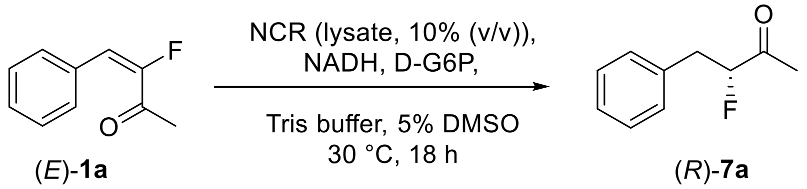


Enzyme reaction run on 130 mg scale. The reaction mixture contains 130 mg of (*E*)-**1a** (10 mM), 1.2 g of D-G6P (disodium salt, 5 mM) and 52 mg of $NAD^+$ (1 mM) in 67.15 mL of Tris-HCl buffer (100 mM, pH 7.5) with 3.95 mL (5% v/v) DMSO. Enzyme lysates of the co-expressed ERs and G6PDH was added at 10% v/v. Reaction was performed using a thermo mixer at 30 °C, 700 rpm. After 16 h, the reaction mixture was extracted with $Et_2O$, washed with ice cold $H_2O$ (3 x 100 mL), brine (100 mL), dried ($MgSO_4$), filtered and concentrated to give (*R*)-**7a** as a yellow oil, no further purification was necessary (113 mg, 86%). HPLC: >99:1 er, $t_{R(major)}$ 5.5 min. $t_{R(minor)}$ 6.0 min (AD-H, 1% IPA/hexane, 1 mL/min). Data in agreement with that reported above.

$[\alpha]_D^{25}$ = +109 (c = 0.8 in $CHCl_3$).

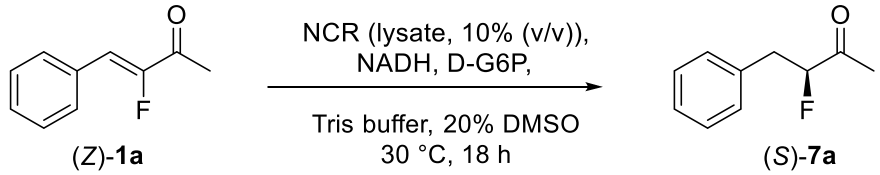


The enzyme reaction was performed on a 30 mg scale. The reaction mixture contained 30 mg of (*Z*)-**1a** (15 mM), 234 mg of D-G6P (75 mM) and 8 mg of $NAD^+$ (1 mM) in 12 mL of Tris-HCl buffer (100 mM, pH 7.5) with 20% (v/v) DMSO. Enzyme lysates of the co-expressed ERs and G6PDH were added at 10% v/v. Reactions were performed using a thermo mixer at 30 °C, 700 rpm. After 16 h the reaction mixture was extracted with EtOAc, washed with ice cold $H_2O$ (5 mL), brine (5 mL), dried ($MgSO_4$), filtered and concentrated to give (*S*)-**7a** as a yellow oil, no further purification was necessary (27 mg, 89%). HPLC: 2:98 er, $t_{R(minor)}$ 5.5 min. $t_{R(major)}$ 6.0 min (AD-H, 1% IPA/hexane, 1 mL/min). Data in agreement with that reported above.

$[\alpha]_D^{25}$ = −92 (c = 0.1 in $CHCl_3$), (Lit.[34] −69, (95:5 er, c = 0.1 in $CHCl_3$)).

As expected from the HPLC traces, the specific rotation of the product from (*Z*)-**1a** was the opposite of that found for the (*E*)-alkene (in agreement with our modelling studies).

**Calculation of green metrics for the preparation of (*R*)-7a using biocatalysis**

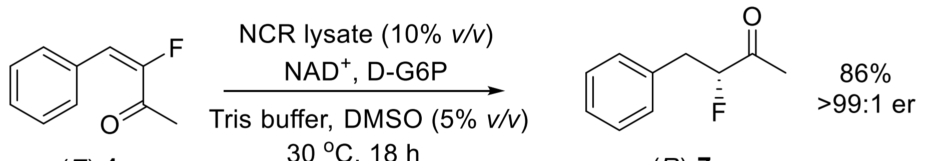


(*E*)-**1a**
130 mg

(*R*)-**7a**
113 mg

| Material | Weight (g) | Volume (mL) |
|---|---|---|
| (*E)*-**1a** | 0.13 | |
| D-G6P | 1.2 | |
| $NAD^+$ | 0.052 | |
| Water | 67.15 | 67.15 |
| TRIS-HCl | 1.06 | |
| DMSO | 4.35 | 3.95 |
| (*R)*-**7a** | 0.113 | |

**E-factor**[65]

*Total input:*

(0.13 g (*E)*-**1a**) + (1.2 g D-G6P) + (0.052 g $NAD^+$) + (1.06 g Tris-HCl) + (4.35 g DMSO)

= 6.792 g

*Waste:*

6.792 g (input) – 0.113 g (product) = 6.679 g

*E factor:*

6.679 g (waste) / 0.113 g (product) = **59**

**Process Mass Intensity**[66]

*Total input (inc. $H_2O$):*

(0.13 g (*E)*-**1a**) + (1.2 g D-G6P) + (0.052 g $NAD^+$) + (1.06 g Tris-HCl) + (4.35 g DMSO) + (67.15 g $H_2O$) + (6.72 g $H_2O$ in lysate)

= 80.662 g

*PMI:*

80.662 g (material input) / 0.113 g (product) = **714**

**EcoScale**[67]

| Parameter | Penalty Points | Comments |
|---|---|---|
| *Yield* | [(100-86)/2] = 7 | 86% yield |
| *Price of reaction components* | 5 | Cost of D-G6P |
| *Safety* | 5 | Flammable solvent used in workup (EtOAc or $Et_2O$) |
| *Technical Setup* | 0 | Common setup |
| *Temperature/Time* | 1 | 18 h reaction time |
| *Workup/Purification* | 3 | Product pure after liquid-liquid extraction |
| **Total:** | 21 | |

EcoScale:

100 – [Sum of penalty points] = 100 – 19 = **79**

## Calculation of green metrics for the preparation of (*S*)-7a using asymmetric hydrogenation

S. Ponra, J. Yang, S. Kerdphon, P. G. Andersson, "Asymmetric Synthesis of Alkyl Fluorides: Hydrogenation of Fluorinated Olefins" *Angew. Chem. Int. Ed.* 2019, **58**, 9282-9287[34]

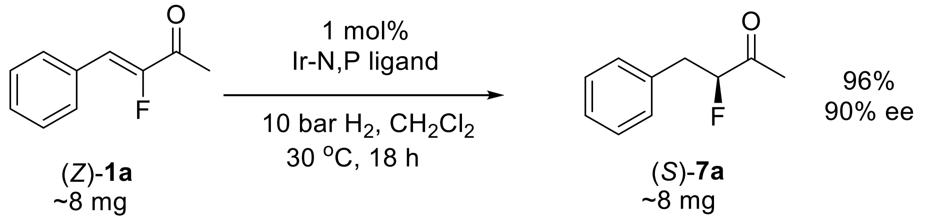


(*Z*)-**1a**
~8 mg

(*S*)-**7a**
~8 mg

| Material | Weight (mg) | Volume (mL) |
|---|---|---|
| (*Z*)-**1a** | 8 | |
| Ir-N,P catalyst | ~1 | |
| $CH_2Cl_2$ | 665 | 0.5 |
| 3 x Argon purge* | 73 | 45 |
| 3 x Hydrogen* | 16 | 45 |
| Hydrogen (10 bar)* | 12 | 15 at 10 bar |
| | | |

*The total reactor volume was estimated to be 15 $cm^3$ in order to calculate gas quantities.

**E-factor**[65]

*Total input:*

(8 mg (*Z*)-**1a**) + (1 mg catalyst) + (665 mg $CH_2Cl_2$) + (73 mg Argon) + (48 mg Hydrogen)

= 775 mg

*Waste:*

775 mg (input) – 8 mg (product) = 667 mg

*E factor:*

667 mg (waste) / 8 mg (product) = **96**

**Process Mass Intensity**[66]

*Total input:* 775 mg

*PMI:*

775 mg (material input) / 8 mg (product) = **97**

**EcoScale**[67]

| Parameter | Penalty Points | Comments |
|---|---|---|
| *Yield* | [(100-96)/2] = 2 | 96% yield |
| *Price of reaction components* | 5 | Cost of catalyst |
| *Safety* | 10 | $H_2$ highly flammable |
| *Technical Setup* | 4 | High pressure; gas atmosphere |
| *Temperature/Time* | 1 | 24 h reaction time |
| *Workup/Purification* | 10 | Silica purification |
| **Total:** | 32 | |

EcoScale:

100 – [Sum of penalty points] = 100 – 32 = **68**

## Calculation of green metrics for the preparation of (*R*)-10a using asymmetric organocatalysis

P. Kwiatkowski, T. D. Beeson, J. C. Conrad, D. W. C. MacMillan, "Enantioselective Organocatalytic α-Fluorination of Cyclic Ketones" *J. Am. Chem. Soc.* 2011, **133**, 1738–1741.[22]

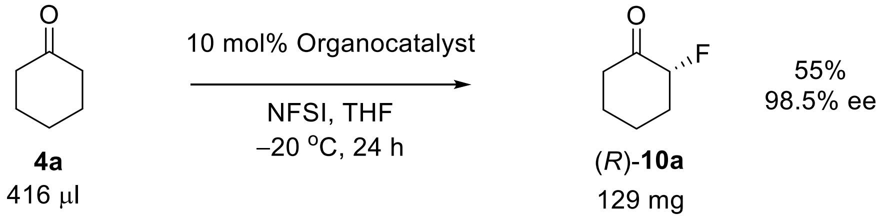


Note: NFSI is the limiting reagent (0.5 eq wrt ketone)

| Material | Weight (mg) | Volume (mL) |
|---|---|---|
| **4a** | 394 | 0.416 |
| NFSI | 651 | |
| $Na_2CO_3$ | 319 | |
| THF | 5330 | 6 |
| 0.1 M catalyst solution in THF | 1881 | 2 |
| | | |
| | | |

*5 mL of the catalyst solution contains: 0.5 mmol 9-amino(9-deoxy)-epi-dihydroquinidine (163 mg), 86 mg trichloroacetic acid, 5 mL THF and 0.5 mmol water. Total mass of solution is 163+86+4445+9=4.703 g.

**E-factor**[65]

*Total input:*

(394 mg **4a**) + (651 mg NFSI) + (319 mg $Na_2CO_3$) + (5330 mg THF) + (1881 mg catalyst solution)

= 8575 mg

*Waste:*

8256 mg (input) – 129 mg (product) = 8446 mg

*E factor:*

8127 mg (waste) / 129 mg (product) = **65**

**Process Mass Intensity**[66]

*Total input:* 8256 mg

*PMI:*

8575 mg (material input) / 129 mg (product) = **66**

**EcoScale**[67]

| Parameter | Penalty Points | Comments |
|---|---|---|
| *Yield* | [(100-55)/2] = 22.5 | 55% yield |
| *Price of reaction components* | 5 | Cost of catalyst |
| *Safety* | 15 | Use of THF (flammable), $Et_2O$ and pentane (highly flammable, harmful to aquatic environment) |
| *Technical Setup* | 1 | Inert atmosphere |
| *Temperature/Time* | 5 | Cooling to < 0 °C |
| *Workup/Purification* | 10 | Silica purification |
| **Total:** | 58.5 | |

EcoScale:

100 – [Sum of penalty points] = 100 – 58.5 = **42** (rounded)

*3-Fluoro-4-(thiophen-2-yl)butan-2-one* (**8b**)

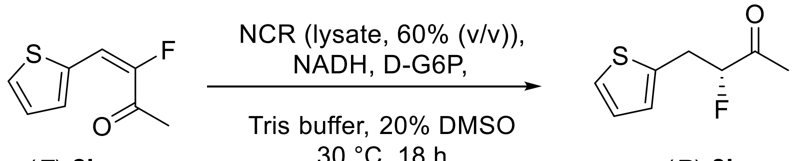


(*E*)-**2b** (*R*)-**8b**

Enzyme reaction run on 18 mg scale. The reaction mixture contains 18 mg of (*E*)-**2b** (10 mM), 140 mg of D-G6P (disodium salt, 50 mM) and 6 mg of $NAD^+$ (1 mM) in 2 mL of Tris-HCl buffer (100 mM, pH 7.5) with 2 mL (20% v/v) DMSO. Enzyme lysates of the co-expressed ERs and G6PDH was added at 60% v/v. Reaction was performed using a thermo mixer at 30 °C, 700 rpm. After 16 h, the reaction mixture was extracted with $Et_2O$, washed with ice cold $H_2O$ (3 x 10 mL), brine (10 mL), dried ($MgSO_4$), filtered and concentrated to give a yellow oil, no further purification was necessary (16 mg, 88%). GC: 98:2 er, $t_{R(major)}$ 16.3 min. $t_{S(minor)}$ 16.0 min (Supelco Beta Dex 225 capillary GC column (30 m × 250 µm, 0.25 µm), method: 50 °C (hold 3 min), 50 °C to 170 °C (5 °C/min), 170 °C (hold 1 min).

$[\alpha]_D^{25}$ = −49 (c = 0.5 in $CHCl_3$).

*3-Fluoro-4-phenylpentan-2-one* (**11a**)

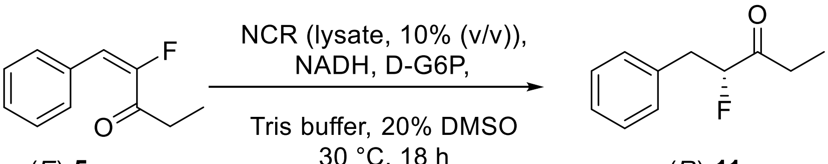


(*E*)-**5a** (*R*)-**11a**

The enzyme reaction was performed on a 20 mg scale. The reaction mixture contained 20 mg of (*E*)-**5a** (10 mM), 143 mg of D-G6P (50 mM) and 7.3 mg of $NAD^+$ (1 mM) in 11 mL of Tris-HCl buffer (100 mM, pH 7.5) with 20% (v/v) DMSO. Enzyme lysates of the co-expressed ERs and G6PDH were added at 10% v/v. Reaction performed using a thermo mixer at 30 °C, 700 rpm. After 16 h the reaction mixture was extracted with EtOAc, washed with ice cold $H_2O$ (5 mL), brine (5 mL), dried ($MgSO_4$), filtered and concentrated to give (*R*)-**11a** as a yellow oil, no further purification was necessary (18 mg, 92%). HPLC: 98:2 er, $t_{R(major)}$ 13.7 min. $t_{R(minor)}$ 16.4 min (OJ-H, 1% IPA/hexane, 1 mL/min). Data in agreement with that reported above.

$[\alpha]_D^{25}$ = +108 (c = 1.1 in $C_6H_6$), (Lit.[108] +29, (>98:2 er, c = 1.3 in $C_6H_6$)).

# Post-Modification (13, 14, 15)

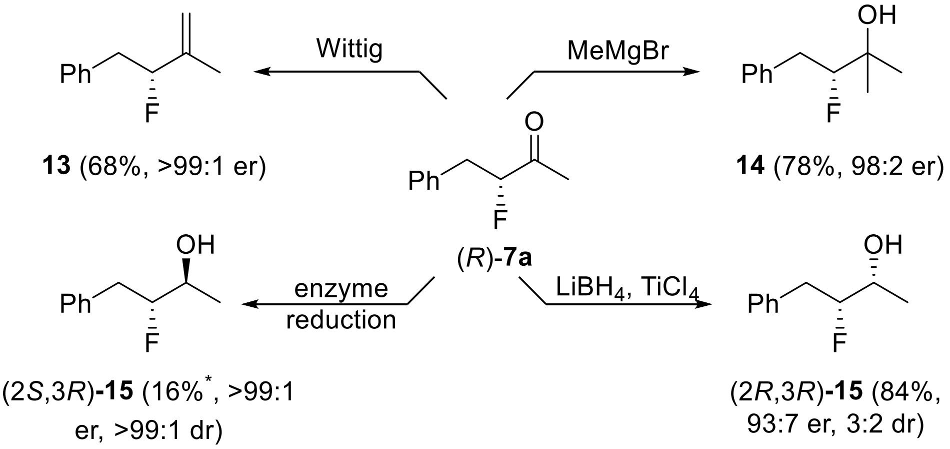


Authentic racemic reference samples for HPLC analysis were synthesised in an analogous manner to the compounds synthesised below.

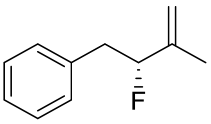


*(R)-(2-fluoro-3-methylbut-3-en-1-yl)benzene* (*R*)-**13**: A suspension of methyltriphenyl phosphonium bromide (54 mg, 0.15 mmol) in THF (1.2 mL) was cooled to -78 °C and treated with $^{n}$BuLi (56 $\mu$L, 0.14 mmol, 2.5 M solution in hexanes) added dropwise. The reaction mixture was stirred at -78 °C for 20 min, 0 °C for 40 min and 25 °C for 1 h. The reaction was cooled back down to -78 °C and treated a solution of (*R*)-3-fluoro-4-phenylbutan-2-one (15 mg, 0.09 mmol) in THF (0.5 mL) added dropwise. The reaction mixture was stirred at 25 °C overnight, quenched with $NaHCO_{3(sat.\ aq.)}$ and extracted with pentane. The combined organic extracts were dried ($MgSO_4$) and filtered. The resultant crude product was diluted with pentane and filtered through a short silica pad. The filtrate was concentrated (evaporation of solvent at rt and pressure, extremely volatile) to give (*R*)-**13** as a pale yellow oil (10 mg, 68%, >99:1 er). $[\alpha]_D^{25}$ = +24.8 (c = 0.25, $CHCl_3$); HPLC: >99:1 er, $t_{R(major)}$ 8.1 min (AD-H, 100% hexane, 0.5 mL/min); $\upsilon_{max}$ (oil) 3030 (s), 2951 (s), 2923 (s), 2852 (m), 1652 (s), 1606 (s), 1496 (s), 1454 (m) $cm^{-1}$; $^{1}H$ NMR (400 MHz, $CDCl_3$) δ 7.26-7.12 (m, 5H), 5.05-4.80 (m, 3H), 3.04-2.78 (m, 2H), 1.73 (s, 3H); $^{13}C$ NMR (101 MHz, $CDCl_3$) δ 143.0 (C, d, *J* = 18.1 Hz), 137.3 (C, d, *J* = 4.0 Hz), 129.5 (CH, s), 128.6 (CH, s), 126.8 (CH, s), 113.5 ($CH_2$, d, *J* = 9.7 Hz), 96.5 (CH, d, *J* = 173.1 Hz), 40.6 ($CH_2$, d, *J* = 23.6 Hz), 17.6 ($CH_3$, d, *J* = 3.2 Hz); $^{19}F$ NMR (376 MHz, $CDCl_3$) δ -176.2 (ddd, *J* = 47.5, 29.1, 17.8 Hz); [HRMS, ESI] Calcd. $(C_{11}H_{13}F+H)^+$ 165.1079, loss of HF seen under ESI and EI conditions, Calcd. $(C_{11}H_{12}+H)^+$ 145.1012 found 145.1010.

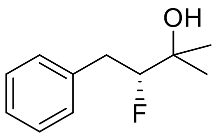

*(R)-3-fluoro-2-methyl-4-phenylbutan-2-ol* ((*R*)-**14**): To a solution of (*R*)-3-fluoro-4-phenylbutan-2-one (20 mg, 0.12 mmol) in THF (0.6 mL) was added 3.0 M THF solution of MeMgCl (103 $\mu$L, 0.31 mmol) at 0 °C, and the mixture was stirred for 30 min. The reaction was quenched with $NH_4Cl_{(sat.\ aq.)}$ and extracted with $Et_2O$ (3 x 10 mL). The combined organic layers were washed with brine, dried ($MgSO_4$), filtered, and conc. *in vacuo*. The crude mixture was re-dissolved in 15% $Et_2O$:hexanes, filtered through a short silica plug and the solvent removed *in vacuo* to give (*R*)-**14** as a pale yellow oil (17 mg, 78%, 92:8 er). $[\alpha]_D^{25}$ = +26.9 (c = 0.75, $CHCl_3$); HPLC: 92:8 er, $t_{R(major)}$ 11.2 min, $t_{R(minor)}$ 13.5 min, (AD-H, 1% IPA/hexane, 1 mL/min); $\upsilon_{max}$ (oil) 3567 (m), 3403 (m), 3029 (m), 1653 (s), 1635 (s), 1605 (m), 1455 (m) $cm^{-1}$; $^1$H NMR (400 MHz, $CDCl_3$) δ 7.39-7.20 (5H, m), 4.44 (1H, ddd, *J* = 48.0, 8.8, 3.6 Hz), 3.03-2.84 (2H, m), 1.31 (6H, s); $^{13}$C NMR (101 MHz, $CDCl_3$) δ 138.1 (C), 129.3 (CH, s), 128.7 (CH, s), 126.7 (CH, s), 100.0 (CH, d, *J* = 176.0 Hz), 72.1 (C, d, *J* = 20.4 Hz), 36.4 ($CH_2$, d, *J* = 21.7 Hz), 25.5 ($CH_3$, d, *J* = 4.0 Hz), 24.9 ($CH_3$, d, *J* = 3.4 Hz);$^{19}$F NMR (376 MHz, $CDCl_3$) δ -189.9 to -190.3 (m); [HRMS, ESI] Calcd. $(C_{11}H_{15}FO+H)^+$ 183.1185, loss of HF seen under ESI and EI conditions, Calcd. $(C_{11}H_{14}O+H)^+$ 163.1117 found 163.1116.

*(2S,3R) and (2R,3S)-3-Fluoro-4-phenylbutan-2-ol* ((2*S*,3*R*)-**15**) and (2*R*,3*R*)-**15**):

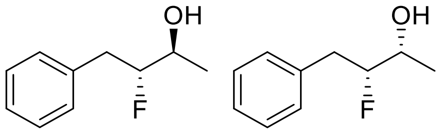

(*R*)-3-Fluoro-4-phenylbutan-2-one (20 mg, 0.12 mmol) was dissolved in anhydrous methanol (0.5 mL) and cooled to 0 °C. $NaBH_4$ (9 mg, 0.24 mmol) was added portion-wise over a period of 5 min, after which time the reaction was warmed to room temperature and stirred for a further 3 h. Subsequently, the reaction was quenched with ice cold water (10 mL) and extracted with diethyl ether (3 x 10 mL). The solvent was removed under reduced pressure and the compound purified by preparative TLC (20% EtOAc:Hexane), to give **15** as a colourless oil (17 mg, 84%, 93:7 er) as a 2:3 mixture of diastereomers anti:syn (The assignment of stereochemistry to the diastereisomers of this compound is contradictory in the literature,[127,128] so a detailed NMR analysis was carried out with the aid of DFT calculations – see below). $[\alpha]_D^{25}$ = +25.8 (c = 0.8, $CHCl_3$); HPLC: 93:7 er $t_{R(major)}$ 15.3 min, $t_{R(major)}$ 23.9, $t_{R(minor)}$ 18.0 min, $t_{R(minor)}$ 16.9 min (OD-H, 1% IPA/hexane, 1 mL/min); $\upsilon_{max}$ (oil) 3382 (m), 3029 (m), 1604 (s) $cm^{-1}$; [HRMS, ESI] Calcd. $(C_{10}H_{13}FO+H)^+$ 169.1029, loss of HF seen under ESI conditions, Calcd. $(C_{11}H_{14}O+H)^+$ 149.0961 found 149.0961.

Major diastereomer (*syn* (2*R*,3*R*-**15**)): $^1$H NMR (400 MHz, $CDCl_3$) δ 7.35-7.22 (5H, m), 4.47 (1H, dtd, *J* = 48.2, 6.4, 4.6 Hz), 3.80 (1H, dqd, *J* = 17.9, 6.5, 4.6 Hz), 3.05-2.94 (2H, m), 1.27 (3H, d, *J* = 6.5 Hz); $^{13}$C NMR (100 MHz, $CDCl_3$) δ 136.9 (C, d, *J* = 4.6 Hz), 129.5 (CH, s), 128.7 (CH, s), 126.9 (CH, s), 97.6 (CH, d, *J* = 173.7 Hz), 68.5 (CH, d, *J* = 20.1

Hz), 37.7 ($CH_2$, d, $J$ = 21.7 Hz), 19.0 ($CH_3$, d, $J$ = 5.3 Hz); $^{19}$F NMR (376 MHz, $CDCl_3$) δ -192.9 to -193.2 (m). Data in agreement with that reported in the literature[127] and NMR analysis reported below.

Minor diastereomer (*anti* (2*R*,3*R*-**15**)): $^{1}$H NMR (500 MHz, $CDCl_3$) δ 7.38-7.20 (5H, m), 4.59 (1H, ddt, $J$ = 48.0, 8.3, 4.0 Hz), 3.93 (1H, dqd, $J$ = 19.7, 6.5, 4.6 Hz), 2.99-2.85 (2H, m), 1.28 (3H, d, $J$ = 6.4 Hz); $^{13}$C NMR (126 MHz, $CDCl_3$) δ 137.3 (C, d, $J$ = 2.7 Hz), 129.4 (CH, s), 128.7 (CH, s), 126.8 (CH, s), 97.3 (CH, d, $J$ = 173.9 Hz), 68.8 (CH, d, $J$ = 22.9 Hz), 36.7 ($CH_2$, d, $J$ = 21.1 Hz), 17.9 ($CH_3$, d, $J$ = 5.9 Hz); $^{19}$F NMR (376 MHz, $CDCl_3$) δ -190.3 - -190.7 (m).

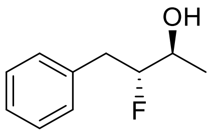


*(2S,3R)-3-Fluoro-4-phenylbutan-2-ol* ((2*S*,3*R*)-**15**)*:* Enzyme reaction run on 10 mg scale. The reaction mixture contained (*R*)-3-fluoro-4-phenylbutan-2-one (10 mg, 10 mM), NADH (21 mg, disodium salt, 5 mM), $ZnCl_2$ (0.8 mg, 1 mM), acetone (50 *μ*L) in KPi buffer (2.95 mL, 100 mM, pH 7.5) with DMSO (0.6 mL, 10% v/v). Enzyme lysates of co-expressed Horse Liver Aldehyde Dehydrogenase (HLADH) and G6PDH were added at 40% v/v. Reaction was performed using a thermo mixer at 25 °C, 700 rpm. After 16 h, the reaction mixture was extracted with $Et_2O$, washed with ice cold $H_2O$ (3 x10 mL), brine (10 mL), dried ($MgSO_4$), filtered and conc. *in vacuo* to give a yellow oil (18% NMR yield, the rest of the material is 3-fluoro-4-phenylbutan-2-one). Ethyl fluoroacetate (5.8 *μL)* was added, the entire solution dissolved in $CDCl_3$ and the yield determined by $^{19}$F NMR spectroscopy using ethyl fluoroacetate as an internal standard. Only a single isomer was detected ((2*S*, 3*R*)-**15**). NMR data in agreement with that reported above. HPLC: >99:1 er $t_{R(major)}$ 23.8 min, (OD-H, 1% IPA/hexane, 1 mL/min).

NMR Comparison of $NaBH_4$ reduction (red, both diastereomers) and HLADH reduction (green, single diastereomer and recovered starting material):

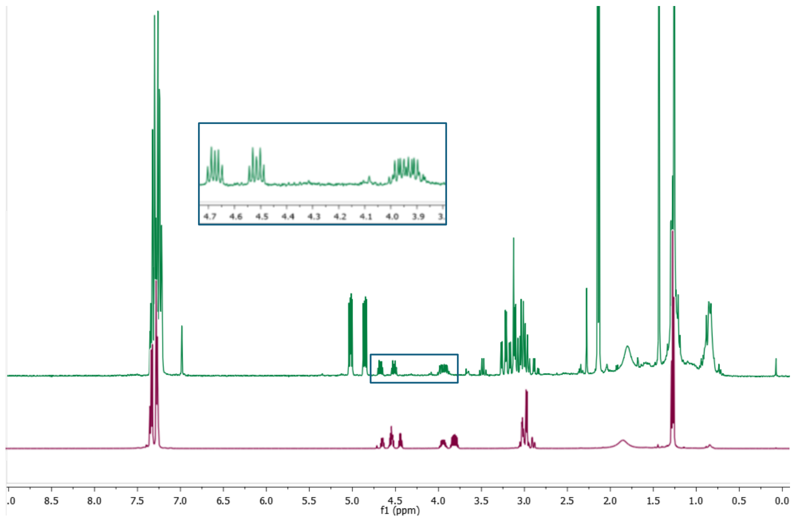

## Cascade Reaction

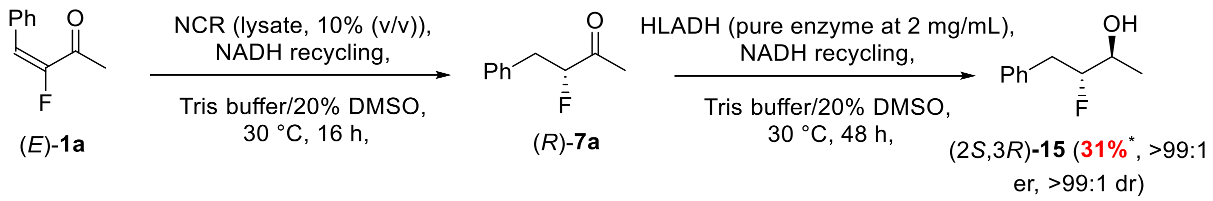


The reaction mixture contained (*E*)-**1a** (3.3 mg, 10 mM), NADH (0.6 mg, disodium salt, 1 mM) and D-G6P (28.2 mg, 50 mM) in Tris buffer (1.4 mL, 100 mM, pH 7.5) with DMSO (400 µL, 20% v/v). Enzyme lysates of co-expressed NCR and G6PDH were added at 10% v/v (200 µL, 10 mg/mL of the total proteins). Reaction was performed using a thermo mixer at 30 °C, 700 rpm overnight (16 h). The reaction was then added $ZnCl_2$ (0.3 mg, 1 mM), acetone (25 $\mu$L) and HLADH (2 mg/mL, Merk, Uk). Reaction was performed at 30 °C, 700 rpm for another 48 h. Then the reaction mixture was extracted with $Et_2O$ (2 mL x 3), washed with ice cold $H_2O$ (2 mL x 3), brine (2 mL), dried ($Na_2SO_4$), filtered and conc. *in vacuo* to give **15** as a yellow oil (31% yield analysed by NMR as described above, HPLC: >99:1 er).

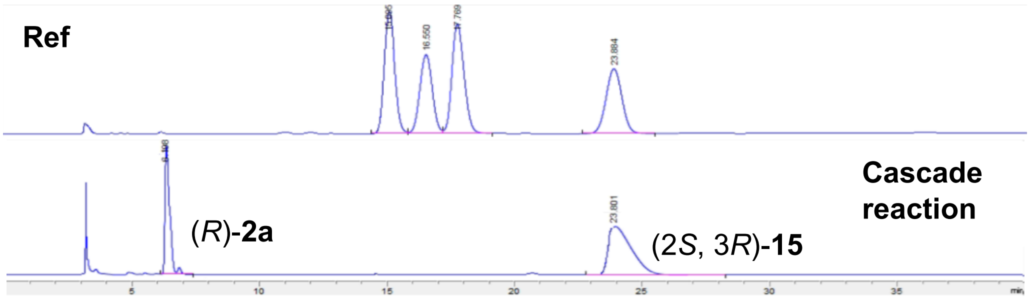

## NMR analysis of compound 15 diastereomers

For alcohol **15** obtained by $NaBH_4$ reduction, the two halves of the multiplet at 4.48 ppm separated by the $J_{HF}$ coupling in the $^1H$ NMR spectrum showed differences: the left half appears as a doublet of doublet of doublets, while the right half is a triplet of doublets (Figure S4). This is due to strong coupling effects which in turn is likely to be caused by the small chemical shift difference for the $CH_2$ protons at ~3 ppm where a strong overlap of signals is observed (Figure S4). Thus, the spectrum cannot be analysed using traditional first-order rules and full line shape analysis[129] is required in order to determine accurately NMR parameters. The results of such analysis are presented in Figure S5. Note that no line shape analysis was undertaken in the literature,[127-128] which have previously reported *J* coupling values for the same compound. $^1H$, $^{13}C$ and $^{19}F$ NMR parameters are summarised in Tables S2 and S3.

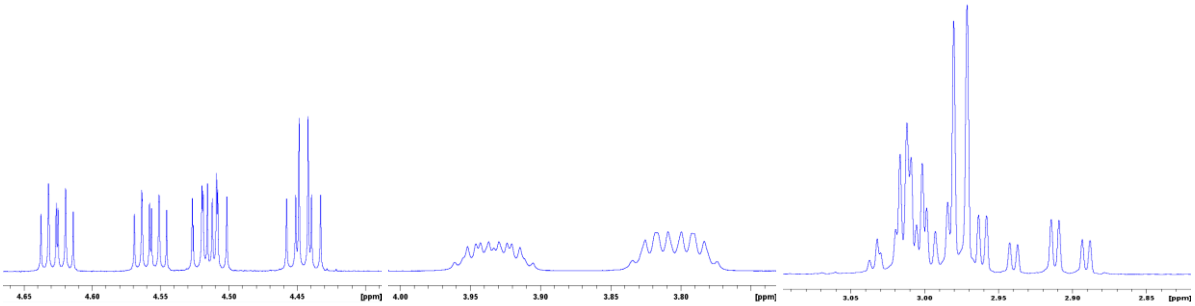


**Figure S4**. $^1H$ NMR spectrum showing multiplets due to CH and $CH_2$ protons of alcohol **15** obtained by chemical methods (in $CDCl_3$, 700 MHz, 298 K). A mixture of two diastereomers (denoted as major and minor) is observed with the ratio major:minor = 1.38:1.

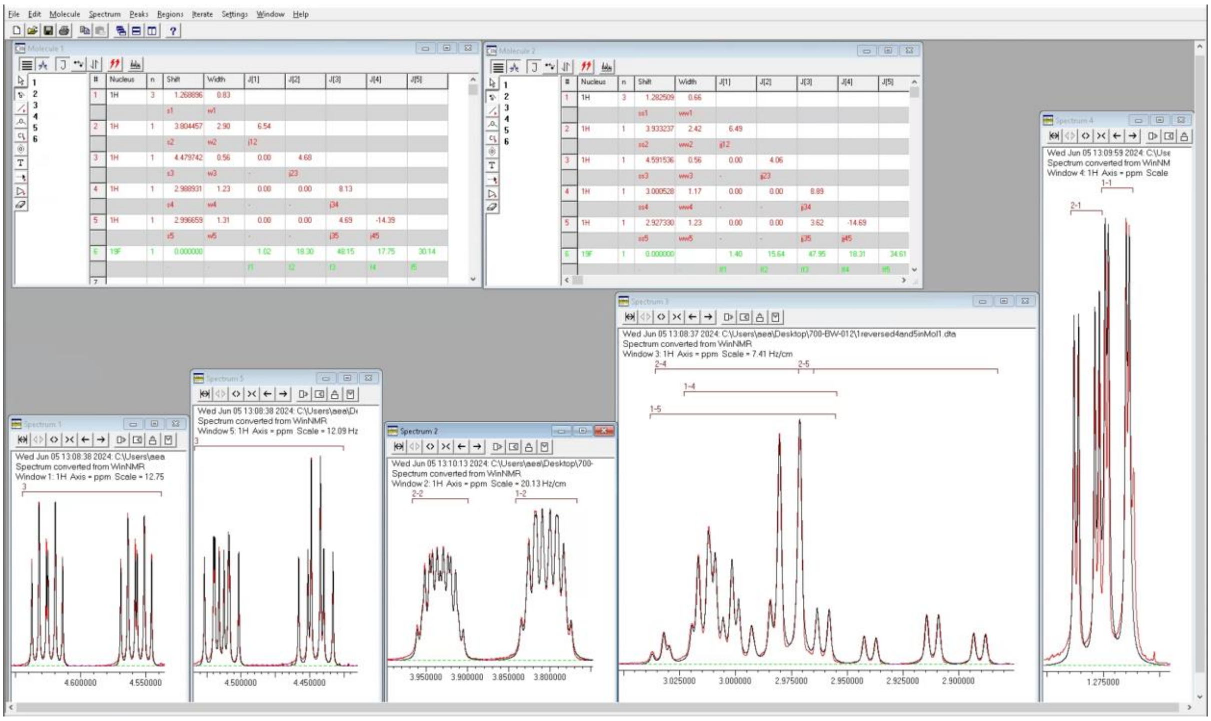

**Figure S5**. The overlaid view of experimental (red) and fitted (black) $^1H$ NMR line shapes of major and minor forms of **15** (in $CDCl_3$, 700 MHz, 298 K). The screenshot of the table of fitting results is also shown (chemical shifts are in ppm; *J* couplings

and linewidths are in Hz). The iterative full line shape analysis of this $^1$H NMR spectrum was carried out using the gNMR program.[130]

**Table S2**. NMR parameters of major and minor forms of **15** in $CDCl_3$ at 298 K. The major:minor ratio measured using $^1$H NMR integral intensities is 1.38:1.

| **Position** | **Major** **$\delta_H$ /ppm** | **Minor** **$\delta_{H/F}$/ppm** | **Major** ***J* / Hz** | **Minor** ***J* / Hz** |
|---|---|---|---|---|
| **1** | 1.27 | 1.28 | 1,2=6.54; 1,F=1.02 | 1,2=6.49; 1,F=1.40 |
| **2** | 3.80 | 3.93 | 2,3=4.68; 2,F=18.30 | 2,3=4.06; 2,F=15.64 |
| **3** | 4.48 | 4.59 | 3,4=8.13; 3,4'=4.69; 3,F=48.15 | 3,4=8.89; 3,4'=3.62; 3,F=47.95 |
| **4** | 2.99 | 3.00 | 4,4'=-14.39; 4,F=17.75 | 4,4'=-14.69; 4,F=18.31 |
| **4'** | 3.00 | 2.93 | 4',F=30.14 | 4',F=34.61 |
| ***o*-Ph** | 7.26 | 7.26 | | |
| ***m*-Ph** | 7.32 | 7.32 | | |
| ***p*-Ph** | 7.25 | 7.25 | | |

**Table S3**. $^{13}$C and $^{19}$F NMR chemical shifts and $J(^{13}C,^{19}F)$ coupling constants for major and minor forms of **15** (in $CDCl_3$ at 298 K).

| | Major $\delta_{C/F}$ / ppm | Minor $\delta_{C/F}$ / ppm | Major $J_{CF}$ / Hz | Minor $J_{CF}$ / Hz |
|---|---|---|---|---|
| **C1** | 18.84 | 17.87 | 5.35 | 5.83 |
| **C2** | 68.46 | 68.79 | 20.15 | 22.85 |
| **C3** | 97.59 | 97.27 | 173.83 | 173.92 |
| **C4** | 37.67 | 36.65 | 21.62 | 21.30 |
| **$C_q$-Ph** | 136.89 | 137.31 | 4.54 | 2.76 |
| **$C_o$-Ph** | 129.51 | 129.41 | | |
| **$C_m$-Ph** | 128.68 | 128.66 | | |
| **$C_p$-Ph** | 126.84 | 126.78 | | |
| **F** | -192.99 | -190.38 | | |

**Assignment of Diastereomer Configurations Using DFT Calculations and NMR Data**

Initially, molecular mechanics calculations were carried out using the PCModel program[131] As the initial ketone used for the reduction had a 3*R*-configuration, two configurations of alcohol **15**, 2*R*,3*R* and 2*S*,3*R*, were considered. Extensive conformational search was carried out to find the conformer with the smallest energy for both diastereomers using the MM3 force field (11.9157 kcal mol$^{-1}$ for 2*S*,3*R* and 11.7909 kcal mol$^{-1}$ for 2*R*,3*R*), which then was used in the grid search routine of the PCModel program. All 4 rotatable bonds (C2−C3, C2−O, C3−C4 and C4−C5) were included with a 30° step for rotations and 20,736 structures were considered in total for each of two diastereomers in the grid search analysis, which identified 19 unique conformers for the 2*S*,3*R* diastereomer and 12 unique conformers for the 2*R*,3*R* diastereomer. The structures were visualised and analysed

using GaussView.[132] The corresponding geometries of the conformers were prepared as initial structures for DFT calculations, which were carried out using the Gaussian 16 program.[133] Two different DFT functionals developed by different groups were considered, which were shown previously[134] to reproduce satisfactorily conformational populations measured experimentally: PW6B95D3[135] and ωB97XD.[136] As a basis set, def2-TZVP was used with these DFT functionals.[137,138] The superfine numerical integration grid (with 175 radial shells and 974 angular points per shell for 1st row atoms and with 250 radial shells and 974 angular points per shell for atoms in the 2nd and later rows) was used in DFT geometry optimisations, combined with the "verytight" convergence condition (requesting the root-mean-square forces to be smaller than $1 \times 10^{-6}$ hartree bohr$^{-1}$). Frequency calculations were subsequently carried out at the same level of theory from which the Gibbs free energies were derived. These calculations were also used to verify that the optimized geometries correspond to true minima by checking whether any imaginary frequency was encountered. On calculating populations from free energies, the harmonic oscillator and rigid rotor models were used. No scaling of calculated frequencies were undertaken and as-calculated frequencies were used (for possible sources of error associated with the use of unscaled frequencies, see Alecu *et al.*[139] Chloroform solvent effects were included in all calculations using the integral equation formalism of the polarizable continuum model (IEFPCM), which is the default *SCRF* (Self-Consistent Reaction Field) method implemented in Gaussian 16.[140,141] Additionally, conductor-like polarizable continuum model (CPCM) was used in $^{19}$F chemical shift calculations.[140-142]

For conformer population calculations, the equilibrium constant was derived from the Gibbs free energy of formation:

$$K = e^{-\frac{\Delta G^0}{RT}} \quad (1)$$

where *R* is the universal gas constant and *T* is the temperature of the system. For the two-site exchange, the equilibrium constant is defined as:

$$K = \frac{p_1}{p_2} \quad (2)$$

where $p_1$ (in %) is the population of the preferred conformer. By calculating the Gibbs free energy differences ($\Delta G°$) using DFT methods and the above two equations, conformer populations can be calculated. For multi-site exchange models, the relative free energies of conformers relative to that of the lowest-energy conformer in a given set of calculations for each diastereomer were calculated. These relative free energies were then used to calculate the corresponding *K* values and populations. In particular, an exchange between 19 conformers was considered for the 2*S*,3*R* diastereomer and between 12 conformers for the 2*R*,3*R* diastereomer. Dihedral angles were also analysed in order rule out duplication of structures after DFT geometry optimisations (Tables S4 and S5). The PW6B95D3-optimised geometries of conformers are shown in Figure S6. Remarkably, a nearly parallel orientation of C−F and O−H bonds is observed in the most preferred conformers for both diastereomers (rr01 and sr01 in Figure S6), as well as in a few other conformers (rr02, rr03, rr04, rr06, sr02, sr04, sr07, sr14 and sr15). Such

orientations put the F atom of the C−F bond and the hydroxyl H atom in close proximity of each other (2.2-2.4 Å) and these are in favour of stabilising O−H…F−C-type non-covalent interactions.

As can be seen from Tables S6 and S7, DFT calculations satisfactorily predict experimental *J* couplings on the assumption that **the major form** identified in the $^1$H NMR spectrum (Figure S4) has the **2*R*,3*R*-configuration** and **the minor form** has the **2*S*,3*R*-configuration**. The root mean square (RMS) deviations for 16 predicted $^3J$ couplings for two diastereomers were 2.01 and 3.80 Hz for PW6B93B and ωB97XD functionals, respectively. The mean absolute deviations (MAD) for 16 predicted $^3J$ couplings for two diastereomers were 1.41 and 2.51 Hz for PW6B93B and ωB97XD functionals, respectively.

The RMS deviations for 8 predicted $^1$H NMR chemical shifts in two diastereomers were 0.096 and 0.142 ppm for PW6B93B and ωB97XD functionals, respectively (Tables S8 and S9; shielding constants of reference compounds are included in Table S10). The MAD values for 8 predicted $^1$H NMR chemical shifts for two diastereomers were 0.074 and 0.128 ppm for PW6B93B and ωB97XD functionals, respectively (Tables S8 and S9). In agreement with the experimental $^{19}$F NMR chemical shifts (major -193.0 ppm and minor -190.4 ppm), a more negative $^{19}$F chemical shift is predicted for the 2R,3R diastereomer (-198.0 and -199.4 ppm by PW6B93B and ωB97XD calculations, respectively) compared to the 2S,3R-diastereomer (-195.4 and -196.0 ppm by PW6B93B and ωB97XD calculations, respectively). Overall, the PW6B93B functional performed better than the ωB97XD functional in reproducing experimentally measured parameters, as before.[134]

On reversing the assignment (i.e. major form to 2*S*,3*R* and minor form to 2*R*,3*R*), the RMS values for 16 predicted $^3J$ couplings for two diastereomers were 2.68 and 4.66 Hz for PW6B93B and ωB97XD functionals, respectively, and MADs were 1.68 and 2.99 Hz for PW6B93B and ωB97XD functionals, respectively. For 8 predicted $^1$H NMR chemical shifts in two diastereomers, the RMS values were 0.108 and 0.178 ppm for PW6B93B and ωB97XD functionals, respectively, and MADs were 0.099 and 0.161 ppm for PW6B93B and ωB97XD functionals, respectively. These deviations for both *J* couplings and $^1$H chemical shifts are significantly higher than those with the assignment of the major form to the 2*R*,3*R* configuration and the minor form to the 2*S*,3*R* configuration. Thus, the assignment of diastereomers is supported by both the *J* couplings and chemical shifts and agrees with that reported in one of the references.[127]

Alcohol **15** obtained via enzymatic reduction showed only one diastereomer (Figure S7), $^1$H NMR chemical shifts of which correspond to those of the minor form observed in the mixture obtained via chemical reduction (Figure S4). Thus, only 2*S*-alcohol is formed in the enzymatic reduction which is consistent with the previously established stereoselectivity of this enzyme.[143]

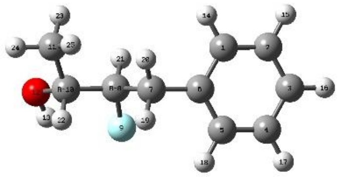

**rr01**

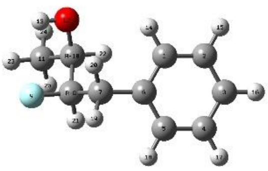

**rr02**

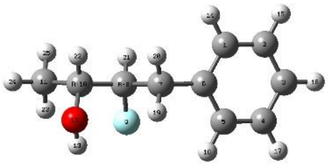

**rr03**

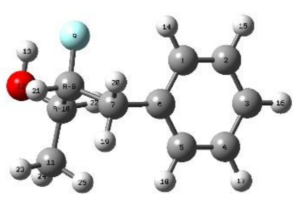

**rr04**

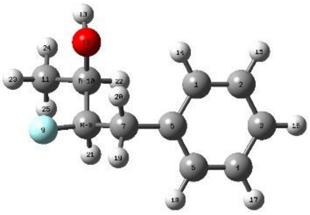

**rr05**

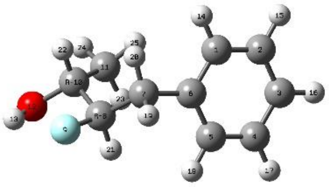

**rr06**

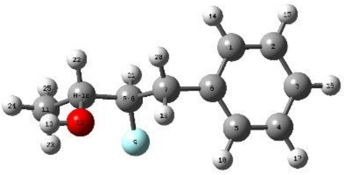

**rr07**

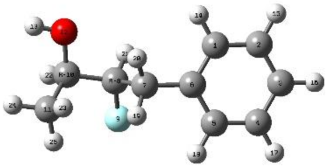

**rr08**

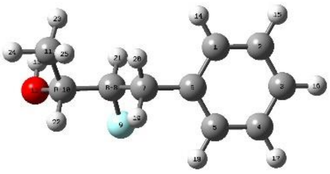

**rr09**

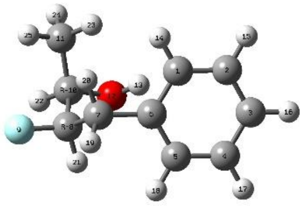

**rr10**

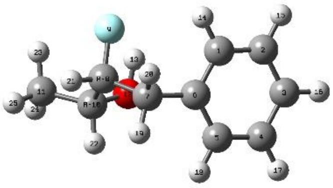

**rr11**

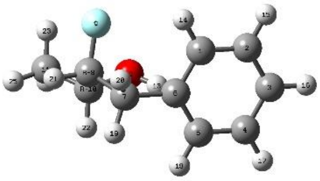

**rr12**

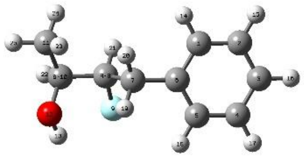

**sr01**

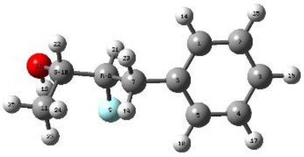

**sr02**

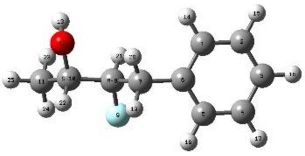

**sr03**

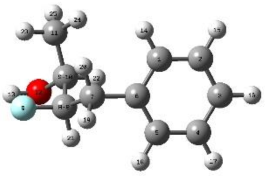

**sr04**

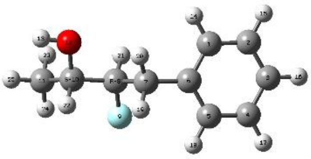

**sr05**

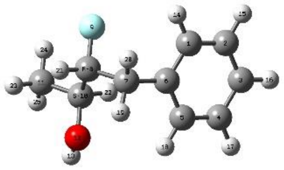

**sr06**

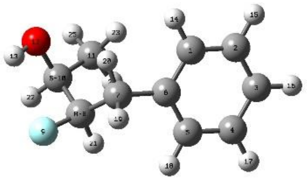

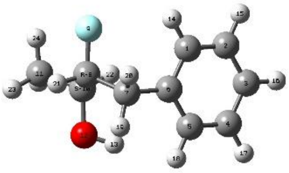

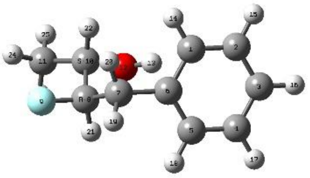

**sr07** **sr08** **sr09**

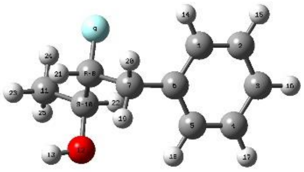

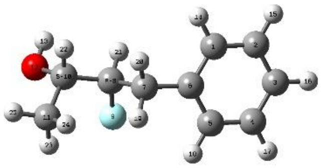

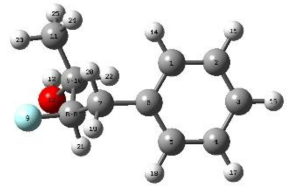

**sr10** **sr11** **sr12**

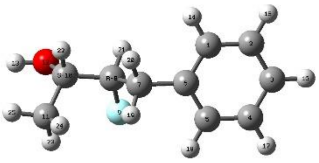

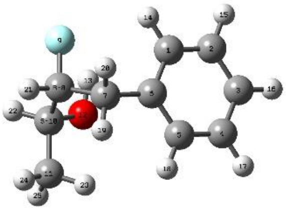

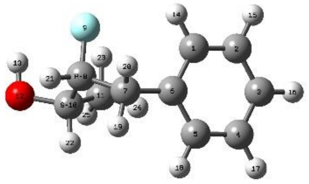

**sr13** **sr14** **sr15**

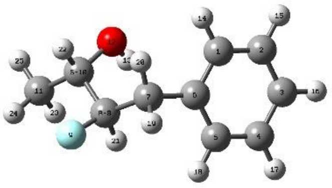

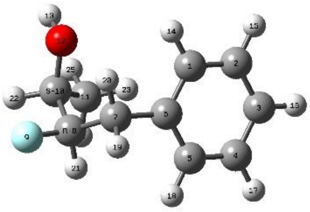

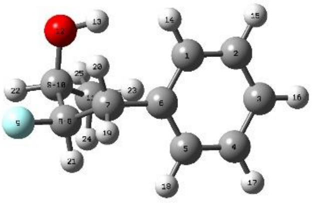

**sr16** **sr17** **sr18**

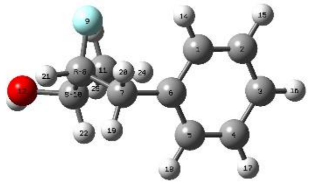

**sr19**

**Figure S6**. The geometries of conformers optimised at the PW6B95D3/def2-TZVP IEFPCM($CHCl_3$) level of theory.

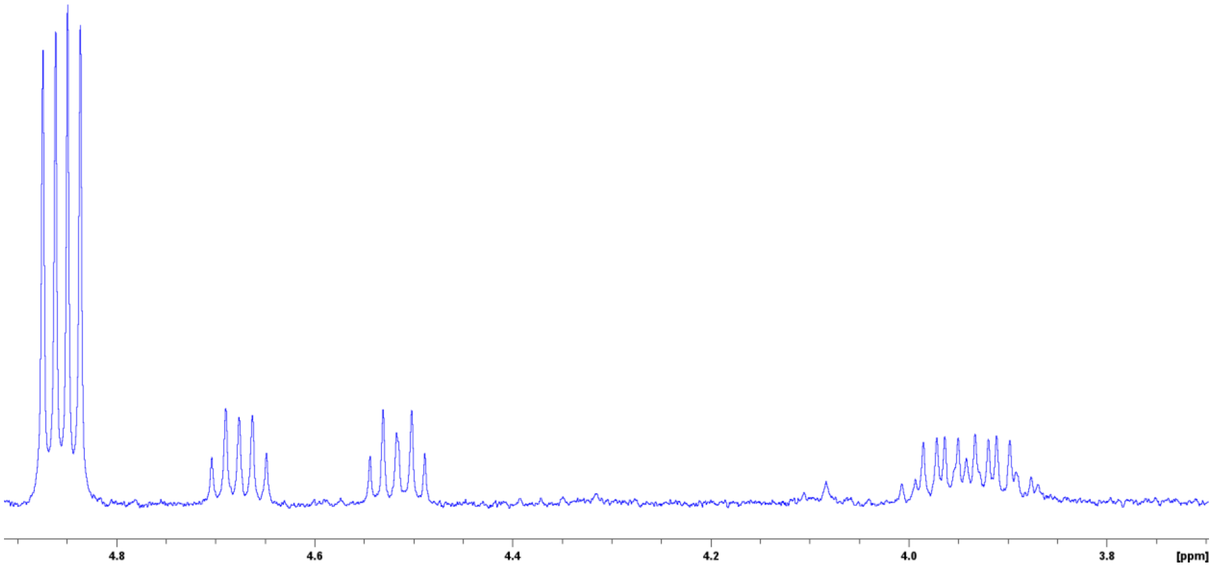


**Figure S7**. $^{1}H$ NMR spectrum showing multiplets due to the 2-CHF proton at 3.94 ppm and the 3-CHF proton at 4.59 ppm of alcohol **15** obtained by enzymatic reduction (in $CDCl_3$, 300 MHz, 298 K). These chemical shifts correspond to the 2*S*-alcohol based on the DFT/NMR analysis above. No signals due to 2R-alcohol (the major form in Table S2) are observed at 3.80 and 4.48 ppm. The doublet of doublets observed at 4.86 ppm is due to the starting material.

**Table S4.** Relative free energies and percentage populations in different conformers of 2*S*,3*R* and 2*R*,3*R* diastereomers from **PW6B95D3**/def2-TZVP IEFPCM($CHCl_3$) calculations. The measured dihedral angles are also shown.

| Conformer | *G*°, hartrees | *ΔG*°, kcal $mol^{-1}$ | Population, % | C5-C4-C3-F, ° | C4-C3-C2-O, ° | C3-C2-O-H, ° |
|---|---|---|---|---|---|---|
| **rr01** | -564.717231 | 0.00 | 26.92 | 60.47 | -174.87 | 51.00 |
| **rr02** | -564.717188 | 0.03 | 25.72 | 175.98 | -54.10 | -62.32 |
| **rr03** | -564.716810 | 0.26 | 17.24 | 62.13 | -57.90 | -61.25 |
| **rr04** | -564.716506 | 0.45 | 12.49 | -69.14 | -176.68 | 53.23 |
| **rr05** | -564.715592 | 1.03 | 4.74 | 174.15 | -50.58 | -174.33 |
| **rr06** | -564.715570 | 1.04 | 4.64 | 159.28 | -174.39 | 51.33 |
| **rr07** | -564.715245 | 1.25 | 3.29 | 64.05 | -50.96 | -172.61 |
| **rr08** | -564.715053 | 1.37 | 2.68 | 68.55 | 68.95 | -179.34 |
| **rr09** | -564.714025 | 2.01 | 0.90 | 57.99 | -177.53 | -68.46 |
| **rr010** | -564.713461 | 2.37 | 0.50 | 176.00 | 78.10 | -70.00 |
| **rr011** | -564.713380 | 2.42 | 0.46 | -63.73 | -68.88 | -44.81 |
| **rr012** | -564.713321 | 2.45 | 0.43 | -67.15 | -70.43 | 57.30 |
| | | | | | | |
| **sr01** | -564.716951 | 0.00 | 24.54 | 60.45 | -59.17 | -56.09 |
| **sr02** | -564.716780 | 0.11 | 20.47 | 67.11 | 178.98 | 57.87 |
| **sr03** | -564.716019 | 0.58 | 9.14 | 65.72 | 61.03 | 78.58 |
| **sr04** | -564.715910 | 0.65 | 8.15 | 177.98 | 177.78 | 58.83 |
| **sr05** | -564.715875 | 0.68 | 7.85 | 64.73 | 61.59 | 174.12 |
| **sr06** | -564.715666 | 0.81 | 6.29 | -64.23 | 57.59 | 176.37 |
| **sr07** | -564.715393 | 0.98 | 4.71 | 157.30 | -58.99 | -55.99 |
| **sr08** | -564.715202 | 1.10 | 3.85 | -73.68 | 54.03 | -79.87 |
| **sr09** | -564.715201 | 1.10 | 3.84 | 178.75 | 79.74 | -66.48 |
| **sr10** | -564.714962 | 1.25 | 2.99 | -63.83 | 55.88 | 76.39 |
| **sr11** | -564.714583 | 1.49 | 2.00 | 65.47 | 175.58 | -57.61 |
| **sr12** | -564.714561 | 1.50 | 1.95 | 174.76 | 170.23 | 178.00 |
| **sr13** | -564.714385 | 1.61 | 1.62 | 64.22 | 170.10 | 173.15 |
| **sr14** | -564.713577 | 2.12 | 0.69 | -63.91 | -65.90 | -43.66 |
| **sr15** | -564.713457 | 2.19 | 0.61 | -66.03 | 161.71 | 68.24 |
| **sr16** | -564.713409 | 2.22 | 0.58 | 153.00 | 48.80 | 64.51 |
| **sr17** | -564.712842 | 2.58 | 0.32 | 174.30 | -38.29 | -175.58 |
| **sr18** | -564.712654 | 2.70 | 0.26 | 178.02 | -39.36 | 83.39 |
| **sr19** | -564.712131 | 3.02 | 0.15 | -69.31 | 148.93 | 171.30 |

**Table S5.** Relative free energies and percentage populations in different conformers of 2*S*,3*R* and 2*R*,3*R* diastereomers from ω**B97XD**/def2-TZVP IEFPCM($CHCl_3$) calculations. The measured dihedral angles are also shown.

| Conformer | *G*°, hartrees | *ΔG*°, kcal $mol^{-1}$ | Population, % | C5-C4-C3-F, ° | C4-C3-C2-O, ° | C3-C2-O-H, ° |
|---|---|---|---|---|---|---|
| **rr01** | -563.834833 | 0.00 | 42.73 | 173.52 | -55.25 | -64.35 |
| **rr02** | -563.834169 | 0.42 | 21.15 | -59.81 | -176.49 | 52.94 |
| **rr03** | -563.833657 | 0.74 | 12.30 | 62.58 | -56.56 | -64.44 |
| **rr04** | -563.833256 | 0.99 | 8.04 | -69.04 | -177.21 | 55.10 |
| **rr05** | -563.832910 | 1.21 | 5.57 | 160.75 | -173.86 | 52.89 |
| **rr06** | -563.832412 | 1.52 | 3.29 | 68.77 | 69.95 | -179.55 |
| **rr07** | -563.832390 | 1.53 | 3.21 | 173.71 | -51.92 | -177.13 |
| **rr08** | -563.831755 | 1.93 | 1.64 | 63.76 | -52.47 | -174.82 |
| **rr09** | -563.830909 | 2.46 | 0.67 | 58.05 | -178.07 | -69.00 |
| **rr010** | -563.830861 | 2.49 | 0.64 | 175.52 | 77.90 | -68.86 |
| **rr011** | -563.830474 | 2.74 | 0.42 | -68.60 | -71.43 | 57.55 |
| **rr012** | -563.830272 | 2.86 | 0.34 | -64.52 | -68.66 | -48.95 |
| | | | | | | |
| **sr01** | -563.833856 | 0.00 | 23.39 | 61.08 | -57.80 | -58.08 |
| **sr02** | -563.833542 | 0.20 | 16.77 | 66.43 | 176.10 | 62.06 |
| **sr03** | -563.833291 | 0.35 | 12.86 | 176.75 | 175.49 | 62.52 |
| **sr04** | -563.832869 | 0.62 | 8.22 | 166.70 | -51.56 | -60.10 |
| **sr05** | -563.832717 | 0.71 | 7.00 | 65.35 | 60.81 | 76.15 |
| **sr06** | -563.832714 | 0.72 | 6.98 | 64.61 | 61.61 | 175.32 |
| **sr07** | -563.832376 | 0.93 | 4.88 | -64.52 | 58.32 | 177.78 |
| **sr08** | -563.832226 | 1.02 | 4.16 | 46.91 | 54.99 | -82.45 |
| **sr09** | -563.832054 | 1.13 | 3.47 | -63.95 | 56.44 | 75.37 |
| **sr10** | -563.831817 | 1.28 | 2.70 | 64.60 | 169.94 | 173.32 |
| **sr11** | -563.831691 | 1.36 | 2.36 | 174.20 | 169.90 | 179.51 |
| **sr12** | -563.831568 | 1.44 | 2.07 | 177.23 | 77.83 | -64.98 |
| **sr13** | -563.831363 | 1.56 | 1.67 | 65.78 | 175.56 | -58.11 |
| **sr14** | -563.830712 | 1.97 | 0.84 | -64.27 | -64.79 | -46.87 |
| **sr15** | -563.830553 | 2.07 | 0.71 | 172.33 | -42.45 | -174.45 |
| **sr16** | -563.830460 | 2.13 | 0.64 | -68.37 | 156.82 | 71.74 |
| **sr17** | -563.830353 | 2.20 | 0.57 | 176.98 | -42.38 | 84.56 |
| **sr18** | -563.830225 | 2.28 | 0.50 | 154.46 | 50.43 | 63.88 |
| **sr19** | -563.829451 | 2.76 | 0.22 | -69.58 | 151.82 | 171.48 |

**Table S6.** Calculated and experimental spin-spin *J* couplings of 2*S*,3*R* and 2*R*,3*R* diastereomers. The optimised geometries from **PW6B95D3**/def2-TZVP IEFPCM($CHCl_3$) calculations were used for calculating *J* couplings at the B3LYP/6-311+G(2d,p) IEFPCM($CHCl_3$) level of theory. The averaged values were calculated using $J_{av} = \sum J_i p_i$, where $J_i$ is the *J* coupling value in conformer *i* and $p_i$ is the population of conformer *i* (percentage population divided by 100, Table S4).

| Conformer | $^3J$(H2,H3), Hz | $^3J$(H3,4'), Hz | $^3J$(H3,H4), Hz | $^3J$(H2,F), Hz | $^3J$(H4,F), Hz | $^3J$(H4',F), Hz | $^3J$(C5,F), Hz | $^3J$(C1,F), Hz |
|---|---|---|---|---|---|---|---|---|
| **rr01** | 6.73 | 1.76 | 9.87 | 10.20 | 12.82 | 39.35 | 0.43 | 4.07 |
| **rr02** | 0.77 | 1.64 | 9.94 | 19.45 | 14.77 | 39.66 | 0.06 | 4.20 |
| **rr03** | 1.05 | 10.03 | 4.73 | 22.47 | 9.07 | 6.63 | 14.07 | 5.60 |
| **rr04** | 7.24 | 4.02 | 2.95 | 9.95 | 42.83 | 16.58 | -1.21 | 4.46 |
| **rr05** | 1.88 | 10.19 | 4.75 | 24.15 | 9.35 | 5.60 | 13.60 | 7.31 |
| **rr06** | 6.65 | 10.17 | 2.33 | 11.81 | 22.41 | -1.10 | 12.91 | 4.87 |
| **rr07** | 1.92 | 1.35 | 9.77 | 22.21 | 16.18 | 38.27 | -0.33 | 6.90 |
| **rr08** | 4.33 | 1.08 | 9.51 | 8.14 | 18.70 | 40.99 | -0.62 | -0.83 |
| **rr09** | 6.80 | 2.09 | 9.90 | 11.54 | 10.87 | 35.91 | 0.87 | 3.85 |
| **rr010** | 3.41 | 10.39 | 4.87 | 10.50 | 9.13 | 6.20 | 13.40 | -1.50 |
| **rr011** | 0.32 | 5.01 | 2.45 | 18.38 | 43.07 | 12.11 | -0.36 | 2.03 |
| **rr012** | 0.44 | 4.61 | 2.93 | 19.70 | 40.00 | 13.11 | -0.93 | 2.29 |
| **Calc. $J_{av}$:** | **3.75** | **4.27** | **7.45** | **15.83** | **17.19** | **27.05** | **3.69** | **4.52** |
| **Exp. $J$:[a]** | **4.68** | **4.69** | **8.13** | **18.30** | **17.75** | **30.14** | **4.54** | **5.35** |
| | | | | | | | | |
| **sr01** | 2.94 | 1.85 | 10.09 | 12.65 | 12.74 | 40.51 | 0.54 | 5.09 |
| **sr02** | 2.62 | 1.01 | 9.64 | 20.45 | 18.68 | 39.19 | -0.84 | 5.09 |
| **sr03** | 7.60 | 1.30 | 9.15 | -0.31 | 17.20 | 40.42 | -0.35 | 2.11 |
| **sr04** | 2.41 | 10.48 | 5.66 | 22.00 | 6.22 | 8.09 | 13.16 | 5.61 |
| **sr05** | 7.15 | 1.42 | 9.22 | 1.51 | 16.69 | 41.23 | -0.19 | 2.88 |
| **sr06** | 7.59 | 4.41 | 2.51 | 3.03 | 42.06 | 13.38 | 0.33 | 1.71 |
| **sr07** | 2.90 | 10.01 | 1.94 | 13.27 | 22.62 | -1.50 | 13.08 | 5.48 |
| **sr08** | 7.65 | 2.99 | 3.71 | 4.75 | 40.86 | 20.04 | -1.65 | -0.59 |
| **sr09** | 6.73 | 9.53 | 4.92 | -2.09 | 7.90 | 9.52 | 13.51 | 6.38 |
| **sr10** | 8.02 | 4.50 | 2.45 | 1.12 | 40.82 | 12.49 | 0.37 | 0.94 |
| **sr11** | 1.89 | 1.15 | 9.54 | 20.02 | 16.84 | 36.29 | -0.52 | 5.88 |
| **sr12** | 1.47 | 10.67 | 5.29 | 22.81 | 8.82 | 6.07 | 12.51 | 7.48 |
| **sr13** | 1.51 | 1.28 | 10.07 | 20.34 | 15.97 | 36.32 | -0.34 | 6.94 |
| **sr14** | 3.63 | 5.03 | 2.41 | 9.51 | 43.93 | 12.95 | -0.92 | 4.87 |
| **sr15** | 1.13 | 5.37 | 2.67 | 23.11 | 42.02 | 12.93 | -0.78 | 7.61 |
| **sr16** | 7.25 | 9.07 | 1.25 | 8.27 | 26.15 | -1.96 | 10.86 | -0.67 |
| **sr17** | 0.79 | 10.14 | 4.92 | 27.24 | 9.71 | 6.42 | 14.73 | 10.19 |
| **sr18** | 0.58 | 9.96 | 5.40 | 27.85 | 6.93 | 8.72 | 14.44 | 9.47 |
| **sr19** | 0.13 | 4.43 | 3.25 | 20.61 | 39.79 | 14.19 | -0.65 | 12.98 |
| **Calc. $J_{av}$:** | **4.28** | **3.55** | **7.66** | **11.74** | **18.73** | **29.49** | **2.45** | **4.31** |
| **Exp. $J$:[b]** | **4.06** | **3.62** | **8.89** | **15.64** | **18.31** | **34.61** | **2.76** | **5.83** |

[a] Experimental values measured for the major form.

[b] Experimental values measured for the minor form.

**Table S7.** Calculated and experimental spin-spin *J* couplings of 2*S*,3*R* and 2*R*,3*R* diastereomers. The optimised geometries from ωB97XD/def2-TZVP IEFPCM($CHCl_3$) calculations were used for calculating *J* couplings at the B3LYP/6-311+G(2d,p) IEFPCM($CHCl_3$) level of theory. The averaged values were calculated using $J_{av} = \sum J_i p_i$, where $J_i$ is the *J* coupling value in conformer *i* and $p_i$ is the population of conformer *i* (percentage population divided by 100, Table S5).

| Conformer | $^3J$(H2,H3), Hz | $^3J$(H3,4'), Hz | $^3J$(H3,H4), Hz | $^3J$(H2,F), Hz | $^3J$(H4,F), Hz | $^3J$(H4',F), Hz | $^3J$(C5,F), Hz | $^3J$(C1,F), Hz |
|---|---|---|---|---|---|---|---|---|
| **rr01** | 0.99 | 10.17 | 4.45 | 23.06 | 9.79 | 4.92 | 13.68 | 4.67 |
| **rr02** | 0.95 | 1.63 | 9.96 | 20.83 | 13.53 | 39.13 | 0.39 | 3.95 |
| **rr03** | 6.69 | 1.90 | 9.98 | 10.38 | 10.94 | 38.73 | 1.03 | 4.27 |
| **rr04** | 7.30 | 4.03 | 2.91 | 9.33 | 42.25 | 15.88 | -1.09 | 4.46 |
| **rr05** | 6.72 | 10.16 | 2.53 | 10.80 | 21.03 | -0.63 | 13.07 | 4.76 |
| **rr06** | 4.12 | 1.09 | 9.54 | 8.61 | 17.56 | 40.76 | -0.43 | -1.08 |
| **rr07** | 1.78 | 10.24 | 4.73 | 24.76 | 9.09 | 5.18 | 13.33 | 6.32 |
| **rr08** | 1.80 | 1.44 | 9.87 | 22.64 | 14.22 | 37.74 | 0.12 | 5.84 |
| **rr09** | 6.81 | 2.13 | 9.95 | 11.24 | 9.68 | 35.74 | 1.32 | 3.91 |
| **rr010** | 3.37 | 10.41 | 4.78 | 10.09 | 8.99 | 5.83 | 13.22 | -1.56 |
| **rr011** | 0.41 | 4.32 | 3.11 | 19.95 | 39.52 | 13.09 | -0.88 | 1.77 |
| **rr012** | 0.37 | 4.91 | 2.53 | 19.34 | 42.42 | 11.61 | -0.36 | 1.84 |
| **Calc. $J_{av}$:** | **2.70** | **6.32** | **6.35** | **18.63** | **14.50** | **18.88** | **7.20** | **4.27** |
| **Exp. *J*:[a]** | **4.68** | **4.69** | **8.13** | **18.30** | **17.75** | **30.14** | **4.54** | **5.35** |
| | | | | | | | | |
| **sr01** | 2.67 | 1.81 | 10.12 | 13.17 | 11.82 | 40.24 | 0.81 | 5.56 |
| **sr02** | 2.40 | 1.12 | 9.76 | 21.38 | 16.40 | 38.38 | -0.36 | 4.89 |
| **sr03** | 2.22 | 10.52 | 5.49 | 23.12 | 6.83 | 7.25 | 13.01 | 5.29 |
| **sr04** | 1.97 | 10.38 | 3.45 | 18.46 | 15.45 | 1.73 | 14.26 | 7.04 |
| **sr05** | 7.62 | 1.39 | 9.24 | -0.42 | 15.37 | 40.03 | 0.15 | 1.70 |
| **sr06** | 7.24 | 1.48 | 9.28 | 1.26 | 15.06 | 40.89 | 0.30 | 2.56 |
| **sr07** | 7.68 | 4.36 | 2.51 | 2.26 | 41.87 | 12.96 | 0.37 | 1.74 |
| **sr08** | 7.73 | 2.96 | 3.71 | 3.64 | 40.60 | 19.72 | -1.57 | -0.56 |
| **sr09** | 8.05 | 4.47 | 2.43 | 0.51 | 40.64 | 12.06 | 0.36 | 0.87 |
| **sr10** | 1.56 | 1.30 | 10.04 | 20.86 | 14.62 | 36.01 | 0.03 | 6.11 |
| **sr11** | 1.46 | 10.67 | 5.19 | 23.46 | 8.81 | 5.65 | 12.37 | 6.78 |
| **sr12** | 6.82 | 9.58 | 4.67 | -2.14 | 8.71 | 8.23 | 13.39 | 5.37 |
| **sr13** | 1.96 | 1.15 | 9.52 | 20.51 | 15.81 | 36.01 | -0.29 | 5.09 |
| **sr14** | 3.36 | 4.98 | 2.43 | 10.17 | 43.22 | 12.29 | -0.81 | 5.36 |
| **sr15** | 1.12 | 10.27 | 4.62 | 24.17 | 10.61 | 4.97 | 14.41 | 9.52 |
| **sr16** | 0.77 | 4.86 | 3.00 | 23.67 | 41.08 | 13.37 | -0.75 | 7.66 |
| **sr17** | 0.76 | 10.05 | 5.28 | 25.80 | 7.34 | 7.84 | 14.21 | 9.11 |
| **sr18** | 7.28 | 9.25 | 1.43 | 6.61 | 25.02 | -1.88 | 11.14 | -0.60 |
| **sr19** | 0.26 | 4.48 | 3.24 | 21.71 | 39.49 | 13.65 | -0.70 | 10.76 |
| **Calc. $J_{av}$:** | **3.82** | **4.27** | **7.41** | **13.54** | **16.93** | **26.62** | **3.76** | **4.52** |
| **Exp. *J*:[b]** | **4.06** | **3.62** | **8.89** | **15.64** | **18.31** | **34.61** | **2.76** | **5.83** |

[a] Experimental values measured for the major form.

[b] Experimental values measured for the minor form.

**Table S8.** Calculated $^1$H NMR chemical shifts at the B3LYP/6-311+G(2d,p) IEFPCM($CHCl_3$) level of theory and calculated $^{19}$F NMR chemical shifts at the ωB97XD/aug-cc-pVDZ CPCM($CHCl_3$) level of theory. The optimised geometries from **PW6B95D3**/def2-TZVP IEFPCM($CHCl_3$) calculations were used for calculating NMR chemical shifts. The averaged values were calculated using $\delta_{av} = \sum \delta_i p_i$, where $\delta_i$ is the chemical shift value in conformer *i* and $p_i$ is the population of conformer *i* (percentage population divided by 100, Table S4). Calculated $^{19}$F chemical shifts were further corrected using equation (4):[134] $\delta_{pred}=(\delta_{calc}+0.02534)/1.0112$.

| Conformer | δ (H2), ppm | δ (H3), ppm | δ (H4), ppm | δ (H4'), ppm | δ (F), ppm |
|---|---|---|---|---|---|
| **rr01** | 4.03 | 4.21 | 2.81 | 2.90 | -201.26 |
| **rr02** | 3.62 | 4.56 | 3.07 | 2.76 | -192.90 |
| **rr03** | 3.76 | 4.46 | 3.55 | 2.76 | -211.38 |
| **rr04** | 3.35 | 4.62 | 2.83 | 2.76 | -189.98 |
| **rr05** | 3.74 | 4.47 | 3.07 | 2.76 | -178.24 |
| **rr06** | 3.97 | 4.77 | 3.55 | 2.76 | -194.45 |
| **rr07** | 4.15 | 4.34 | 3.54 | 2.76 | -198.90 |
| **rr08** | 4.40 | 4.68 | 2.86 | 2.76 | -208.77 |
| **rr09** | 3.76 | 4.03 | 2.79 | 2.76 | -190.03 |
| **rr010** | 3.95 | 5.17 | 3.42 | 2.76 | -177.56 |
| **rr011** | 3.88 | 4.90 | 2.80 | 2.76 | -207.56 |
| **rr012** | 4.18 | 4.76 | 2.83 | 2.76 | -209.66 |
| **Calc. $\delta_{av}$:** | **3.79** | **4.46** | **3.08** | **2.80** | **-198.004** |
| **Exp. δ:[a]** | **3.80** | **4.48** | **2.99** | **3.00** | **-192.99** |
| | | | | | |
| **sr01** | 4.42 | 4.48 | 3.26 | 2.78 | -190.04 |
| **sr02** | 3.80 | 4.71 | 2.90 | 2.66 | -211.37 |
| **sr03** | 3.63 | 3.91 | 2.79 | 3.60 | -203.37 |
| **sr04** | 3.17 | 5.10 | 3.47 | 2.76 | -175.03 |
| **sr05** | 4.00 | 4.10 | 2.78 | 3.52 | -189.18 |
| **sr06** | 3.47 | 4.53 | 3.38 | 3.20 | -194.54 |
| **sr07** | 4.41 | 5.06 | 3.14 | 3.59 | -195.11 |
| **sr08** | 3.23 | 4.56 | 3.11 | 3.49 | -186.35 |
| **sr09** | 4.09 | 4.41 | 3.59 | 2.82 | -191.18 |
| **sr10** | 3.32 | 4.38 | 3.49 | 3.18 | -208.75 |
| **sr11** | 4.11 | 4.45 | 2.88 | 2.67 | -199.01 |
| **sr12** | 3.68 | 5.03 | 3.35 | 2.80 | -175.62 |
| **sr13** | 4.14 | 4.73 | 2.91 | 2.65 | -208.87 |
| **sr14** | 4.40 | 4.91 | 2.98 | 3.46 | -187.80 |
| **sr15** | 3.66 | 5.26 | 2.79 | 3.65 | -167.50 |
| **sr16** | 3.72 | 4.40 | 3.38 | 3.19 | -206.65 |
| **sr17** | 4.43 | 4.83 | 3.22 | 3.53 | -168.58 |
| **sr18** | 4.07 | 4.90 | 3.41 | 3.31 | -173.48 |
| **sr19** | 4.10 | 5.17 | 2.81 | 3.46 | -202.33 |
| **Calc. $\delta_{av}$:** | **3.90** | **4.55** | **3.12** | **3.00** | **-195.37** |
| **Exp. δ:[b]** | **3.93** | **4.59** | **3.00** | **2.93** | **-190.38** |

[a] Experimental values measured for the major form.

[b] Experimental values measured for the minor form.

**Table S9.** Calculated $^{1}$H NMR chemical shifts at the B3LYP/6-311+G(2d,p) IEFPCM($CHCl_3$) level of theory and calculated $^{19}$F NMR chemical shifts at the ωB97XD/aug-cc-pVDZ CPCM($CHCl_3$) level of theory. The optimised geometries from ω**B97XD**/def2-TZVP IEFPCM($CHCl_3$) calculations were used for calculating NMR chemical shifts. The averaged values were calculated using $\delta_{av} = \sum \delta_i p_i$, where $\delta_i$ is the chemical shift value in conformer *i* and $p_i$ is the population of conformer *i* (percentage population divided by 100, Table S5). Calculated $^{19}$F chemical shifts were further corrected using equation (4):[134] $\delta_{pred}=(\delta_{calc}+0.02534)/1.0112$.

| Conformer | δ (H2), ppm | δ (H3), ppm | δ (H4), ppm | δ (H4'), ppm | δ (F), ppm |
|---|---|---|---|---|---|
| **rr01** | 3.48 | 4.55 | 3.09 | 3.42 | -201.98 |
| **rr02** | 4.15 | 4.44 | 2.82 | 2.96 | -194.66 |
| **rr03** | 3.90 | 4.65 | 3.53 | 2.78 | -212.98 |
| **rr04** | 3.51 | 4.72 | 2.95 | 3.46 | -191.38 |
| **rr05** | 4.09 | 4.82 | 3.65 | 2.78 | -179.41 |
| **rr06** | 4.51 | 4.86 | 2.89 | 3.10 | -195.28 |
| **rr07** | 3.79 | 4.63 | 3.23 | 3.49 | -199.72 |
| **rr08** | 4.28 | 4.57 | 3.54 | 2.81 | -210.71 |
| **rr09** | 3.87 | 4.25 | 2.81 | 2.90 | -191.60 |
| **rr010** | 4.07 | 5.24 | 3.51 | 3.04 | -177.85 |
| **rr011** | 4.32 | 4.85 | 2.97 | 3.52 | -208.61 |
| **rr012** | 4.01 | 4.98 | 2.93 | 3.54 | -211.14 |
| **Calc. $\delta_{av}$:** | **3.78** | **4.59** | **3.11** | **3.19** | **-199.36** |
| **Exp. δ:[a]** | **3.80** | **4.48** | **2.99** | **3.00** | **-192.99** |
| | | | | | |
| **sr01** | 4.40 | 4.53 | 3.11 | 2.86 | -191.36 |
| **sr02** | 3.91 | 4.93 | 2.90 | 2.88 | -213.12 |
| **sr03** | 3.32 | 5.17 | 3.54 | 2.89 | -203.12 |
| **sr04** | 4.52 | 5.08 | 3.33 | 3.62 | -174.25 |
| **sr05** | 3.74 | 4.13 | 2.81 | 3.78 | -191.00 |
| **sr06** | 4.11 | 4.34 | 2.79 | 3.72 | -196.17 |
| **sr07** | 3.68 | 4.64 | 3.49 | 3.31 | -196.18 |
| **sr08** | 3.37 | 4.67 | 3.22 | 3.58 | -186.84 |
| **sr09** | 3.50 | 4.49 | 3.59 | 3.29 | -191.82 |
| **sr10** | 4.28 | 4.97 | 2.91 | 2.88 | -210.58 |
| **sr11** | 3.80 | 5.15 | 3.46 | 2.92 | -199.69 |
| **sr12** | 4.21 | 4.53 | 3.70 | 2.95 | -177.08 |
| **sr13** | 4.24 | 4.64 | 2.91 | 2.85 | -210.33 |
| **sr14** | 4.52 | 4.96 | 3.11 | 3.54 | -188.97 |
| **sr15** | 4.55 | 4.97 | 3.35 | 3.62 | -169.65 |
| **sr16** | 3.79 | 5.35 | 2.95 | 3.67 | -207.18 |
| **sr17** | 4.20 | 4.99 | 3.52 | 3.38 | -170.12 |
| **sr18** | 3.85 | 4.50 | 3.51 | 3.28 | -174.56 |
| **sr19** | 4.22 | 5.27 | 2.94 | 3.60 | -204.31 |
| **Calc. $\delta_{av}$:** | **3.98** | **4.74** | **3.16** | **3.15** | **-195.98** |
| **Exp. δ:[b]** | **3.93** | **4.59** | **3.00** | **2.93** | **-190.38** |

[a] Experimental values measured for the major form.

[b] Experimental values measured for the minor form.

**Table S10.** Calculated $^{1}H$ and $^{19}F$ reference shielding constants ($\sigma_H$ and $\sigma_F$, ppm) of $Si(CH_3)_4$ and $CFCl_3$. DFT functionals, basis sets and solvation models used in geometry optimisations and NMR calculations are also shown.

| | Geometry Optimisation | NMR GIAO | $\sigma_H$, ppm | $\sigma_F$, ppm |
|---|---|---|---|---|
| $Si(CH_3)_4$ | PW6B95D3<br>def2-TZVP<br>IEFPCM(CHCl3) | B3LYP<br>6-311+G(2d,p)<br>IEFPCM(CHCl3) | 32.1288 | - |
| $Si(CH_3)_4$ | ωB97XD<br>def2-TZVP<br>IEFPCM(CHCl3) | B3LYP<br>6-311+G(2d,p)<br>IEFPCM(CHCl3) | 31.9880 | - |
| $CFCl_3$ | PW6B95D3<br>def2-TZVP<br>IEFPCM(CHCl3) | ωB97XD<br>aug-cc-pVDZ<br>CPCM($CHCl_3$) | - | 203.1195 |
| $CFCl_3$ | ωB97XD<br>def2-TZVP<br>IEFPCM(CHCl3) | ωB97XD<br>aug-cc-pVDZ<br>CPCM($CHCl_3$) | - | 202.3381 |

**Table S11.** Error function (RMS and MAD) values for two alternative assignment of diastereomer configurations. Smaller values of error functions correspond to better agreement between experimentally measured and DFT predicted values of NMR *J* couplings and chemical shifts.

| DFT Method for geometry optimisations and population predictions | Error Function | Error function value for assignment: Major=*R*,*R* Minor=*S*,*R* | Error function value for assignment: Major=*S*,*R* Minor=*R*,*R* |
|---|---|---|---|
| PW6B95D3/def2-TZVP IEFPCM($CHCl_3$) | RMS($^{3}J_{HH}$,$^{3}J_{HF}$,$^{3}J_{CF}$) / Hz, 16 values | 2.01 | 2.68 |
| | RMS($^{3}J_{HH}$) / Hz, 6 values | 0.72 | 0.85 |
| | RMS($\delta_H$) / ppm, 8 values | 0.096 | 0.108 |
| | MAD($^{3}J_{HH}$,$^{3}J_{HF}$,$^{3}J_{CF}$) / Hz, 16 values | 1.41 | 1.68 |
| | MAD($^{3}J_{HH}$) / Hz, 6 values | 0.59 | 0.74 |
| | MAD($\delta_H$) / ppm, 8 values | 0.074 | 0.099 |
| ωB97XD/def2-TZVP IEFPCM($CHCl_3$) | RMS($^{3}J_{HH}$,$^{3}J_{HF}$,$^{3}J_{CF}$) / Hz , 16 values | 3.80 | 4.66 |
| | RMS($^{3}J_{HH}$) / Hz, 6 values | 1.44 | 1.68 |
| | RMS($\delta_H$) / ppm, 8 values | 0.142 | 0.178 |
| | MAD($^{3}J_{HH}$,$^{3}J_{HF}$,$^{3}J_{CF}$) / Hz, 16 values | 2.51 | 2.99 |
| | MAD($^{3}J_{HH}$) / Hz, 6 values | 1.29 | 1.68 |
| | MAD($\delta_H$) / ppm, 8 values | 0.128 | 0.161 |

# Docking Studies

## Preparing your protein

The crystal structure of NCR was downloaded from the PBD database (4A3U).[107] The pQR1445 structure was predicted by AlphaFold2,[145] and the highest rank was chosen for docking experiments. Using Chimera (UCFS Chimera version 1.13.1),[146] the protein PDB was loaded and any water molecules and other subunits deleted. Selenomethionine residues were mutated to methionine and selenocysteine to cysteine. The protein was then saved as a pdb file. The protein pdb file was opened using AutoDock Tools (version 1.5.6)[147] and hydrogens added to the protein. The box size for ligand docking was set to cover the whole protein (to encompass the active site, but not bias the results) and saved as a pdbqt file.

## Preparing the ligand

ChemDraw smiles were generated and the energy of the ligand minimised using Avogadro (version 1.2.0).[148] Again, the ligand pdb file was converted into a pdbqt file via AutoDock Tools.

## Using Autodock Vina

The docking was performed via Vina (v.1.2.0)[60,61] through a terminal window. Vina was run and the binding modes results and energies were saved in a log file (log.txt).

## Viewing the results

Docking results (binding modes) were viewed using Chimera by loading in the receptor pdb file and then out.pdb (all the output files). Nine possible binding modes were generated and ranked according to the affinity free energy. The scoring function used in Vina was derived using the PDBbind data set, in which the receptors were treated as rigid components, and the ligands as flexible molecules with the number of active rotatable bonds ranging from 0 to 32. Vina uses a gradient optimization method in its local optimization procedure. The calculation of the gradient gives the optimization algorithm a ”sense of direction” from a single evaluation. By using multithreading, Vina can further speed up the execution by taking advantage of multiple CPUs or CPU cores.

**Table S12** Global docking analysis for NCR with compounds (*E/Z*)-**1a,** (*E/Z*)-**5a** and (*E/Z*)-**6a**, and forNCR/pQR1445 with (*E/Z*)-**2a**

| Enzyme | Affinity (kcal/mol) | Ranking[b] | In catalytic pot | Orentation state[c] | Distance 1[d] | Distance 2[e] |
|---|---|---|---|---|---|---|
| NCR (4A3U) with (*E*)-**1a** | -7.0 | 1 | Folded | No | - | - |
| | -6.1 | 2 | Folded | No | - | - |
| | -5.9 | 3 | Folded | No | - | - |
| | -5.5 | 4 | Folded | No | - | - |
| | -5.4 | 5 | Folded | Yes | 5.0 Å | 3.5 Å |
| | -5.3 | 6 | Unfolded | No | - | - |
| | -5.1 | 7 | Unfolded | No | - | - |
| | -4.8 | 8 | Unfolded | - | - | - |
| | -4.7 | 9 | Unfolded | - | - | - |
| NCR (4A3U) with (*Z*)-**1a** | -5.9 | 1 | Folded | No | - | - |
| | -5.8 | 2 | Folded | No | - | - |
| | -5.5 | 3 | Folded | No | - | - |
| | -5.3 | 4 | Folded | No | - | - |
| | -5.3 | 5 | Folded | Yes | 6.2 Å | 3.5 Å |
| | -5.2 | 6 | Folded | Yes | 6.5 Å | 3.5 Å |
| | -4.7 | 7 | Unfolded | - | - | - |
| | -4.6 | 8 | Folded | No | - | - |
| | -4.6 | 9 | Folded | No | - | - |
| NCR (4A3U) with (*E*)-**5a** | -6.2 | 1 | Folded | Yes | 5.0 Å | 3.9 Å |
| | -5.9 | 2 | Folded | Yes | 7.5 Å | 4.8 Å |
| | -5.9 | 3 | Folded | No | - | - |
| | -5.6 | 4 | Folded | No | - | - |
| | -5.5 | 5 | Folded | No | - | - |
| | -5.5 | 6 | Folded | No | - | - |
| | -5.0 | 7 | Folded | No | - | - |
| | -4.9 | 8 | Folded | No | - | - |
| | -4.9 | 9 | Folded | No | - | - |
| NCR (4A3U) with (*Z*)-**5a** | -6.1 | 1 | Folded | Yes | 6.2 Å | 3.5 Å |
| | -6.0 | 2 | Folded | No | - | - |
| | -5.8 | 3 | Folded | No | - | - |
| | -5.6 | 4 | Folded | No | - | - |
| | -5.5 | 5 | Folded | No | - | - |
| | -.5.4 | 6 | Folded | No | - | - |
| | -5.3 | 7 | Unfolded | - | - | - |
| | -5.2 | 8 | Unfolded | - | - | - |
| | -4.9 | 9 | Unfolded | - | - | - |
| NCR (4A3U) with (*E*)-**6a** | -6.7 | 1 | Folded | No | - | - |
| | -6.1 | 2 | Folded | No | - | - |
| | -5.2 | 3 | Folded | No | - | - |
| | -5.0 | 4 | Folded | No | - | - |
| | -5.0 | 5 | Folded | Yes | 4.8 Å | 3.5 Å |
| | -4.8 | 6 | Folded | No | - | - |
| | -4.8 | 7 | Unfolded | - | - | - |
| | -4.6 | 8 | Unfolded | - | - | - |
| | -4.6 | 9 | Unfolded | - | - | - |
| NCR (4A3U) with (*Z*)-**6a** | -5.9 | 1 | Folded | No | - | - |
| | -5.8 | 2 | Folded | No | - | - |
| | -5.5 | 3 | Folded | No | - | - |
| | -5.5 | 4 | Folded | Yes | 6.4 Å | 3.8 Å |
| | -5.5 | 5 | Folded | No | - | - |
| | -5.3 | 6 | Folded | No | - | - |
| | -5.2 | 7 | Unfolded | - | - | - |
| | -5.0 | 8 | Unfolded | - | - | - |
| | -4.8 | 9 | Unfolded | - | - | - |
| NCR (4A3U) with (*E*)-**2a** | -5.5 | 1 | Folded | No | - | - |
| | -5.4 | 2 | Folded | Yes | 5.6 Å | 4.9 Å |

| | | | | | | |
|---|---|---|---|---|---|---|
| | -5.2 | 3 | Folded | No | - | - |
| | -5.1 | 4 | Folded | No | - | - |
| | -5.0 | 5 | Folded | No | - | - |
| | -5.0 | 6 | Folded | No | - | - |
| | -4.9 | 7 | Folded | No | - | - |
| | -4.8 | 8 | Folded | No | - | - |
| | -4.7 | 9 | Folded | No | - | - |
| NCR (4A3U) with (*Z*)-**2a** | -5.6 | 1 | Folded | Yes | 5.4 Å | 5.0 Å |
| | -5.4 | 2 | Folded | No | - | - |
| | -5.1 | 3 | Folded | Yes | - | - |
| | -5.0 | 4 | Folded | No | - | - |
| | -5.0 | 5 | Folded | No | - | - |
| | -4.5 | 6 | Folded | No | - | - |
| | -4.8 | 7 | Folded | No | - | - |
| | -4.6 | 8 | Folded | No | - | - |
| | -4.5 | 9 | Folded | No | - | - |
| pQR1445 (generated with AlphaFold 2) with (*E*)-**2a** | -6.1 | 1 | Folded | Yes | 5.4 Å | 4.2 Å |
| | -5.6 | 2 | Unfolded | No | - | - |
| | -5.1 | 3 | Folded | Yes | 3.8 Å | 4.5 Å |
| | -5.0 | 4 | Unfolded | No | - | - |
| | -5.0 | 5 | Unfolded | No | - | - |
| | -4.6 | 6 | Unfolded | No | - | - |
| | -4.6 | 7 | Unfolded | No | - | - |
| | -4.5 | 8 | Unfolded | No | - | - |
| | -4.4 | 9 | Unfolded | No | - | - |
| pQR1445 (generated with AlphaFold 2) with (Z)-**2a** | -6.1 | 1 | Folded | Yes | 5.3 Å | 4.4 Å |
| | -5.7 | 2 | Unfolded | No | - | - |
| | -5.2 | 3 | Folded | Yes | 3.8 Å | 3.6 Å |
| | -4.8 | 4 | Unfolded | No | - | - |
| | -4.7 | 5 | Unfolded | No | - | - |
| | -4.6 | 6 | Unfolded | No | - | - |
| | -4.5 | 7 | Unfolded | No | - | - |
| | -4.5 | 8 | Unfolded | No | - | - |
| | -4.4 | 9 | Unfolded | No | - | - |

a. The crystal structure of *Zm*NCR (PDB code: 4A3U) is obtained via PBD database.

b. The ranking order followed the affinity energy.

c. Orientation state (see below): If Yes, the carbonyl group on the ligand binds to His172 and/or Asn175, meanwhile the distance betweem FNM hydride to *β*-C of ligand and Tyr177 to *α*-C of ligand should be no more than 7 angstrom (Å). Otherwise, the ligand is not in a suitable orientation state.

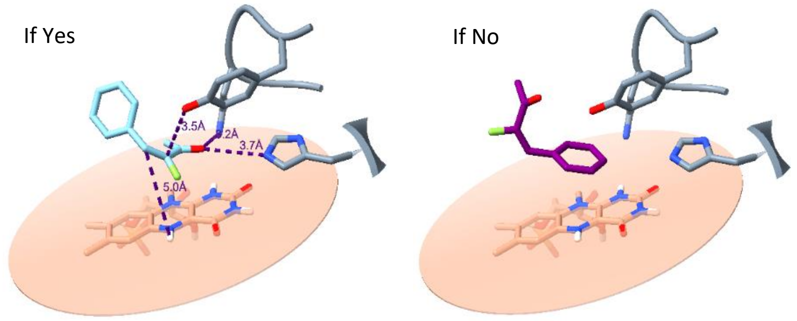


d. Distance 1: the FNM hydride to *β*-C of ligands.

e. Distance 2: Tyr177 to *α*-C of ligands.

# Additional Docking Studies

Docking studies were also carried out to provide insights into the higher reactivity of pQR1445 to (*E*)-**2a**, but lower enantioselectivities compared to NCR. The structure of pQR1445 was predicted using Alphafold2. The pyridine structure adopts a unique configuration within the enzyme active site showing a distinct intermolecular O-H---N($sp^2$) (3.11-3.22 Å) hydrogen bond with ASN175 in preference to an O-H---O=C interaction (Figure S8). The hydrogen bond strength, thus binding affinity of (*E*)-**2a** is higher in pQR1445 (3.11 A, -6.1 kcal/mol) in comparison to NCR (3.22 A, -5.4 kcal/mol). The reduced er of (*E*)-**2a** may be due to the larger catalytic pocket in PQR1445 which can give rise to two productive conformations for reduction (Figure S7 B,C,E) with each conformation giving the opposite stereoisomer, in comparison to one for NCR (Figure S7 A,D). The lowest energy conformation for pQR1445 generates the *R* product (-6.1 kcal/mol), while the other gives the *S* product (-5.1 kcal/mol).

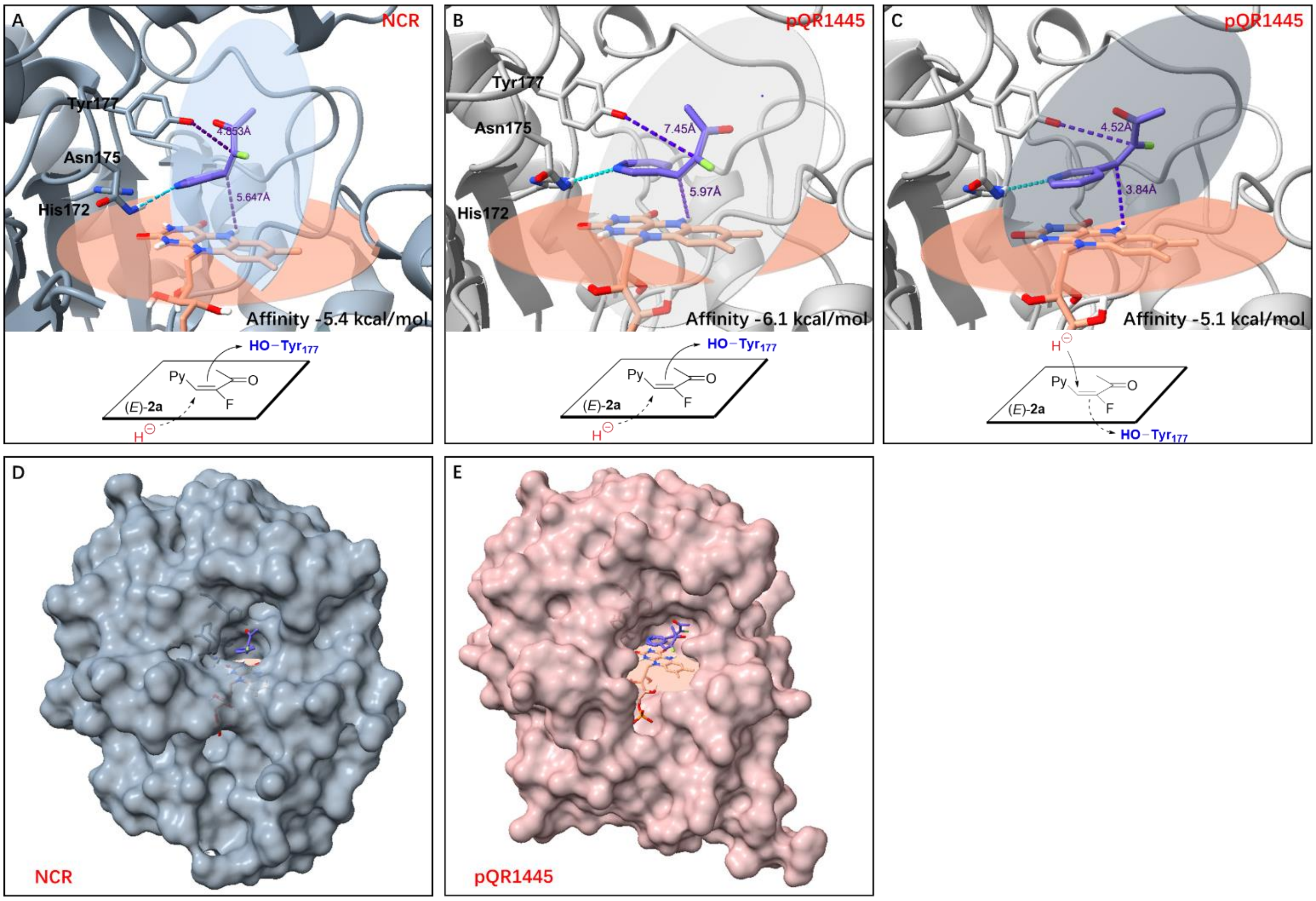


**Figure S8:** Docking NCR and pQR1445 with (*E*)-**2a**. **A**. NCR with (*E*)-**2a**, giving (*R*)-**8a**. **B.** pQR1445 with (*E*)-**2a**, giving (*R*)-**8a**. **C.** pQR1445 with (*E*)-**2a**, giving (*S*)-**8a**. **D.** NCR catalytic pocket. **E.** pQR1445 catalytic pocket.

When docking (*Z*)-**5a** with NCR, it adopts a similar orientation to the ester compound (*Z*)-**6a**, and so is not reactive as the hydride delivery and protonation would take place on the same face (Figure S9A). Docking studies with (*Z*)-**5b** (with the bulkier phenyl ketone) and NCR indicated that this substrate binds into the active site in a different

orientation where the carbonyl oxygen no longer binds to Asn175 and His172 and the phenyl group occupies the site previously occupied by the alkene substituent, enabling reduction to (*S*)-**11b** (Figure S9B). This explains why (*Z*)-**5b** is reactive even with a bulkier ketone substituent than (*Z*)-**5a**.

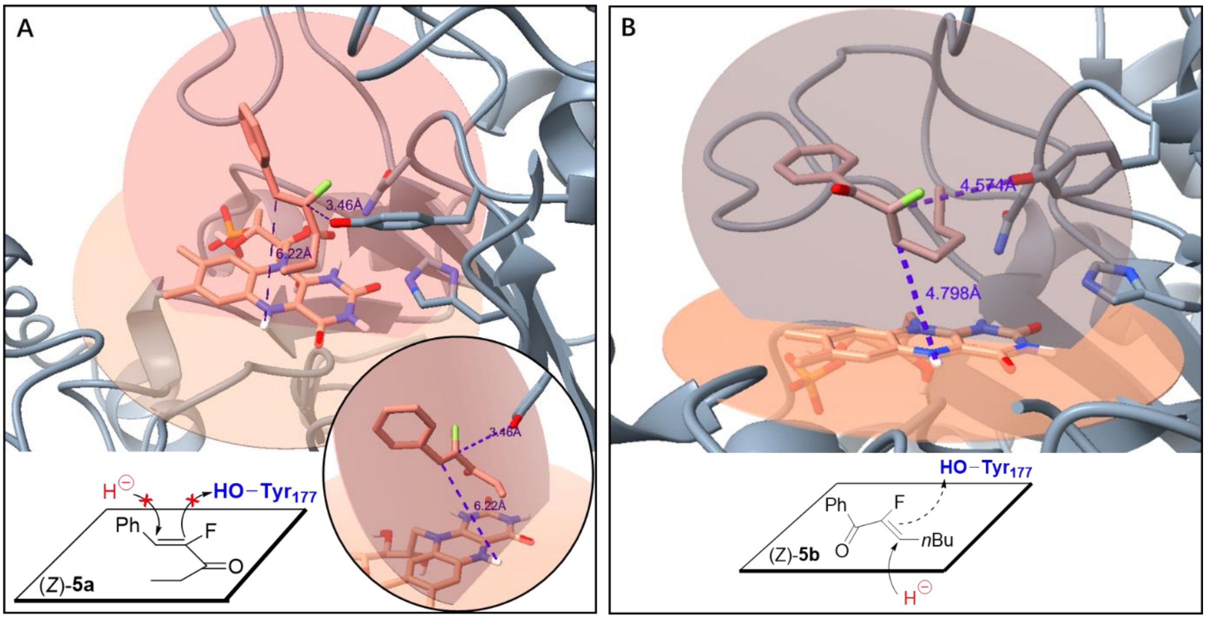


**Figure S9:** Docking NCR with (*Z*)-**5a** (which is unreactive) and (*Z*)-**5b** giving (*S*)-**11b**.

# Mutant design for (*E*)-1e

Since the enantioselectivity towards (*E*)-**1e** when using the wildtype NCR (WT-NCR) was low (78% yield, 67:33 e.r), we employed *in-silico* docking to design NCR variants predicted to enhance this enantioselectivity. As shown in Figure S9, docking calculations revealed two viable binding modes for (*E*)-**1e** in the WT-NCR active sites: one that positions the substrate for formation of (*R*)-**7e** (ΔG_bind = - 6.4 kcal mol$^{-1}$; Figure S10A) and another that would furnish (*S*)-**7e** (ΔG_bind = - 6.0 kcal mol$^{-1}$; Figure S9**B**). The slight energetic preference for the (*R*)-productive made the (*R*)-**7e** domain in the product distribution. To improve the (*R*)-selectivity, the residue Gly270 was substituted with a bulkier tyrosine (G270Y). Modelling indicates that the phenolic ring sterically occupied the space required for the (*S*)-productive binding mode, thereby blocking the formation of (*S*)-**7e**. (Figure S10C).

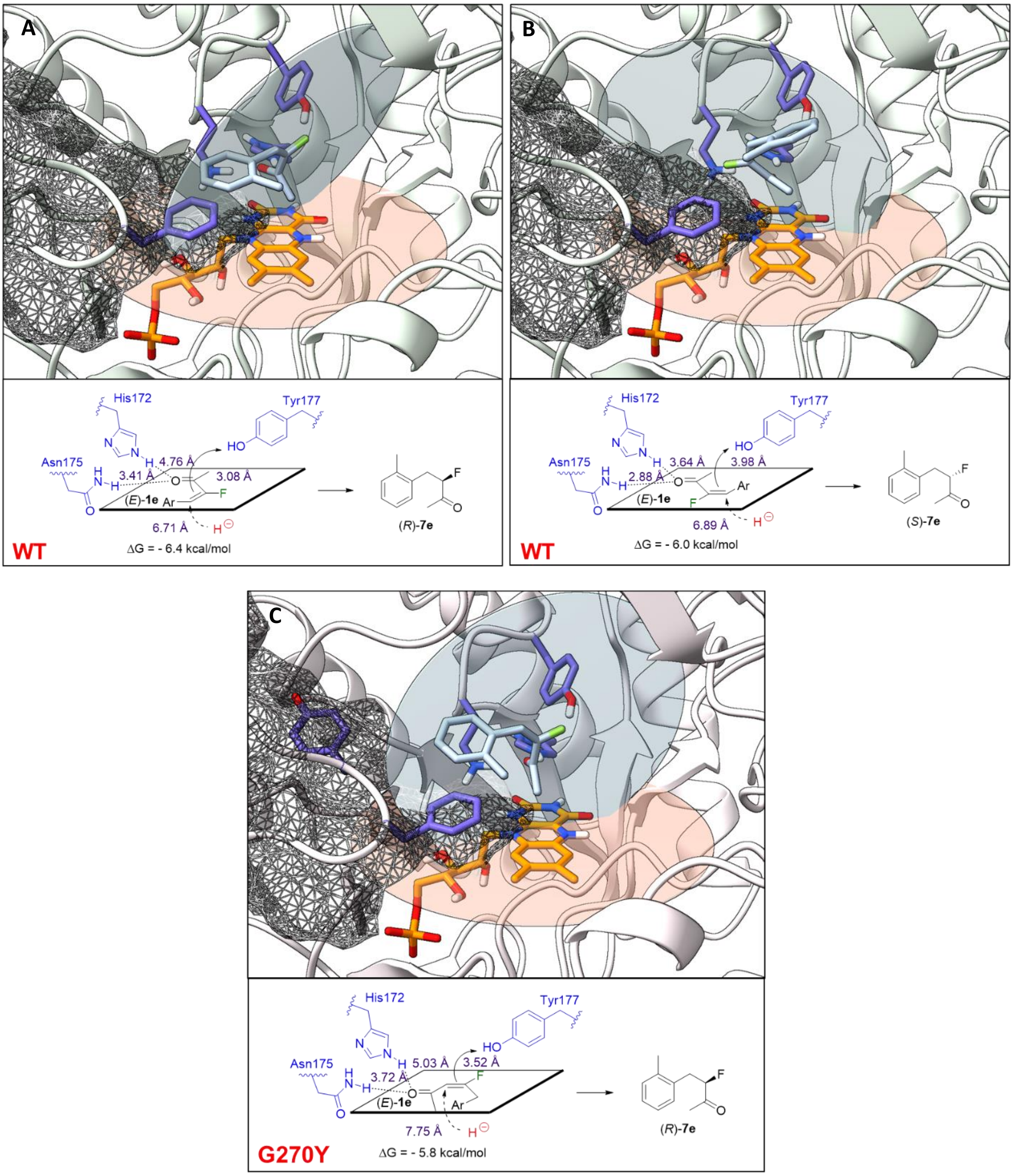


**Figure S10:** Docking of WT-NCR and NCR-G270Y with (*E*)-**1e. A.** Docking the WT-NCR with (*E*)-**1a**, yielding (*R*)-**7e**. **B.** Docking the WT-NCR with (*E*)-**1a**, yielding (*S*)-**7e**. **C.** Docking the NCR-G270Y with (*E*)-**1a**, yielding (*R*)-**7e**.

The gene encoding the NCR variant G270Y was synthesised (GenScript, UK), cloned into a pET-28 vector and expressed in *E. coli* BL21 (DE3) as detailed in the SI 'Enzyme Expression'. Enzyme reaction was performed as follows: substrate (E)-1a (10 mM), ERED lysates (80% (v/v) total protein in the lysates 5 mg $mL^{-1}$) and G6PDH lysates (10% (v/v), total protein in the lysates 1 mg $mL^{-1}$), $NAD^+$ (1 mM), G6PNa (50 mM), in Tris–HCl (100 mM) and DMSO (10%) at pH 7.5, 30 °C, 16 h, 700 rpm (Figure S11A). Reactions were performed in triplicate. Yields and

enantiomeric ratios were determined by GC analysis (against the standard curve, Figure S11C. GC conditions: Supelco Beta Dex 225 capillary GC column (30 m × 250 μm, 0.25 μm), method: 50 °C (hold 3 min), 50 °C to 170 °C (5 °C/min), 170 °C (hold 1 min)). As shown in Figure S11B, NCR-G270Y gave a higher enantioselectivity towards (*R*)-**7e** (90:10 e.r), but a lower yield at 41% (yield analysed by GC) compared to the WT-NCR (78% yield, 67:33 e.r). The docking result showed the binding energy of (*E*)-**1e** with NCR-G270Y ($\Delta$G_bind = - 5.8 kcal/mol, Figure S10C) is higher than the WT-NCR, rationalising the lower conversion while corroborating the enhanced R-selectivity.

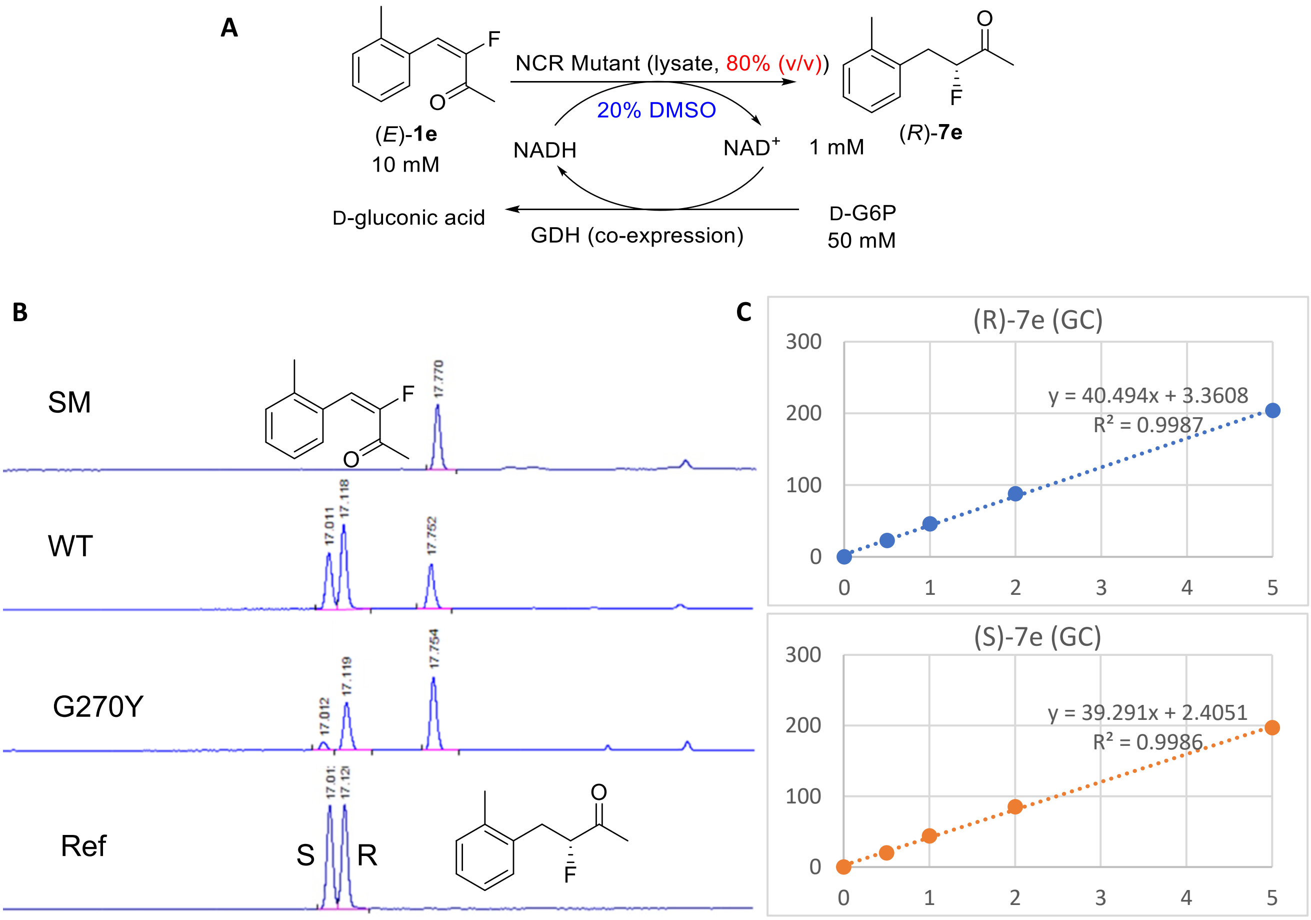


**Figure S11: A.** Scheme of enzyme reactions of (*E*)-**1e** using NCR mutants. **B.** GC trace comparison of the starting material (SM, (*E*)-**1e**), reaction using the WT-NCR (WT) and the mutant (G270Y) and the standard of (*rac*)-**7e** (Ref). **C**. Standard curve of (*R*)-**7e** and (*S*)-**7e** with GC analysis.

## Determination of Absolute Stereochemistry

Authentic reference standards of (*R*)-**12a**, (*R*)-**4a** and (*R*)-**11a** were synthesised according to the route below. Comparison of the HPLC traces of our products and these standards led to the assigned stereochemistry, whereby the (*E*) alkene gives the (*R*)-enantiomer.

In addition to this, the optical rotations of the products from the large-scale reactions were compared to literature values. These support the stereochemical assignments made from our enantioenriched sample. Andersson *et al.*[34] report a value of −110 (94:6 er, c = 0.1, $C_6D_6$) for (*S*)-**11a** which is similar to the order of magnitude we found for (*R*)-**11a**. The assignment of the absolute stereochemistry by Andersson for (*S*)-**7a** is inferred by comparison of their value for (*S*)-**11a** to the results reported by Enders. Enders *et al.*[108] synthesised an authentic sample via electrophilic fluorination of enantiopure α-silylketones.

| Starting Material | $[\alpha]^{D}25$ | Literature Value | Assignment |
|---|---|---|---|
| (*Z*)-**1a** | −92 (c = 0.1, $CHCl_3$) | −69 (95:5 er, c = 0.1 in $CHCl_3$)[34] | (*S*)-**7a** |
| (*E*)-**11a** | +108 (c = 1.10, $C_6D_6$) | +29 (>98: 2 er, c= 1.3 in $C_6D_6$)[108] | (*R*)-**11a** |

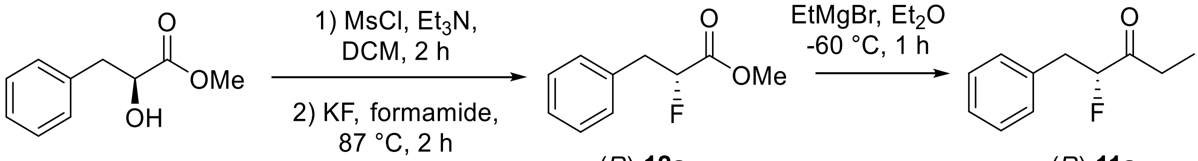


(*R*)-**12a** (*R*)-**11a**

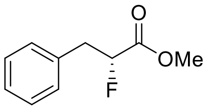


*(R)-Methyl 2-fluoro-3-phenylpropanoate* (**12a**): A solution of (*S*)-methyl-2-hydroxy-3-phenyl propanoate (2.40 g, 13.2 mmol) in $CH_2Cl_2$ (20 mL) was treated with methane sulfonyl chloride (2.04 mL, 26.4 mmol) followed by $Et_3N$ (4.58 mL, 33.0 mmol) and stirred at rt for 2 h. After this time $H_2O$ (20 mL) was added and the reaction mixture extracted with $CH_2Cl_2$ (20 mL), dried ($MgSO_4$), filtered and conc. *in* vacuo. The resultant orange oil was dissolved in formamide (6.6 mL), treated with KF (3.07 g, 52.8 mmol) and heated to 87 °C for 2 h. After this time the reaction was allowed to cool to rt and extracted with EtOAc (30 mL), washed with brine (10 mL), separated, dried ($MgSO_4$), filtered and conc. *in vacuo.* Purification *via* flash column chromatography (0-10% $Et_2O$/pentane) gave (*R*)-**12a** as a colourless oil (389 mg, 16%). Data in agreement with that reported above; HPLC: 98:2 er $t_{R(major)}$ 10.8 min, $t_{R(minor)}$ 11.9 min, (OD-H, 1% IPA/hexane, 1 mL/min); $[\alpha]_D^{25}$ = +22.2 (c = 1.19, $CHCl_3$).

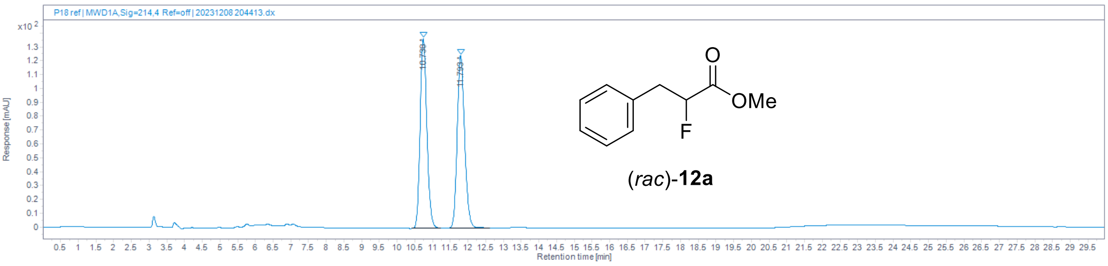


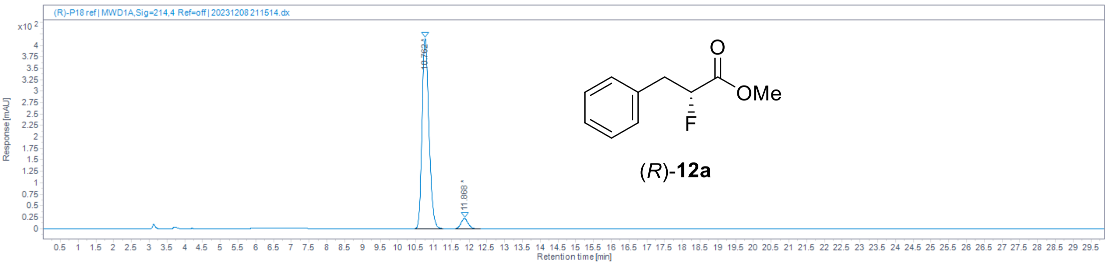


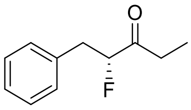

*(R)-3-Fluoro-4-phenylpentan-2-one* (**11a**): A solution of (*R*)-**12a** (100 mg, 0.549 mmol) in $Et_2O$ (1 mL) was cooled to -60 °C and treated with EtMgBr (0.55 mL, 1 M in THF), added dropwise over 20 min. The reaction was stirred at -60 °C for 10 min and quenched with $NH_4Cl_{(sat.\ aq.)}$ (1 mL). After warming to rt the solution was taken up in $Et_2O$ (10 mL), separated, dried ($MgSO_4$), filtered and conc. *in vacuo*. Purification *via* flash column chromatography (0-10% $Et_2O$/pentane) gave (*R*)-**11a** as a colourless oil (21 mg, 21%). Data in agreement with that reported above; HPLC: 98:2 er $t_{R(major)}$ 12.6 min, $t_{R(minor)}$ 14.5 min, (OD-H, 1% IPA/hexane, 1 mL/min); $[\alpha]_D^{25}$ = +97.6 (c = 1.00, $CHCl_3$).

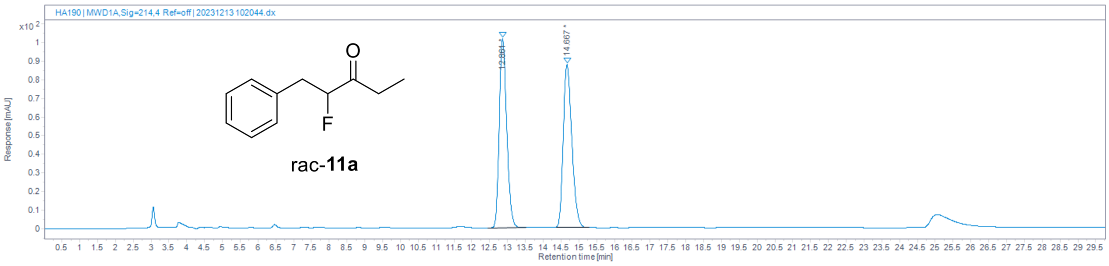


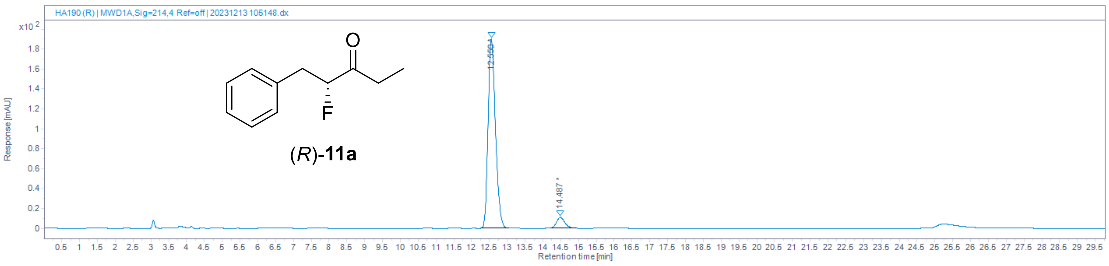

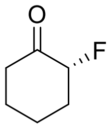


*(R)-2-Fluorocyclohexanone* (**10a**): Following the procedure of Enders *et al.*:[104] A solution of cyclohexanone (274 µL, 2.65 mmol), (2*S*,4*R* )-4-hydroxypyrrolidine-2-carboxylic acid (39 mg, 0.612 mmol) in MeCN (6 mL) was treated with Selectfluor (722 mg, 2.04 mmol) stirred at rt for 22 h. After this time the reaction solution was filtered through a silica plug, washed with MeCN (10 mL) and conc. *in* vacuo. The resultant oil/solid was dissolved in Et2O (30 mL), filtered and conc. *in vacuo.* Purification *via* flash column chromatography (0-50% $Et_2O$/pentane) gave (*R*)-**10a** as a colourless oil (94 mg, 40%). Data in agreement with that reported above; GC 64:36 er $t_{R(major)}$ 18.2 min, $t_{R(minor)}$ 18.0 min, (Supelco Beta Dex 225 capillary GC column (30 m × 250 µm, 0.25 µm), method: 50 °C (hold 3 min), 50 °C to 170 °C (5 °C/min), 170 °C (hold 1 min)); $[\alpha]_D^{25}$ = +14.6 (c = 0.30, $CHCl_3$) (assigned as (*R*) by comparison of the optical rotation value to the literature[100] +45.7 (c = 0.73, $CHCl_3$).

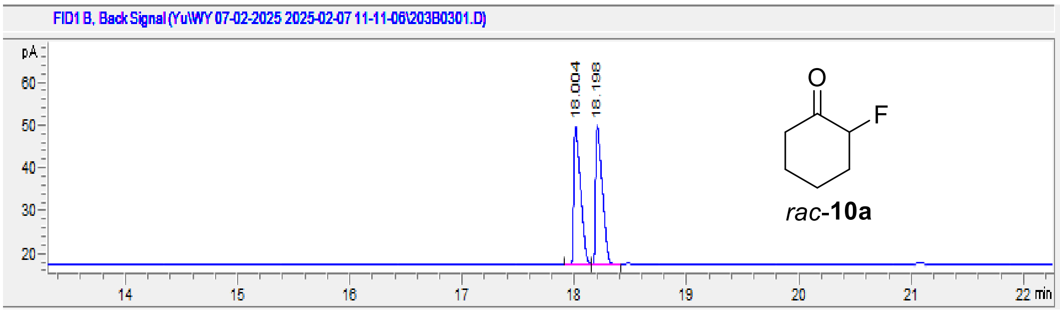


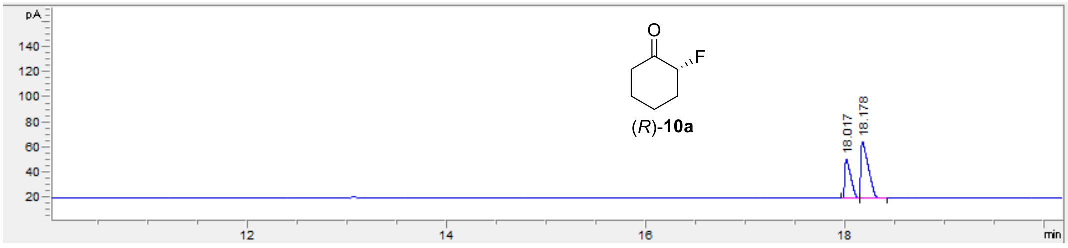

# Confirmation of Bio-reduction Stereochemistry

## Synthesis

*(E/Z)-3-fluoro-4-phenylbut-3-en-2-one-4-d* ((*E*/*Z*)-4-D-**1a**)

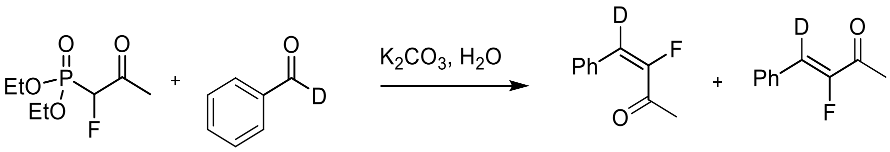


Following the procedure of Coutrot *et al.*:[56] In an Eppendorf tube, a mixture of diethyl (1-fluoro-2-oxopropyl)phosphonate (477 mg, 2.24 mmol) and benzaldehyde-d[1] (200 mg, 1.87 mmol) was treated with $K_2CO_3$ solution (50% wt solution, 763 µL). An exotherm was observed and the solution shaken for 25 min. After this time the reaction was diluted with $H_2O$ (8 mL), extracted into EtOAc (2 x 25 mL), dried ($MgSO_4$), filtered and conc. *in vacuo*. Purification by flash column chromatography (2-5% EtOAc/cyclohexane) gave *E*-4-D-**1a** as a pale-yellow oil (106 mg, 29%) and *Z*-4-D-**1a** as white solid (74 mg, 20%).

(*E*)-4-D-**1a**: $\upsilon_{max}$ (oil) 3028 (w), 1725 (s), 1702 (m), 1685 (s), 1621 (m), 1493 (m), 1448 (m), 1328 (m) 1268 (s); $^1$H NMR (700 MHz, $CDCl_3$) $\delta$ 7.60-7.59 (2H, m), 7.38-7.35 (3H, m), 2.32 (3H, d, *J* = 4.8 Hz); $^{13}$C NMR (176 MHz, $CDCl_3$) $\delta$ 192.9 (C, d, *J* = 38.4 Hz), 153.1 (C, d, *J* = 257.4 Hz), 130.9 (C, d, *J* = 10.2 Hz), 130.2 (CH, d, *J* = 2.7 Hz), 129.4 (CH, s), 128.4 (CH, s), 119.9 (CD, dt, *J* = 27.2, 23.3 Hz), 28.4 ($CH_3$, s); $^{19}$F NMR (659 MHz, $CDCl_3$) $\delta$ -113.3 to -113.2 (m); *m/z* [LRMS, ESI] (relative intensity, M) 166 (68, $[M+H]^+$); [HRMS, ESI] Calcd. $(C_{10}DH_8FO+H)^+$ 166.0773, found 166.0774.

(*Z*)-4-D-**1a**: m.p. 51-52 °C; $\upsilon_{max}$ (oil) 3028 (w), 2923 (m), 1725 (s), 1703 (s), 1686 (m), 1621 (m), 1493 (m), 1448 (m), 1328 (m) 1267 (s) cm$^{-1}$; $^1$H NMR (700 MHz, $CDCl_3$) $\delta$ 7.68 (2H, d, *J* = 7.4 Hz), 7.43-7.39 (3H, m), 2.43 (3H, d, *J* = 3.4 Hz); $^{13}$C NMR (176 MHz, $CDCl_3$) $\delta$ 192.5 (C, d, *J* = 33.2 Hz), 154.1 (C, d, *J* = 271.4 Hz), 131.1 (C, d, *J* = 4.1 Hz), 130.8 (CH, d, *J* = 8.3 Hz), 130.1 (CH, d, *J* = 2.8 Hz), 129.0 (CH, s), 115.6 (CD, td, *J* = 24.0, 5.8 Hz m), 25.9 ($CH_3$, s); $^{19}$F NMR (659 MHz, $CDCl_3$) $\delta$ -123.9 to -123.8 (m); *m/z* [LRMS, ESI] (relative intensity, M) 166 (100, $[M+H]^+$); [HRMS, ESI] Calcd. $(C_{10}DH_8FO+H)^+$ 166.0773, found 166.0774.

## Enzyme Reactions

*(3R,4S)-3-fluoro-4-phenylbutan-2-one-4-d* ((3*R*,4*S*)-**7a**)

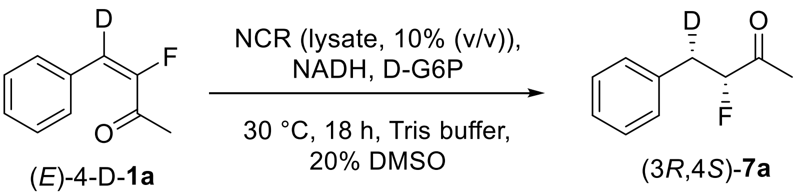


Enzyme reaction run on 30 mg scale. The reaction mixture contained 30 mg of (*E*)-4-D-**1a** (15 mM), 234 mg of D-G6P (75 mM) and 8 mg of $NAD^+$ (1 mM) in 12 mL of Tris-HCl buffer (100 mM, pH 7.5) with 20% (v/v) DMSO. Enzyme

lysates of the co-expressed ERs and G6PDH was added at 10% v/v. Reaction performed using a thermo mixer at 30 °C, 700 rpm. After 16 h, the reaction mixture was extracted with EtOAc, washed with ice cold $H_2O$ (5 mL), brine (5 mL), dried ($MgSO_4$), filtered and concentrated to give a yellow oil, no further purification was necessary (25 mg, 83%). $[\alpha]_D^{25}$ = −12 (c = 0.1, $CHCl_3$); $\upsilon_{max}$ (oil) 2923 (m), 2853 (w), 1724 (s); $^1H$ NMR (700 MHz, $CDCl_3$) $\delta$ 7.29-7.20 (5H, m), 4.93 (1H, ddt, *J* = 49.9, 3.8, 1.1 Hz), 3.17 (1H, ddt, *J* = 28.9, 3.8, -2.2 Hz), 2.13 (3H, d, *J* = 4.9 Hz); $^{13}C$ NMR (176 MHz, $CDCl_3$) $\delta$ 208.2 (C, d, *J* = 26.3 Hz), 135.4 (C, s), 129.6 (CH, s), 128.7 (CH, s), 127.3 (CH, s), 96.0 (CH, d, *J* = 187.3 Hz), 37.9 (CDH, dt, *J* = 20.1, 19.9 Hz), 26.6 ($CH_3$, s); $^{19}F$ NMR (659 MHz, $CDCl_3$) $\delta$ -188.41 (dds(6), *J* = 49.9, 28.8, 4.4 Hz); *m/z* [LRMS, EI] Calcd. $(C_{10}DH_{10}FO)^+$ 167, found 167, loss of HF seen under ESI conditions. Data in agreement with that reported in the literature where the diastereoisomers were isolated as a mixture and not resolved.[149]

*(3S,4S)-3-fluoro-4-phenylbutan-2-one-4-d* ((3*S*,4*S*)-**7a**)

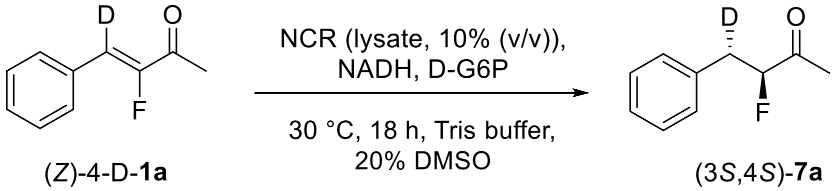


(*Z*)-4-D-**1a** (3*S*,4*S*)-**7a**

Enzyme reaction run on 30 mg scale. The reaction mixture contained 30 mg of (*Z*)-4-D-**1a** (15 mM), 234 mg of D-G6P (75 mM) and 8 mg of $NAD^+$ (1 mM) in 12 mL of Tris-HCl buffer (100 mM, pH 7.5) with 20% (v/v) DMSO. Enzyme lysates of the co-expressed ERs and G6PDH was added at 10% v/v. Reaction was performed using a thermo mixer at 30 °C, 700 rpm. After 16 h, the reaction mixture was extracted with EtOAc, washed with ice cold $H_2O$ (5 mL), brine (5 mL), dried ($MgSO_4$), filtered and concentrated to give a yellow oil, no further purification was necessary (28 mg, 92%). $[\alpha]_D^{25}$ = +24 (c = 0.1, $CHCl_3$); $\upsilon_{max}$ (oil) 1724 (s); $^1H$ NMR (700 MHz, $CDCl_3$) $\delta$ 7.29-7.23 (5H, m), 4.90 (1H, dd, *J* = 49.9, 7.6 Hz), 3.00 (1H, ddt, *J* = 26.1, 7.6, -2.2 Hz), 2.11 (3H, d, *J* = 4.9 Hz); $^{13}C$ NMR (176 MHz, $CDCl_3$) $\delta$ 208.1 (C, d, *J* = 26.2 Hz), 135.4 (C, s), 129.6 (CH, s), 128.7 (CH, s), 127.2 (CH, s), 96.0 (CH, d, *J* = 187.3 Hz), 38.2 (CDH, dt, *J* = 20.1, 20.1 Hz), 26.6 ($CH_3$, s); $^{19}F$ NMR (659 MHz, $CDCl_3$) $\delta$ -188.38 (dds(6), *J* = 49.9, 26.1, 4.5 Hz); *m/z* [HRMS, ESI] Calcd. $(C_{10}DH_{10}FO+H)^+$ 168.0930, loss of HF seen under ESI conditions, Calcd. $(C_{10}DH_9O+H)^+$ 148.0867 found 148.0871. Data in agreement with that reported in the literature where the diastereoisomers were isolated as a mixture and not resolved.[149]

The stereoselectivity of the reactions was confirmed by NMR analysis (see below).

# NMR Analysis

All spectra run on 700 MHz machine in $CDCl_3$.

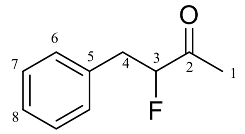


**2a**

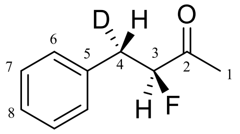


**(3*S*,4*S*)-7a**

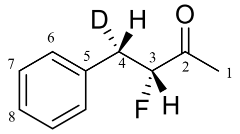


**(3*R*,4*S*)-7a**

**7a**

| Proton | Chemical shift $^1$H | Coupling pattern | *J* Values | Chemical shift $^{13}$C | Coupling pattern | *J* Values |
|---|---|---|---|---|---|---|
| 1-Me | 2.13 | D | $^4J_{HF}$ 4.89 | 26.54 | | |
| 2-CO | - | | | 208.11 | D | $^2J_{CF}$ 26.27 |
| 3-CHF | 4.93 | ddd | 7.59, 3.81, $^2J_{HF}$ 49.87 | 96.03 | D | $^1J_{CF}$ 187.25 |
| 4 | 3.05 | ddd | -14.83, 7.59, $^3J_{HF}$ 26.17 | 38.24 | D | d $^2J_{CF}$ 20.50 |
| 4' | 3.18 | ddd | -14.83, 3.81, $^3J_{HF}$ 28.91 | | | |
| 5-$C_q$ | - | | | 135.42 | | |
| 6-*o* | 7.23 | M | | 129.63 | | |
| 7-*m* | 7.32 | M | | 128.71 | | |
| 8-*p* | 7.29 | M | | 127.25 | | |

**Reduction of (*Z*)-4-D-1a gives (3*S*,4*S*)-7a**, 4' is replaced by D; $\gamma_H / \gamma_D$ = 6.514

| Proton | Chemical shift $^1$H | Coupling pattern | *J* Values | Chemical shift $^{13}$C | Coupling pattern | *J* Values |
|---|---|---|---|---|---|---|
| 1-Me | 2.111 | D | $^4J_{HF}$ 4.88 | 26.55 | | |
| 2-CO | - | | | 208.11 | D | $^2J_{CF}$ 26.19 |
| 3-CHF | 4.904 | dd | $^2J_{HF}$ 49.86, **7.63** | 95.97 | D | $^1J_{CF}$ 187.29 |
| 4 | 3.003 | ddt | $^3J_{HF}$ **26.05**, 7.63, $^2J_{HD}$ -2.20 | 38.24 | Dt | $^2J_{CF}$ 19.96, $^2J_{CD}$ 20.10 |
| 5-$C_q$ | - | | | 135.35 | | |
| 6-*o* | 7.201 | M | | 129.60 | | |
| 7-*m* | 7.290 | M | | 128.69 | | |
| 8-*p* | 7.239 | M | | 127.23 | | |

**Reduction of (*E*)-4-D-1a gives (3*R*,4*S*)-7a**, 4' is replaced by D; $\gamma_H$ / $\gamma_D$ = 6.514

| Proton | Chemical shift $^{1}$H | Coupling pattern | *J* Values | Chemical shift $^{13}$C | Coupling pattern | *J* Values |
|---|---|---|---|---|---|---|
| 1-Me | 2.133 | D | $^{4}J_{HF}$ 4.91 | 26.57 | | |
| 2-CO | - | | | 208.18 | D | $^{2}J_{CF}$ 26.25 |
| 3-CHF | 4.929 | ddt | $^{2}J_{HF}$ 49.85, **3.82**, $^{3}J_{HD}$ 1.07 | 95.97 | D | $^{1}J_{CF}$ 187.29 |
| 4 | 3.166 | ddt | $^{3}J_{HF}$ **28.85**, 3.82, $^{2}J_{HD}$ -2.23 | 37.92 | Dt | $^{2}J_{CF}$ 19.89, $^{2}J_{CD}$ 20.09 |
| 5-$C_q$ | - | | | 135.35 | | |
| 6-*o* | 7.225 | M | | 129.62 | | |
| 7-*m* | 7.314 | M | | 128.71 | | |
| 8-*p* | 7.263 | M | | 127.25 | | |

From (*Z*)-4-D-**1a**: $^{19}$F -188.38 dds(6) 49.9, 26.1, ~4.5

From (*E*)-4-D-**1a**: $^{19}$F -188.41 dds(6) 49.9, 28.8, ~4.4

From (*Z*)-4-D-**1a**: $^{3}J$($^{19}$F,$^{2}$H) coupling in the F19CPD (400, #24) spectrum is 4.30 Hz (=>19F,1H 28.0 Hz)

From (*E*)-4-D-**1a**: $^{3}J$($^{19}$F,$^{2}$H) coupling in the F19CPD (400, #24) spectrum is 3.86 Hz (=>19F,1H 25.1 Hz)

| $^{19}$F HOEs | | | | | Volume Integrations | | |
|---|---|---|---|---|---|---|---|
| | | o-Ph | 4-H | Me | o-Ph | 4-H | Me |
| 600ms | Z | 22 | 55 | 56 | | | |
| #32 both | E | 15 | 27 | 39 | | | |
| | | | | | | | |
| 400ms | Z | 11 | 31 | 32 | 13.6 | 34.2 | 31.1 |
| #33 both | E | 7 | 19 | 26 | 6.5 | 20.5 | 24.6 |

HOESY spectra of products obtained from (*Z*)-4-D-**1a** (left) and (*E*)-4-D-**1a** (right) alkenes (mixing time 400ms):

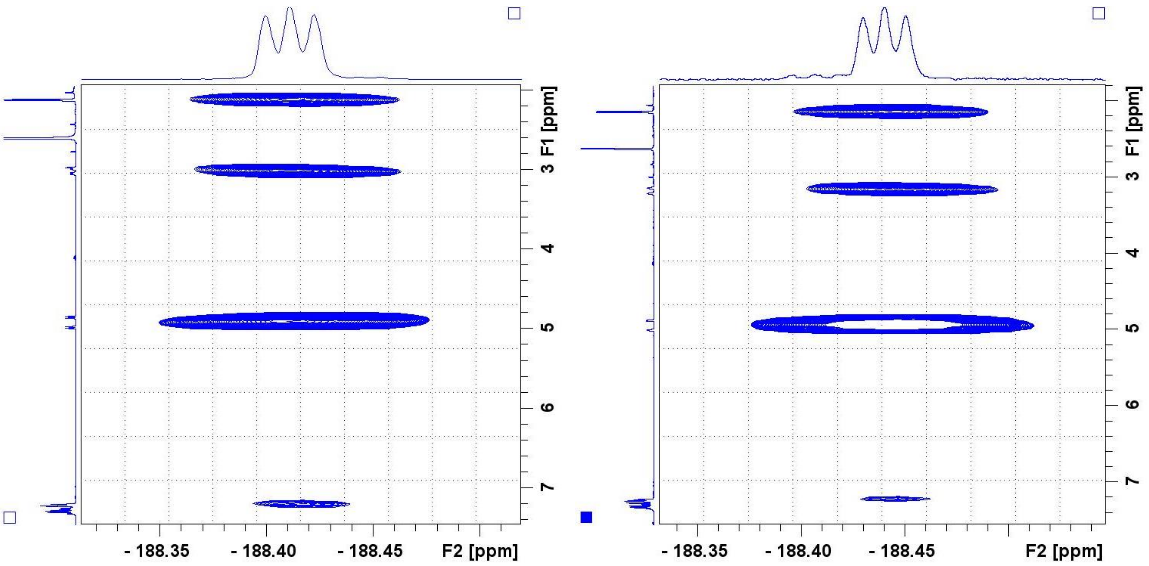


**Conformational Analysis using NMR and Computational Techniques**.

Initially, molecular mechanics calculations were carried out using MMX force field of the PCM program (version 8.5)[131] Two configurations (4*S*,3*S*) and (4*S*,3*R*) were considered, where the deuterated carbon in position 4 has *S* configuration in both diastereomers, whereas the fluorinated carbon in position 3 changes its configuration in two diastereomers. For simplicity, we refer to these as *S*,*S* and *S*,*R* diastereomers in further discussion. Using relaxed grid search of the PCM program, 7 and 5 conformers were identified for *S*,*S* and *S*,*R* diastereomers, respectively. The relative MMX energies of these conformers (in kcal mol$^{-1}$) were:

| | | | |
|---|---|---|---|
| **ss1** | 0.000 | **sr1** | 0.000 |
| **ss2** | 0.981 | **sr2** | 0.523 |
| **ss3** | 0.526 | **sr3** | 0.955 |
| **ss4** | 3.712 | **sr4** | 3.705 |
| **ss5** | 3.716 | **sr5** | 3.807 |
| **ss6** | 3.773 | | |
| **ss7** | 3.806 | | |

The structures were visualised and analysed using GaussView (version 6).[132] The corresponding geometries of the conformers were prepared as initial structures for DFT calculations, which were carried out using the *Gaussian 16* program (revision A.03).[133] Two different DFT functionals were considered, which have been shown previously[134] to reproduce satisfactorily experimentally measured conformational populations: PW6B95D3,[135] and M11L.[150] As a basis set, def2-TZVP was used in all DFT calculations.[137,138] The superfine numerical integration grid (with 175 radial shells and 974 angular points per shell for 1$^{st}$ row atoms and with 250 radial shells and 974

angular points per shell for atoms in the 2nd and later rows) was used in DFT geometry optimisations, combined with the "verytight" convergence condition (requesting the root-mean-square forces to be smaller than $1 \times 10^{-6}$ hartree bohr$^{-1}$). Frequency calculations were subsequently carried out at the same level of theory from which the Gibbs free energies were derived. These calculations were also used to verify that the optimized geometries correspond to true minima by checking whether any imaginary frequency was encountered. On calculating populations from free energies, the harmonic oscillator and rigid rotor models were used. No scaling of calculated frequencies were undertaken and as-calculated frequencies were used (for possible sources of error associated with the use of unscaled frequencies, see Alecu *et al*.[139] Chloroform solvent effects were included in all calculations using the integral equation formalism of the polarizable continuum model (IEFPCM), which is the default *SCRF* (Self-Consistent Reaction Field) method implemented in *Gaussian 16*.[140,141]

For conformer population calculations, the equilibrium constant was derived from the Gibbs free energy of formation:

$$K = e^{-\frac{\Delta G^0}{RT}} \quad (1)$$

where *R* is the universal gas constant and *T* is the temperature of the system. For the two-site exchange, the equilibrium constant is defined as:

$$K = \frac{p_1}{p_2} \quad (2)$$

where $p_1$ (in %) is the population of the preferred conformer. By calculating the Gibbs free energy differences ($\Delta G°$) using DFT methods and the above two equations, conformer populations were calculated. For multi-site exchange models, the relative free energies of conformers relative to that of the lowest-energy conformer in a given set of calculations for each diastereomer were calculated. These relative free energies were then used to calculate the corresponding *K* values and populations. In particular, an exchange between 7 conformers was considered for *S*,*S* diastereomer and a 5-site exchange was considered for the *S*,*R* diastereomer. As can be seen from Tables 1-3, DFT calculations satisfactorily predict experimental *J* couplings on the assumption that the reduction of the Z alkene leads to the *S*,*S* product and the reduction of the E alkene leads to the *S*,*R* product. The root mean square deviations for 6 predicted *J* couplings for two diastereomers were 1.17 and 1.22 Hz for PW6B93B and M11L functionals, respectively. Thus, as before,[133] the PW6B95D3 functional performed better than M11L. The PW6B95D3-optimised geometries of conformers are shown in Figure S9.

Additionally, we also considered a 3-site exchange model for both diastereomers where three conformers with the lowest relative energies (highest populations) were included into consideration. This model did not show in significant differences from the results included in Tables S13 and S14.

Heteronuclear Overhauser effects (HOEs) between $^{19}$F and $^{1}$H nuclei were also measured using mixing time of 400 ms. Calibrated relative to the geminal $^{19}$F,$^{1}$H Overhauser interaction (100%), the relative $^{19}$F Overhauser effects

were 14%, 34% and 31% for protons o-Ph, H4 and Me, respectively, for the product obtained from *Z* alkene and 7%, 21% and 25% for protons o-Ph, H4 and Me, respectively, for the product obtained from *E* alkene. Thus, for the product obtained from *Z* alkene, a significantly higher HOEs are observed for the (F3,H4) pair (34%) compared to that obtained from *E* alkene (21%). The PW6B95D3 functional predicts the following preferred *S*,*S* (**ss3**, 55% population) and *S*,*R* conformers (**sr2**, 56%):

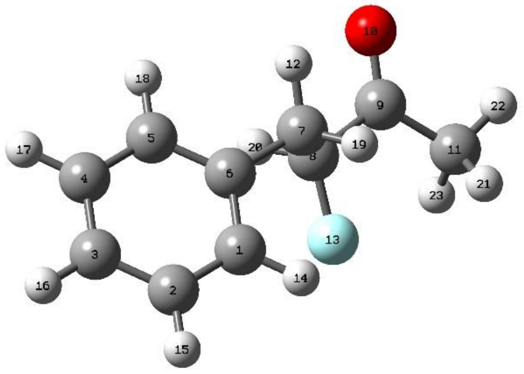

**ss3**

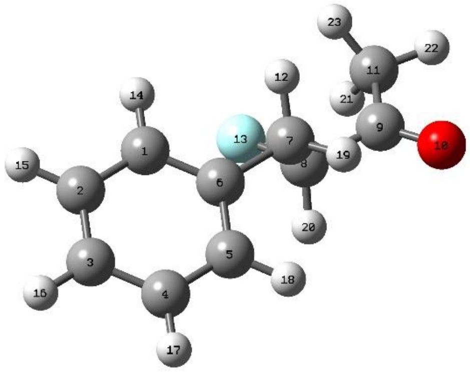

**sr2**

In both structures position 12 is deuterated. The internuclear distance between F (position 13 above) and H4 (position 19 above) is 2.60 Å in **ss3** and 3.28 Å in **sr2**. Thus, the observed HOEs are also in agreement with the conclusion reached from the *J* coupling analysis: the reduction of the *Z* alkene leads to the *S*,*S* product and the reduction of the *E* alkene leads to the *S*,*R* product.

**Table S13.** Relative free energies and percentage populations in different conformers of *S*,*S* and *S*,*R* diastereomers from **PW6B95D3**/def2-TZVP IEFPCM($CHCl_3$) calculations. The optimised geometries from these calculations were used for calculating *J* couplings at the B3LYP/6-311+G(2d,p) IEFPCM($CHCl_3$) level of theory. The $^3J$(H3,D) value was converted into $^3J$(H3,H4′) using the $J_{HH}$=6.512×$J_{HD}$ relationship. The averaged values were calculated using $J_{av} = \sum J_i p_i$, where $J_i$ is the *J* coupling value in conformer *i* and $p_i$ is the population of conformer *i* (percentage population divided by 100). The experimental value of $^3J$(H3,H4′) was determined from the $^1$H NMR spectrum of the nondeuterated sample.

| Conformer | *ΔG°*, kcal mol$^{-1}$ | Population, % | $^3J$(H3,H4), Hz | $^3J$(H3,D), Hz | $^3J$(H4,F), Hz | $^3J_{av}$(H3,H4), Hz | $^3J_{av}$(H3,H4′), Hz | $^3J_{av}$(H4,F), Hz |
|---|---|---|---|---|---|---|---|---|
| **ss7** | 2.627 | 0.65 | 5.23 | 1.68 | 13.02 | 7.27 | 3.59 | 23.70 |
| **ss6** | 2.283 | 1.17 | 10.21 | 0.25 | 15.42 | (exp. value | (exp. value | (exp. value |
| **ss4** | 2.223 | 1.30 | 3.35 | 0.74 | 39.57 | 7.63 Hz)[a] | 3.82 Hz)[a] | 26.06 Hz)[a] |
| **ss5** | 2.115 | 1.56 | 6.50 | 1.51 | 5.20 | | | |
| **ss2** | 1.561 | 3.97 | 5.35 | 1.66 | 13.88 | | | |
| **ss1** | 0.257 | 35.90 | 2.82 | 0.79 | 39.39 | | | |
| **ss3** | 0.000 | 55.45 | 10.37 | 0.28 | 14.69 | | | |
| | | | | | | | | |
| **sr5** | 2.302 | 1.15 | 1.63 | 1.57 | 35.42 | 3.44 | 7.26 | 27.39 |
| **sr4** | 2.086 | 1.66 | 4.75 | 0.52 | 15.21 | (exp. value | (exp. value | (exp. value |
| **sr3** | 1.569 | 3.98 | 10.83 | 0.83 | 3.56 | 3.82 Hz)[b] | 7.63 Hz)[b] | 28.85 Hz)[b] |
| **sr1** | 0.253 | 36.80 | 5.13 | 0.43 | 13.19 | | | |
| **sr2** | 0.000 | 56.41 | 1.82 | 1.59 | 38.52 | | | |

[a] The experimental values for the product obtained from (*Z*)-4-D-**1a**.

[b] The experimental values for the product obtained from (*E*)-4-D-**1a**.

**Table S14.** Relative free energies and percentage populations in different conformers of *S,S* and *S,R* diastereomers from **M11L**/def2-TZVP IEFPCM($CHCl_3$) calculations. The optimised geometries from these calculations were used for calculating *J* couplings at the B3LYP/6-311+G(2d,p) IEFPCM($CHCl_3$) level of theory. The $^3J$(H3,D) value was converted into $^3J$(H3,H4′) using the $J_{HH}$=6.512×$J_{HD}$ relationship. The averaged values were calculated using $J_{av} = \sum J_i p_i$, where $J_i$ is the *J* coupling value in conformer *i* and $p_i$ is the population of conformer *i* (percentage population divided by 100). The experimental value of $^3J$(H3,H4′) was determined from the $^1$H NMR spectrum of the nondeuterated sample.

| Conformer | *ΔG°*, kcal mol$^{-1}$ | Population, % | $^3J$(H3,H4), Hz | $^3J$(H3,D), Hz | $^3J$(H4,F), Hz | $^3J_{av}$(H3,H4), Hz | $^3J_{av}$(H3,H4′), Hz | $^3J_{av}$(H4,F), Hz |
|---|---|---|---|---|---|---|---|---|
| **ss4** | 2.448 | 0.85 | 3.44 | 0.81 | 41.51 | 8.16 | 3.94 | 23.17 |
| **ss7** | 2.399 | 0.92 | 4.97 | 1.84 | 16.68 | (exp. value | (exp. value | (exp. value |
| **ss5** | 2.221 | 1.24 | 6.58 | 1.68 | 8.15 | 7.63 Hz)[a] | 3.82 Hz)[a] | 26.06 Hz)[a] |
| **ss2** | 1.287 | 6.02 | 5.62 | 1.82 | 15.75 | | | |
| **ss6** | 1.184 | 7.16 | 11.38 | 0.33 | 13.35 | | | |
| **ss1** | 0.319 | 30.89 | 2.89 | 0.87 | 41.32 | | | |
| **ss3** | 0.000 | 52.93 | 11.25 | 0.3 | 14.93 | | | |
| | | | | | | | | |
| **sr4** | 2.282 | 1.13 | 5.24 | 0.54 | 16.11 | 3.44 | 8.12 | 28.92 |
| **sr3** | 1.269 | 6.24 | 11.87 | 0.86 | 3.55 | (exp. value | (exp. value | (exp. value |
| **sr5** | 1.196 | 7.06 | 2.13 | 1.75 | 37.08 | 3.82 Hz)[b] | 7.63 Hz)[b] | 28.85 Hz)[b] |
| **sr1** | 0.296 | 32.30 | 5.68 | 0.44 | 13.13 | | | |
| **sr2** | 0.000 | 53.28 | 1.98 | 1.73 | 40.66 | | | |

[a] The experimental values for the product obtained from (*Z*)-4-D-**1a**.

[b] The experimental values for the product obtained from (*E*)-4-D-**1a**.

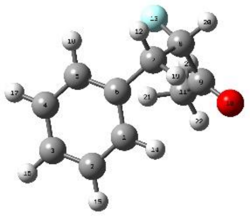

**ss1**

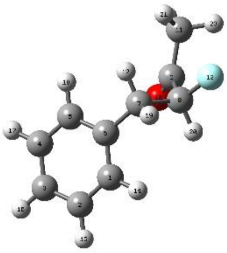

**ss2**

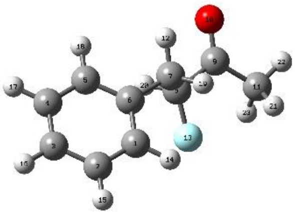

**ss3**

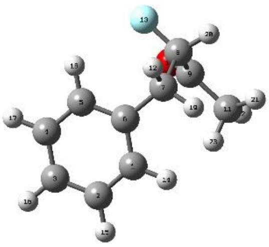

**ss4**

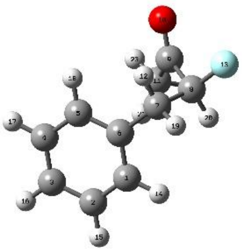

**ss5**

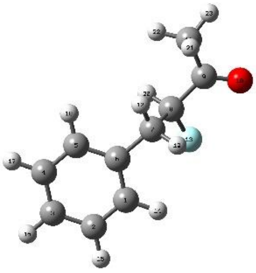

**ss6**

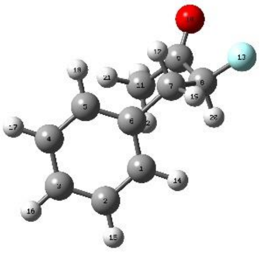

**ss7**

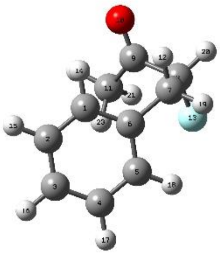

**sr1**

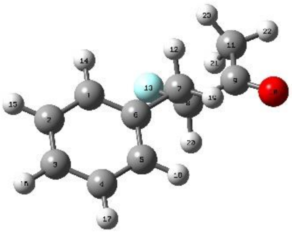

**sr2**

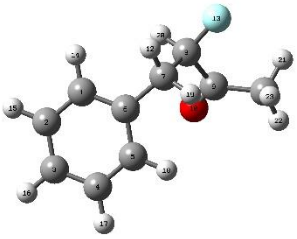

**sr3**

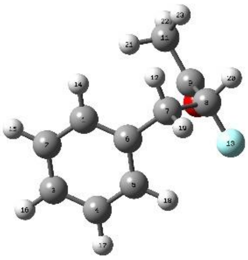

**sr4**

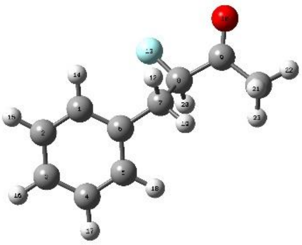

**sr5**

**Figure S12**. The geometries of conformers optimised at the PW6B95D3/def2-TZVP IEFPCM($CHCl_3$) level of theory. In all 12 structures, position 12 is deuterated.

# References


1. S. Ali, J. Zhou, "Highlights on U.S. FDA-approved fluorinated drugs over the past five years (2018–2022)" *Eur. J. Med. Chem.* **2023**, 115476.
2. G. Shabir, A. Saeed, W. Zahid, F. Naseer, Z. Riaz, N. Khalil, Muneeba, F. Albericio, "Chemistry and Pharmacology of Fluorinated Drugs Approved by the FDA (2016–2022)" *Pharmaceuticals* **2023**, *16*, 1162.
3. I. Inoue, Y. Sumii, N. Shibata, "Contribution of Organofluorine Compounds to Pharmaceuticals" *ACS Omega*, **2020**, *5*, 10633-10640.
4. P. Jeschke, "Recent developments in fluorine-containing pesticides" *Pest Manag. Sci.* 2024, *80*, 3065-3087.
5. Y. Ogawa, E. Tokunaga, O. Kobayashi, K. Hirai, N. Shibata, "Current Contributions of Organofluorine Compounds to the Agrochemical Industry" *iScience*, **2020**, *23*, 101467.
6. S. Purser, P. R. Moore, S. Swallow, V. Gouverneur, "Fluorine in medicinal chemistry" *Chem. Soc. Rev*. **2008**, *37*, 320-330;
7. E. P. Gillis, K. J. Eastman, M. D. Hill, D. J. Donnelly, N. A. Meanwell, "Applications of fluorine in medicinal chemistry" *Med. Chem.* **2015**, *58*, 8315–8359;
8. C. S. Teschers, C. G. Daniliuc, G. Kehr, R. Gilmour, "Conformational control enabled by the fluorine gauche effect in a model of the β2-AR agonist salbutamol (Ventolin™)" *J. Fluor. Chem.* **2018**, *210*, 1-5;
9. P. Bentler, N. Erdeljac, K. Bussmann, M. Ahlqvist, L. Knerr, K. Bergander, C. G. Daniliuc, R. Gilmour, "Stereocontrolled synthesis of tetrafluoropentanols: Multivicinal fluorinated alkane units for drug discovery" *Org. Lett.* **2019**, *21*, 7741-7745.
10. A. G. Myers, J. K. Barbay, B. Zhong, "Asymmetric Synthesis of Chiral Organofluorine Compounds: Use of Nonracemic Fluoroiodoacetic Acid as a Practical Electrophile and Its Application to the Synthesis of Monofluoro Hydroxyethylene Dipeptide Isosteres within a Novel Series of HIV Protease Inhibitors" *J. Am. Chem. Soc.* **2001**, *123*, 7207-7219.
11. D. Cahard, X. Xu, S. Couve-Bonnaire, X. Pannecoucke, "Fluorine & chirality: how to create a nonracemic stereogenic carbon–fluorine centre?" *Chem. Soc. Rev.* **2010**, *39*, 558-568.
12. J.-A. Ma, D. Cahard, "Update 1 of: Asymmetric Fluorination, Trifluoromethylation, and Perfluoroalkylation Reactions" *Chem. Rev.* **2008**, *108*, PR1-PR43.
13. C. Bobbioa, V. Gouverneur, "Catalytic asymmetric fluorinations" *Org. Biomol. Chem.* **2006**, *4*, 2065-2075.
14. J.-A. Ma, D. Cahard, "Asymmetric Fluorination, Trifluoromethylation, and Perfluoroalkylation Reactions" *Chem. Rev.* **2004**, *104*, 6119-6146.
15. T. D. Beeson, D. W. C. MacMillan, "Enantioselective Organocatalytic α-Fluorination of Aldehydes" *J. Am. Chem. Soc.* **2005**, *127*, 8826-8828.
16. M. Marigo, D. Fielenbach, A. Braunton, A. Kjærsgaard, K. A. Jørgensen, "Enantioselective Formation of Stereogenic Carbon–Fluorine Centers by a Simple Catalytic Method" *Angew. Chem. Int. Ed.* **2005**, *44*, 3703-3706.
17. D. D. Steiner, N. Mase, C. F. Barbas, "Direct Asymmetric α-Fluorination of Aldehydes" *Angew. Chem. Int. Ed.* **2005**, *44*, 3706-3710.
18. F. Li, Z. Wu, J. Wang, "Oxidative Enantioselective α-Fluorination of Aliphatic Aldehydes Enabled by *N*-Heterocyclic Carbene Catalysis" *Angew. Chem. Int. Ed.* **2015**, *54*, 656-659.
19. X. Dong, W. Yang, W. Hu, J. Sun, "*N*-Heterocyclic Carbene Catalyzed Enantioselective α-Fluorination of Aliphatic Aldehydes and α-Chloro Aldehydes: Synthesis of α-Fluoro Esters, Amides, and Thioesters" *Angew. Chem. Int. Ed.* **2015**, *54*, 660-663.
20. P. Wheeler, H. U. Voraa, T. Rovis, "Asymmetric NHC-catalyzed synthesis of α-fluoroamides from readily accessible α-fluoroenals" *Chem. Sci.* **2013**, *4*, 1674-1679.

21. L. Wang, X. Jiang, J. Chen, Y. Huang, "Enantio- and Diastereoselective Hydrofluorination of Enals by *N*-Heterocyclic Carbene Catalysis" *Angew. Chem. Int. Ed.* **2019**, *58*, 7410-7414.
22. P. Kwiatkowski, T. D. Beeson, J. C. Conrad, D. W. C. MacMillan, "Enantioselective Organocatalytic α-Fluorination of Cyclic Ketones" *J. Am. Chem. Soc.* **2011**, *133*, 1738–1741.
23. M. W. Ashford, C. Xu, J. J. Molloy, C. Carpenter-Warren, A. M. Z. Slawin, A. G. Leach, A. J. B. Watson, "Catalytic Enantioselective Synthesis of Heterocyclic Vicinal Fluoroamines by Using Asymmetric Protonation: Method Development and Mechanistic Study" *Chem. Eur. J.* **2020**, *26*, 12249-12255.
24. R. Liu, Q. Zhang, Z. Lin, T. N. Snaddon, W. Zi, "Modular Access to Fluorinated Quaternary Stereocenters via Palladium/Lewis Base Synergistic Catalysis" *Angew. Chem. Int. Ed.* **2025**, accepted, e202504121.
25. G. Pupo, A. C. Vicini, D. M. H. Ascough, F. Ibba, K. E. Christensen, A. L. Thompson, J. M. Brown, R. S. Paton, V. Gouverneur, "Hydrogen Bonding Phase-Transfer Catalysis with Potassium Fluoride: Enantioselective Synthesis of β-Fluoroamines" *J. Am. Chem. Soc.* **2019**, *141*, 2878-2883.
26. G. Pupo, F. Ibba, D. M. H. Ascough, A. C. Vicini, P. Ricci, K. E. Christensen, L. Pfeifer, J. R. Morphy, J. M. Brown, R. S. Paton, V. Gouverneur, "Asymmetric nucleophilic fluorination under hydrogen bonding phase-transfer catalysis" *Science*, **2018**, *360*, 638-642.
27. D. Cahard, C. Audouard, J. C. Plaquevent, N. Roques, "Design, Synthesis, and Evaluation of a Novel Class of Enantioselective Electrophilic Fluorinating Agents: *N*-Fluoro Ammonium Salts of cinchona Alkaloids (F-CA-BF4)" *Org. Lett.* **2000**, *2*, 3699–3701.
28. W. L. Hu, X. G. Hu, L. Hunter, "Recent Developments in the Deoxyfluorination of Alcohols and Phenols: New Reagents, Mechanistic Insights, and Applications" *Synthesis*, **2017**, 4917-4930.
29. H. Won Moon, M. N. Lavagnino, S. Lim, M. D. Palkowitz, M. D. Mandler, G. L. Beutner, M. J. Drance, J. M. Lipshultz, P. M. Scola, A. T. Radosevich, "Deoxyfluorination of 1°, 2°, and 3° Alcohols by Nonbasic O–H Activation and Lewis Acid-Catalyzed Fluoride Shuttling" *J. Am. Chem. Soc.* **2023**, *145*, 22735–22744.
30. M. K. Nielsen, C. R. Ugaz, W. Li, A. G. Doyle, "PyFluor: A Low-Cost, Stable, and Selective Deoxyfluorination Reagent" *J. Am. Chem. Soc.* **2015**, *137*, 9571–9574
31. F. Sladojevich, S. I. Arlow, P. Tang, T. Ritter, "Late-Stage Deoxyfluorination of Alcohols with PhenoFluor" *J. Am. Chem. Soc.* **2013**, *135*, 2470–2473.
32. A. L'Heureux, F. Beaulieu, C. Bennett, D. R. Bill, S. Clayton, F. LaFlamme, M. Mirmehrabi, S. Tadayon, D. Tovell, M. Couturier, "Aminodifluorosulfinium Salts: Selective Fluorination Reagents with Enhanced Thermal Stability and Ease of Handling" *J. Org. Chem.* **2010**, *75*, 3401–3411.
33. For a review article, see: T. Charvillat, P. Bernardelli, M. Daumas, X. Pannecoucke, V. Ferey, T. Besset, "Hydrogenation of fluorinated molecules: an overview" *Chem. Soc. Rev.* **2021**, *50*, 8178-8192.
34. S. Ponra, J. Yang, S. Kerdphon, P. G. Andersson, "Asymmetric Synthesis of Alkyl Fluorides: Hydrogenation of Fluorinated Olefins" *Angew. Chem. Int. Ed.* **2019**, *58*, 9282-9287.
35. D. M. Sedgwick, G. B. Hammond, " The history and future challenges associated with the hydrogenation of vinyl fluorides" *J. Fluor. Chem.* **2018**, *207*, 45.
36. M. Biosca, E. Salomó, P. De La Cruz-Sánchez, A. Riera, X. Verdaguer, O. Pàmies, M. Diéguez, "Extending the Substrate Scope in the Hydrogenation of Unfunctionalized Tetrasubstituted Olefins with Ir-P Stereogenic Aminophosphine-Oxazoline Catalysts" *Org. Lett.,* **2019**, *21*, 807 – 811.
37. M. Saburi, L. Shao, T. Sakurai, Y. Uchida, *Tetrahedron Lett.* **1992**, *33*, 7877-7880.
38. M.Buhler, H. Giesel, W. Tischer, H. Simon, "Occurrence and the possible physiological role of 2-enoate reductases" *FEBS Letters*, **1980**, *109*, 244-246.
39. M. Buhler, H. Simon, "On the Kinetics and Mechanism of Enoate Reductase" *Z. Physiol. Chem.* **1982**, *363*, 609-625.
40. F. Luo, P. Wang, Y. Gong, "Highly enantioselective bioreduction of 2-fluorocinnamyl alchohols mediated by Saccharomyces cerevisiae" *Tetrahedron Lett.* **2010**, *51*, 1693-1695.

41. S. Ju, D. Li, B. Khanh Mai, X. Liu, A. Vallota-Eastman, J. Wu, D. L. Valentine, P. Liu, Y. Yang, "Stereodivergent photobiocatalytic radical cyclization through the repurposing and directed evolution of fatty acid photodecarboxylases" *Nat. Chem.* **2024**, *16*, 1339-1347.
42. H. S. Toogood, J. M. Gardiner, N. S. Scrutton, "Biocatalytic Reductions and Chemical Versatility of the Old Yellow Enzyme Family of Flavoprotein Oxidoreductases" *ChemCatChem* **2010**, *2*, 892-914.
43. H. S. Toogood, N. S. Scrutton, "Discovery, Characterization, Engineering, and Applications of Ene-Reductases for Industrial Biocatalysis" *ACS Catal.* **2018**, *8*, 3532-3549.
44. K. T. Roy, R. Sreedharan, P. Ghosh, T. Gandhi, D. Maiti, "Ene-Reductase: A Multifaceted Biocatalyst in Organic Synthesis" *Chem. Eur. J.* **2022**, *28*, e202103949.
45. D. Dobrijevic, L. Benhamou, A. E. Aliev, D. Méndez-Sánchez, D. Dawson, D. Baud, N. Tappertzhofen, T. S. Moody, C. A. Orengo, H. C. Hailes, J. M. Ward, "Metagenomic ene-reductases for the bioreduction of sterically challenging enones" *RSC Adv.* **2019**, *9*, 36608-36614.
46. C. K. Winkler, D. Clay, S. Davies, P. O'Neill, P. McDaid, S. Debarge, J. Steflik, M. Karmilowicz, J. W. Wong, K. Faber, "Chemoenzymatic Asymmetric Synthesis of Pregabalin Precursors via Asymmetric Bioreduction of β-Cyanoacrylate Esters Using Ene-Reductases" *J. Org. Chem.* **2013**, *78*, 1525-1533.
47. D. Mangan, I. Miskelly, T. S. Moody, "A Three-Enzyme System Involving an Ene-Reductase for Generating Valuable Chiral Building Blocks" *Adv. Syn. Catal.* **2012**, *354*, 2185-2190.
48. A. Papadopoulou, C. Peters, S. Borchert, K. Steiner, R. Buller, "Development of an Ene Reductase-Based Biocatalytic Process for the Production of Flavor Compounds" *Org. Process Res. Dev.* **2022**, *26*, 2102-2110.
49. Y. Liu, S. G. Bender, D. Sorigue, D. J. Diaz, A. D. Ellington, G. Mann, S. Allmendinger, T. K. Hyster, "Photoenzymatic Generation of Unstabilized Alkyl Radicals: An Asymmetric Reductive Cyclization" *J. Am. Chem. Soc.* **2024**, *146*, 7191–7197.
50. In the review article [42] a general scheme for asymmetric reduction of enones with EREDs implies that α-fluoroenones have previously been reduced with EREDs. However, none of the cited references contain any such reactions.
51. E. Brenna, F. G. Gatti, A. Manfredi, D. Monti, F. Parmeggiani, "Biocatalyzed Enantioselective Reduction of Activated C=C Bonds: Synthesis of Enantiomerically Enriched α-Halo-β-arylpropionic Acids" *Eur. J. Org. Chem.* **2011**, 4015-4022;
52. G. Tasnádi, C. K. Winkler, D. Clay, N. Sultana, W. M. F. Fabian, M. Hall, K. Ditrich, K. A. Faber, "A Substrate-Driven Approach to Determine Reactivities of α,β-Unsaturated Carboxylic Esters Towards Asymmetric Bioreduction" *Chem. Eur. J.* **2012**, *18*, 10362-10367.
53. F. Parmeggiani, A. R. Casamajo, D. Colombo, M. C. Ghezzi, J. L. Galman, R. A. Chica, E. Brenna, N. J. Turner, "Biocatalytic retrosynthesis approaches to D-(2,4,5-trifluorophenyl)alanine, key precursor of the antidiabetic sitagliptin" *Green Chem.* **2019**, *21*, 4368-4379;
54. F. Parmeggiani, E. Brenna, D. Colombo, F. G. Gatti, F. Tentori, D. Tessaro, ""A Study in Yellow": Investigations in the Stereoselectivity of Ene-Reductases" *ChemBioChem*, **2022**, *23*, e202100445.
55. I. M. Ferreira, D. E. Q. Jimenez, S. Q. Lopes, A de N. de Oliveira, M. J. S. Matos, A. L. M. Porto, "Ene-reductases: Contribution from Brazilian fungi in organic synthesis" *Results in Chem.* **2023**, *5*, 100965.
56. P. Coutrot, C. Grison, M. Lachgar, A. Ghribi, "A general and efficient synthesis of β-ketophosphonates" *Bull. Soc. Chim. Fr.* **1995**, *132*, 925-942.
57. P. Coutrot, C. Grison, ". Synthese Generale de α-Cetophosphonates α-Fluorés Precurseurs d'Enones [illegible]-Fluorées. Application en Serie Pyrethrenique" *Tetrahedron Lett.* **1988**, *29*, 2655-2658.
58. I. Shimizu, H. Ishii, "Synthesis of α-fluoroketones based on palladium-catalyzed Decarboxylation reactions of allyl β-keto carboxylates" *Tetrahedron*, **1994**, *50*, 487-495.
59. S. Reich, H. Wolfgang Hoeffken, B. Rosche, B. M. Nestl, B. Hauer, "Crystal Structure Determination and Mutagenesis Analysis of the Ene Reductase NCR" *ChemBioChem*, **2012**, *13*, 2400.
60. J. Eberhardt, D. Santos-Martins, A. F. Tillack, S. Forli, "AutoDock Vina 1.2.0: New Docking Methods, Expanded Force Field, and Python Bindings" *J. Chem. Inf. Model.* **2021**, *61*, 3891-3898;

61. O. Trott, A. Olson, "AutoDock Vina: improving the speed and accuracy of docking with a new scoring function, efficient optimization and multithreading" *J. Comp. Chem.* **2010**, *31*, 455-461.
62. J. K. Augustine, A. Bombrun, S. Venkatachaliah, A. Jothib, "Titanium mediated olefination of aldehydes with α-haloacetates: an exceptionally stereoselective and general approach to (*Z*)-α-haloacrylates" *Org. Biomol. Chem.* **2013**, *11*, 8065-8072.
63. F. Zhu, P. Xu, F. Zhou, C. Wang, J. Zhou, "Recycle Waste Salt as Reagent: A One-Pot Substitution/Krapcho Reaction Sequence to α-Fluorinated Esters and Sulfones" *Org. Lett.* **2015**, *17*, 962-975.
64. Y. A. Pompeu, B. Sullivan, J. D. Stewart, "X-ray Crystallography Reveals How Subtle Changes Control the Orientation of Substrate Binding in an Alkene Reductase" ACS Catal. 2013, 3, 2376-2390.
65. R. A. Sheldon, "The E factor at 30: a passion for pollution prevention" Green Chem. 2023, 25, 1704-1728.
66. C. Jimenez-Gonzalez, C. S. Ponder, Q. B. Broxterman, J. B. Manley, "Using the Right Green Yardstick: Why Process Mass Intensity Is Used in the Pharmaceutical Industry To Drive More Sustainable Processes" Org. Proc. Res. Dev. 2011, 15, 912-917.
67. K. Van Aken, L. Strekowski, L. Patiny, "EcoScale, a semi-quantitative tool to select an organic preparation based on economical and ecological parameters" Beilstein J. Org. Chem. 2006, 2, doi: 10.1186/1860-5397-2-3.

Supporting Information References

68. K. Radwan-Olszewska, F. Palacios, P. Kafarski, „Selective Synthesis of α-Fluoro-β-keto- and α-Fluoro-β-aminophosphonates via Electrophilic Fluorination by Selectfluor" *J. Org. Chem.* **2011**, *76*, 1170-1173
69. P. Coutrot, C. Grison, R. Sauvetre, "A general and efficient synthesis of β-ketophosphonates" *J. Organomet. Chem.* **1987**, *332*, 1-8.
70. R. Waschbüsch, J. Carran, P. Savignac, "New routes to diethyl 1-fluoromethylphosphonocarboxylates and diethyl 1-fluoromethylphosphonocarboxylic acid" *Tetrahedron* **1997**, *53*, 6391-6400.
71. D. Y. Kim, Y. M. Lee, Y. J. Choi, "Acylation of α-fluorophosphonoacetate derivatives using magnesium chloride-triethylamine" *Tetrahedron*, **1999**, *55*, 12983-12990.
72. K. V. Tarasenko, V. D. Romanenko, A. E. Sorochinsky, "Condensation of diethyl fluoromethylphosphonate with esters: An alternative synthetic route to diethyl α-fluoro-β-ketophosphonates" *J. Fluor. Chem.*, **2018**, *211*, 124-128.
73. S. Ponra, W. Rabten, J. Yang, H. Wu, S. Kerdphon, P. G. Andersson, Diastereo- and Enantioselective Synthesis of Fluorine Motifs with Two Contiguous Stereogenic Centers" *J. Am. Chem. Soc.* **2018**, *140*, 13878-13883.
74. G. Etemad-Moghadam, J. Seyden-Penne, "Synthese stereoselective d'esters α,β-ethyleniques α-fluores E par reaction de Wittig-Horner a partir du diethyl phosphono α-fluoroacetate de methyle. Etude comparative avec le diphenyl phosphonoxy α-fluoroacetate de methyle" *Bull. Soc. Chim. Fr.* **1985**, 448-454.
75. E. Elkik, M. Imbeaux-Oudotte, "Cetones α-fluoro α-ethyleniques: preparation et reaction avec le methylure de dimethylsulfoxonium" *Tetrahedron Lett.* **1985**, *26*, 3977-3980.
76. J. Zhou, Y. Fang, F. Wang, J. Li, "Catalyst-free regioselective hydroxyfluorination and aminofluorination of α,β-unsaturated ketones" *Org. Biomol. Chem.* **2019**, *17*, 4470-4474.
77. Q. Liu, Y. Mu, T. Koengeter, R. R. Schrock, A. H. Hoveyda, "Stereodefined alkenes with a fluoro-chloro terminus as a uniquely enabling compound class" *Nature Chem.* **2022**, *14*, 463-473.
78. G. Dutheuil, C. Paturel, X. Lei, S. Couve-Bonnaire, X. Pannecoucke, "First Stereospecific Synthesis of (E)- or (Z)-α-Fluoroenones via a Kinetically Controlled Negishi Coupling Reaction" *J. Org. Chem.* **2006**, *71*, 4316-4319.
79. T. B. Patrick, T. Y. Agboka, K. Gorrell, "Heck reaction with 3-fluoro-3-buten-2-one" *J. Fluor. Chem.* **2008**, *129*, 983-985.

80. X. Song, J. Chang, D. Zhu, J. Li, C. Xu, Q. Liu, M. Wang, "Catalytic Domino Reaction of Ketones/Aldehydes with Me3SiCF2Br for the Synthesis of α-Fluoroenones/α-Fluoroenals" *Org. Lett.* **2015**, *17*, 1712-1715.
81. C. Kong, T. G. Driver, "Rh2(II)-Catalyzed Ester Migration to Afford 3H-Indoles from Trisubstituted Styryl Azides" *Org. Lett.* **2015**, *17*, 802-805.
82. J. Hierold, D. W. Lupton, "C-C bond fragmentation by Grob/Eschenmoser reactions, applications in dendrimer synthesis" *Org. Bio. Chem.* **2013**, *11*, 6150-6160.
83. J. T. Mohr, D. C. Behenna, A. M. Harned, B. M. Stoltz, "C-C bond fragmentation by Grob/Eschenmoser reactions, applications in dendrimer synthesis" *Angew. Chem. Int. Ed.* **2005**, *44*, 6924-6927.
84. A. Armstrong, B. Dominguez-Fernandez, T. Tsuchiya, "Bicyclo[3.2.1]octanone catalysts for asymmetric alkene epoxidation: the effect of disubstitution" *Tetrahedron*, **2006**, *62*, 6614-6620.
85. Y. Kageshima, C. Suzuki, K. Oshiro, H. Amii, "Highly Controlled Ring-Opening of Siloxydifluorocyclopropanes: A Versatile Route to Cyclic Fluoroketones" *Synlett*, **2015**, *26*, 63-66.
86. Chuit *et al. J. Chem. Res. Miniprint* **1977**, 1147.
87. G. Stavber, M. Zupan, S. Stavber, "Micellar-System-Mediated Direct Fluorination of Ketones in Water" *SynLett* **2009**, 589-594.
88. F. Tan, M. Pu, J. He, J. Li, J. Yang, S. Dong, X. Liu, Y-D. Wu, X. Feng, "Catalytic Asymmetric Homologation of Ketones with α-Alkyl α-Diazo Esters" *J. Am. Chem. Soc.* **2021**, *143*, 2394-2402.
89. G. Xing, Z. Zhi, C. Yi, J. Zou, X. Jing, A. Yiu-Ho Woo, B. Lin, L. Pan, Y. Zhang, M. Cheng, "8-Hydroxyquinolin-2(1H)-one analogues as potential β2-agonists: Design, synthesis and activity study" *Eur. J. Med. Chem.* **2021**, *224*, 113697.
90. Y. Feng, J. Wang, J. Yang, F. Chen, Z. Zhang, C. Ke, J. Lin, H. Lin, "Native Amino Group Directed Site-Selective ε-C(sp2)–H Iodination of Primary Amines" *Org. Lett.* **2023**, *25*, 1348-1352.
91. S. J. Wittenberger, W. R. Baker, B. G. Donner, "A diastereoselective synthesis of pseudo-C2 -symmetric 1,3-diamino-2-propanols as core units in HIV protease inhibitors" *Tetrahedron* **1993**, *49*, 1547-1556.
92. S. E. Howson, G. J. Clarkson, A. D. Faulkner, "Optically pure heterobimetallic helicates from self-assembly and click strategies" R. A. Kaner, M. J. Whitmore, P. Scott, *Dalton Trans.* **2013**, *42*, 14967-14981.
93. J. Hutchinson, G. Sandford, J. F. S. Vaughan, "Alkylation and decarboxylation of ethyl 2-fluoro-3-oxobutanoate as a route to functionalised α-fluoro-ketones" *Tetrahedron* **1998**, *54*, 2867-2876.
94. D. S. Goldfarb, J. A. Maddry, L. Rasmussen, E. L. White, K. Wennerberg, "Compounds and methods for altering lifespan of eukaryotic organisms" U.S. Patent WO201162964, **2011**.
95. T. J. Donohoe, O. Hoff, J. B. Hoffman, A. Kelly, J. C. L. Walker, S. Werrel, "A Heck reaction/photochemical alkene isomerization sequence to prepare functionalized quinolines" *Tetrahedron* **2020**, *76*, 131396.
96. M. Sawada, S. Kubo, K. Matsumura, Y. Takemoto, H. Kobayashi, E. Tashiro, T. Kitahara, H. Watanabe, M. Imoto, "Synthesis and anti-migrative evaluation of moverastin derivatives" *Bioorg. Med. Chem. Lett.* **2011**, *21*, 1385-1389.
97. E. Larionov, L. Lin, L. Gune, C. Mazet, "Scope and Mechanism in Palladium-Catalyzed Isomerizations of Highly Substituted Allylic, Homoallylic, and Alkenyl Alcohols" *J. Am. Chem. Soc.* **2014**, *136*, 16882-16894.
98. R. D. Chambers, M. Parsons, G. Sandford, E. Thomas, J. Trmcic, J. S. Moilliet, "Elemental fluorine. Part 19: Electrophilic fluorination of hexyl derivatives bearing electron withdrawing groups" *Tetrahedron* **2006**, *62*, 7162-7167.
99. R. Manikandan, R. S. Phatake, N. G. Lemcoff, "Metal-Free Photochemical Olefin Isomerization of Unsaturated Ketones via 1,5-Hydrogen Atom Transfer" *Angew. Chem. Int. Ed.* **2022**, e202200634.
100. J. Ni, X. Xia, D. Gu, Z. Wang, "Ti-Catalyzed Modular Ketone Synthesis from Carboxylic Derivatives and gem-Dihaloalkanes" *J. Am. Chem. Soc*. **2023**, *145*, 14884–14893.
101. L.-J. Cheng, C. J. Cordier, "Catalytic Nucleophilic Fluorination of Secondary and Tertiary Propargylic Electrophiles with a Copper–N-Heterocyclic Carbene Complex" *Angew. Chem. Int. Ed.* **2015**, *54*, 13734-13738.

102. L. Kollár, P. Pongrácz, “Tandem hydroformylation/aldol condensation reactions: Synthesis of unsaturated ketones from olefins” *J. Organomet. Chem*. **2018**, *866*, 184-188.
103. J. D. Griffin, M. A. Zeller, D. A. Nicewicz, “Hydrodecarboxylation of Carboxylic and Malonic Acid Derivatives via Organic Photoredox Catalysis: Substrate Scope and Mechanistic Insight” *J. Am. Chem. Soc.* **2015**, *137*, 11340–11348.
104. D. Enders, M. R. M. Hüttl, “Direct Organocatalytic a-Fluorination of Aldehydes and Ketones“ *Synlett*, **2005**, *6*, 991-993.
105. A. S. Reddy, K. K. Laali, “Mild and selective α-fluorination of carbonyl compounds (ketones, 1,3-diketones, β-ketoesters, α-nitroketones, and β-ketonitriles) with Selectfluor (F-TEDA-BF4) in imidazolium ILs [BMIM/PF6 or BMIM/NTf2] with Brønsted-acidic IL [PMIM(SO3H)/OTf] as promoter“ *Tetrahedron Lett.* **2015**, *56*, 5495-5499.
106. K. Shanab, W. Wadsak, L. Mien, M. Mitterhauser, W. Holzer, V. Polster, H. Viernstein, H. Spreitzer, “Synthesis of in vivo metabolites of the new adenosine $A_3$ receptor PET-radiotracer [$^{18}$F]Fe@suppy” *Heterocycles* **2008**, *75*, 339-356.
107. M. S. Marshall, E. S. Starchman, J. R. Vyvyan, “Synthesis of (±)-rupestines B and C by intramolecular Mizoroki-Heck cyclization” *Tetrahedron Lett.* **2020**, *61*, 151837.
108. D. Enders, S. Faure, M. Potthoff, J. Runsink, ”Diastereoselective Electrophilic Fluorination of Enantiopure α-Silylketones Using N-Fluoro-benzosulfonimide: Regio- and Enantioselective Synthesis of α-Fluoroketones” *Synthesis* **2001**, 2307-2319.
109. X. Tan, W. Zeng, J. Wen, X. Zhang, “Iridium-Catalyzed Asymmetric Hydrogenation of α-Fluoro Ketones via a Dynamic Kinetic Resolution Strategy” *Org. Lett.* **2020**, *18*, 7230-7233.
110. S. Sato, M. Yoshida, S. Hara, “Fluorination of Ketones Using Iodotoluene Difluoride” *Synthesis*, **2005**, *15*, 2602-2605.
111. J. Qian, W. Yi, M. Lv, C. Cai, “A Facile and Mild Approach for Stereoselective Synthesis of α-Fluoro-α,β-unsaturated Esters from α-Fluoro-β-keto Esters via Deacylation” *Synlett* **2015**, 127-132.
112. K. Notsu, Y. Zushi, S. Ota, T. Kawasaki-Takasuka, T. Yamazaki, “Synthetic Utilization of 2-Chloro-1,1,1,2-tetrafluoroethane” *Chem. Eur. J.* **2011**, *17*, 9200-9208.
113. K. Rousée, J. Bouillon, S. Couve-Bonnaire, X. Pannecoucke, “Stereospecific Synthesis of Tri- and Tetrasubstituted α-Fluoroacrylates by Mizoroki–Heck Reaction” *Org. Lett.* **2016**, *18*, 540-543.
114. S. Xie, X. Gao, H. Wu, F. Zhou, J. Zhou, “Direct Electrochemical Defluorinative Carboxylation of gem-Difluoroalkenes with Carbon Dioxide” *Org. Lett.* **2020**, *22*, 8424-8429.
115. F. Elkik, *Comptes Rendus des Seances de l'Academie des Sciences, Serie C: Sciences Chimiques* **1970**, *270*, 443.
116. D. Tan, E. Lin, W. Ji, Y. Zeng, W. Fan, Q. Li, H. Gao, H. Wang, “Copper-Catalyzed Stereoselective Defluorinative Borylation and Silylation of gem-Difluoroalkenes” *Adv. Syn. Catal.* **2018**, *360*, 1032-1037.
117. L. Zoute, G. Dutheuil, J. Quirion, P. Jubault, X. Pannecoucke, “Efficient Synthesis of Fluoroalkenes via Diethylzinc-Promoted Wittig Reaction” *Synthesis* **2006**, 3409-3418.
118. A. Nouaille, X. Pannecoucke, T. Poisson, S. Couve-Bonnaire, “Access to Trisubstituted Fluoroalkenes by Ruthenium-Catalyzed Cross-Metathesis” *Adv. Synth. Catal.* **2021**, *363*, 2140-2147.
119. P. Martinet, R. Sauvetre, J.-F. Normant, “Hydrogenation catalytique d'aldehydes, cetones et esters α,β-ethyleniques α-fluores et de cetones α,β-ethyleniques α,β-difluorees” *J. Fluorine Chem.* **1991**, *52*, 419-432.
120. Y. He, N. Shen, X. Fan, X. Zhang, “Synthesis of β-fluoroenones and their reductive rearrangement in aqueous media“ *Tetrahedron*, **2013**, *69*, 8818-8823.
121. C. W. Kee, K. F. Chin, M. W. Wong, C.-H. Tan, “Selective fluorination of alkyl C–H bonds via photocatalysis“ *Chem. Commun*. **2014**, *50*, 8211-8214.
122. X. Gao, K. Gong, M. Wang, B. Xu, J. Han, “Preparation of [18F]Alkenyl Fluorides Using No-Carrier-Added [18F]AgF via Silver-Mediated Direct Radiofluorination of Alkynes“ *Org. Lett.* **2022**, *24*, 6438-6442.

123. T. Zeng, C. Huang, Y. Zhang, Y. Luo, D. Niu, "C–H fluorination promoted by pyridine N-oxyl radicals" *Green Chem.* **2024**, *26*, 8701-8705.
124. R. C. McAtee, J. W. Beatty, C. C. McAtee, C. R. J. Stephenson, "Radical Chlorodifluoromethylation: Providing a Motif for (Hetero)arene Diversification" *Org. Lett.* **2018**, *20*, 3491-3495.
125. Z. Feng, B. Zhu, B. Dong, L. Cheng, Y. Li, Z. Wang, Z. Wu, "Visible-Light-Promoted Synthesis of α-CF2H-Substituted Ketones by Radical Difluoromethylation of Enol Acetates" *Org. Lett.* **2021**, *23*, 508-513.
126. M. M. Bradford, "Rapid and sensitive method for the quantitation of microgram quantities of protein utilizing the principle of protein-dye binding" *Anal. Biochem.*, **1976**, *72*, 248-254.
127. J. Liu, S. Krajangsri, J. Q. Yang, P. G. Andersson, "Iridium-catalysed asymmetric hydrogenation of allylic alcohols via dynamic kinetic resolution" *Nature Catal.* **2018**, *1*, 438-443.
128. T. Desrues, J. Merad, D. Andrei, J. M. Pons, J. L. Parrain, M. Médebielle, A. Quintard, C. Bressy, "Impact of the Difluoromethylene Group in the Organocatalyzed Acylative Kinetic Resolution of α,α-Difluorohydrins" *Angew. Chem. Int. Ed.* **2021**, *60*, 24924-24929.
129. D. S. Stephenson, G. Binsch. G. "Automated analysis of high-resolution NMR spectra. I. Principles and computational strategy" *J. Magn. Reson.* **1980**, *37*, 395-407.
130. gNMR, version 5.0.6. NMR Simulation Program by Budzelaar, P. H. M. **2006**.
131. K. E. Gilbert, J. J. Gajewski, PCMODEL version 8.5, Molecular Modeling Software for Windows Operating System, Apple Macintosh OS, Linux, Unix. Serena Software.
132. GaussView, Version 6, R. Dennington, T. Keith, J. Millam, Semichem Inc., Shawnee Mission, KS, **2016**.
133. Gaussian 16, Revision A.03, M. J. Frisch, G. W. Trucks, H. B. Schlegel, G. E. Scuseria, M. A. Robb, J. R. Cheeseman, G. Scalmani, V. Barone, G. A. Petersson, H. Nakatsuji, X. Li, M. Caricato, A. V. Marenich, J. Bloino, B. G. Janesko, R. Gomperts, B. Mennucci, H. P. Hratchian, J. V. Ortiz, A. F. Izmaylov, J. L. Sonnenberg, D. Williams-Young, F. Ding, F. Lipparini, F. Egidi, J. Goings, B. Peng, A. Petrone, T. Henderson, D. Ranasinghe, V. G. Zakrzewski, J. Gao, N. Rega, G. Zheng, W. Liang, M. Hada, M. Ehara, K. Toyota, R. Fukuda, J. Hasegawa, M. Ishida, T. Nakajima, Y. Honda, O. Kitao, H. Nakai, T. Vreven, K. Throssell, J. A. Montgomery, J. E. Peralta, F. Ogliaro, M. J. Bearpark, J. J. Heyd, E. N Brothers, K. N. Kudin, V. N. Staroverov, T. A. Keith, R. Kobayashi, J. Normand, K. Raghavachari, A. P. Rendell, J. C. Burant, S. S. Iyengar, J. Tomasi, M. Cossi, J. M. Millam, M. Klene, C. Adamo, R. Cammi, J. W. Ochterski, R. L. Martin, K. Morokuma, O. Farkas, J. B.Foresman, D. J. Fox, D. J. Gaussian, Inc., Wallingford CT, **2016**.
134. A. E. Aliev, L. Nunar, "Quantification of the Strength of π-Noncovalent Interactions in Molecular Balances using Density Functional Methods" *Chemistry - Methods*, **2023**, *3*, e202200044.
135. Y. Zhao, D. G. Truhlar, "Design of Density Functionals That Are Broadly Accurate for Thermochemistry, Thermochemical Kinetics, and Nonbonded Interactions" *J. Phys. Chem. A* **2005**, *109*, 5656-5667.
136. J.-D. Chai, M. Head-Gordon, "Long-range corrected hybrid density functionals with damped atom–atom dispersion corrections" *Phys. Chem. Chem. Phys.* **2008**, *10*, 6615-6620.
137. F. Weigend, R. Ahlrichs, "Balanced basis sets of split valence, triple zeta valence and quadruple zeta valence quality for H to Rn: Design and assessment of accuracy" *Phys. Chem. Chem. Phys.* **2005**, *7*, 3297-3305.
138. F. Weigend, "Accurate Coulomb-fitting basis sets for H to Rn" *Phys. Chem. Chem. Phys.* **2006**, *8*, 1057-1065.
139. I. M. Alecu, J. Zheng, Y. Zhao, D. G. Truhlar, "Computational Thermochemistry: Scale Factor Databases and Scale Factors for Vibrational Frequencies Obtained from Electronic Model Chemistries" *J. Chem. Theory Comput*. **2010**, *6*, 2872-2887.
140. E. Cances, B. Mennucci, "New applications of integral equations methods for solvation continuum models: ionic solutions and liquid crystals" *J. Math. Chem.* **1998**, *23*, 309-326.
141. M. Cossi, N. Rega, G. Scalmani, V. Barone, "Energies, structures, and electronic properties of molecules in solution with the C-PCM solvation model" *J. Comp. Chem.* **2003**, *24*, 669-681.

142. A. S. Dumon, H. S. Rzepa, C. Alamillo-Ferrer, J. Bures, R. Procter, T. S. Sheppard, A. Whiting, "A computational tool to accurately and quickly predict $^{19}F$ NMR chemical shifts of molecules with fluorine-carbon and fluorine-boron bonds" *Phys. Chem. Chem. Phys.*, **2022**, 24, 20409-20425.
143. H. W. Adolph, P. Maurer, H. Schneider-Bernlöhr, C. Sartorius, C. M. Zeppezauer, "Substrate specificity and stereoselectivity of horse liver alcohol dehydrogenase" *Eur. J. Biochem.* **1991**, *201*, 615-625.
144. S. Reich, H. Wolfgang Hoeffken, B. Rosche, B. M. Nestl, B. Hauer, "Crystal Structure Determination and Mutagenesis Analysis of the Ene Reductase NCR" *ChemBioChem*, **2012**, *13*, 2400-2407.
145. J. Jumper, R. Evans, A. Pritzel, T. Green, M. Figurnov, O. Ronneberger, K. Tunyasuvunakool, R. Bates, A. Žídek, A. Potapenko, A. Bridgland, C. Meyer, S. A. A. Kohl, A. J. Ballard, A. Cowie, B. Romera-Paredes, S. Nikolov, R. Jain, J. Adler, T. Back, S. Petersen, D. Reiman, E. Clancy, M. Zielinski, M. Steinegger, M. Pacholska, T. Berghammer, S. Bodenstein, D. Silver, O. Vinyals, A. W. Senior, K. Kavukcuoglu, P. Kohli, D. Hassabis, "Highly accurate protein structure prediction with AlphaFold" *Nature*, **2021**, *596*, 583-589.
146. E. F. Pettersen, T. D. Goddard, C. C. Huang, G. S. Couch, D. M. Greenblatt, E. C. Meng, T. E. J. Ferrin, "UCSF Chimera--a visualization system for exploratory research and analysis" *Comput Chem.* **2004**, *13*, 1605-1612.
147. G. M. Morris, R. Huey, W. Lindstrom, M. F. Sanner, R. K. Belew, D. S. Goodsell, A. J. Olson, "AutoDock4 and AutoDockTools4: Automated docking with selective receptor flexibility" *J. Comp. Chem.* **2009**, *30*, 2785-2791.
148. M. D. Hanwell, D. E. Curtis, D. C. Lonie, T. Vandermeersch, E. Zurek, G. R. Hutchison, "Avogadro: An advanced semantic chemical editor, visualization, and analysis platform" *J. Cheminformatics*, **2012**, *4*, 17.
149. N. Ahlstena, B. Martín-Matute, "Ir-catalysed formation of C−F bonds. From allylic alcohols to α-fluoroketones" *Chem. Commun.* **2011**, *47*, 8331-8333.
150. R. Peverati, D. G. Truhlar, "M11-L: A Local Density Functional That Provides Improved Accuracy for Electronic Structure Calculations in Chemistry and Physics" *J. Phys. Chem. Lett*. **2012**, *3*, 117-124.

# HPLC and GC Traces

## 3-Fluoro-4-phenylbutan-2-one (7a)

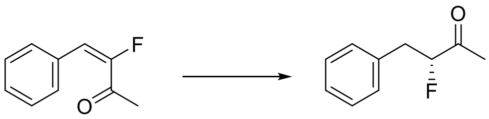


(*E*)-**1a** (*R*)-**7a**
>99% yield, 97:3 er

| Compound | $t_R$ |
|---|---|
| (*E*)-**1a** | 5.1 min |
| (*R*)-**7a** | 5.5 min |
| (*S*)-**7a** | 6.0 min |

**Conditions:** HPLC, 1% IPA/hexane, 1 mL/min$^{-1}$, 214 nm, AD-H column.

Starting Material:

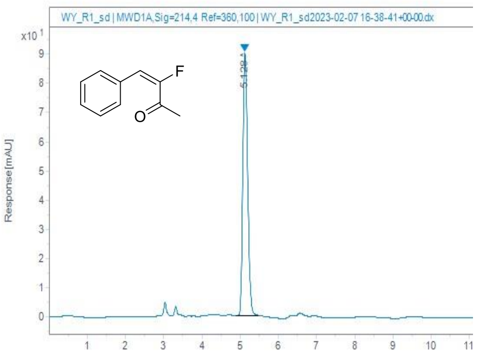


Racemic Standard:

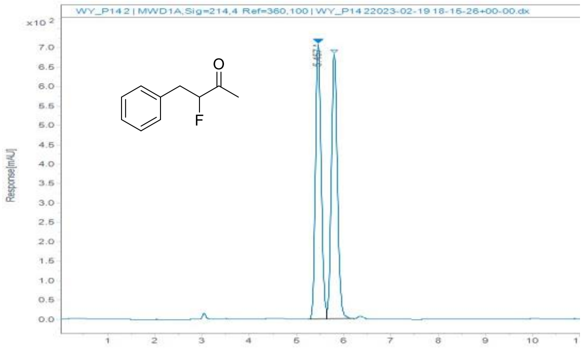


Reaction Trace:

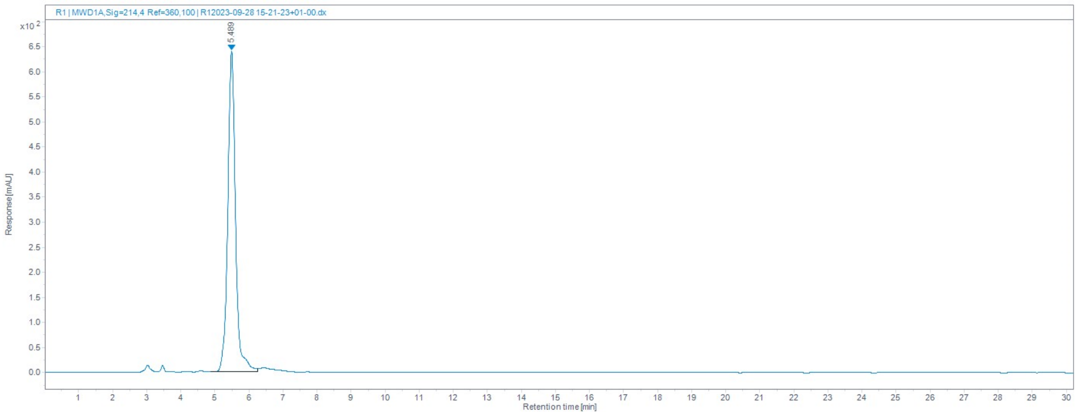


Calibration Curves:

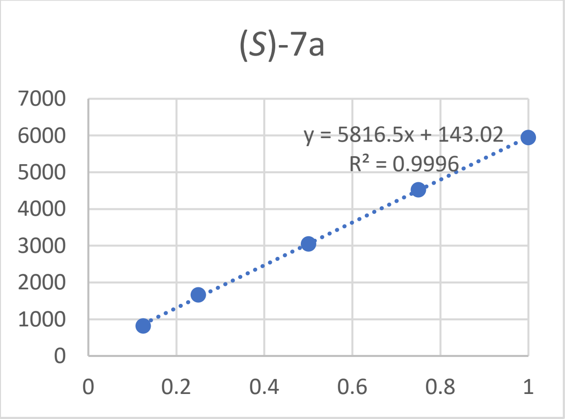


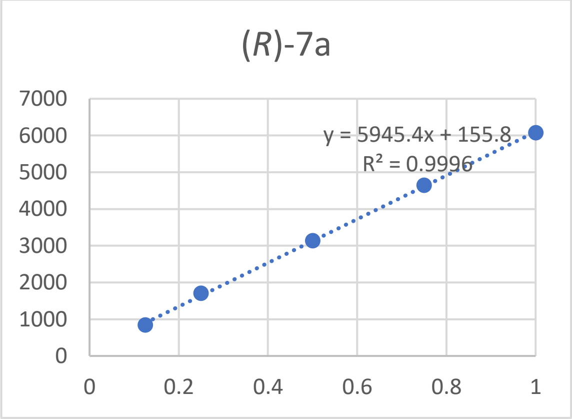

*3-Fluoro-4-phenylbutan-2-one* (**7a**)

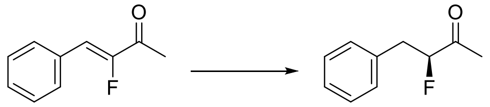


(*Z*)-**1a** (*S*)-**7a** 98% yield, 99:1 er

| Compound | $t_R$ |
|---|---|
| (*Z*)-**1a** | 6.2 min |
| (*R*)-**7a** | 5.5 min |
| (*S*)-**7a** | 6.0 min |

**Conditions:** HPLC, 1% IPA/hexane, 1 mL/min$^{-1}$, 214 nm, AD-H column.

Starting Material:

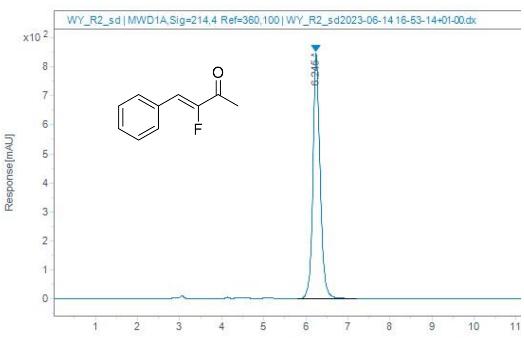


Racemic Standard:

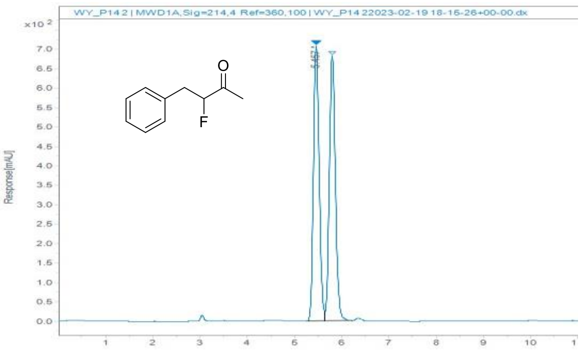


Reaction Trace:

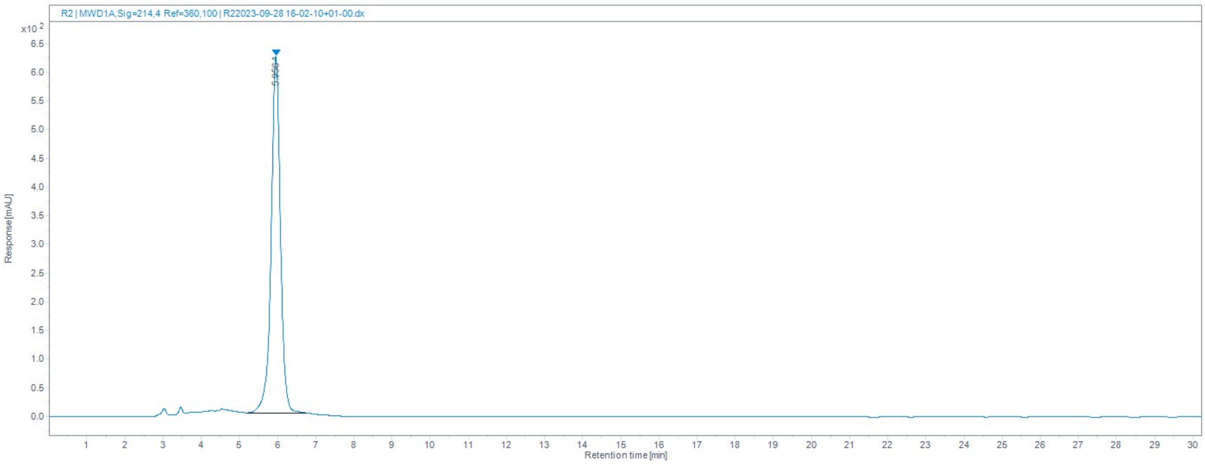


Calibration Curves:

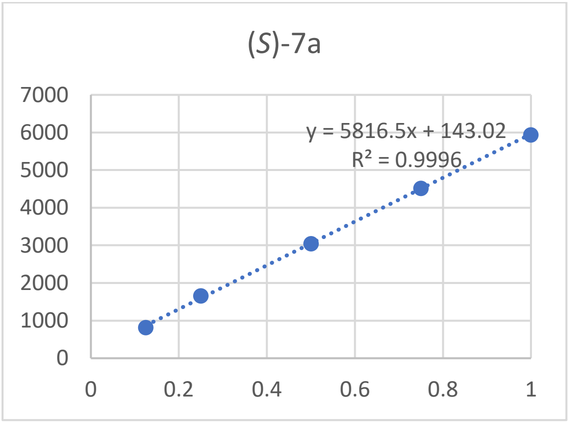


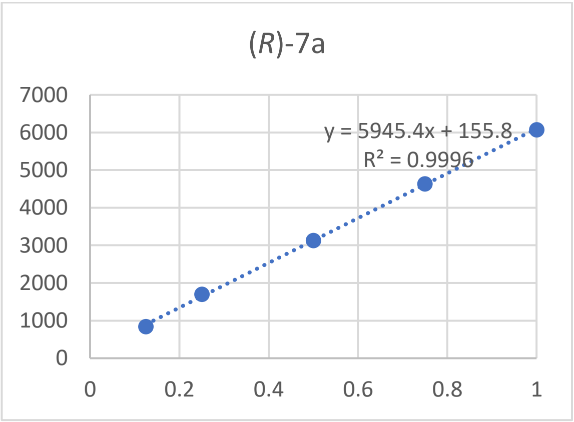

## 3-Fluoro-4-(4-trifluoromethylphenyl)-butan-2-one (7b)

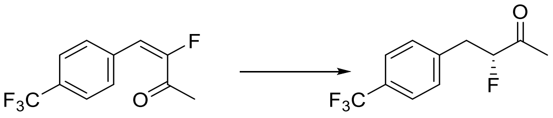

(*E*)-**1b** → (*R*)-**7b**, >99% yield, 95:5 er

| Compound | $t_R$ |
|---|---|
| (*E*)-**1b** | 5.1 min |
| (*R*)-**7b** | 5.7 min |
| (*S*)-**7b** | 6.1 min |

**Conditions:** HPLC, 1% IPA/hexane, 1 mL/min$^{-1}$, 214 nm, AD-H column.

Starting Material:

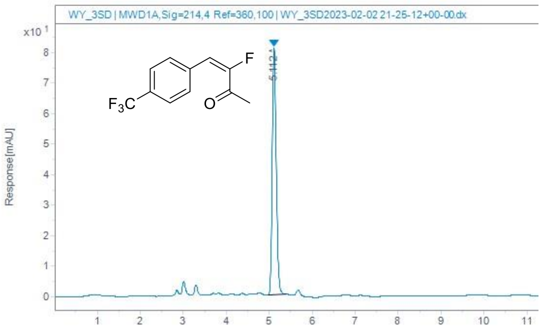


Racemic Standard:

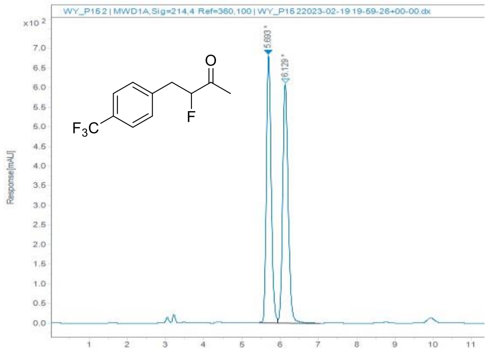


Reaction Trace:

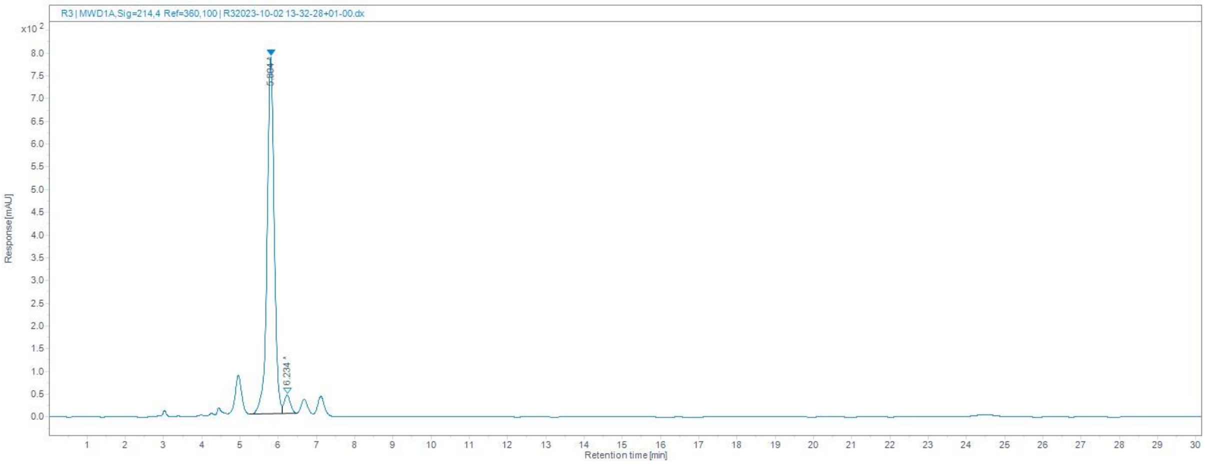


Calibration Curves:

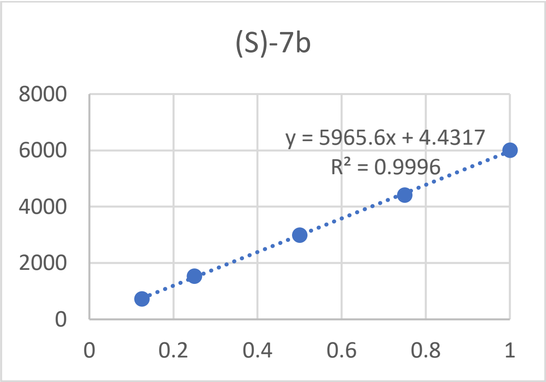


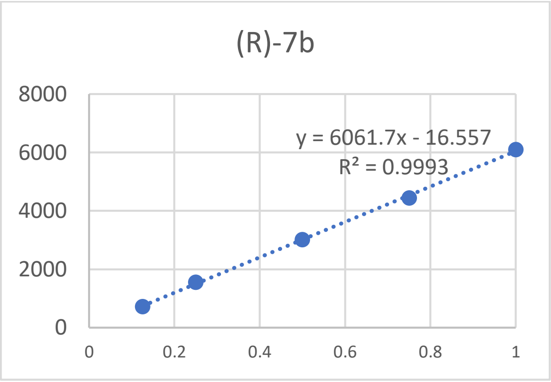

*3-Fluoro-4-(4-trifluoromethylphenyl)-butan-2-one* (**2b**)

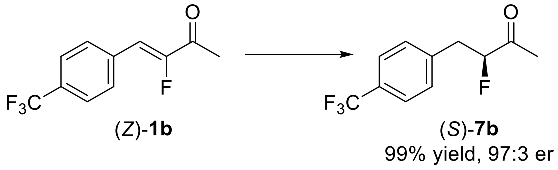


| Compound | $t_R$ |
|---|---|
| (*Z*)-**1b** | 6.4 min |
| (*R*)-**7b** | 5.7 min |
| (*S*)-**7b** | 6.1 min |

**Conditions:** HPLC, 1% IPA/hexane, 1 mL/min$^{-1}$, 214 nm, AD-H column.

Starting Material:

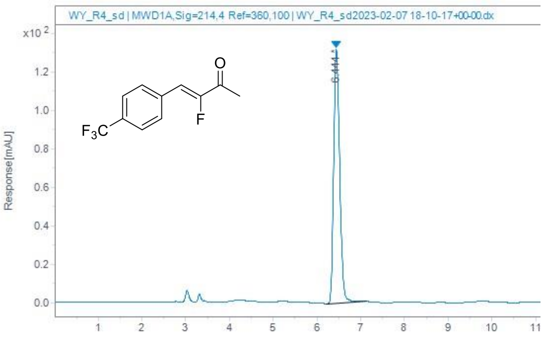


Racemic Standard:

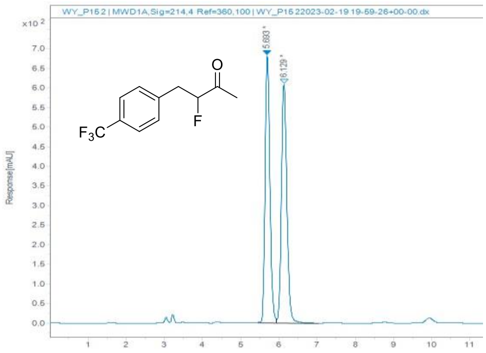


Reaction Trace:

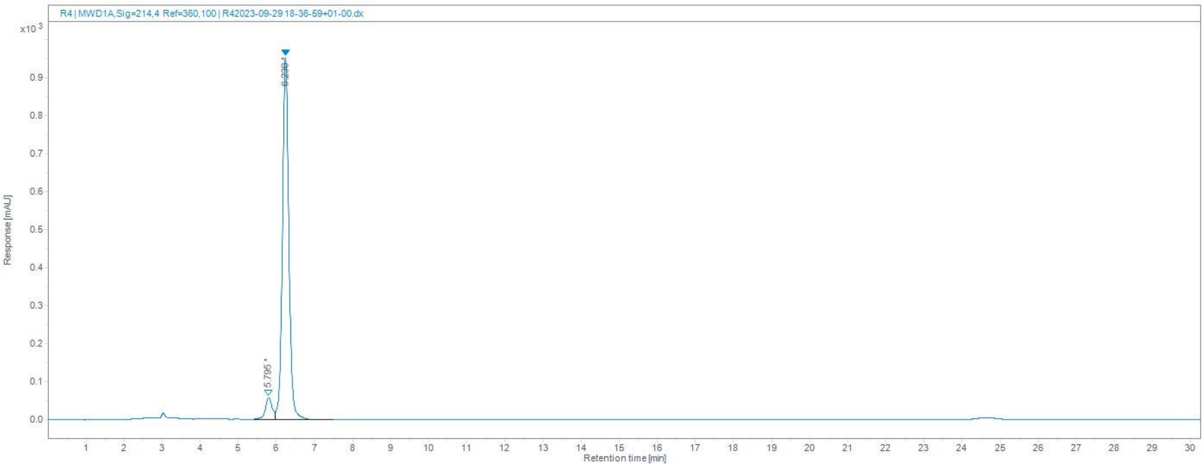


Calibration Curves:

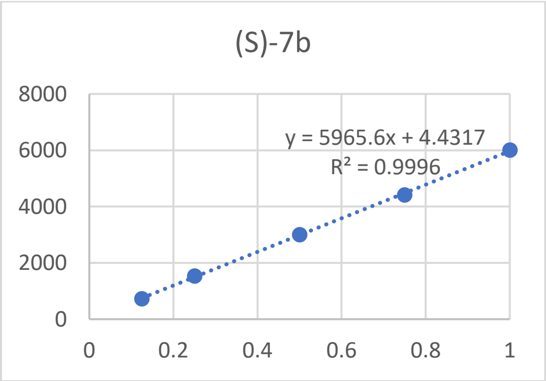


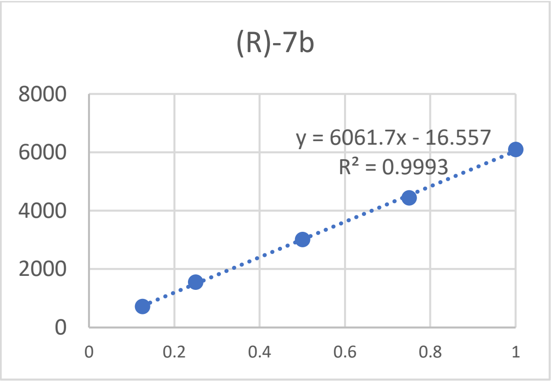

## 3-Fluoro-4-(4-methoxyphenyl)-butan-2-one (7c)

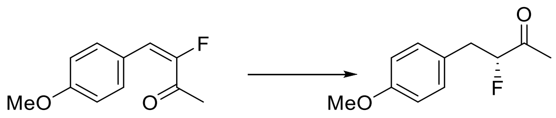


(*E*)-**1c** (*R*)-**7c**
>99% yield, 99:1 er

| Compound | $t_R$ |
|---|---|
| (*E*)-**1c** | 8.3 min |
| (*R*)-**7c** | 7.6 min |
| (*S*)-**7c** | 8.2 min |

**Conditions:** HPLC, 1% IPA/hexane, 1 mL/min$^{-1}$, 214 nm, AD-H column.

Starting Material:

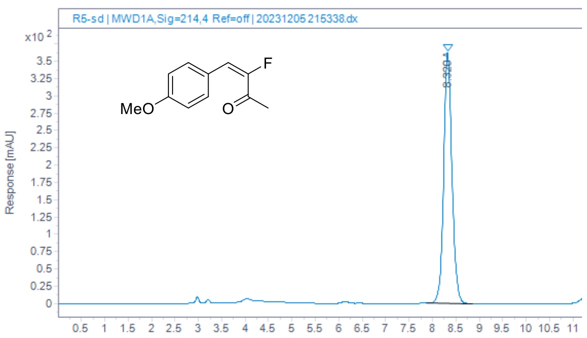


Racemic Standard:

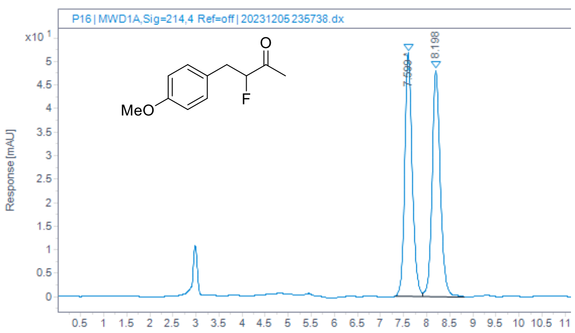


Reaction Trace:

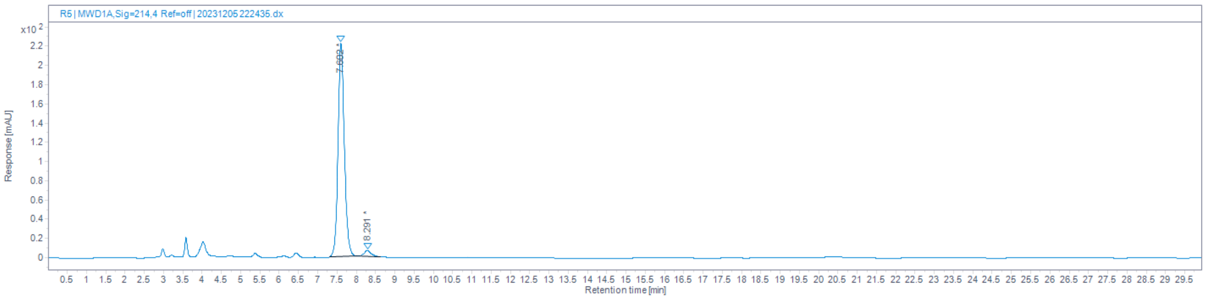


Calibration Curves:

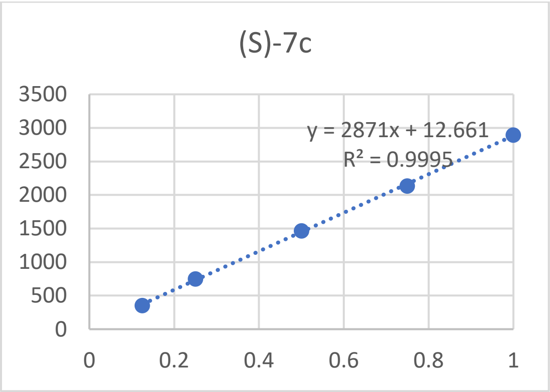


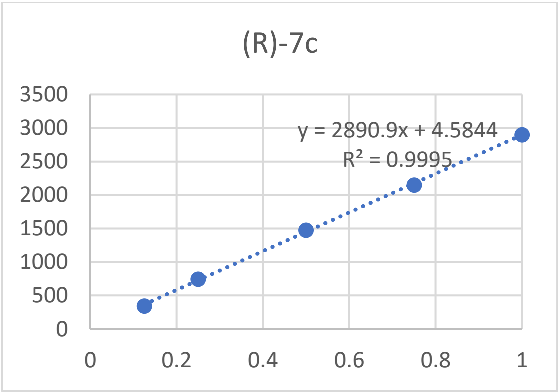

*3-Fluoro-4-(4-methoxyphenyl)-butan-2-one* (**7c**)

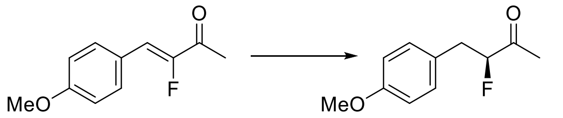


(*Z*)-**1c** (*S*)-**7c**
98% yield, 95:5 er

| Compound | $t_R$ |
|---|---|
| (*Z*)-**1c** | 11.2 min |
| (*R*)-**7c** | 7.6 min |
| (*S*)-**7c** | 8.2 min |

**Conditions:** HPLC, 1% IPA/hexane, 1 mL/min$^{-1}$, 214 nm, AD-H column.

Starting Material:

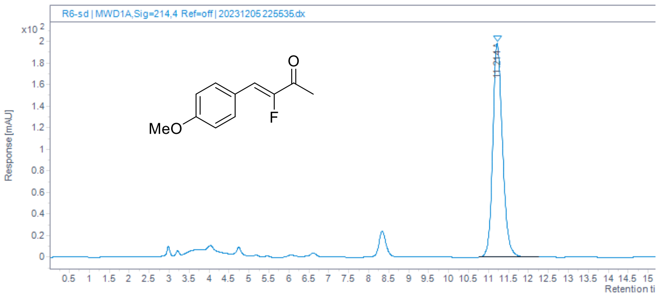


Racemic Standard:

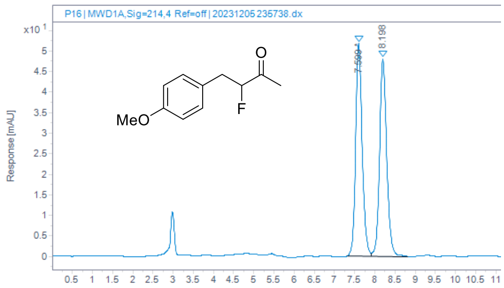


Reaction Trace:

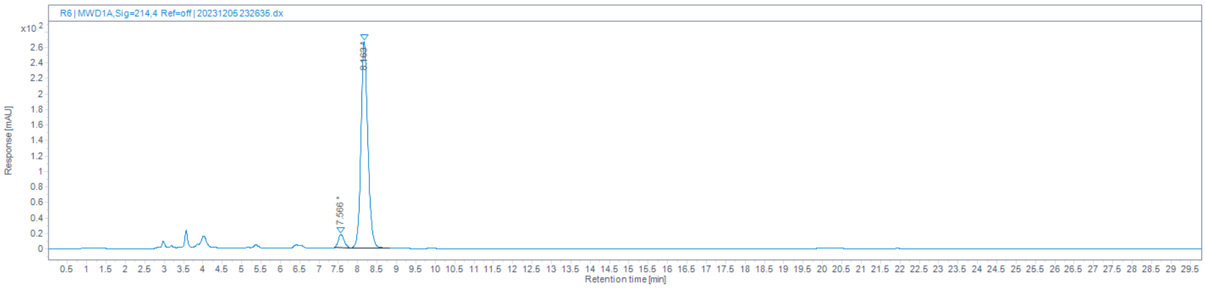


Calibration Curves:

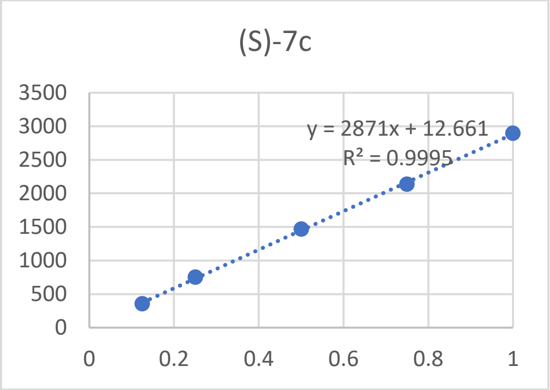


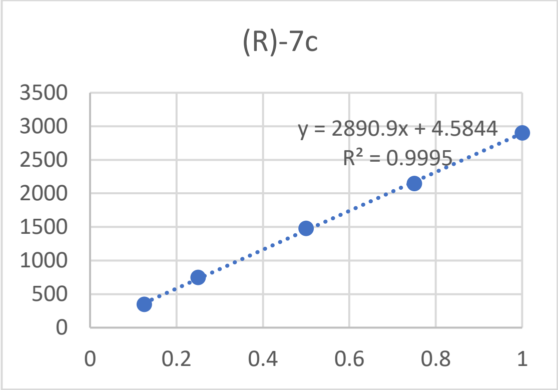

# 3-Fluoro-4-(4-cyanophenyl)-butan-2-one (7d)

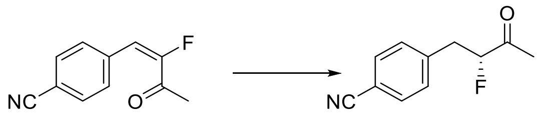


(*E*)-**1d** (*R*)-**7d**
>99% yield, 98:2 er

| Compound | $t_R$ |
|---|---|
| (*E*)-**1d** | 16.7 min |
| (*R*)-**7d** | 21.7 min |
| (*S*)-**7d** | 23.7 min |

**Conditions:** HPLC, 1% IPA/hexane, 1 mL/min$^{-1}$, 214 nm, AD-H column.

Starting Material:

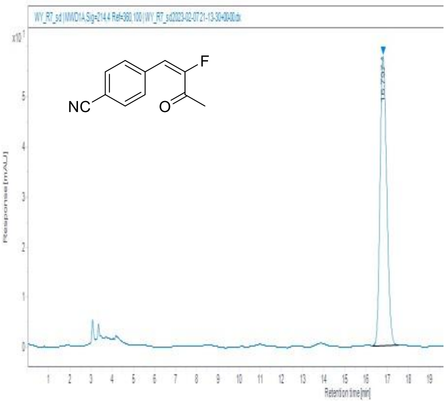

Racemic Standard:

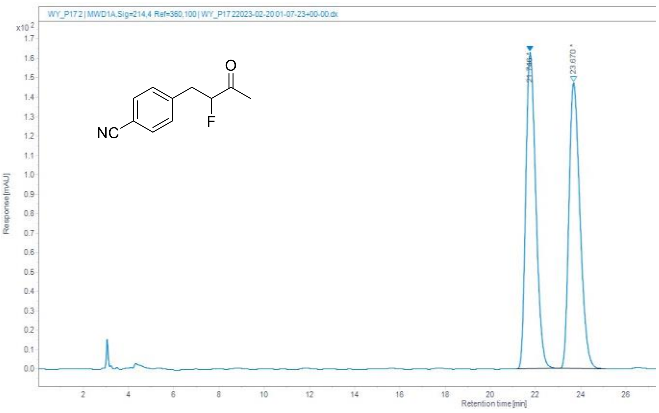


Reaction Trace:

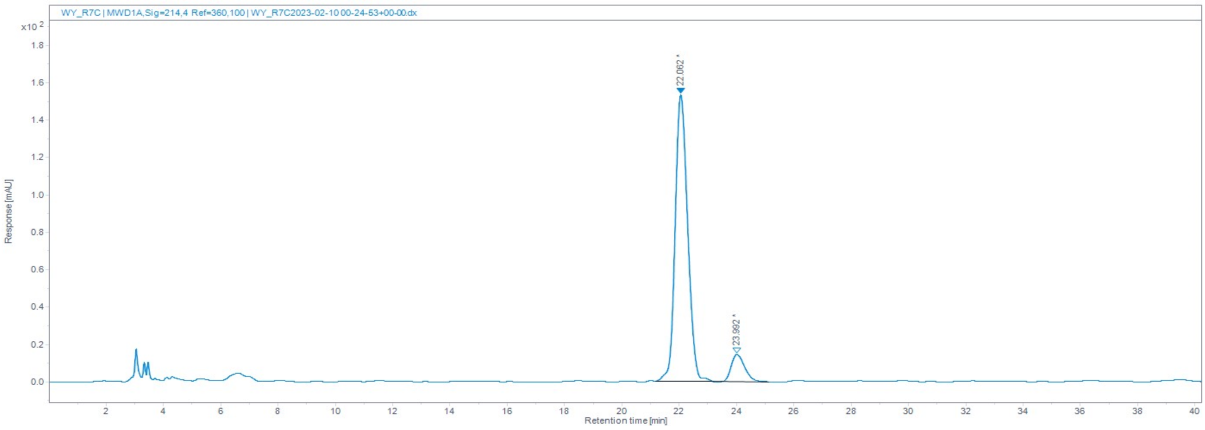


Calibration Curves:

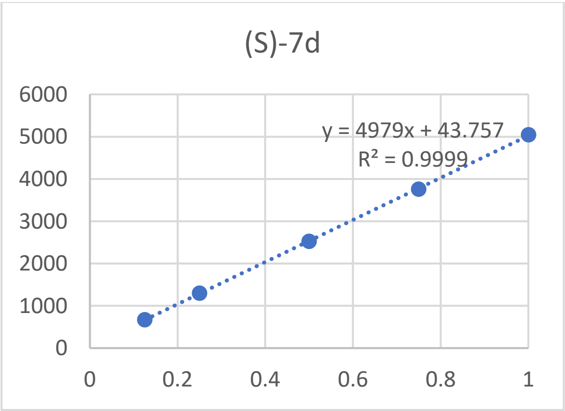


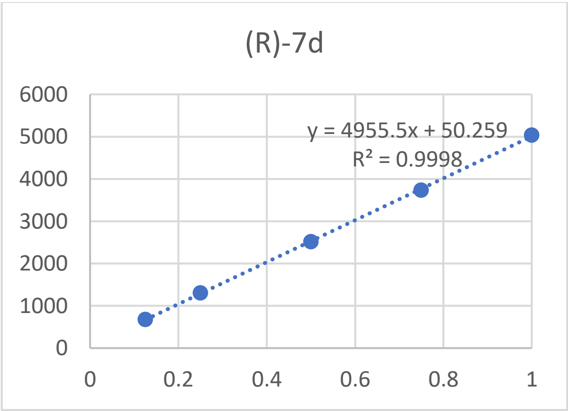

*3-Fluoro-4-(4-cyanophenyl)-butan-2-one* (**7d**)

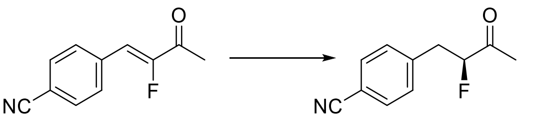


(*Z*)-**1d** (*S*)-**7d**

97% yield, 99:1 er

| Compound | $t_R$ |
|---|---|
| (*Z*)-**1d** | 22.7 min |
| (*R*)-**7d** | 21.7 min |
| (*S*)-**7d** | 23.7 min |

**Conditions:** HPLC, 1% IPA/hexane, 1 mL/min$^{-1}$, 214 nm, AD-H column.

Starting Material:

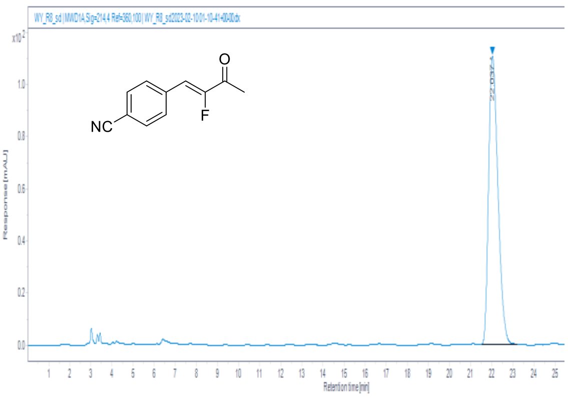


Racemic Standard:

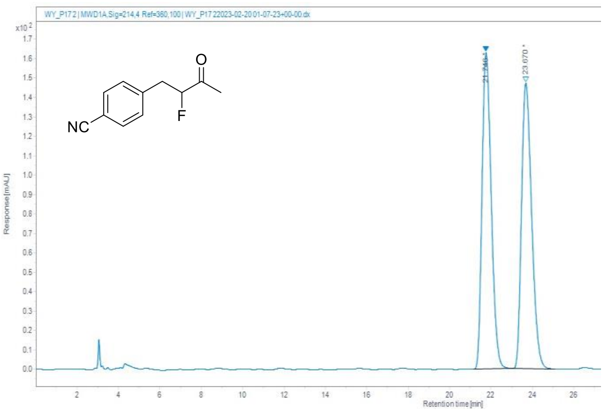


Reaction Trace:

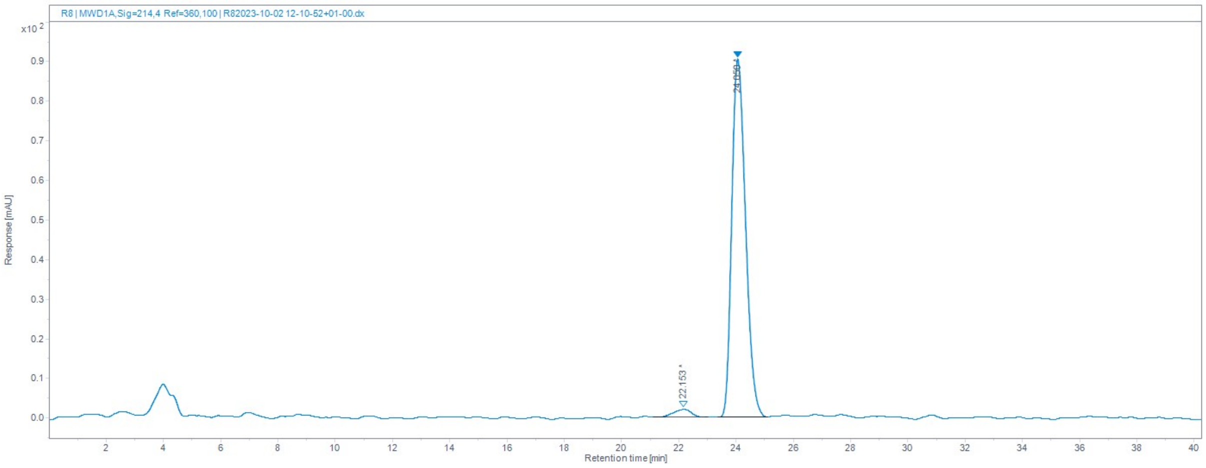


Calibration Curves:

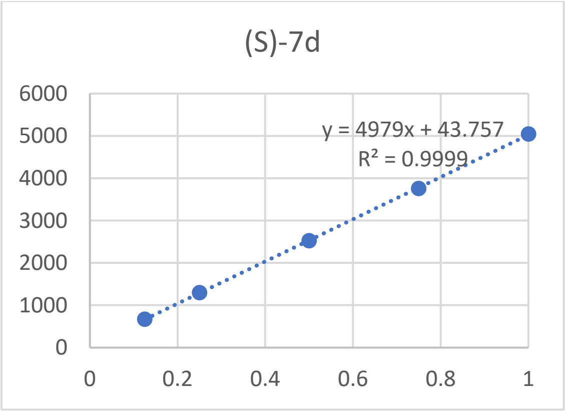


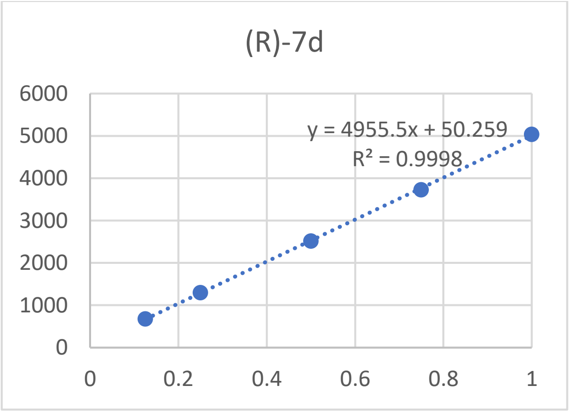

## 3-Fluoro-4-(o-tolyl)butan-2-one (7e)

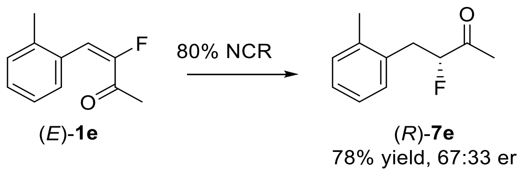


| Compound | $t_R$ |
|---|---|
| (*E*)-**1e** | 6.6 min |
| (*R*)-**7e** | 7.2 min |
| (*S*)-**7e** | 7.6 min |

**Conditions:** HPLC, 1% IPA/hexane, 1 mL/min$^{-1}$, 214 nm, OD-H column.

Starting Material:

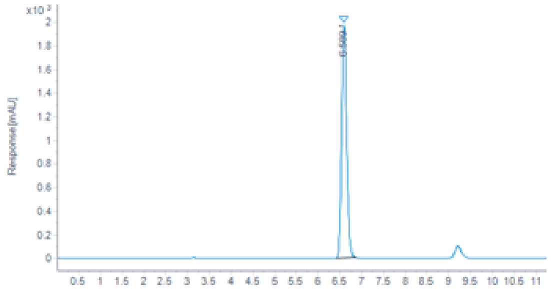


Racemic Standard:

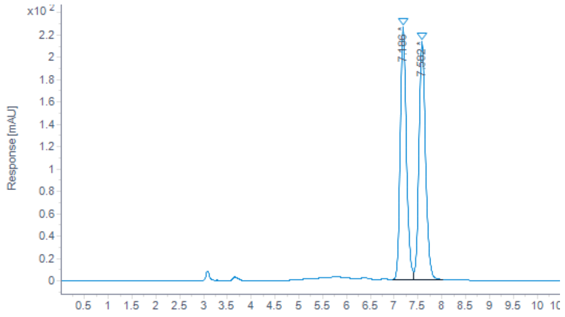


Reaction Trace:

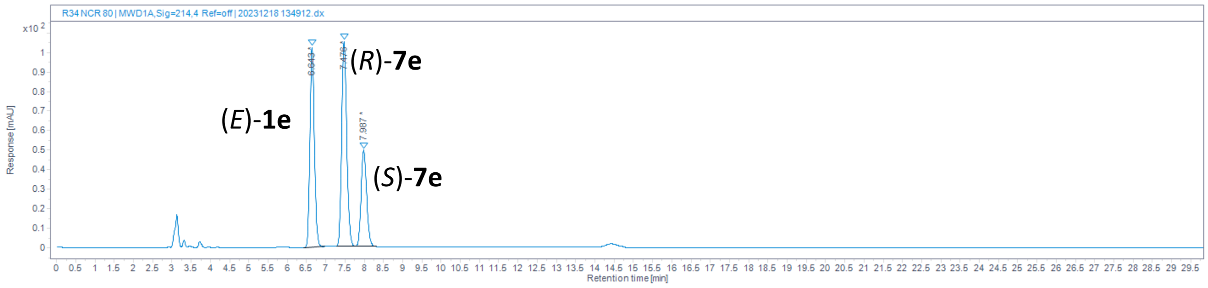


Calibration Curves:

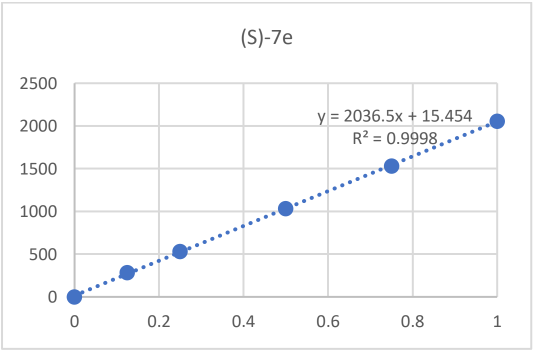


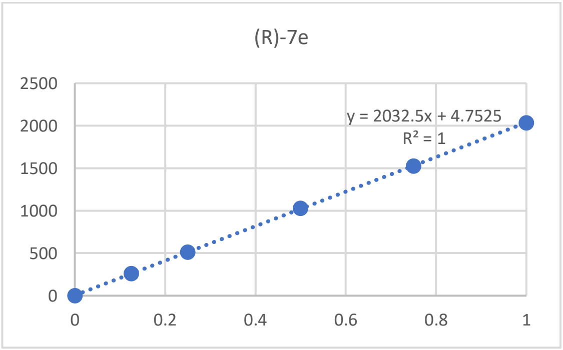

3-Fluoro-4-(o-tolyl)butan-2-one (**7e**) – Engineered Mutant

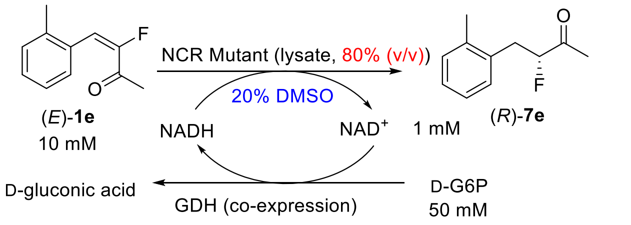


| Compound | $t_R$ |
|---|---|
| (*E*)-**1e** | 17.8 min |
| (*R*)-**7e** | 17.1 min |
| (*S*)-**7e** | 17.0 min |

**Conditions:** GC, Supelco Beta Dex 225 capillary GC column (30 m × 250 µm, 0.25 µm), method: 50 °C (hold 3 min), 50 °C to 170 °C (5 °C/min), 170 °C (hold 1 min).

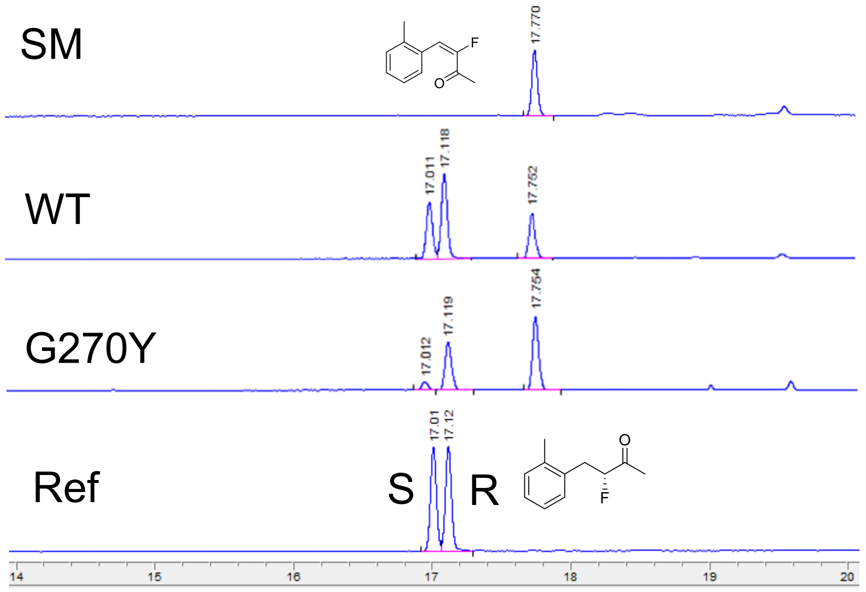

*3-Fluoro-4-(o-tolyl)butan-2-one* (**7e**)

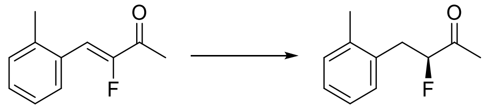

(*Z*)-**1e** (*S*)-**7e**
99% yield, 97:3 er

| Compound | $t_R$ |
|---|---|
| (*Z*)-**1e** | 9.2 min |
| (*R*)-**7e** | 7.2 min |
| (*S*)-**7e** | 7.6 min |

**Conditions:** HPLC, 1% IPA/hexane, 1 mL/min$^{-1}$, 214 nm, OD-H column. pQR1907.

Starting Material:

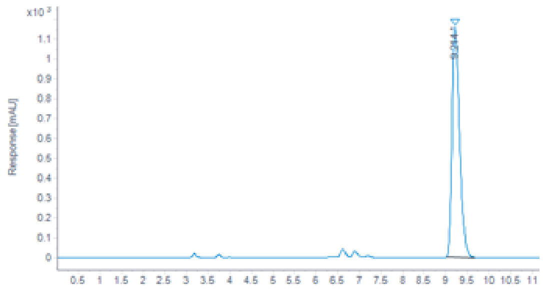


Racemic Standard:

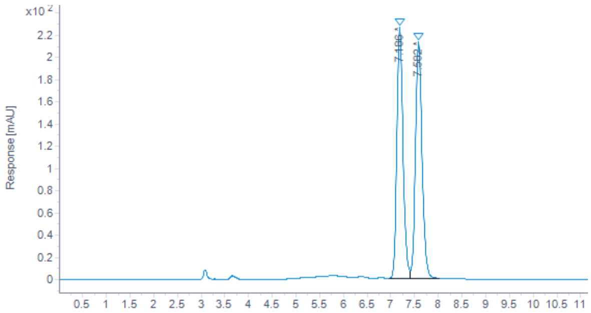


Reaction Trace:

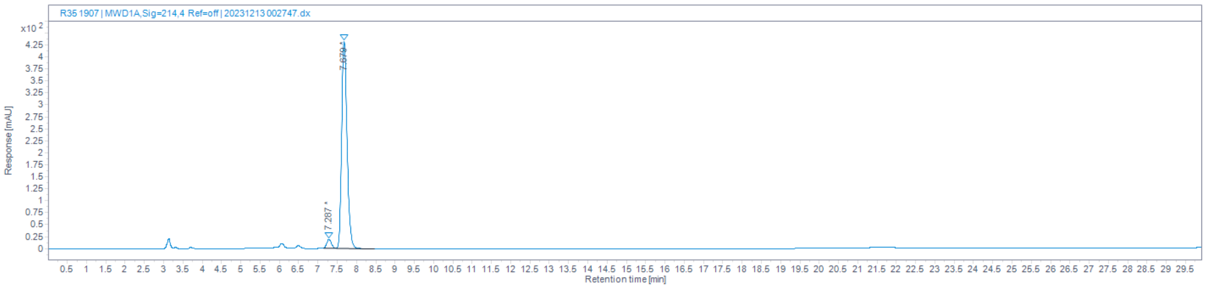


Calibration Curves:

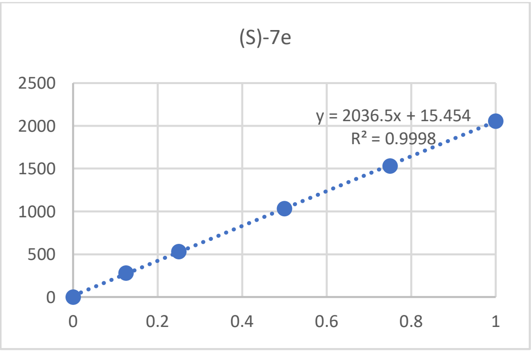


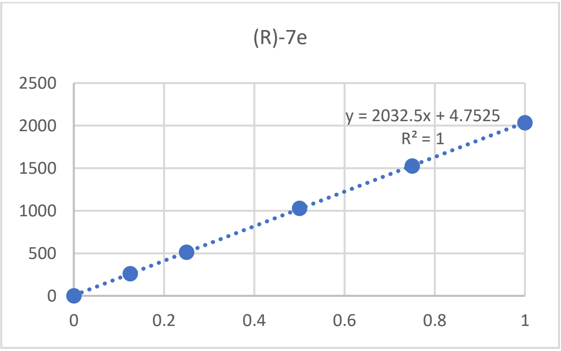

## 3-Fluoro-4-(3-trifluoromethylphenyl)-butan-2-one (7f)

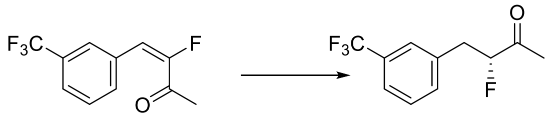

(*E*)-**1f**

(*R*)-**7f**
>99% yield, 99:1 er

| Compound | $t_R$ |
|---|---|
| (*E*)-**1f** | 8.5 min |
| (*R*)-**7f** | 10.0 min |
| (*S*)-**7f** | 10.4 min |

**Conditions:** HPLC, 1% IPA/hexane, 1 mL/min$^{-1}$, 214 nm, OJ-H column.

Starting Material:

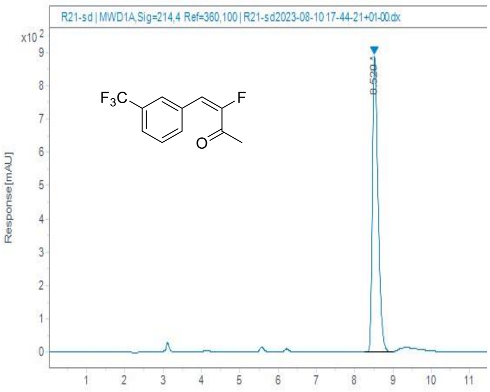


Racemic Standard:

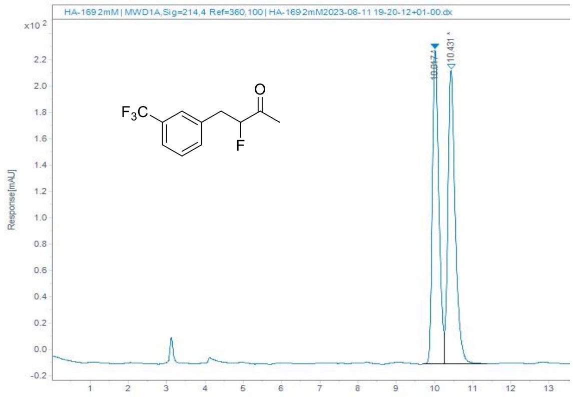


Reaction Trace:

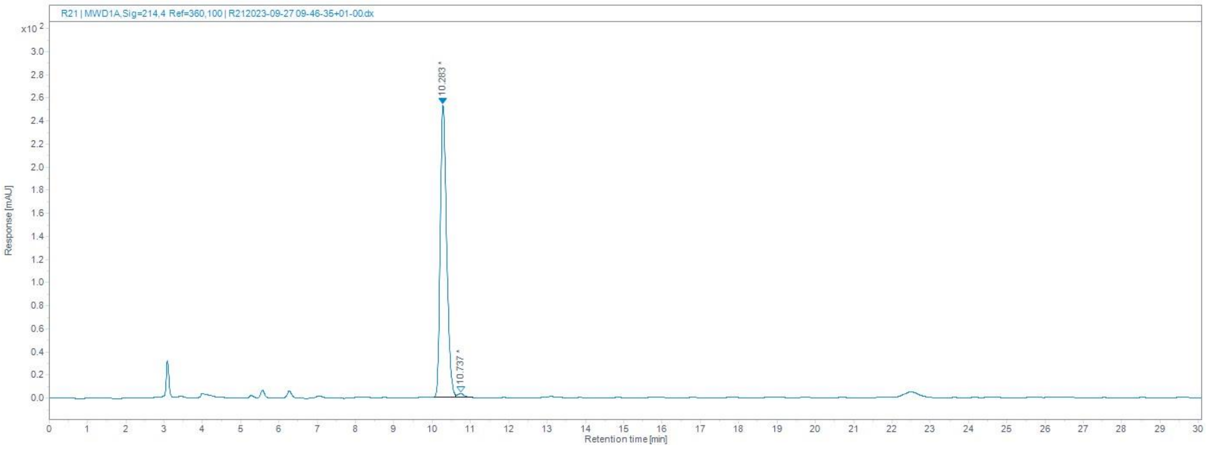


Calibration Curves:

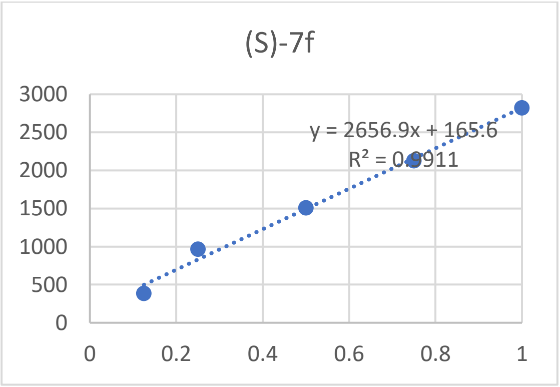


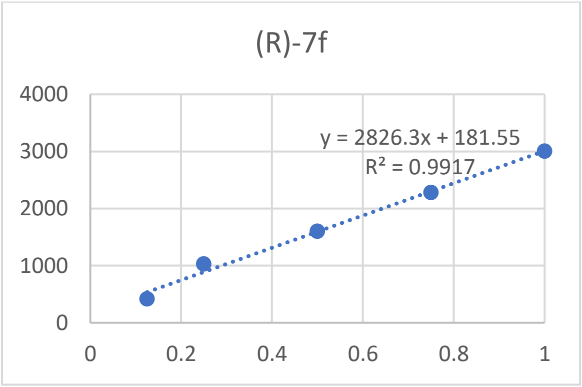

*3-Fluoro-4-(3-trifluoromethylphenyl)-butan-2-one* (**7f**)

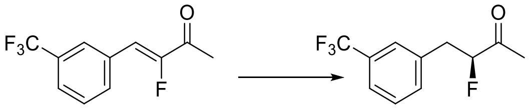


(*Z*)-**1f** (*S*)-**7f**
98% yield, 98:2 er

| Compound | $t_R$ |
|---|---|
| (*Z*)-**1f** | 10.3 min |
| (*R*)-**7f** | 10.0 min |
| (*S*)-**7f** | 10.4 min |

**Conditions:** HPLC, 1% IPA/hexane, 1 mL/min$^{-1}$, 214 nm, OJ-H column.

Starting Material:

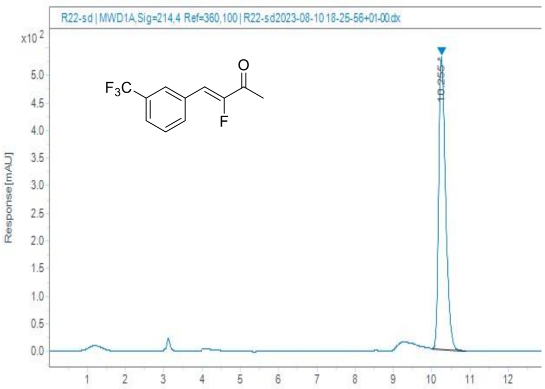


Racemic Standard:

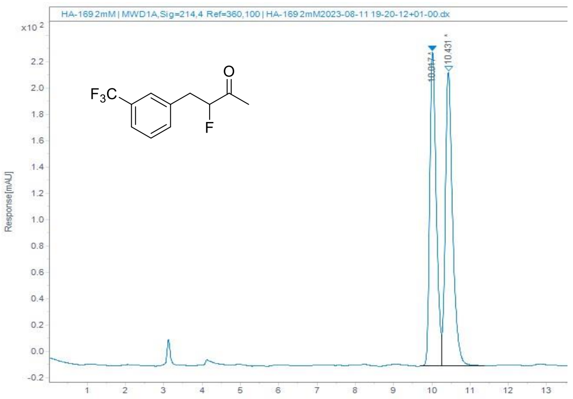


Reaction Trace:

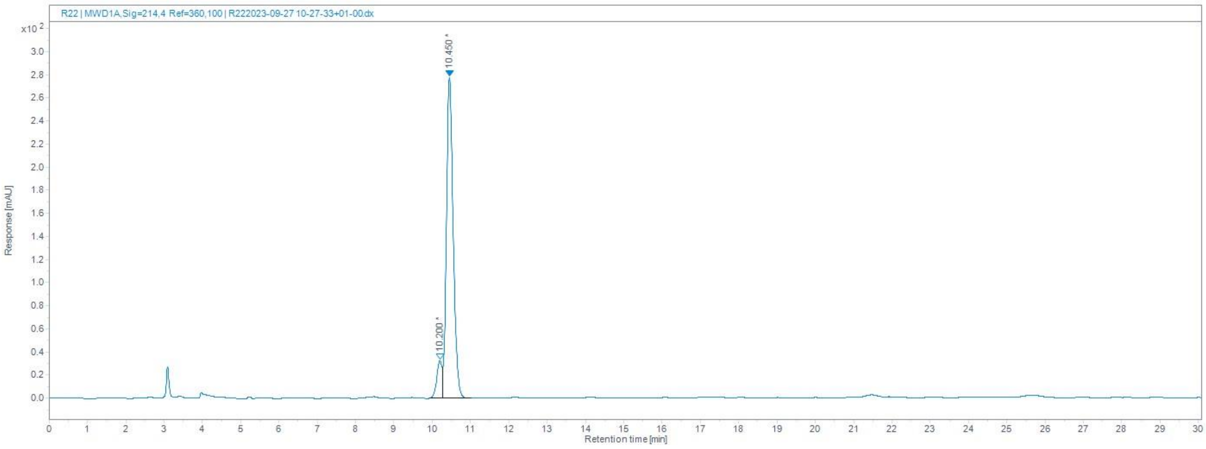


Calibration Curves:

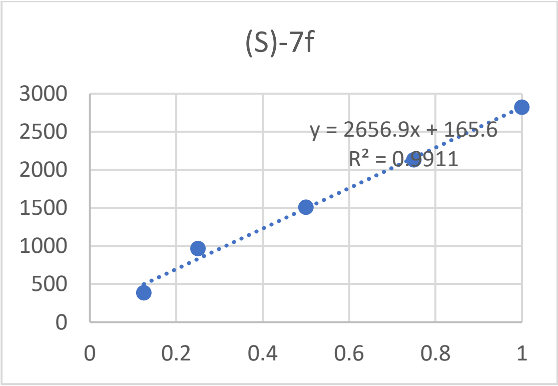


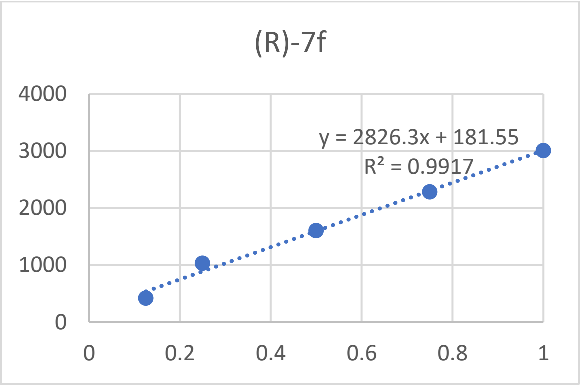

# 3-Fluoro-4-(pyridin-3-yl)butan-2-one (8a)

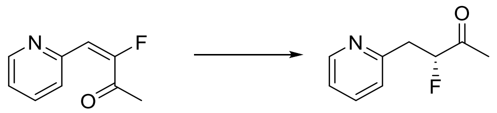

(*E*)-**2a**

(*R*)-**8a**
40% yield, 90:10 er (80% NCR)
94% yield, 88:12 er (20% pQR1445)

| Compound | $t_R$ |
|---|---|
| (*E*)-**2a** | 9.7 min |
| (*R*)-**8a** | 11.5 min |
| (*S*)-**8a** | 12.3 min |

**Conditions:** HPLC, 10% IPA/hexane, 1 mL/min$^{-1}$, 214 nm, OJ-H column.

Starting Material:

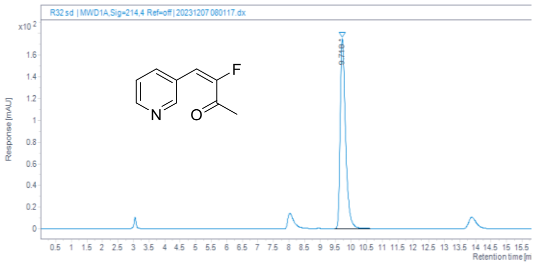


Racemic Standard:

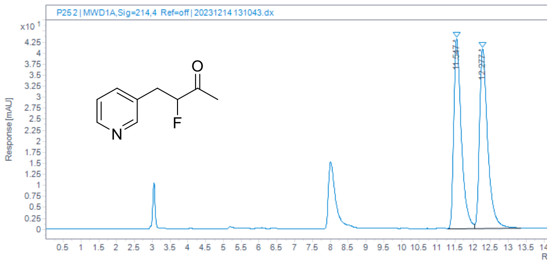


Reaction Trace:

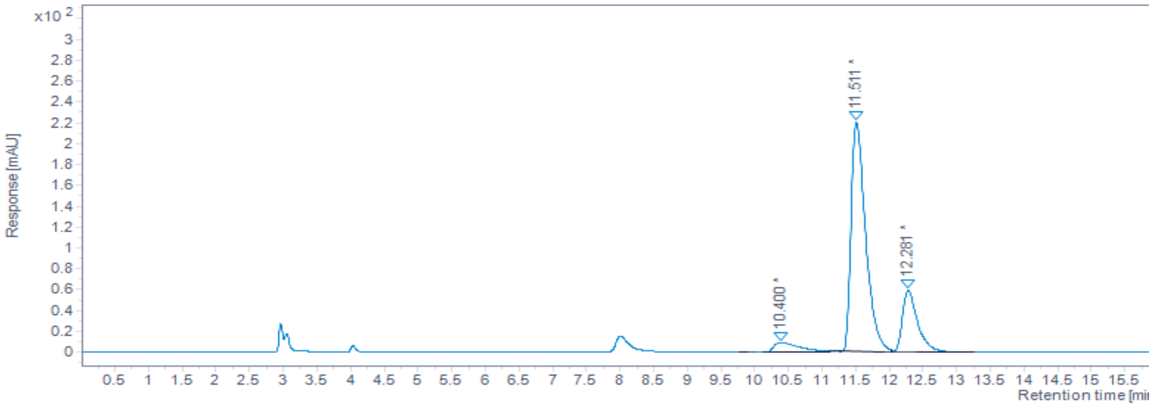


Calibration Curves:

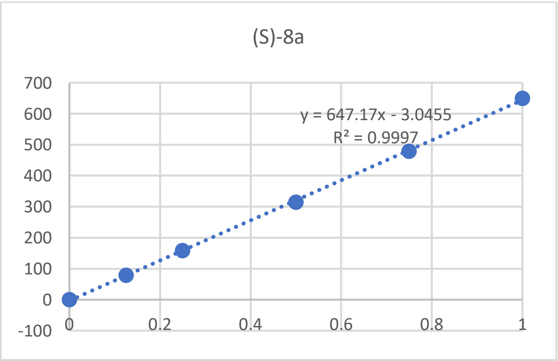


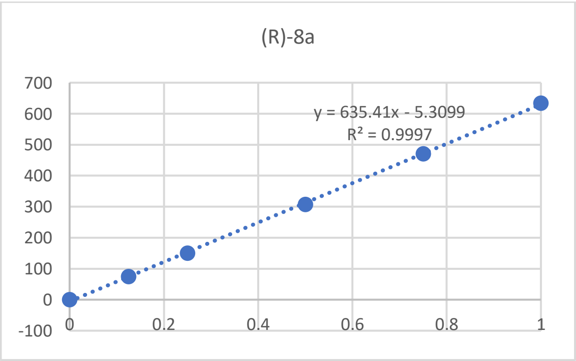

*3-Fluoro-4-(pyridin-3-yl)butan-2-one* (**8a**)

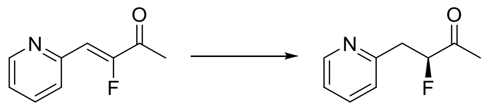


(*Z*)-**2a**

(*S*)-**8a**
94% yield, 91:9 er
(20% pQR1445)

| Compound | $t_R$ |
|---|---|
| (*Z*)-**2a** | 13.7 min |
| (*R*)-**8a** | 11.6 min |
| (*S*)-**8a** | 12.2 min |

**Conditions:** HPLC, 1% IPA/hexane, 1 mL/min$^{-1}$, 214 nm, OJ-H column.

Starting Material:

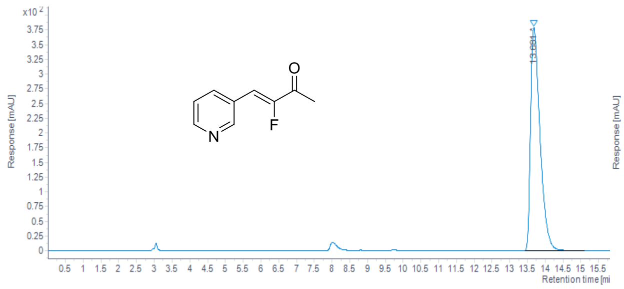


Racemic Standard:

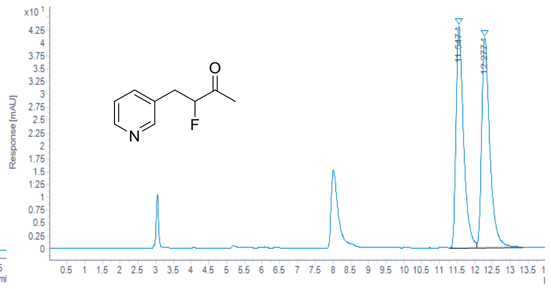


Reaction Trace:

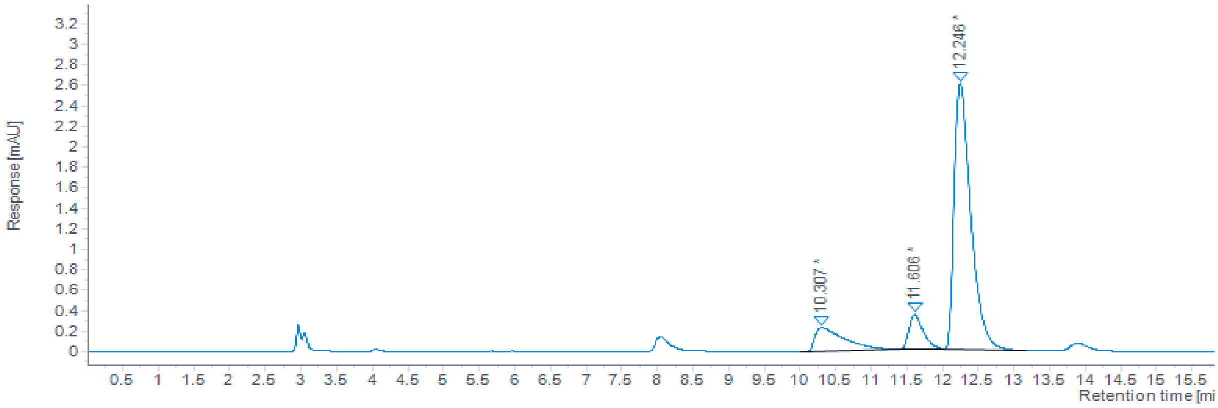


Calibration Curves:

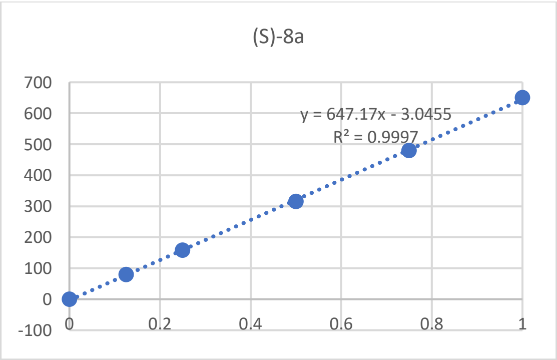


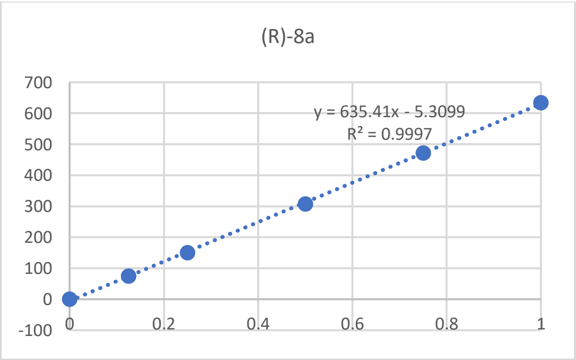

## 3-Fluoro-4-(thiophen-2-yl)butan-2-one (8b)

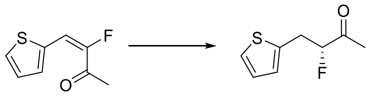

(*E*)-**2b** (*R*)-**8b**
>99% yield, 98:2 er (60% v/v NCR)
42% yield, 85:15 er (20% v/v pQ1445

| Compound | $t_R$ |
|---|---|
| (*E*)-**2b** | 20.2 min |
| (*R*)-**8b** | 16.3 min |
| (*S*)-**8b** | 16.0 min |

**Conditions:** GC, Supelco Beta Dex 225 capillary GC column (30 m × 250 µm, 0.25 µm), method: 50 °C (hold 3 min), 50 °C to 170 °C (5 °C/min), 170 °C (hold 1 min).

Starting Material:

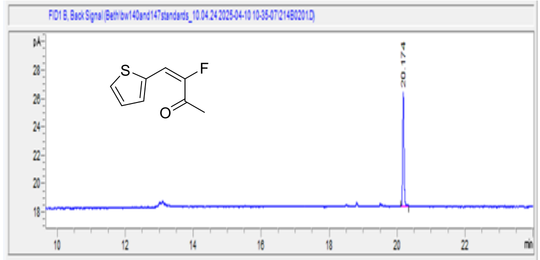


85:15 using pQR1445:

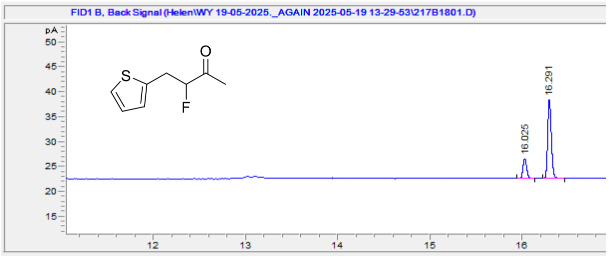


Reaction Trace:

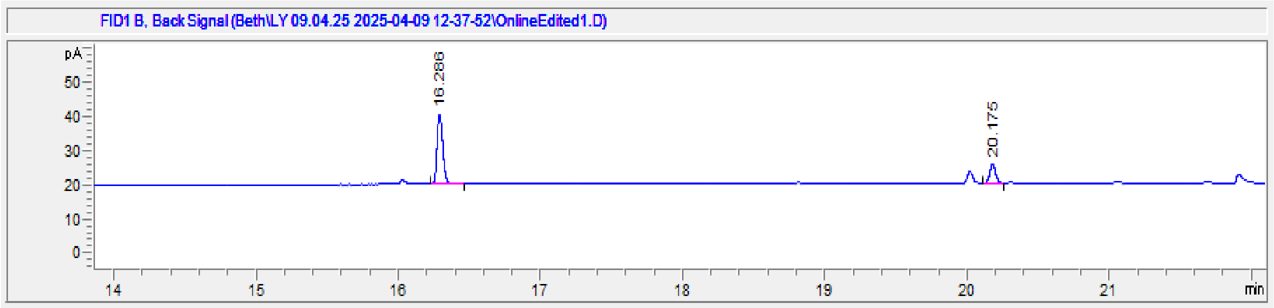


Calibration Curves: (using the large-scale reaction products)

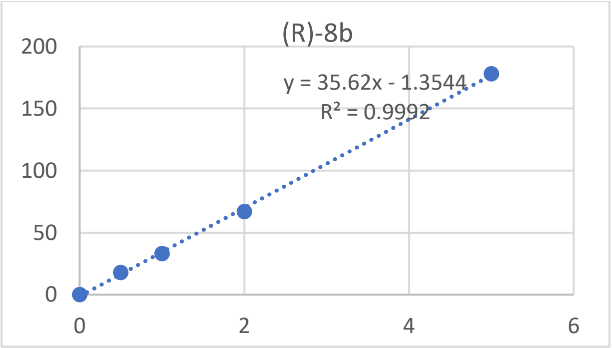

# 4-Cyclohexyl-3-fluorobutan-2-one (9a)

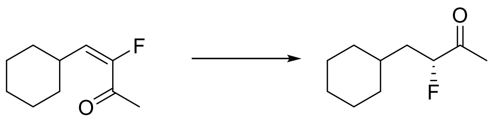

(*E*)-**3a** (*R*)-**9a**
>99% yield, 98:2 er

| Compound | $t_R$ |
|---|---|
| (*E*)-**3a** | 8.0 min |
| (*R*)-**9a** | 10.0 min |
| (*S*)-**9a** | 10.3 min |

**Conditions:** GC, Supelco Beta Dex 225 capillary GC column (30 m × 250 µm, 0.25 µm), method: 60 °C (hold 0 min), 60 °C to 100 °C (5 °C/min), 100 °C (hold 5 min), 100 °C to 220 °C (20 °C/min) and 220 °C (hold 5 min).

Starting Material:

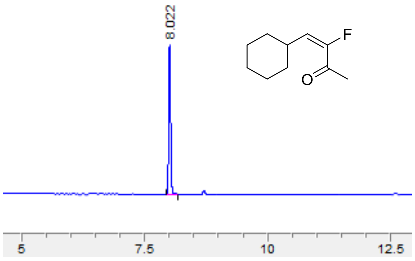


Racemic Standard:

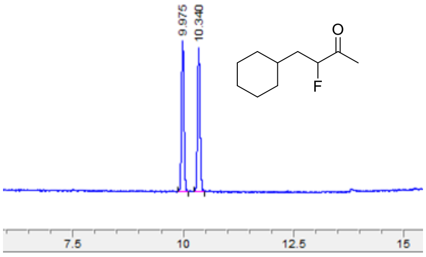


Reaction Trace:

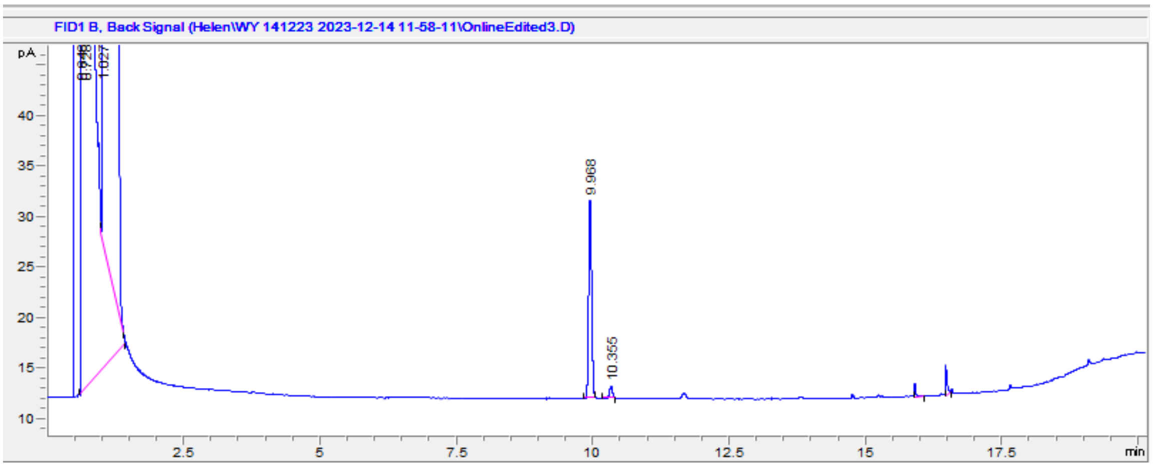


Calibration Curves:

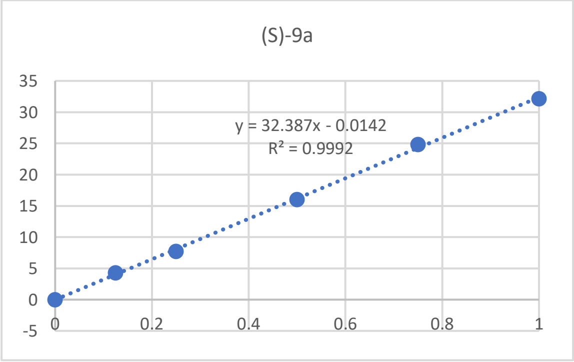


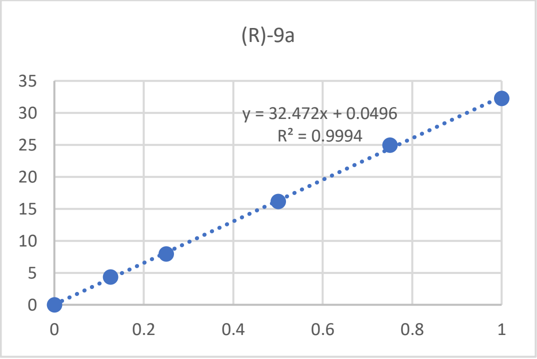

*4-Cyclohexyl-3-fluorobutan-2-one* (**9a**)

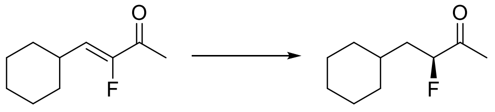


(*Z*)-**3a** (*S*)-**9a**

>99% yield, 99:1 er

| Compound | $t_R$ |
|---|---|
| (*Z*)-**3a** | 12. 5 min |
| (*R*)-**9a** | 10.0 min |
| (*S*)-**9a** | 10.3 min |

**Conditions:** GC, Supelco Beta Dex 225 capillary GC column (30 m × 250 μm, 0.25 μm), method: 60 °C (hold 0 min), 60 °C to 100 °C (5 °C/min), 100 °C (hold 5 min), 100 °C to 220 °C (20 °C/min) and 220 °C (hold 5 min).

Starting Material:

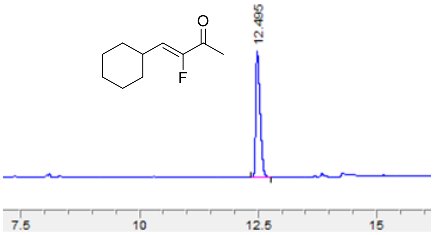


Racemic Standard:

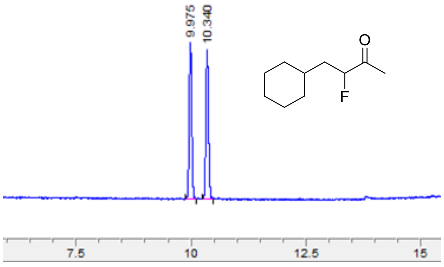


Reaction Trace:

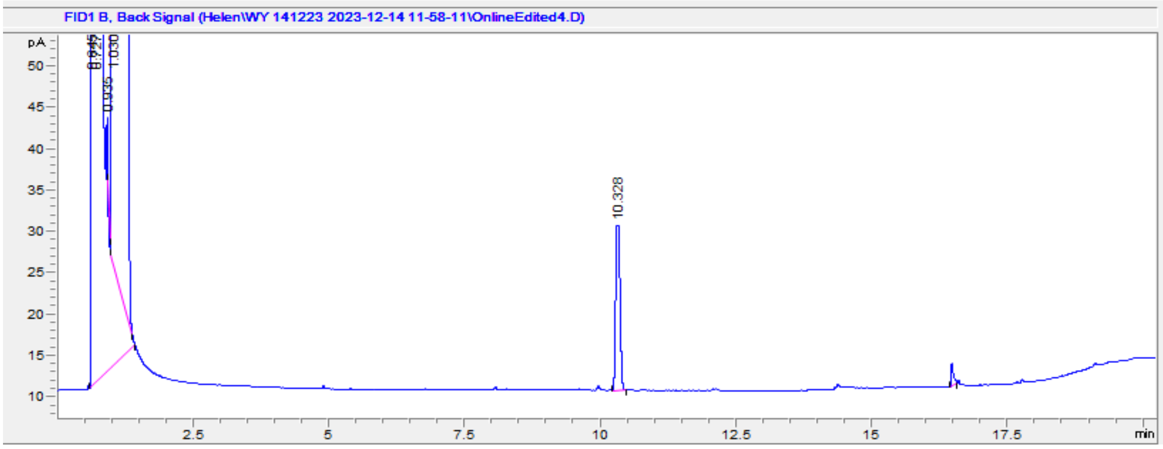


Calibration Curves:

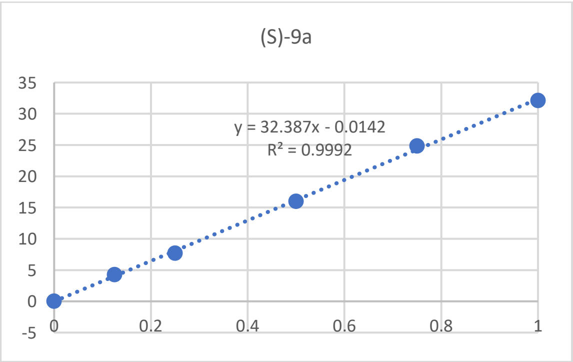


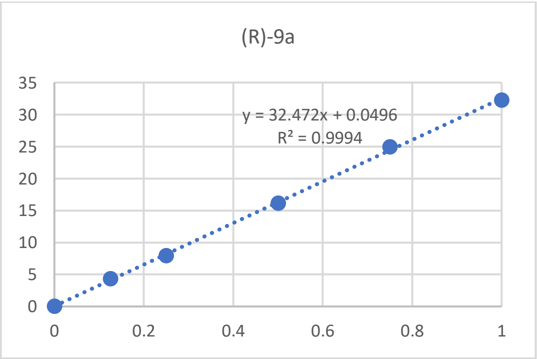

## 3-Fluoro-4-phenylbutan-2-one (9b)

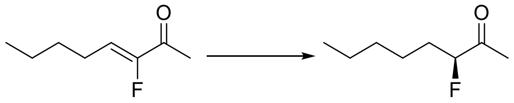


(*Z*)-**3b** (*S*)-**9b**
No SM remained, 99:1 er

| Compound | $t_R$ |
|---|---|
| (Z)-**3b** | 6.8 min |
| (*R*)-**9b** | 5.3 min |
| (*S*)-**9b** | 5.9 min |

**Conditions:** GC, Supelco Beta Dex 225 capillary GC column (30 m × 250 µm, 0.25 µm), method: 60 °C (hold 0 min), 60 °C to 100 °C (5 °C/min), 100 °C (hold 5 min), 100 °C to 220 °C (20 °C/min) and 220 °C (hold 5 min).

Starting Material:

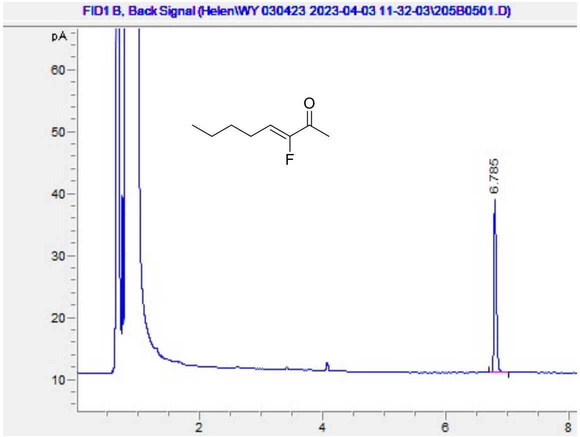


Racemic Standard:

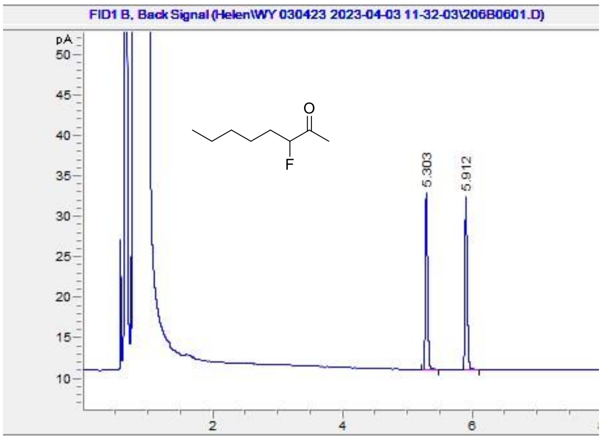


Reaction Trace:

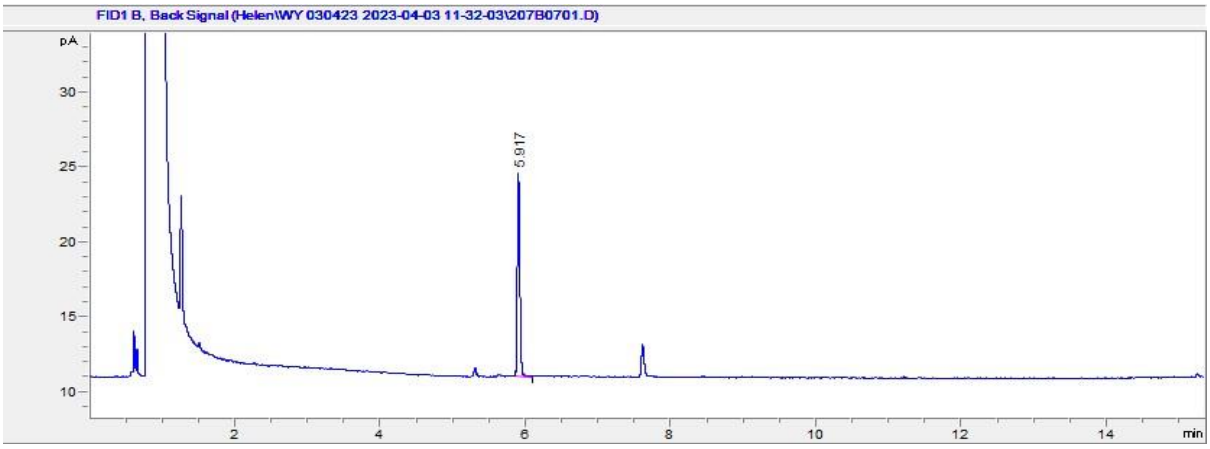

# 3-Fluoro-6-phenylpentan-2-one (9c)

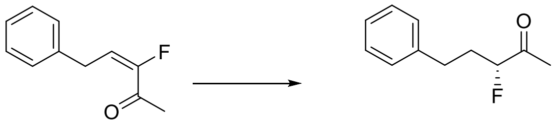


(*E*)-**3c**

(*R*)-**9c**
>99% yield, 86:14 er
60% v/v NCR

| Compound | $t_R$ |
|---|---|
| (*E*)-**3c** | 17.2 min |
| (*R*)-**9c** | 19.0 min |
| (*S*)-**9c** | 18.9 min |

**Conditions:** GC, Supelco Beta Dex 225 capillary GC column (30 m × 250 µm, 0.25 µm), method: 50 °C (hold 3 min), 50 °C to 170 °C (5 °C/min), 170 °C (hold 1 min).

Starting Material:

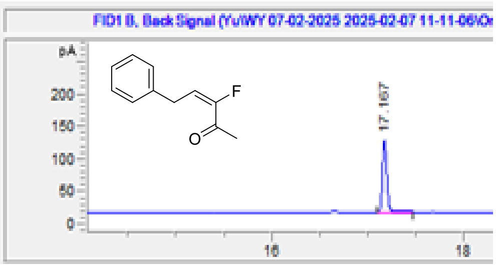


Racemic Standard:

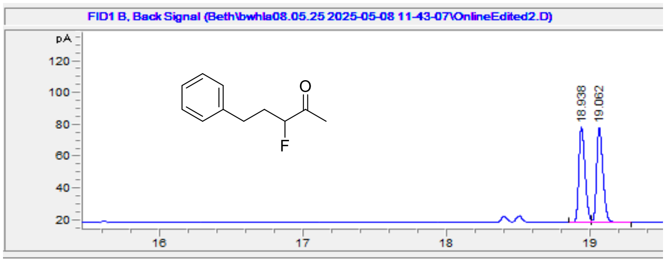


Reaction Trace:

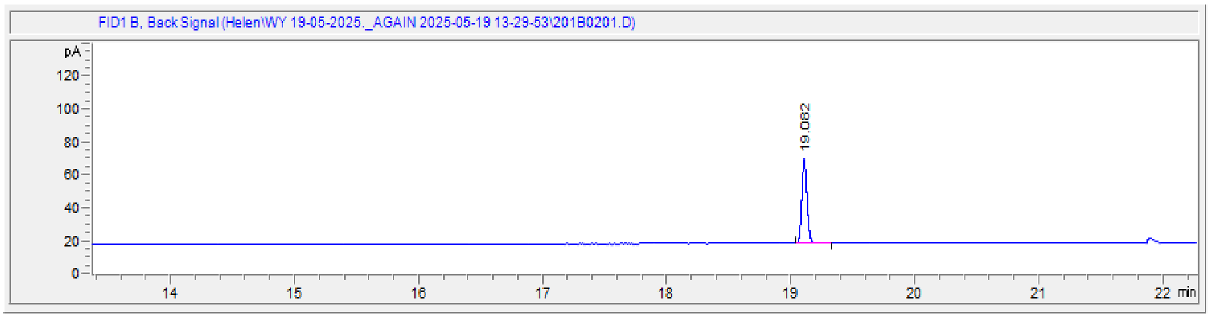


Calibration Curves:

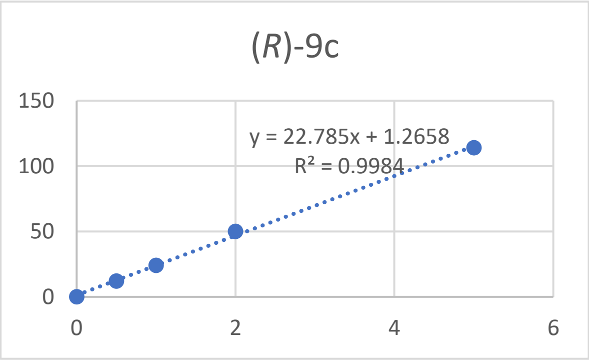


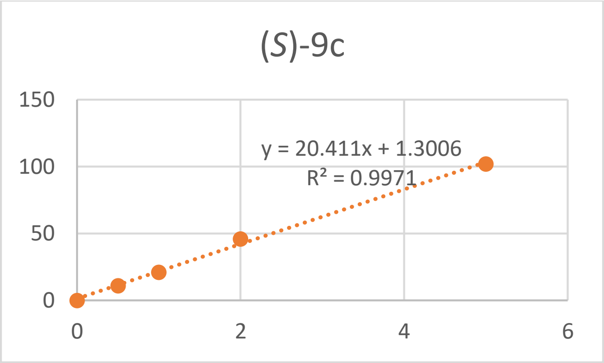

## 3-Fluoro-6-phenylhexan-2-one (9d)

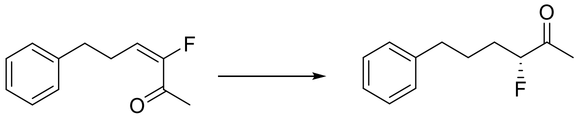


(*E*)-**3d** → (*R*)-**9d**
82% yield, 78:22 er
60% v/v NCR

| Compound | $t_R$ |
| --- | --- |
| (*E*)-**3d** | 19.6 min |
| (*R*)-**9d** | 21.6 min |
| (*S*)-**9d** | 21.3 min |

**Conditions:** GC, Supelco Beta Dex 225 capillary GC column (30 m × 250 µm, 0.25 µm), method: 50 °C (hold 3 min), 50 °C to 170 °C (5 °C/min), 170 °C (hold 1 min).

Starting Material:

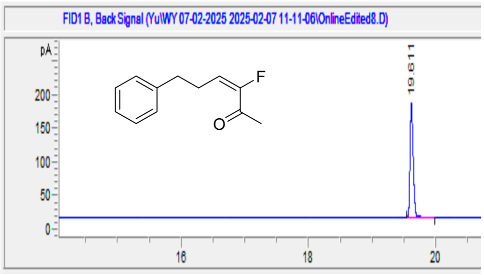


Racemic Standard:

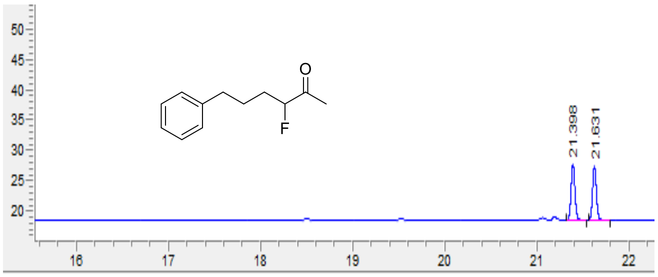


Reaction Trace:

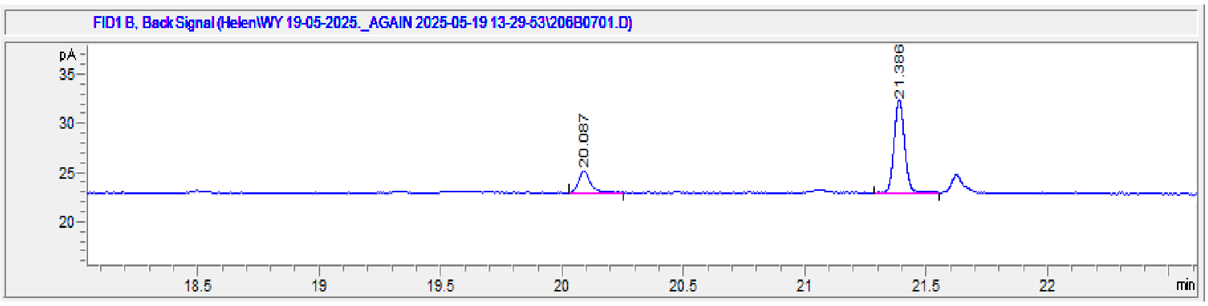


Calibration Curves:

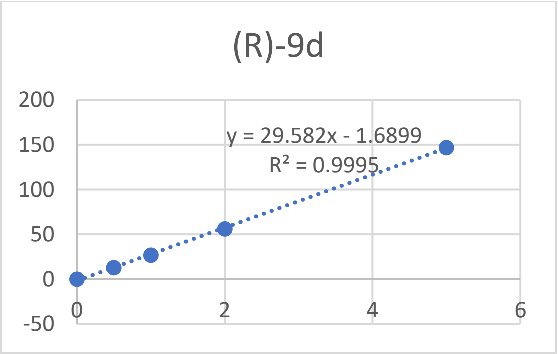


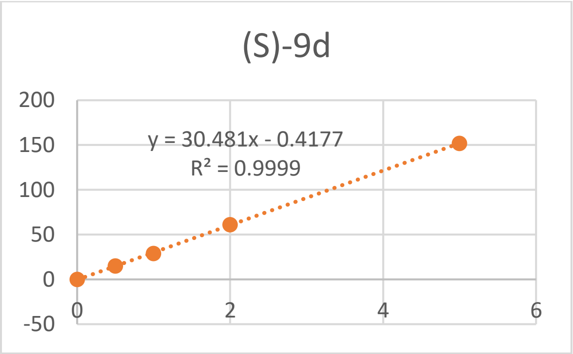

## 2-Fluorocyclohexan-1-one (10a)

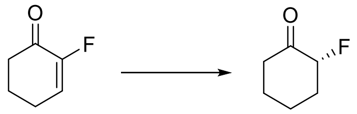

(*E*)-**4a** → (*R*)-**10a**

>99% yield, 99:1 er

| Compound | $t_R$ |
|---|---|
| (*E*)-**4a** | 20.7 min |
| (*R*)-**10a** | 18.0 min |
| (*S*)-**10a** | 18.2 min |

**Conditions:** GC, Supelco Beta Dex 225 capillary GC column (30 m × 250 µm, 0.25 µm), method: 50 °C (hold 3 min), 50 °C to 170 °C (5 °C/min), 170 °C (hold 1 min).

Starting Material:

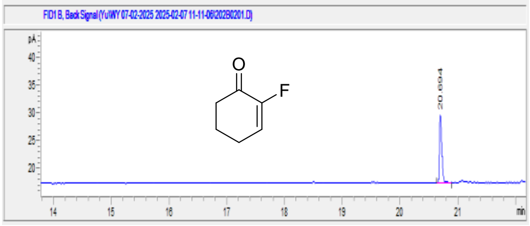

Racemic Standard:

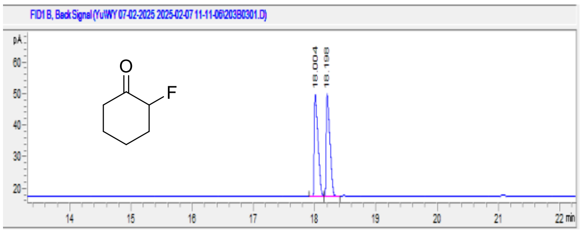

Reaction Trace:

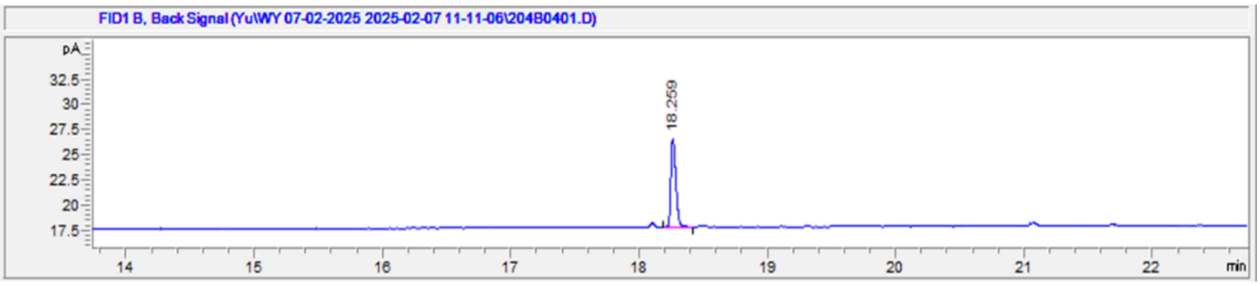


Calibration Curves:

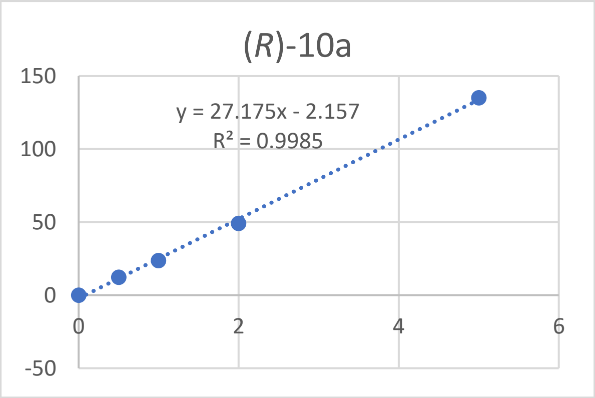


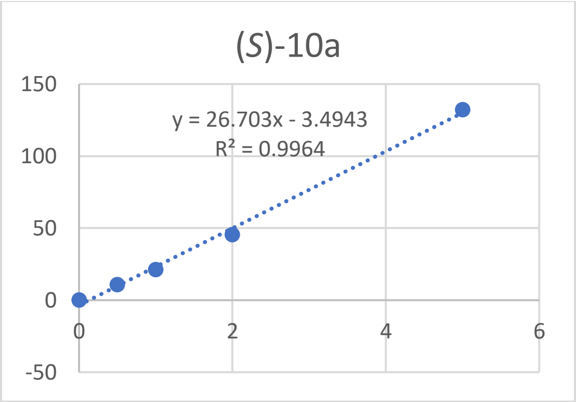

## 2-Fluorocyclohept-1-one (10b)

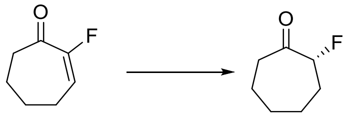

(*E*)-**4b** (*R*)-**10b**
>99% yield, 99:1 er
60% v/v NCR

| Compound | $t_R$ |
|---|---|
| (*E*)-**4b** | 21.4 min |
| (*R*)-**10b** | 16.9 min |
| (*S*)-**10b** | 15.4 min |

**Conditions:** GC, Supelco Beta Dex 225 capillary GC column (30 m × 250 µm, 0.25 µm), method: 50 °C (hold 3 min), 50 °C to 170 °C (5 °C/min), 170 °C (hold 1 min).

Starting Material:

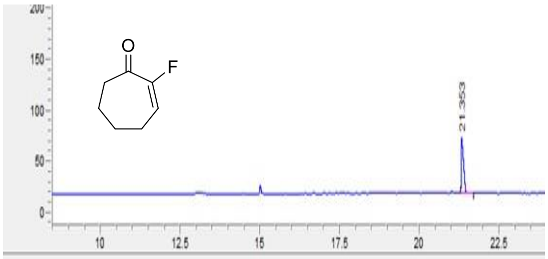

Racemic Standard:

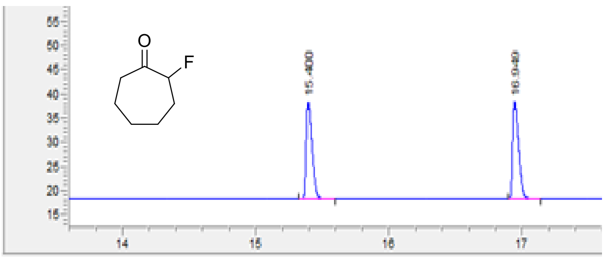

Reaction Trace:

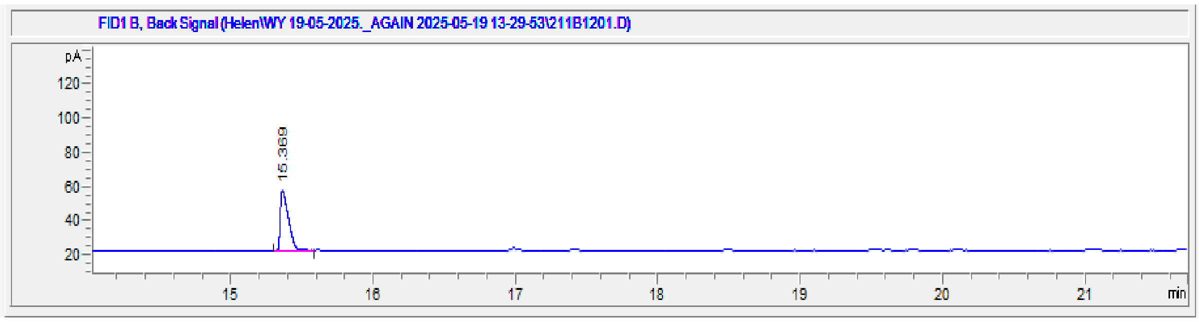


Calibration Curves:

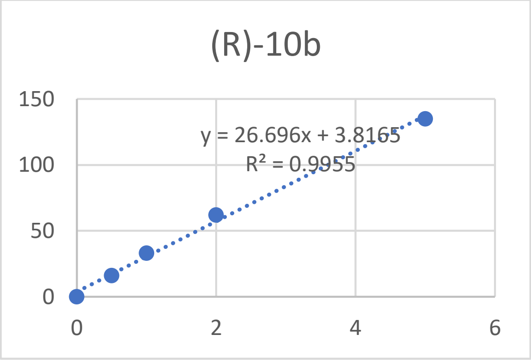


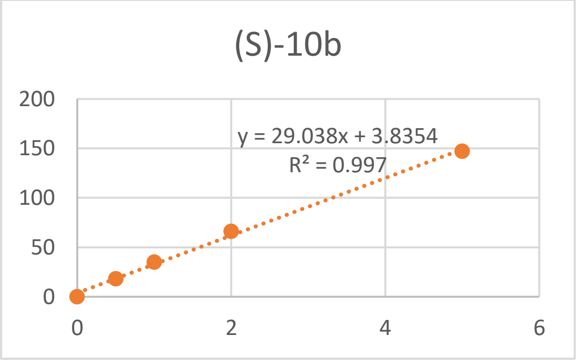

## 2-Fluoro-1-phenylpentan-3-one (11a)

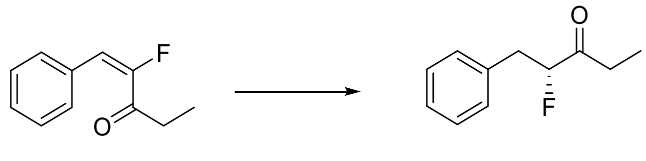


(*E*)-**5a**

(*R*)-**11a**
98% yield, 98:2 er

| Compound | $t_R$ |
|---|---|
| (*E*)-**5a** | 20.9 min |
| (*R*)-**11a** | 13.7 min |
| (*S*)-**11a** | 16.4 min |

**Conditions:** HPLC, 1% IPA/hexane, 1 mL/min$^{-1}$, 214 nm, OJ-H column. (The differences of retention time from 'Determination of Absolute Stereochemistry' (12.6/14.5 min) are due to the usage of a different HPLC device)

Starting Material:

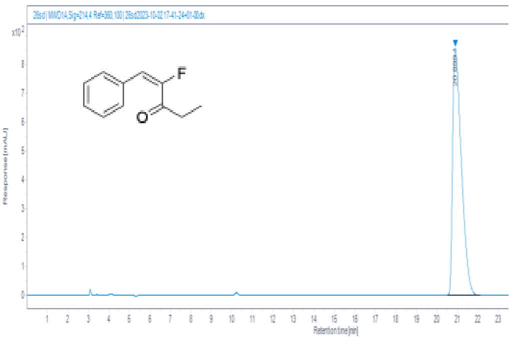


Racemic Standard:

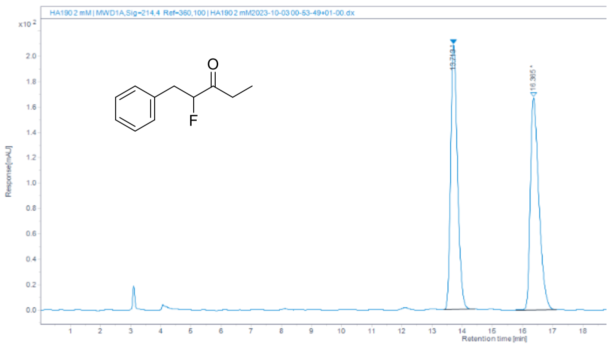


Reaction Trace:

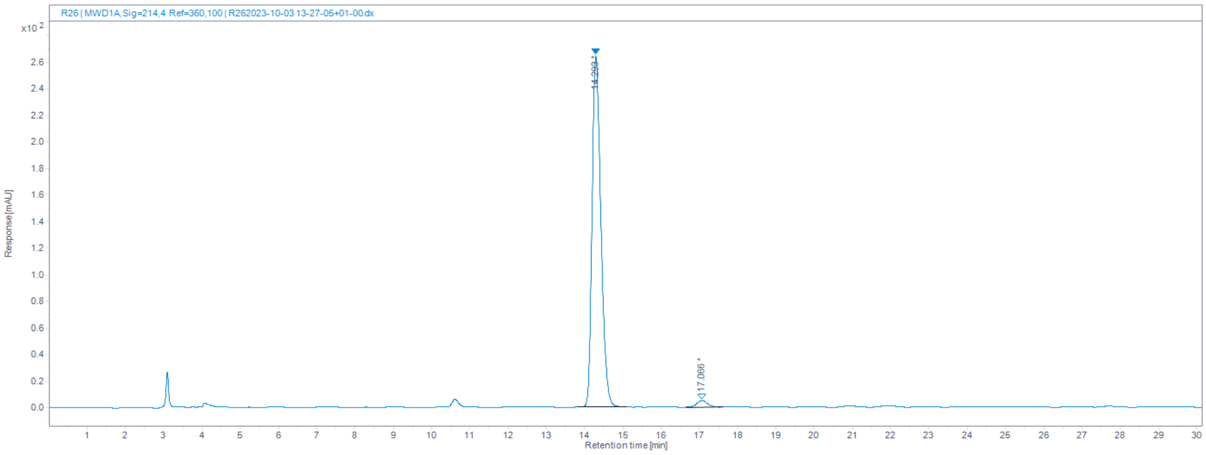


Calibration Curves:

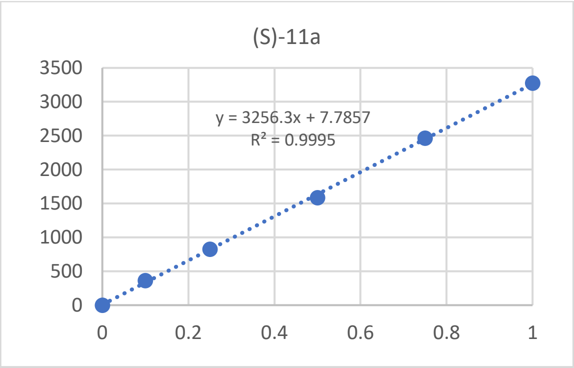


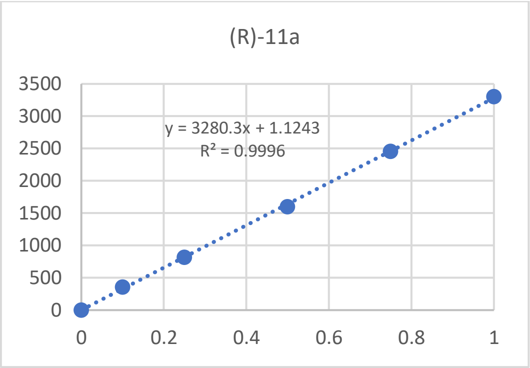

*2-Fluoro-1-phenylpentan-3-one* (**11a**)

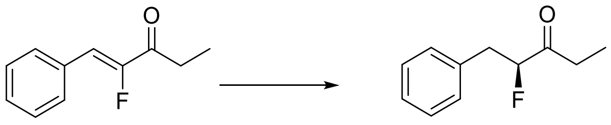


(*Z*)-**5a**

(*S*)-**11a**
<5% conversion

| Compound | $t_R$ |
|---|---|
| (*Z*)-**5a** | 10.2 min |
| (*R*)-**11a** | 13.7 min |
| (*S*)-**11a** | 16.4 min |

**Conditions:** HPLC, 1% IPA/hexane, 1 mL/min$^{-1}$, 214 nm, OJ-H column. (The differences of retention time from 'Determination of Absolute Stereochemistry' (12.6/14.5 min) are due to the usage of a different HPLC device).

Starting Material:

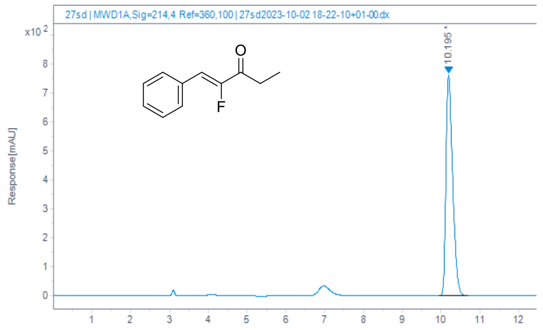

Racemic Standard:

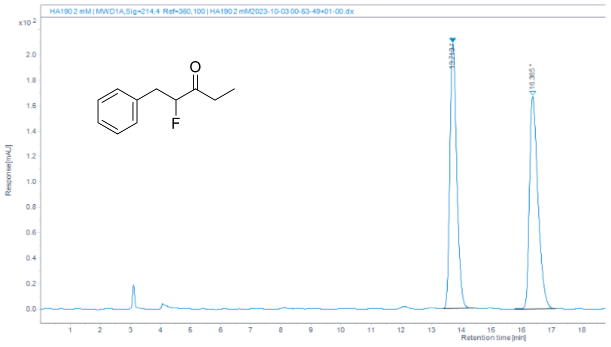

Reaction Trace:

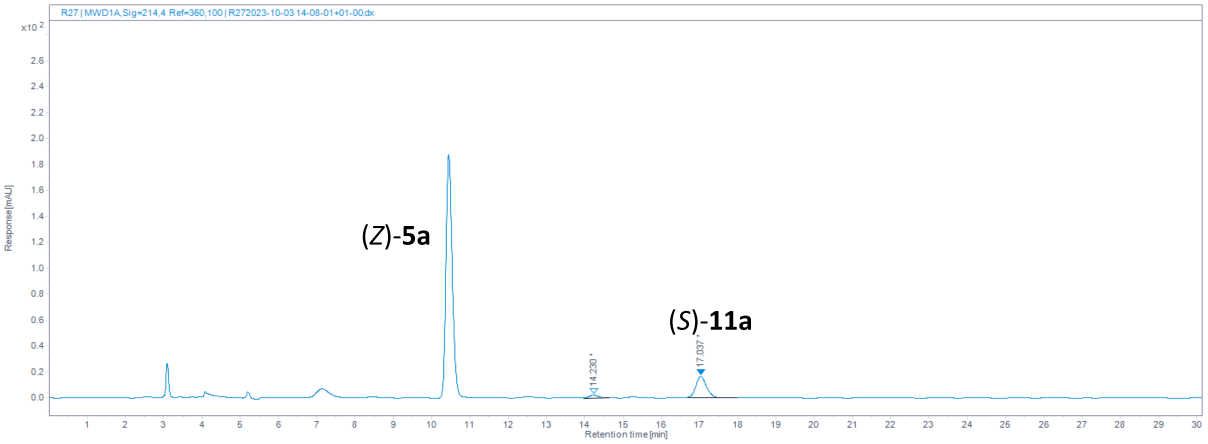


Calibration Curves:

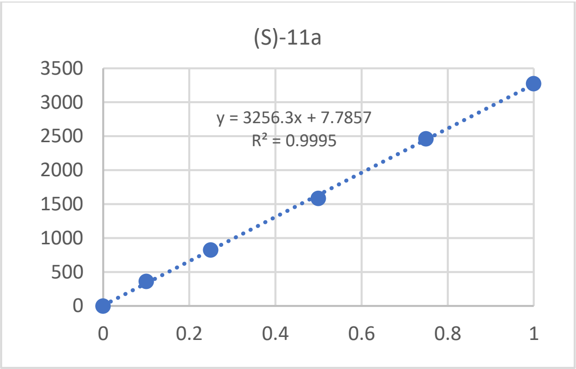


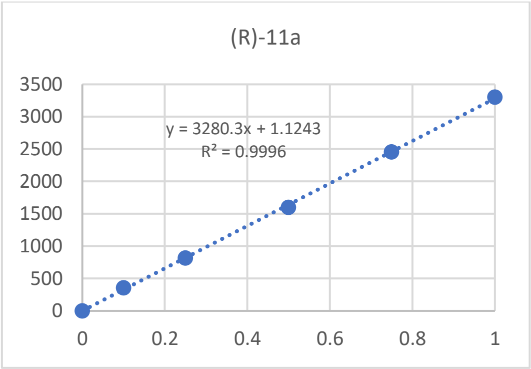

## 2-Fluoro-1-phenylheptan-1-one (11b)

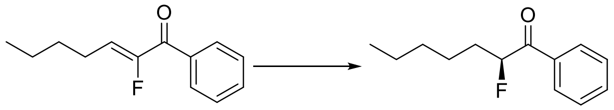

(*Z*)-**5b**

(*S*)-**11b**
>99% yield, 98:2 er

| Compound | $t_R$ |
|---|---|
| (*Z*)-**5b** | 4.8 min |
| (*R*)-**11a** | 4.9 min |
| (*S*)-**11a** | 5.2min |

**Conditions:** HPLC, 1% IPA/hexane, 1 mL/min$^{-1}$, 214 nm, OD-H column.

Starting Material:

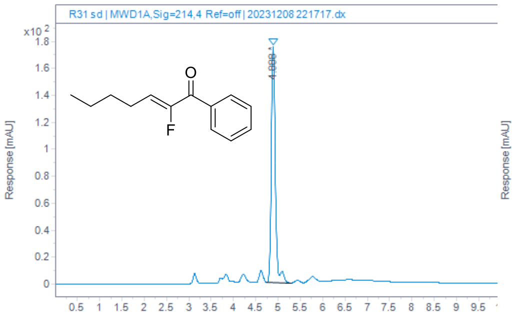


Racemic Standard:

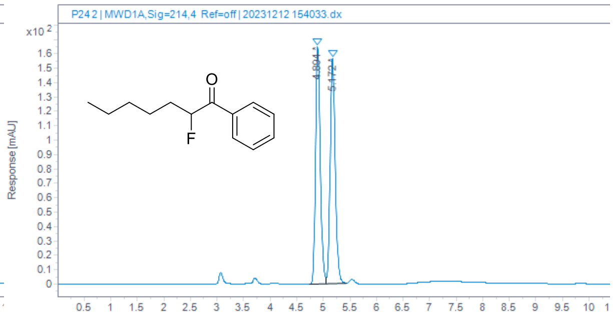


Reaction Trace:

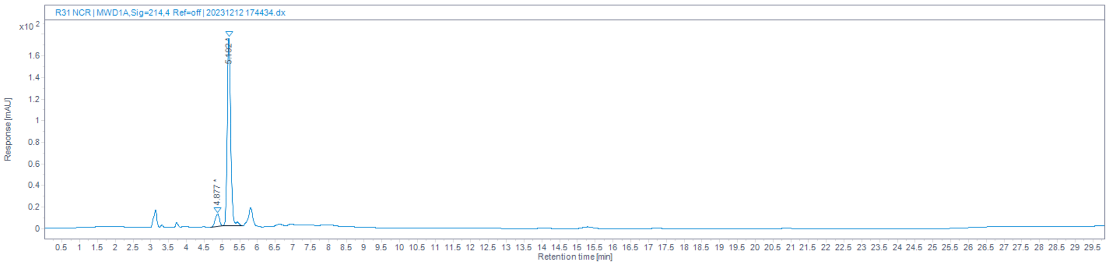


Calibration Curves:

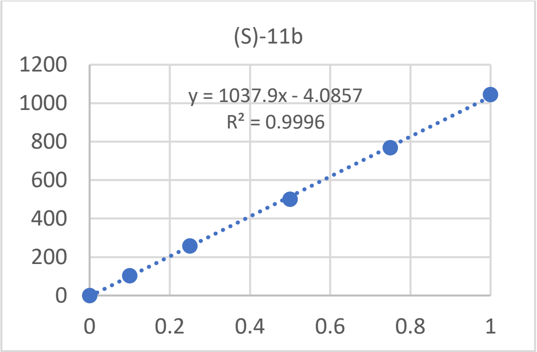


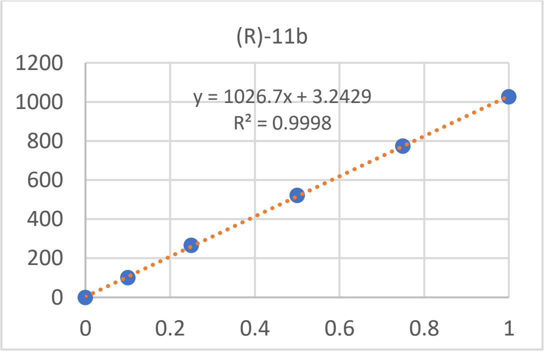

## Methyl 2-fluoro-3-phenylpropanoate (12a)

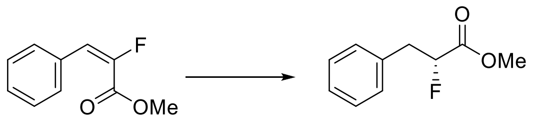

(*E*)-**6a** (8:1 *E*:*Z*) (*R*)-**12a** 55% yield, 99:1 er

| Compound | $t_R$ |
|---|---|
| (*E*)-**6a** | 4.2 min |
| (*Z*)-**6a** | 15.4 min |
| (*R*)-**12a** | 7.2 min |
| (*S*)-**12a** | 8.1 min |

**Conditions:** HPLC, 1% IPA/hexane, 1 mL/min$^{-1}$, 214 nm, OD-H column. (The differences of retention time from 'Determination of Absolute Stereochemistry' (10.8/11.9 min) are due to the usage of a different HPLC device)

Starting Material:

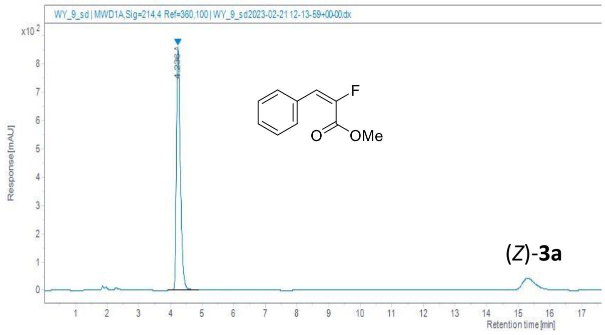


Racemic Standard:

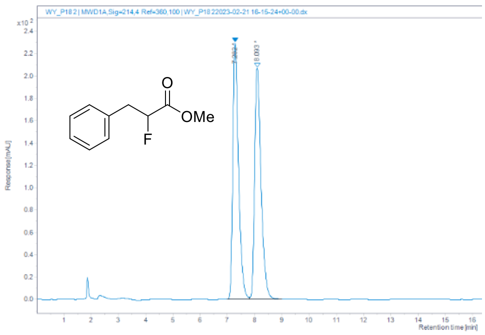


Reaction Trace:

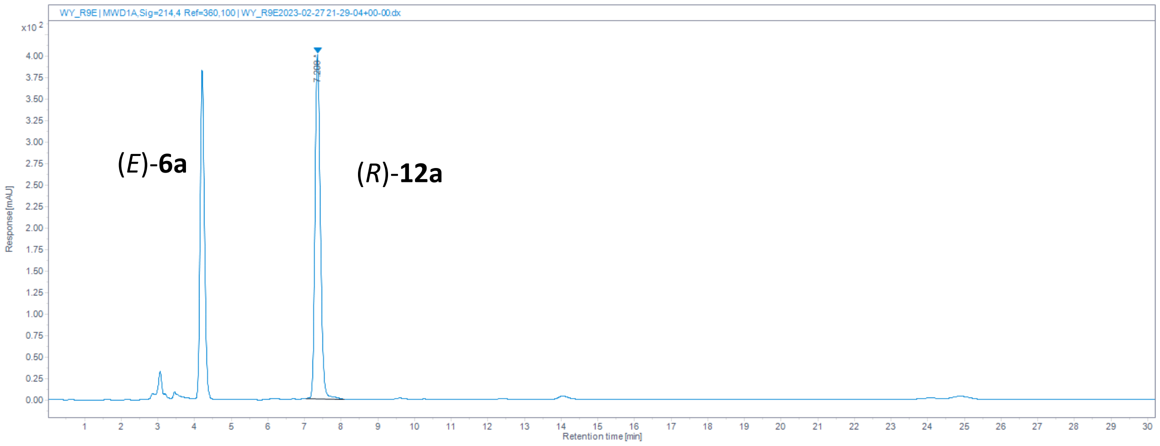


Calibration Curves:

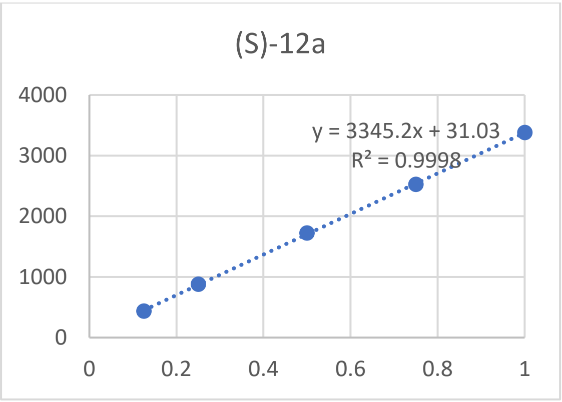


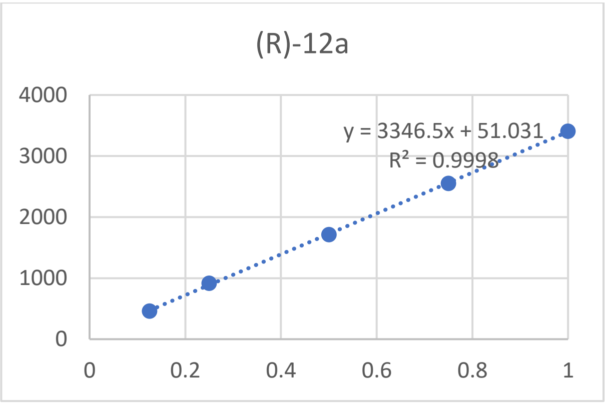

*Methyl 2-fluoro-3-phenylpropanoate* (**12a**)

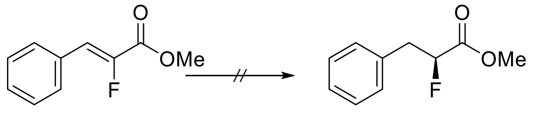

(*Z*)-**6a**
Not accepted

(*S*)-**12a**

| Compound | $t_R$ |
|---|---|
| (*Z*)-**6a** | 15.4 min |
| (*R*)-**12a** | 7.2 min |
| (*S*)-**12a** | 8.1 min |

**Conditions:** HPLC, 1% IPA/hexane, 1 mL/min$^{-1}$, 214 nm, OD-H column. (The differences of retention time from 'Determination of Absolute Stereochemistry' (10.8/11.9 min) are due to the usage of a different HPLC device)

Starting Material:

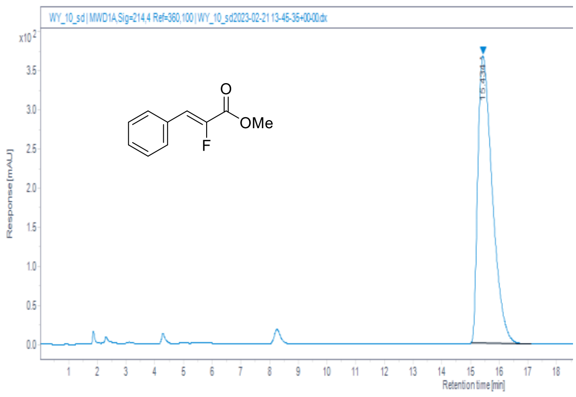

Racemic Standard:

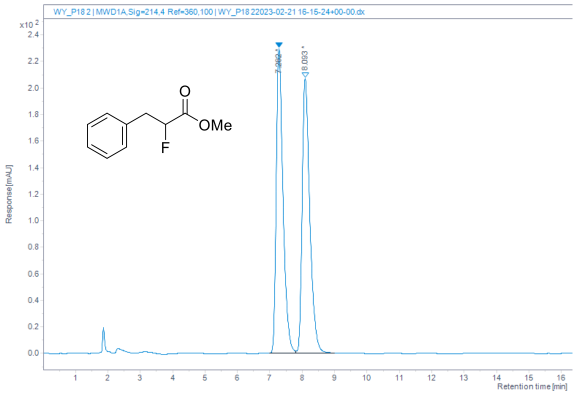

Reaction Trace:

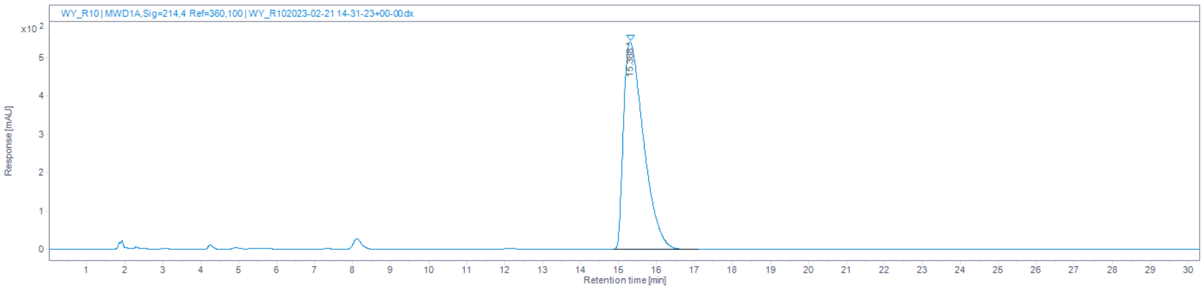

Calibration Curves:

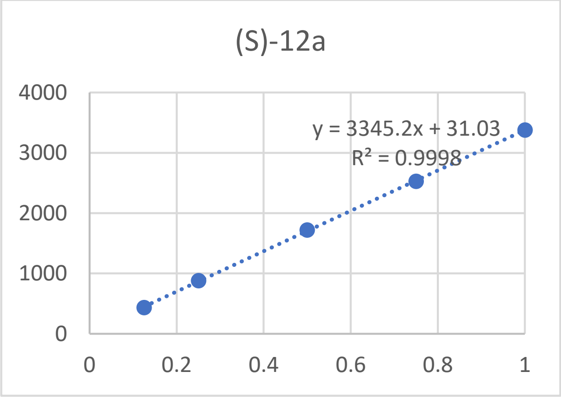


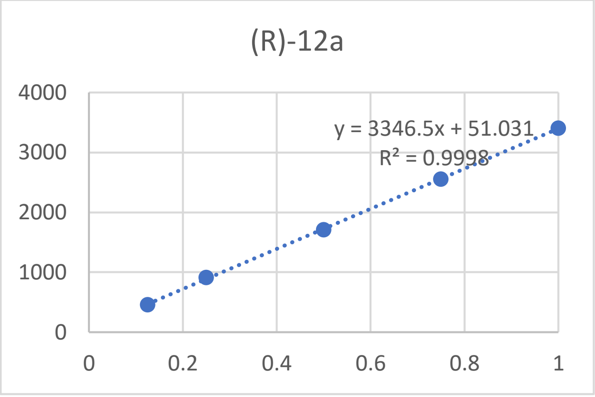

# Methyl 2-fluoro-3-(4-trifluoromethylphenyl)propanoate (12b)

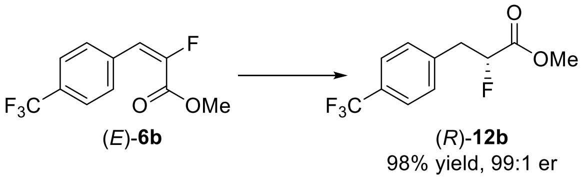


| Compound | $t_R$ |
|---|---|
| (*E*)-**6b** | 5.4 min |
| (*R*)-**12b** | 6.7 min |
| (*S*)-**12b** | 7.2 min |

**Conditions:** HPLC, 1% IPA/hexane, 1 mL/min$^{-1}$, 214 nm, AD-H column.

Starting Material:

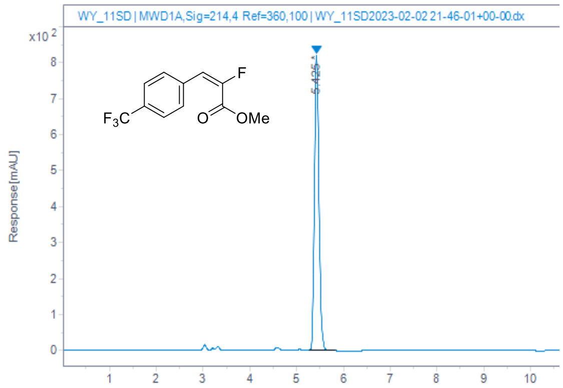


Racemic Standard:

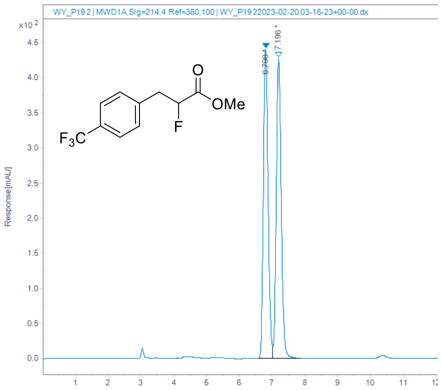


Reaction Trace:

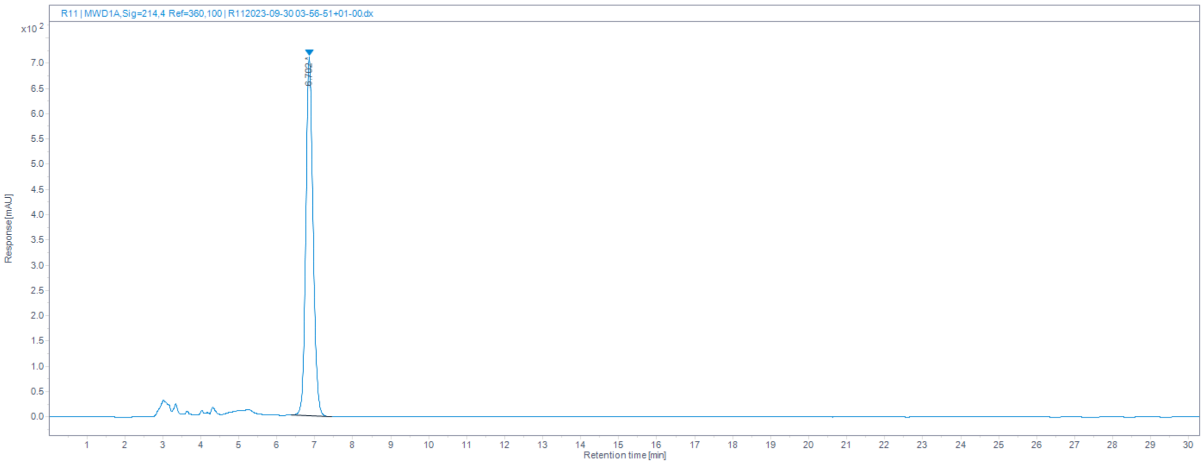


Calibration Curves:

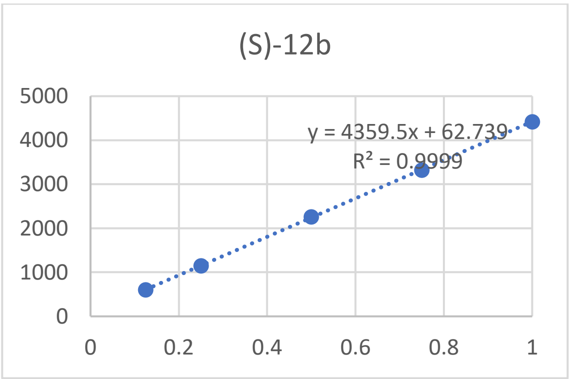


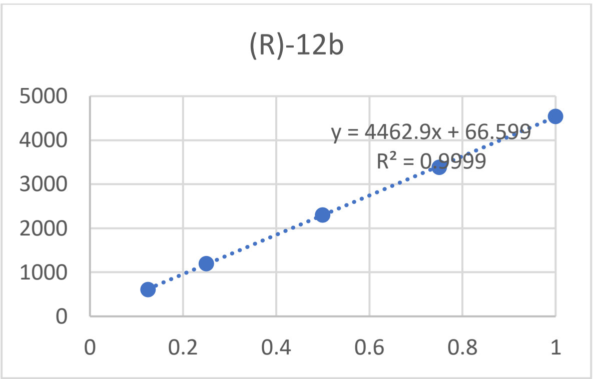

*Methyl 2-fluoro-3-(4-trifluoromethylphenyl)propanoate* (**12b**)

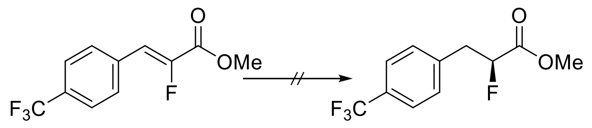


(*Z*)-**6b**
Not accepted

(*S*)-**12b**

| Compound | $t_R$ |
|---|---|
| (*Z*)-**6b** | 6.9 min |
| (*R*)-**12b** | 6.7 min |
| (*S*)-**12b** | 7.2 min |

**Conditions:** HPLC, 1% IPA/hexane, 1 mL/min$^{-1}$, 214 nm, AD-H column.

Starting Material:

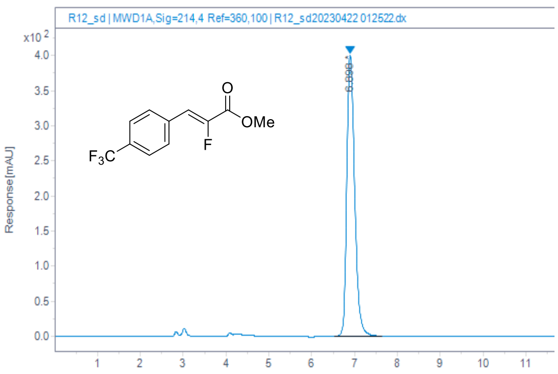


Racemic Standard:

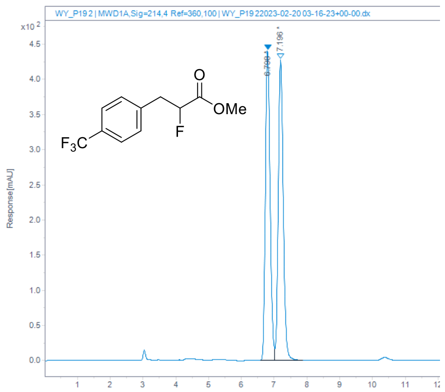


Reaction Trace:

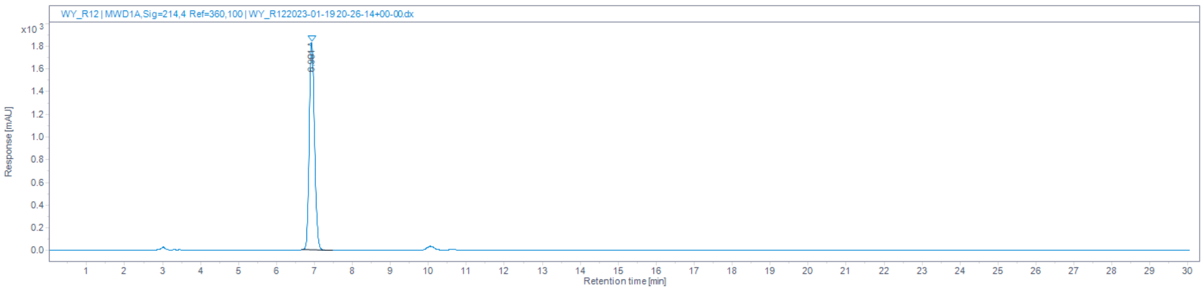


Calibration Curves:

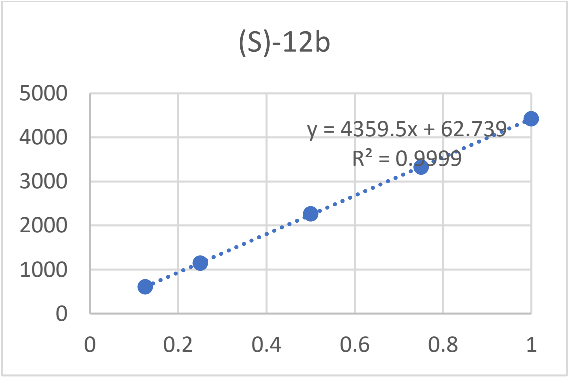


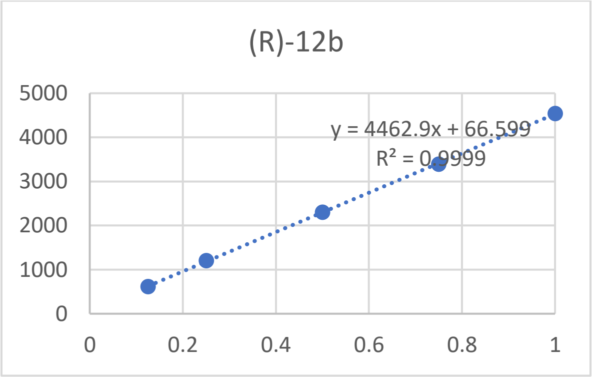

## Methyl 2-fluoro-3-(4-methoxyphenyl)propanoate (12c)

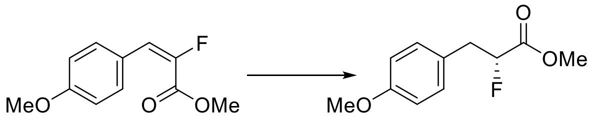

(*E*)-**6c** (5:1 *E*:*Z*) → (*R*)-**12c** 43% yield, 99:1 er

| Compound | $t_R$ |
|---|---|
| (*E*)-**6c** | 8.9 min |
| (*Z*)-**6c** | 12.9 min |
| (*R*)-**12c** | 10.3 min |
| (*S*)-**12c** | 11.0 min |

**Conditions:** HPLC, 1% IPA/hexane, 1 mL/min$^{-1}$, 214 nm, AD-H column.

Starting Material:

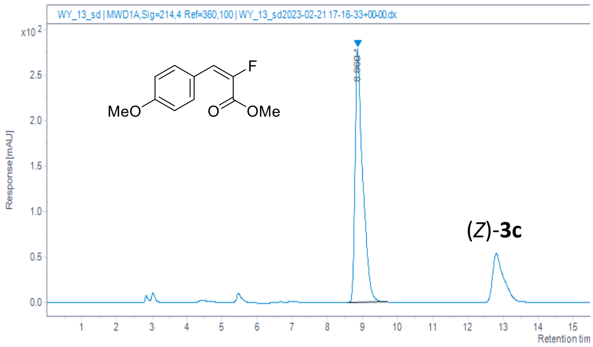


Racemic Standard:

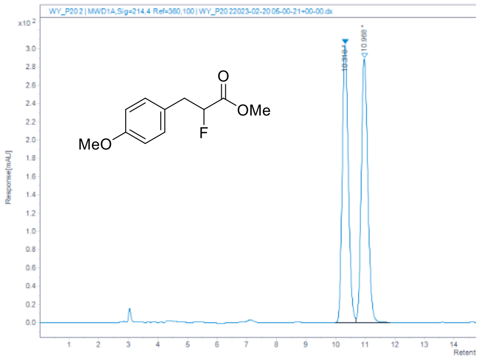

Reaction Trace:

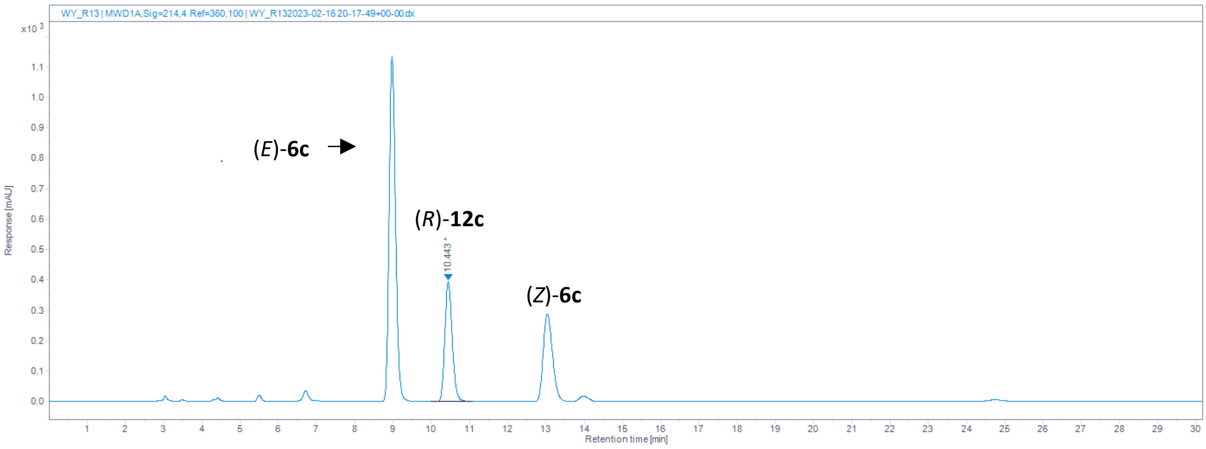


Calibration Curves:

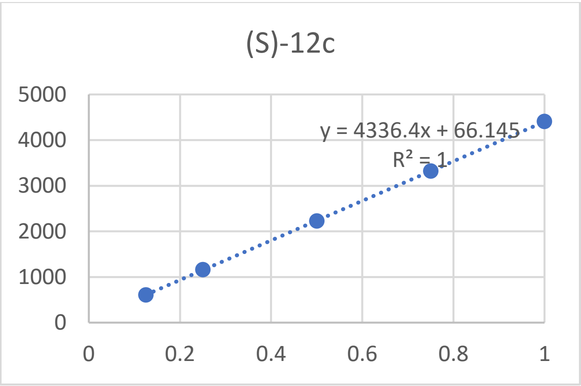


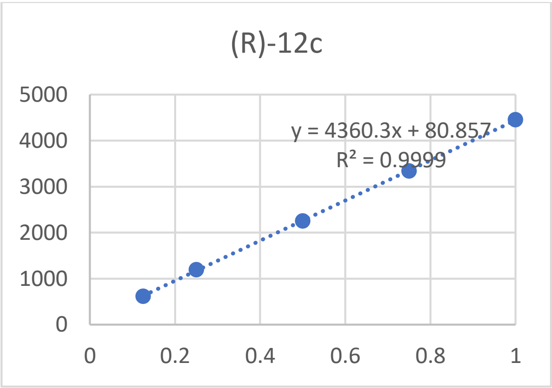

*Methyl 2-fluoro-3-(4-methoxyphenyl)propanoate* (**12c**)

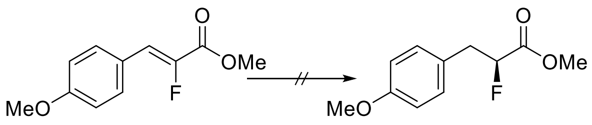


(*Z*)-**6c**
Not accepted

(*S*)-**12c**

| Compound | $t_R$ |
|---|---|
| (*Z*)-**6c** | 12.9 min |
| (*R*)-**12c** | 10.3 min |
| (*S*)-**12c** | 11.0 min |

**Conditions:** HPLC, 1% IPA/hexane, 1 mL/min$^{-1}$, 214 nm, AD-H column.

Starting Material:

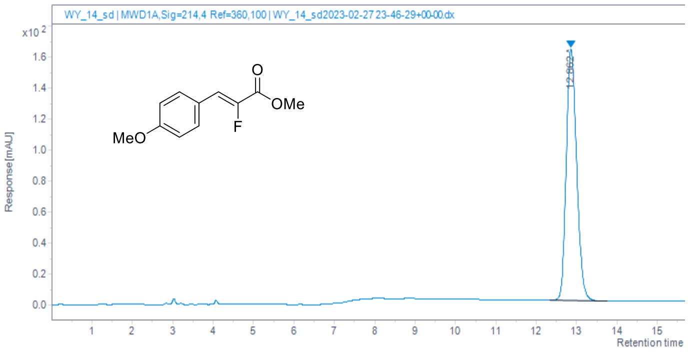

Racemic Standard:

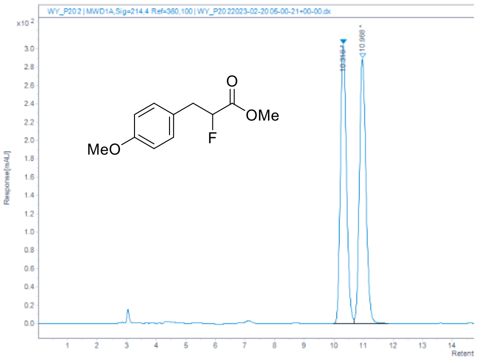

Reaction Trace:

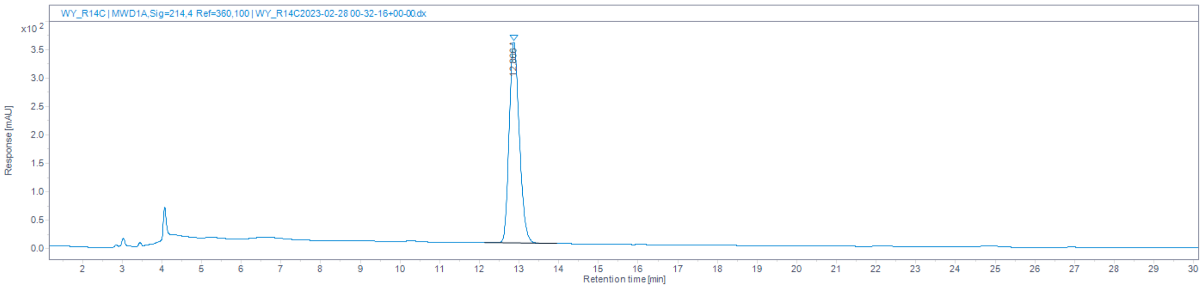

Calibration Curves:

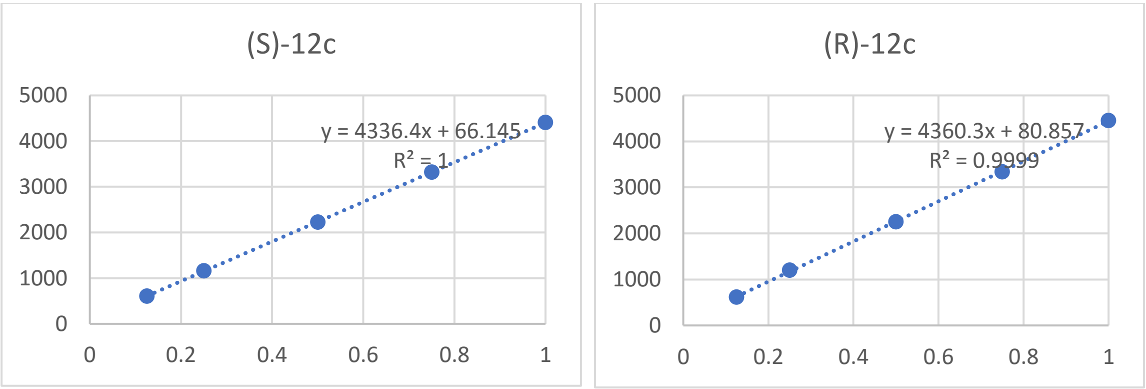

# Ethyl 2-fluoro-3-(4-trifluoromethylphenyl)propanoate (12d)

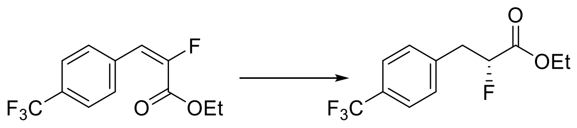


(*E*)-**6d** (*R*)-**12d**
99% yield, 99:1 er

| Compound | $t_R$ |
|---|---|
| (*E*)-**6d** | 5.0 min |
| (*R*)-**12d** | 6.4 min |
| (*S*)-**12d** | 7.0 min |

**Conditions:** HPLC, 1% IPA/hexane, 1 mL/min$^{-1}$, 214 nm, AD-H column.

Starting Material:

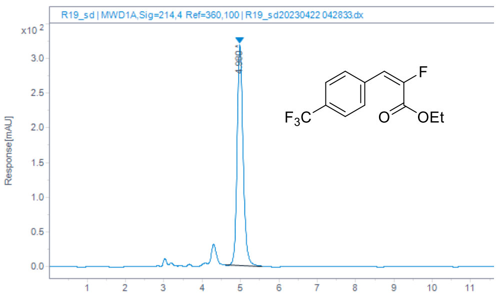

Racemic Standard:

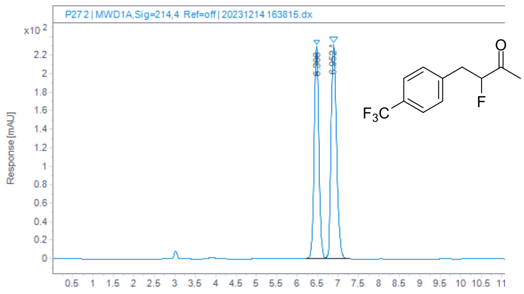

Reaction Trace:

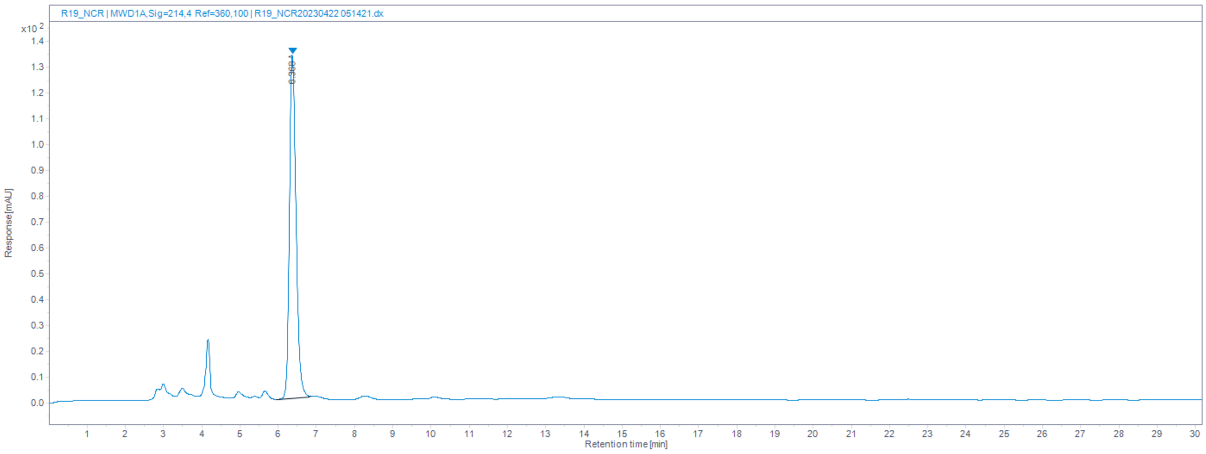

Calibration Curves:

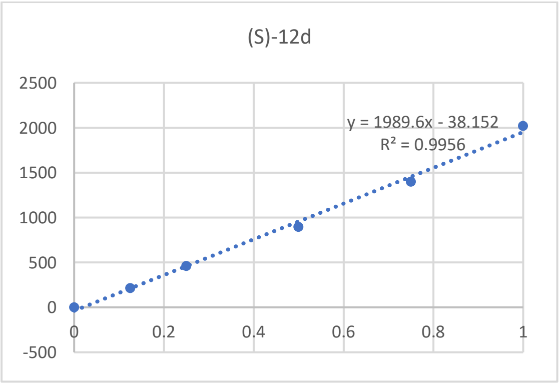


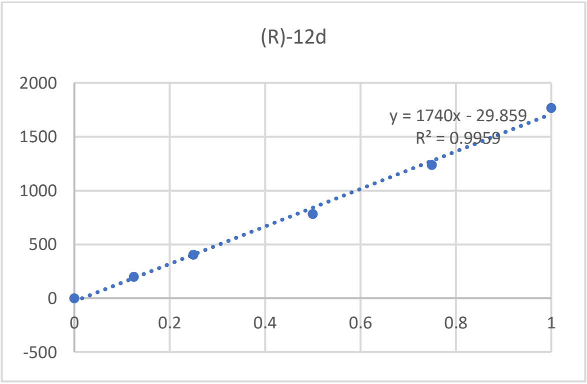

*Ethyl 2-fluoro-3-(4-trifluoromethylphenyl)propanoate* (**12d**)

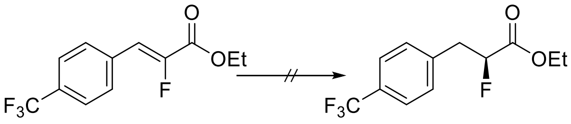


(*Z*)-**6d** Not accepted    (*S*)-**12d**

| Compound | $t_R$ |
|---|---|
| (*Z*)-**6d** | 6.8 min |
| (*R*)-**12d** | 6.4min |
| (*S*)-**12d** | 7.0 min |

**Conditions:** HPLC, 1% IPA/hexane, 1 mL/min$^{-1}$, 214 nm, AD-H column.

Starting Material:

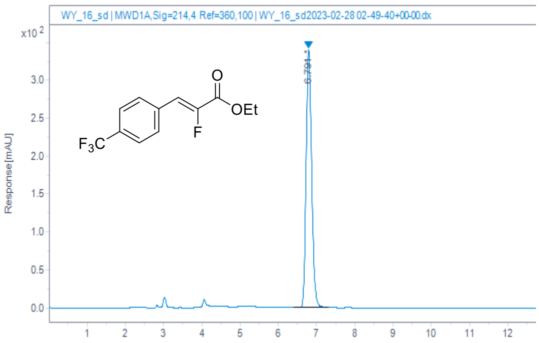


Racemic Standard:

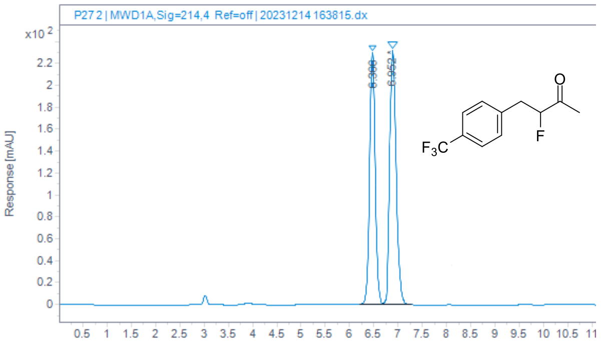


Reaction Trace:

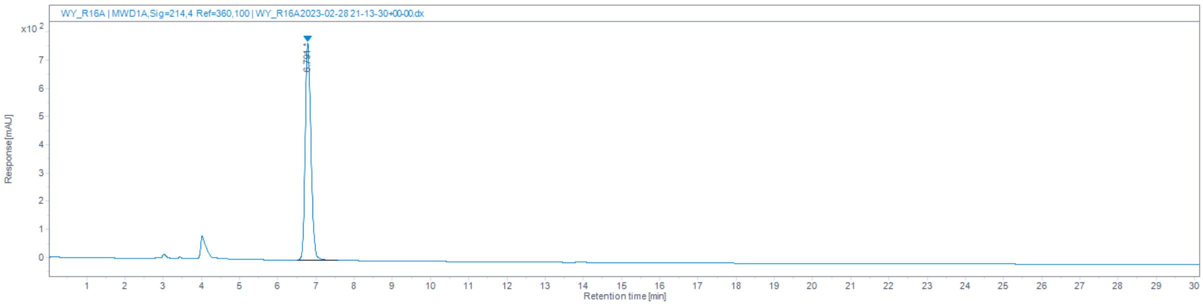


Calibration Curves:

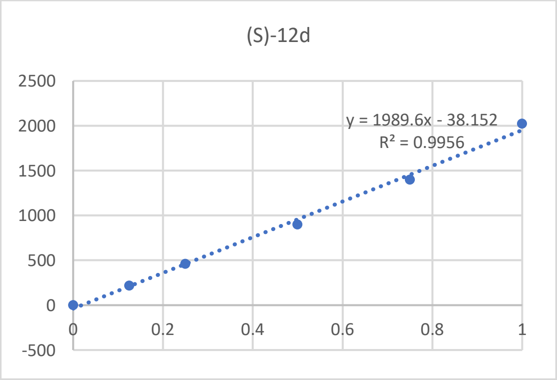


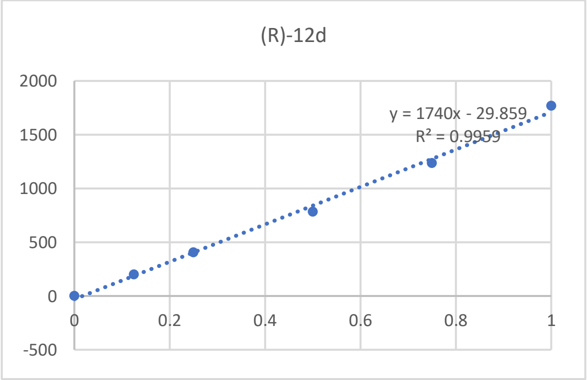

# Methyl 2-fluoroheptanoate (12e)

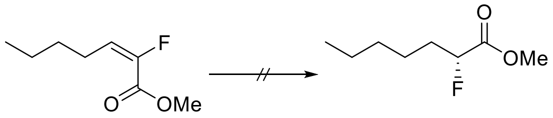


(*E*)-**6e**
(13:1 *E*:*Z*)
Not accepted

(*R*)-**12e**

| Compound | $t_R$ |
| --- | --- |
| (*E*)-**6e** | 6.7 min |
| (*R*)-**12e** | 6.8 min |
| (*S*)-**12e** | 7.4 min |

**Conditions:** GC, Supelco Beta Dex 225 capillary GC column (30 m × 250 µm, 0.25 µm), method: 60 °C (hold 0 min), 60 °C to 100 °C (5 °C/min), 100 °C (hold 5 min), 100 °C to 220 °C (20 °C/min) and 220 °C (hold 5 min).

Starting Material:

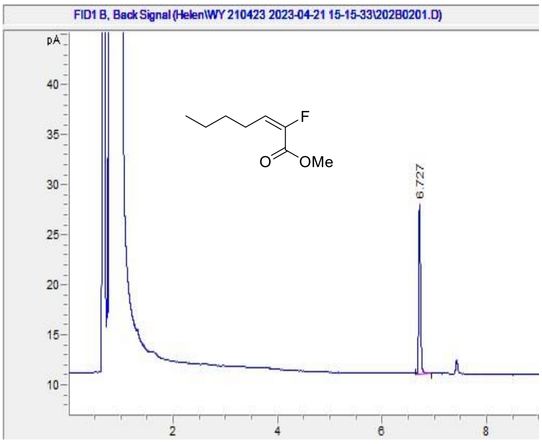


Racemic Standard:

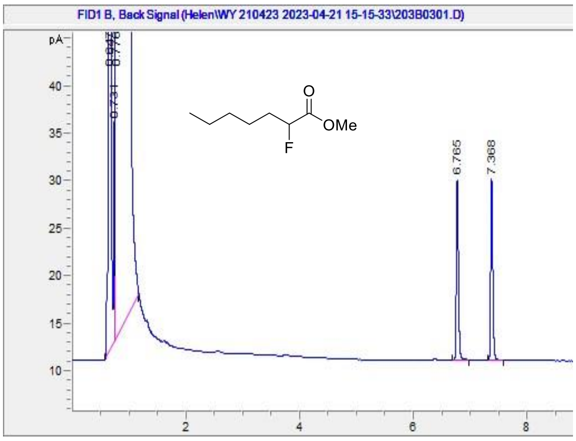


Reaction Trace:

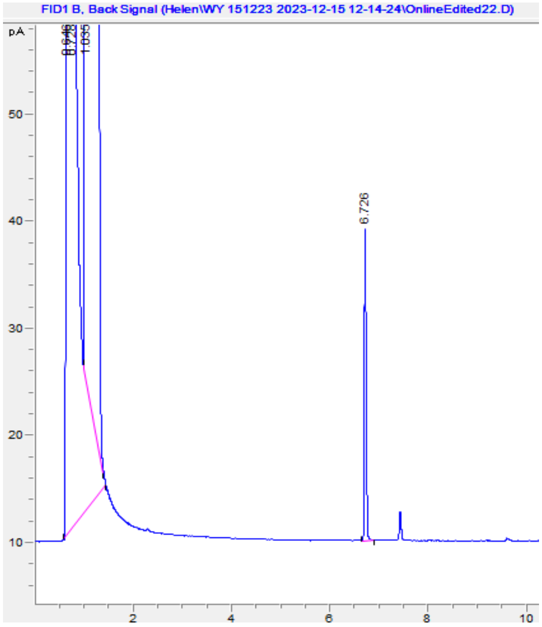


Reaction and Racemic Standard mix:

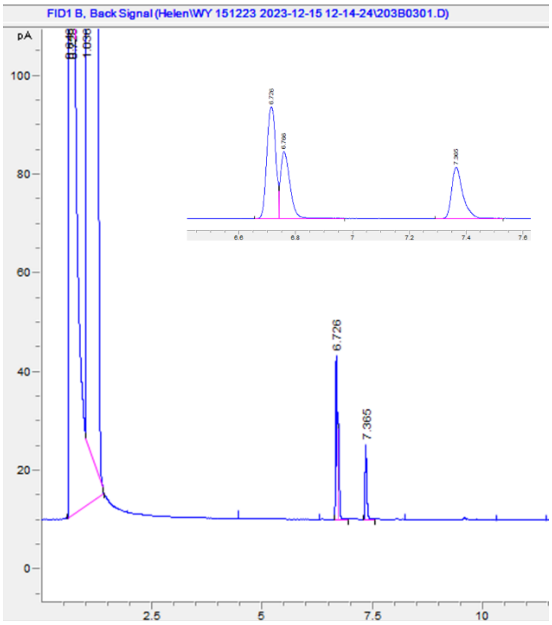

## (*R*)-(2-fluoro-3-methylbut-3-en-1-yl)benzene (13)

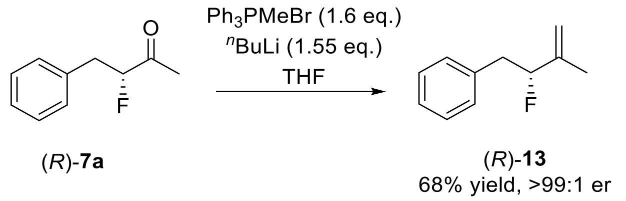


| Compound | $t_R$ |
|---|---|
| (*R*)-**13** | 8.1 min |
| (*S*)-**13** | 8.6 min |

**Conditions:** HPLC, 100% hexane, 0.5 mL/min, AD-H column.

Racemic Standard:

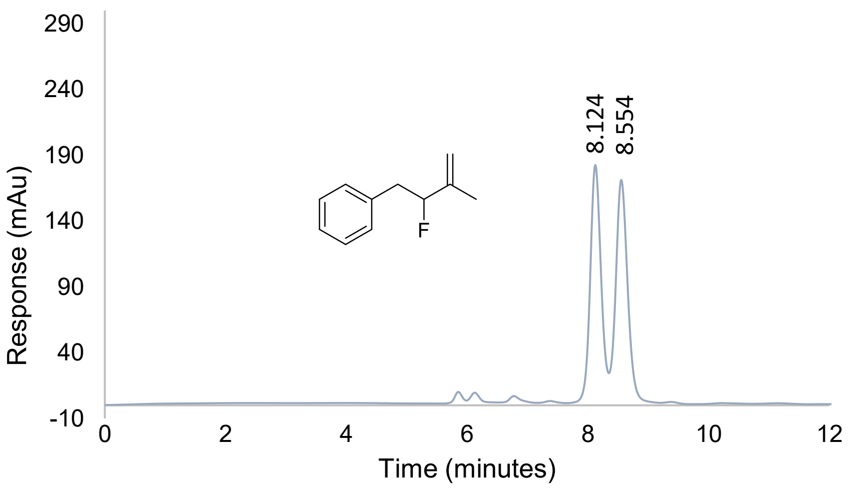


Reaction Product:

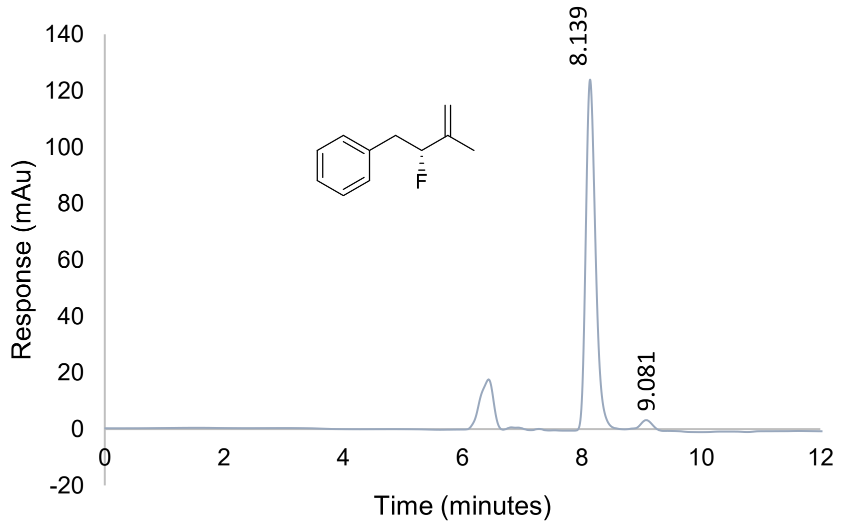

## (*R*)-3-fluoro-2-methyl-4-phenylbutan-2-ol (14)

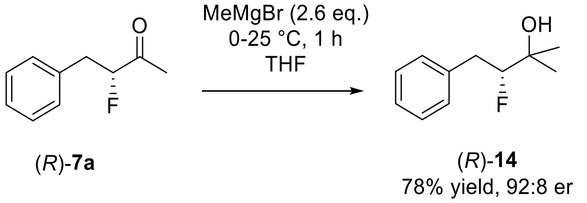


| Compound | $t_R$ |
|---|---|
| (*R*)-**14** | 11.2 min |
| (*S*)-**14** | 13.5 min |

**Conditions:** HPLC, 1% IPA/hexane, 1 mL/min, AD-H column.

Racemic Standard:

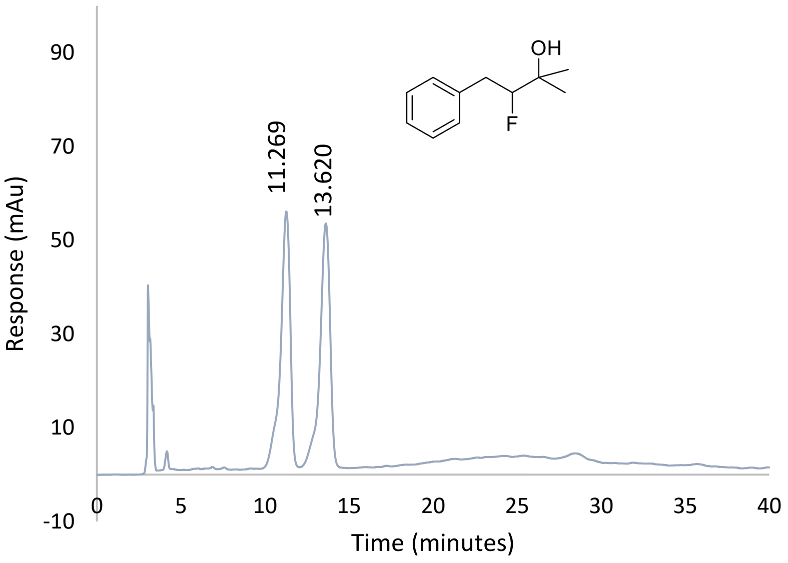


Reaction Product:

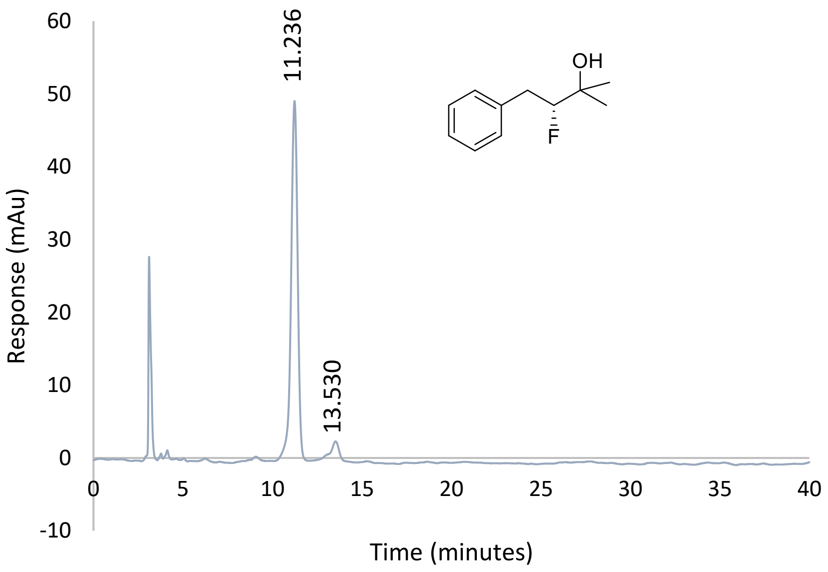

# (2*R*,3*S*)-3-Fluoro-4-phenylbutan-2-ol (15) Enzyme reduction

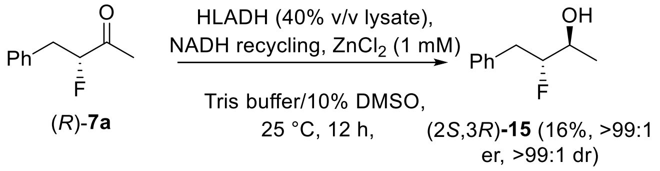


| Compound | $t_R$ |
|---|---|
| (*R*)-**7a** | 6.1 min |
| (2*S*,3*S*) | 15.2 min |
| (2*R*,3*R*) | 16.8 min |
| (2*S*,3*R*) | 17.9 min |
| (2*R*,3*S*) | 23.8 min |

**Conditions:** HPLC, 1% IPA/hexane, 1 mL/min, OD-H column. A different HPLC machine was used compared to the ene reductase reaction above, this accounts for the different retention time of (*R*)-**7a**.

Starting Material:

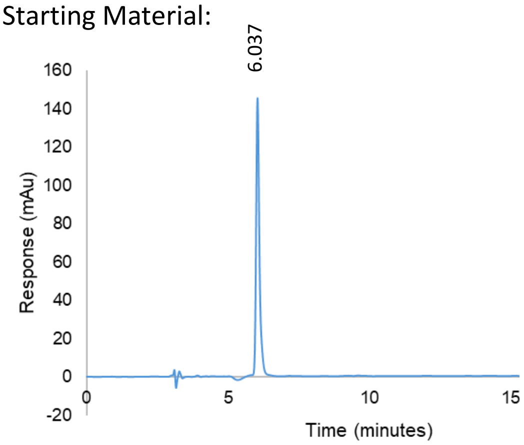


Reference Standard:

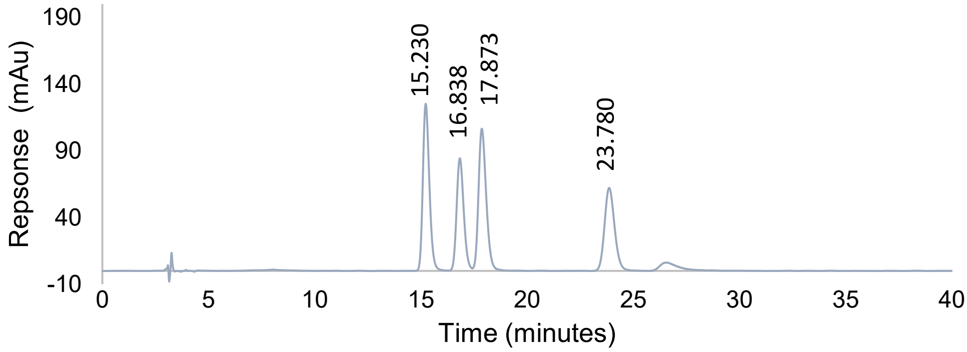


Reaction Product:

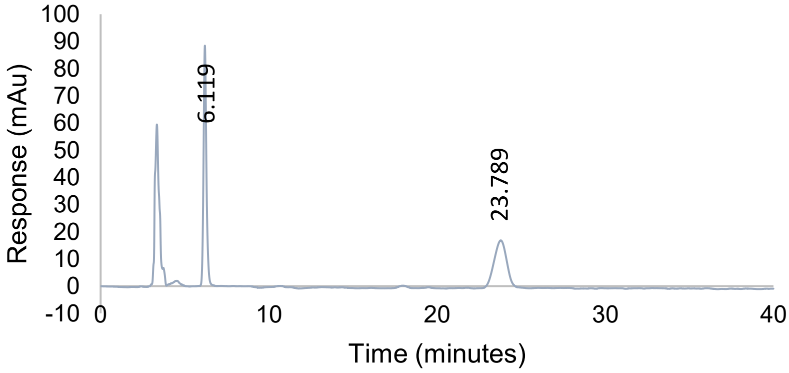

## 3-Fluoro-4-phenylbutan-2-ol (15) $NaBH_4$ reduction

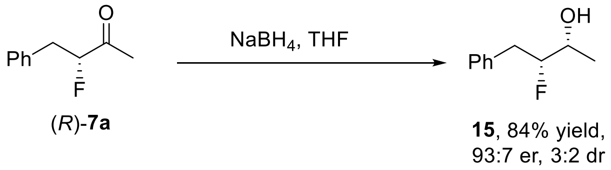


| Compound | $t_R$ |
|---|---|
| (2*R*,3*S*) | 15.2 min |
| (2*R*,3*R*) | 16.8 min |
| (2*S*,3*R*) | 17.9 min |
| (2*R*,3*S*) | 23.8 min |

**Conditions:** HPLC, 1% IPA/hexane, 1 mL/min, OD-H column.

Reference Standard:

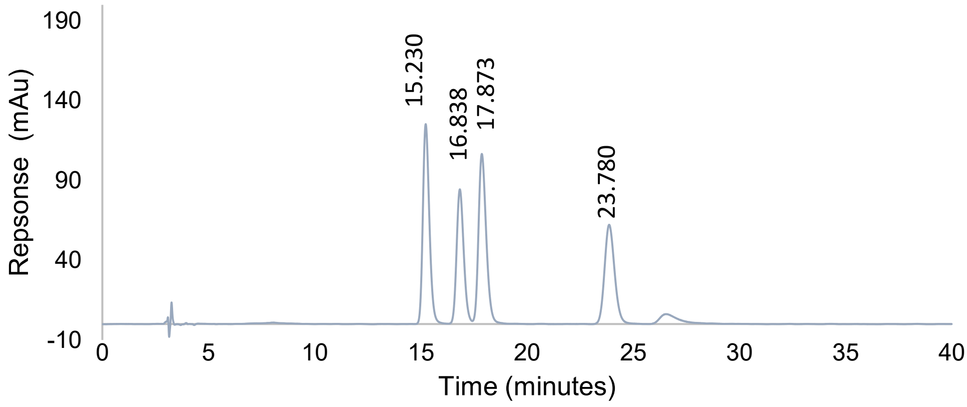


Reaction Product:

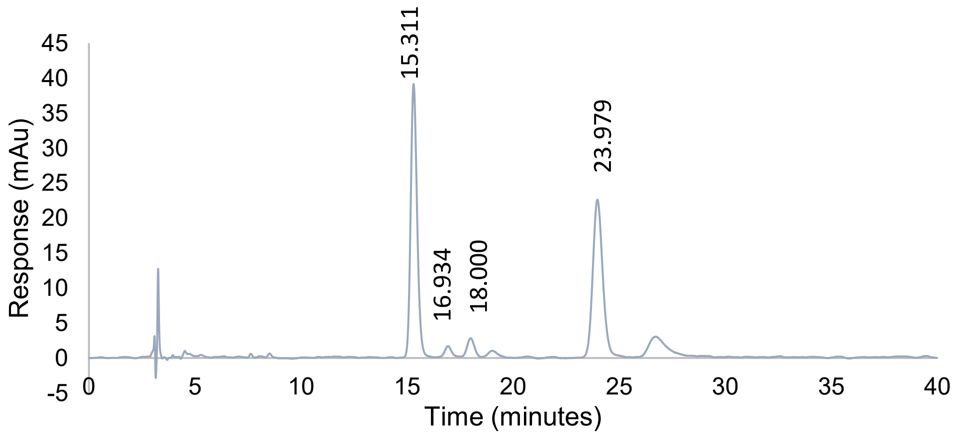

# NMR Spectra

## Diethyl(1-fluoro-2-oxopropyl)phosphonate

$^{1}H$ NMR, $CDCl_3$, 700 MHz

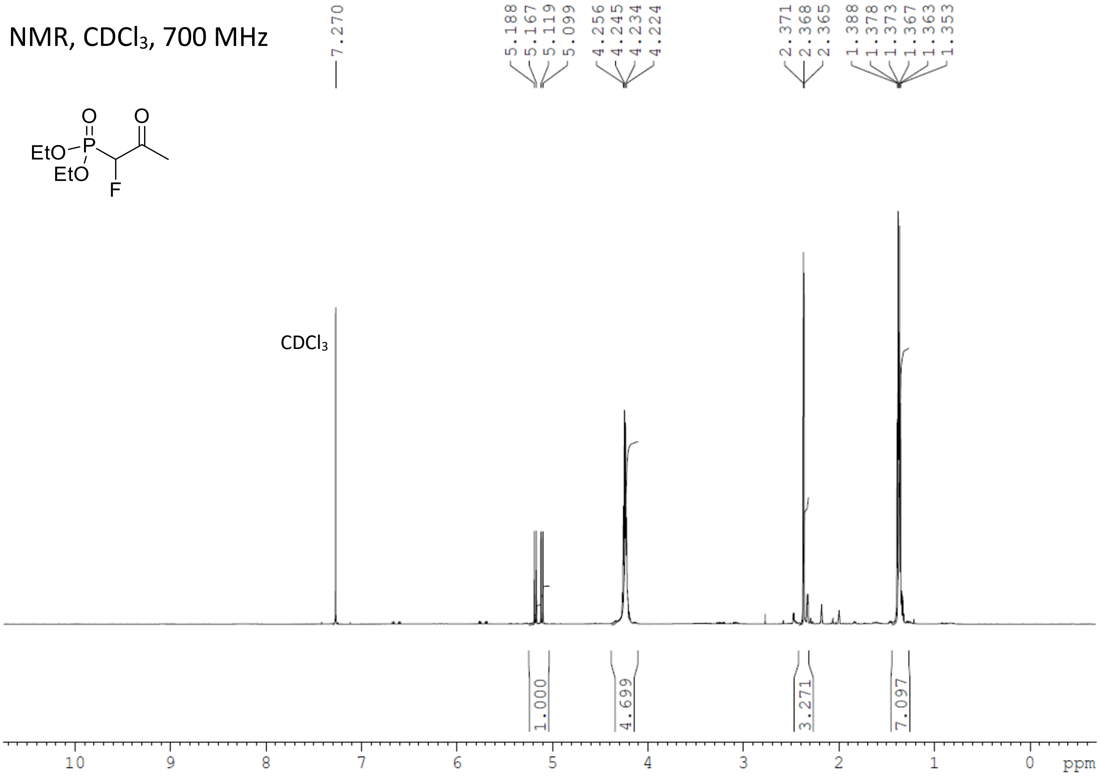


$^{13}C$ NMR, $CDCl_3$, 176 MHz

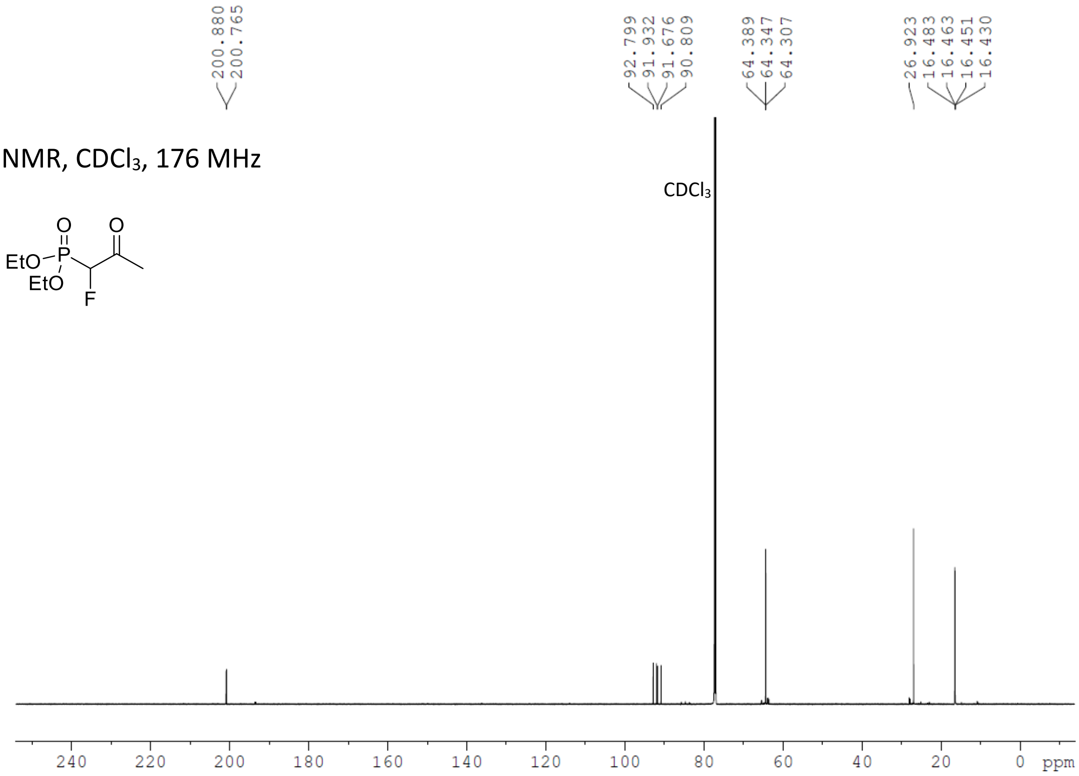

$^{19}F$ NMR, $CDCl_3$, 376 MHz

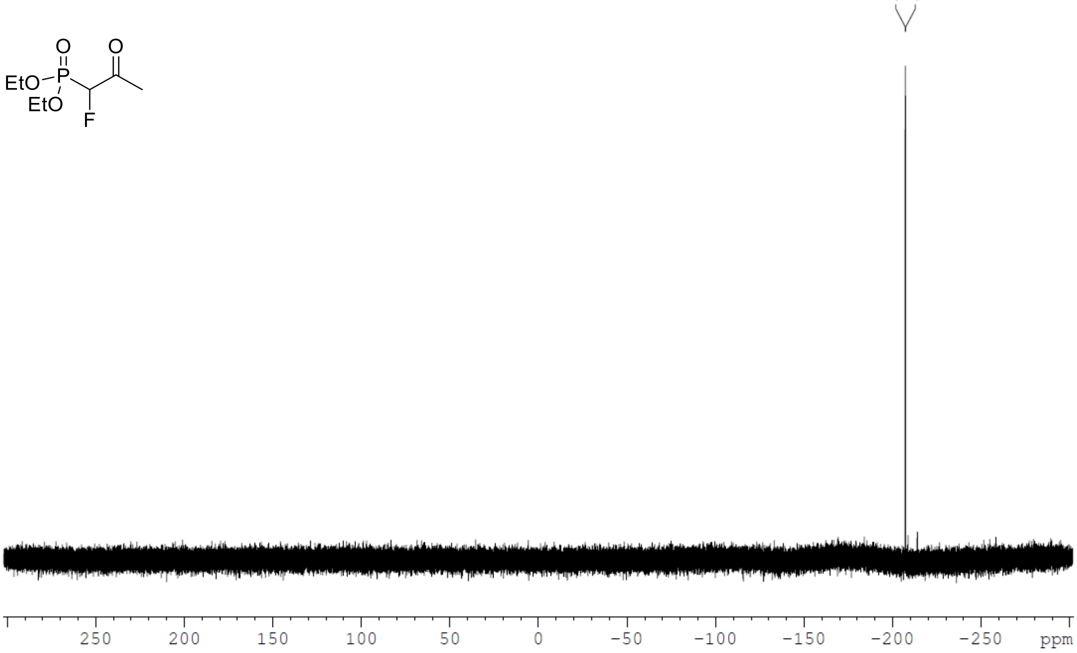


$^{31}P$ NMR, $CDCl_3$, 283 MHz

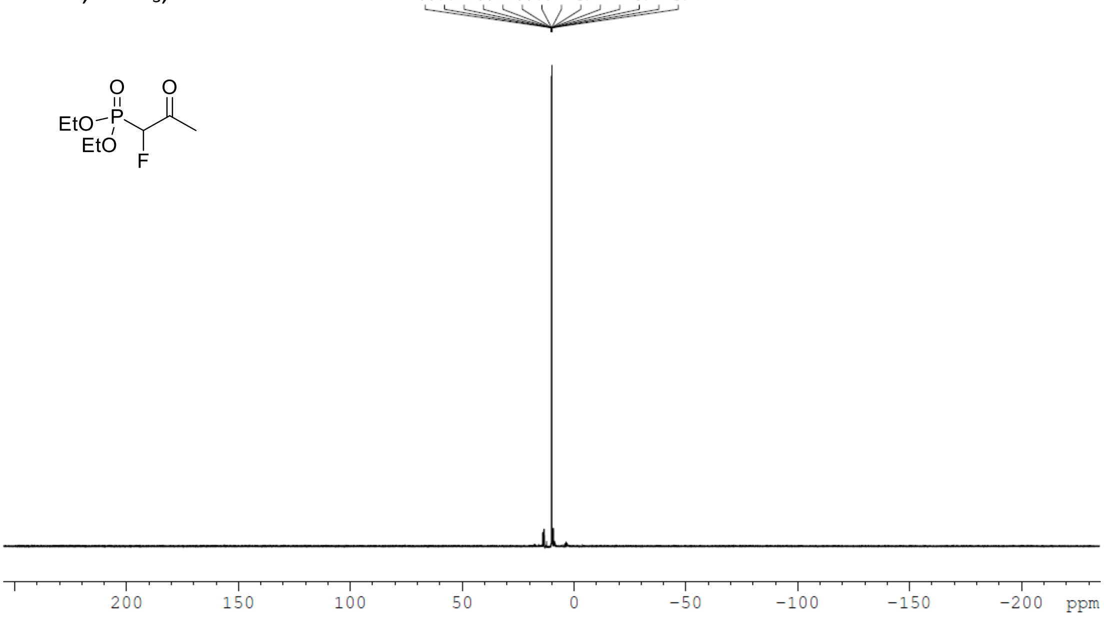

# Diethyl(1-fluoro-2-oxobutyl)phosphonate

$^{1}$H NMR, $CDCl_3$, 700 MHz

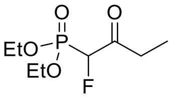


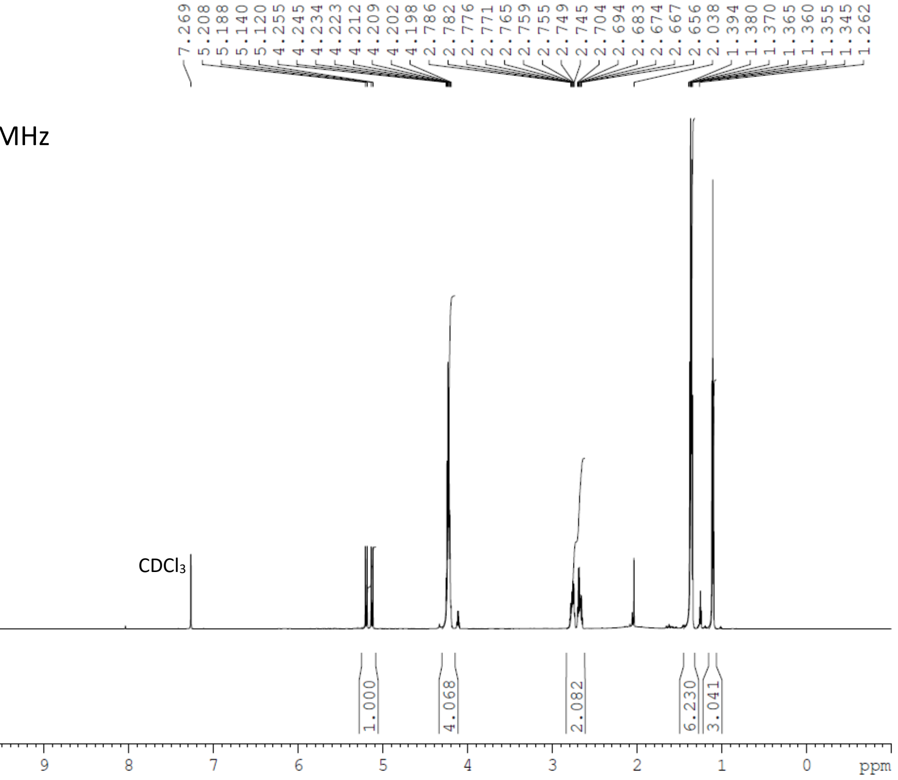


$^{13}$C NMR, $CDCl_3$, 176 MHz

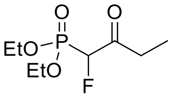


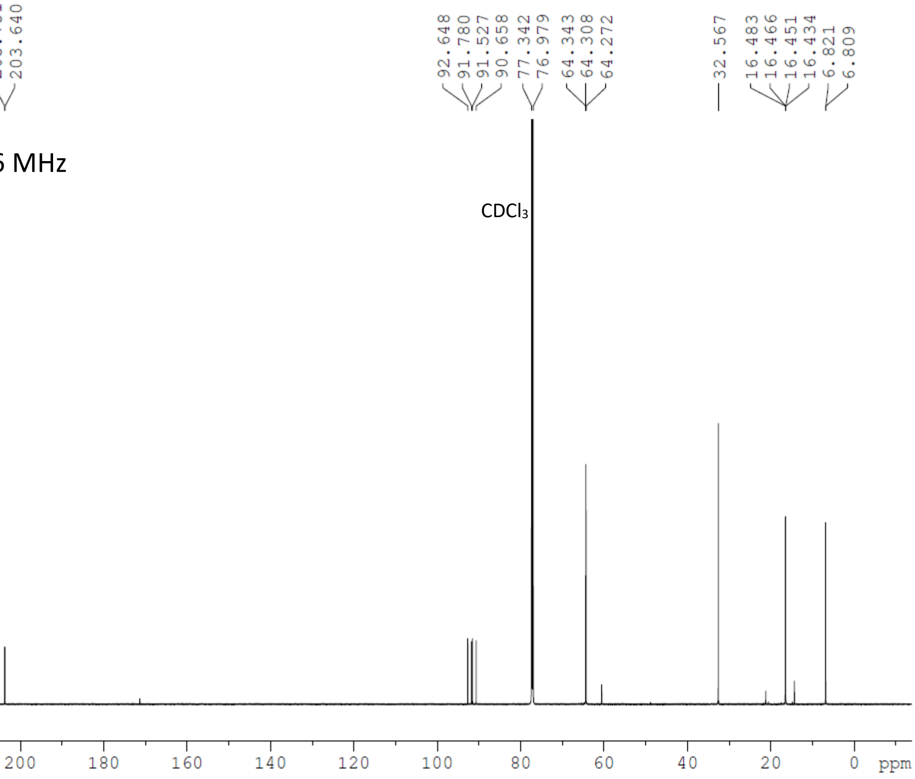

$^{19}F$ NMR, $CDCl_3$, 659 MHz

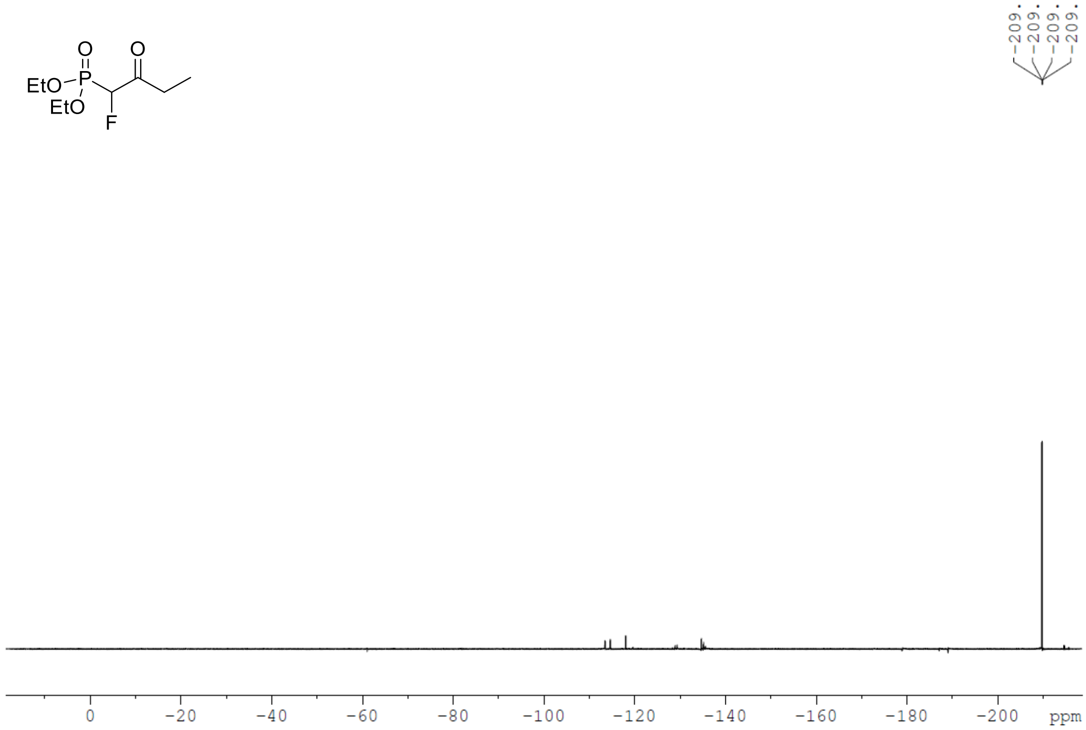


$^{31}P$ NMR, $CDCl_3$, 283 MHz

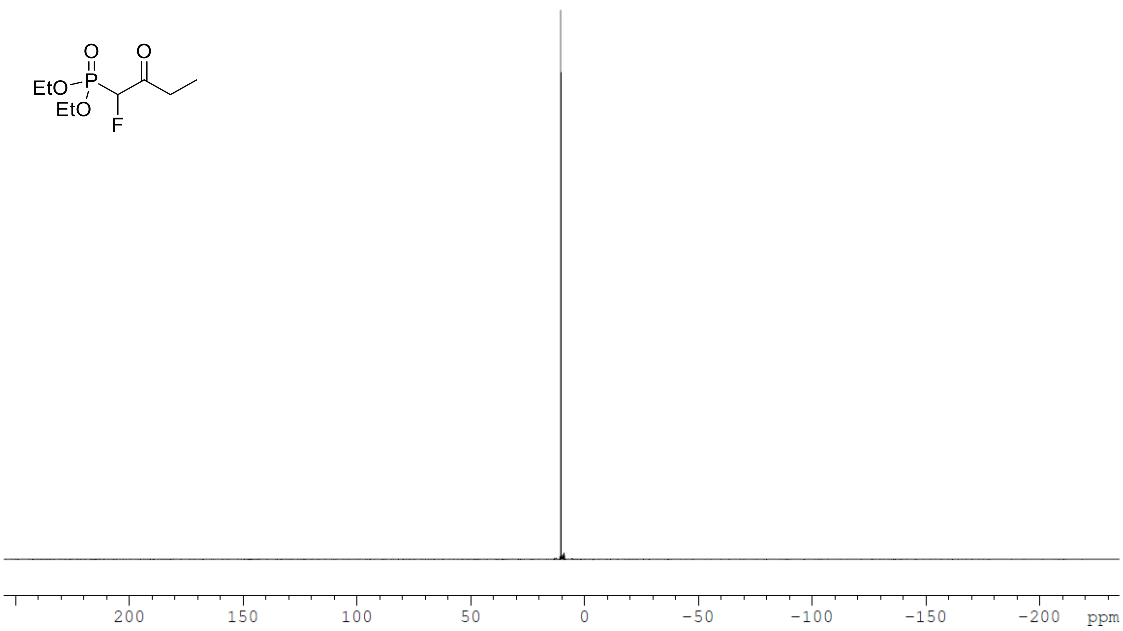

# (Diethoxyphosphoryl)fluoroacetic acid

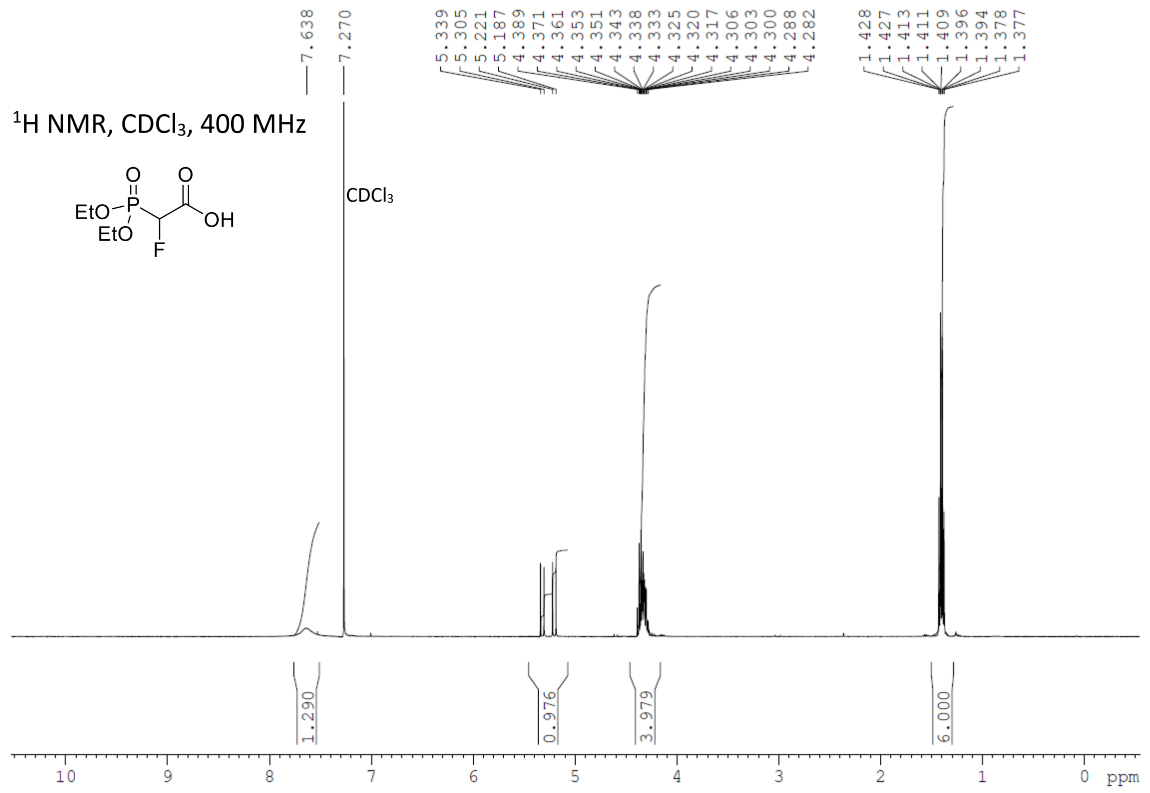


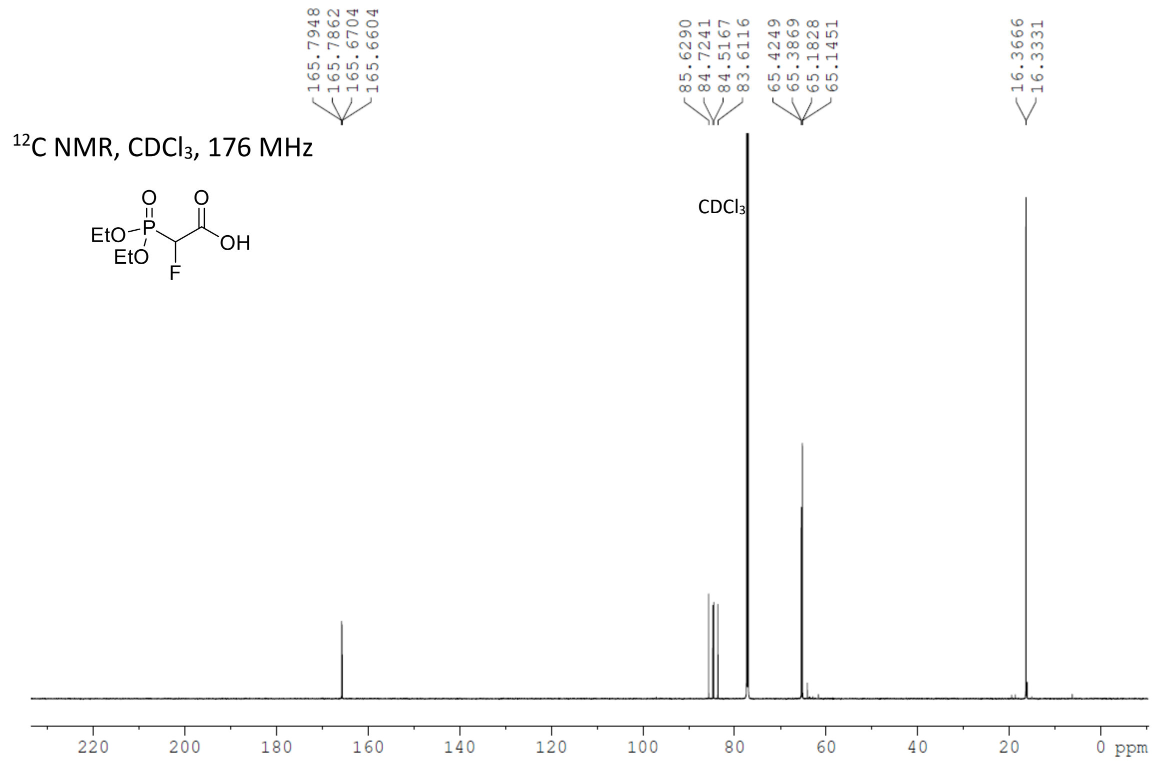

$^{19}F$ NMR, $CDCl_3$, 659 MHz

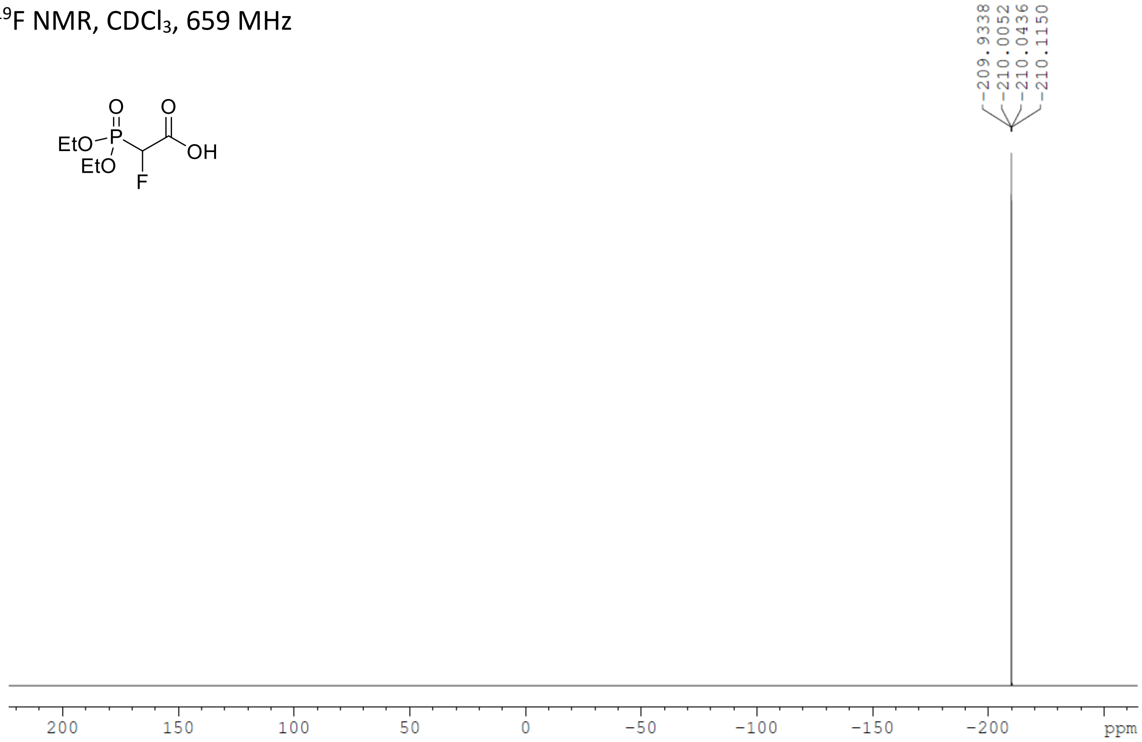


$^{31}P$ NMR, $CDCl_3$, 162 MHz

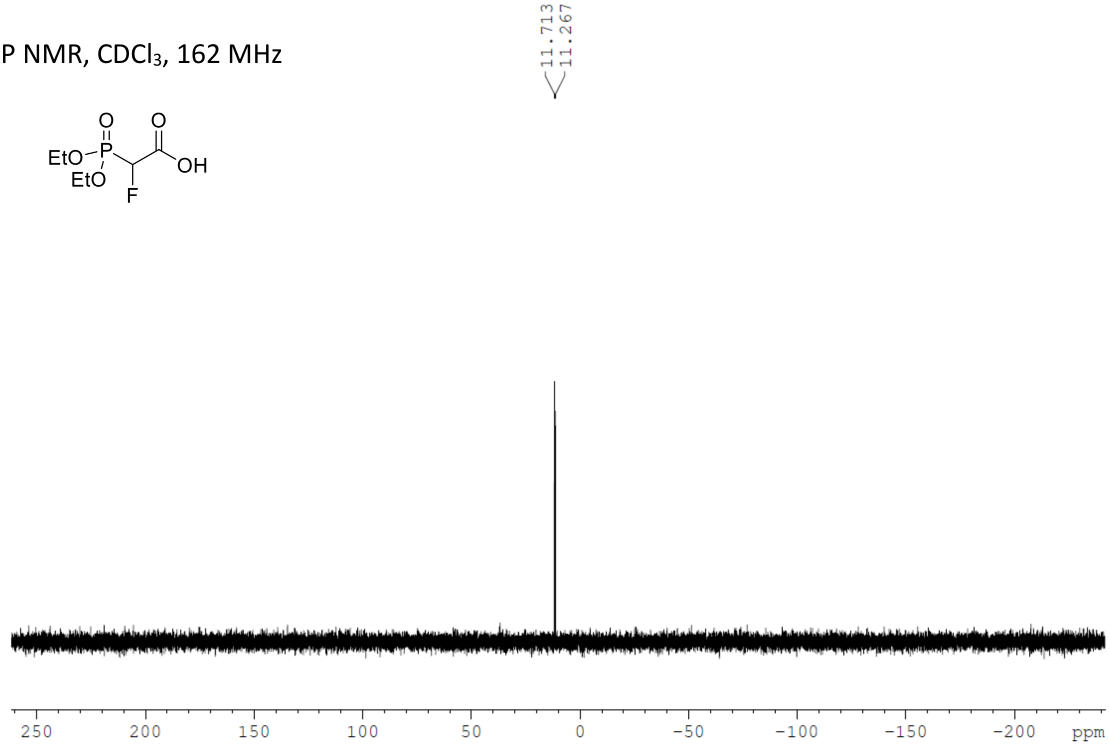

# Diethyl(1-fluoro-2-oxo-2-phenylethyl)phosphonate

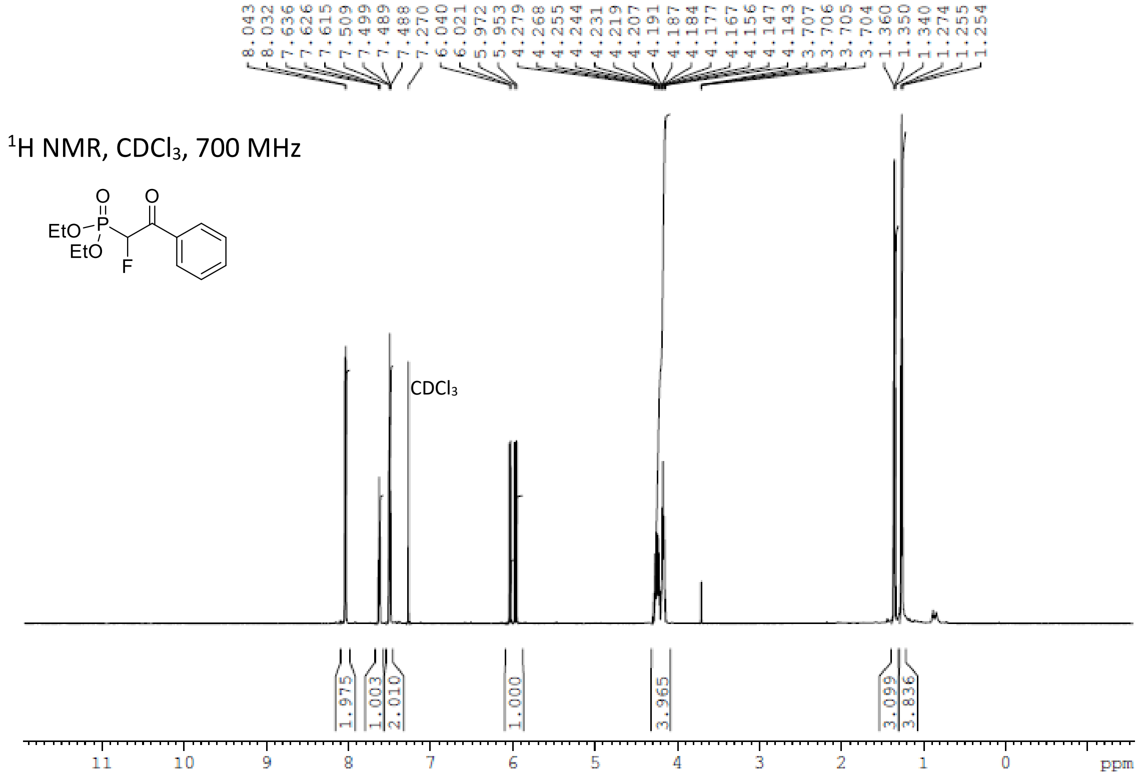


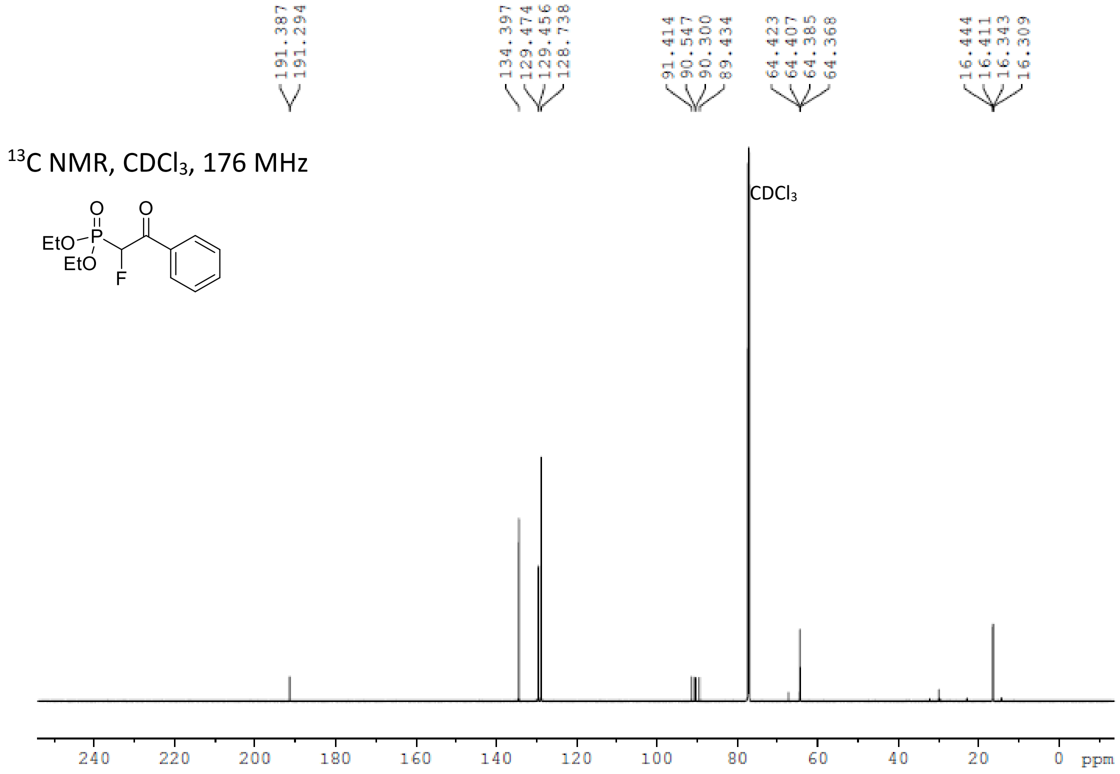

$^{19}F$ NMR, $CDCl_3$, 659 MHz

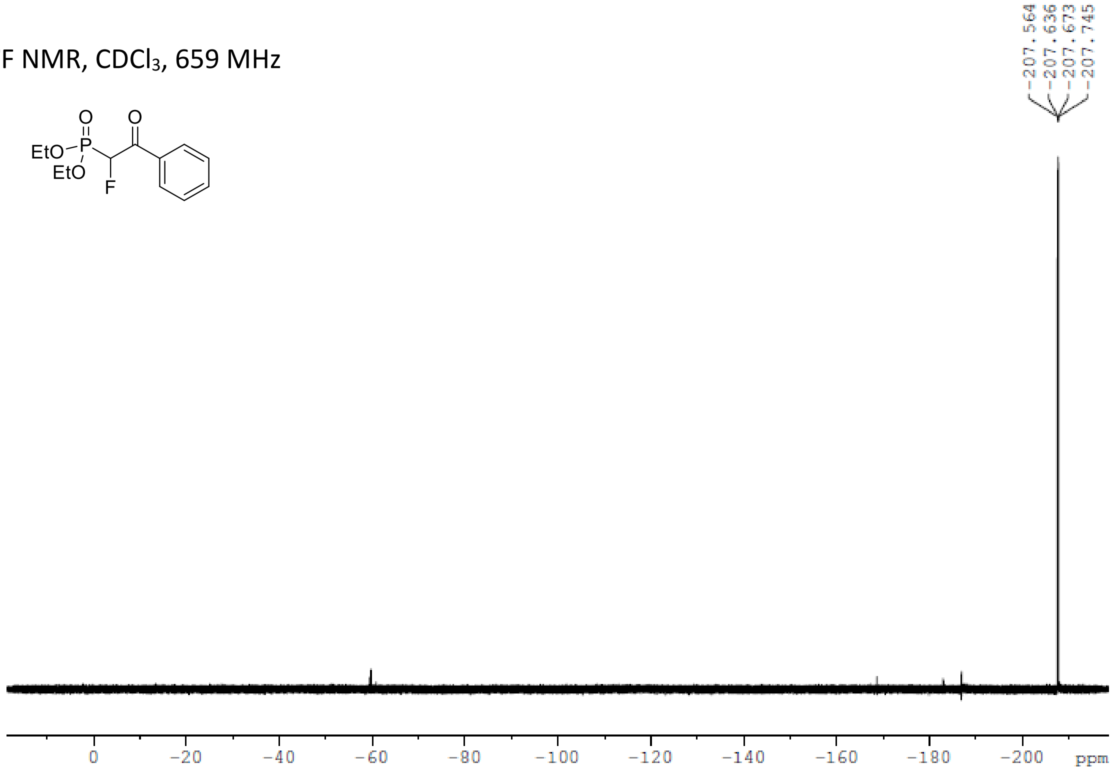


$^{31}P$ NMR, $CDCl_3$, 283 MHz

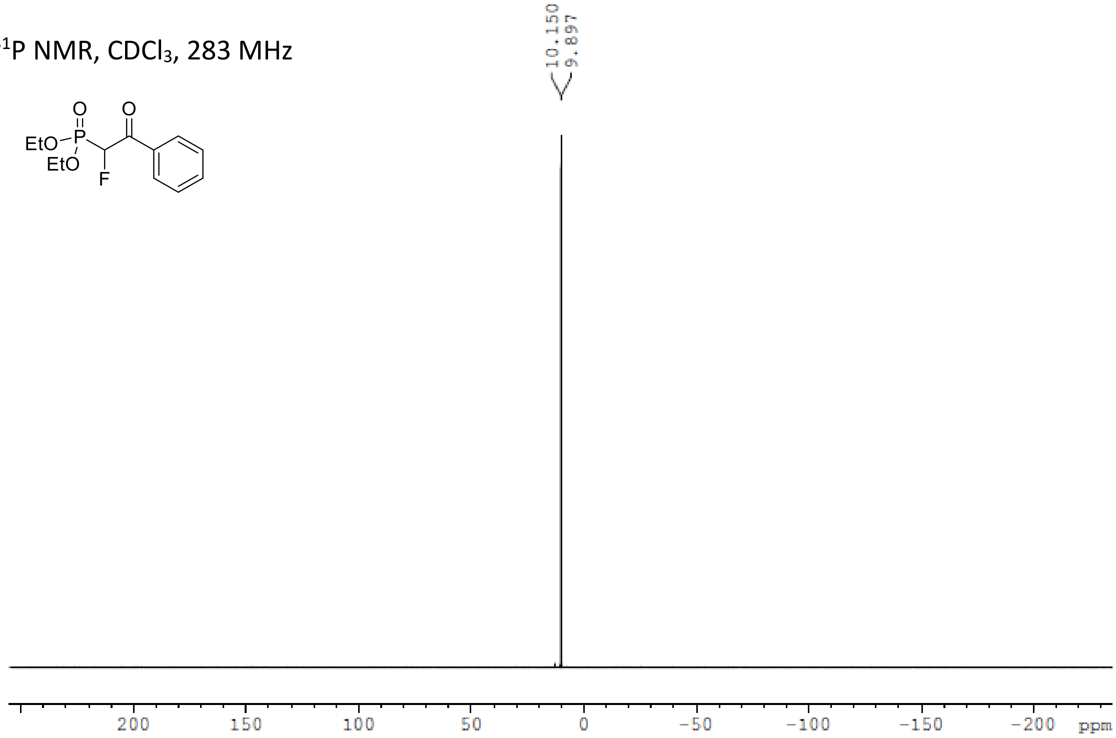

Diethylmethyl-1-fluoromethylphosphonocarboxylate

$^{1}H$ NMR, $CDCl_3$, 700 MHz

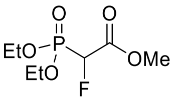


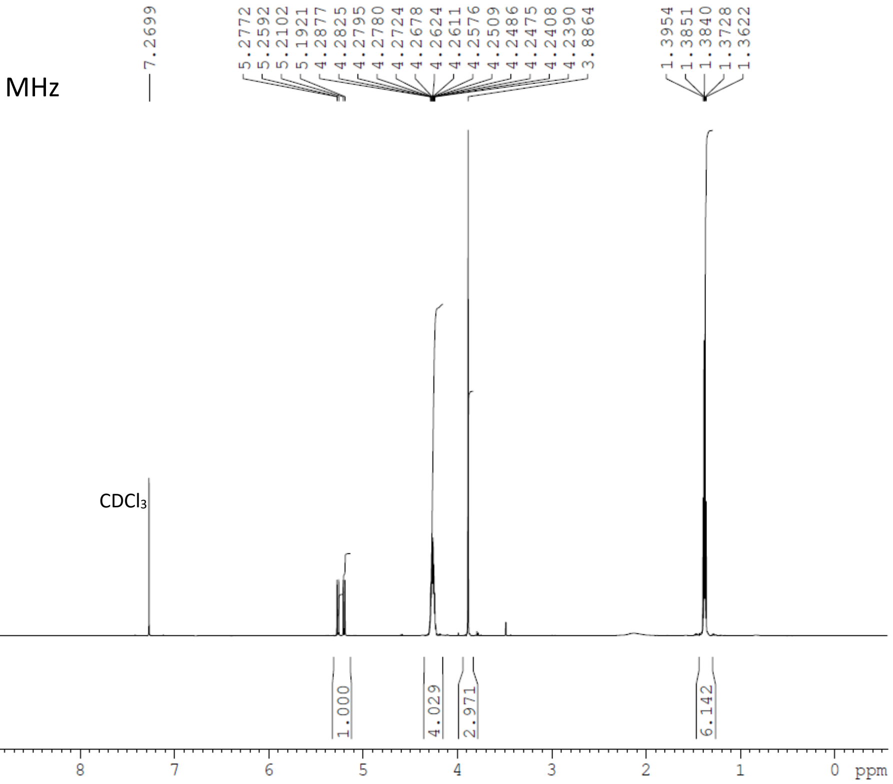


$^{13}C$ NMR, $CDCl_3$, 176 MHz

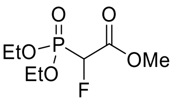


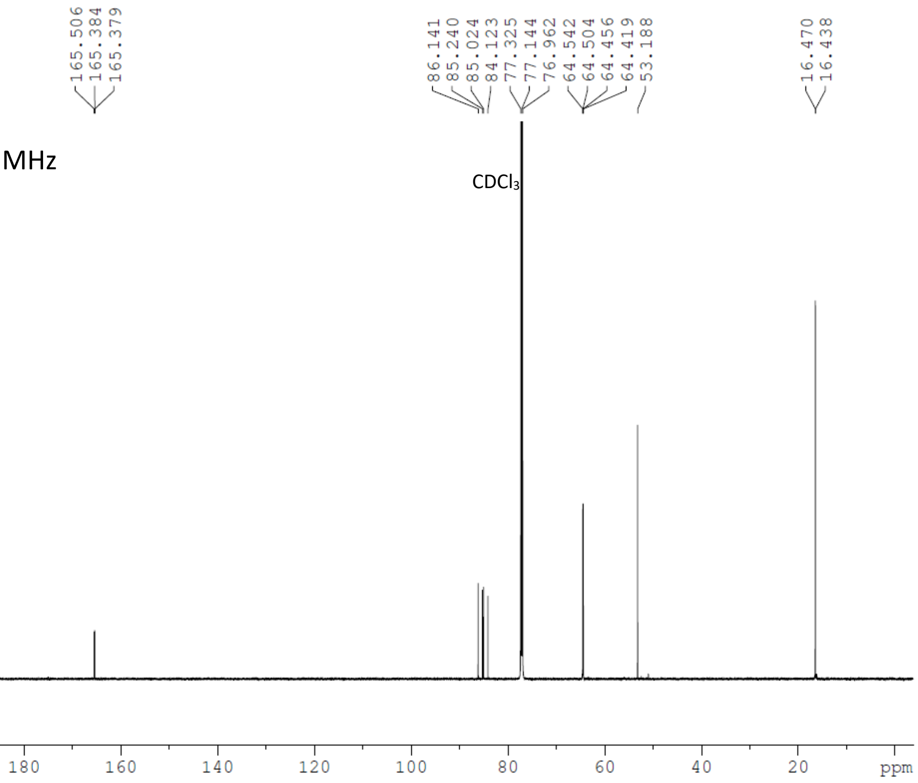

$^{19}F$ NMR, $CDCl_3$, 659 MHz

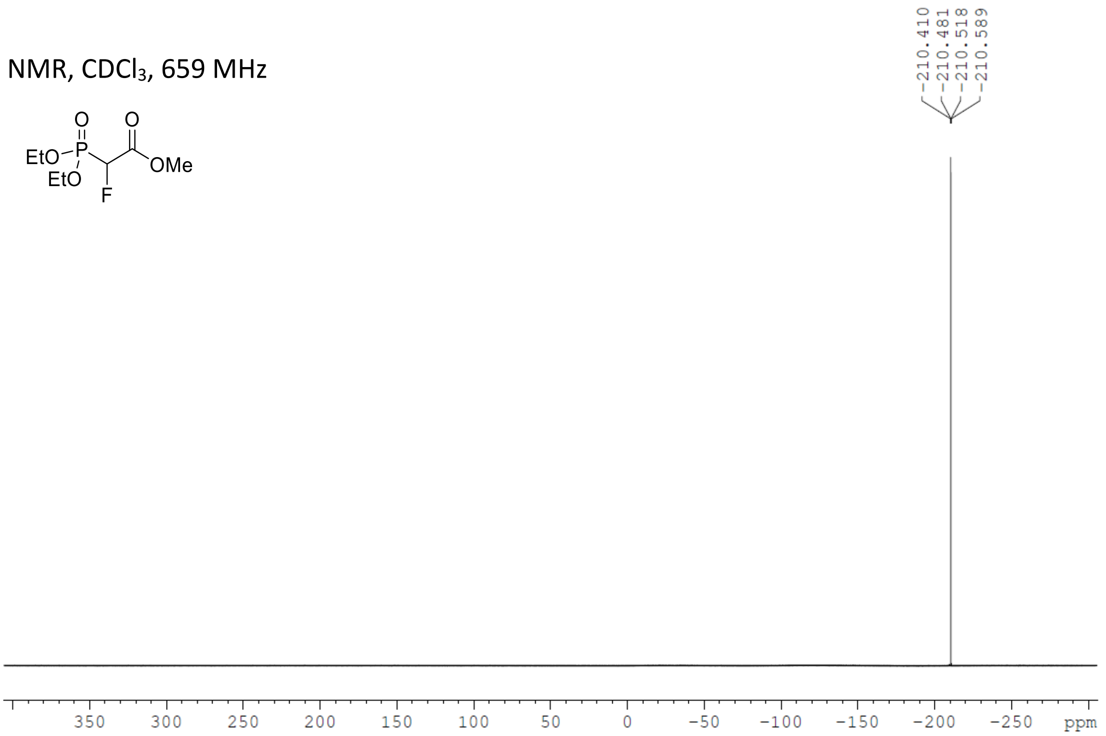


$^{31}P$ NMR, $CDCl_3$, 283 MHz

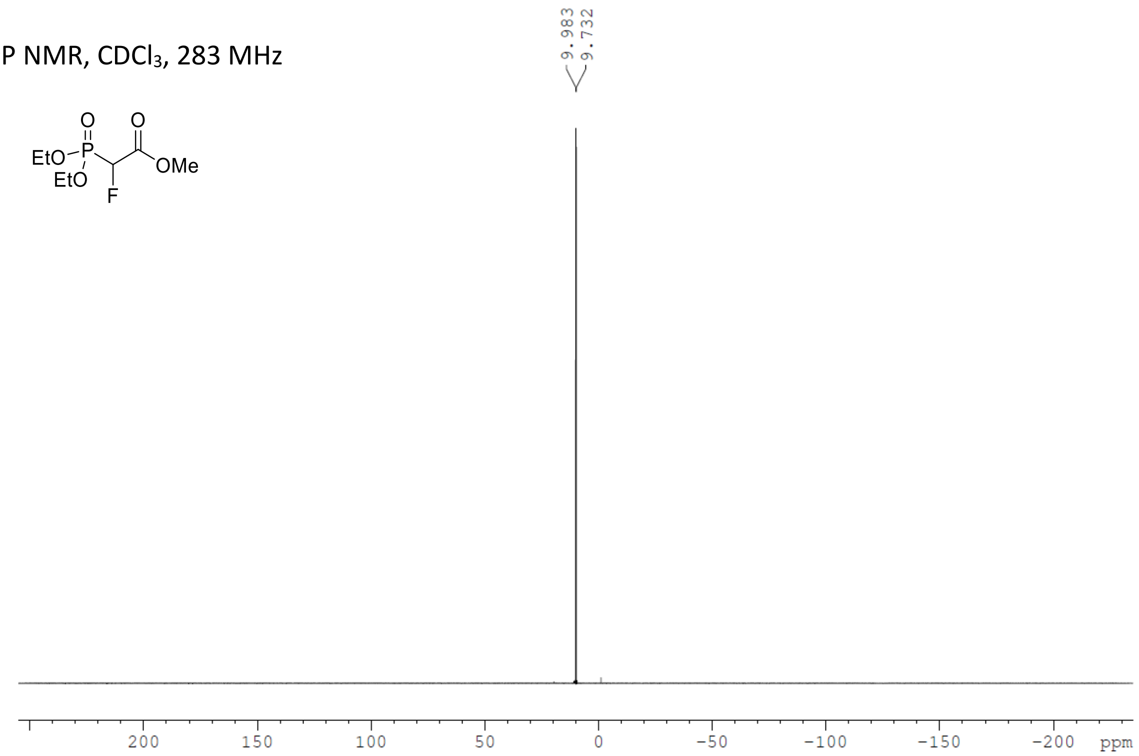

4-(2-Methylphenyl)butan-2-one

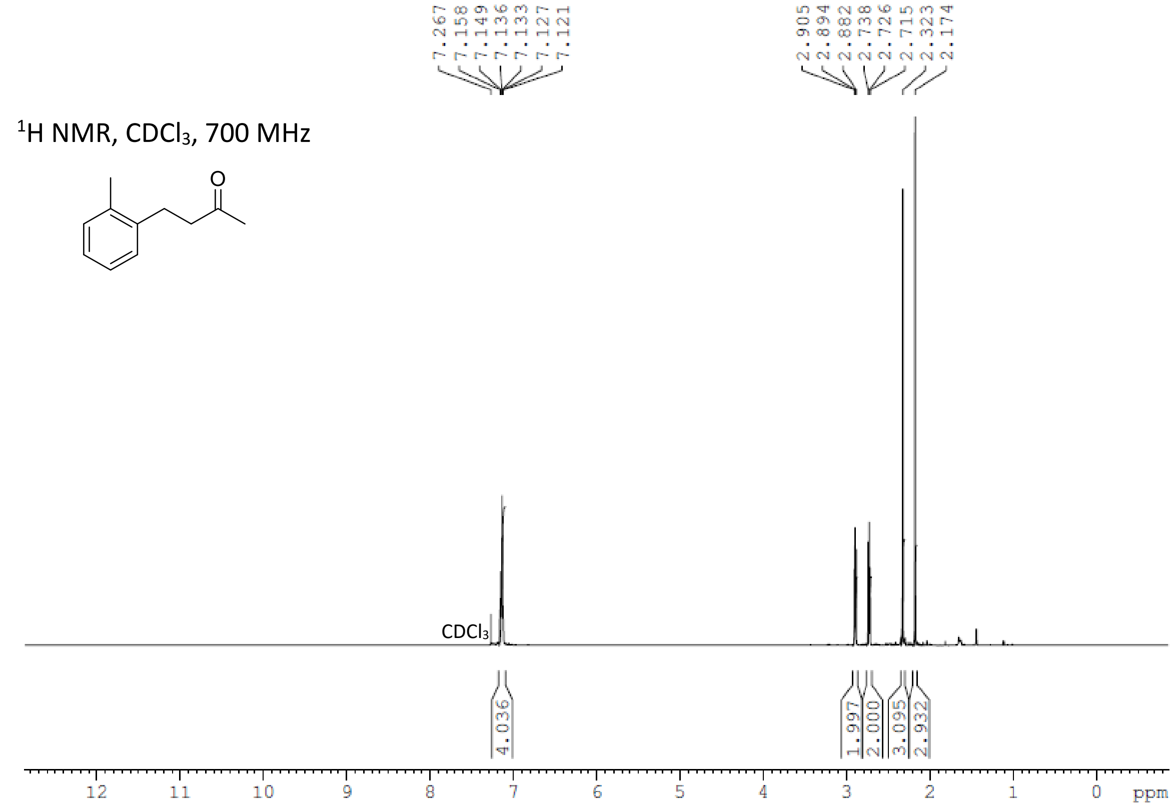


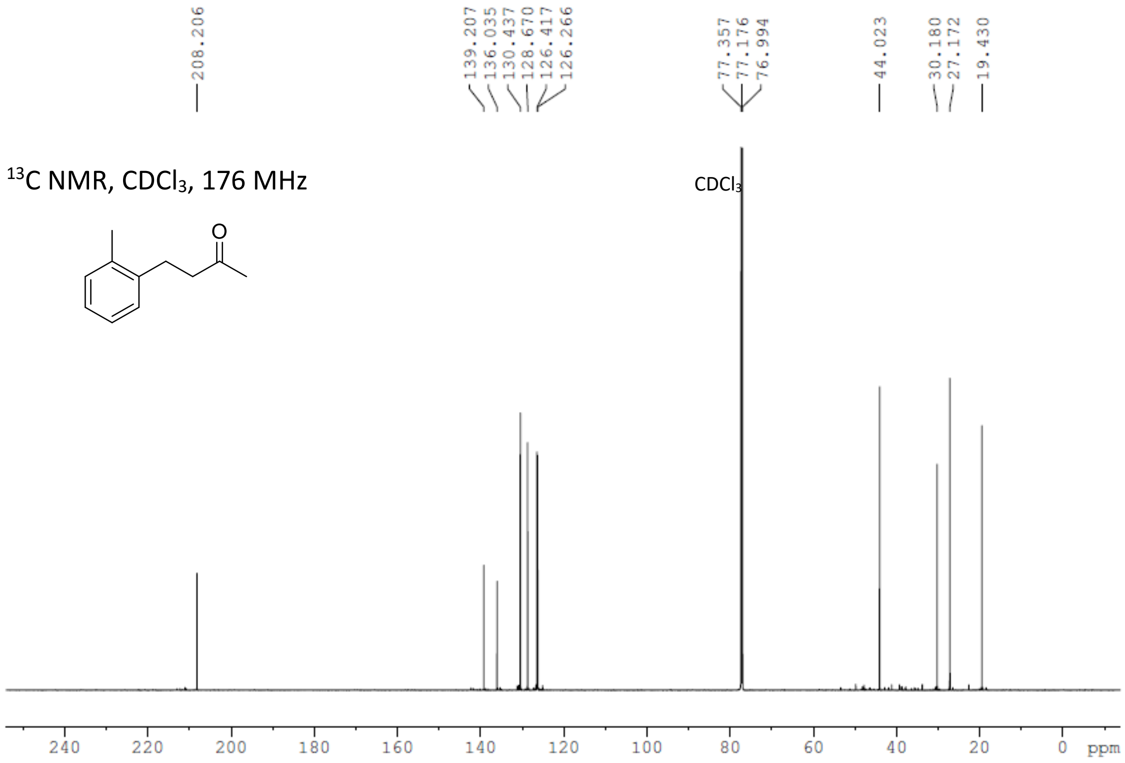

# (*E*)-4-Cyclohexylbut-3-en-2-one

$^{1}$H NMR, $CDCl_3$, 700 MHz

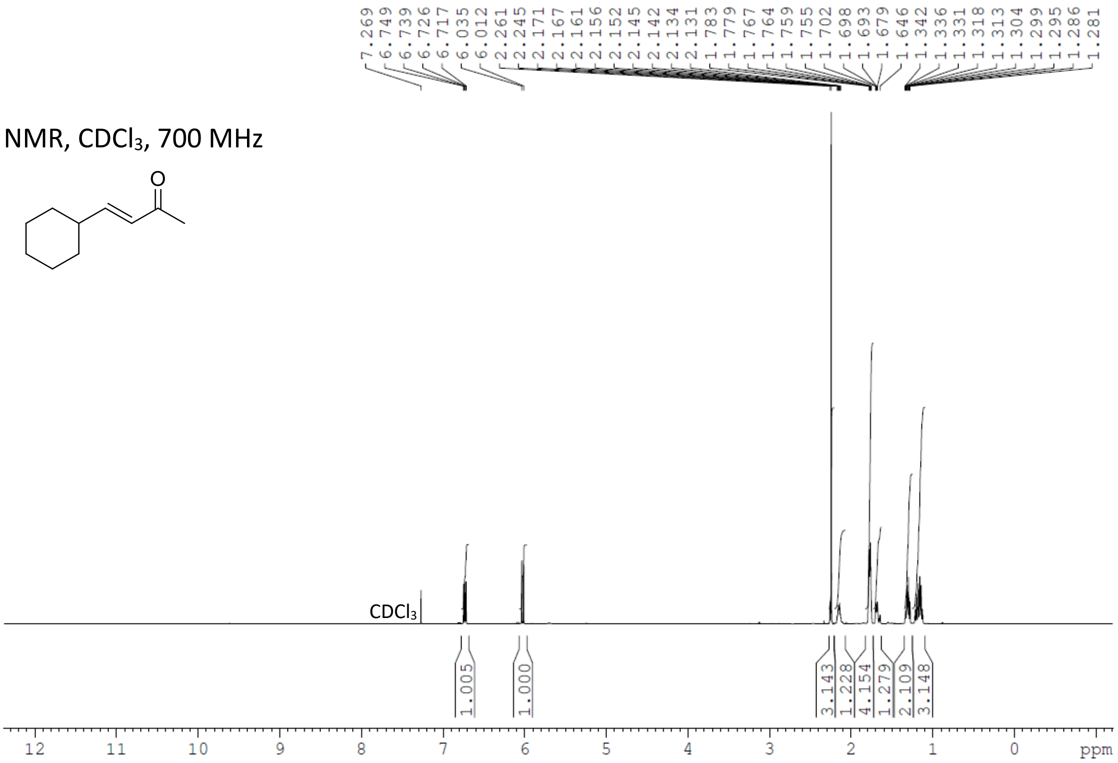


$^{13}$C NMR, $CDCl_3$, 176 MHz

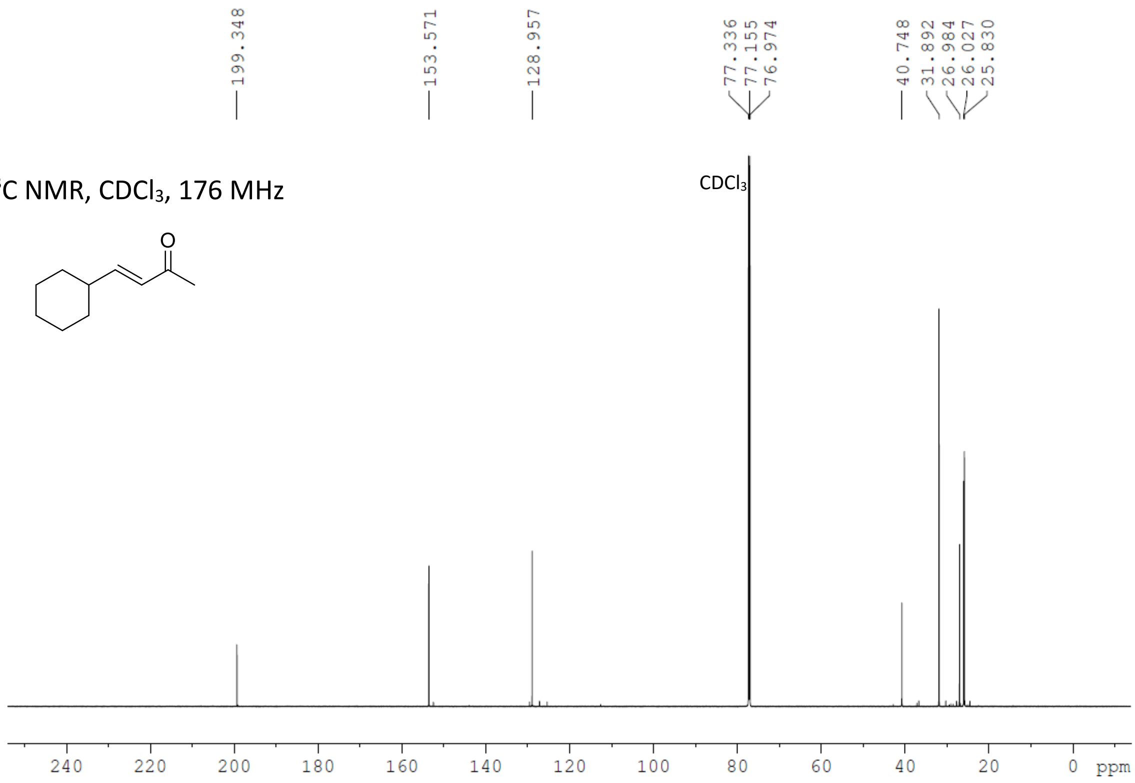

# 4-Cyclohexylbutan-2-one

$^{1}$H NMR, $CDCl_3$, 500 MHz

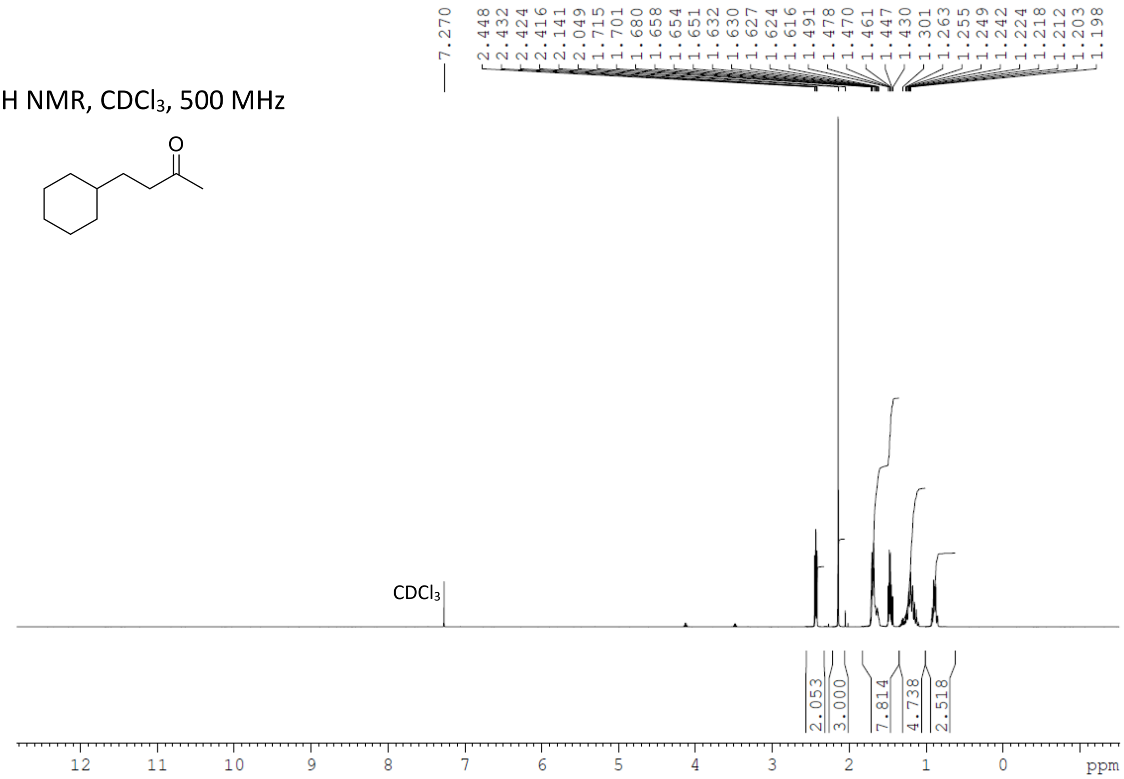


$^{13}$C NMR, $CDCl_3$, 125 MHz

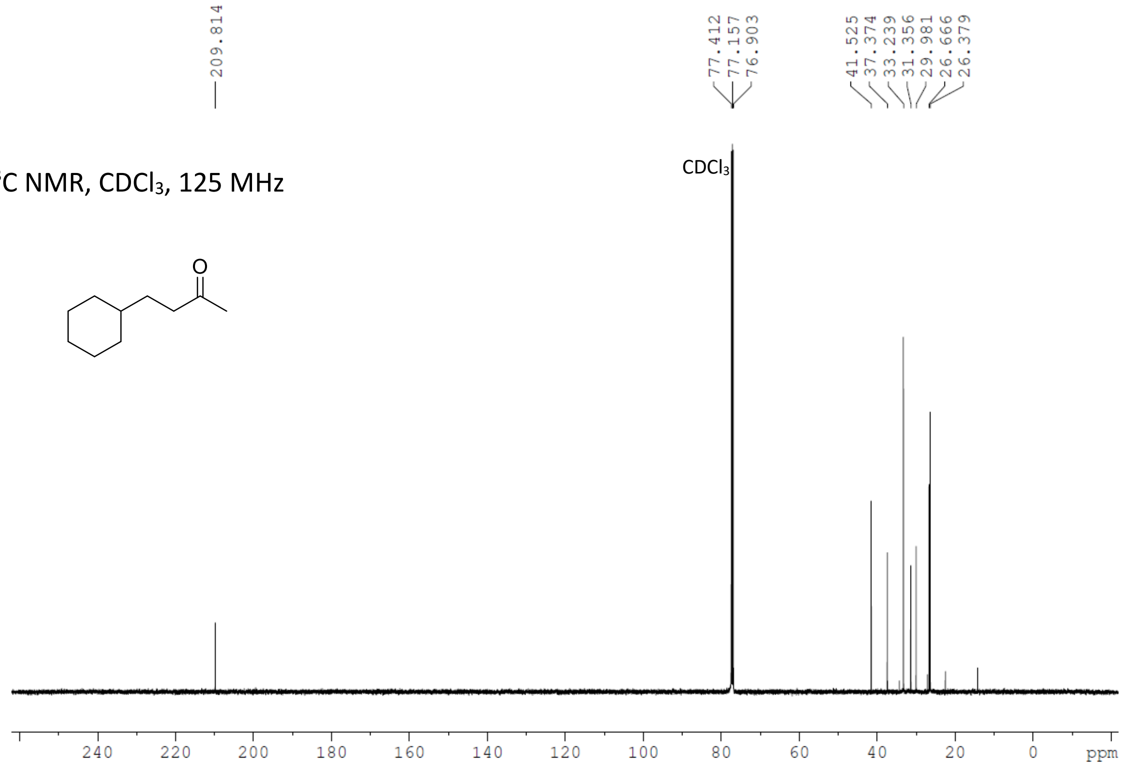

# Allyl 2-oxocyclohexane-1-carboxylate

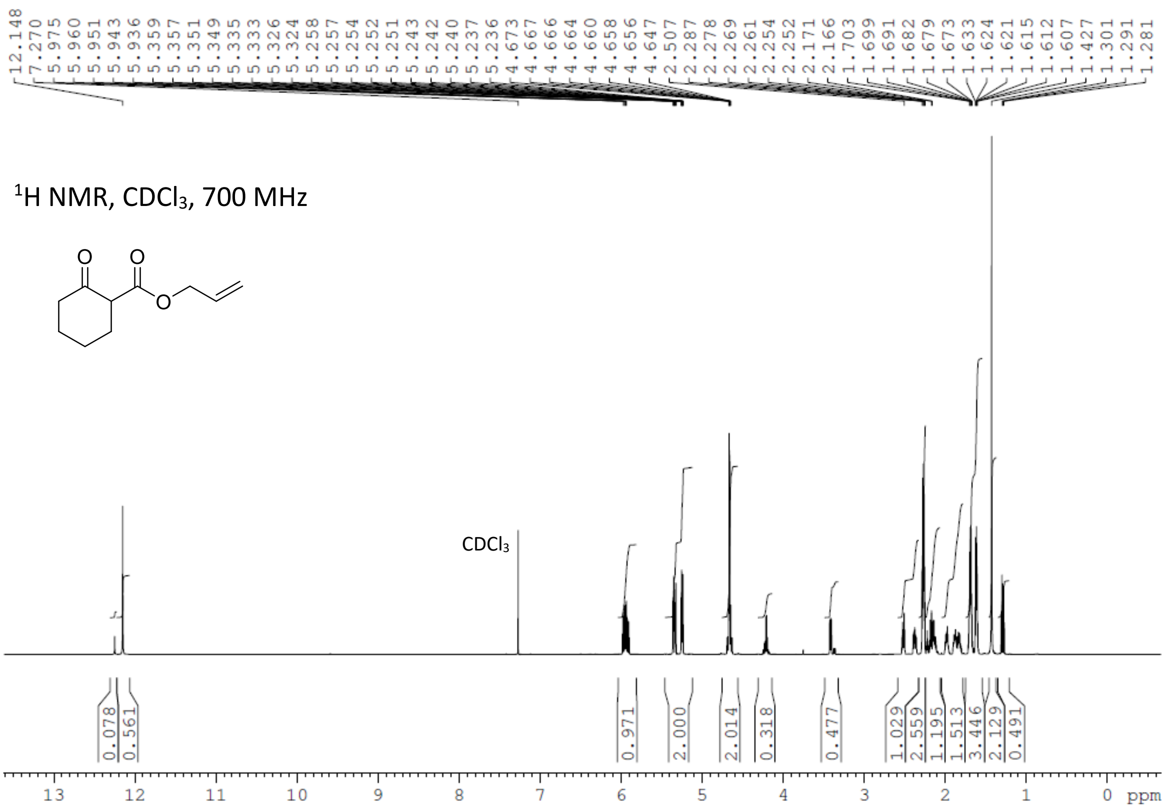


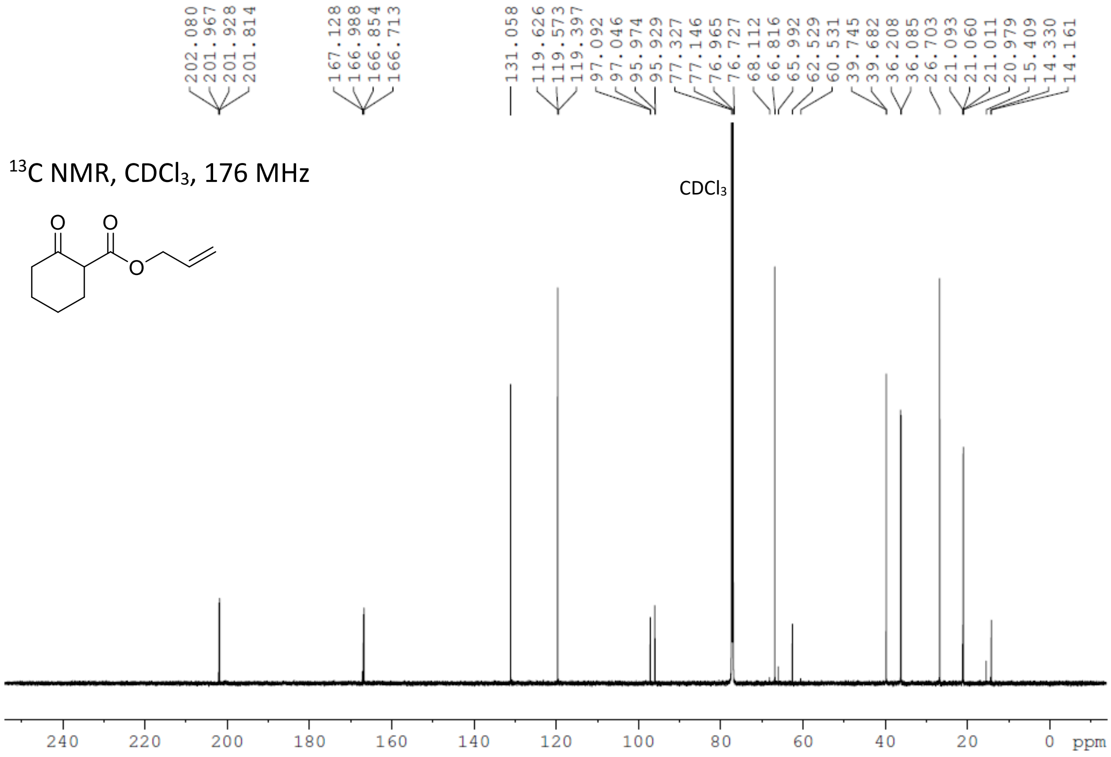

# Allyl 1-fluoro-2-oxocyclohexanonecarboxylate

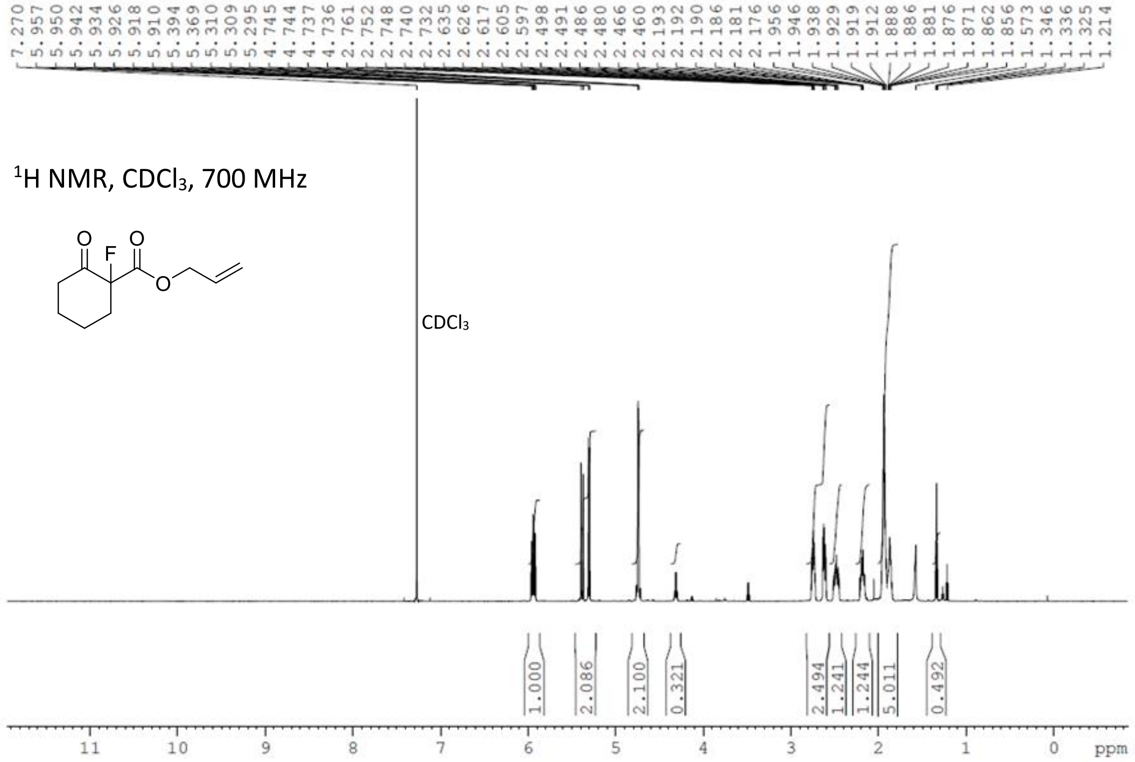


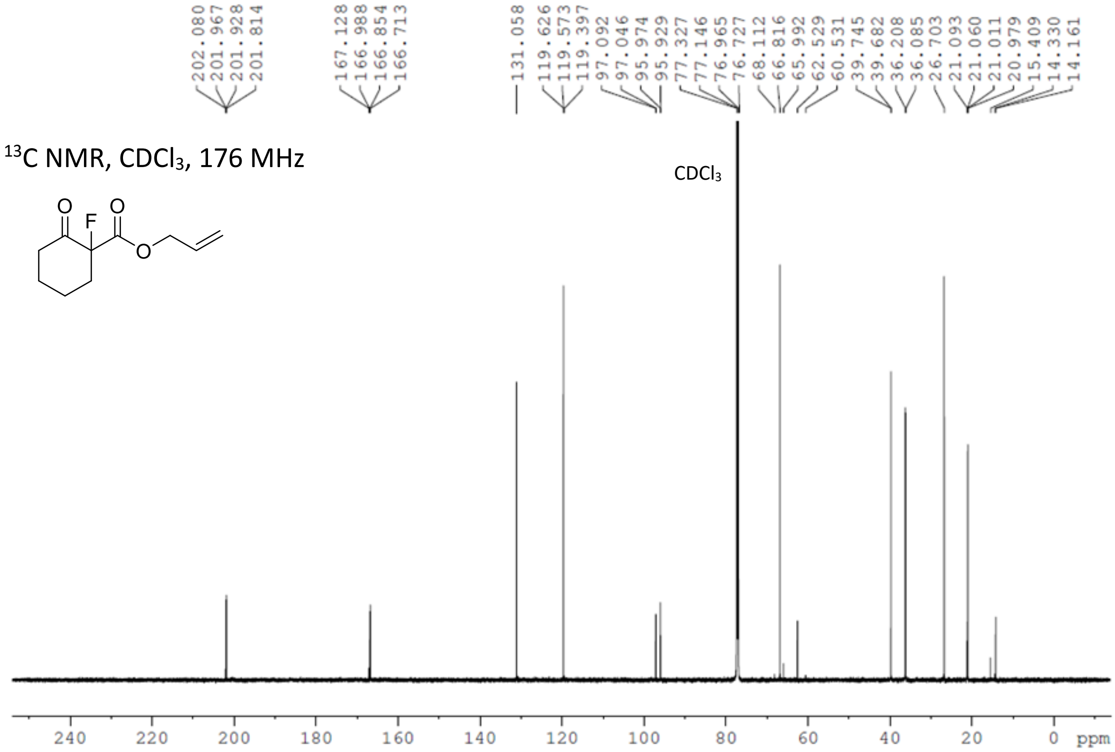

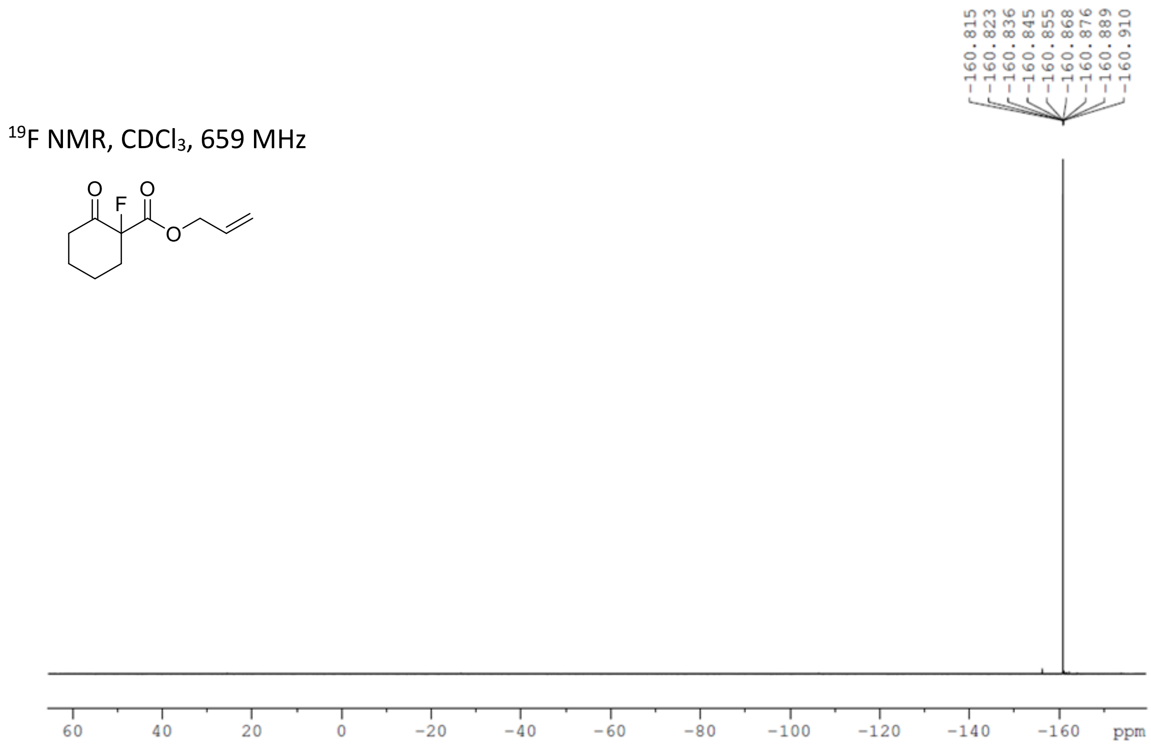


# 2-Chloro-2-fluorocycloheptan-1-one

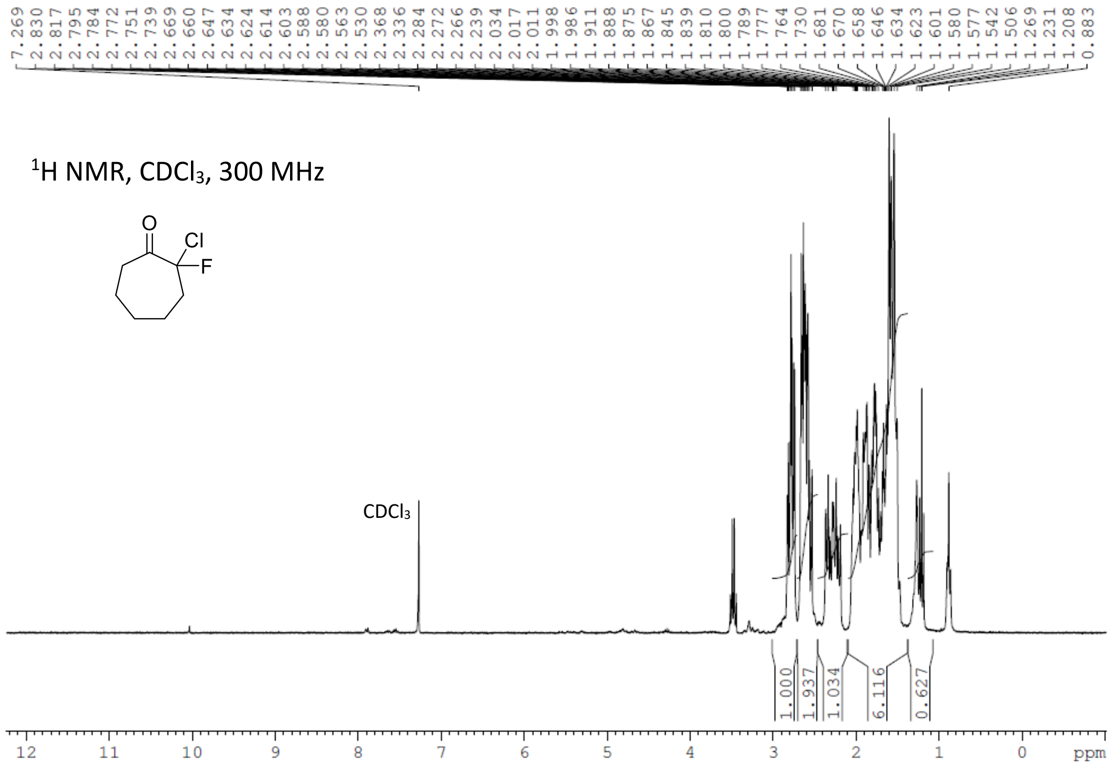

$^{13}C$ NMR, $CDCl_3$, 101 MHz

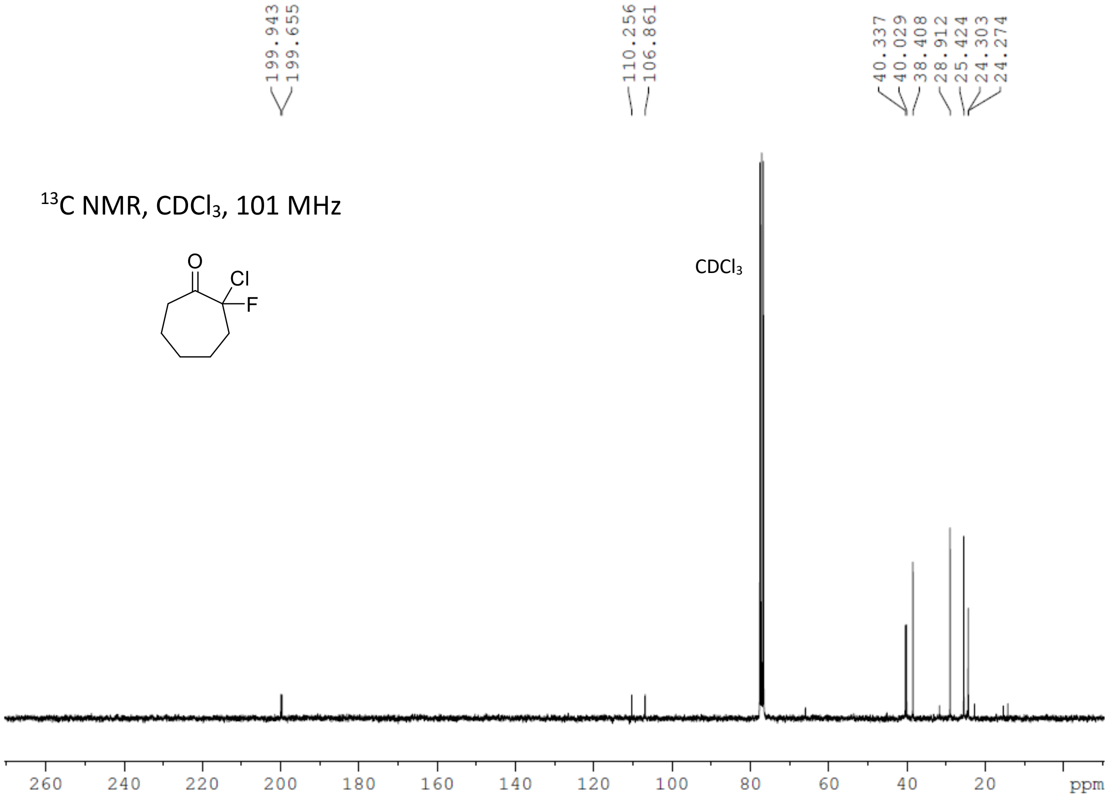


$^{19}F$ NMR, $CDCl_3$, 282 MHz

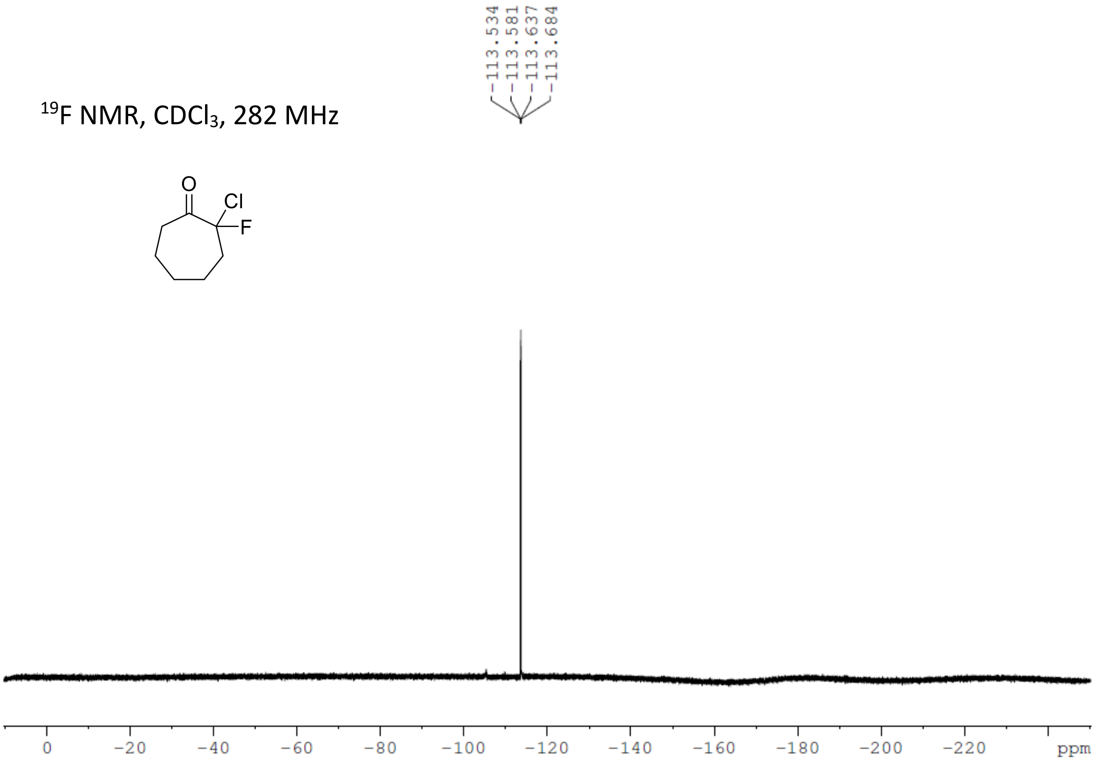

# (*E*/*Z*)-5-Phenylpent-3-en-2-one

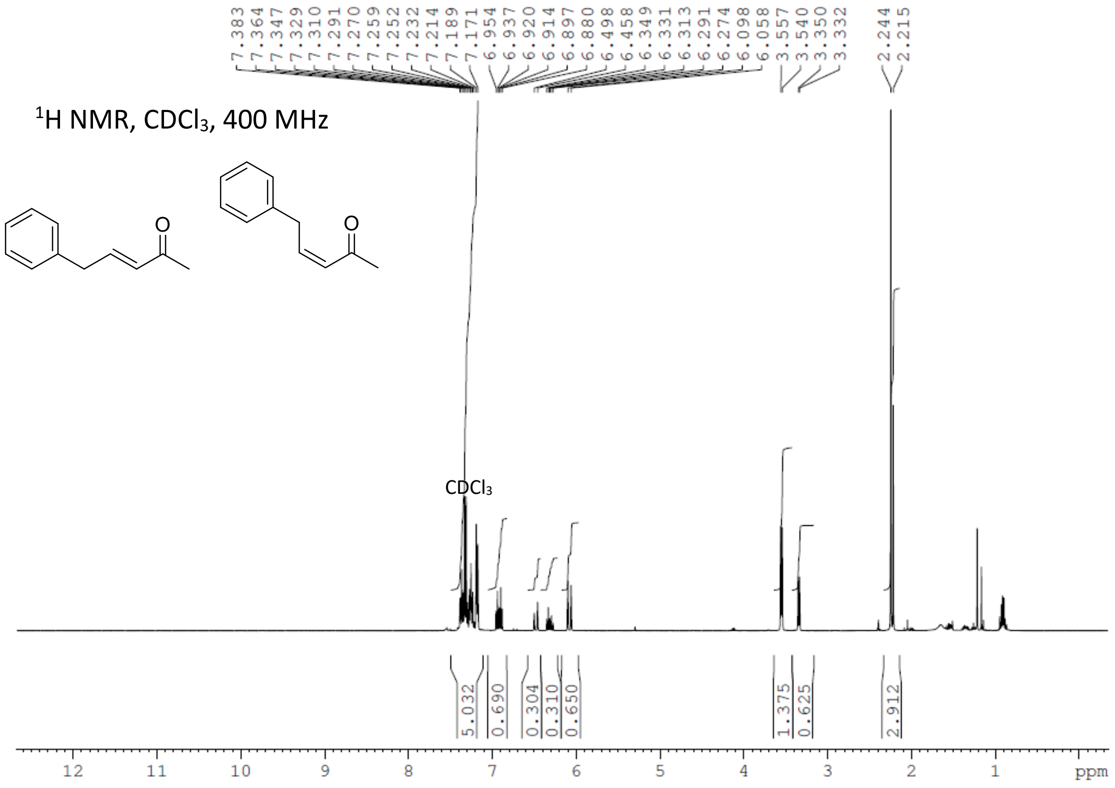


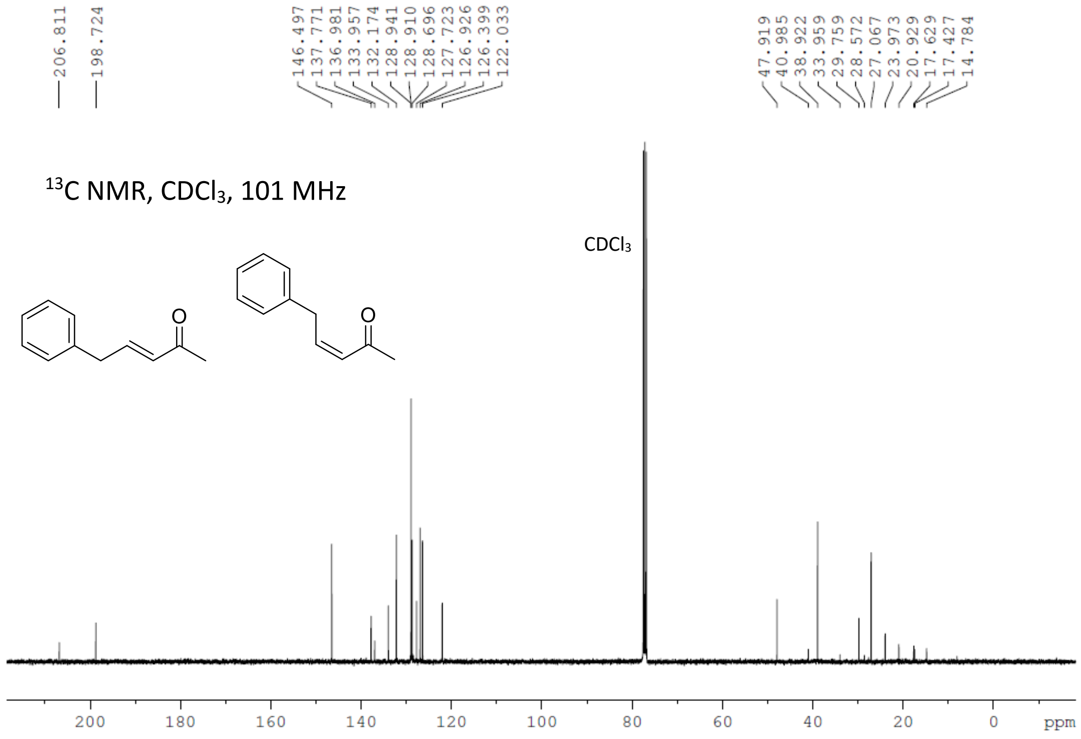

# 5-Phenylpentan-2-one

$^1H$ NMR, $CDCl_3$, 400 MHz

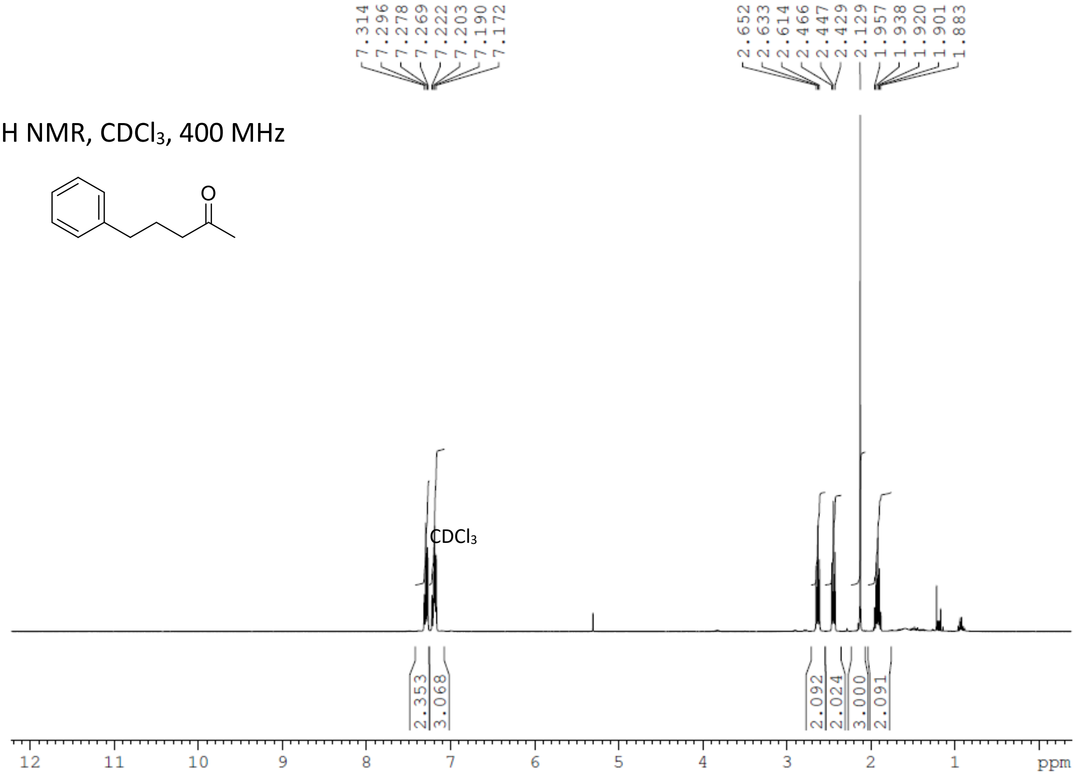


$^{13}C$ NMR, $CDCl_3$, 101 MHz

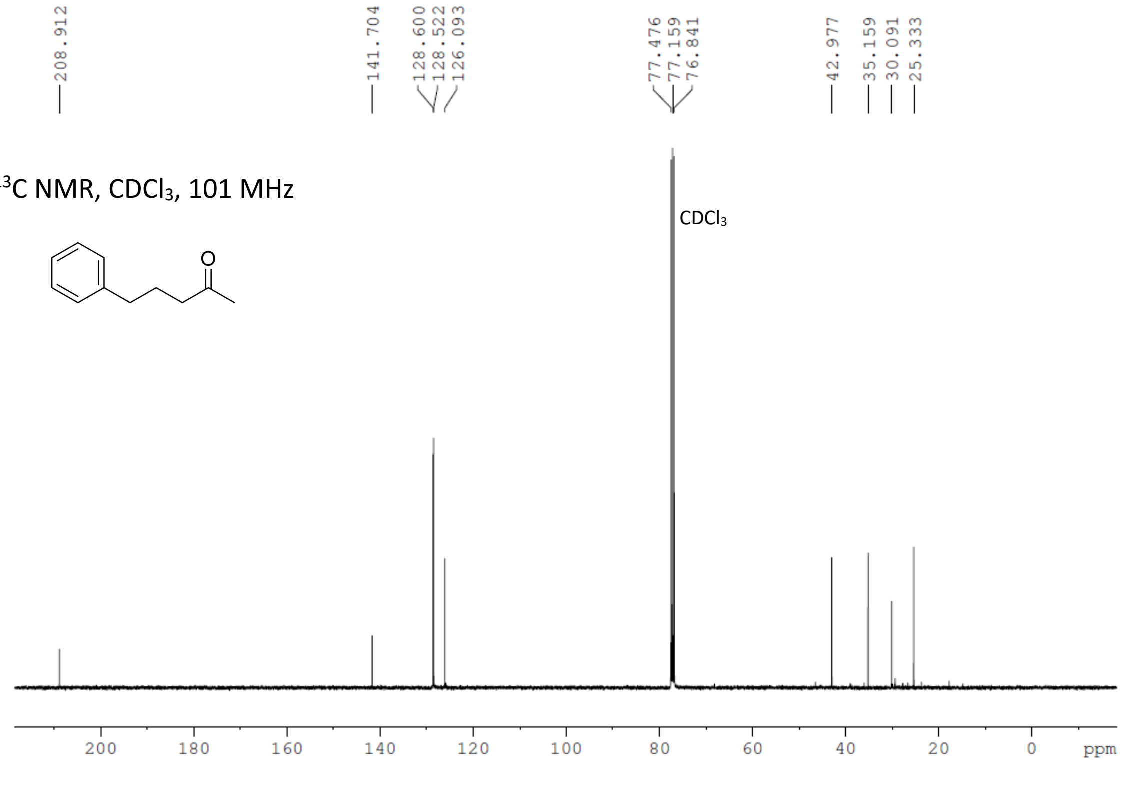

## (*E*)-6-Phenylhex-3-en-2-one

$^{1}H$ NMR, $CDCl_3$, 400 MHz

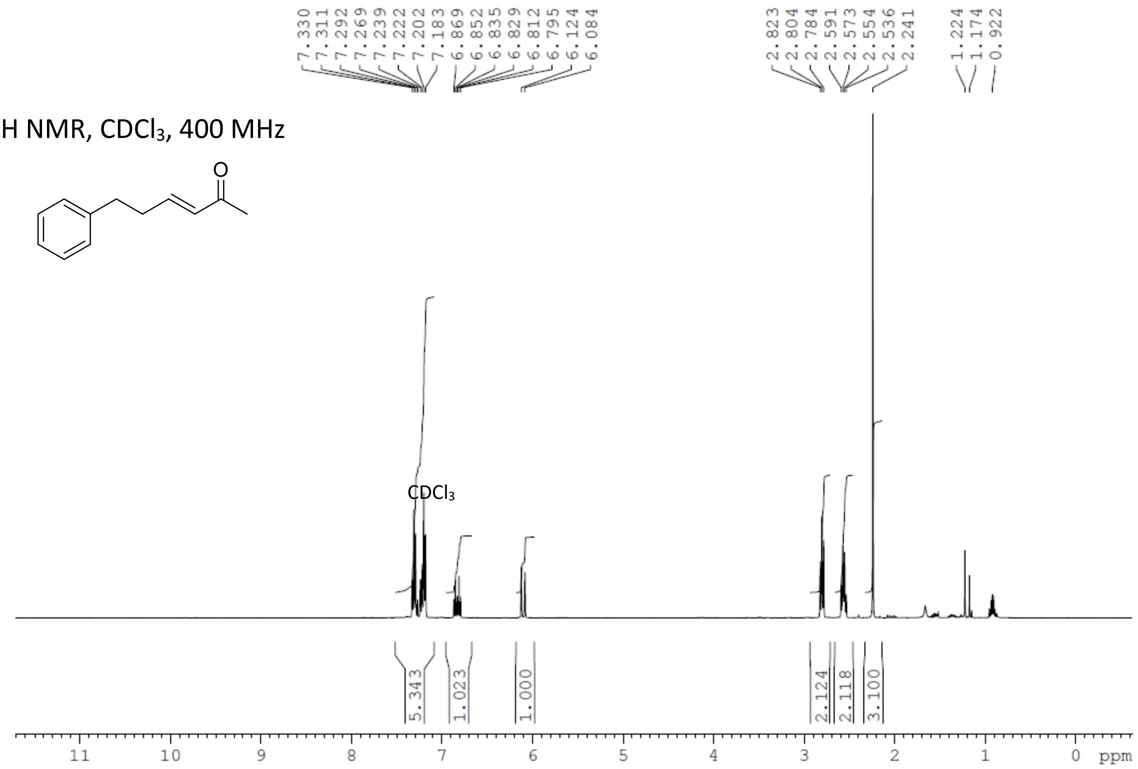


$^{13}C$ NMR, $CDCl_3$, 101 MHz

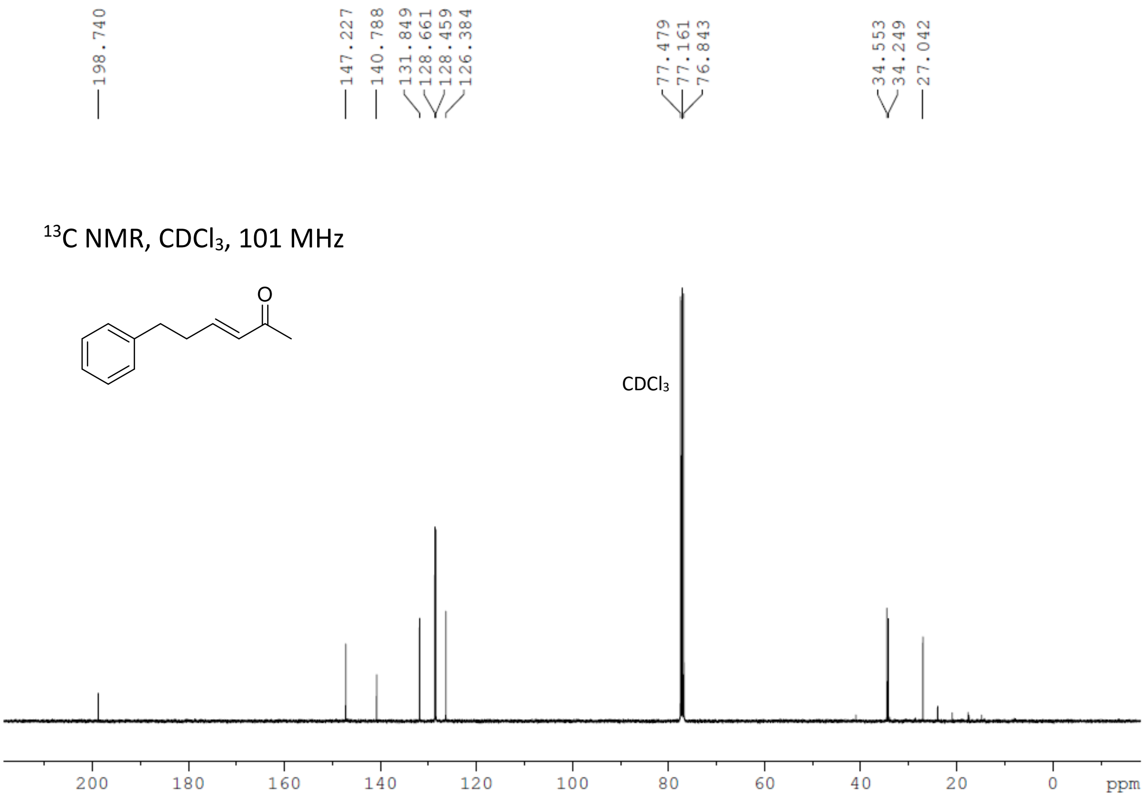

# 6-Phenylhexan-2-one

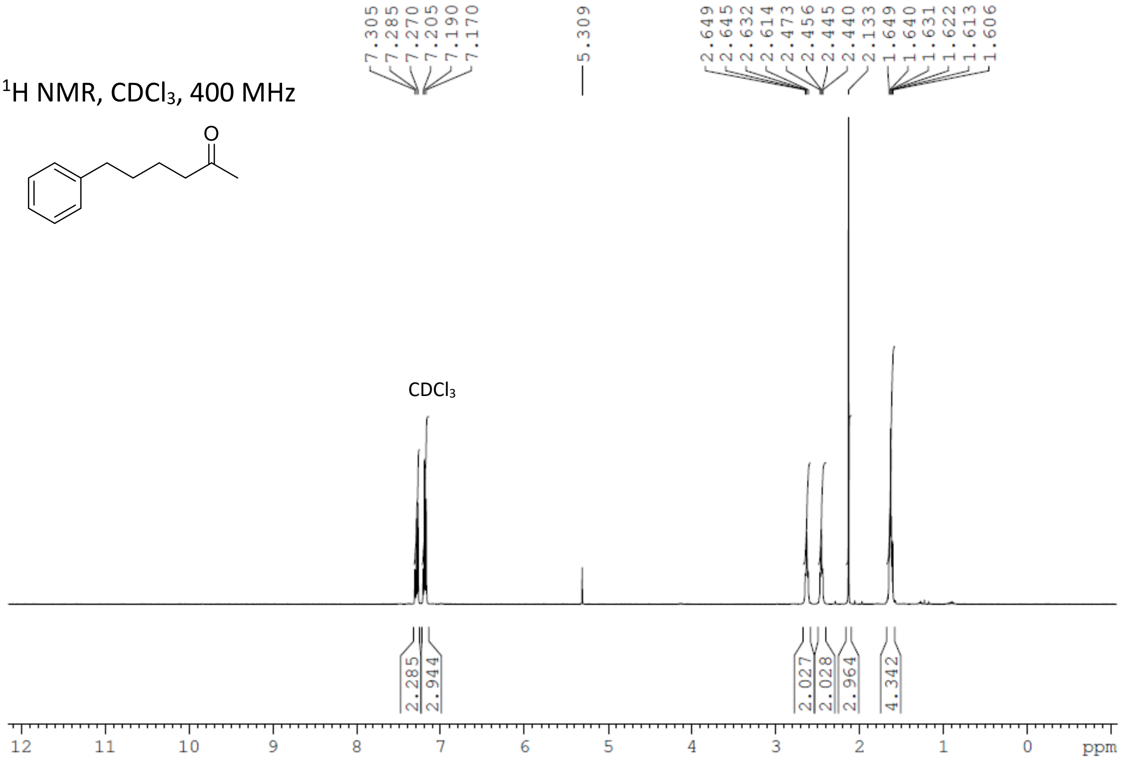


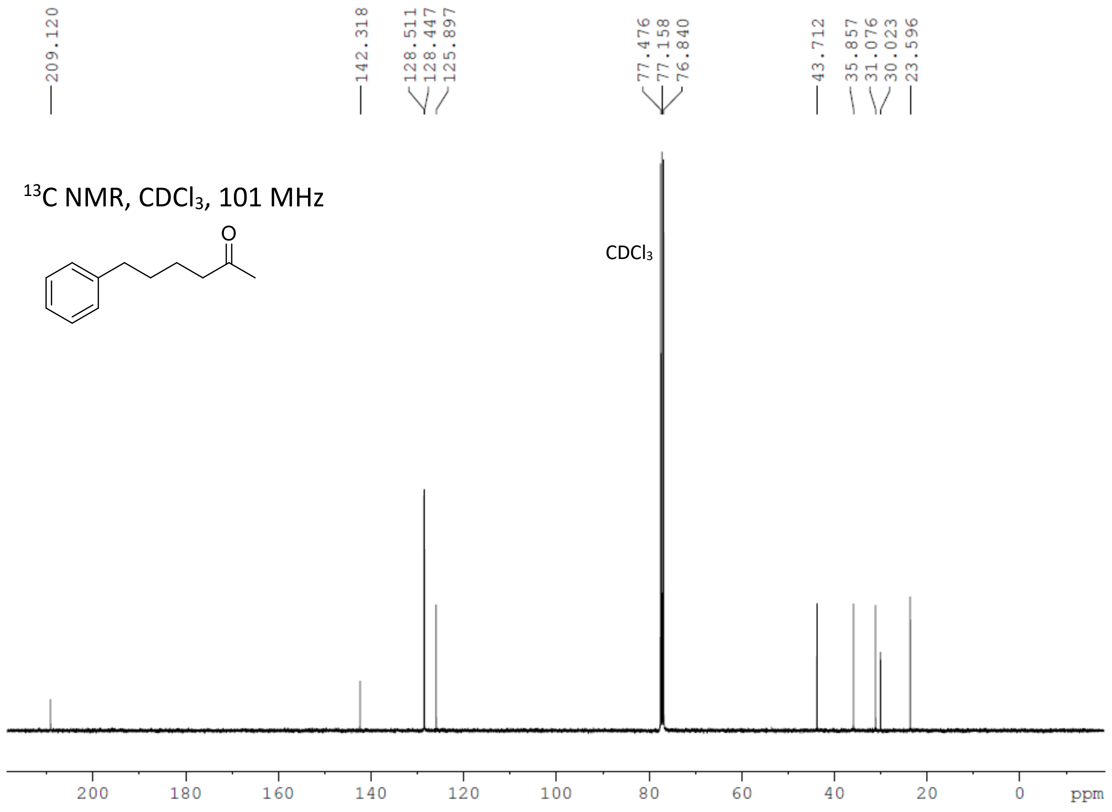

# Allyl 3-oxopentanoate

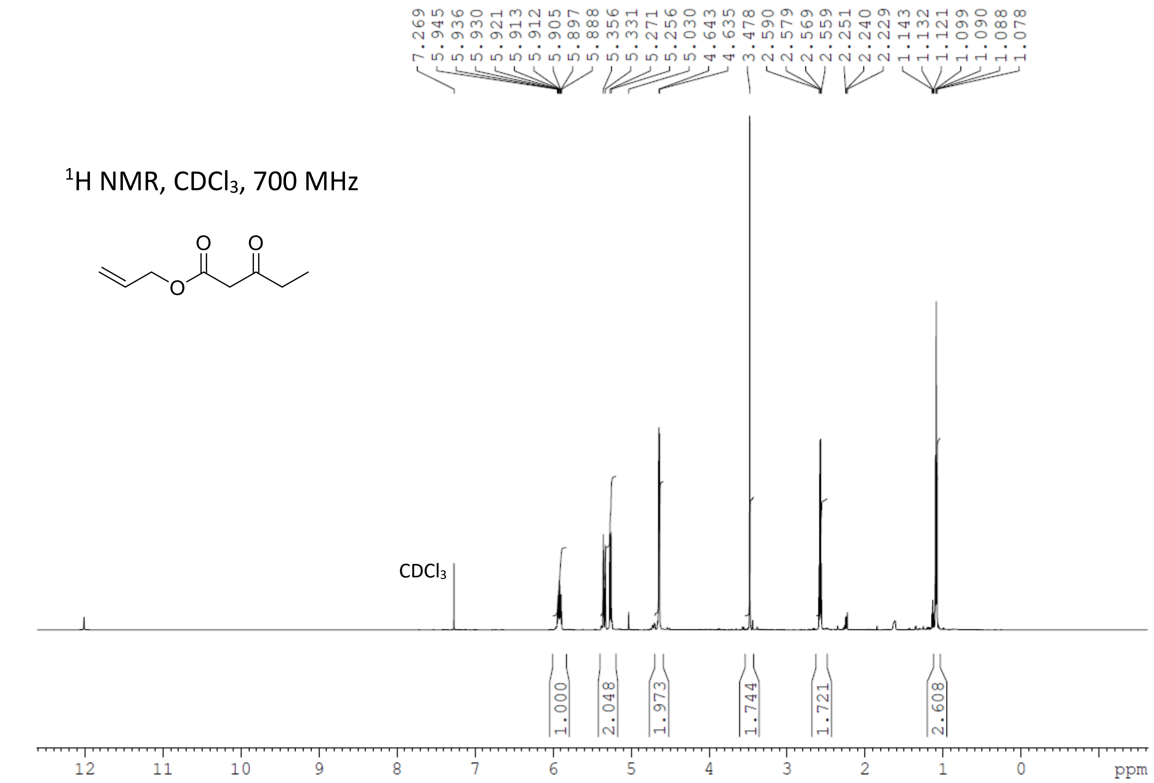


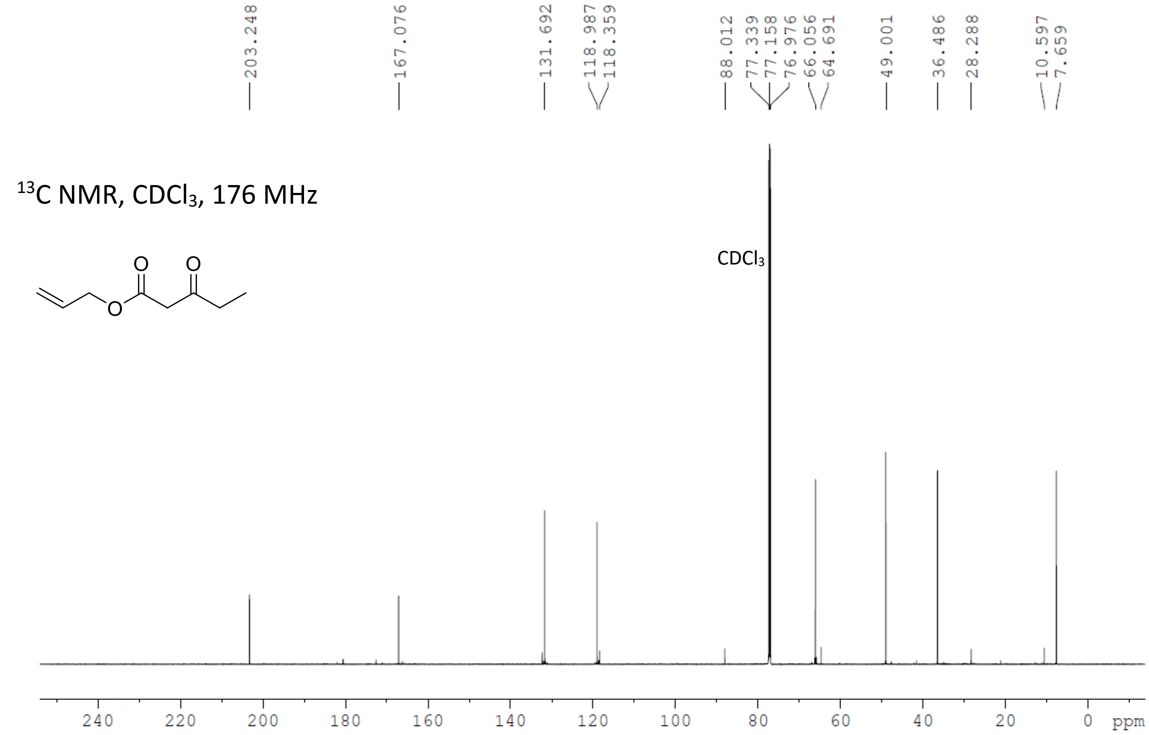

Allyl 3-oxo-2-(4-(trifluoromethyl)benzyl)butanoate (S1-7b)

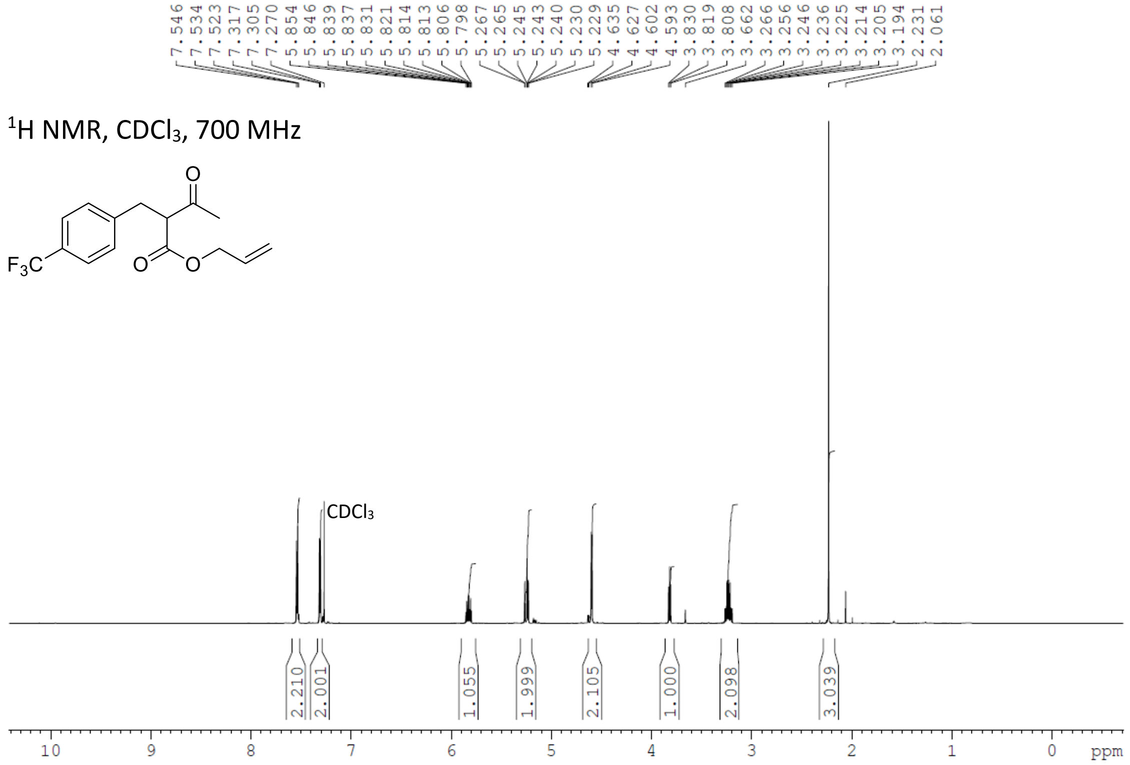


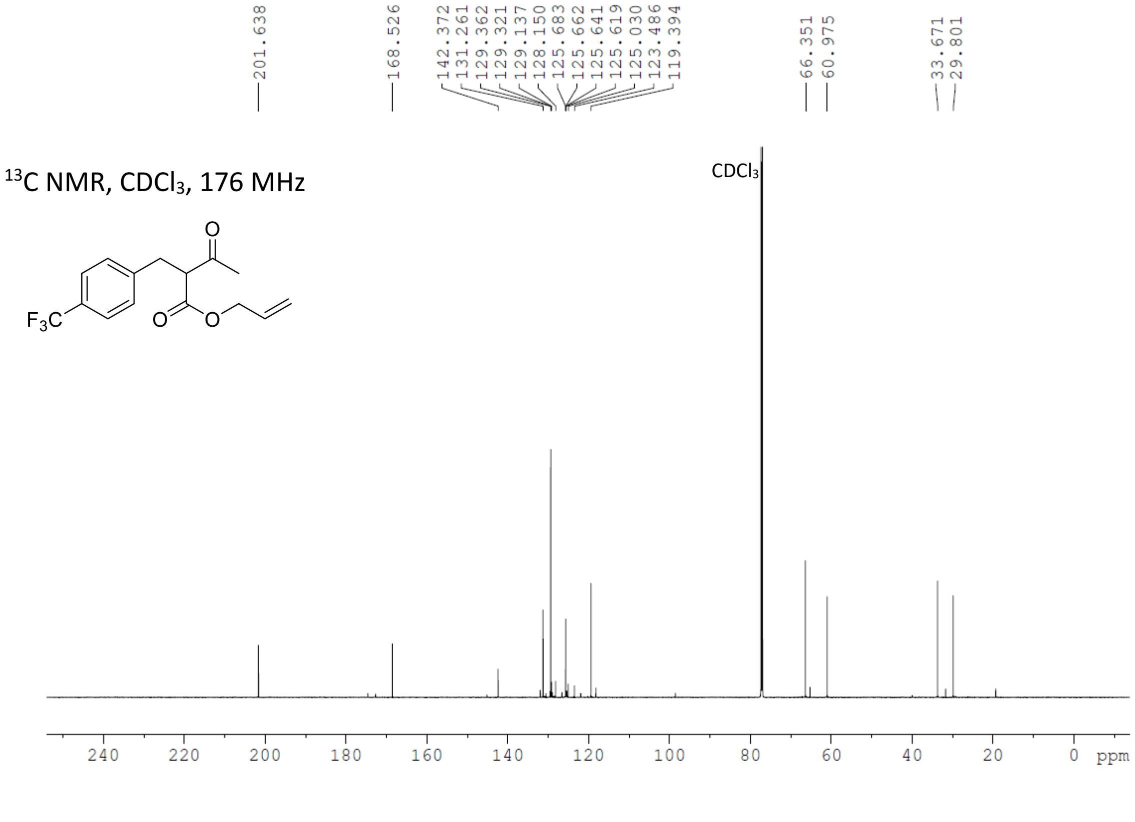

## Allyl 3-oxo-2-(4-methoxybenzyl)butanoate (S1-7c)

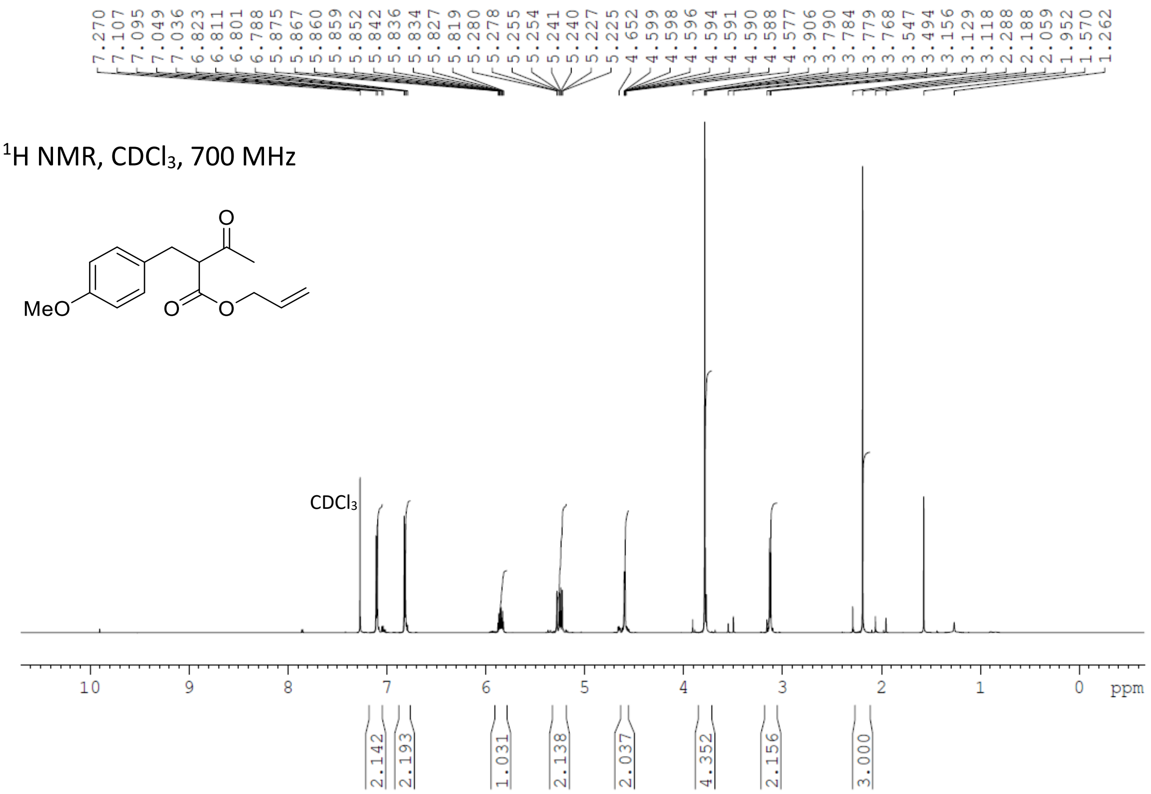


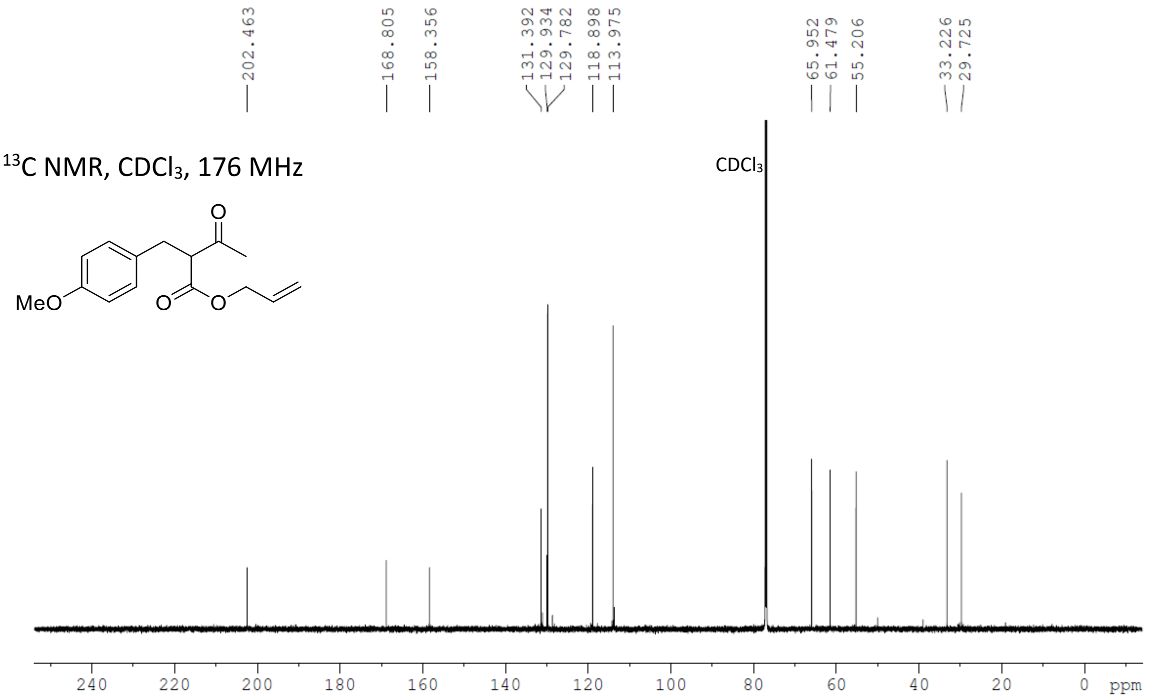

## Allyl 3-oxo-2-(4-cyanobenzyl)butanoate (S1-7d)

$^{1}$H NMR, $CDCl_3$, 700 MHz

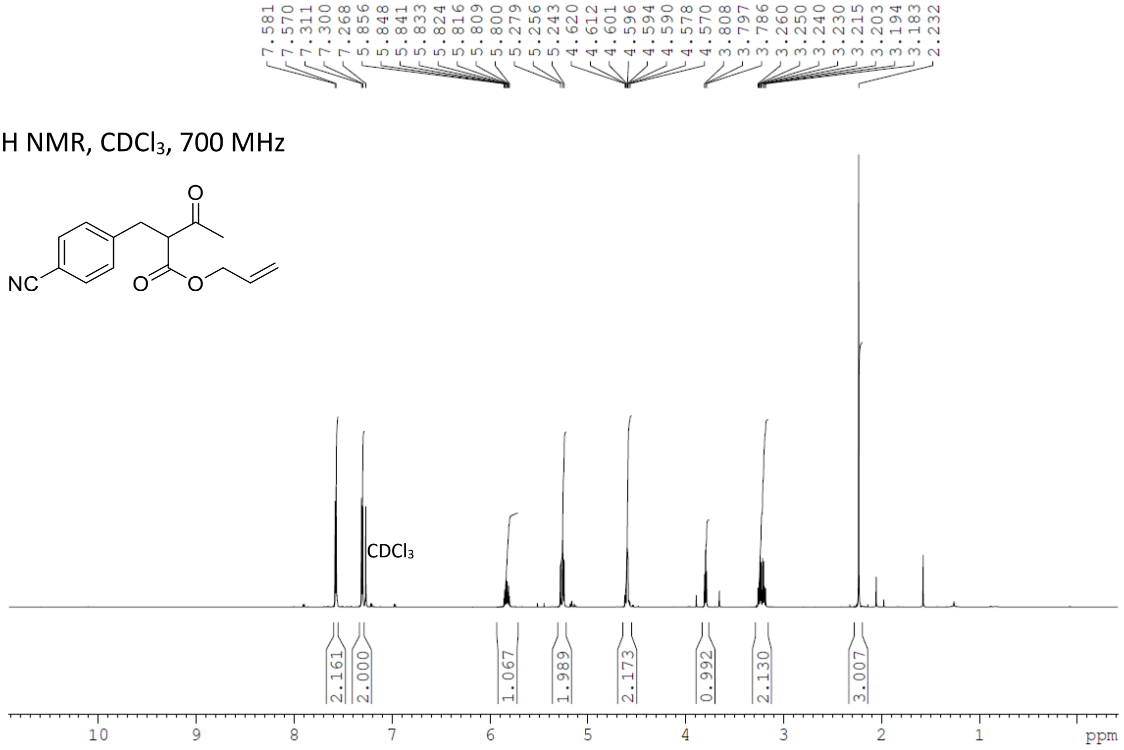


$^{13}$C NMR, $CDCl_3$, 176 MHz

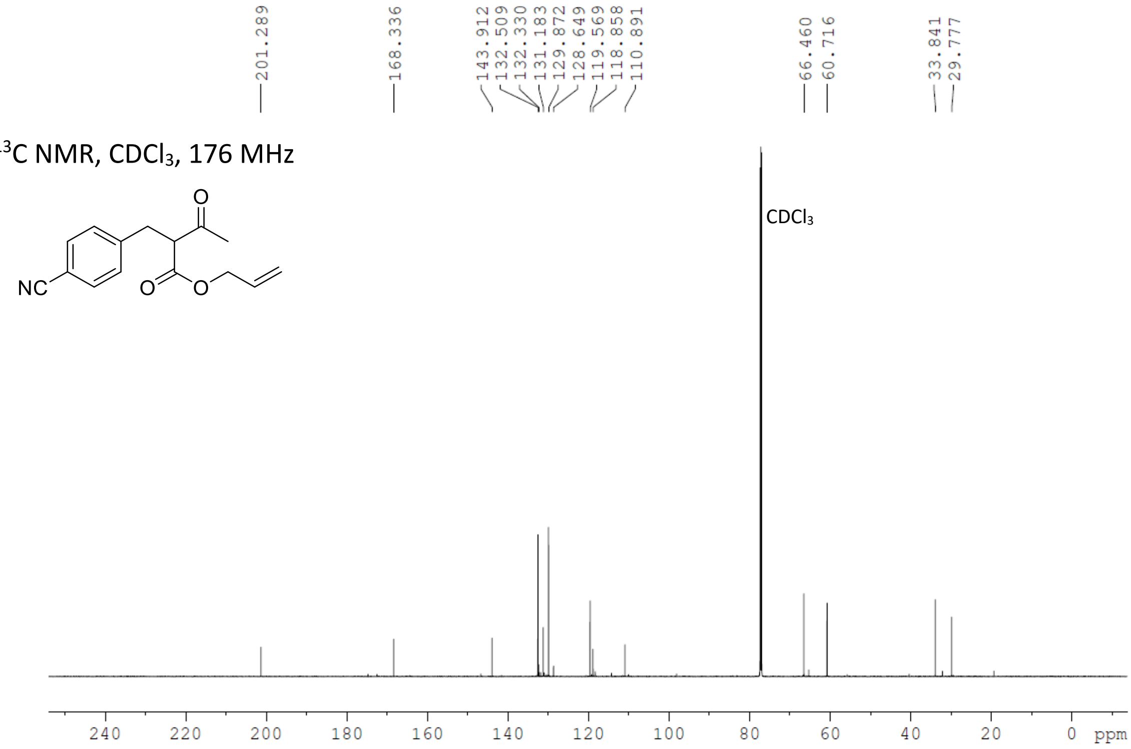

## Allyl 3-oxo-2-(3-(trifluoromethyl)benzyl)butanoate (S1-7f)

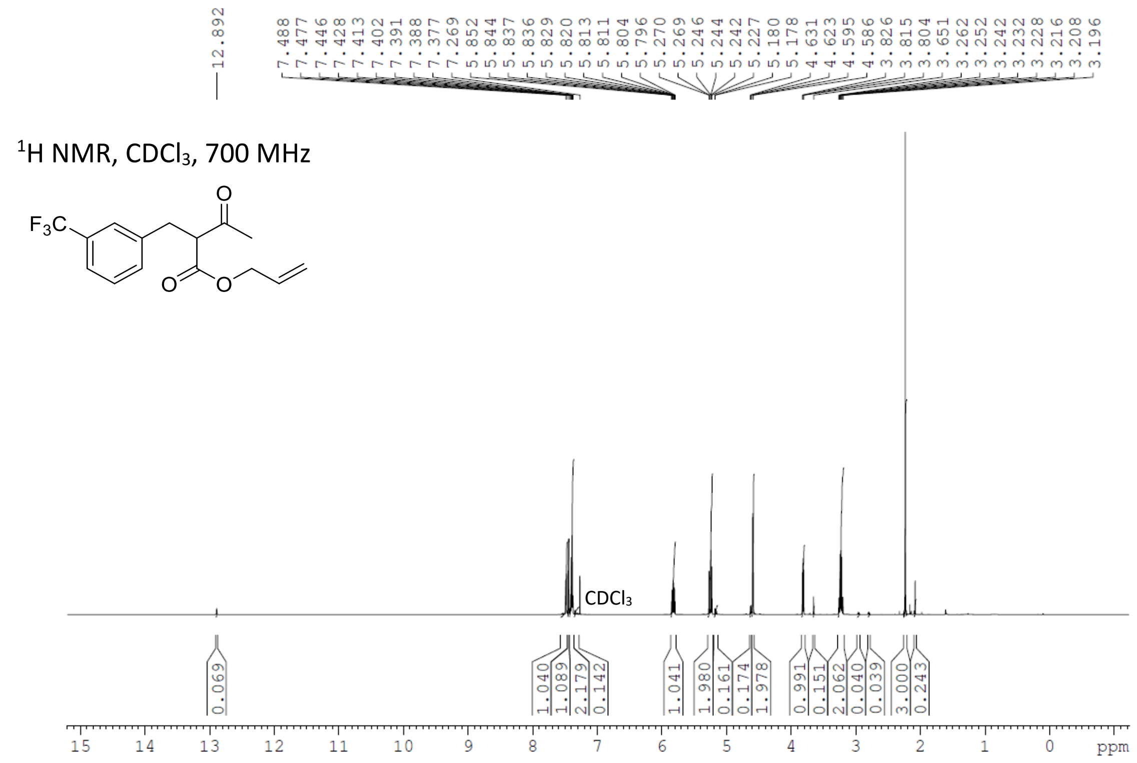


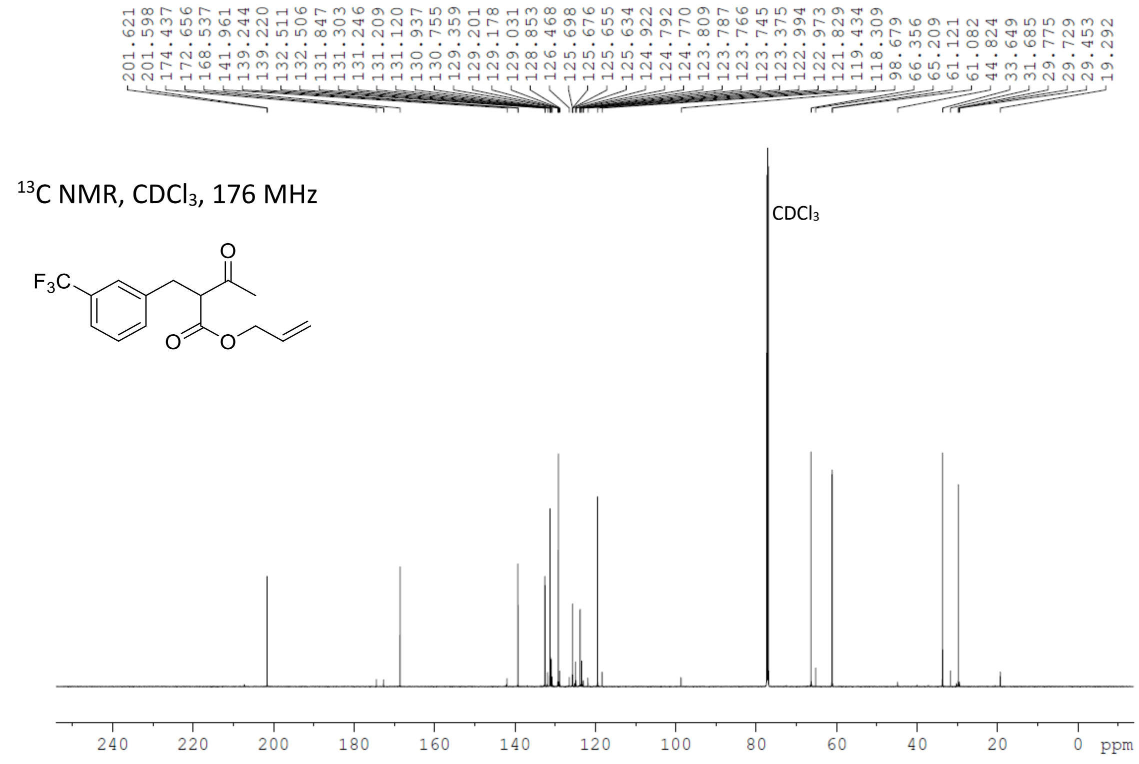

## Allyl 2-benzyl-3-oxopentanoate (S1-11a)

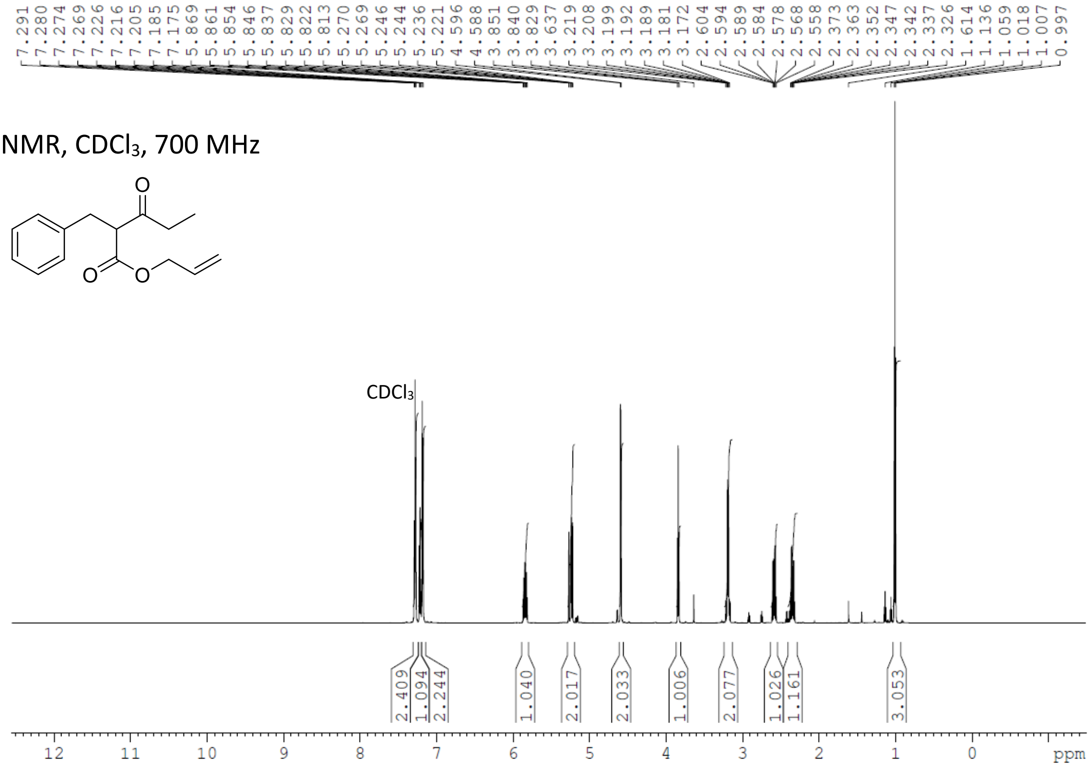


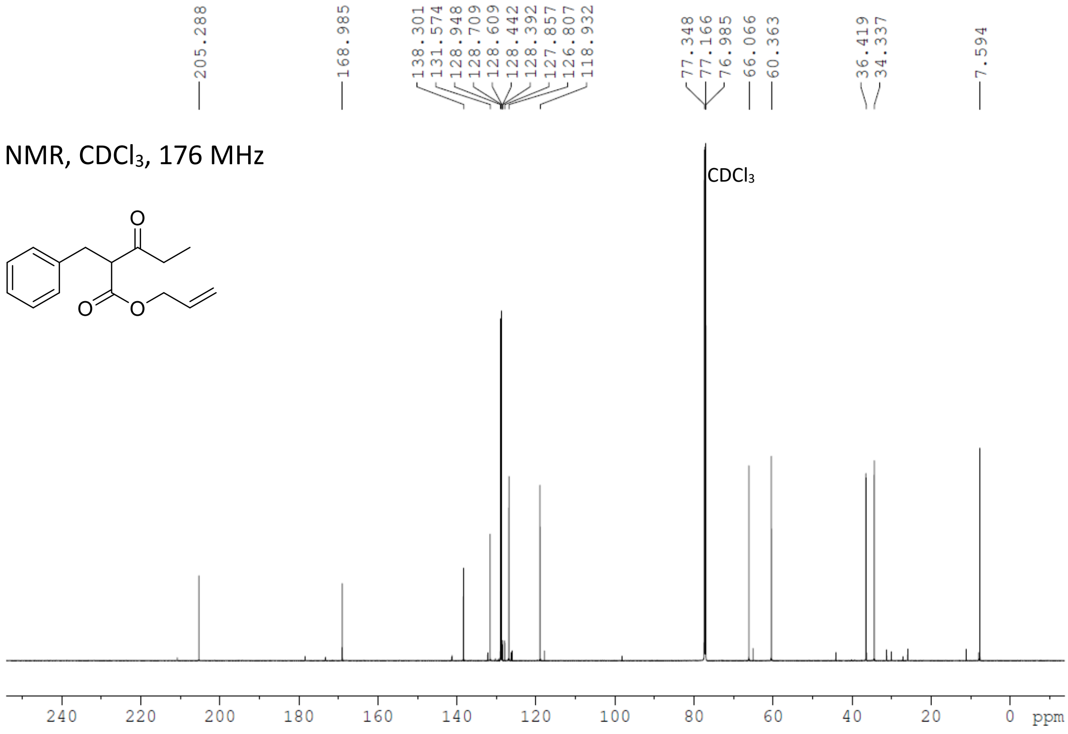

## Allyl 2-fluoro-3-oxo-2-(4-(trifluoromethyl)benzyl)butanoate (S2-7b)

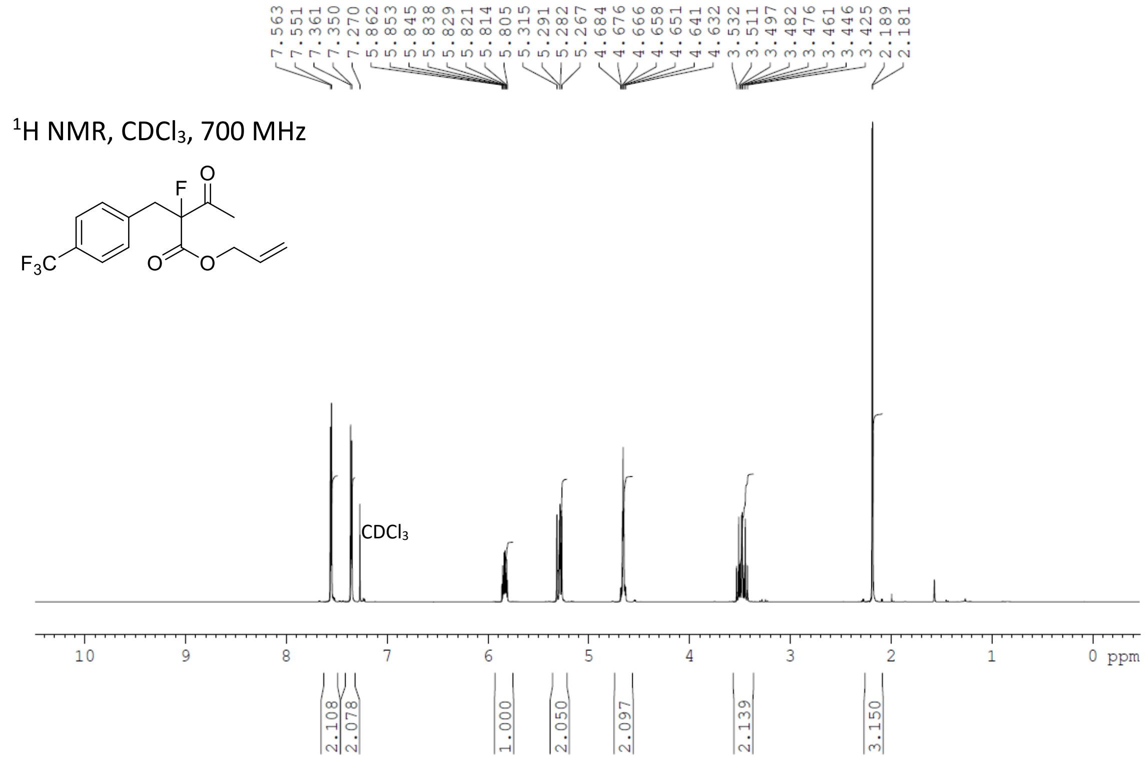


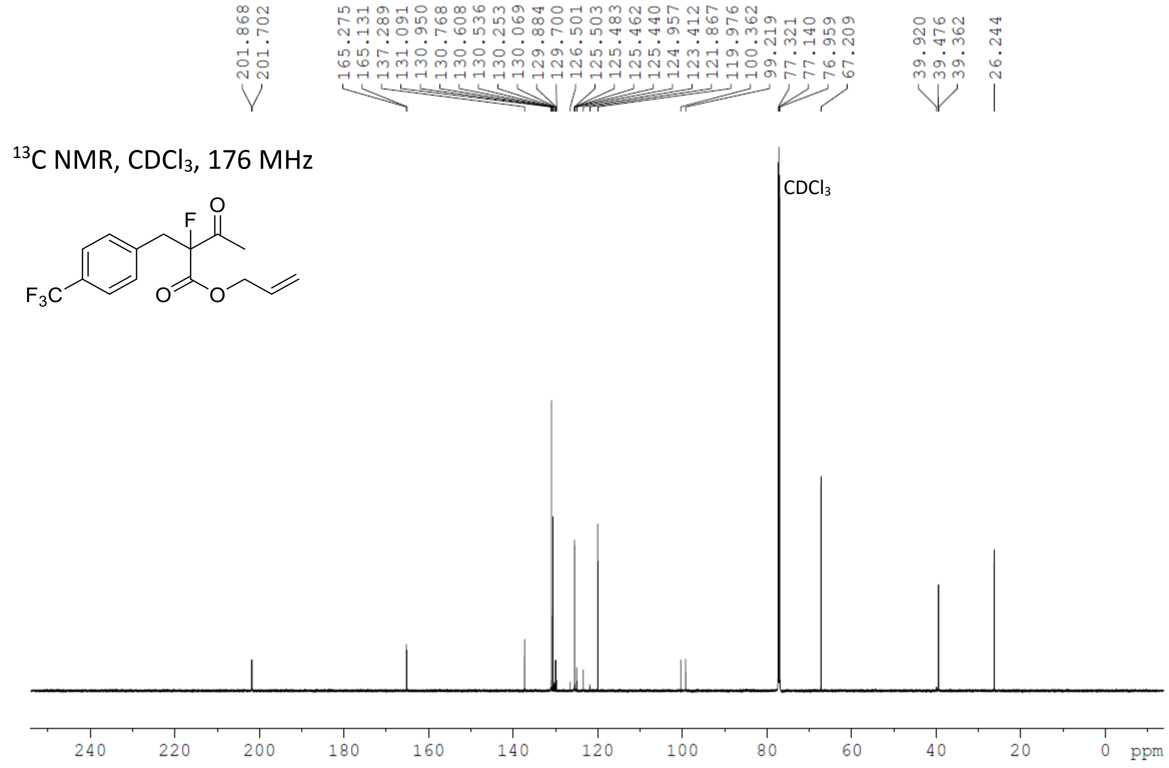

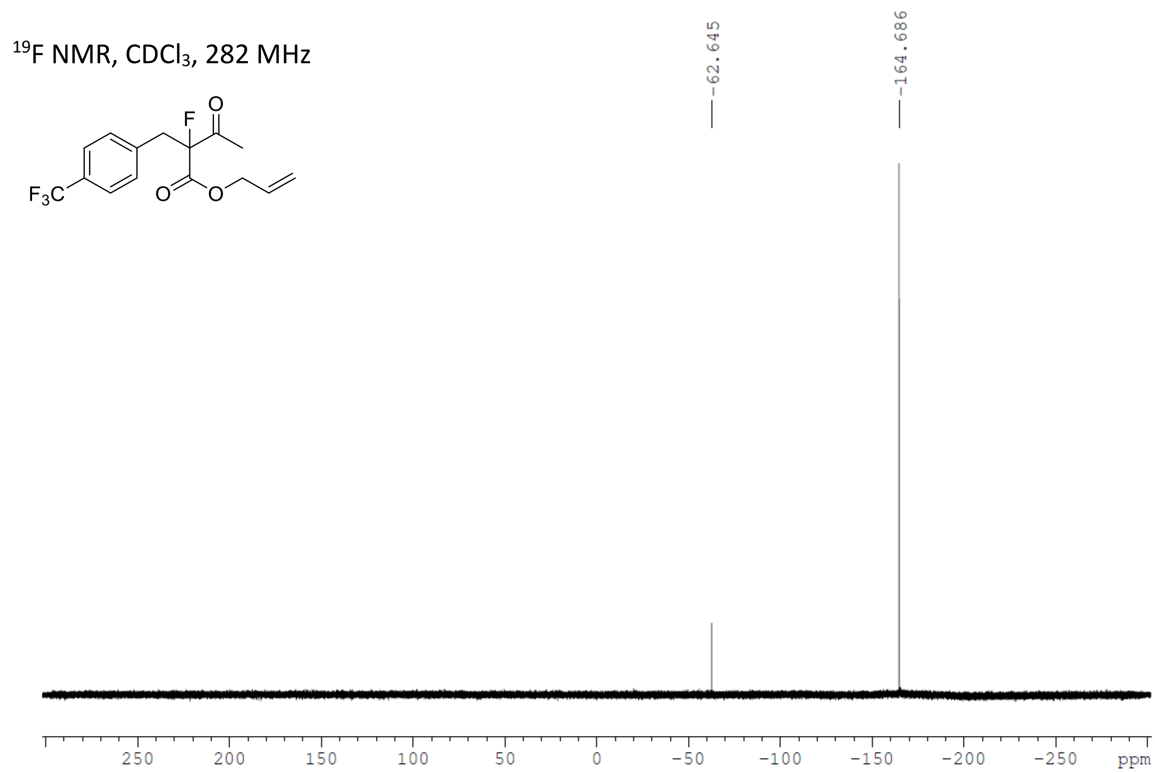


## Allyl 2-fluoro-3-oxo-2-(4-methoxybenzyl)butanoate (S2-7c)

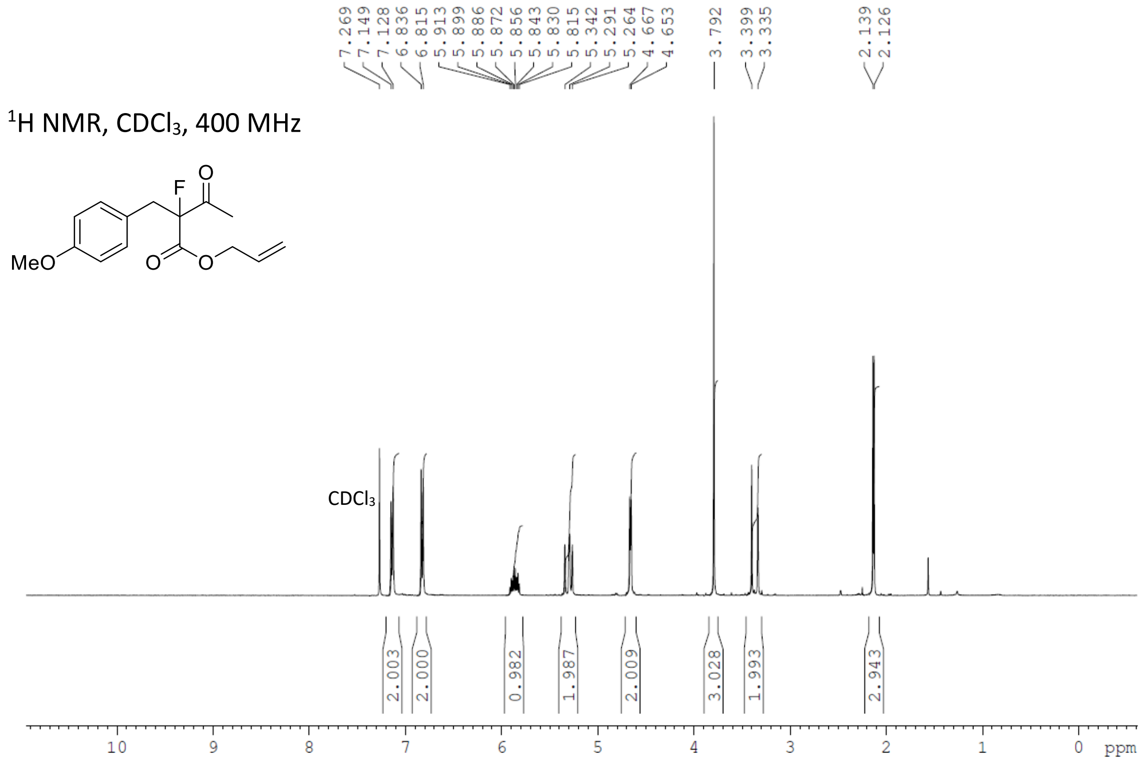

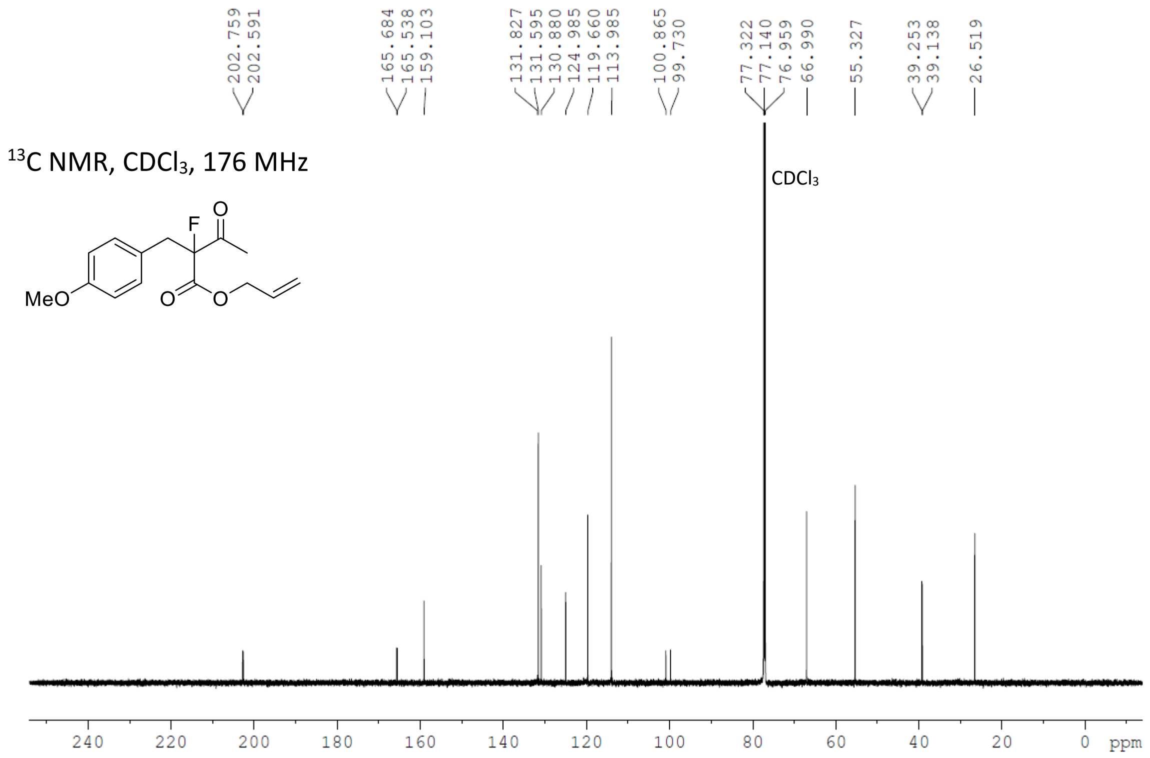
202.759
202.591
165.684
165.538
159.103
131.827
131.595
130.880
124.985
119.660
113.985
100.865
99.730
77.322
77.140
76.959
66.990
55.327
39.253
39.138
26.519
13C NMR, CDCl3, 176 MHz
CDCl3
F
O
MeO
O
O
240
220
200
180
160
140
120
100
80
60
40
20
0
ppm

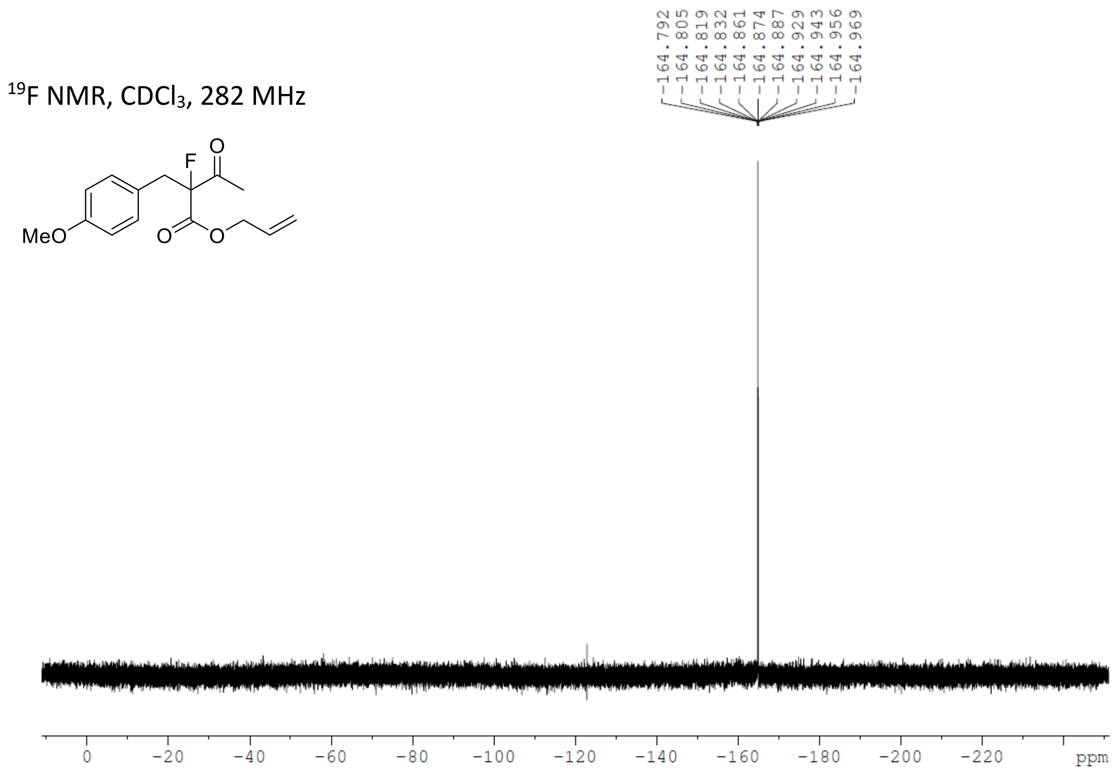
-164.792
-164.805
-164.819
-164.832
-164.861
-164.874
-164.887
-164.929
-164.943
-164.956
-164.969
19F NMR, CDCl3, 282 MHz
F
O
MeO
O
O
0
-20
-40
-60
-80
-100
-120
-140
-160
-180
-200
-220
ppm

## Allyl 2-fluoro-3-oxo-2-(4-cyanobenzyl)butanoate (S2-7d)

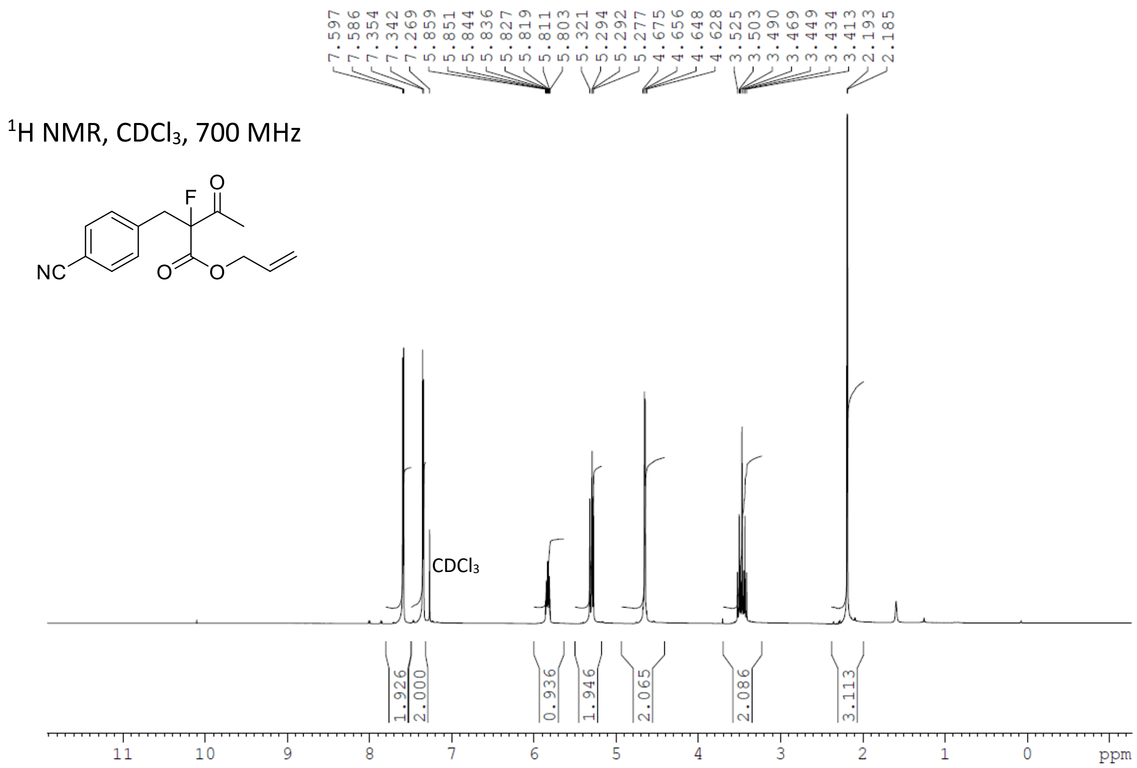


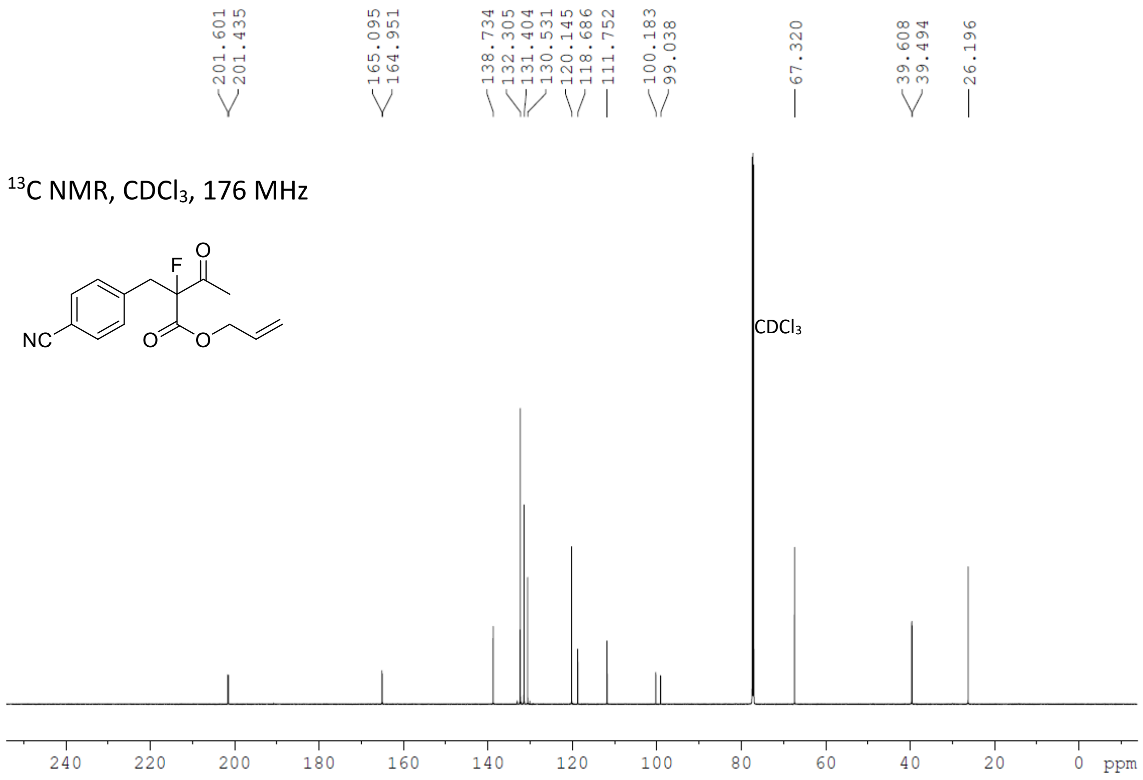

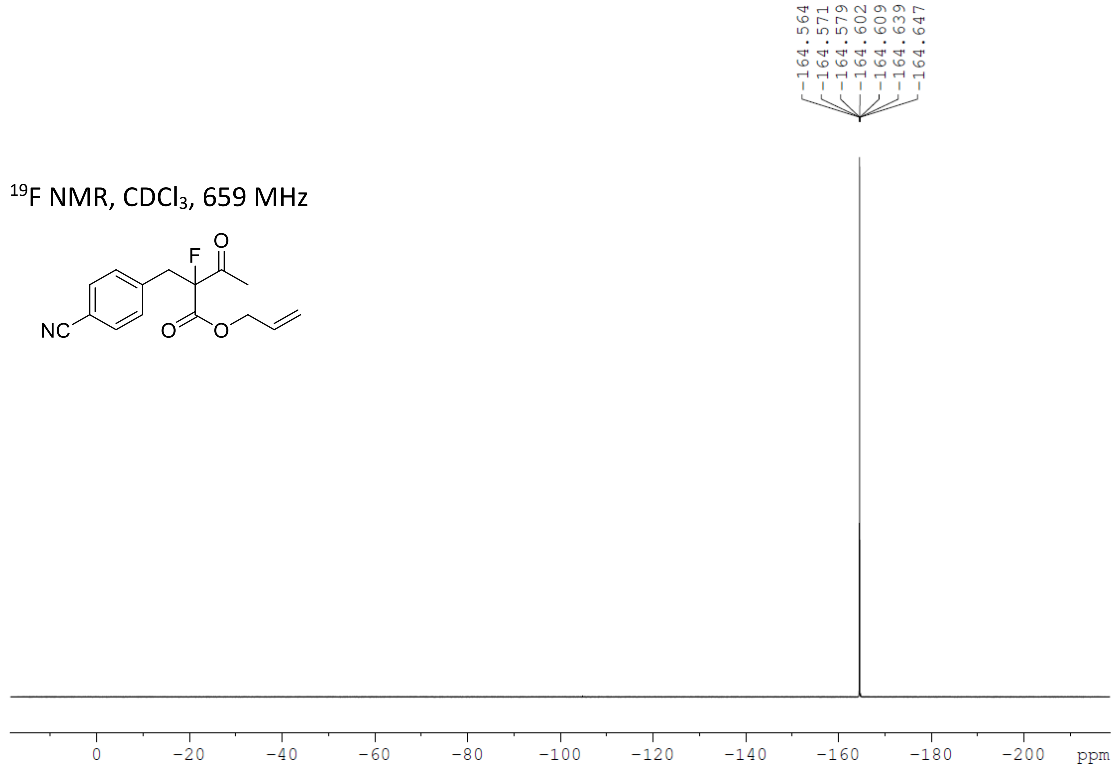


# Allyl 2-fluoro-3-oxo-2-(3-(trifluoromethyl)benzyl)butanoate (S2-7f)

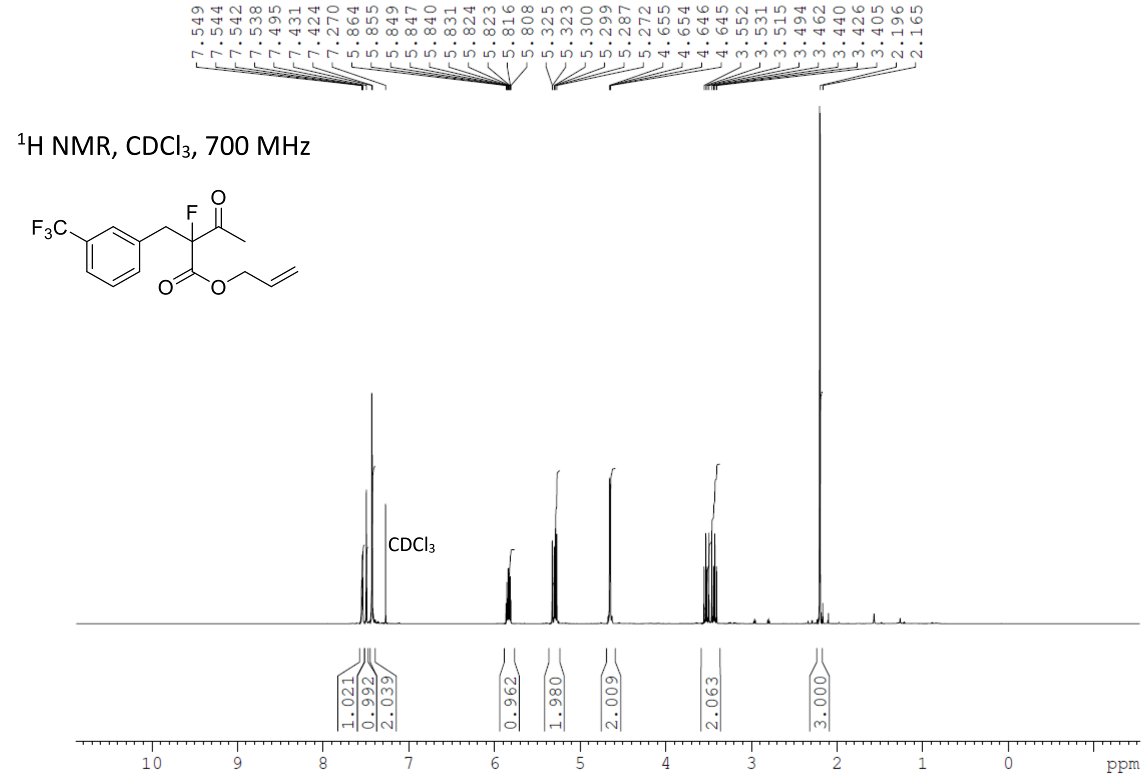

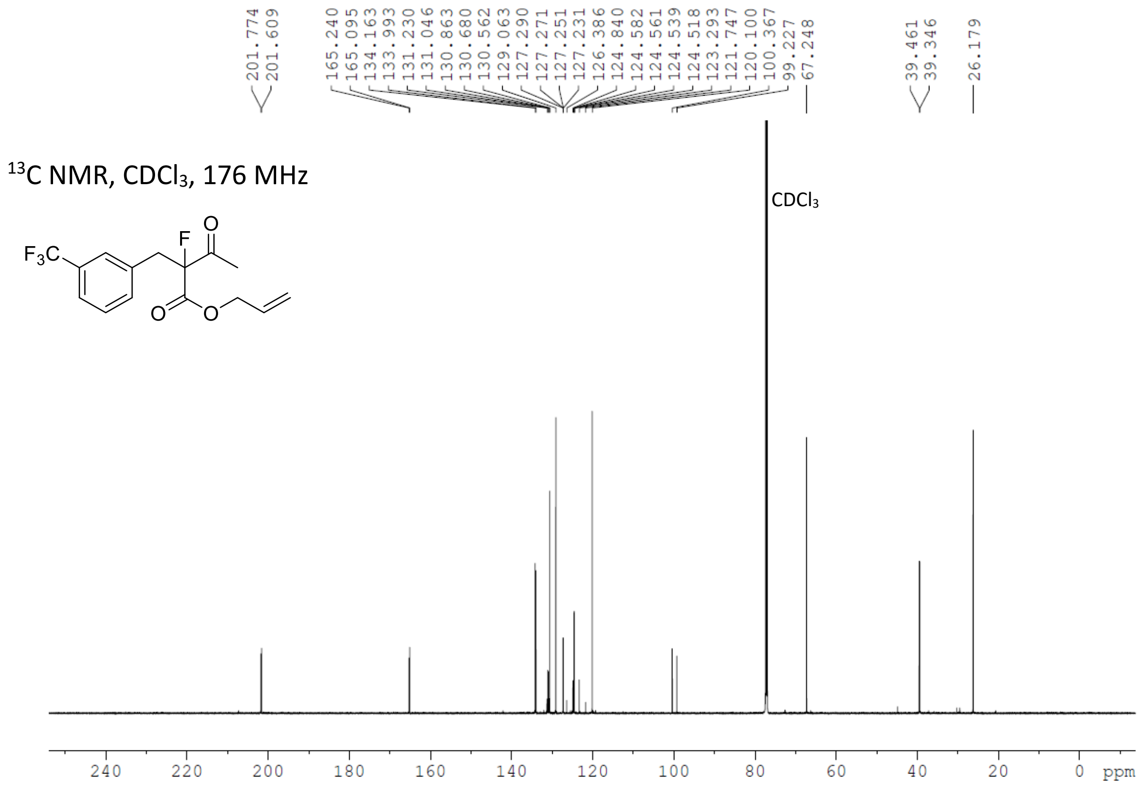
13C NMR, CDCl3, 176 MHz
CDCl3
F3C
F
O
O
O
201.774
201.609
165.240
165.095
134.163
133.993
131.230
131.046
130.863
130.680
130.562
129.063
127.290
127.271
127.251
127.231
126.386
124.840
124.582
124.561
124.539
124.518
123.293
121.747
120.100
100.367
99.227
67.248
39.461
39.346
26.179
240
220
200
180
160
140
120
100
80
60
40
20
0
ppm

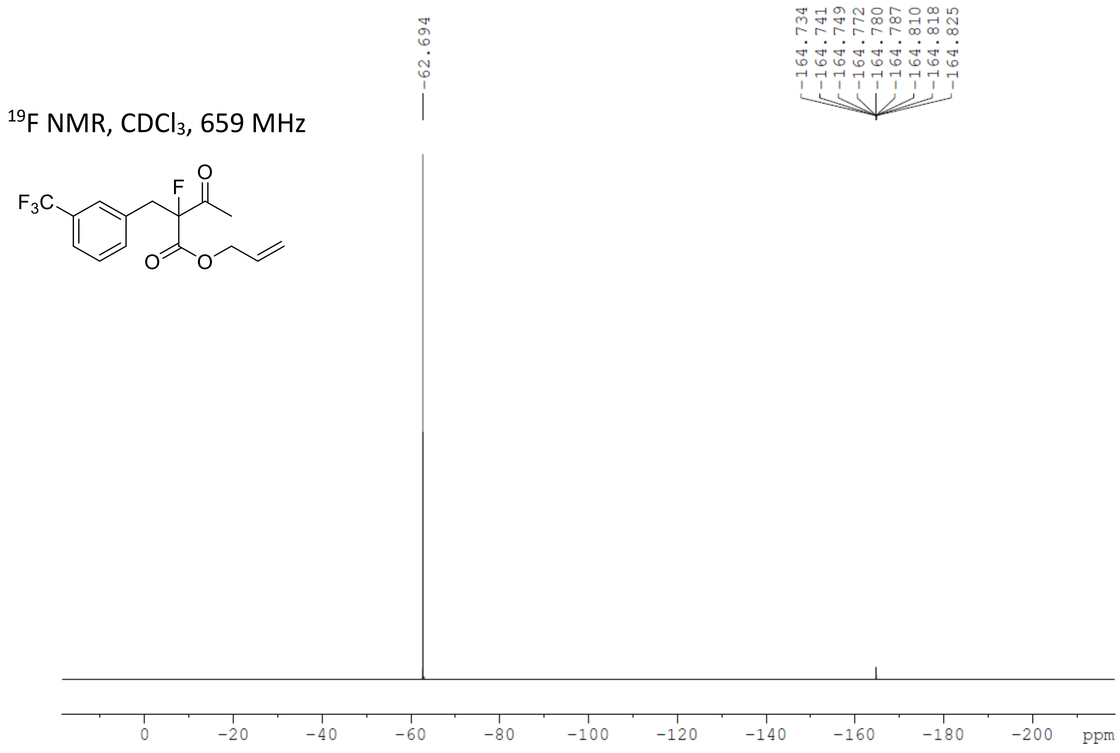
19F NMR, CDCl3, 659 MHz
F3C
F
O
O
O
-62.694
-164.734
-164.741
-164.749
-164.772
-164.780
-164.787
-164.810
-164.818
-164.825
0
-20
-40
-60
-80
-100
-120
-140
-160
-180
-200
ppm

Allyl 2-benzyl-2-fluoro-3-oxopentanoate (S2-11a)

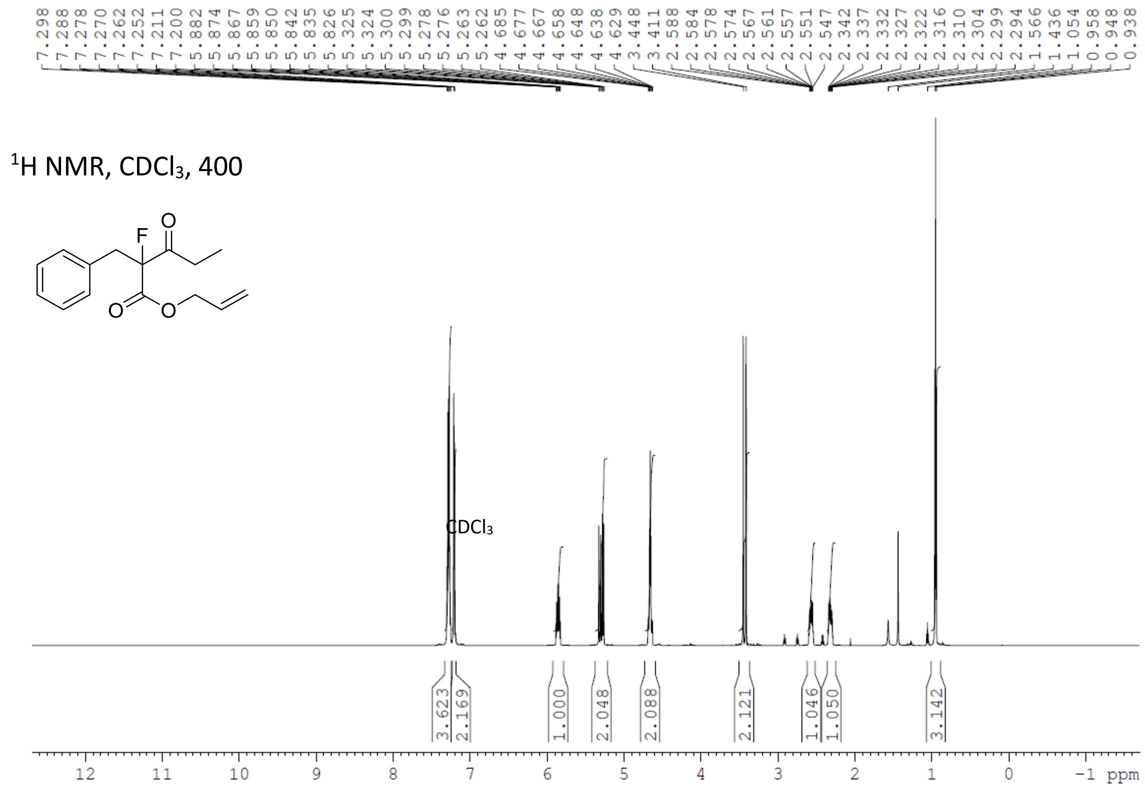


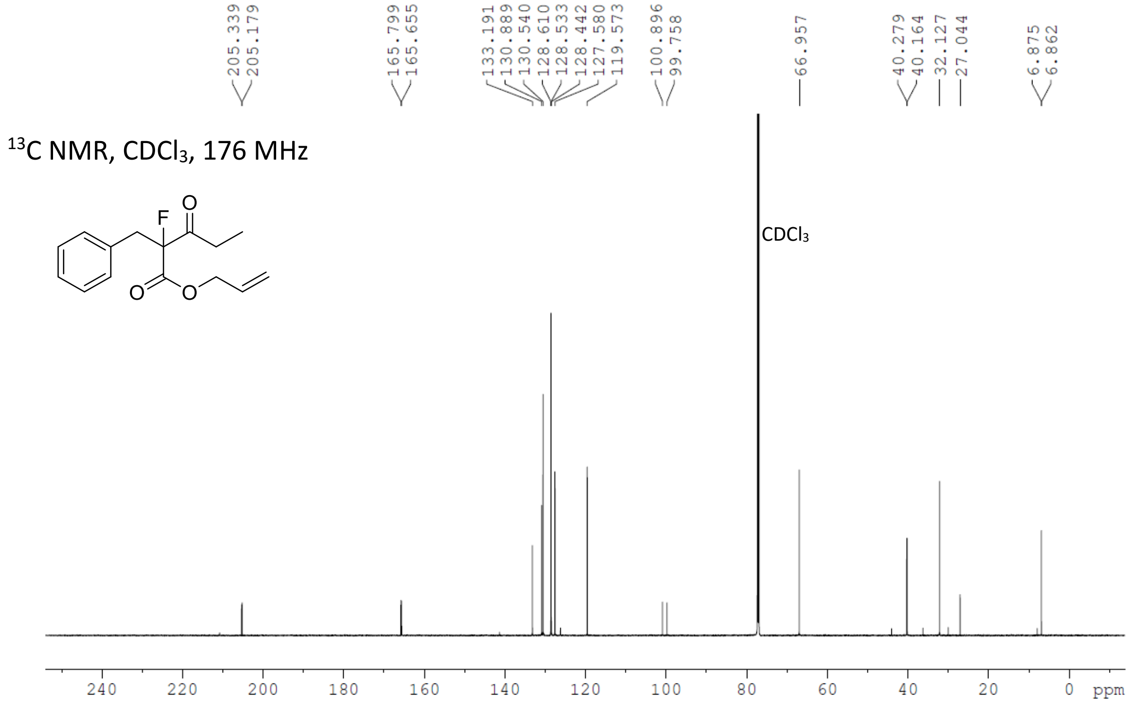

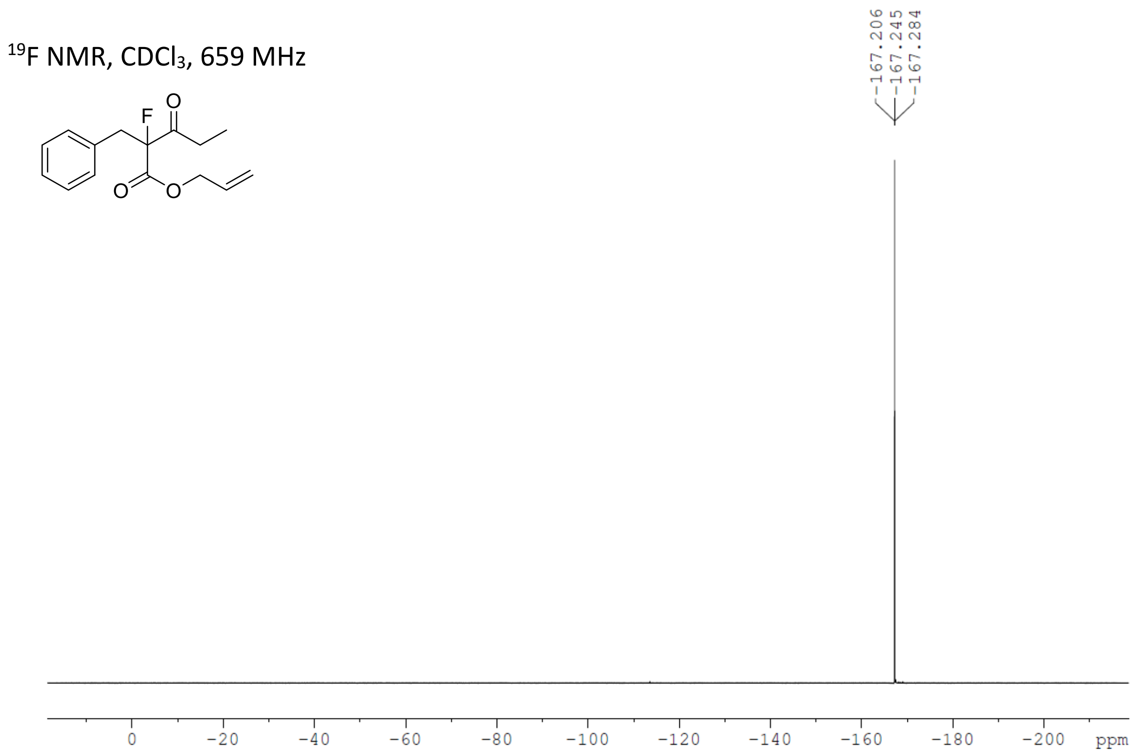


## Ethyl (*Z*)-2-fluoro-3-phenylacrylate ((*Z*)-S3-6a)

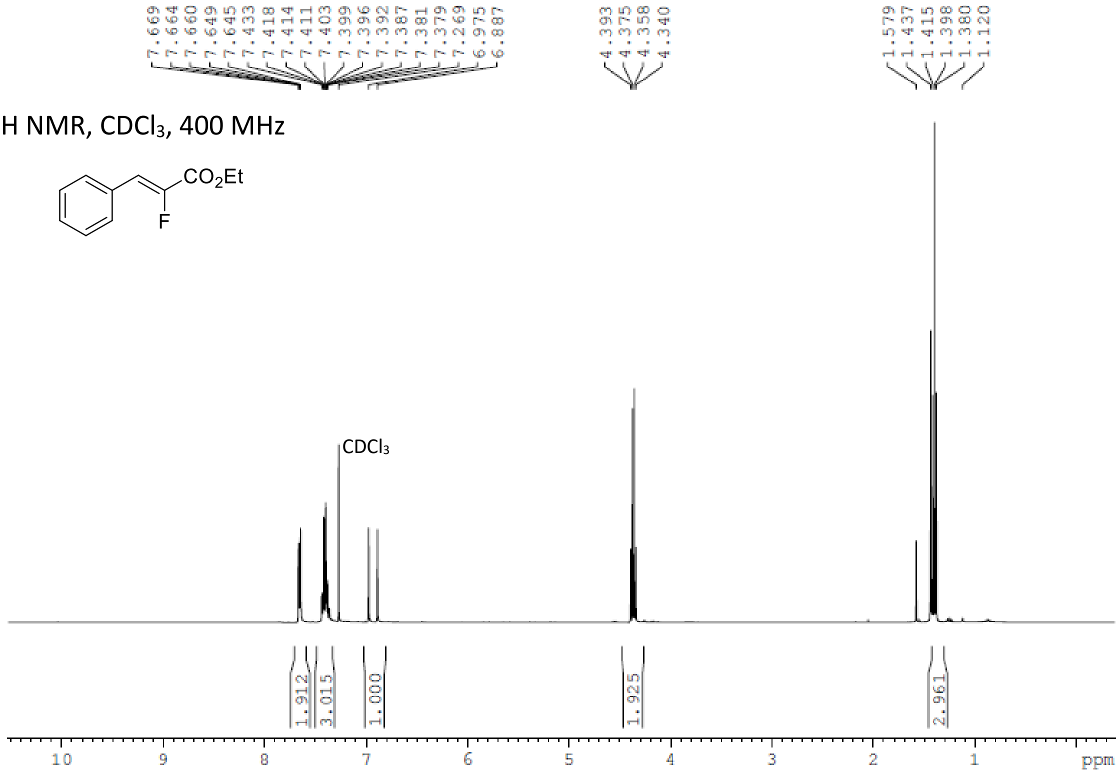

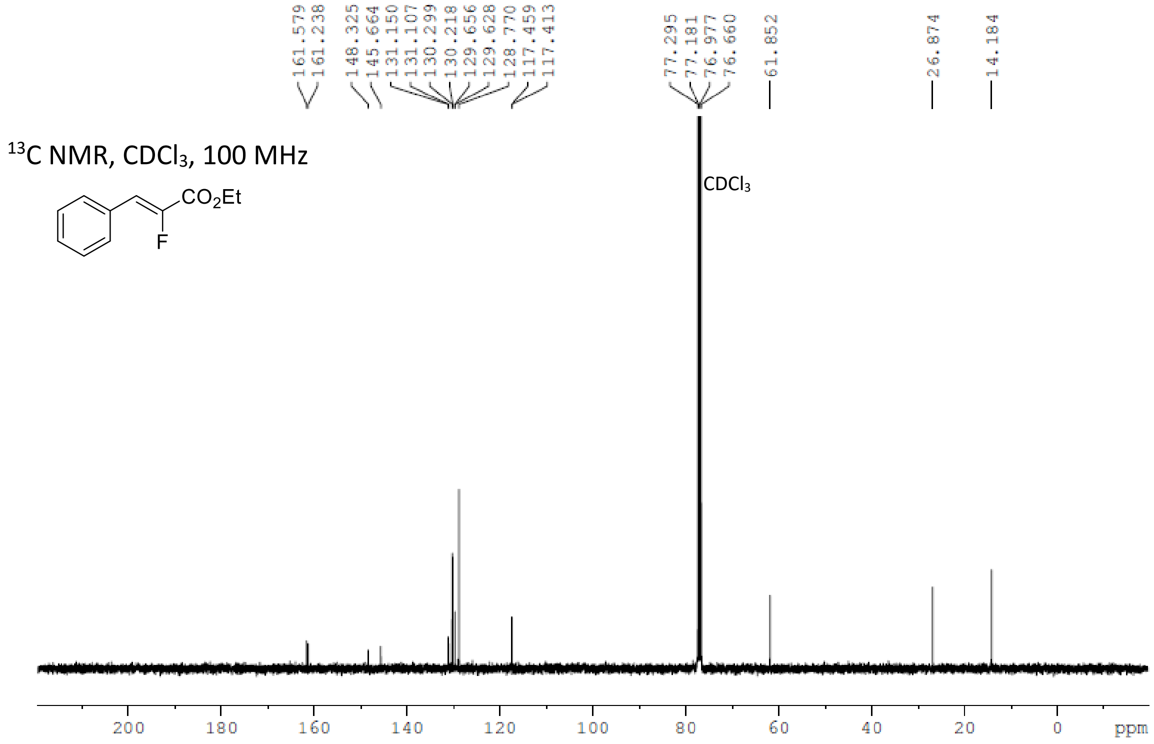
161.579
161.238
148.325
145.664
131.150
131.107
130.299
130.218
129.656
129.628
128.770
117.459
117.413
77.295
77.181
76.977
76.660
61.852
26.874
14.184
13C NMR, CDCl3, 100 MHz
CO2Et
F
CDCl3
200
180
160
140
120
100
80
60
40
20
0
ppm

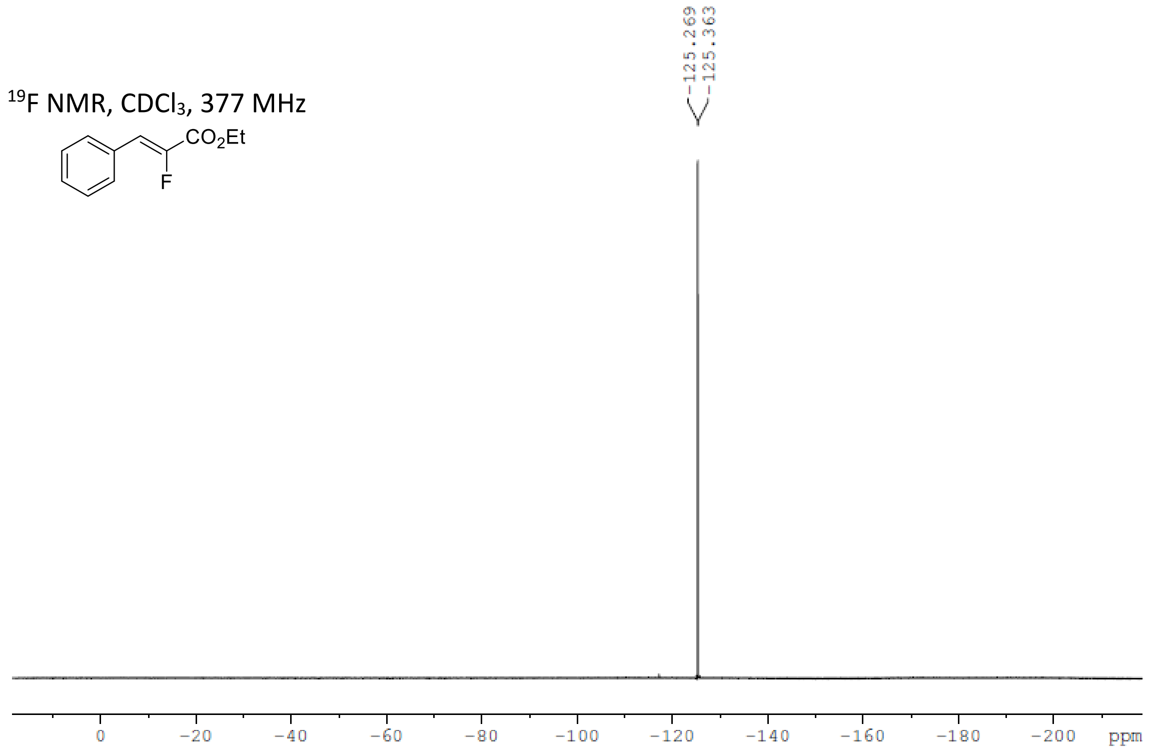
-125.269
-125.363
19F NMR, CDCl3, 377 MHz
CO2Et
F
0
-20
-40
-60
-80
-100
-120
-140
-160
-180
-200
ppm

Ethyl (*Z*)-2-fluoro-3-(4-trifluoromethylphenyl)acrylate ((*Z*)-S3-6b)

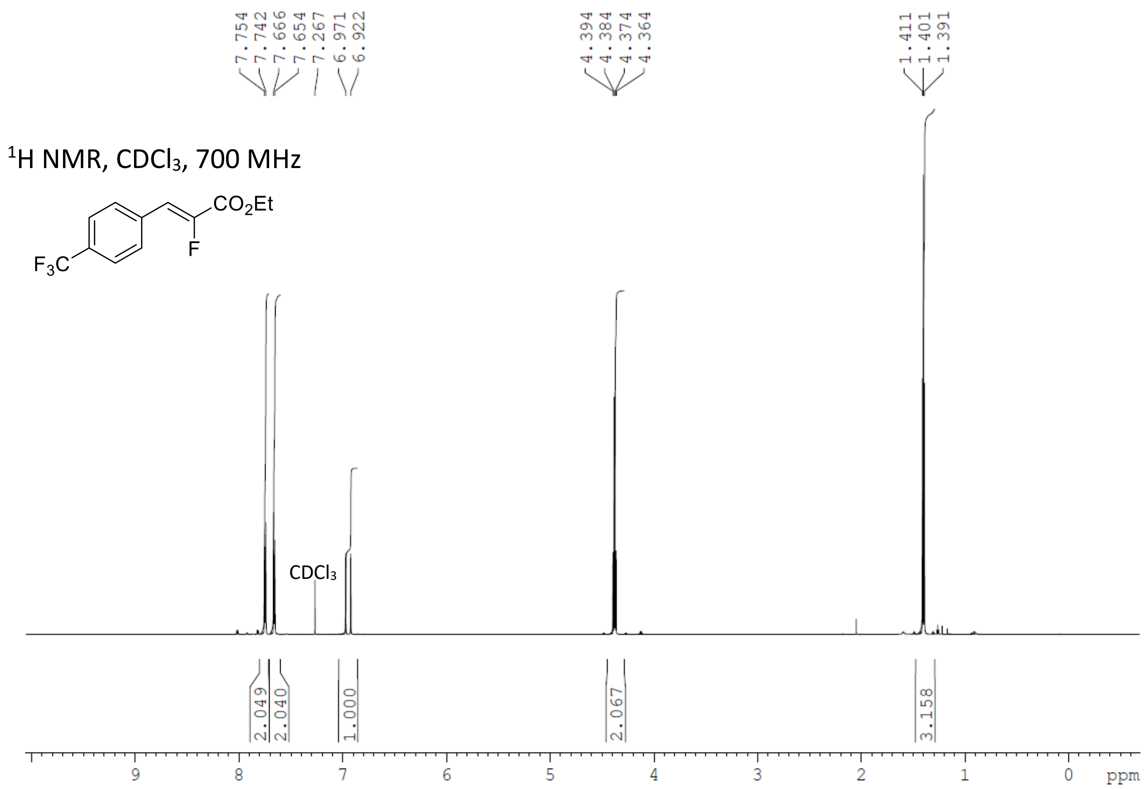


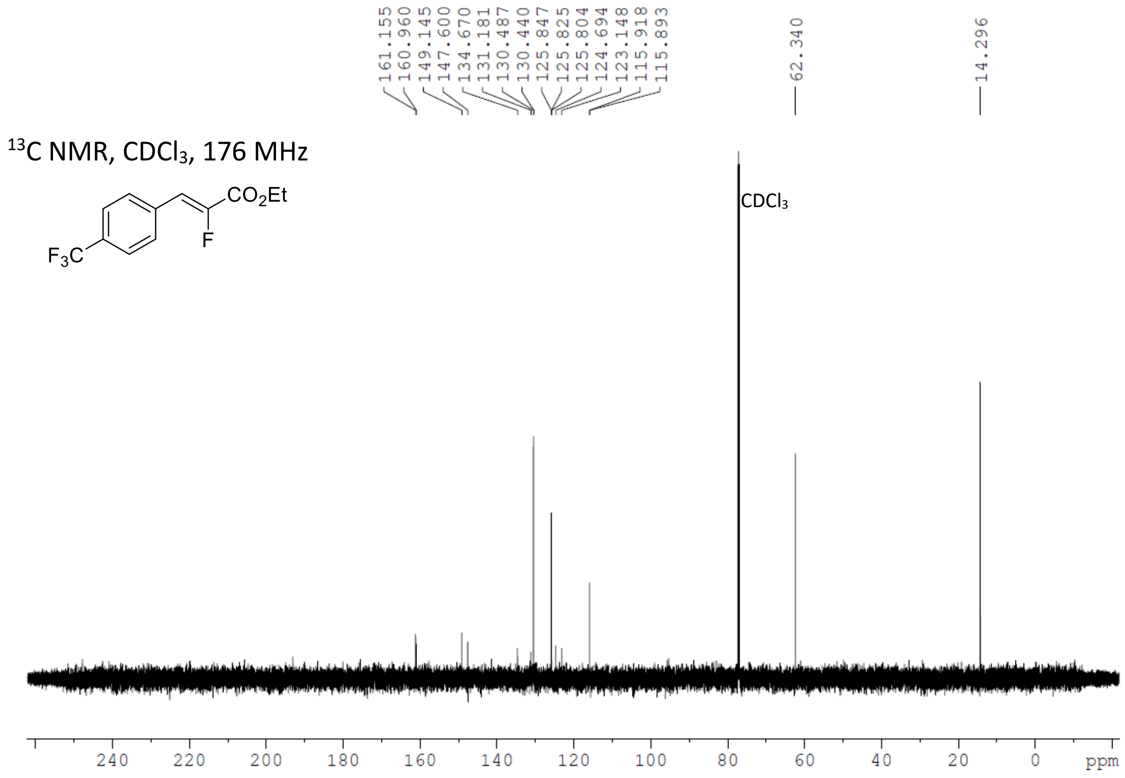

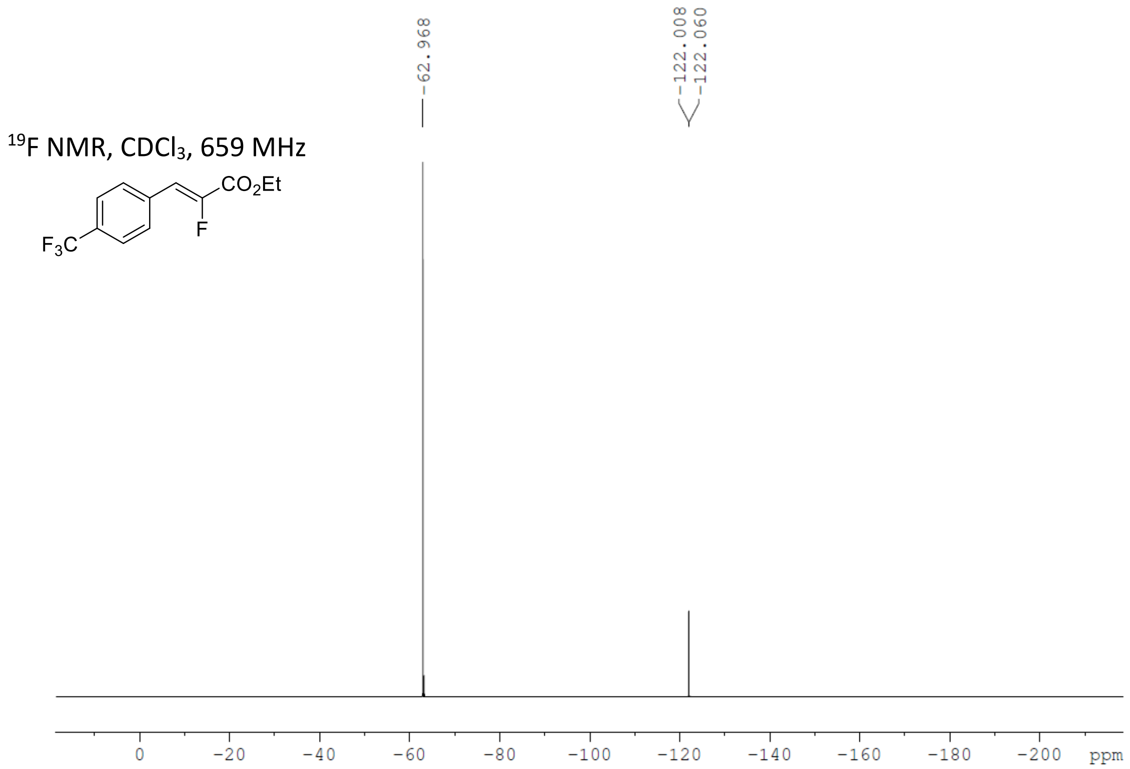


## Ethyl (*Z*)-2-fluoro-3-(4-methoxyphenyl)acrylate ((*Z*)-S3-6c)

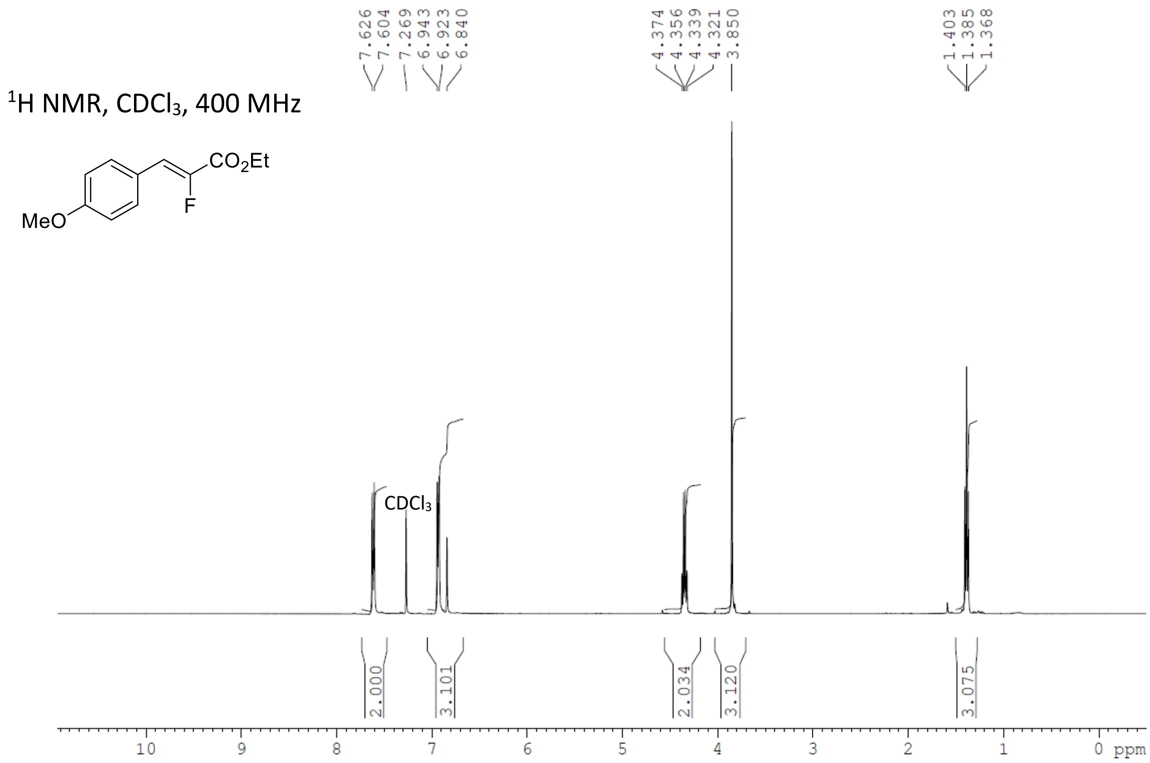

$^{13}C$ NMR, $CDCl_3$, 100 MHz

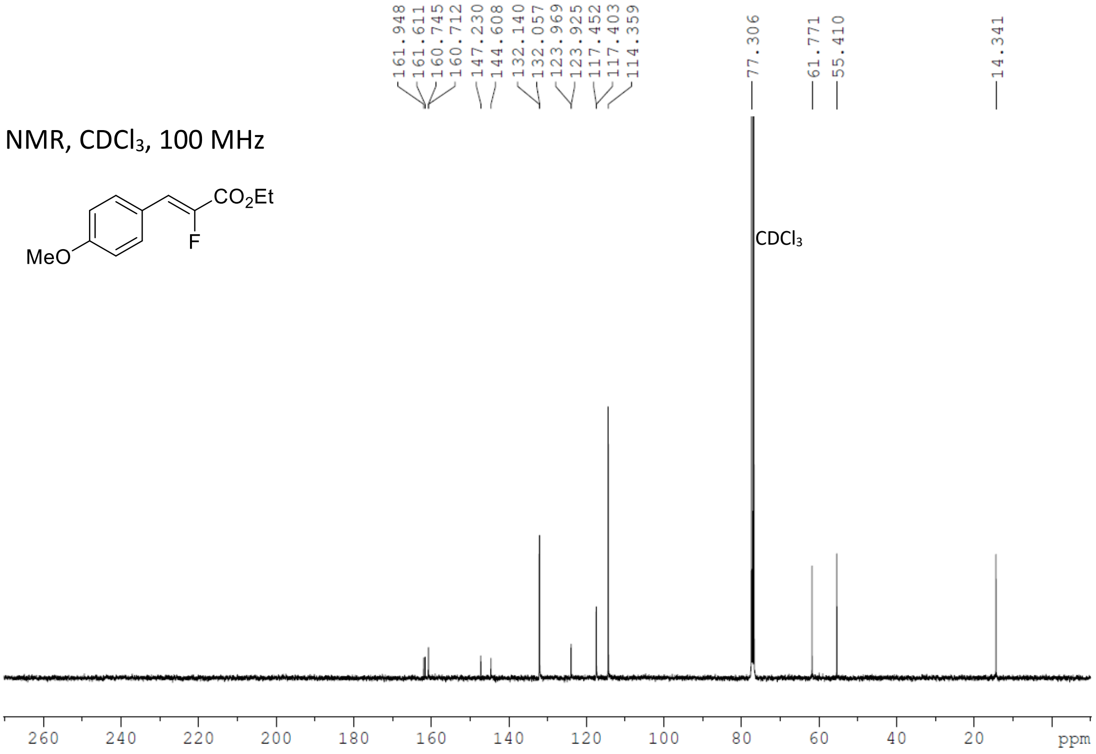


$^{19}F$ NMR, $CDCl_3$, 377 MHz

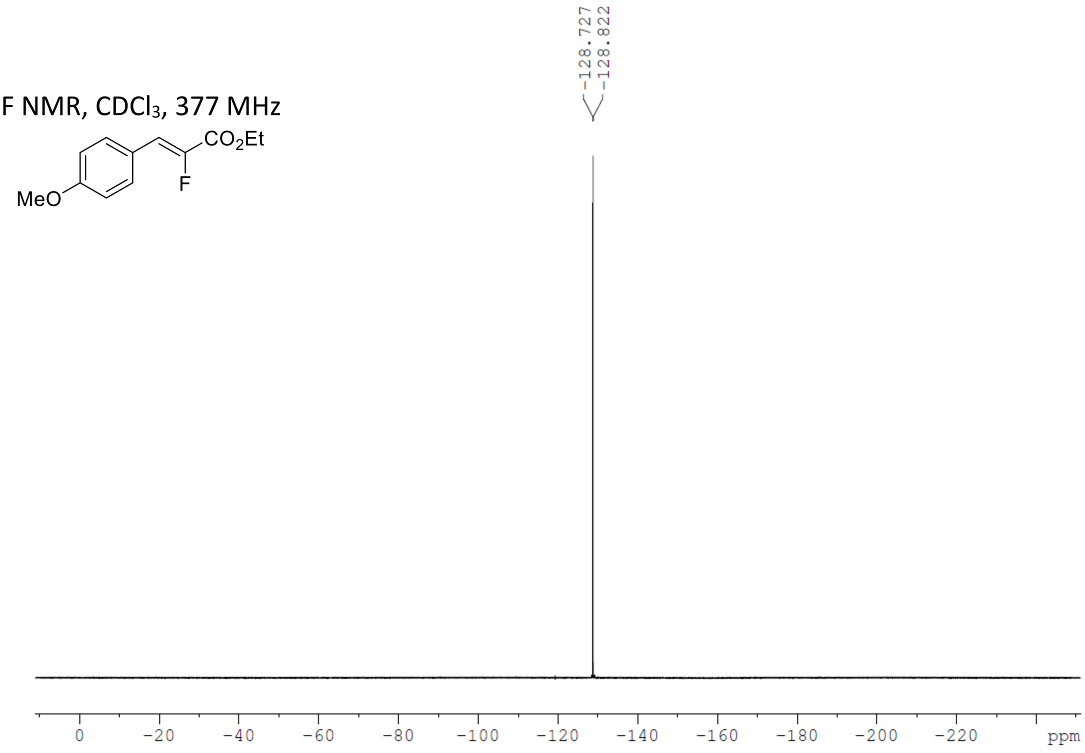

## (*Z*)-2-Fluoro-1-phenylbut-1-ene-3-one ((*Z*)-1a)

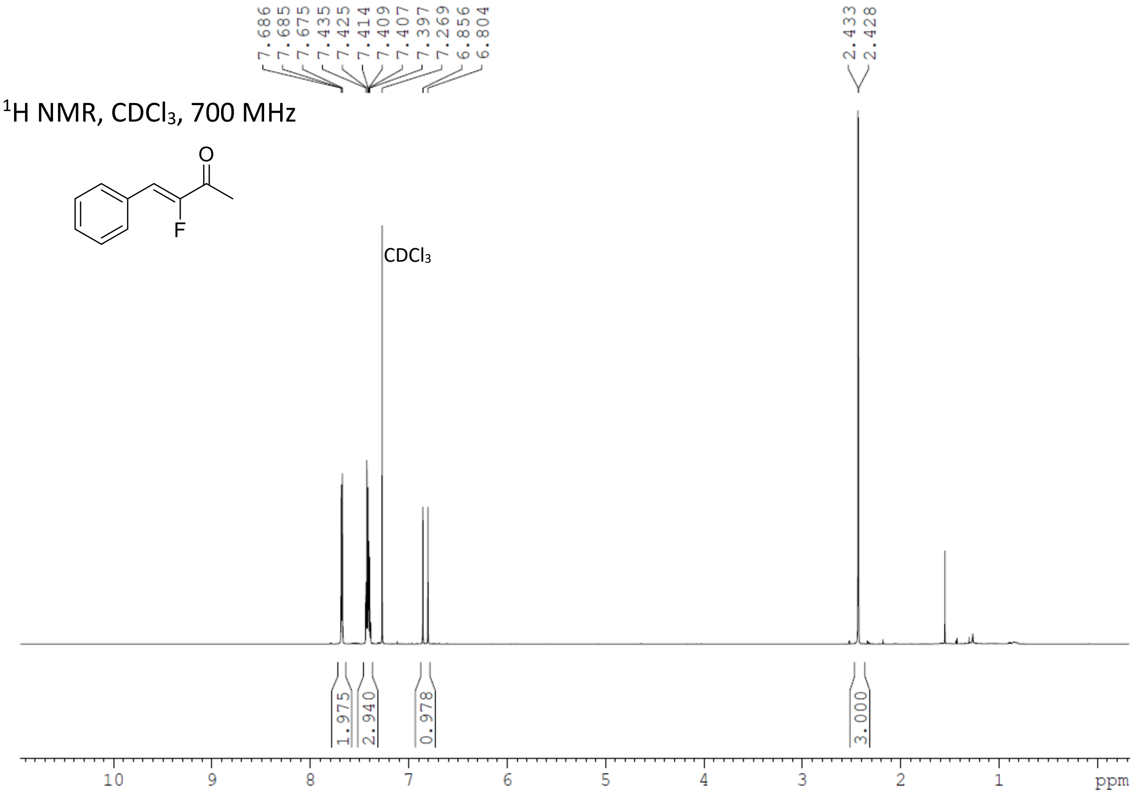


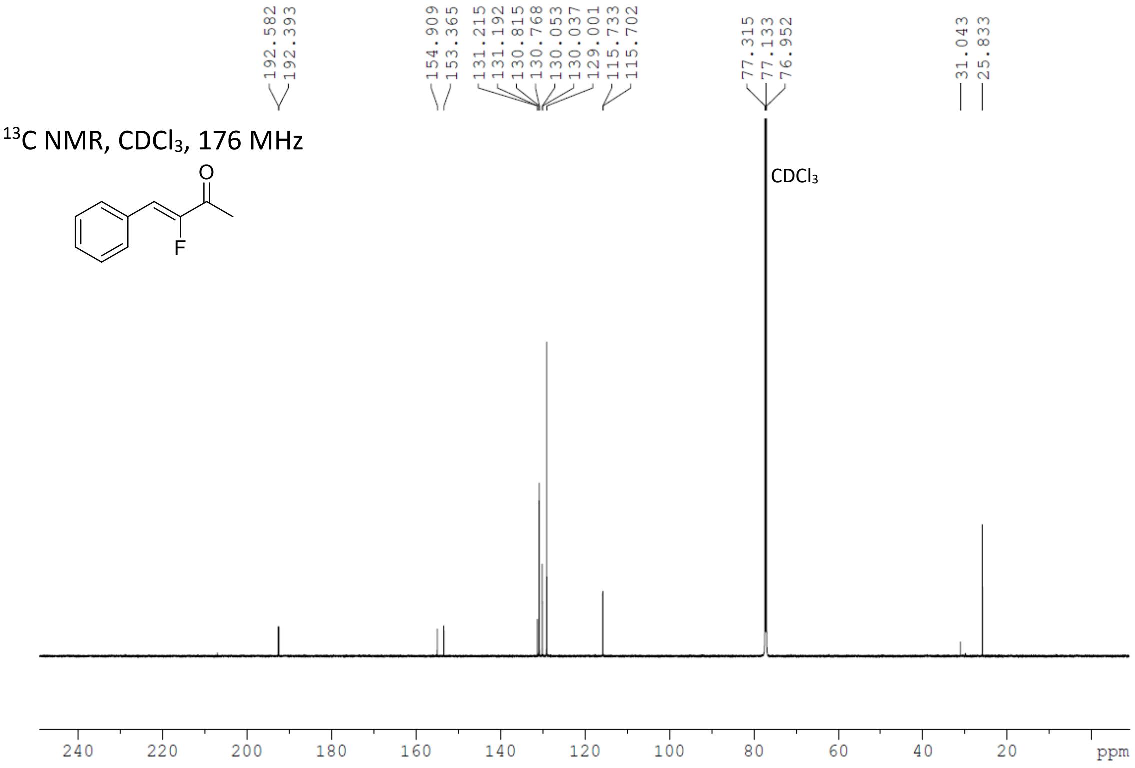

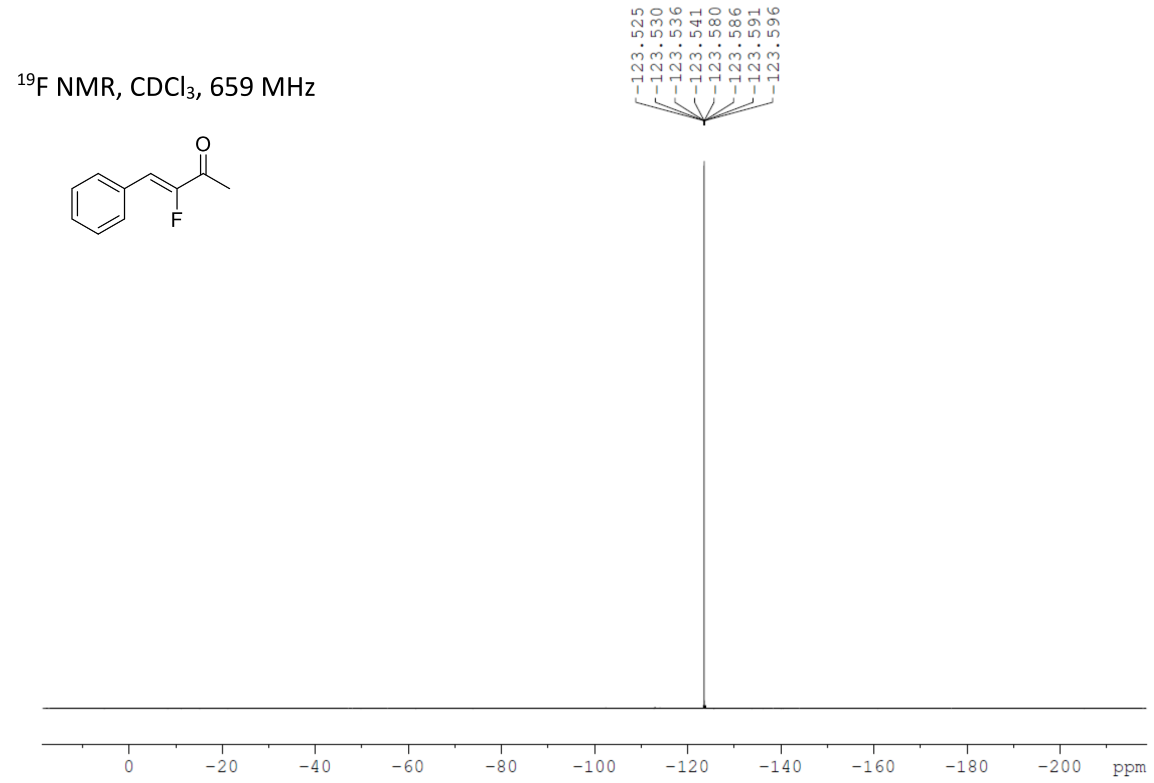


## (*E*)-2-Fluoro-1-phenylbut-1-ene-3-one ((*E*)-1a)

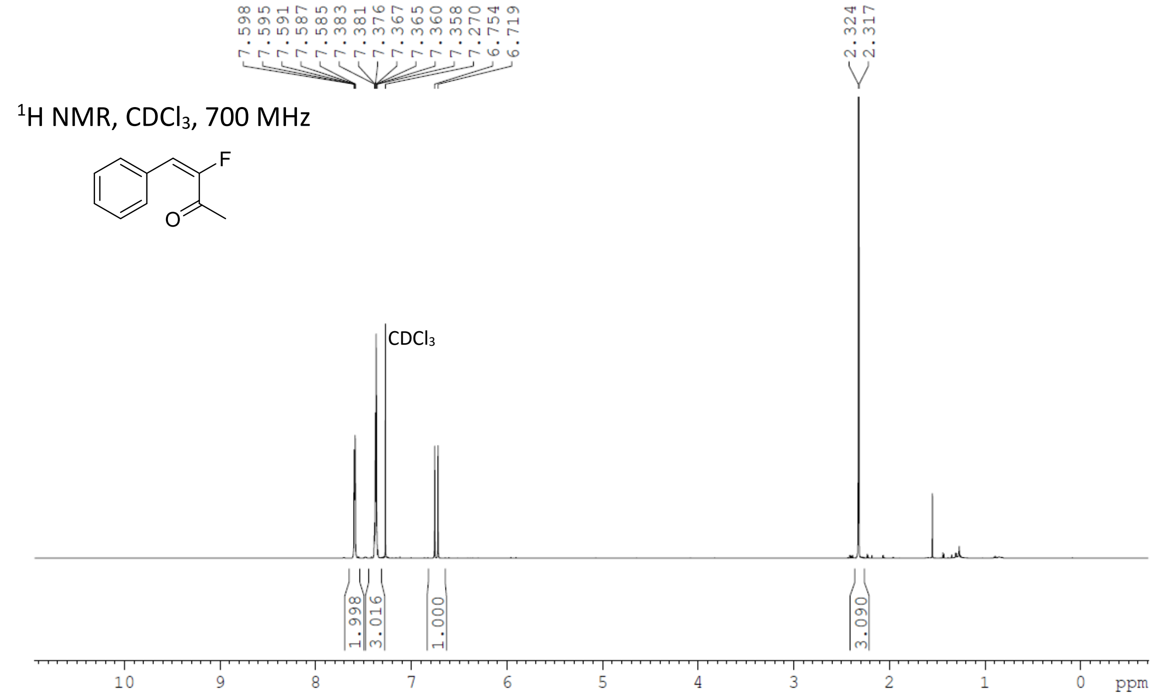

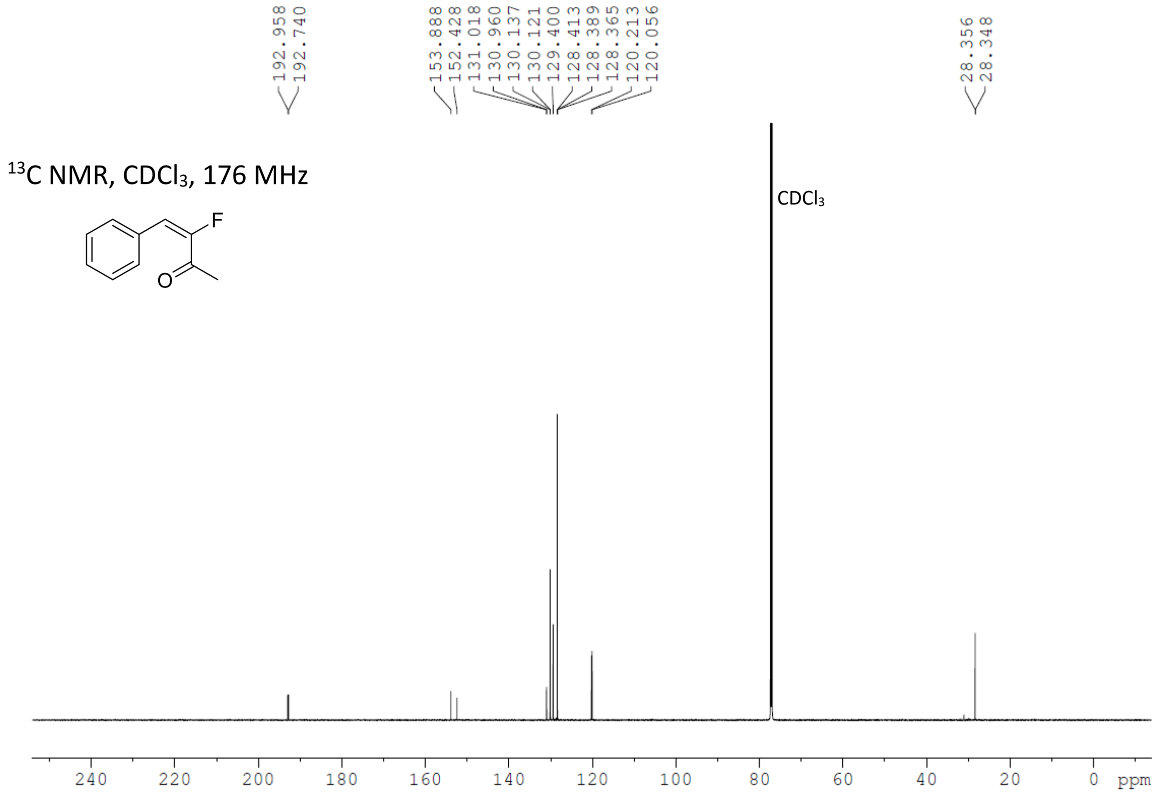
192.958
192.740
153.888
152.428
131.018
130.960
130.137
130.121
129.400
128.413
128.389
128.365
120.213
120.056
28.356
28.348
$^{13}C$ NMR, $CDCl_3$, 176 MHz
F
O
$CDCl_3$
240 220 200 180 160 140 120 100 80 60 40 20 0 ppm

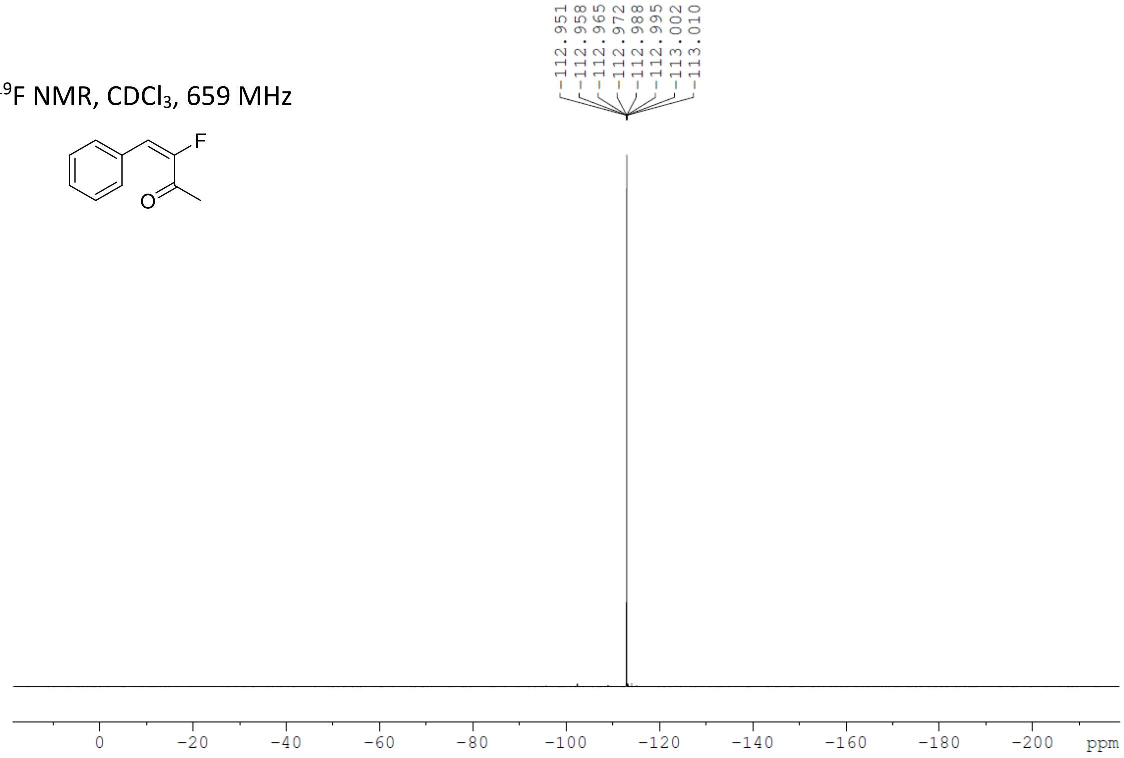
-112.951
-112.958
-112.965
-112.972
-112.988
-112.995
-113.002
-113.010
$^{19}F$ NMR, $CDCl_3$, 659 MHz
F
O
0 -20 -40 -60 -80 -100 -120 -140 -160 -180 -200 ppm

(*Z*)-3-Fluoro-4-(4-trifluoromethylphenyl)-3-buten-2-one ((*Z*)-1b)

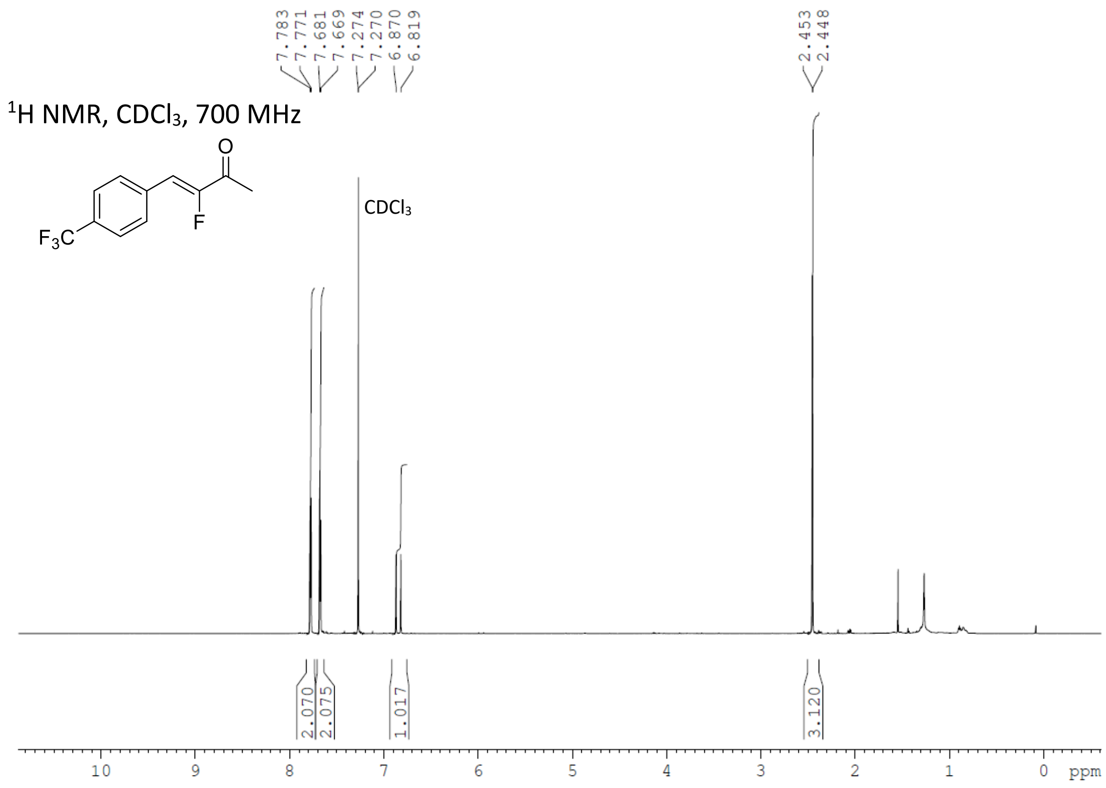


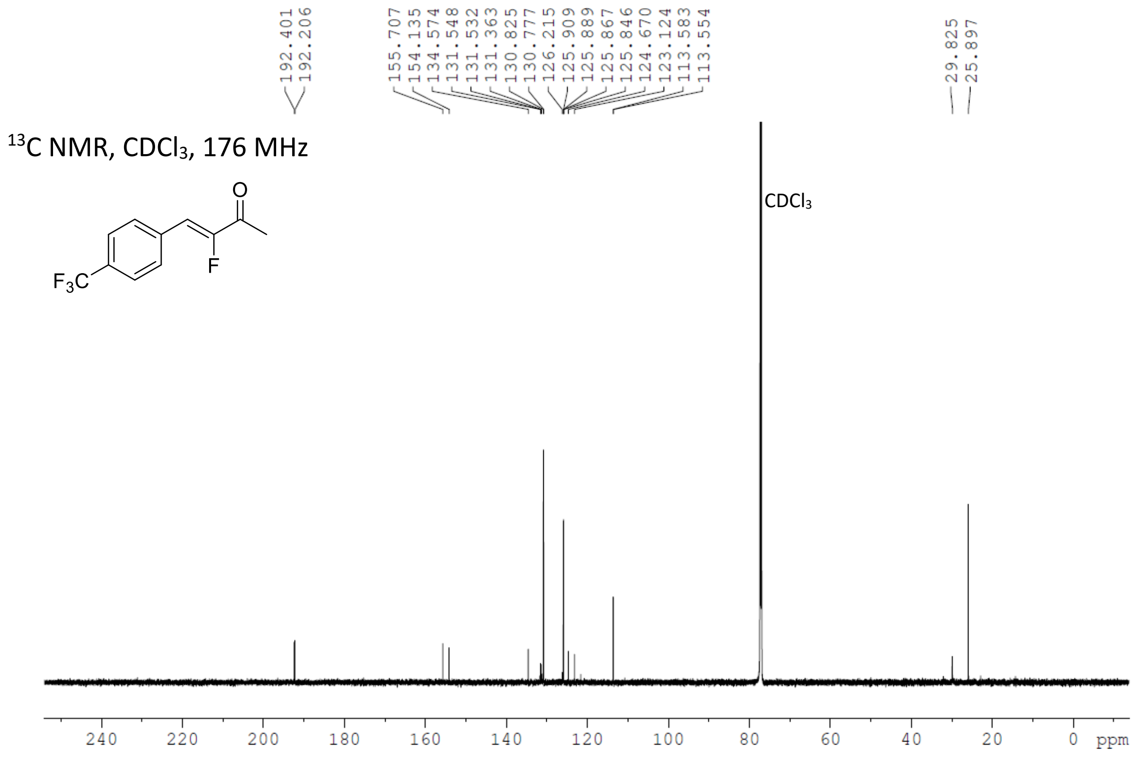

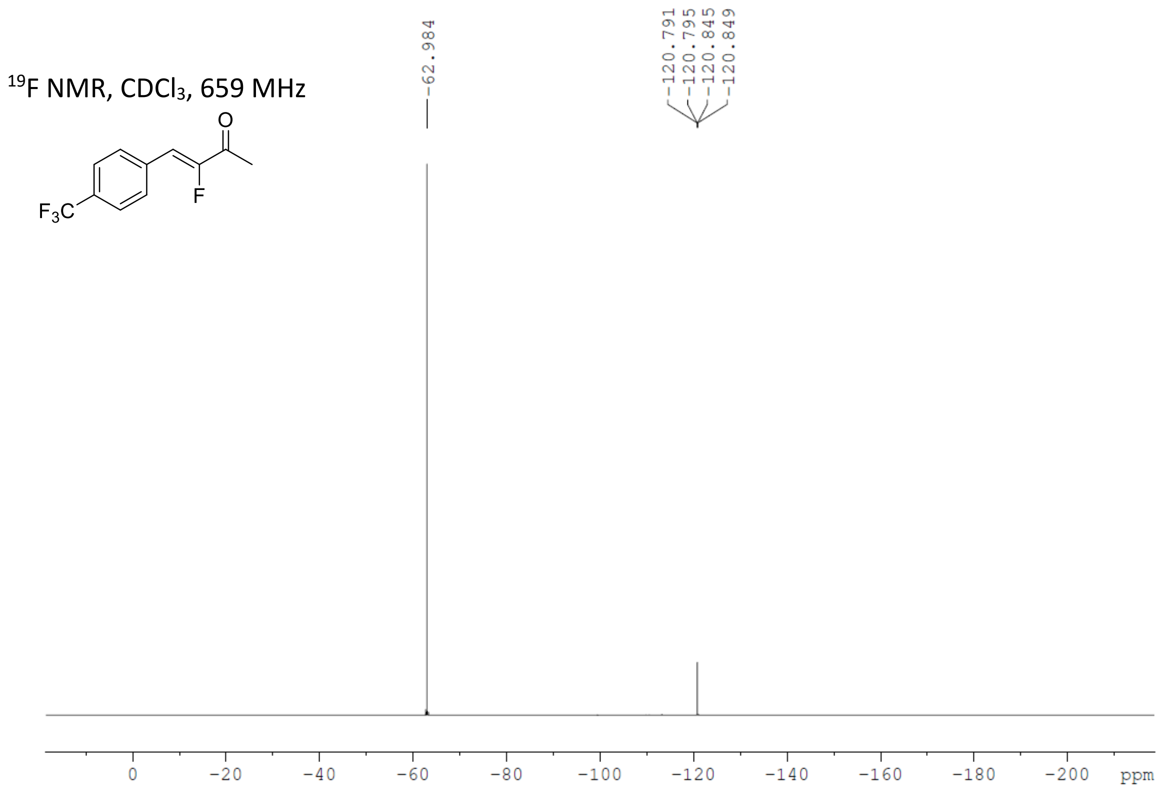


## (*E*)-3-Fluoro-4-(4-trifluoromethylphenyl)-3-buten-2-one ((*E*)-1b)

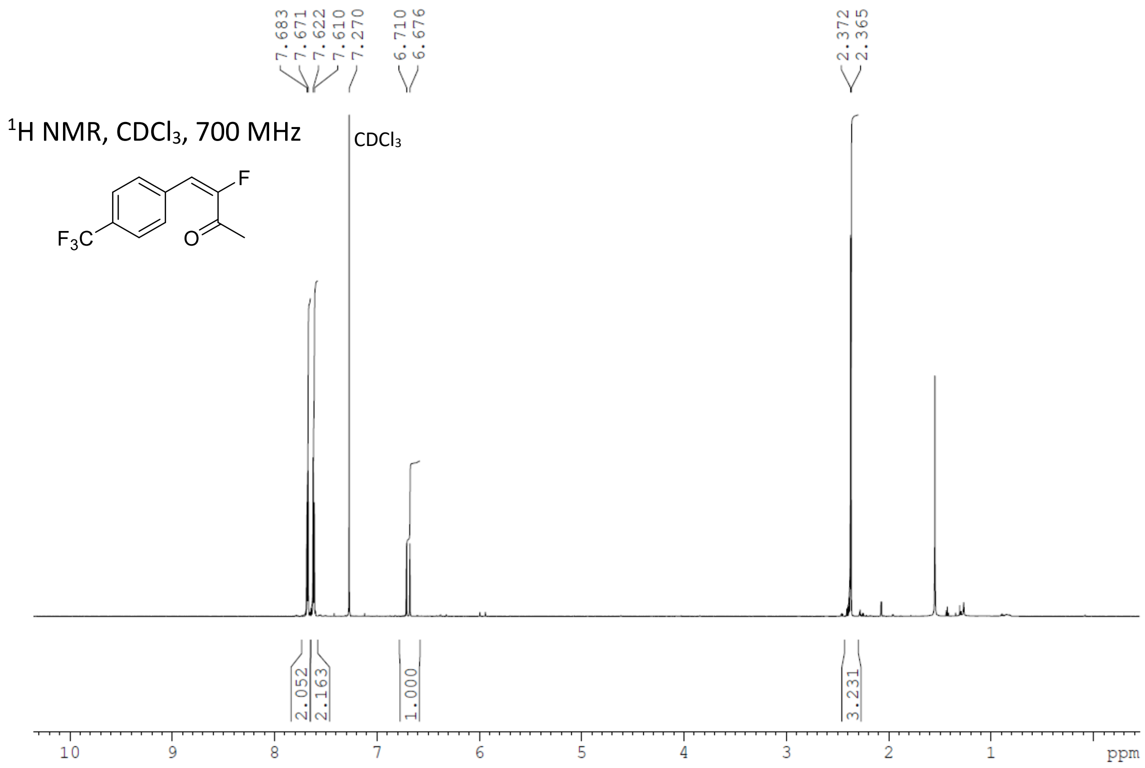

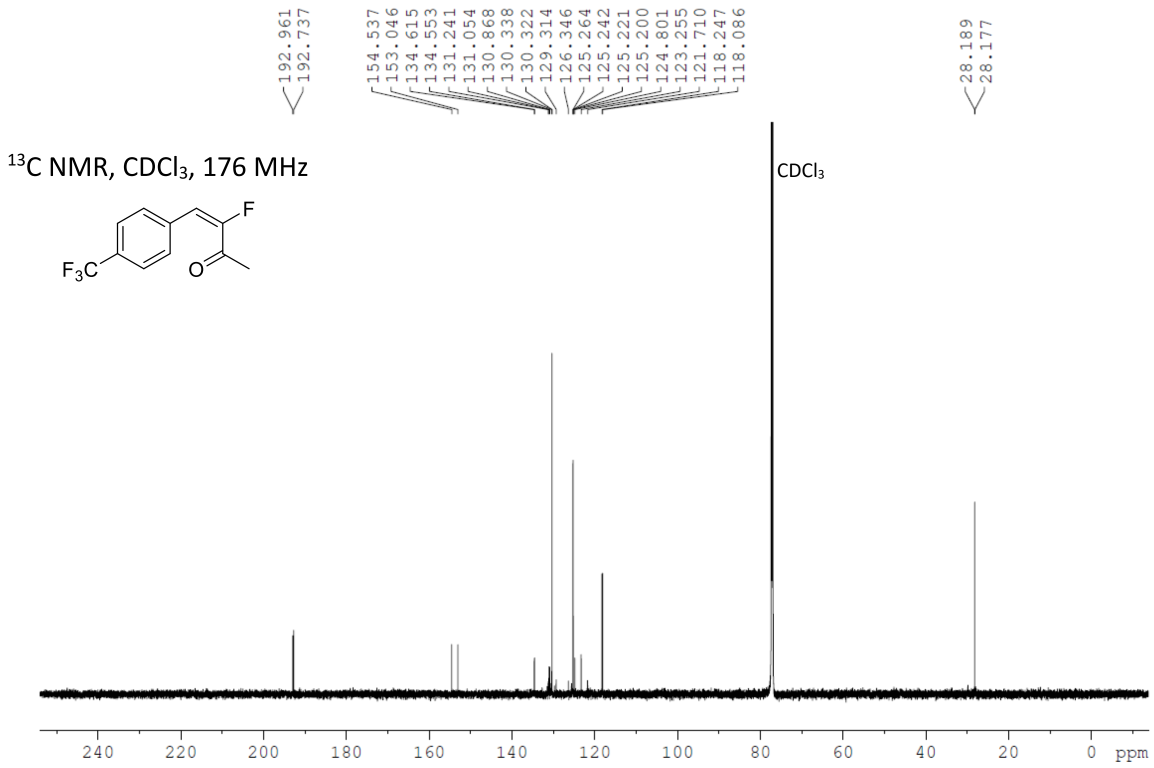

192.961
192.737
154.537
153.046
134.615
134.553
131.241
131.054
130.868
130.338
130.322
129.314
126.346
125.264
125.242
125.221
125.200
124.801
123.255
121.710
118.247
118.086
28.189
28.177
$^{13}C$ NMR, $CDCl_3$, 176 MHz
F
$F_3C$
O
$CDCl_3$
240
220
200
180
160
140
120
100
80
60
40
20
0
ppm


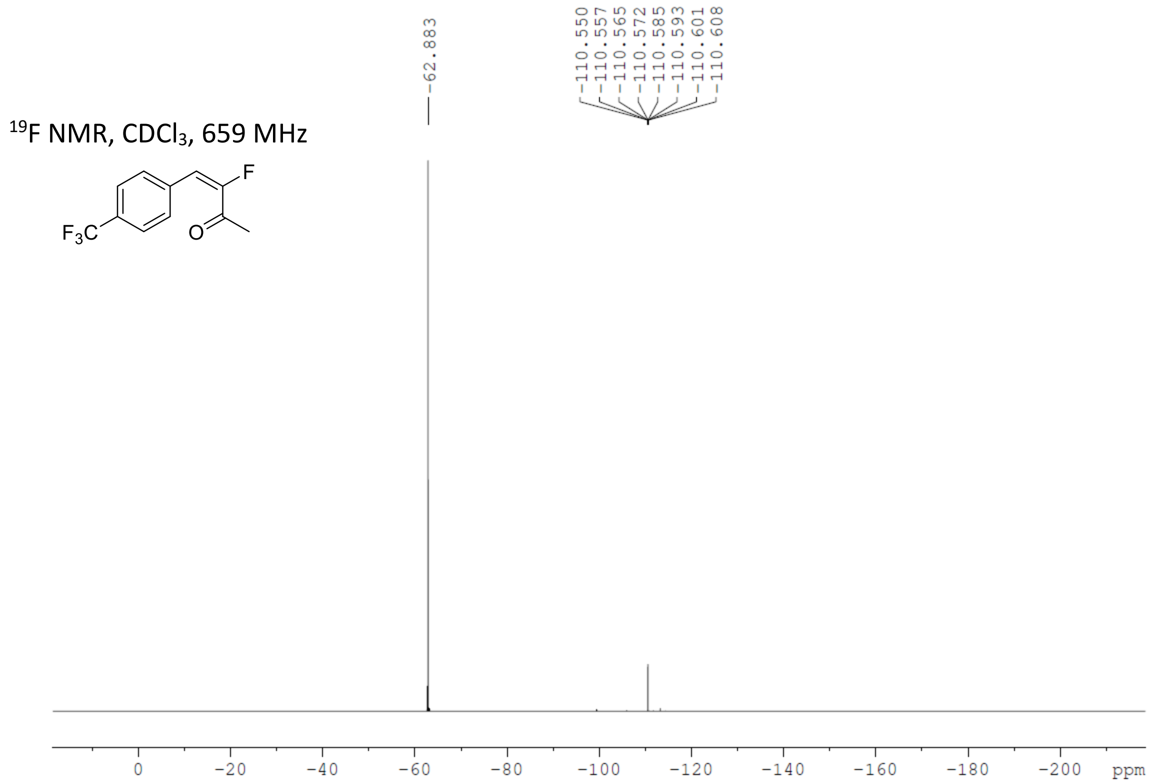

-62.883
-110.550
-110.557
-110.565
-110.572
-110.585
-110.593
-110.601
-110.608
$^{19}F$ NMR, $CDCl_3$, 659 MHz
F
$F_3C$
O
0
-20
-40
-60
-80
-100
-120
-140
-160
-180
-200
ppm

(*Z*)-3-Fluoro-4-(4-methoxyphenyl)-3-buten-2-one ((*Z*)-1c)

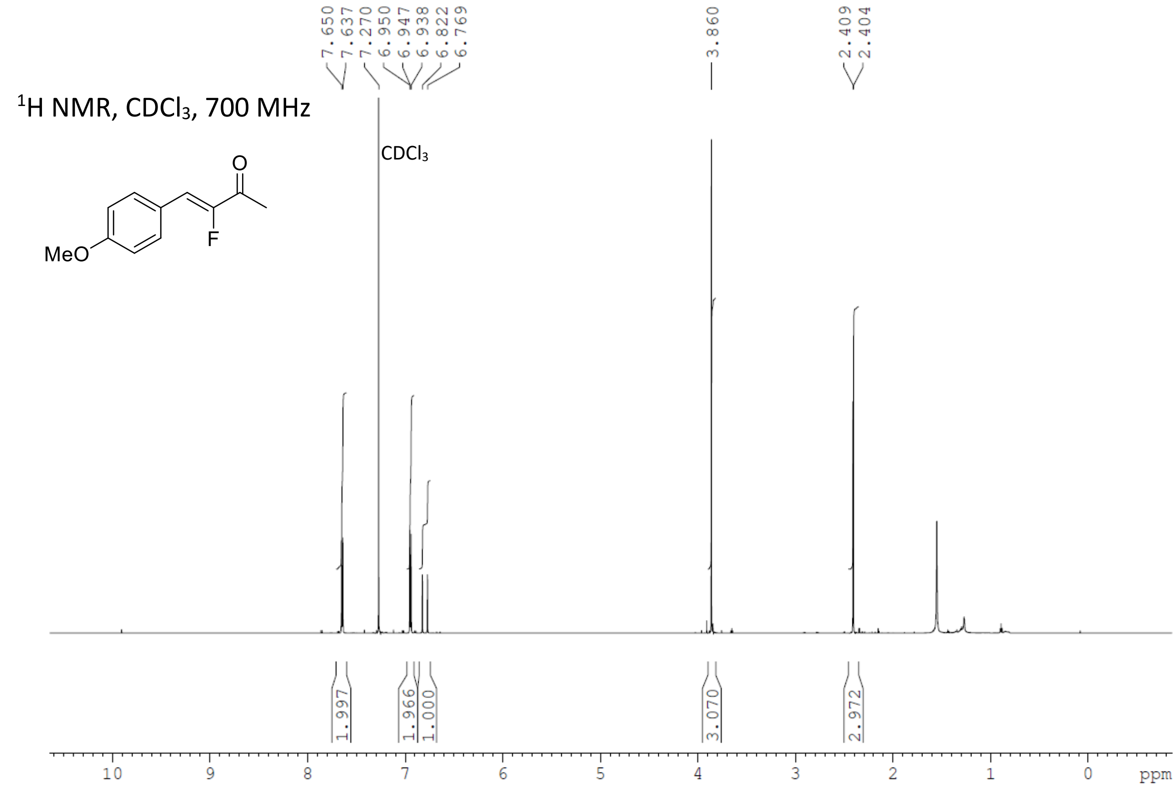


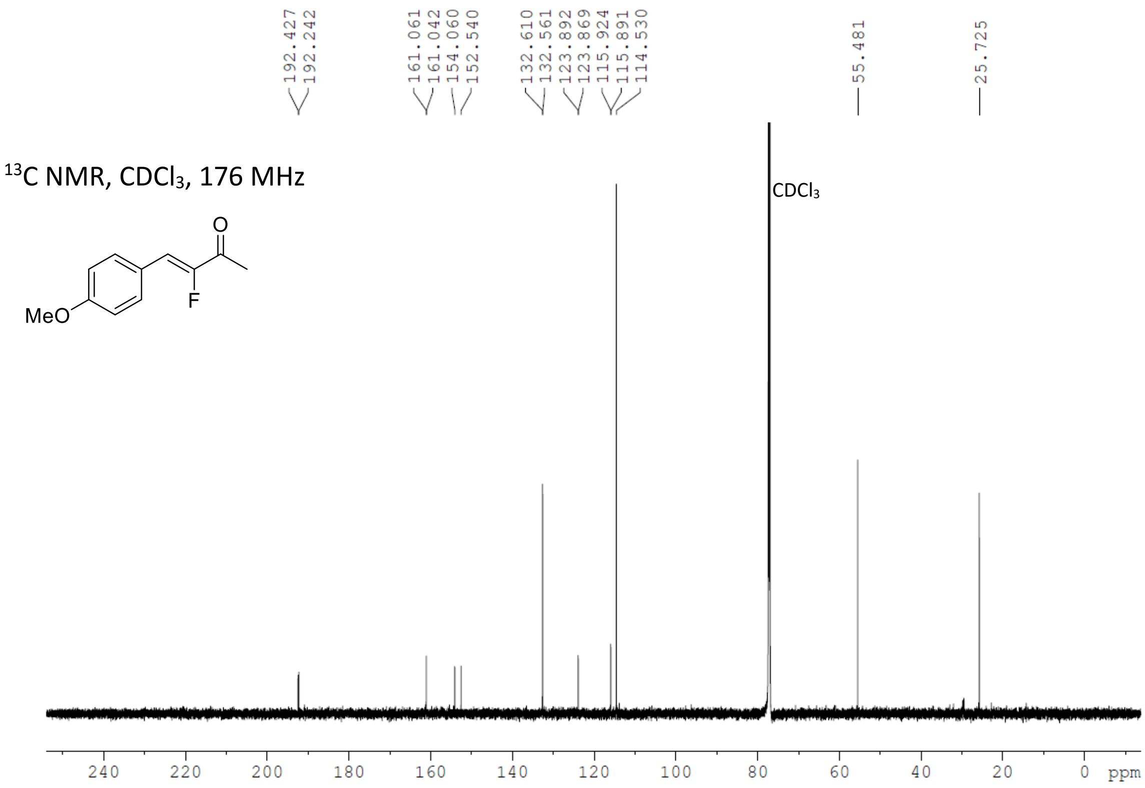

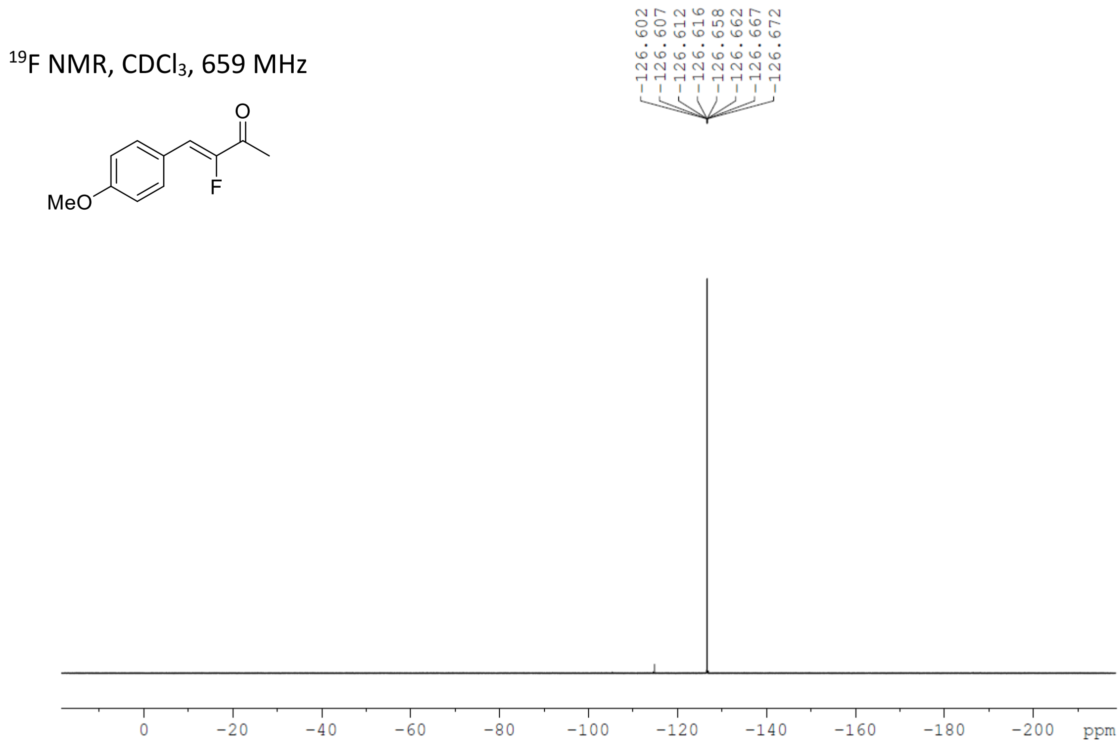


## (*E*)-3-Fluoro-4-(4-methoxyphenyl)-3-buten-2-one ((*E*)-1c)

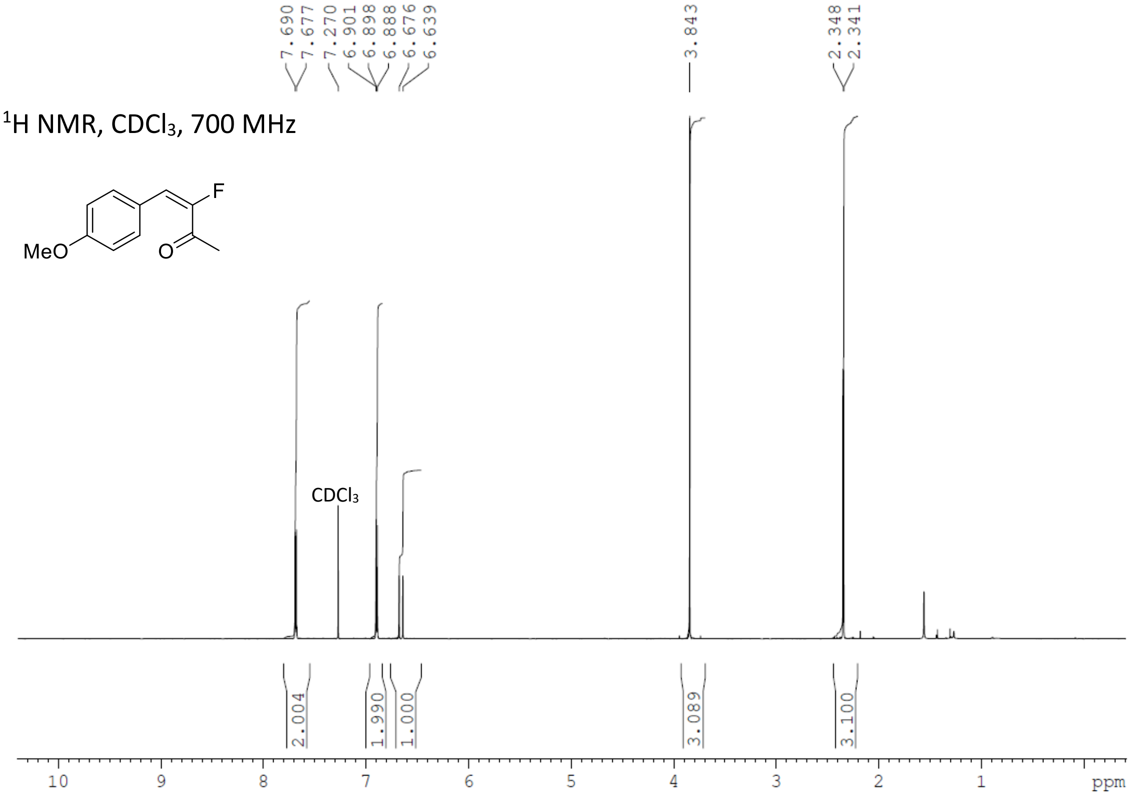

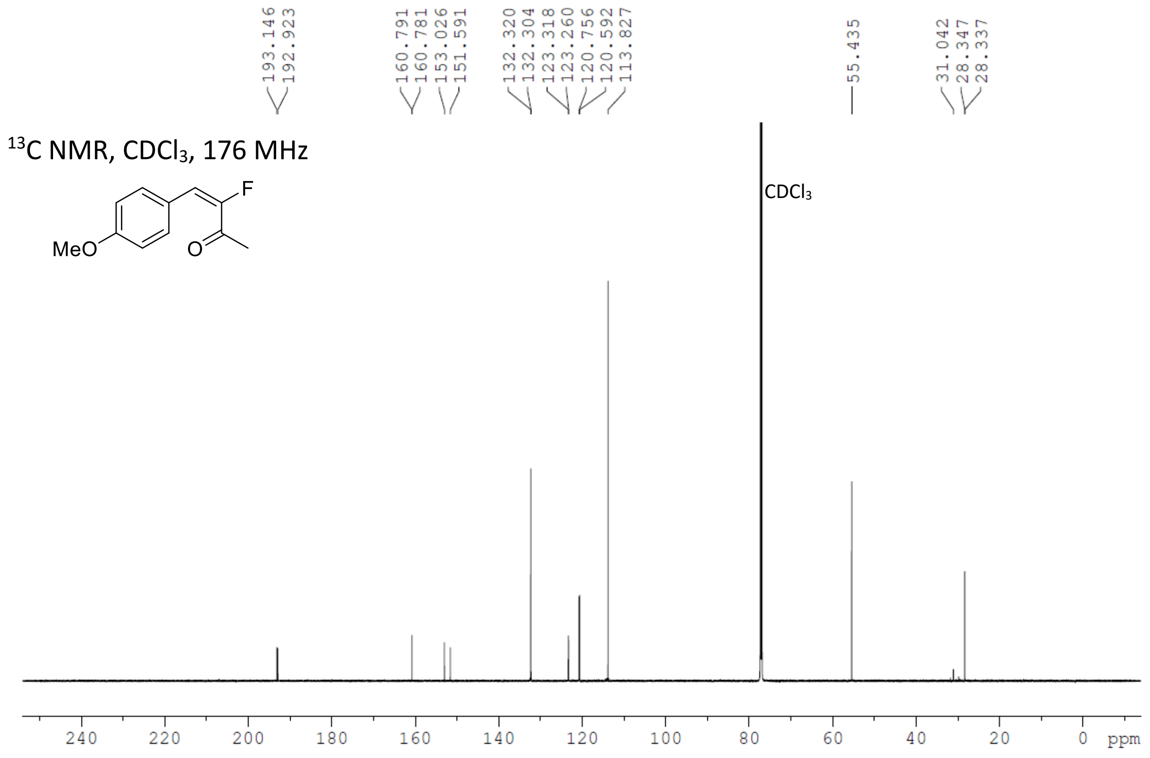

13C NMR, CDCl3, 176 MHz
MeO
F
O
CDCl3
193.146
192.923
160.791
160.781
153.026
151.591
132.320
132.304
123.318
123.260
120.756
120.592
113.827
55.435
31.042
28.347
28.337
240 220 200 180 160 140 120 100 80 60 40 20 0 ppm


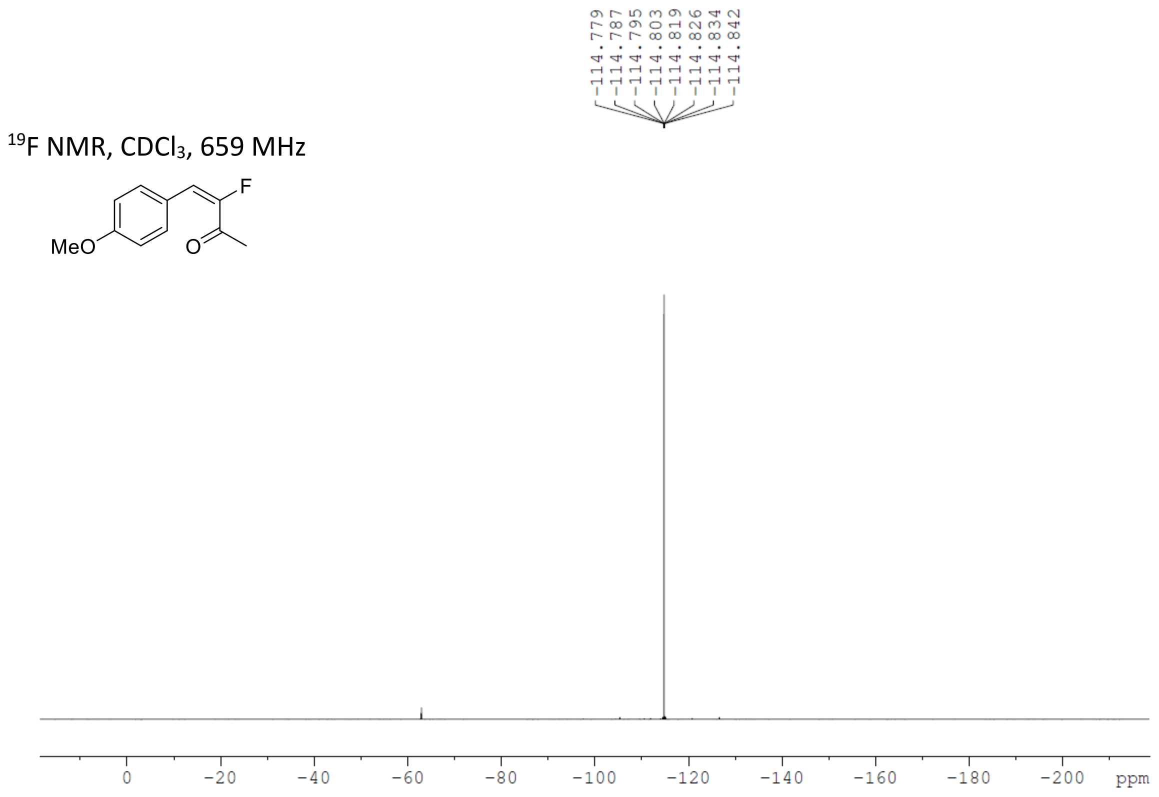

19F NMR, CDCl3, 659 MHz
MeO
F
O
-114.779
-114.787
-114.795
-114.803
-114.819
-114.826
-114.834
-114.842
0 -20 -40 -60 -80 -100 -120 -140 -160 -180 -200 ppm

(*Z*)-3-Fluoro-4-(4-cyanophenyl)-3-buten-2-one ((*Z*)-1d)

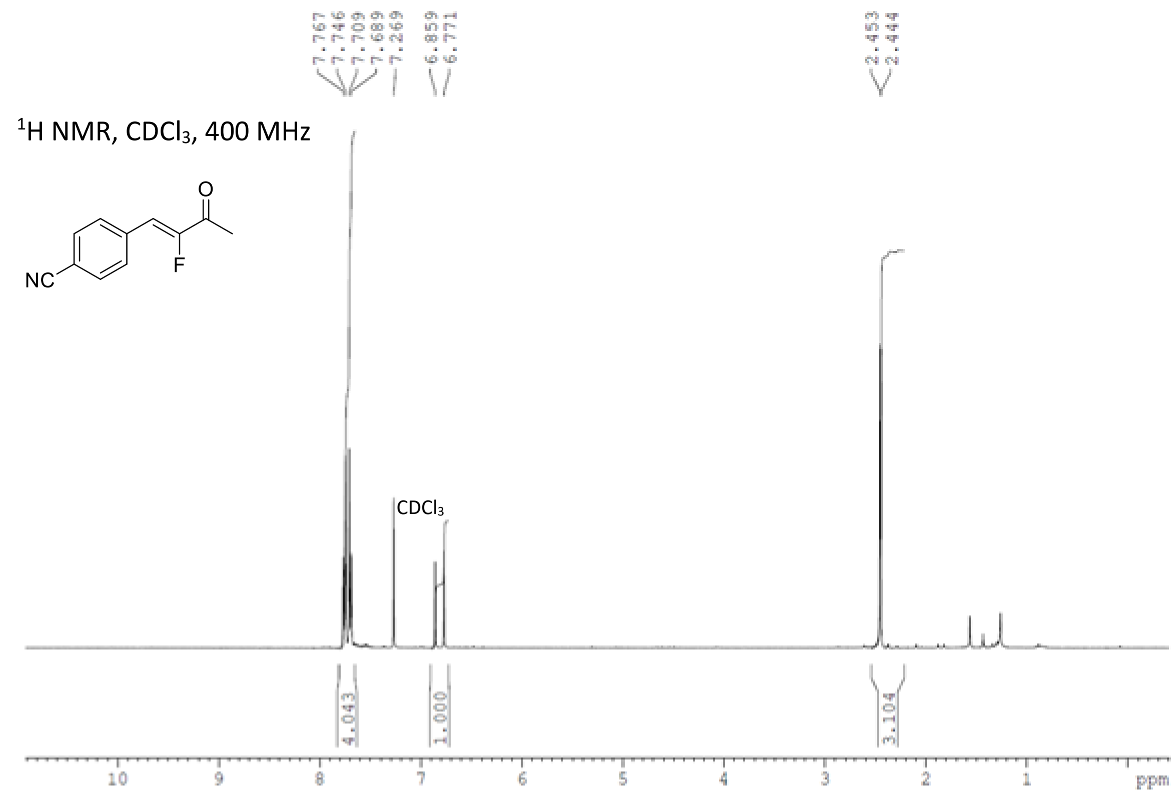


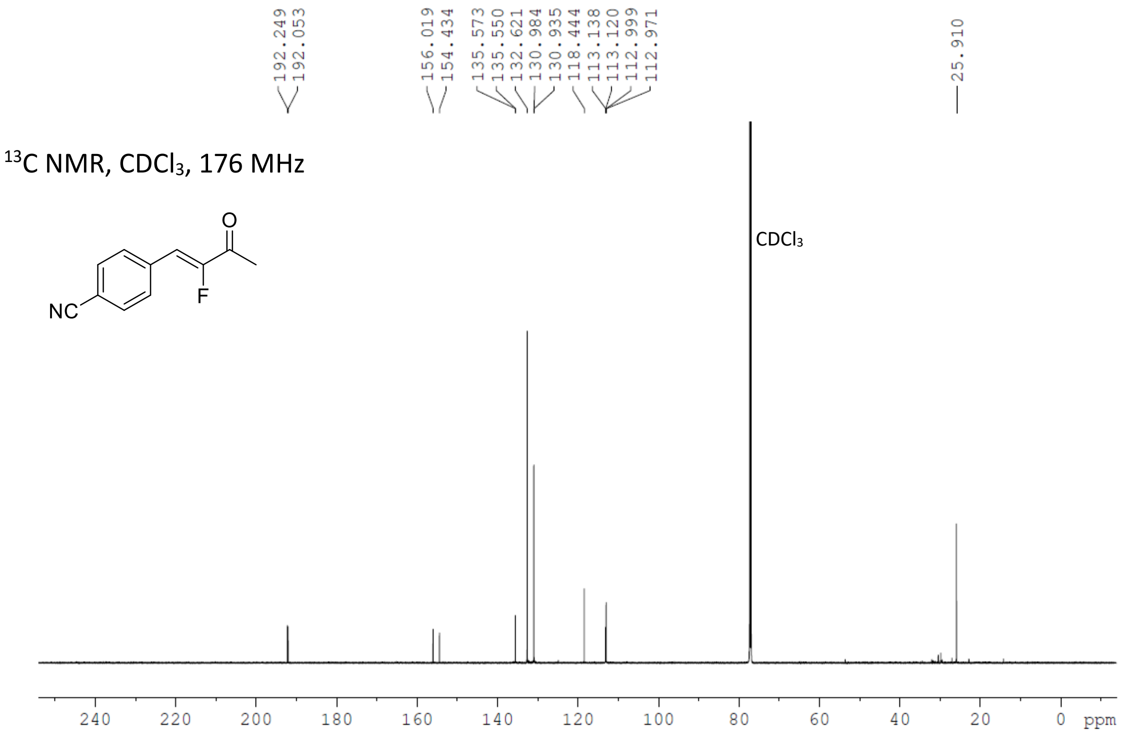

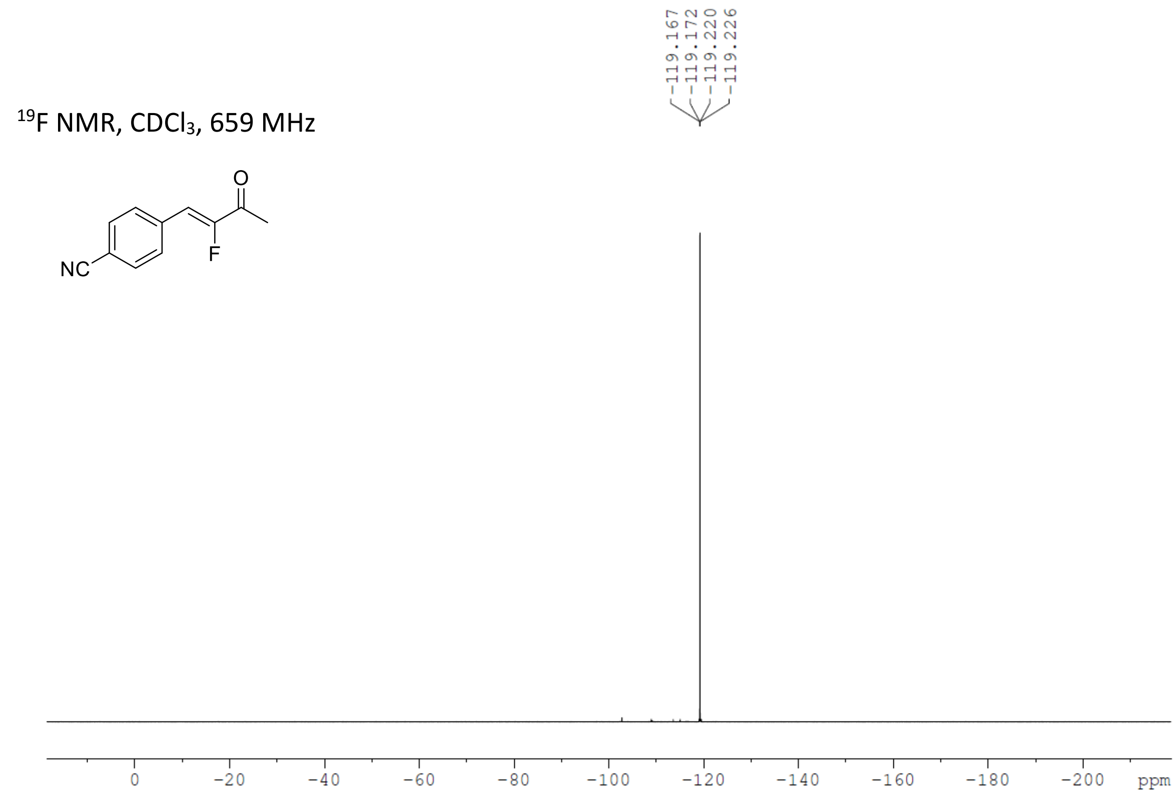


## (*E*)-3-Fluoro-4-(4-cyanophenyl)-3-buten-2-one ((*E*)-1d)

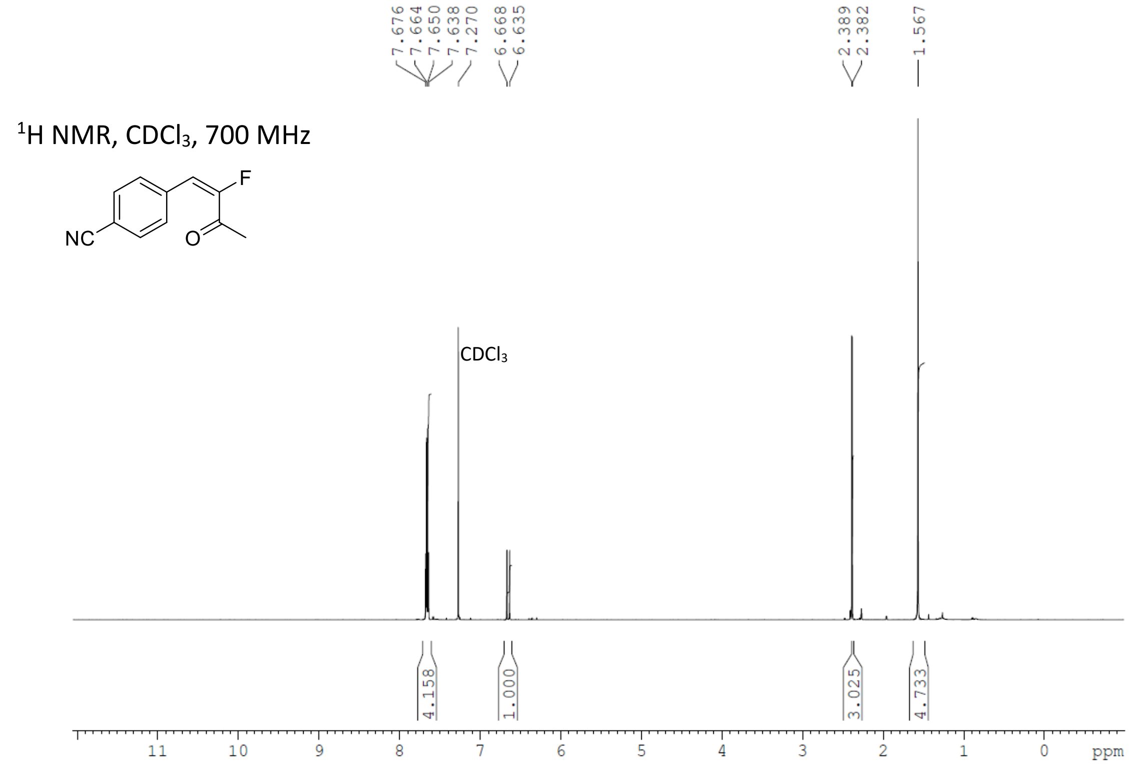

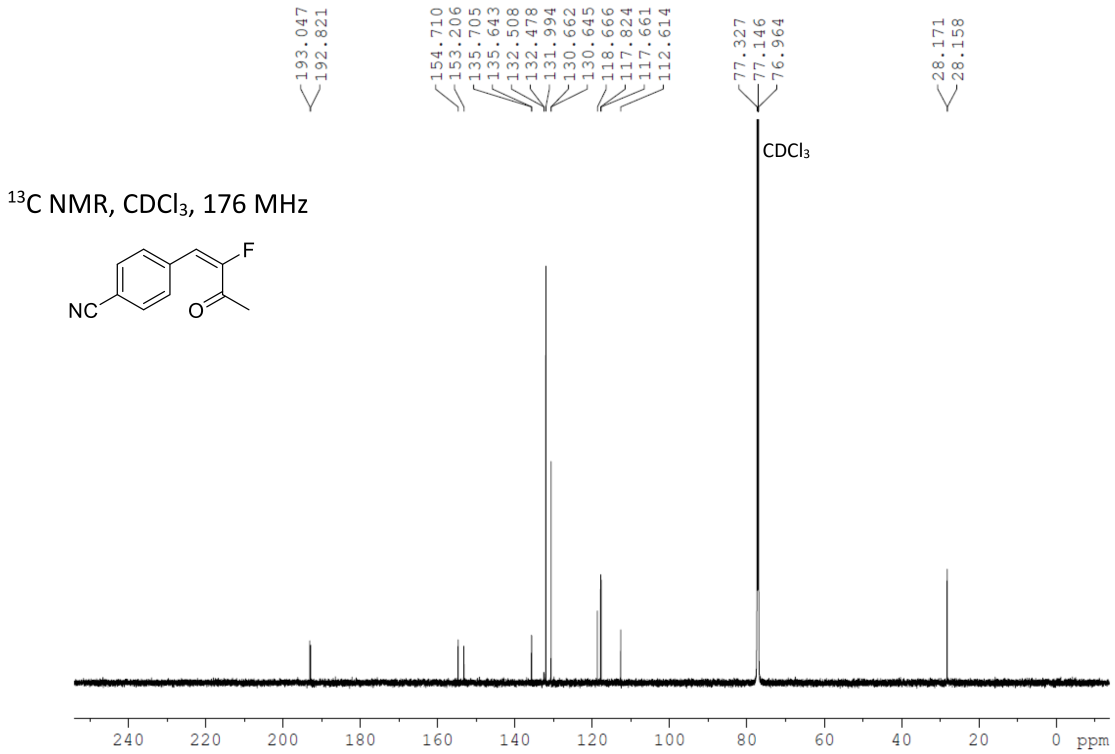
193.047
192.821
154.710
153.206
135.705
135.643
132.508
132.478
131.994
130.662
130.645
118.666
117.824
117.661
112.614
77.327
77.146
76.964
28.171
28.158
CDCl3
13C NMR, CDCl3, 176 MHz
F
NC
O
240 220 200 180 160 140 120 100 80 60 40 20 0 ppm

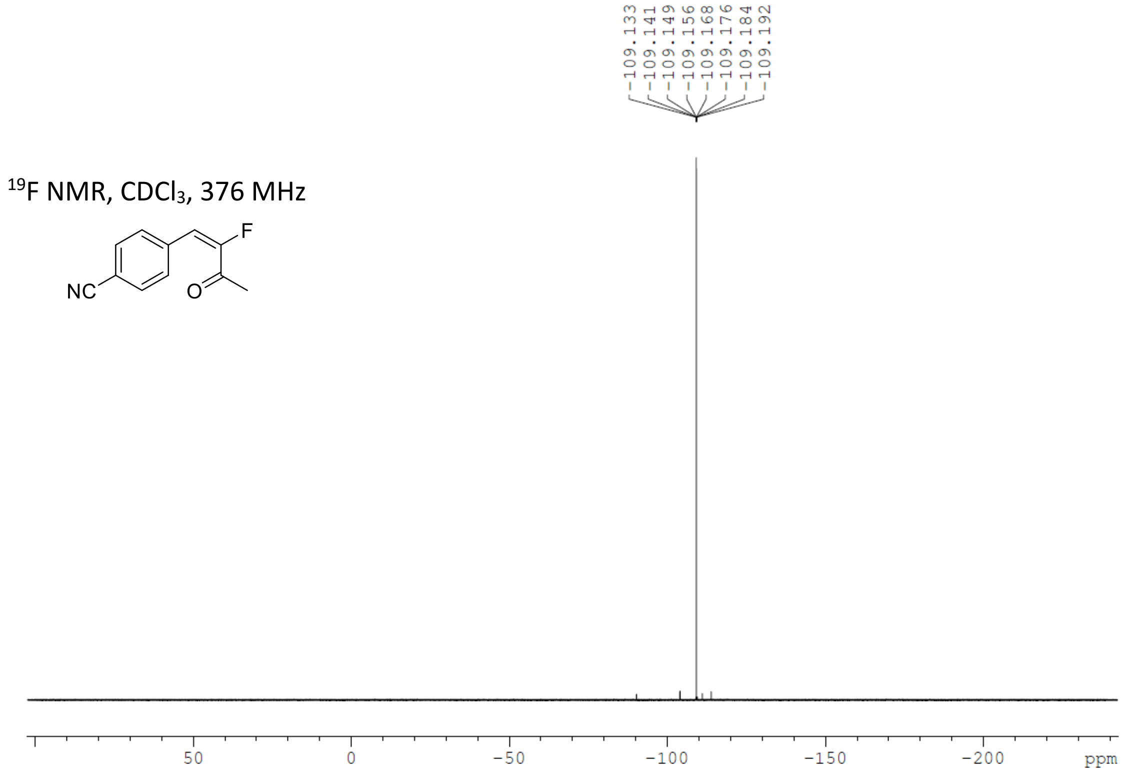
-109.133
-109.141
-109.149
-109.156
-109.168
-109.176
-109.184
-109.192
19F NMR, CDCl3, 376 MHz
F
NC
O
50 0 -50 -100 -150 -200 ppm

## (*Z*)-3-Fluoro-4-(2-methylphenyl)-3-buten-2-one ((*Z*)-1e)

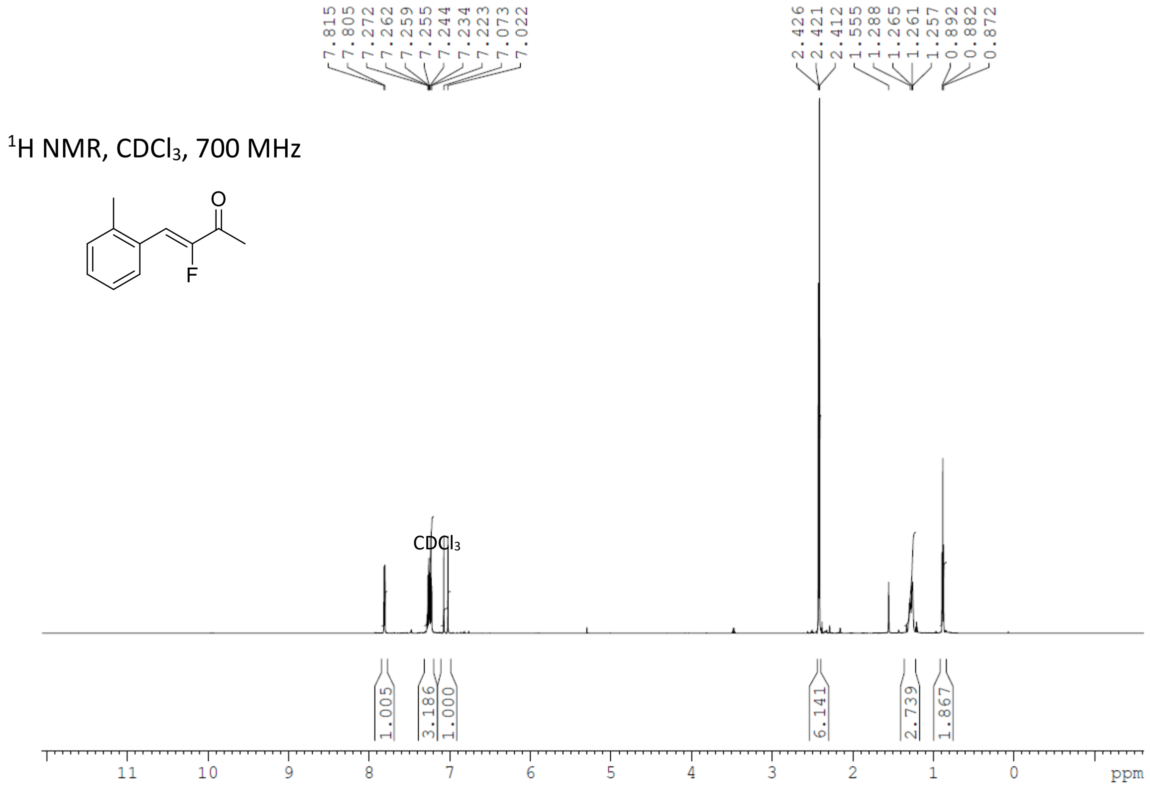


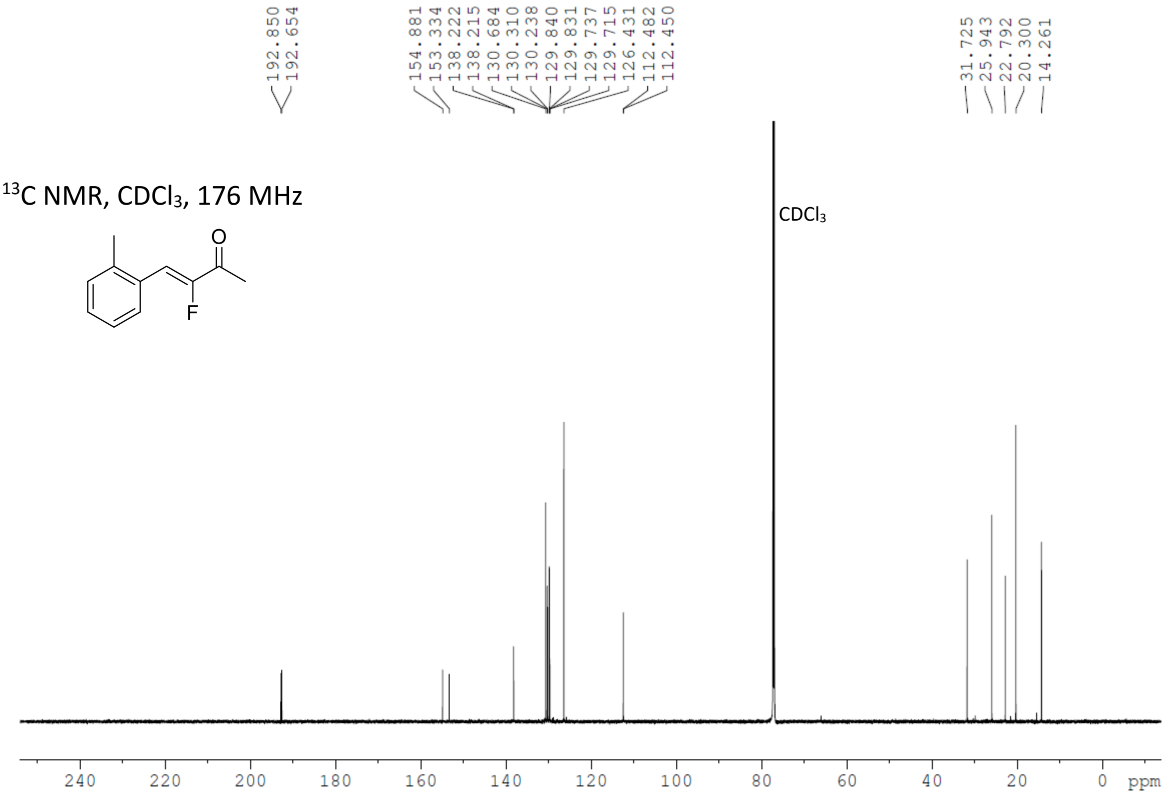

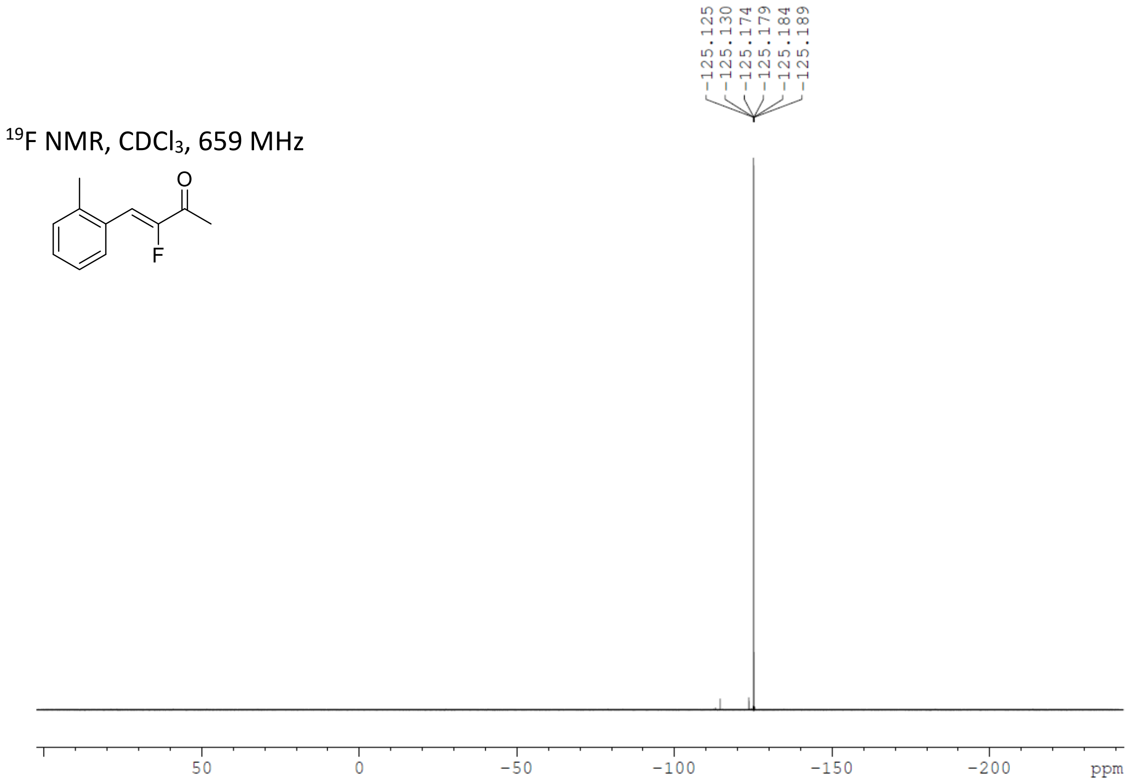


# (*E*)-3-Fluoro-4-(2-methylphenyl)-3-buten-2-one ((*E*)-1e)

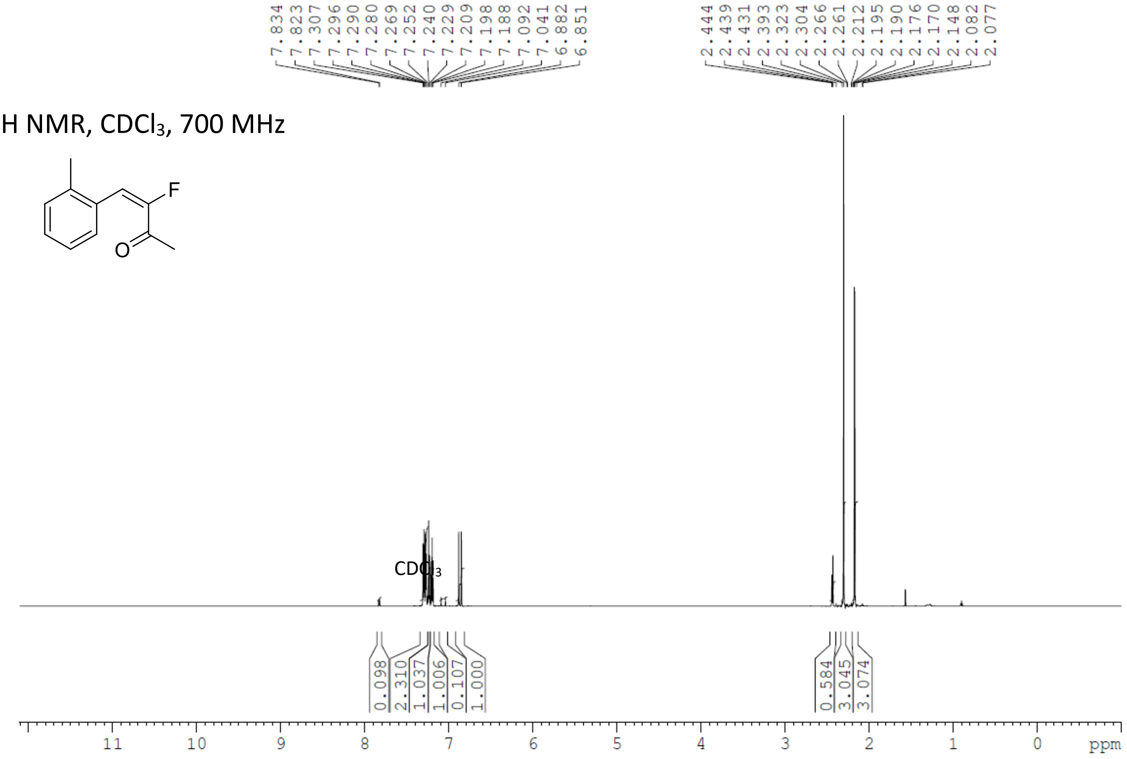

$^{13}$C NMR, $CDCl_3$, 176 MHz

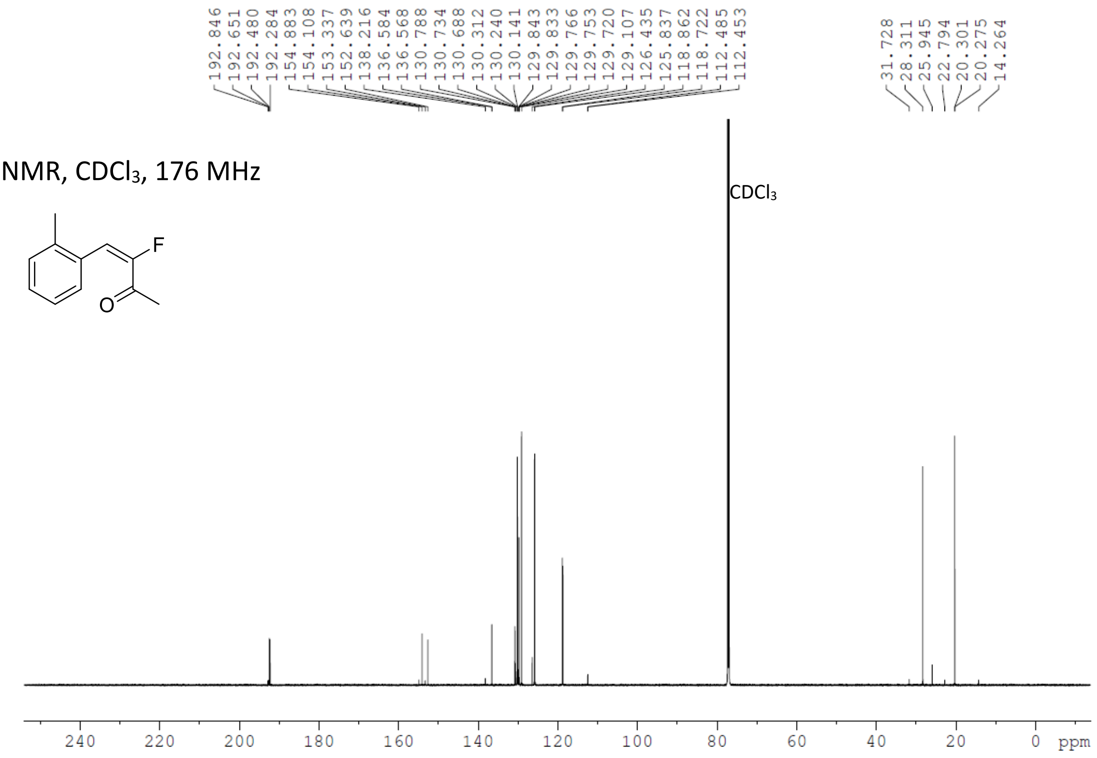


$^{19}$F NMR, $CDCl_3$, 659 MHz

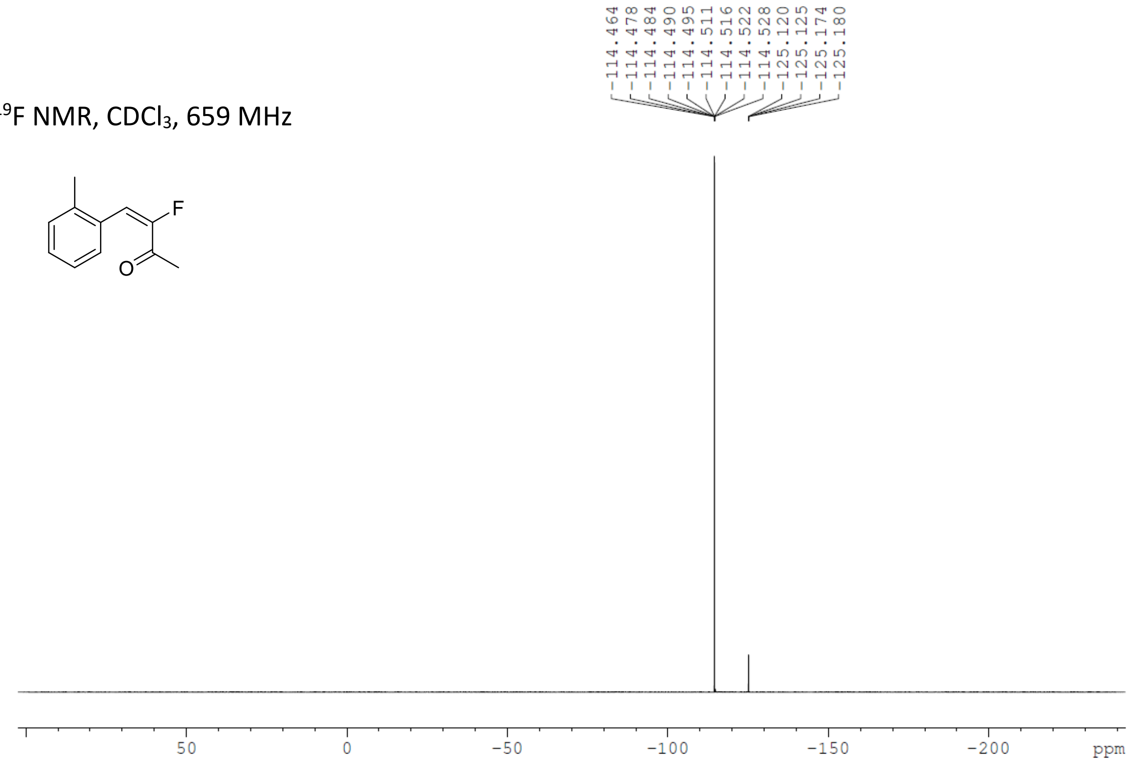

## (*Z*)-3-Fluoro-4-(3-trifluoromethylphenyl)-3-buten-2-one ((*Z*)-1f)

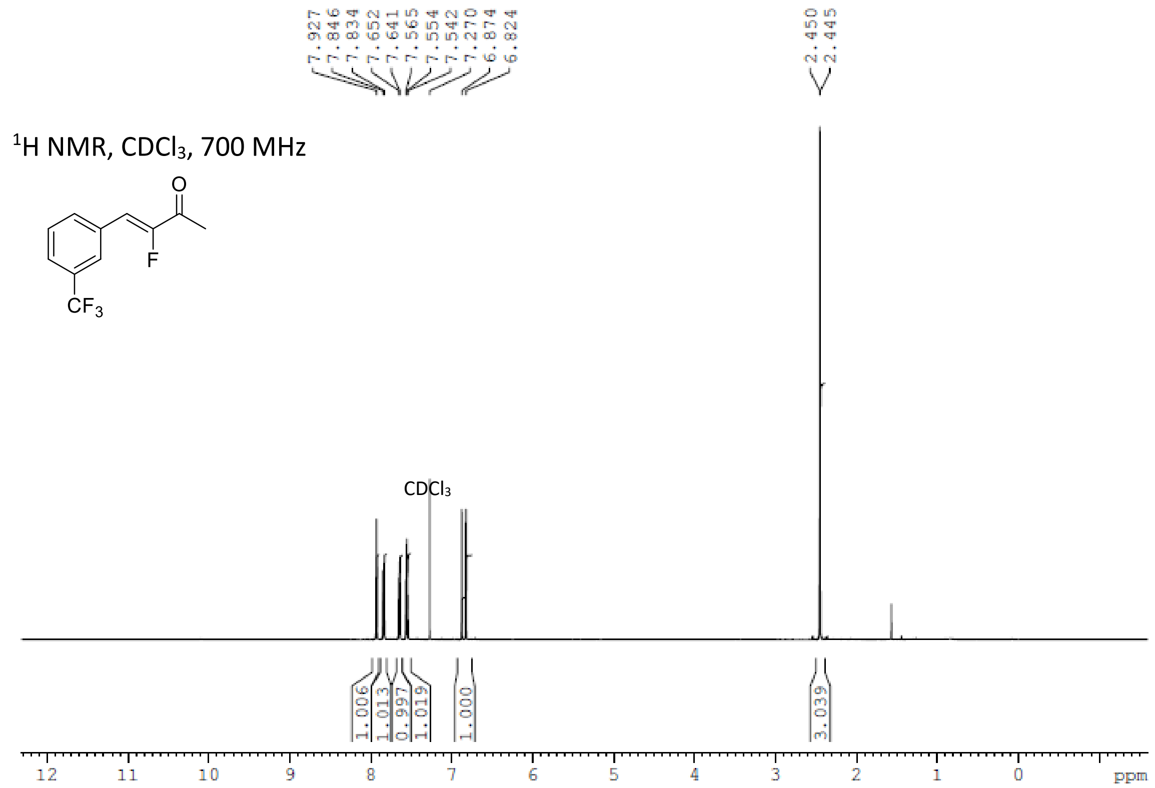


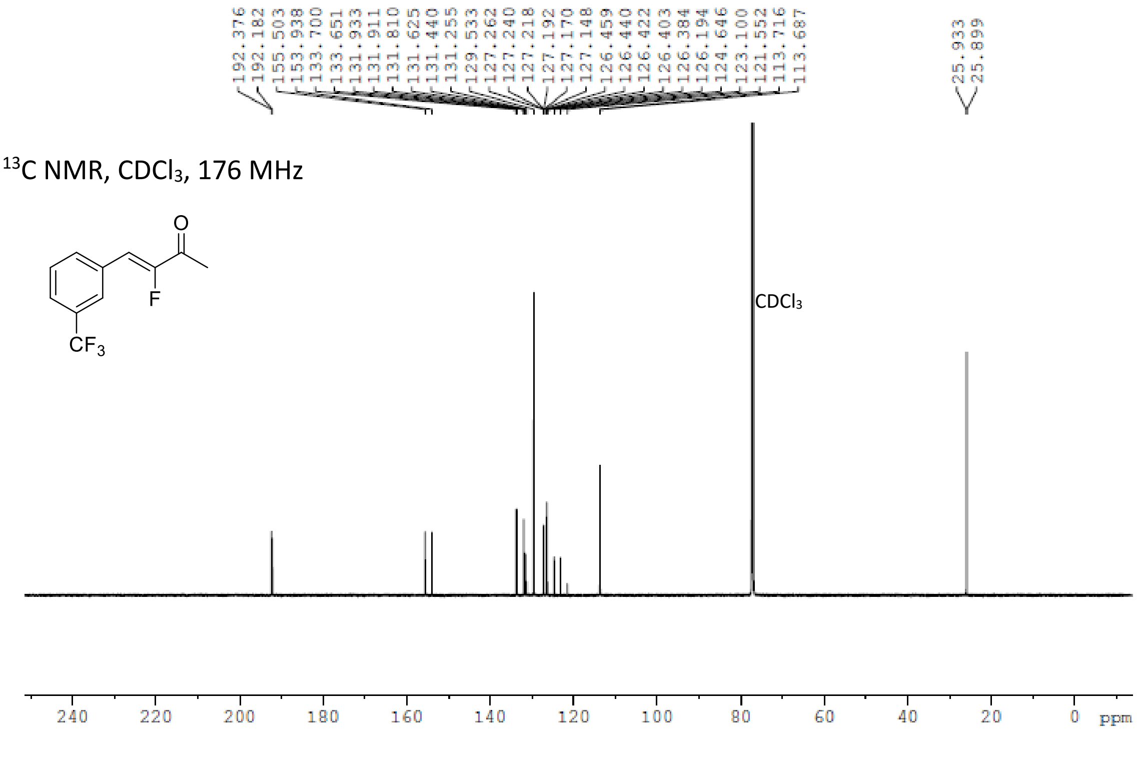

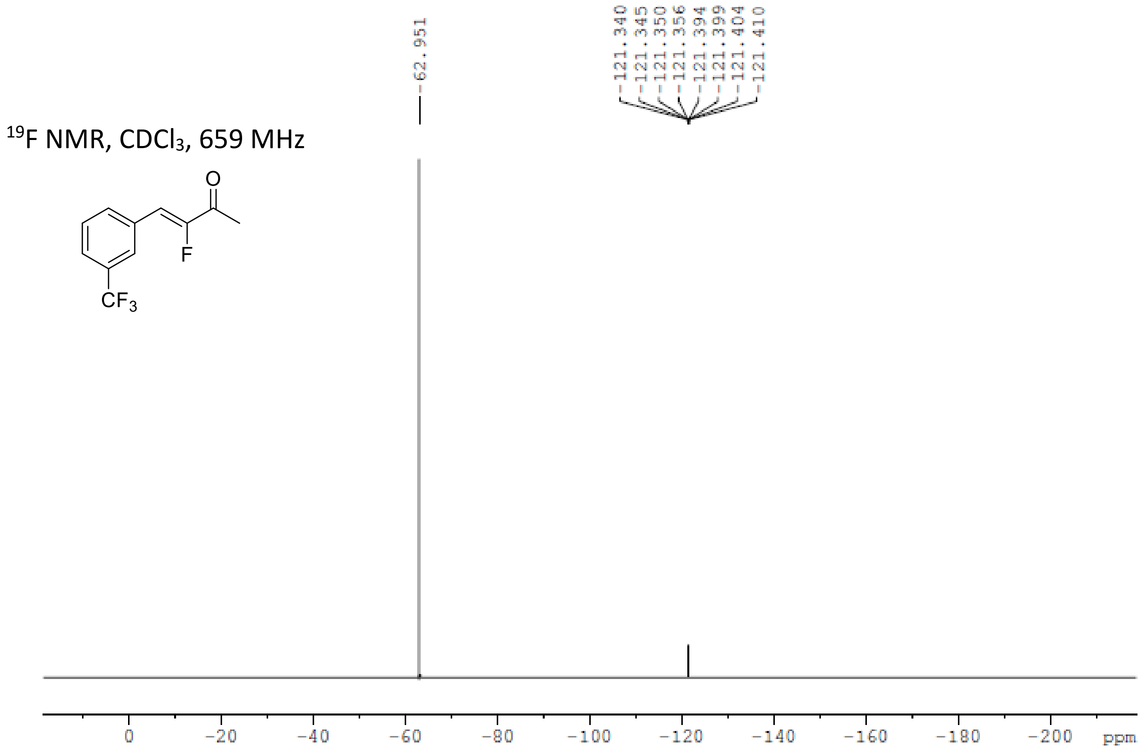


## (*E*)-3-Fluoro-4-(3-trifluoromethylphenyl)-3-buten-2-one ((*E*)-1f)

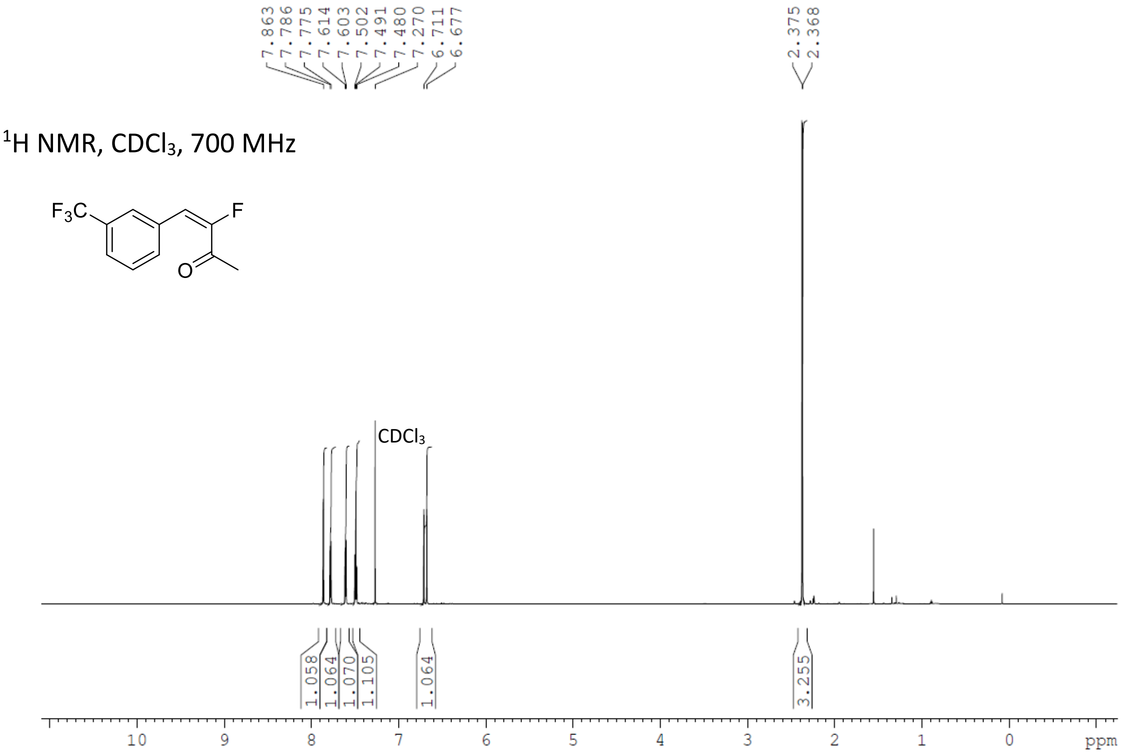

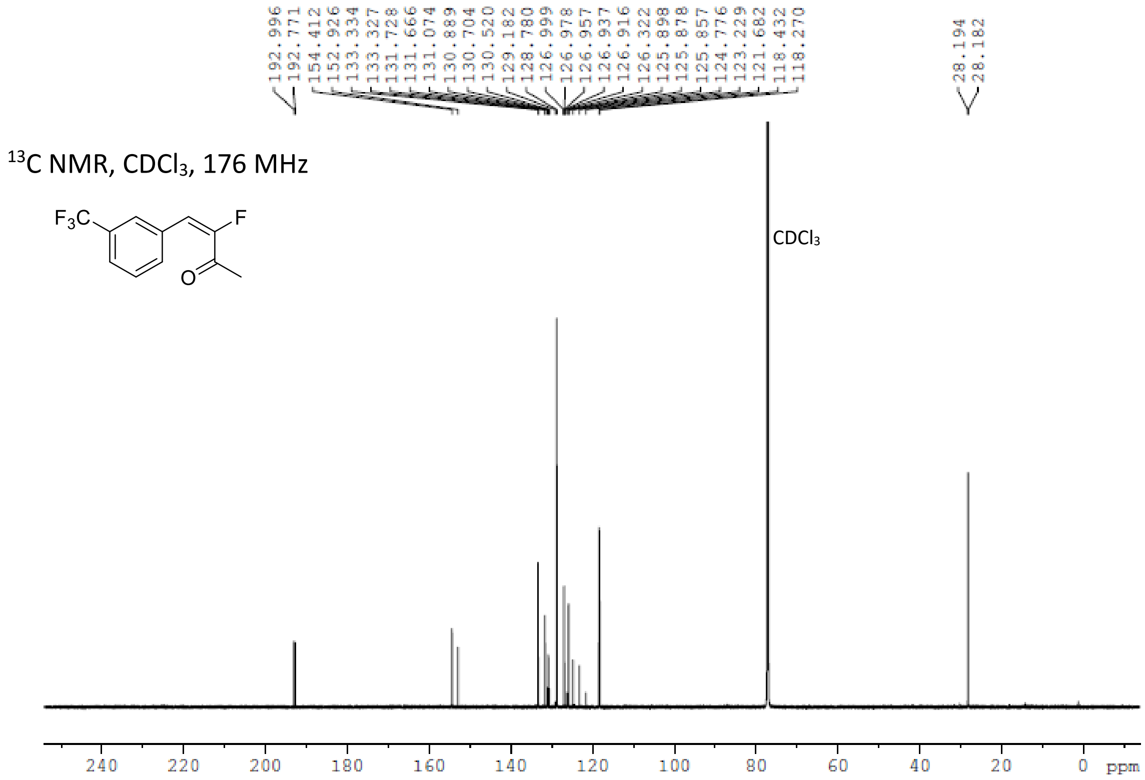
13C NMR, CDCl3, 176 MHz
F3C
F
O
CDCl3
192.996
192.771
154.412
152.926
133.334
133.327
131.728
131.666
131.074
130.889
130.704
130.520
129.182
128.780
126.999
126.978
126.957
126.937
126.916
126.322
125.898
125.878
125.857
124.776
123.229
121.682
118.432
118.270
28.194
28.182
240
220
200
180
160
140
120
100
80
60
40
20
0
ppm

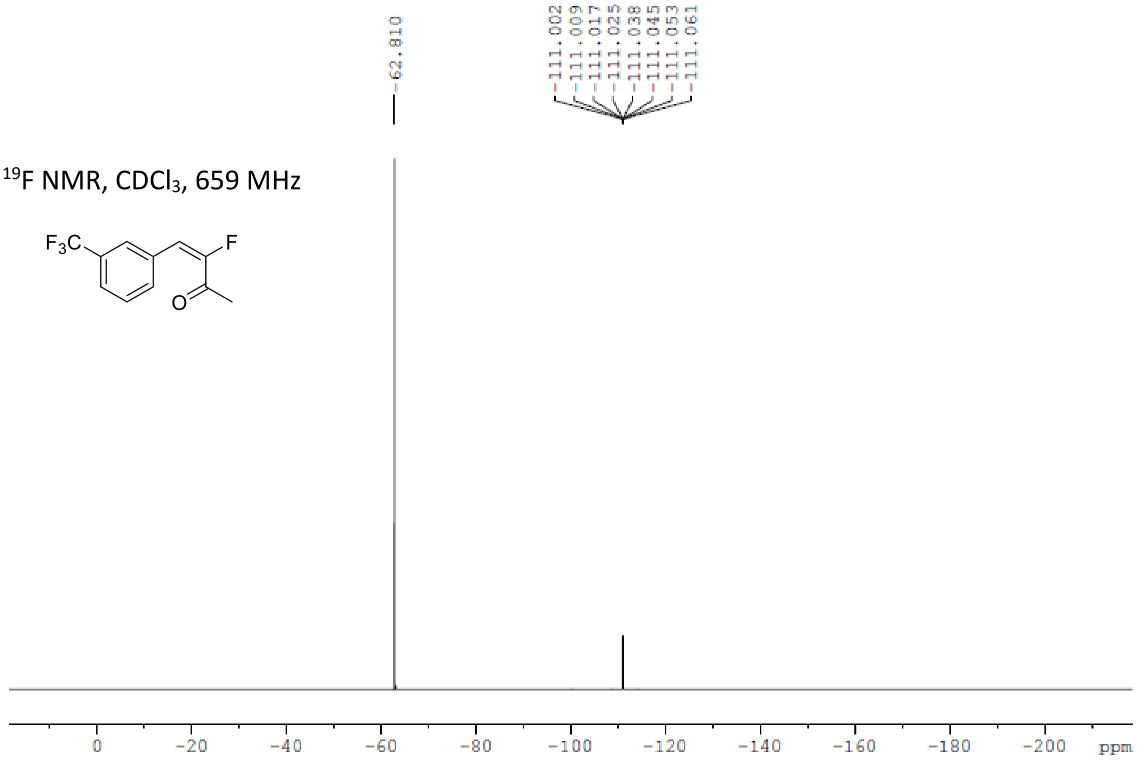
19F NMR, CDCl3, 659 MHz
F3C
F
O
-62.810
-111.002
-111.009
-111.017
-111.025
-111.038
-111.045
-111.053
-111.061
0
-20
-40
-60
-80
-100
-120
-140
-160
-180
-200
ppm

## ($Z$)-3-Fluoro-4(pyridine-3-yl)but-3-en-one (($Z$)-2a)

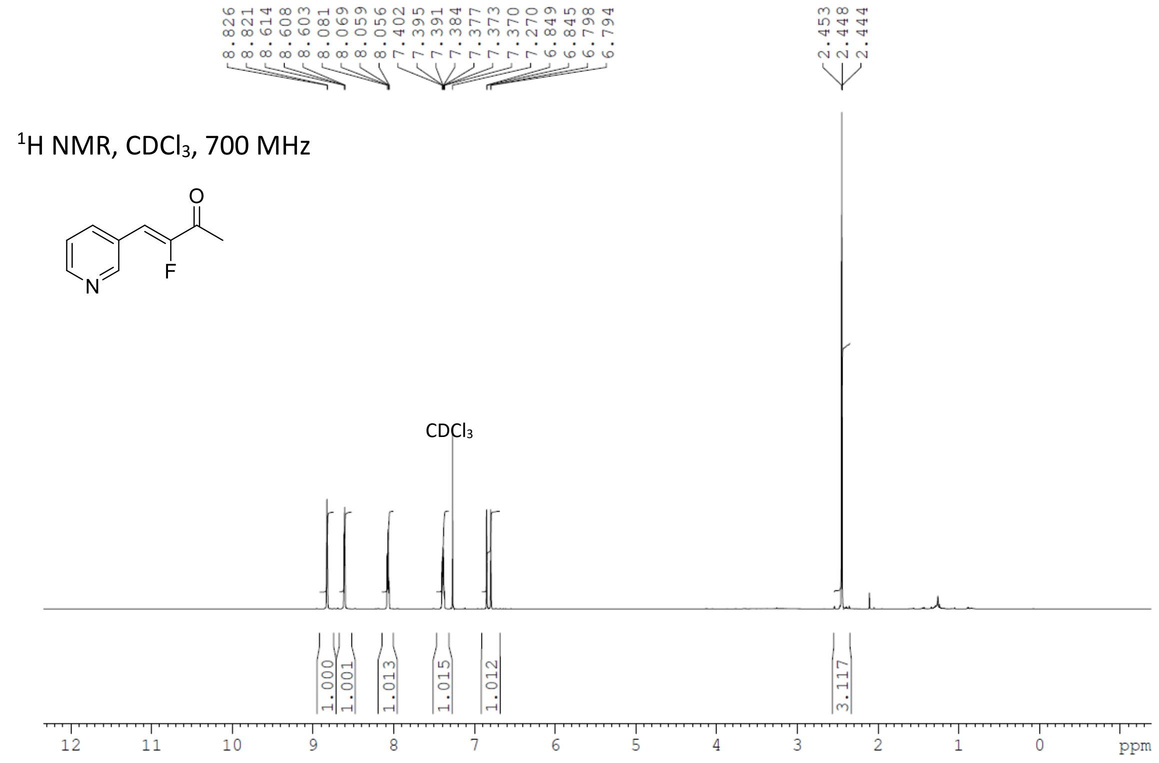


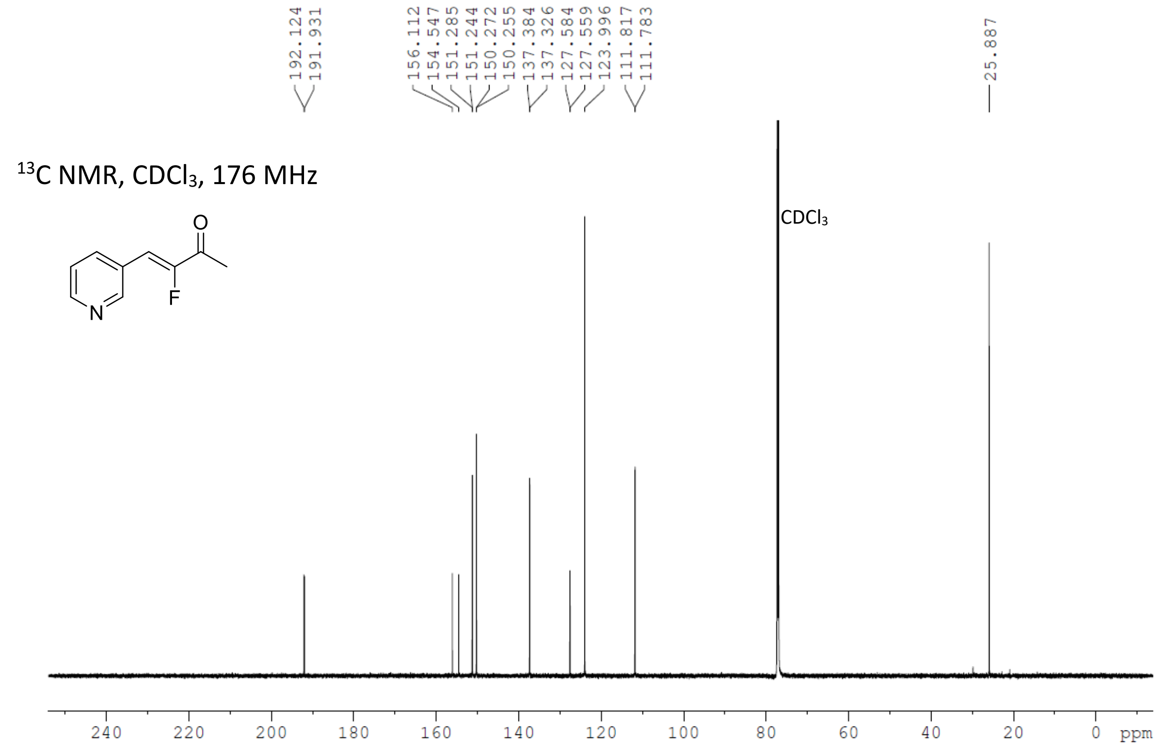

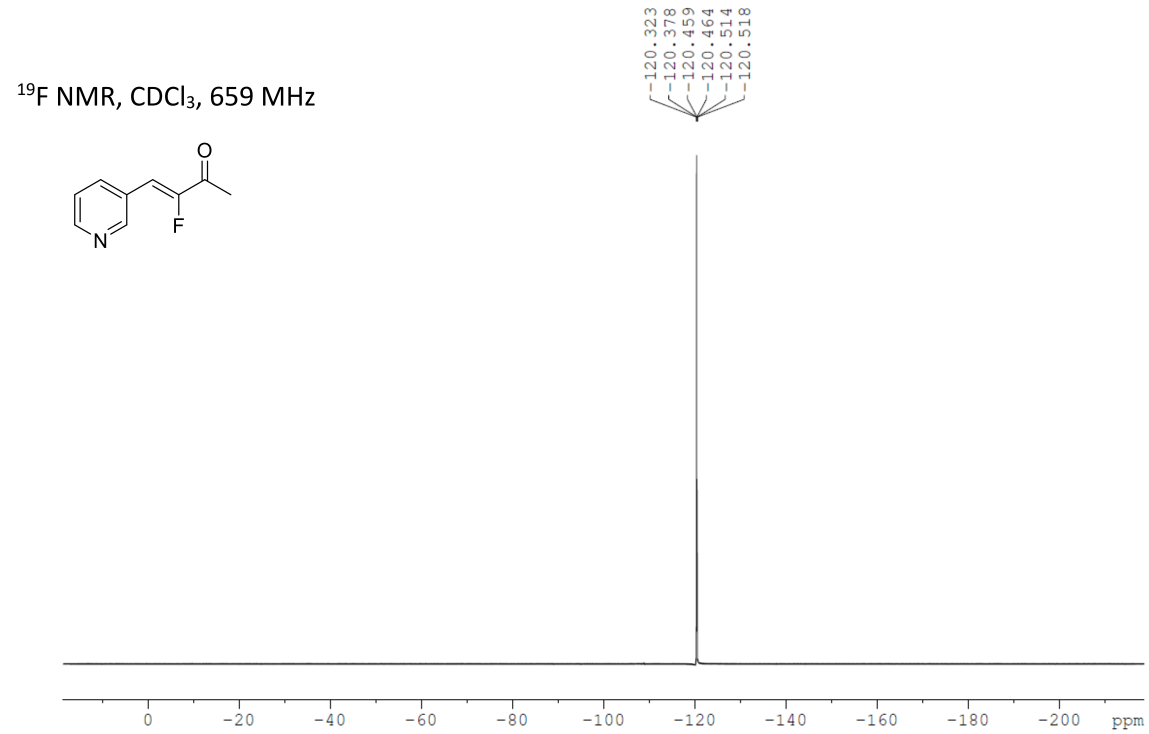


## (*E*)-3-Fluoro-4-(pyridin-3-yl)but-3-en-2-one ((*E*)-2a)

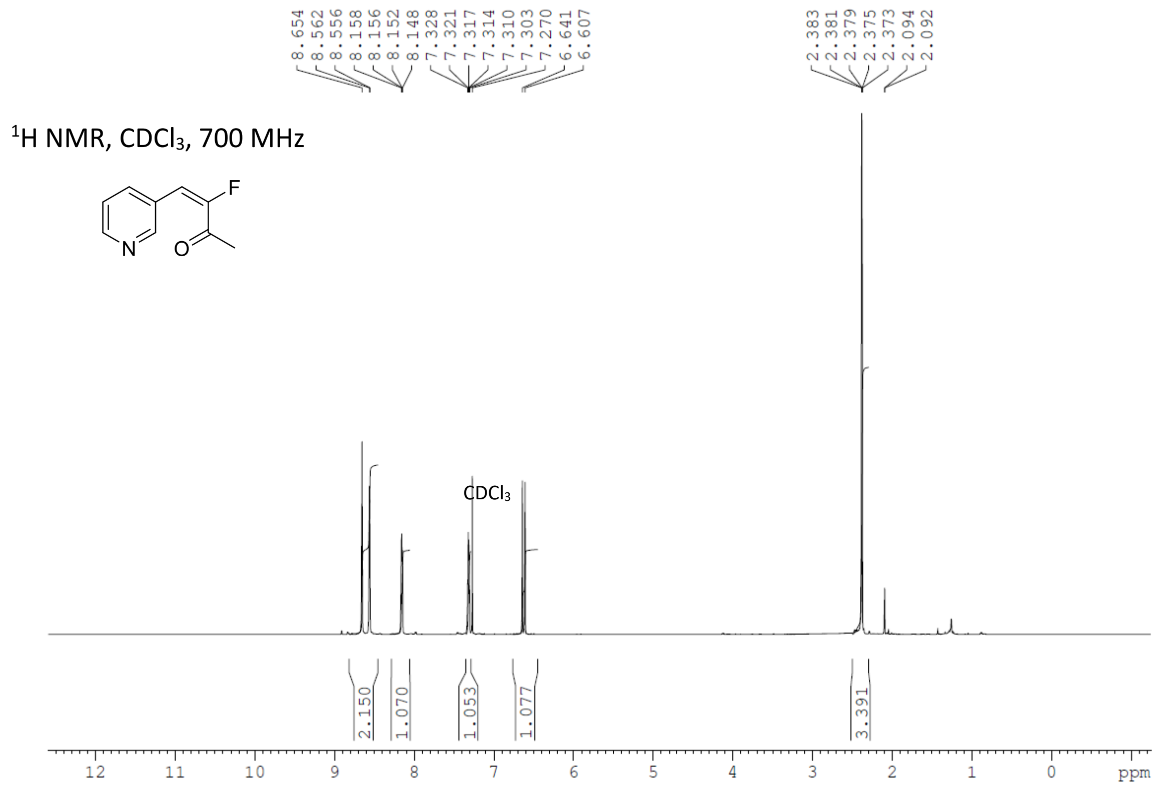

$^{13}C$ NMR, $CDCl_3$, 176 MHz

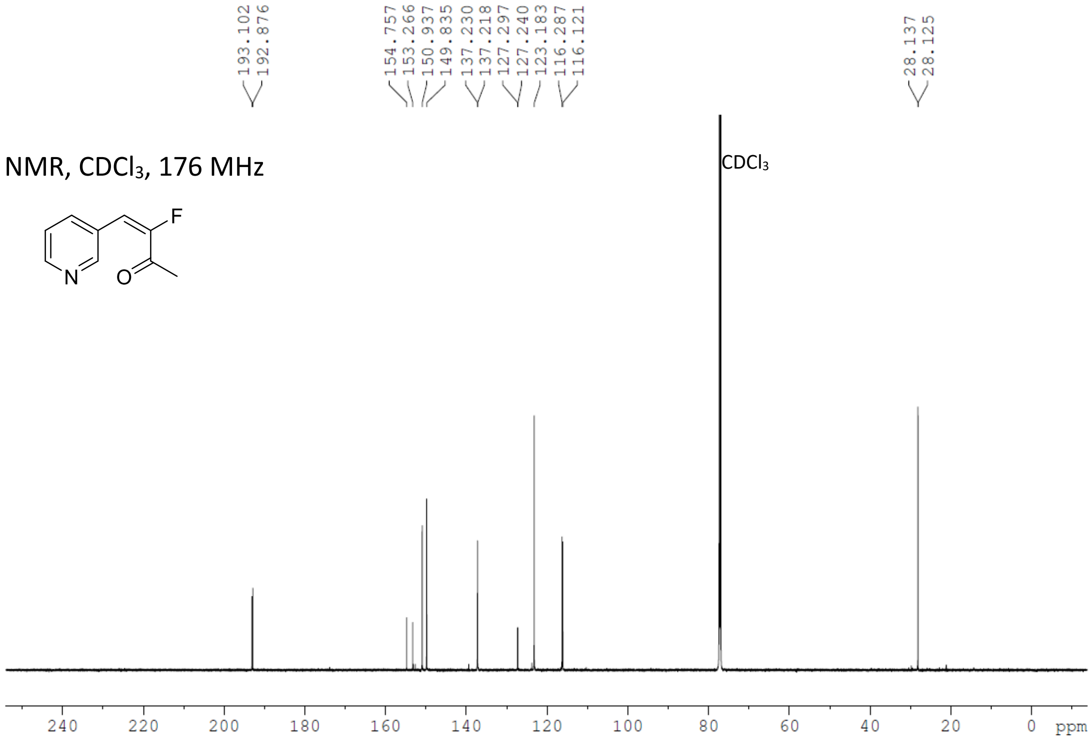


$^{19}F$ NMR, $CDCl_3$, 659 MHz

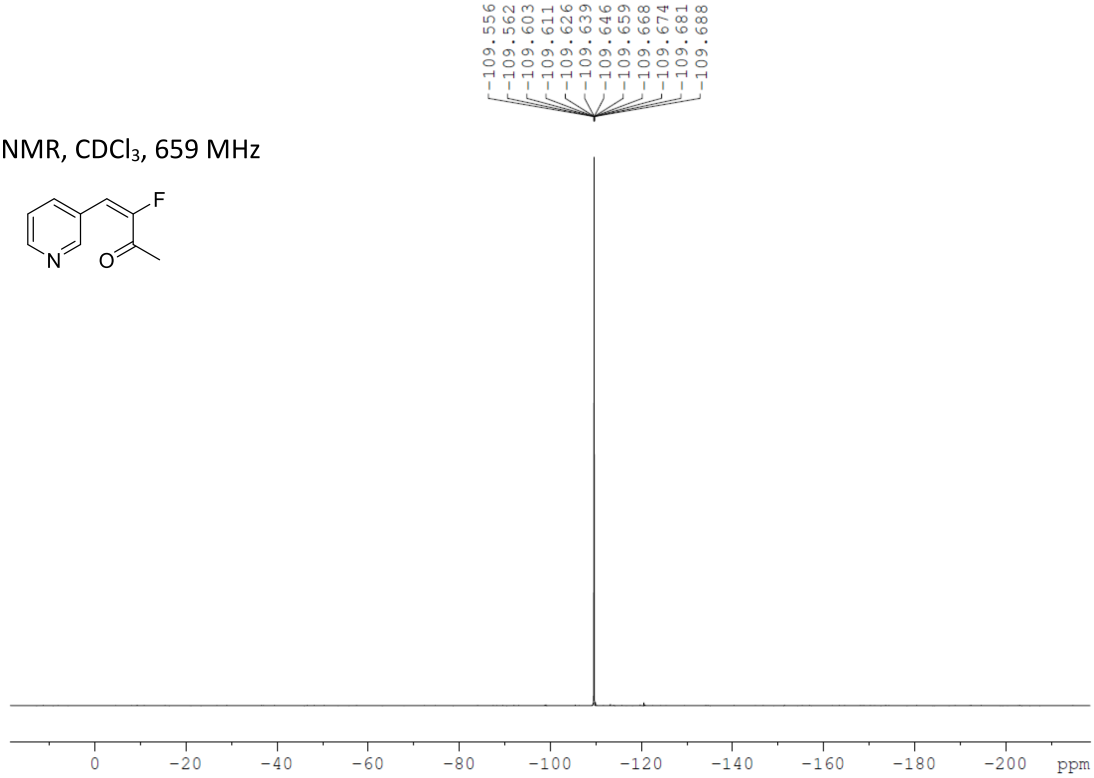

## ($E$)-3-Fluoro-4-(thiophen-2-yl)but-3-en-2-one (($E$)-2b)

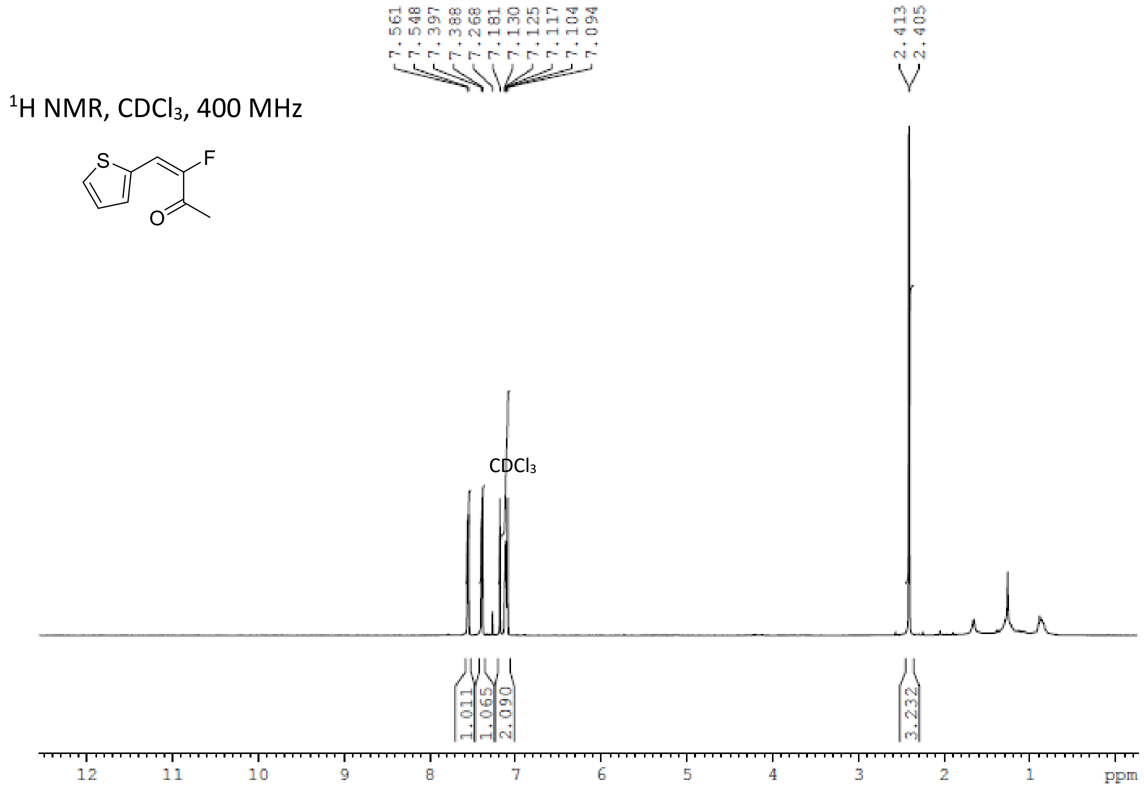


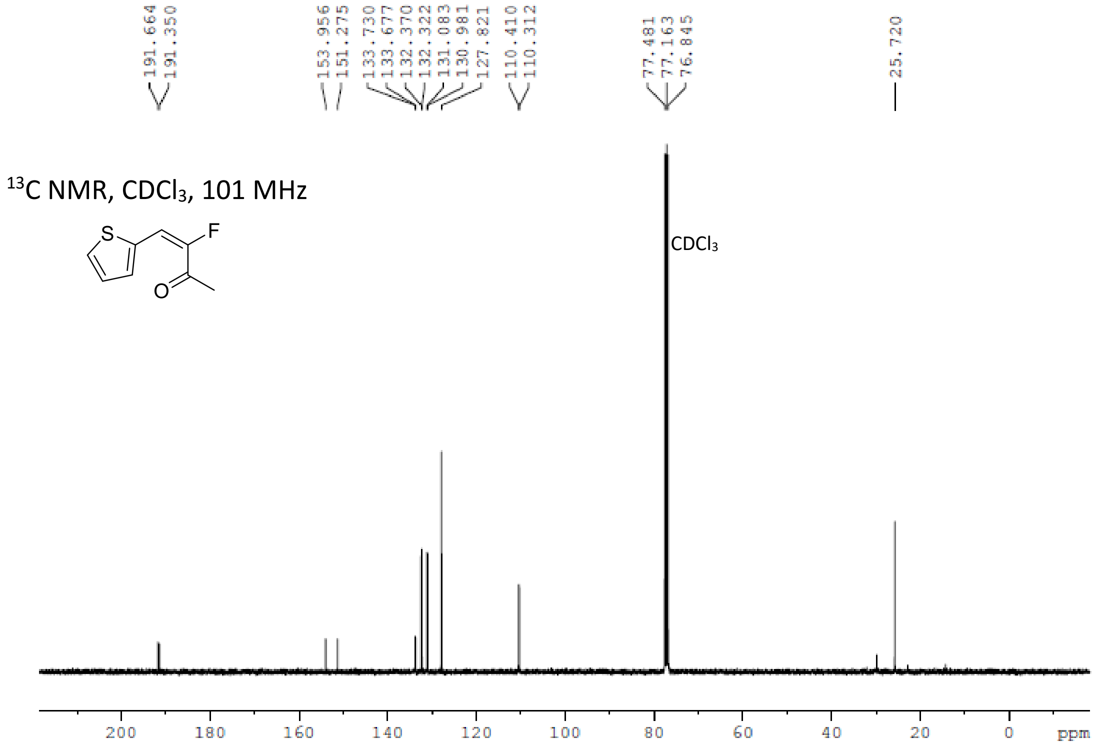

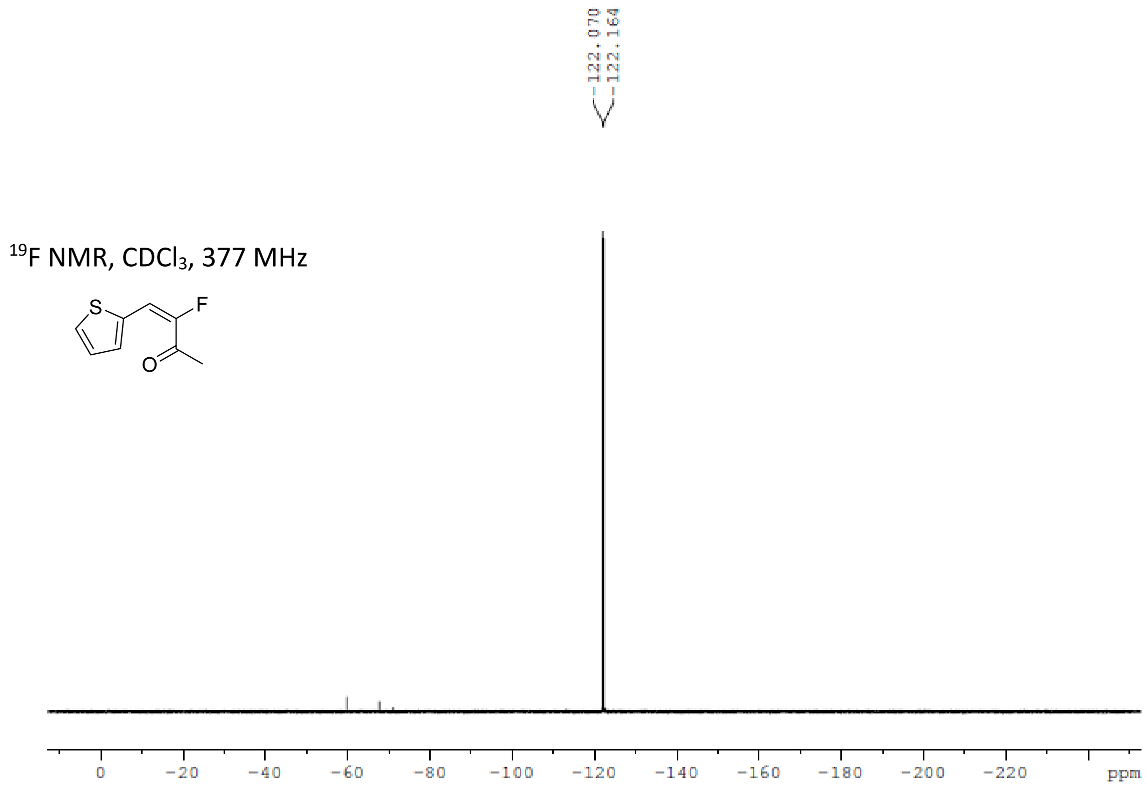


## (*Z*)-3-Fluoro-4-cyclohexylbut-3-en-2-one ((*Z*)-3a)

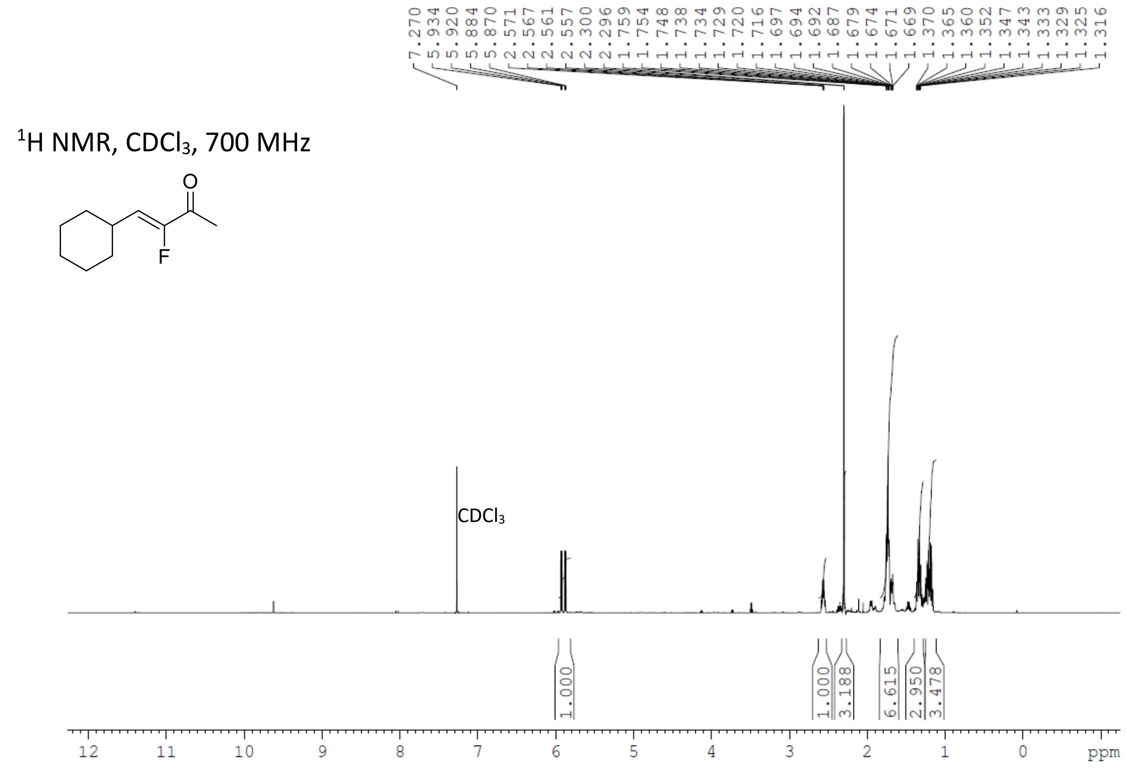

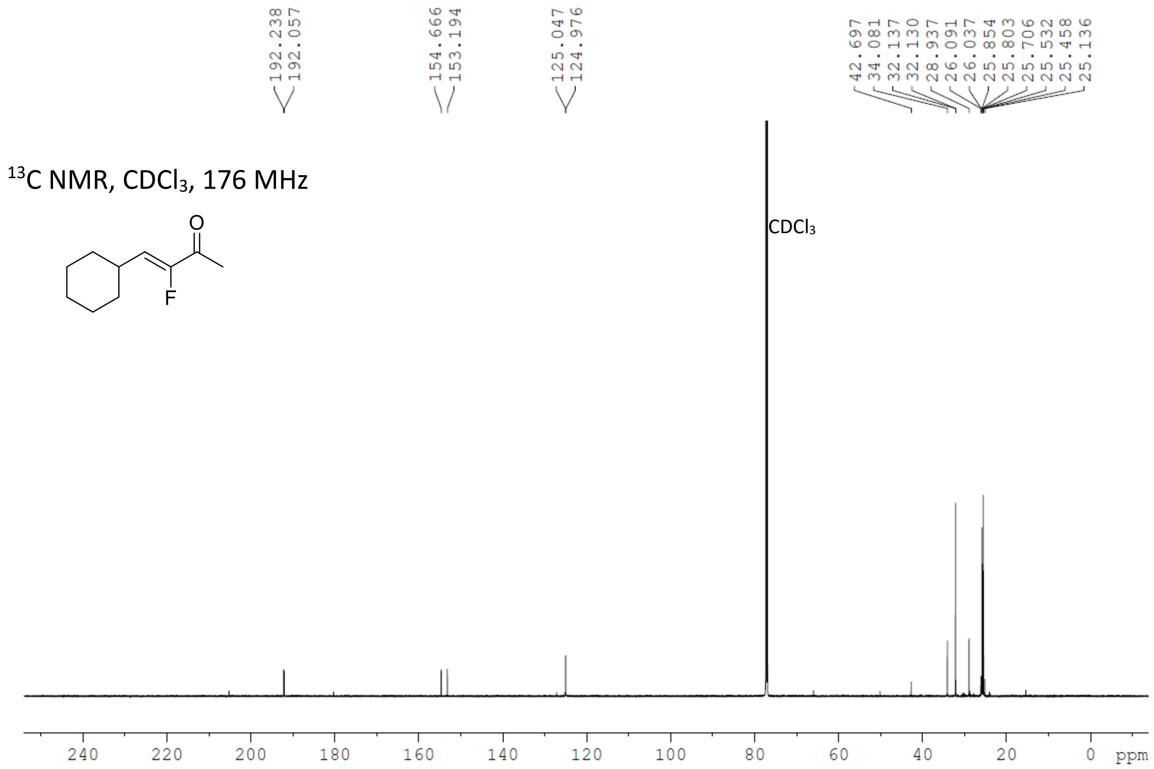
192.238
192.057
154.666
153.194
125.047
124.976
42.697
34.081
32.137
32.130
28.937
26.091
26.037
25.854
25.803
25.706
25.532
25.458
25.136
13C NMR, CDCl3, 176 MHz
O
F
CDCl3
240
220
200
180
160
140
120
100
80
60
40
20
0
ppm

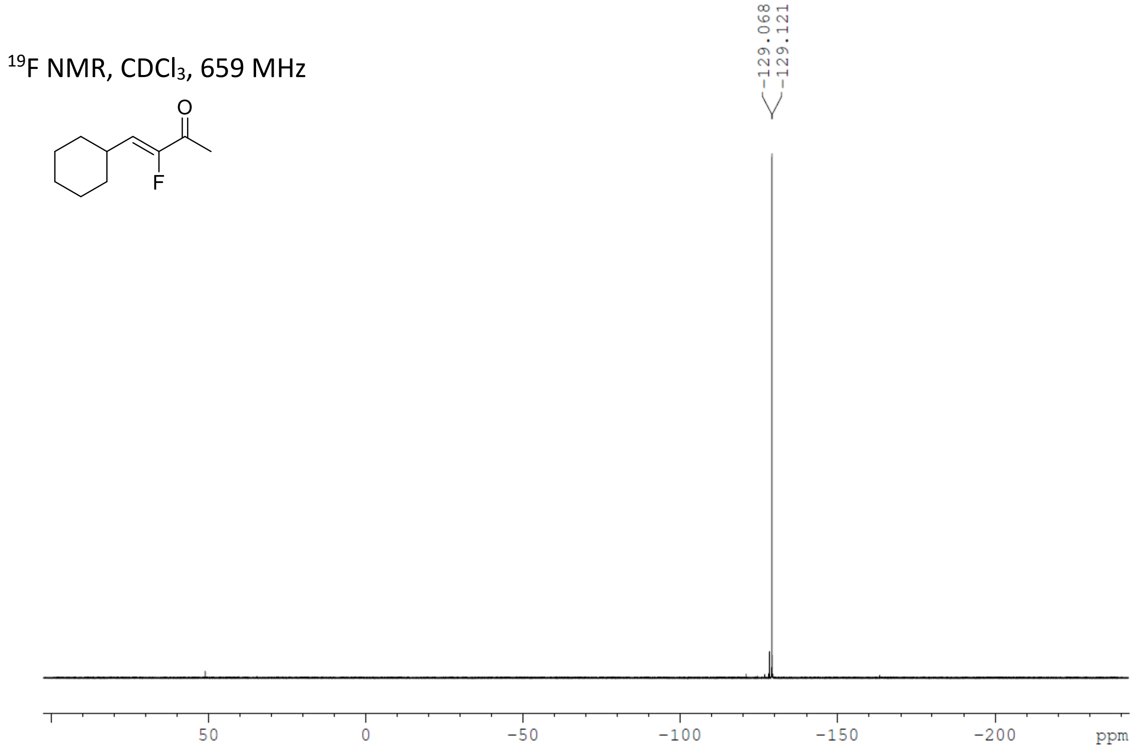
-129.068
-129.121
19F NMR, CDCl3, 659 MHz
O
F
50
0
-50
-100
-150
-200
ppm

(*E*)-3- Fluoro-4-cyclohexylbut-3-en-2-one ((*E*)-3a)

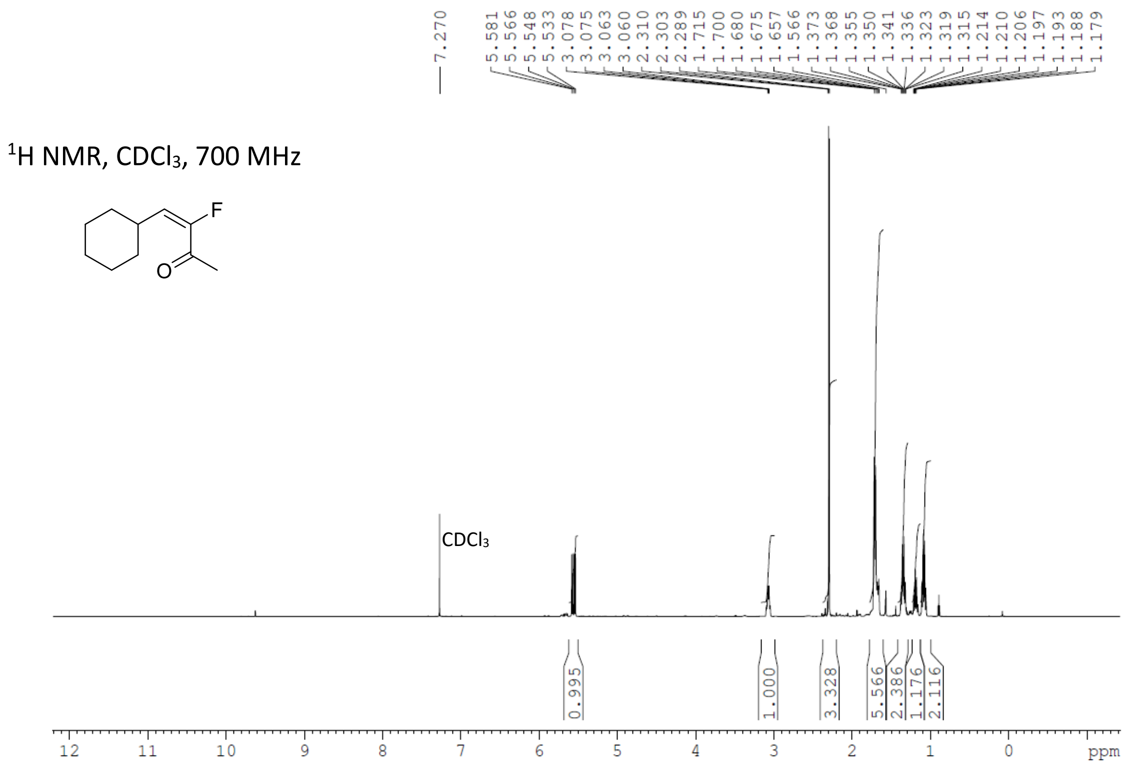


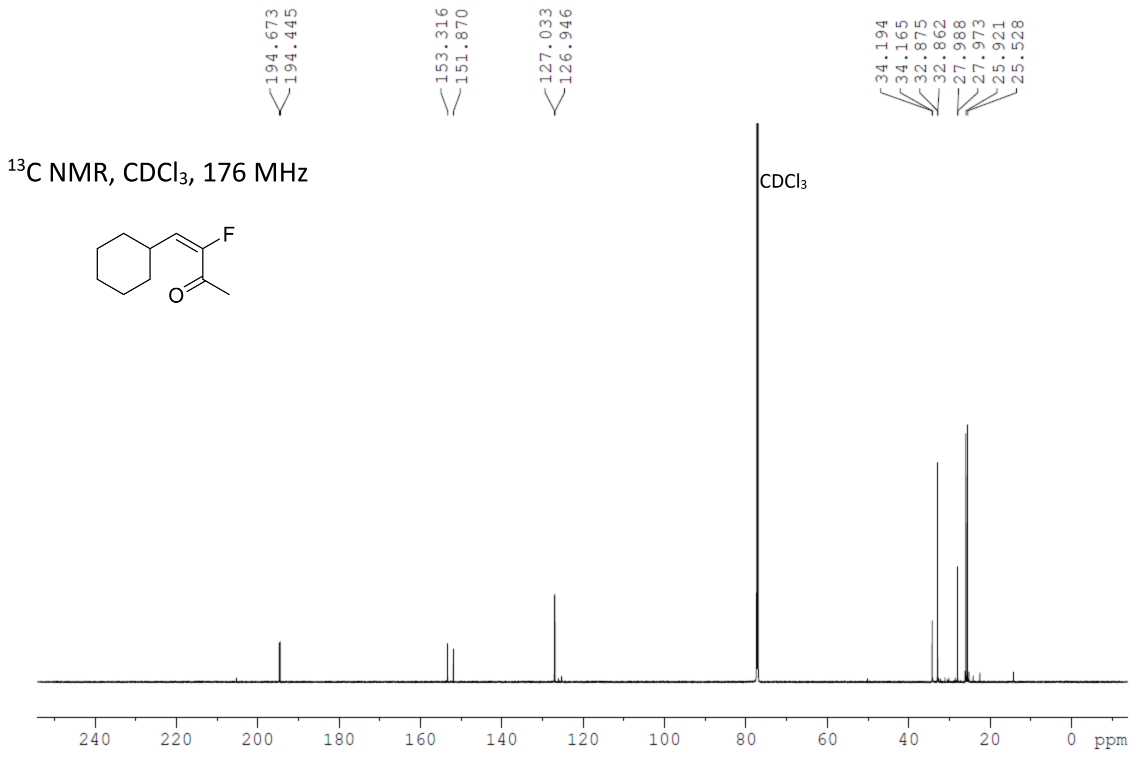

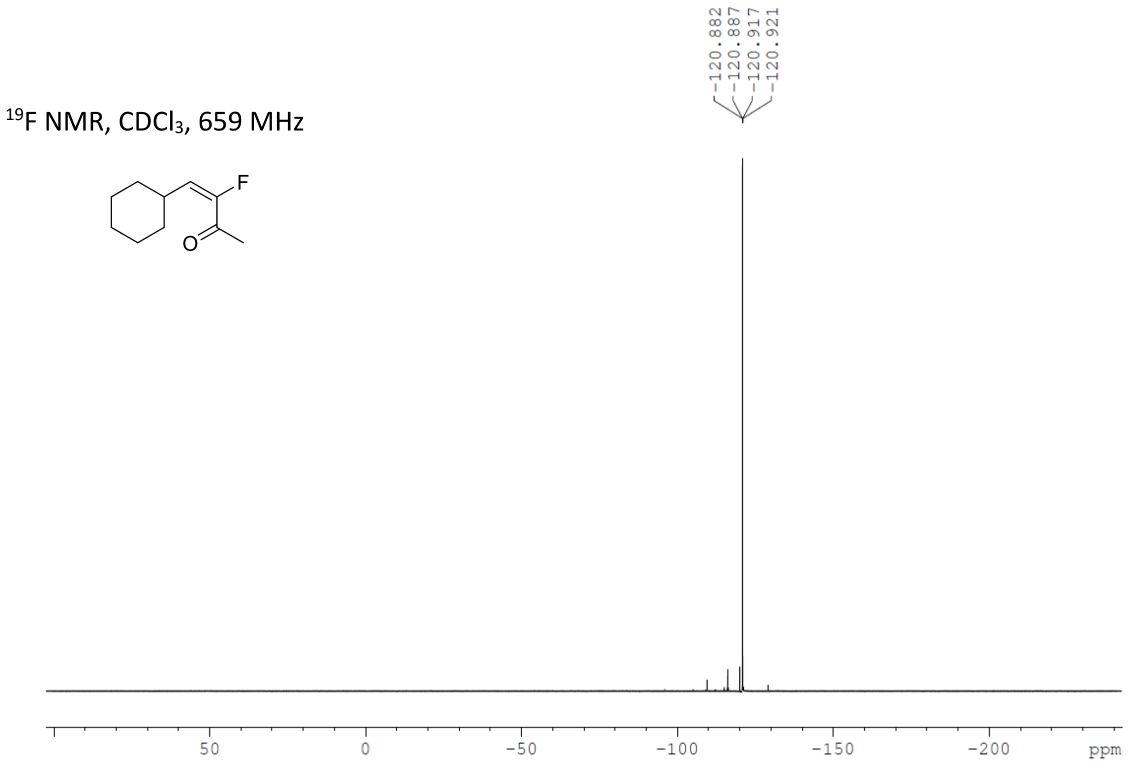


## (*Z*)-3-Fluorooct-3-en-2-one ((*Z*)-3b)

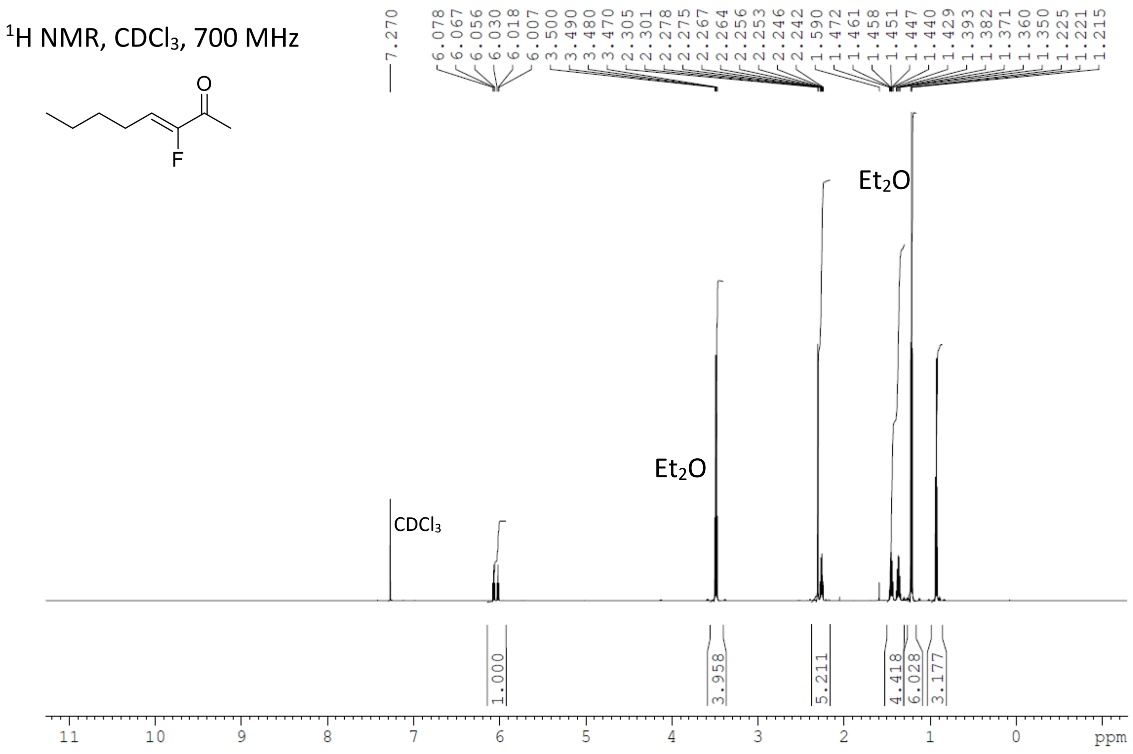

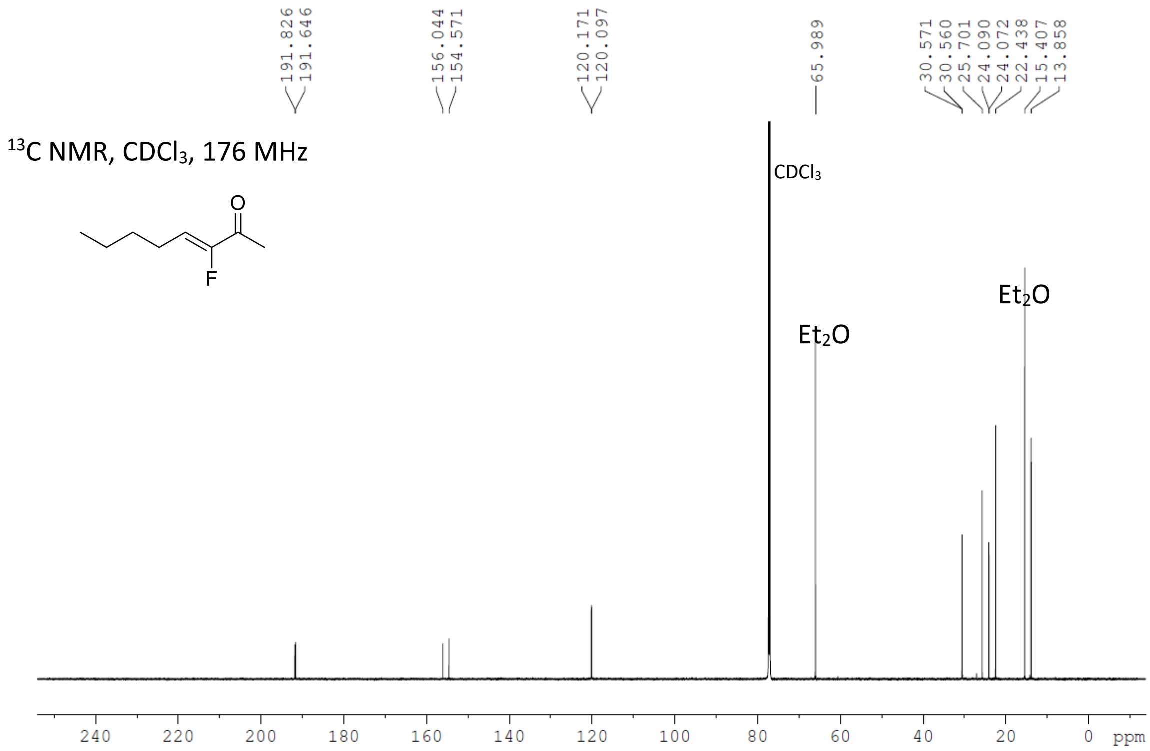

$^{13}$C NMR, $CDCl_3$, 176 MHz
O
F
191.826
191.646
156.044
154.571
120.171
120.097
65.989
30.571
30.560
25.701
24.090
24.072
22.438
15.407
13.858
$CDCl_3$
$Et_2O$
$Et_2O$
240 220 200 180 160 140 120 100 80 60 40 20 0 ppm


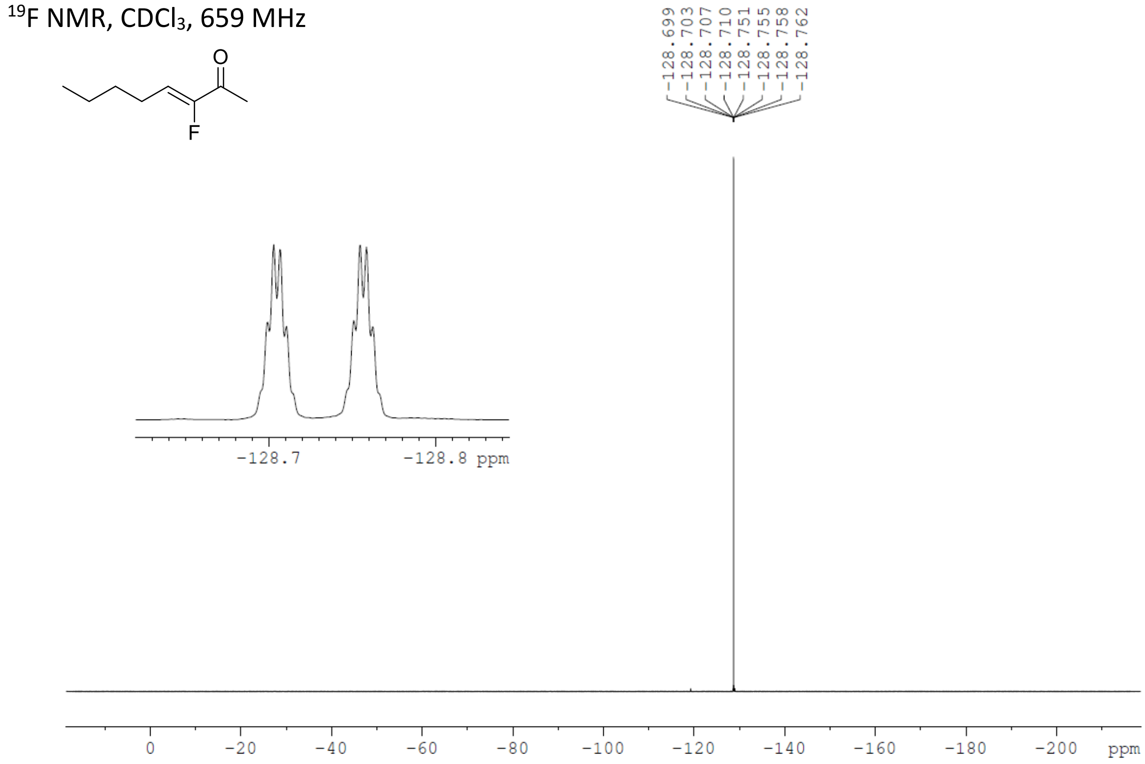

$^{19}$F NMR, $CDCl_3$, 659 MHz
O
F
-128.699
-128.703
-128.707
-128.710
-128.751
-128.755
-128.758
-128.762
-128.7
-128.8 ppm
0 -20 -40 -60 -80 -100 -120 -140 -160 -180 -200 ppm

## (*E*)-3-Fluorooct-3-en-2-one ((*E*)-3b)

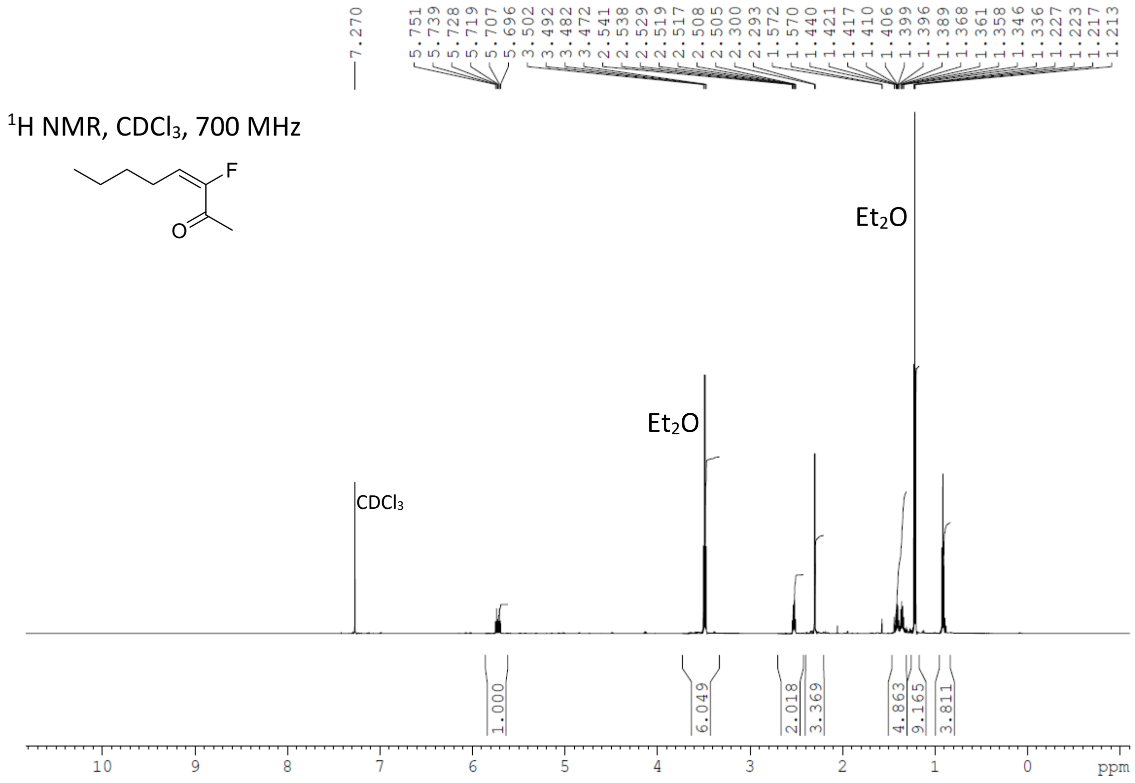


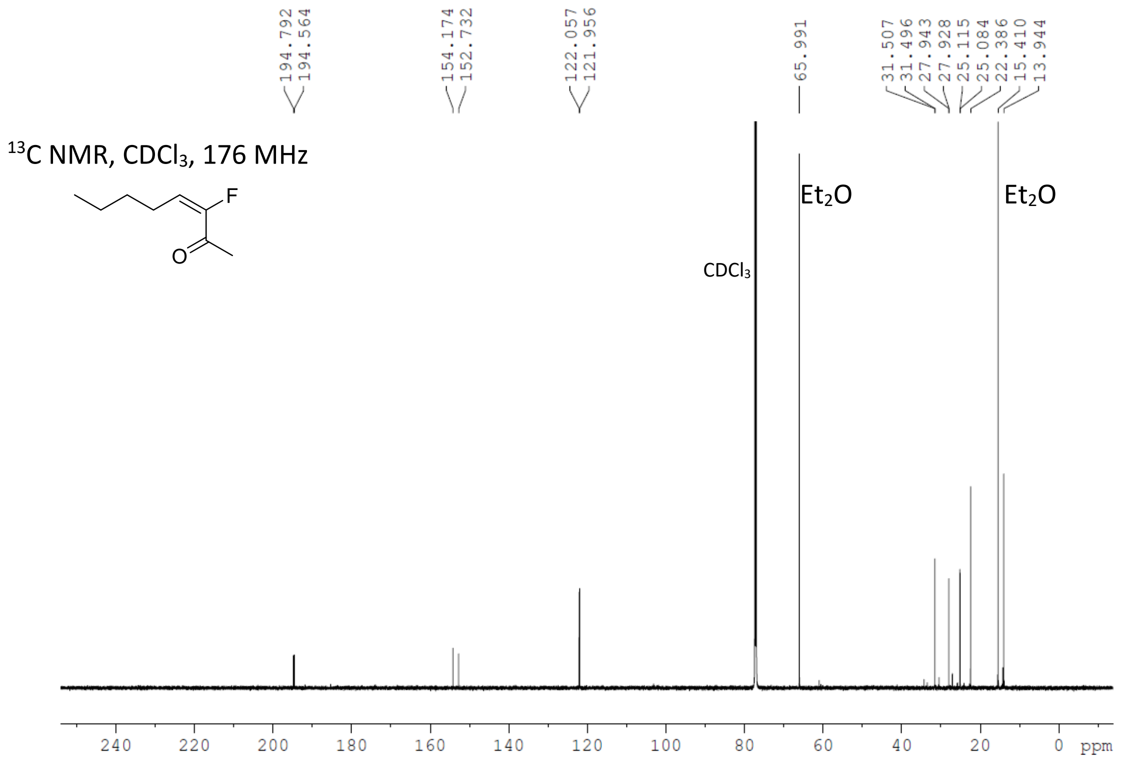

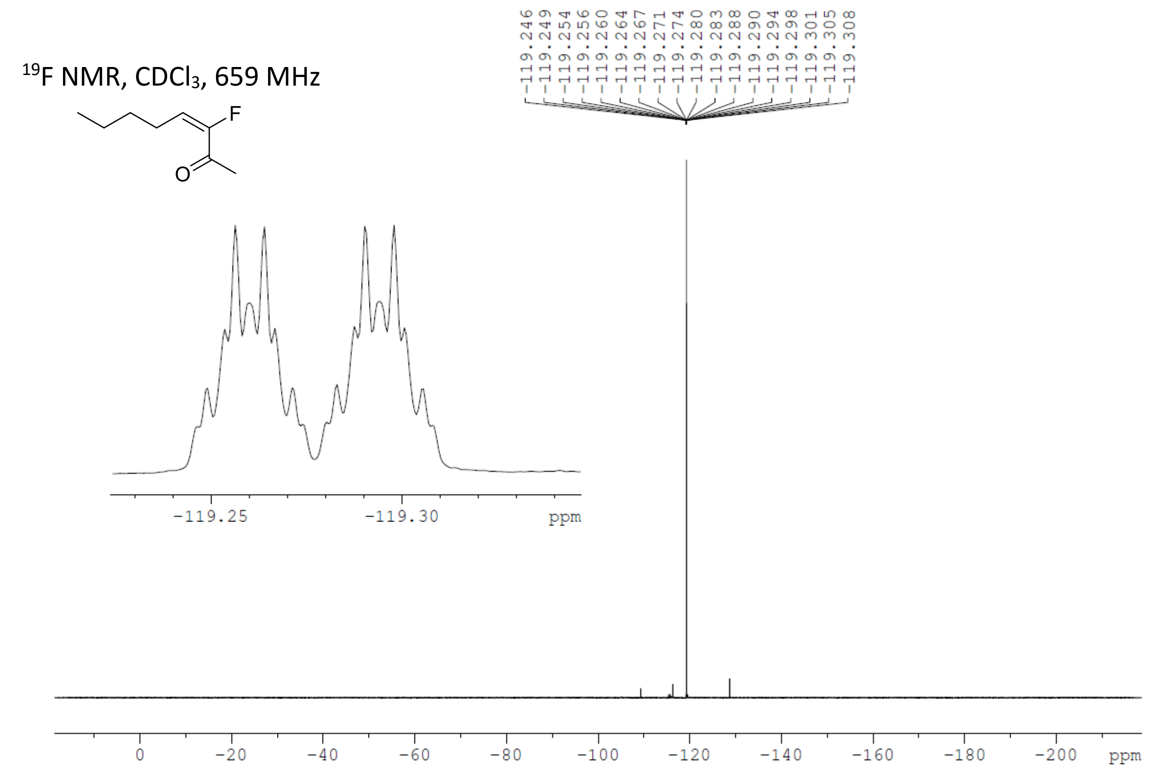


## (*E*)-3-Fluoro-5-phenylpent-3-en-2-one ((*E*)-3c)

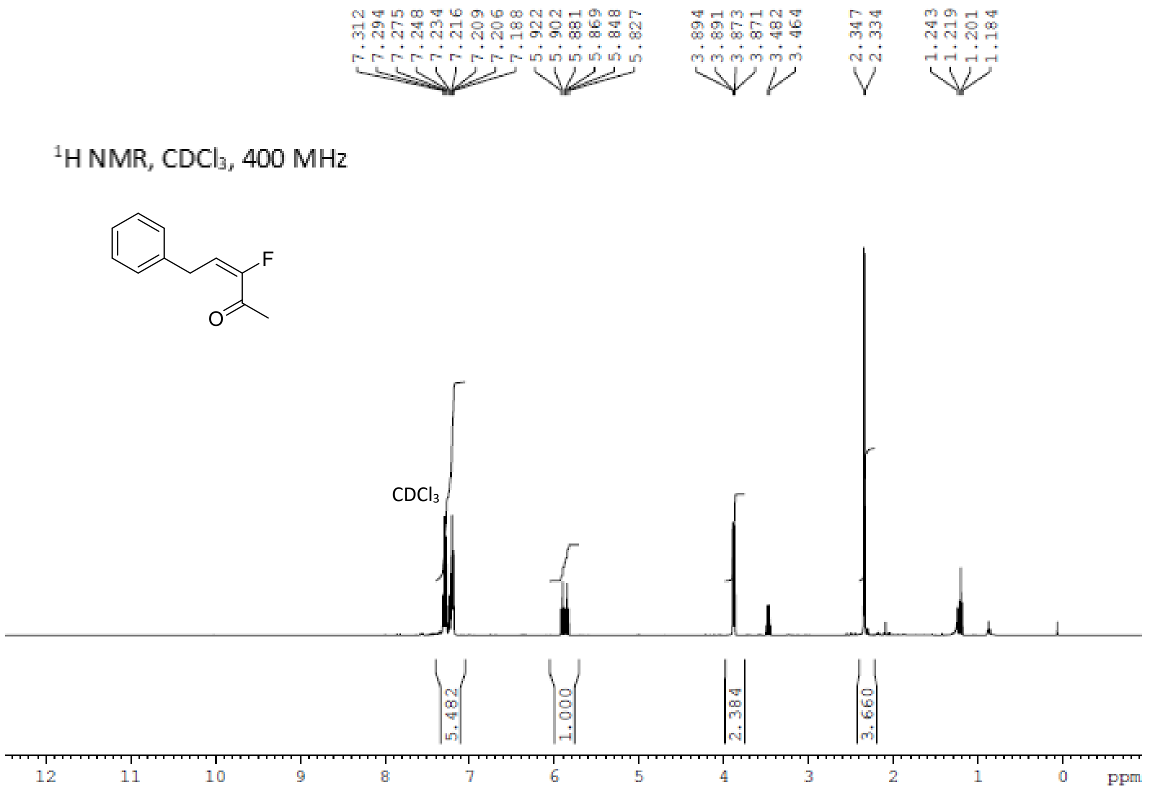

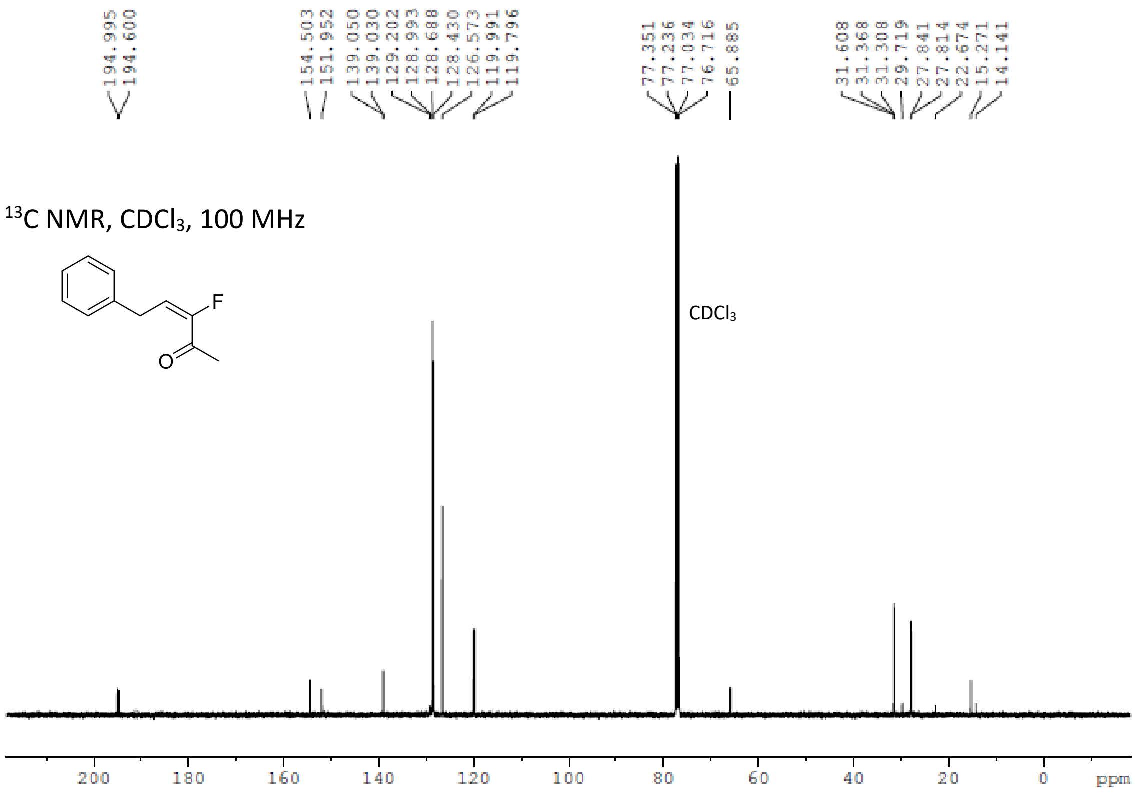
194.995
194.600
154.503
151.952
139.050
139.030
129.202
128.993
128.688
128.430
126.573
119.991
119.796
77.351
77.236
77.034
76.716
65.885
31.608
31.368
31.308
29.719
27.841
27.814
22.674
15.271
14.141
13C NMR, CDCl3, 100 MHz
F
O
CDCl3
200
180
160
140
120
100
80
60
40
20
0
ppm

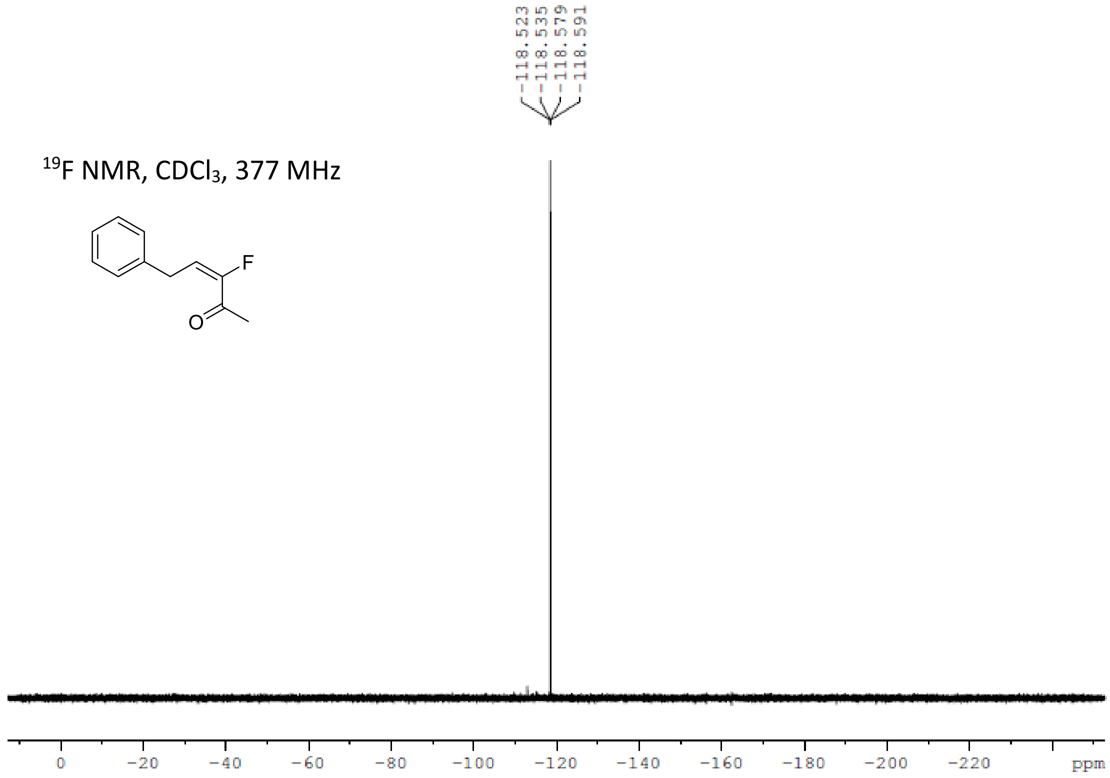
-118.523
-118.535
-118.579
-118.591
19F NMR, CDCl3, 377 MHz
F
O
0
-20
-40
-60
-80
-100
-120
-140
-160
-180
-200
-220
ppm

(*E*)- 3-Fluoro-6-phenylhex-3-en-2-one ((*E*)-3d)

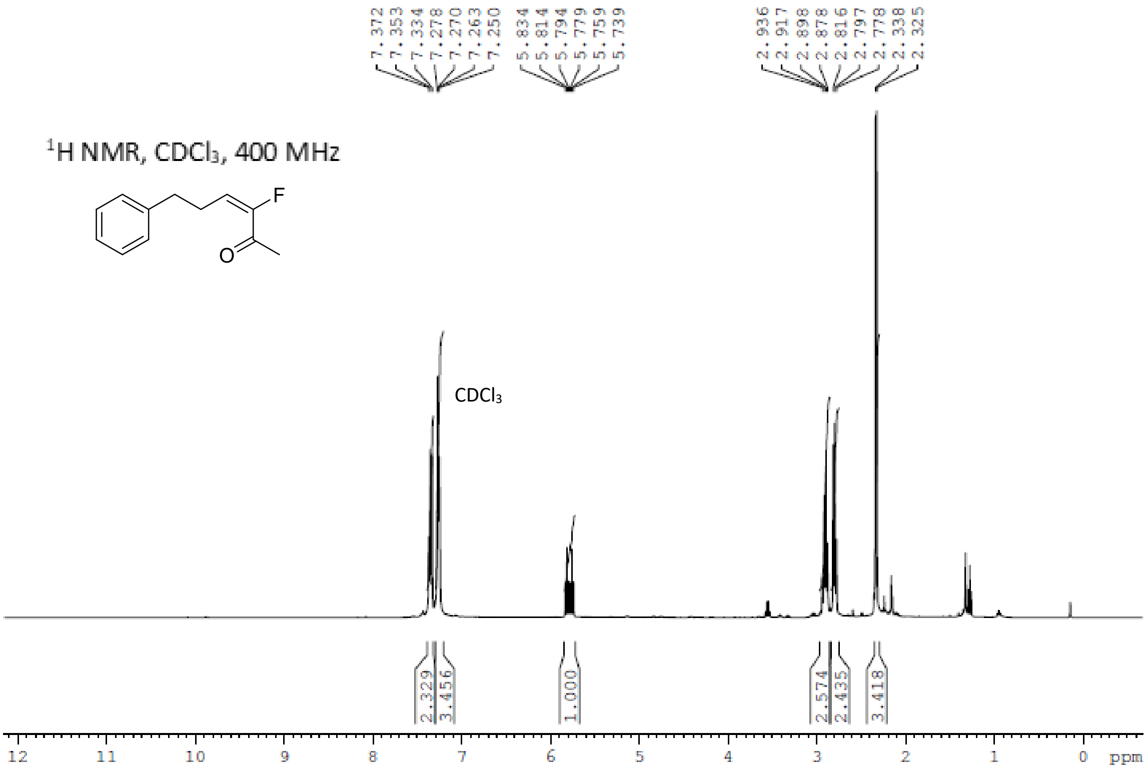


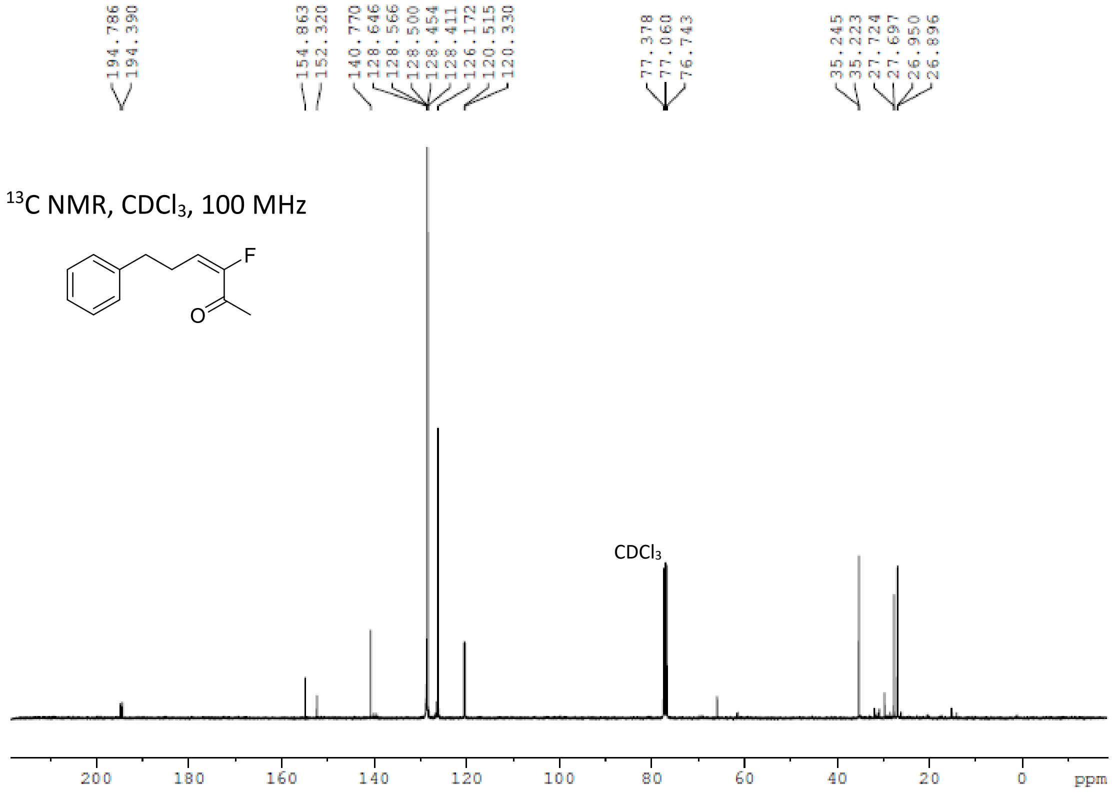

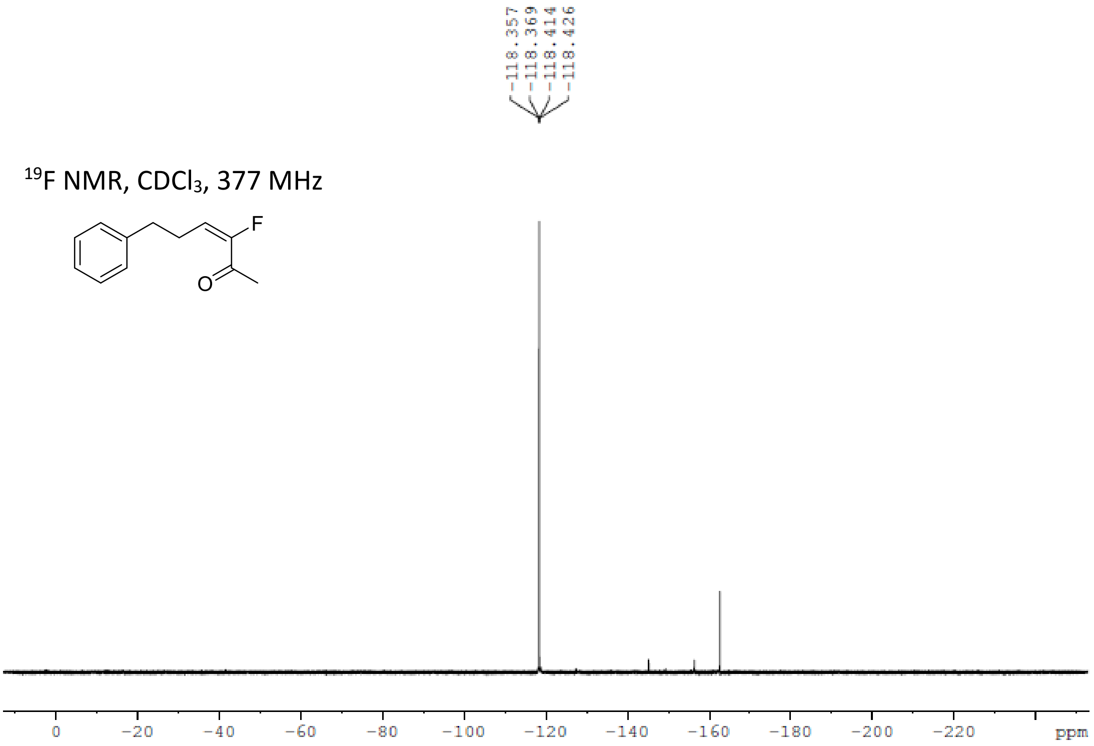


## (*E*)-2-Fluorocyclohex-2-en-1-one ((*E*)-4a)

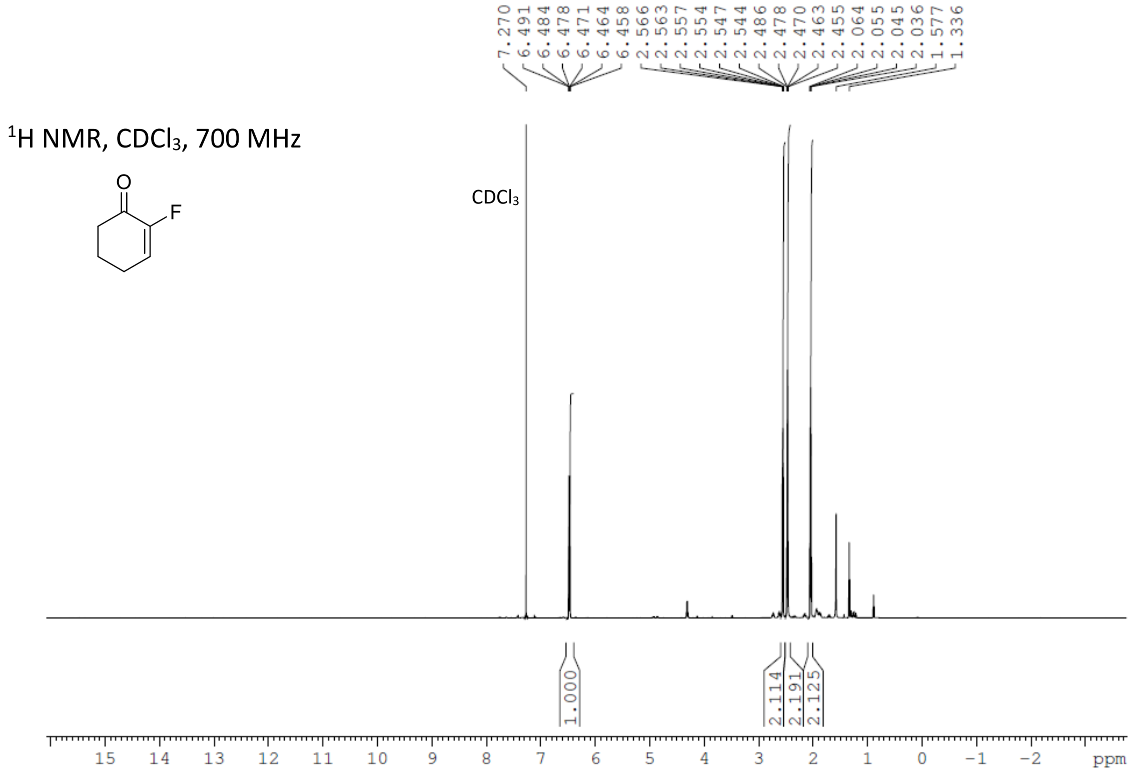

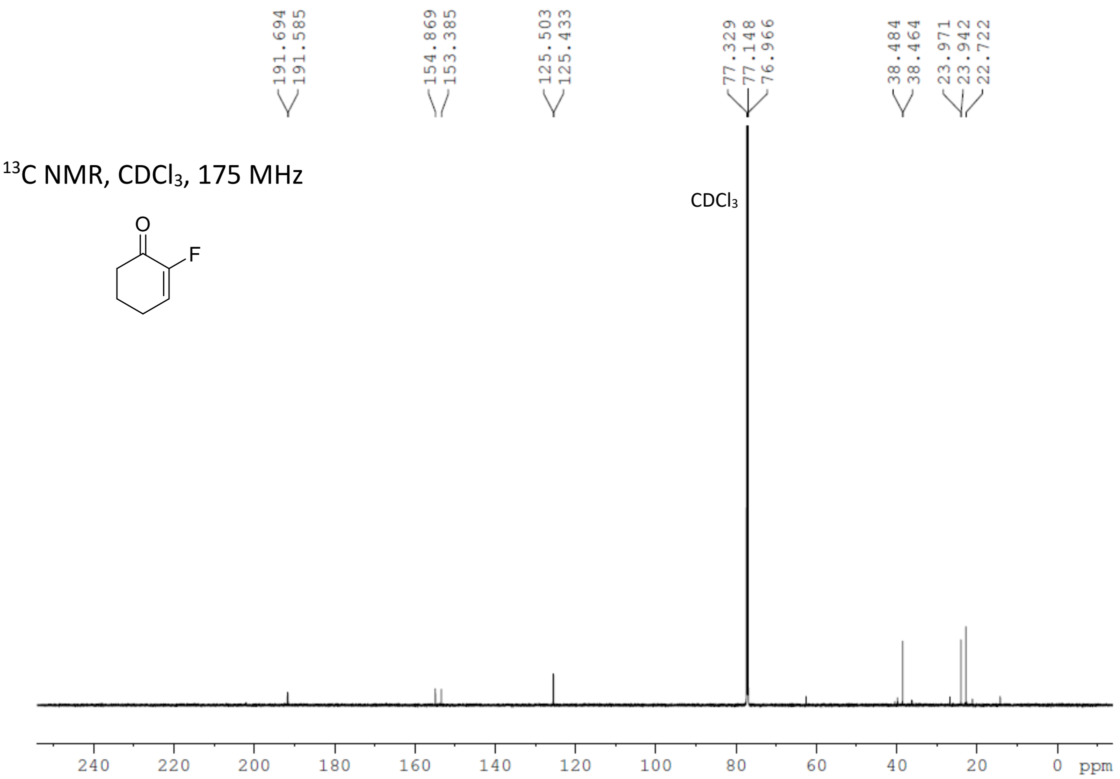
191.694
191.585
154.869
153.385
125.503
125.433
77.329
77.148
76.966
38.484
38.464
23.971
23.942
22.722
$^{13}$C NMR, $CDCl_3$, 175 MHz
O
F
$CDCl_3$
240 220 200 180 160 140 120 100 80 60 40 20 0 ppm

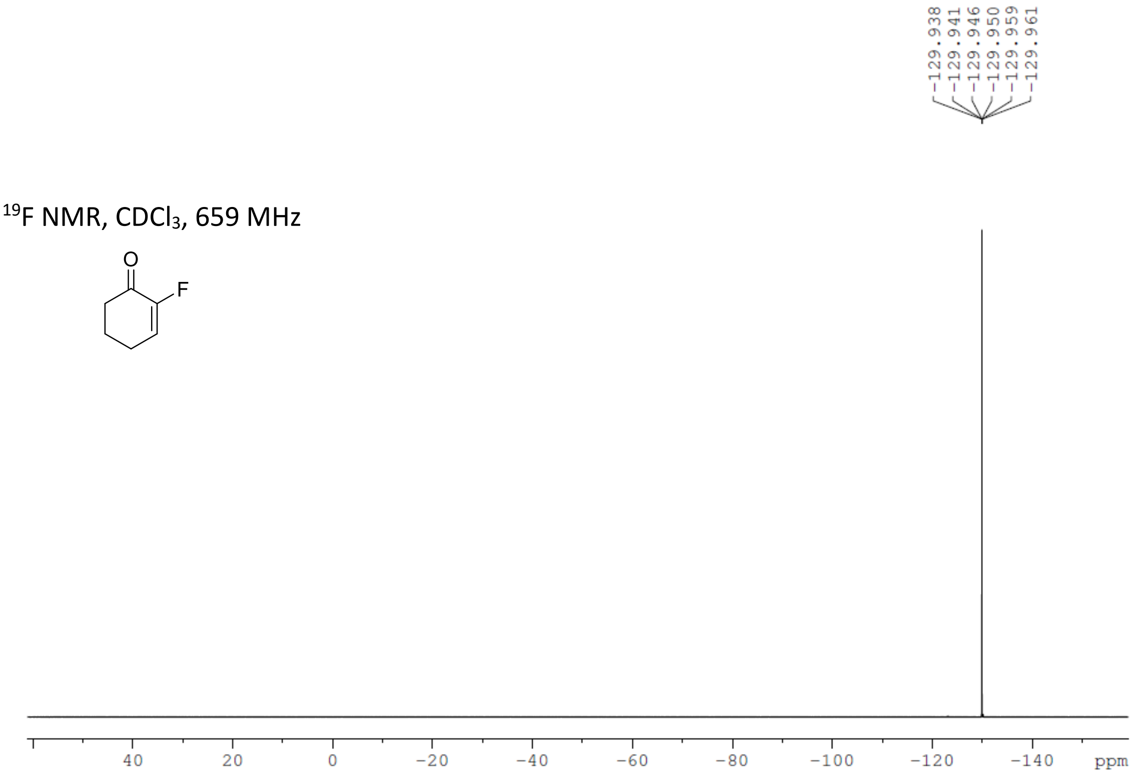
-129.938
-129.941
-129.946
-129.950
-129.959
-129.961
$^{19}$F NMR, $CDCl_3$, 659 MHz
O
F
40 20 0 -20 -40 -60 -80 -100 -120 -140 ppm

(*E*)-2-Fluorocyclohept-2-en-1-one ((*E*)-4b)

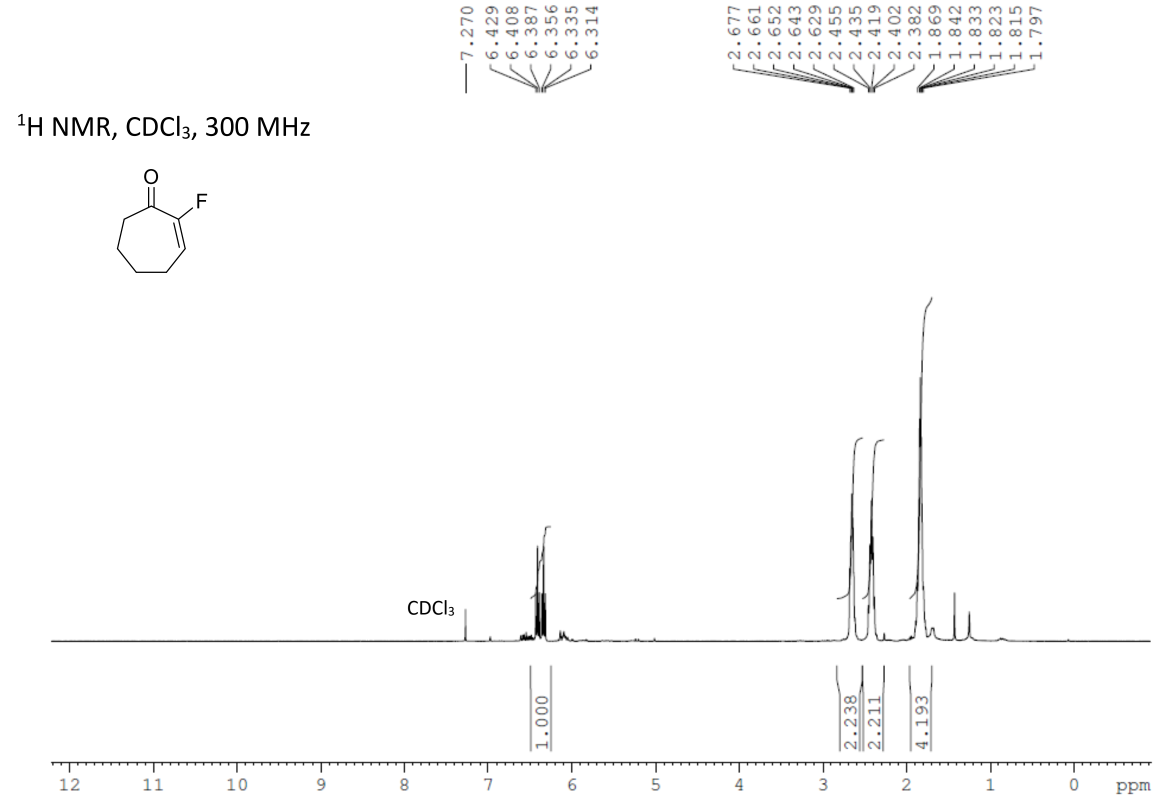

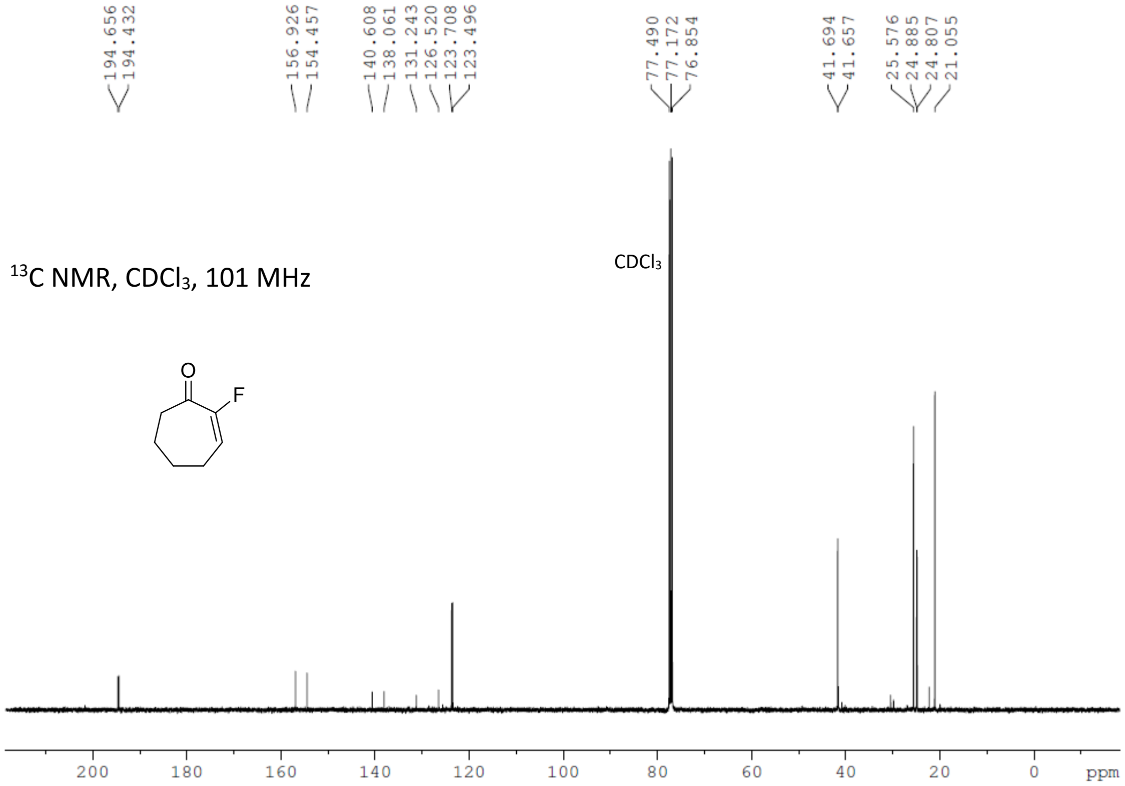
194.656
194.432
156.926
154.457
140.608
138.061
131.243
126.520
123.708
123.496
77.490
77.172
76.854
41.694
41.657
25.576
24.885
24.807
21.055
13C NMR, CDCl3, 101 MHz
CDCl3
O
F
200
180
160
140
120
100
80
60
40
20
0
ppm

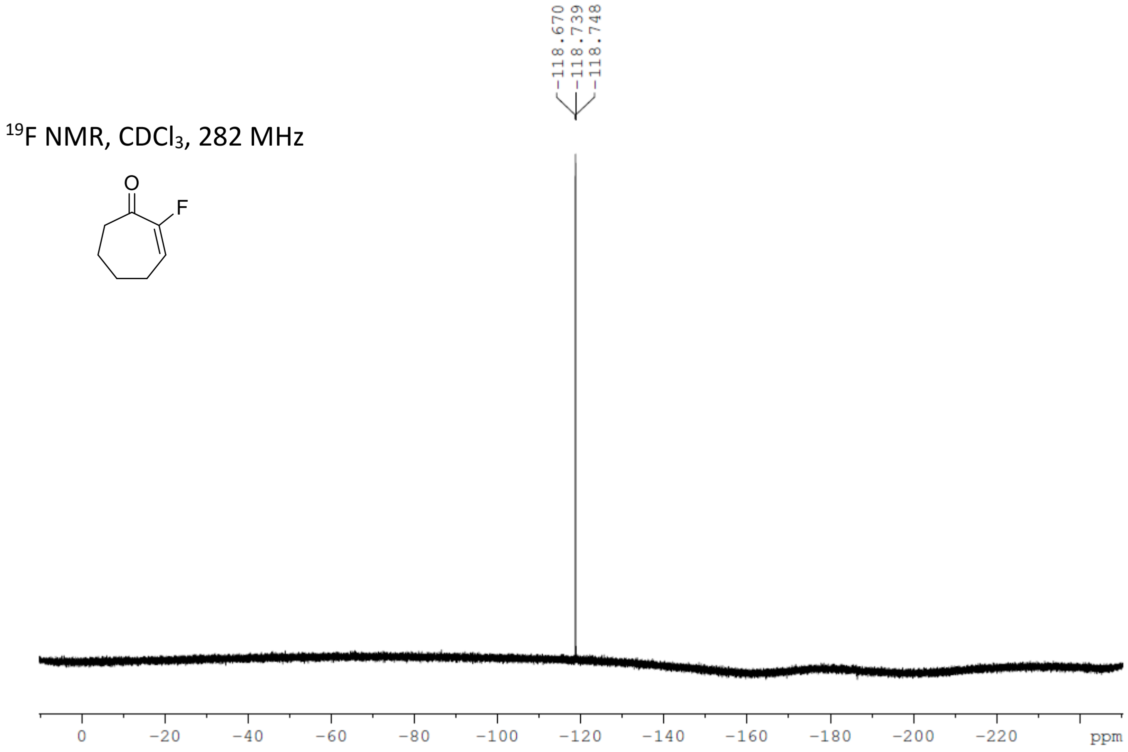
-118.670
-118.739
-118.748
19F NMR, CDCl3, 282 MHz
O
F
0
-20
-40
-60
-80
-100
-120
-140
-160
-180
-200
-220
ppm

# (*Z*)-2-Fluoro-1-phenylpent-1-en-3-one ((*Z*)-5a)

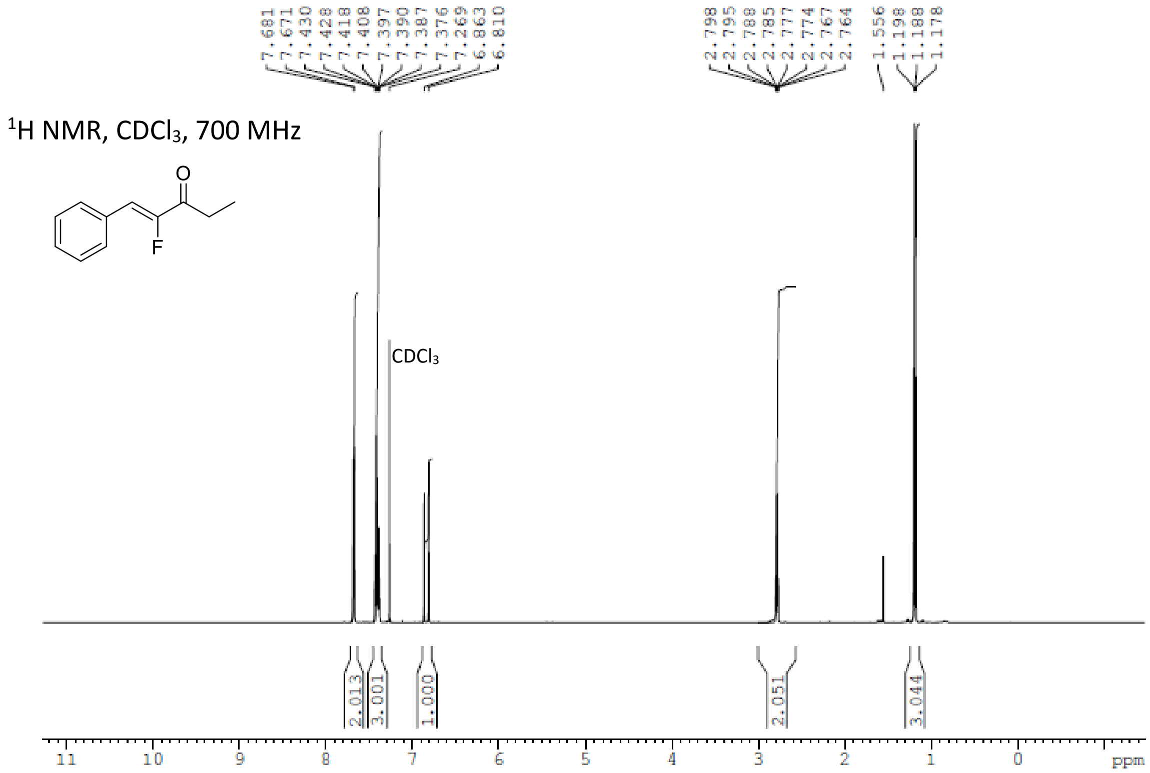


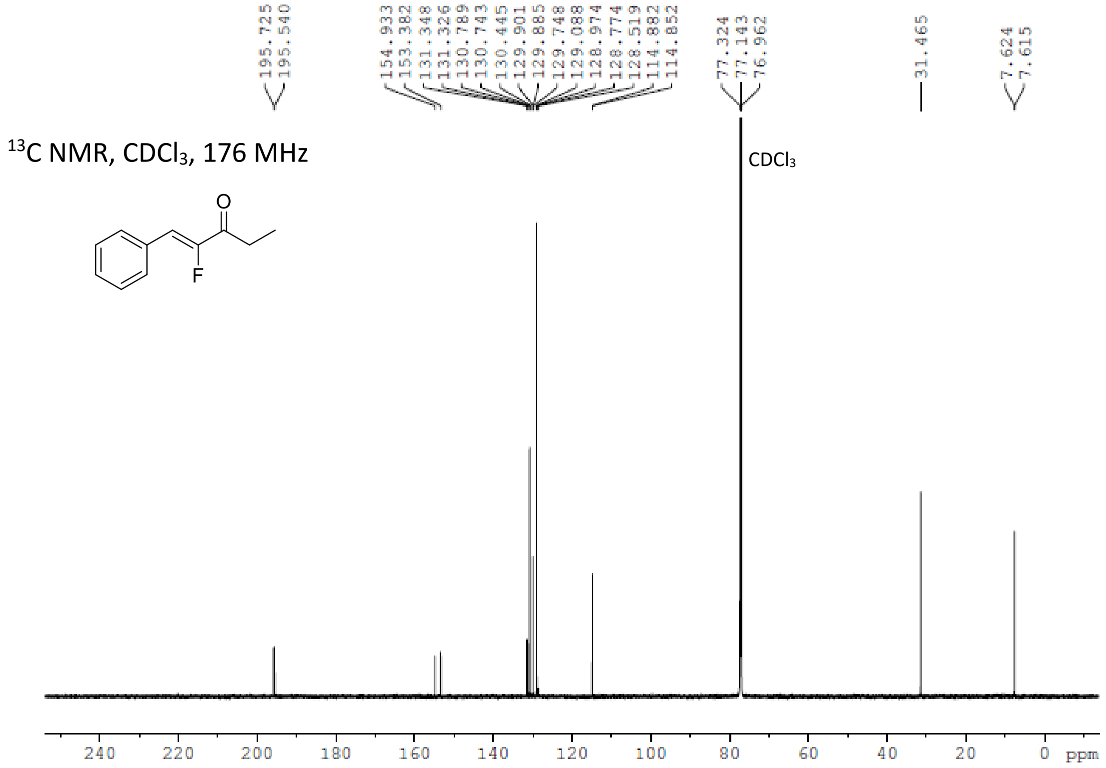

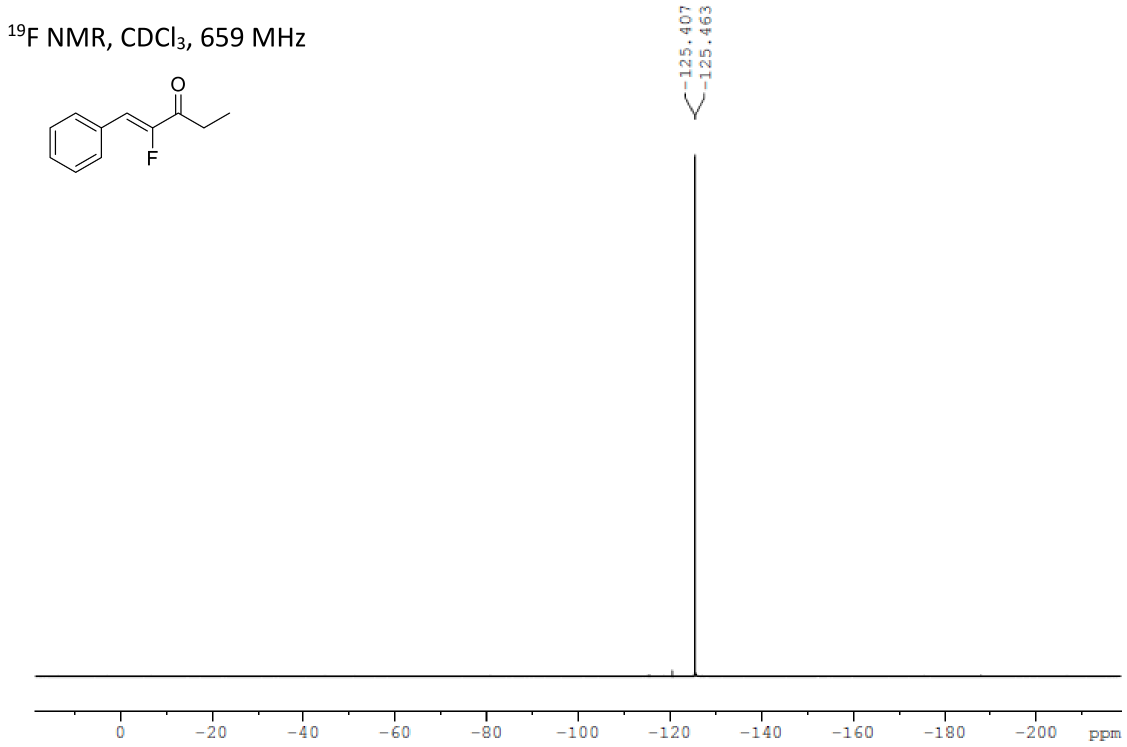


## (*E*)-2-Fluoro-1-phenylpent-1-en-3-one ((*E*)-5a)

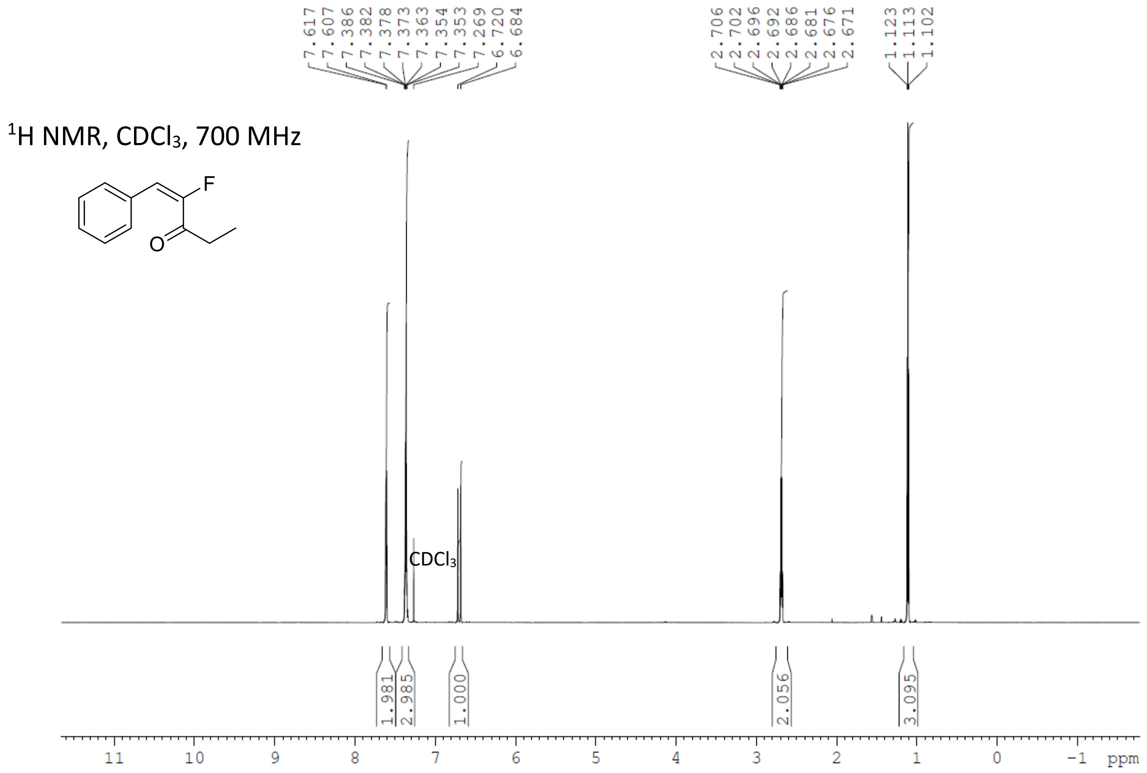

$^{13}$C NMR, $CDCl_3$, 176 MHz

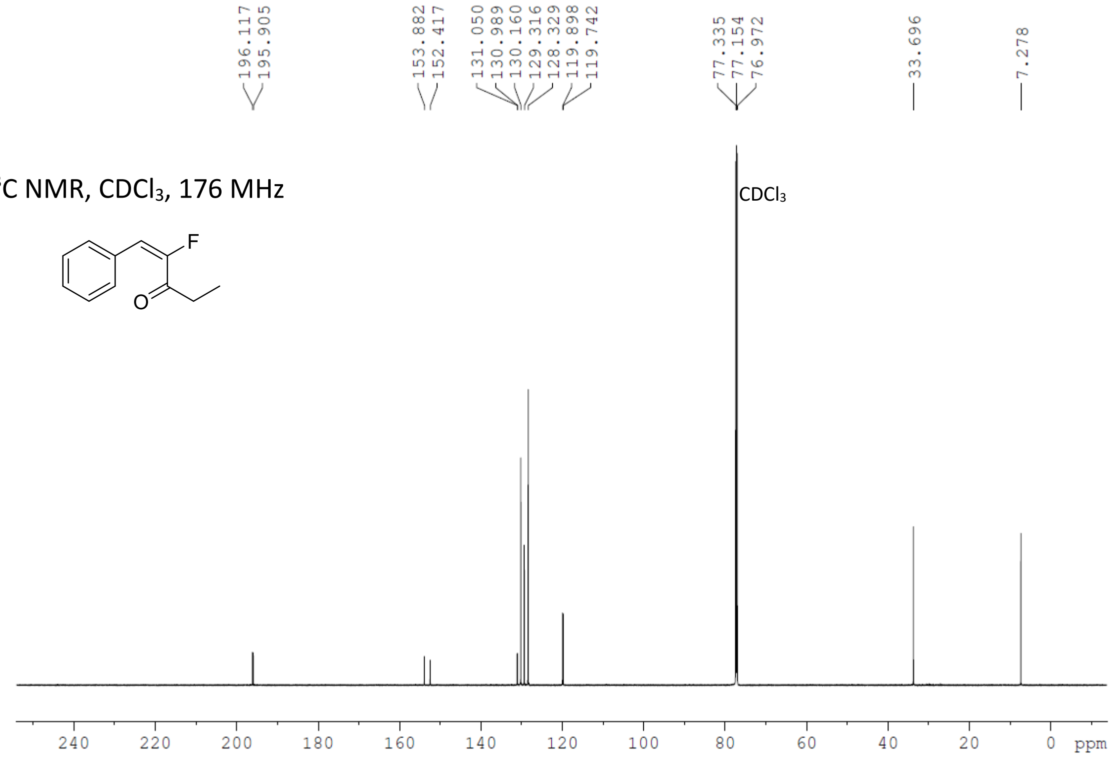


$^{19}$F NMR, $CDCl_3$, 659 MHz

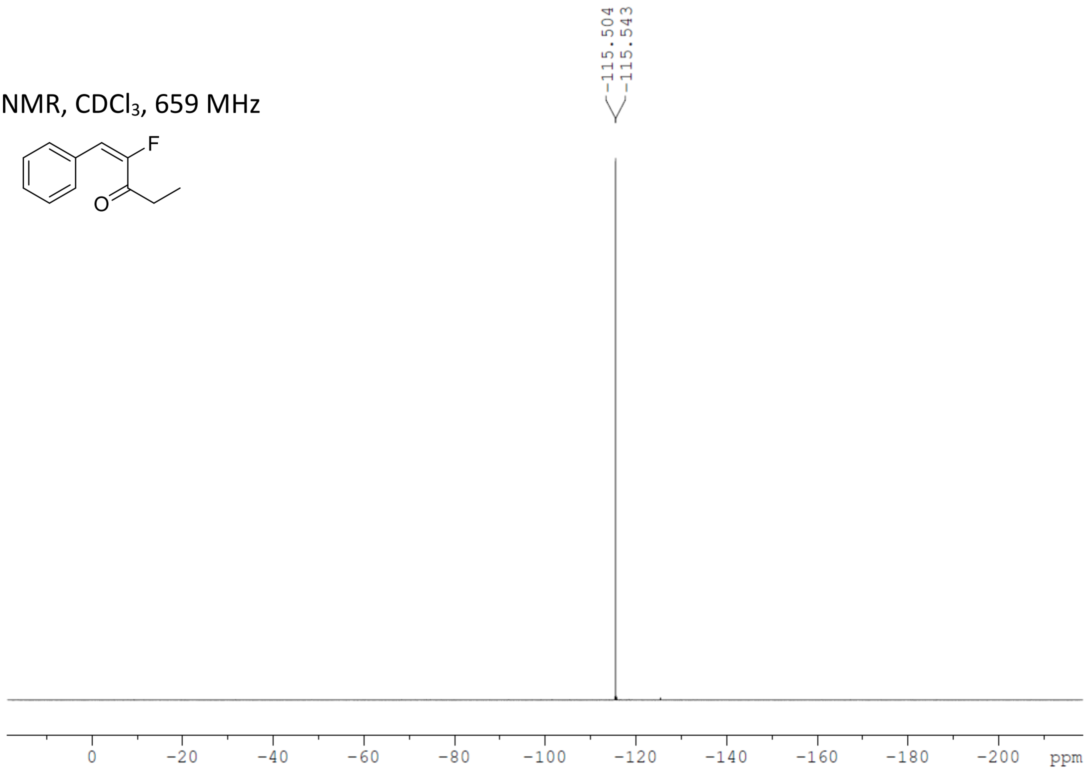

## (*Z*)-2-Fluoro-1-phenylhept-2-en-1-one ((*Z*)-5b)

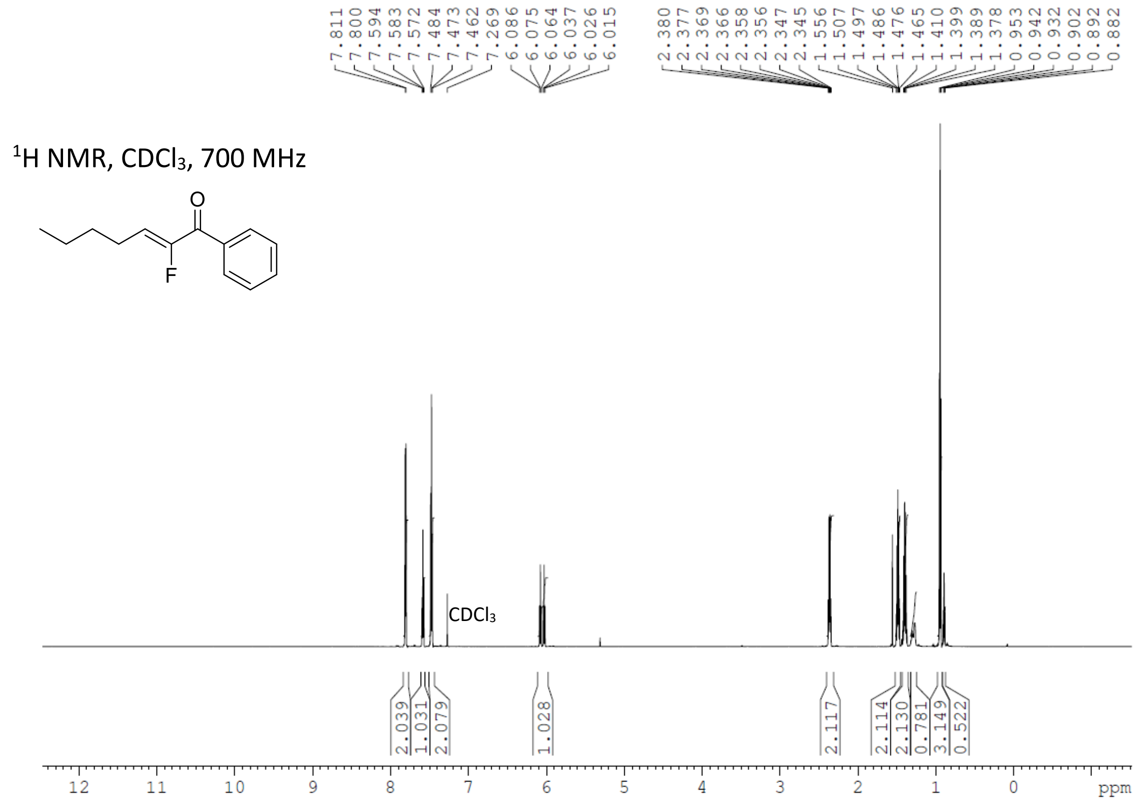


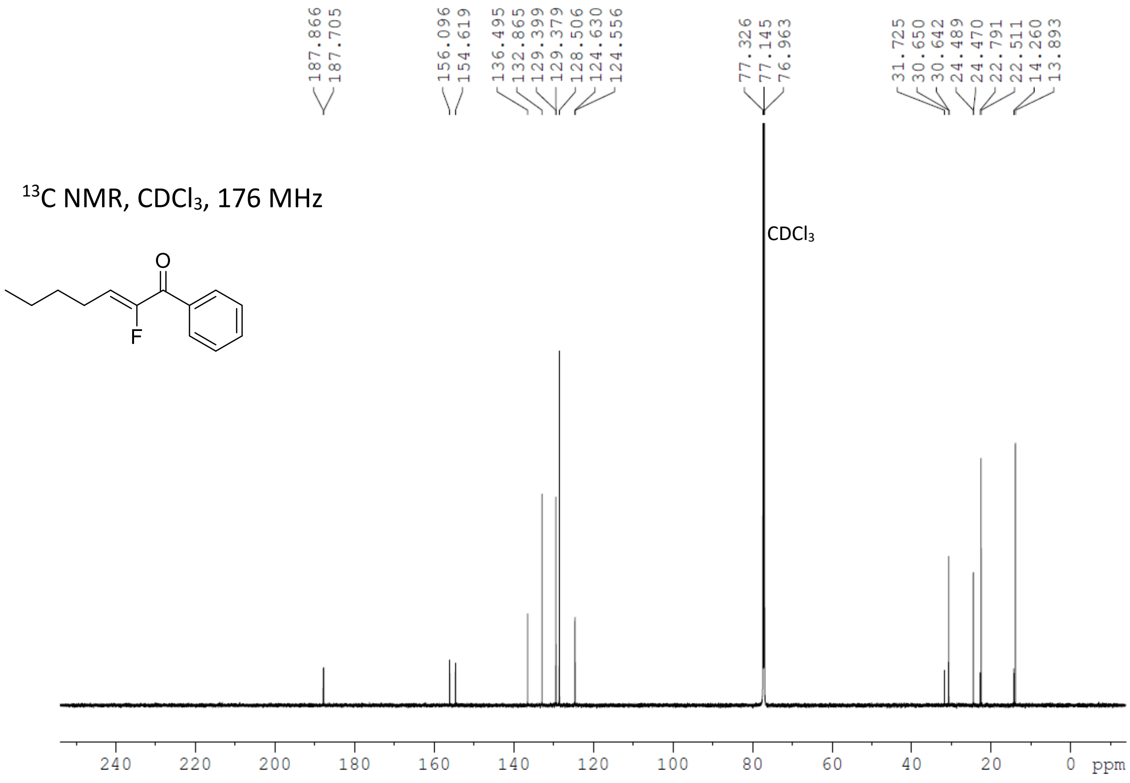

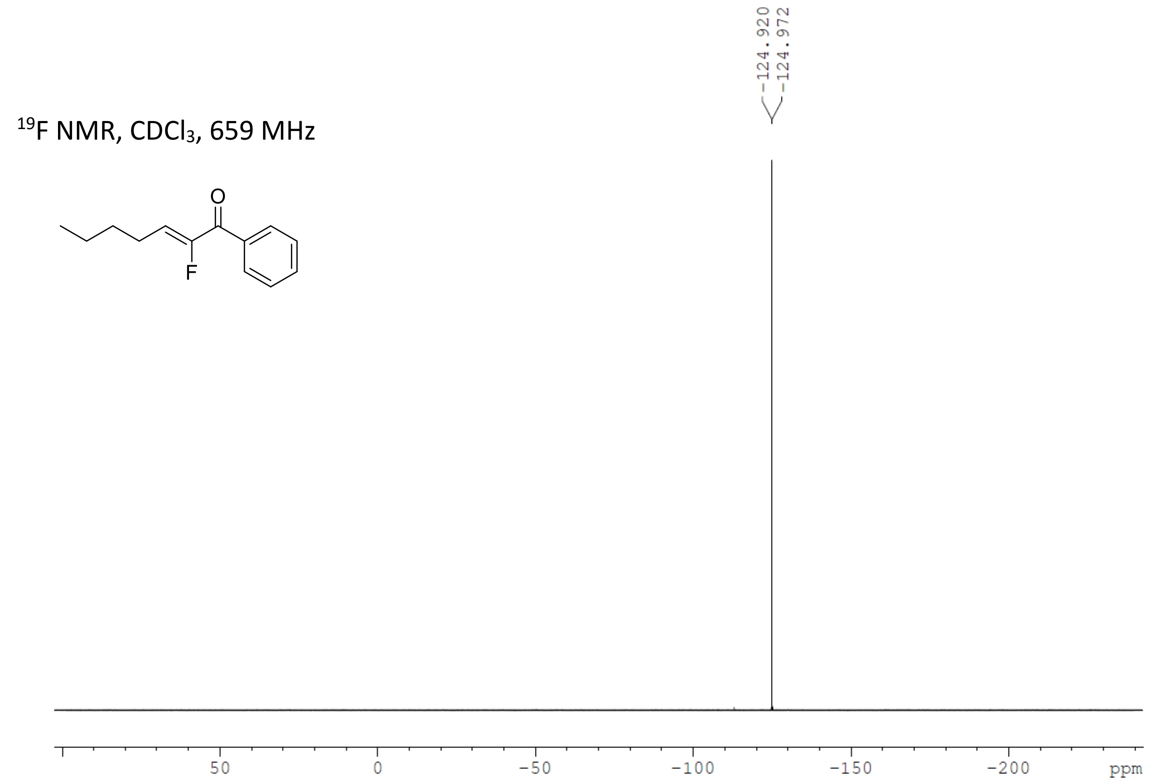


## (*E*)-2-Fluoro-1-phenylhept-2-en-1-one ((*E*)-5b) (impure)

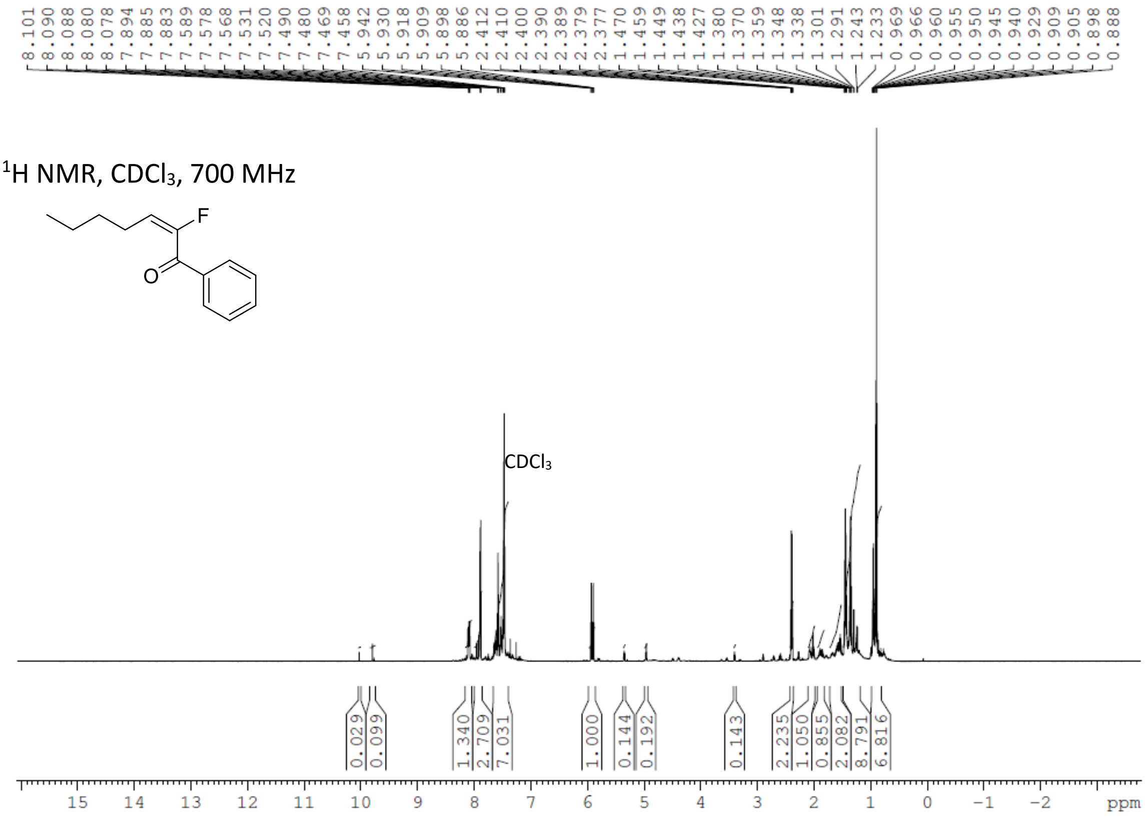

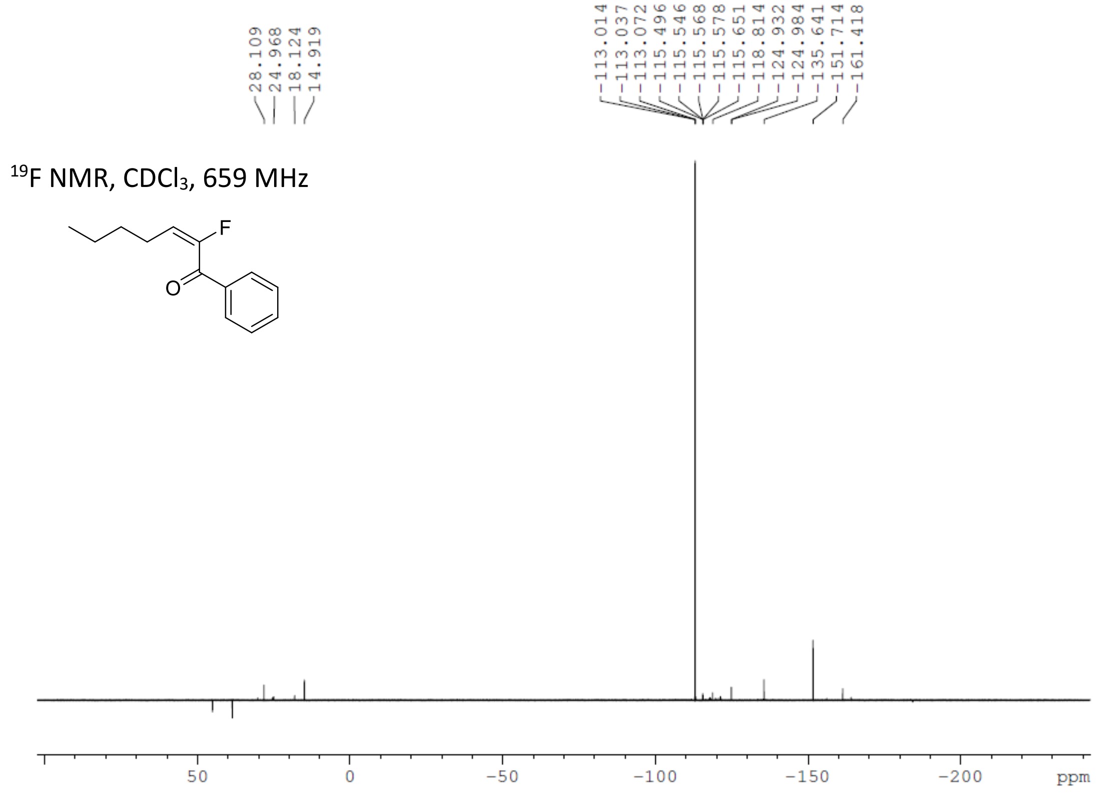


## (*Z*)-3-fluoro-4-phenylbut-3-en-2-one-4-d ((*Z*)-4-D-1a)

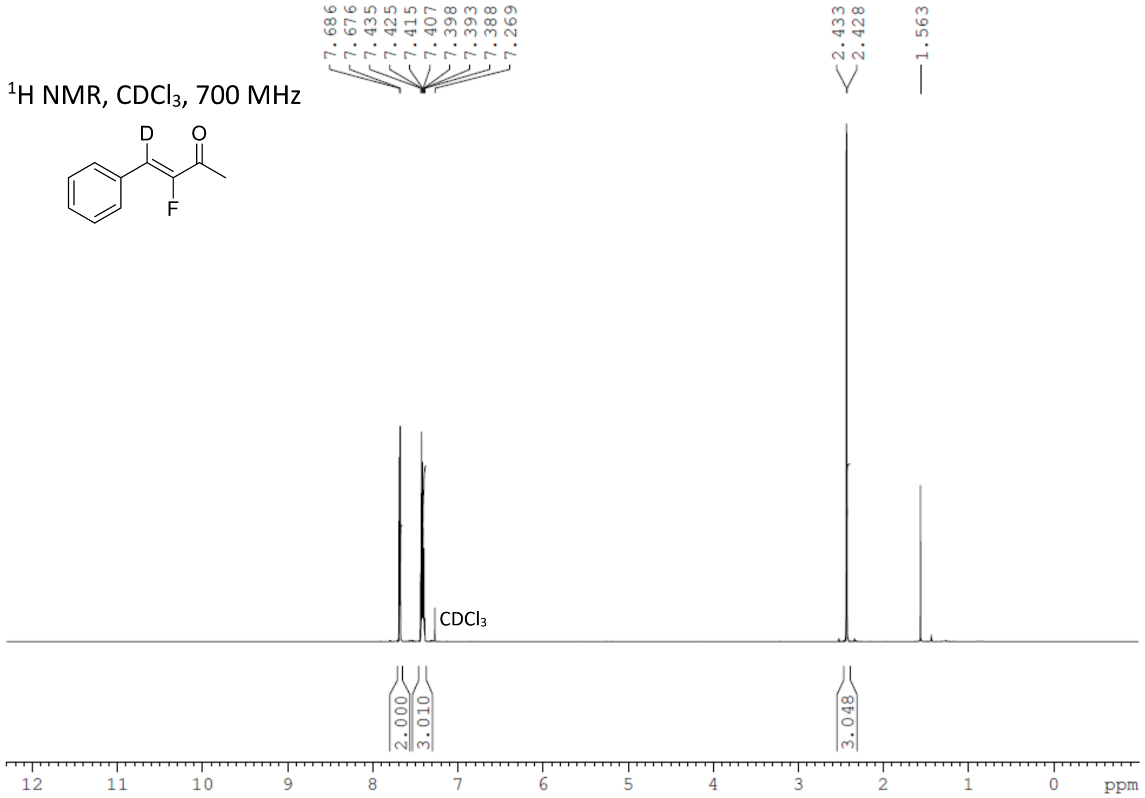

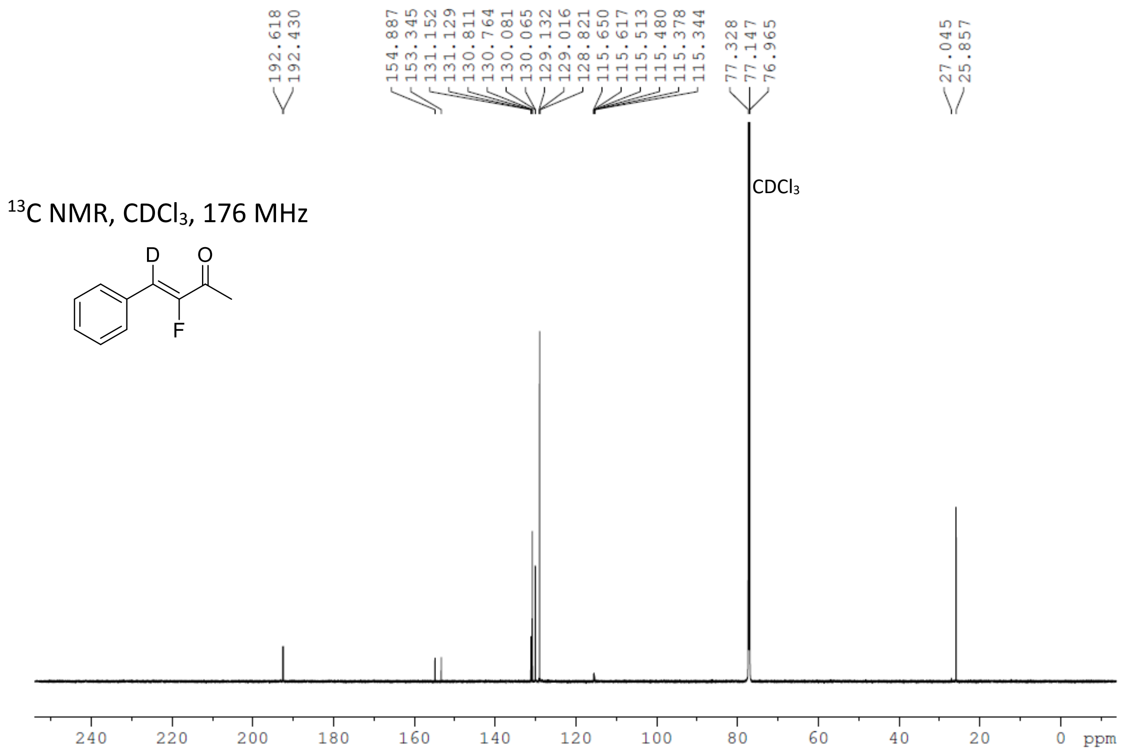
192.618
192.430
154.887
153.345
131.152
131.129
130.811
130.764
130.081
130.065
129.132
129.016
128.821
115.650
115.617
115.513
115.480
115.378
115.344
77.328
77.147
76.965
27.045
25.857
CDCl3
13C NMR, CDCl3, 176 MHz
D
O
F
240
220
200
180
160
140
120
100
80
60
40
20
0
ppm

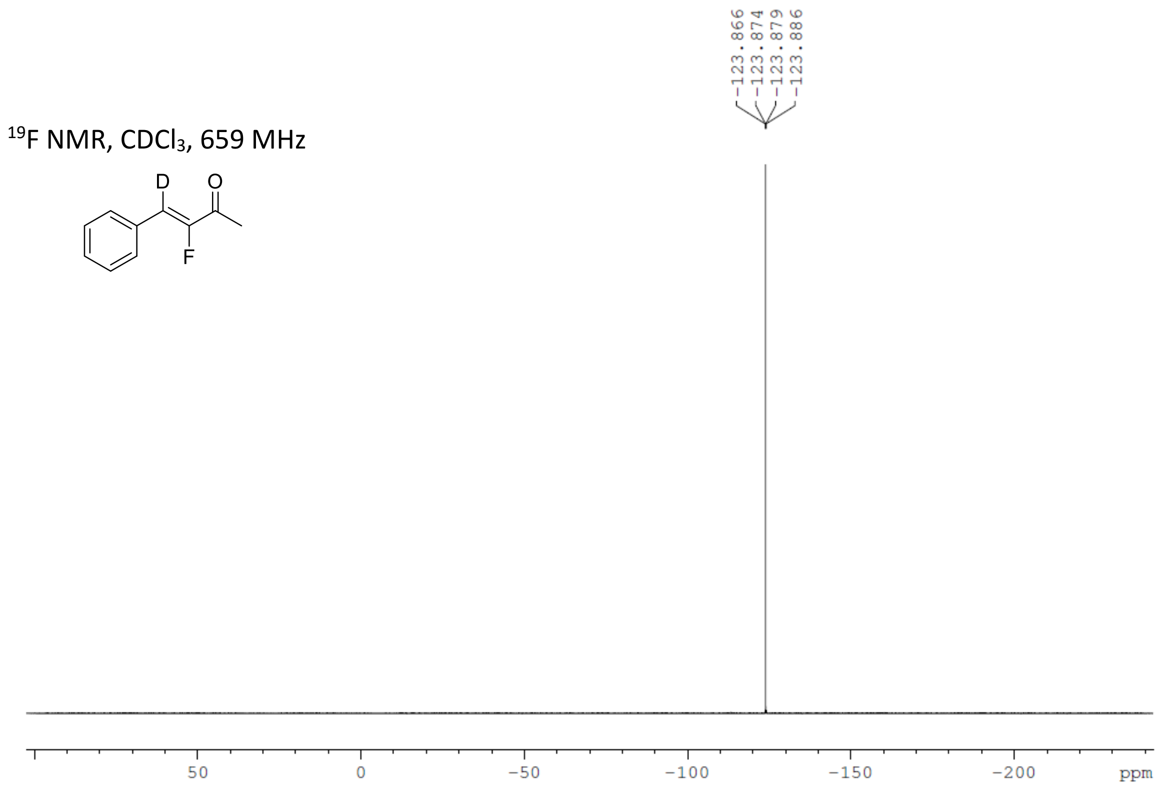
-123.866
-123.874
-123.879
-123.886
19F NMR, CDCl3, 659 MHz
D
O
F
50
0
-50
-100
-150
-200
ppm

(*E*)-3-Fluoro-4-phenylbut-3-en-2-one-4-d ((*E*)-4-D-1a)

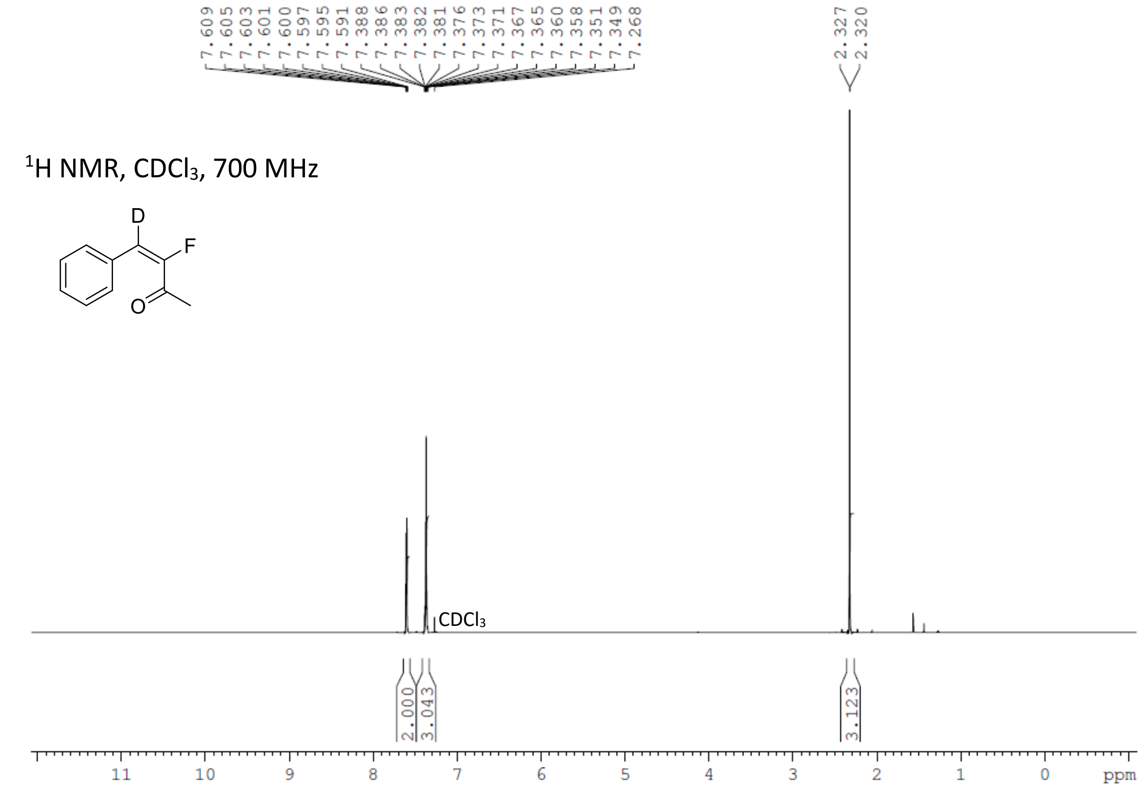


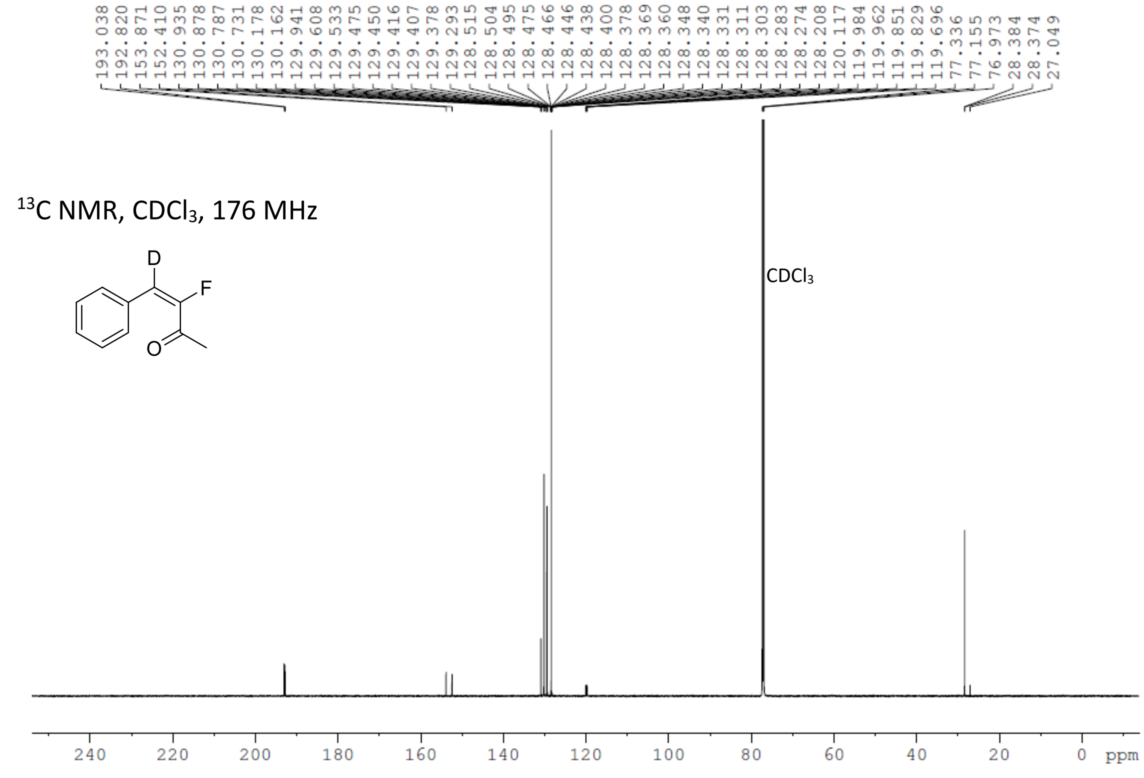

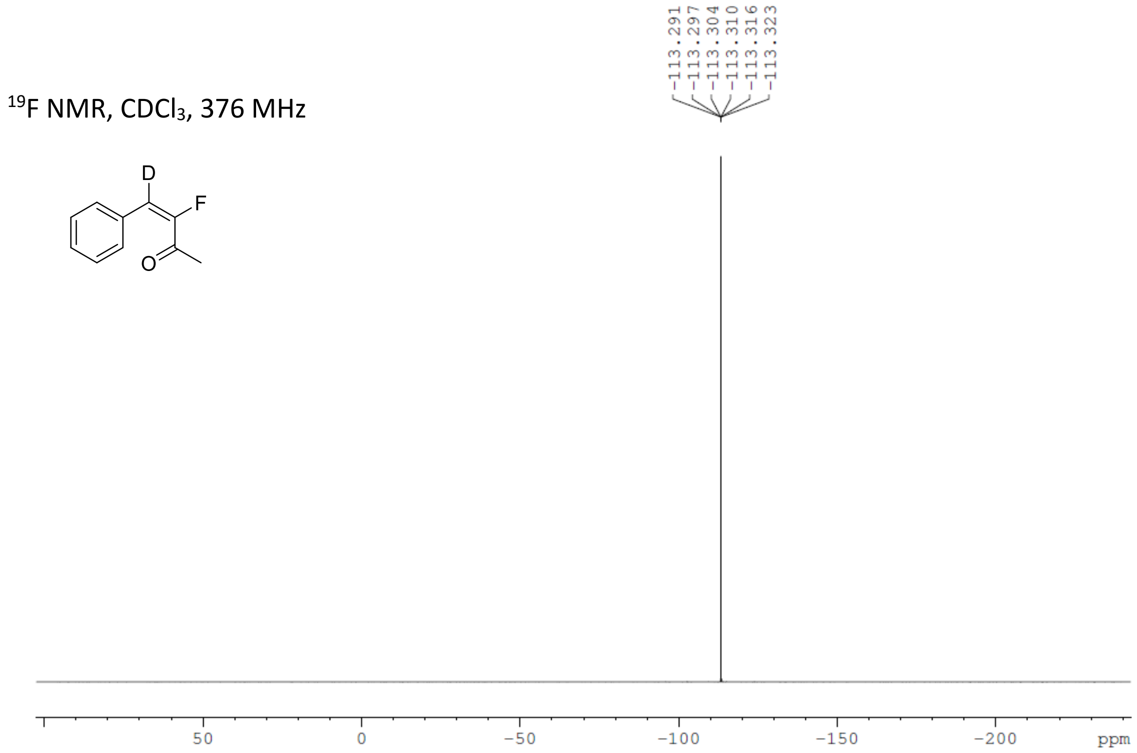


# 3-Fluoro-4-phenylbutan-2-one (7a)

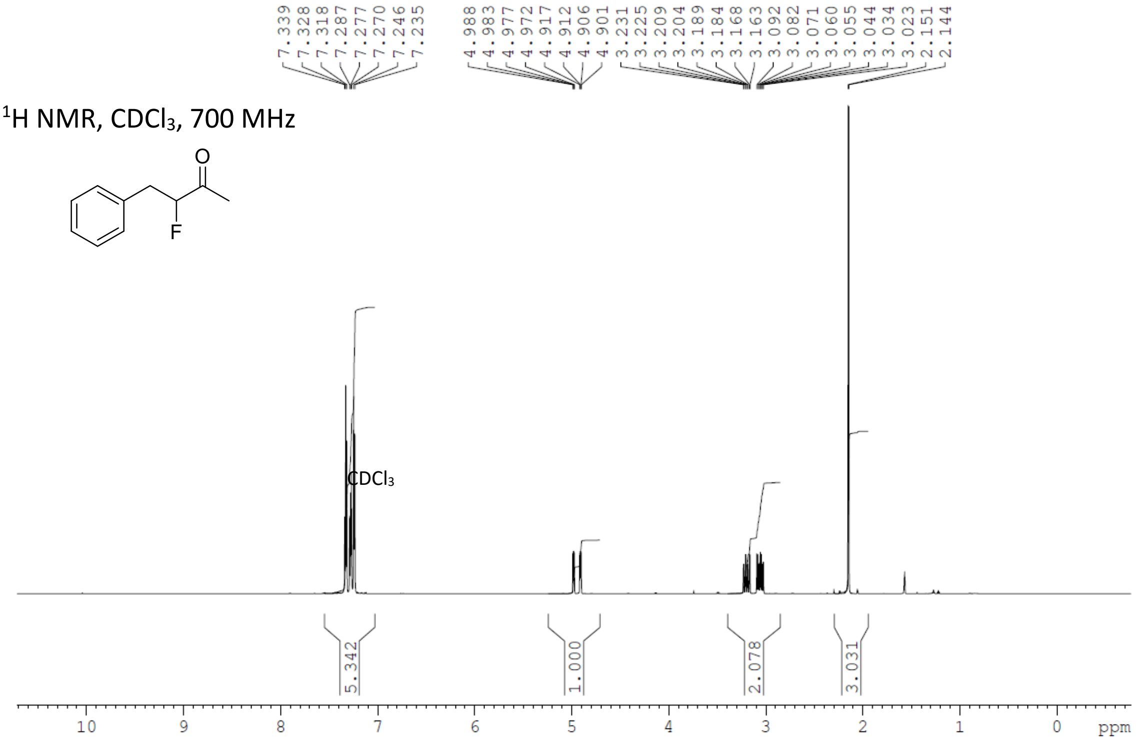

$^{13}C$ NMR, $CDCl_3$, 176 MHz

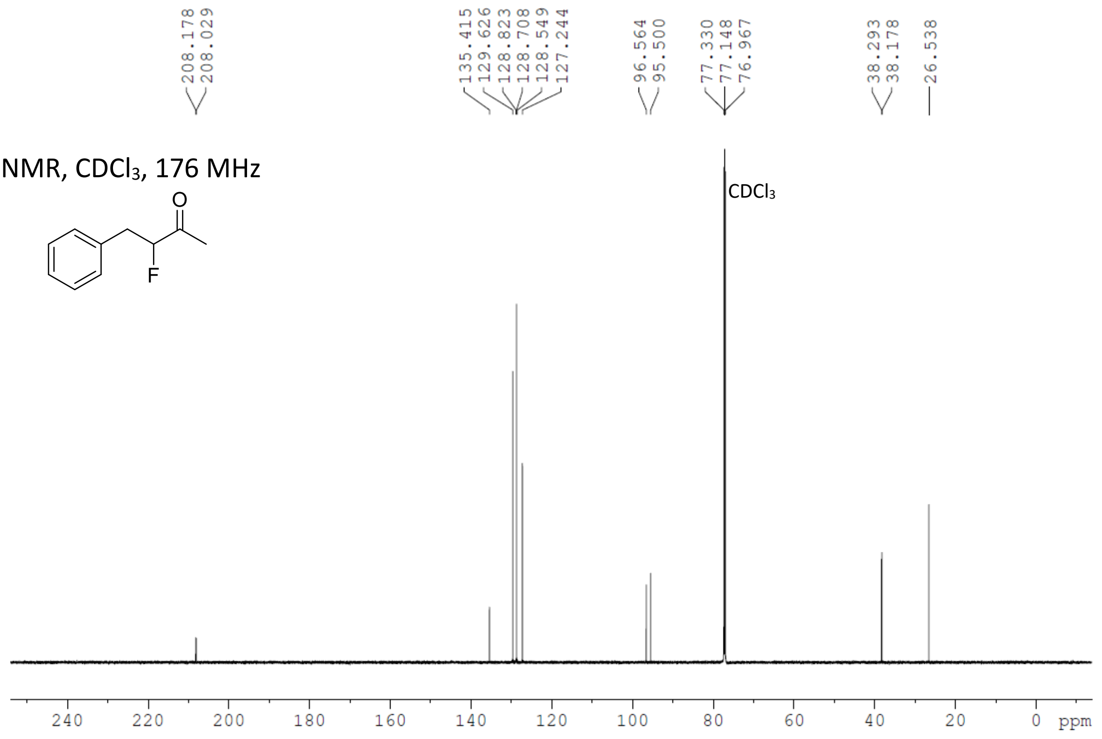


$^{19}F$ NMR, $CDCl_3$, 376 MHz

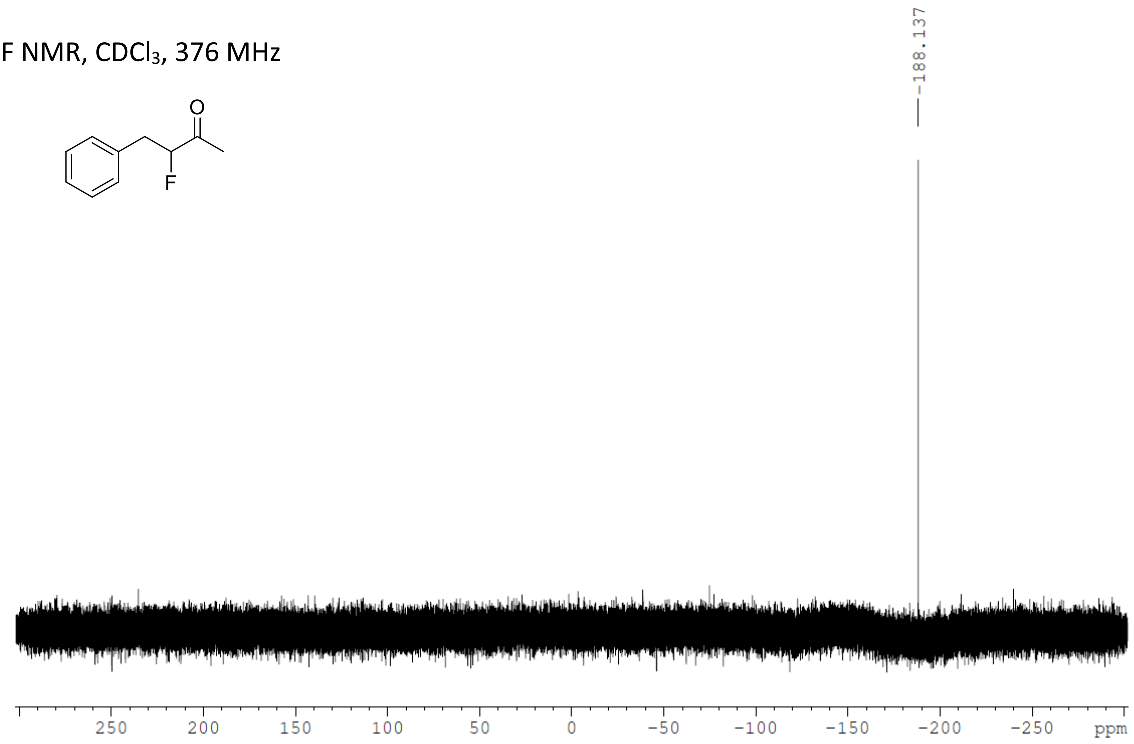

3-Fluoro-4-(4-trifluoromethylphenyl)-butan-2-one (7b)

$^{1}$H NMR, $CDCl_3$, 700 MHz

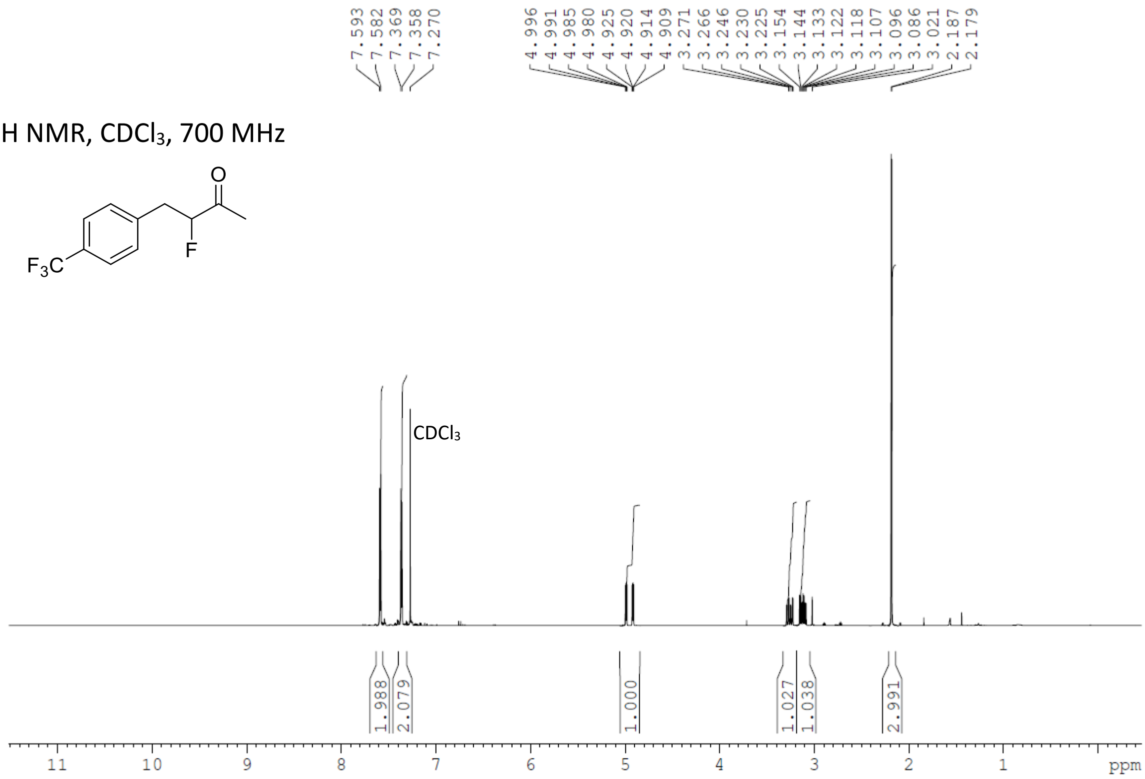


$^{13}$C NMR, $CDCl_3$, 176 MHz

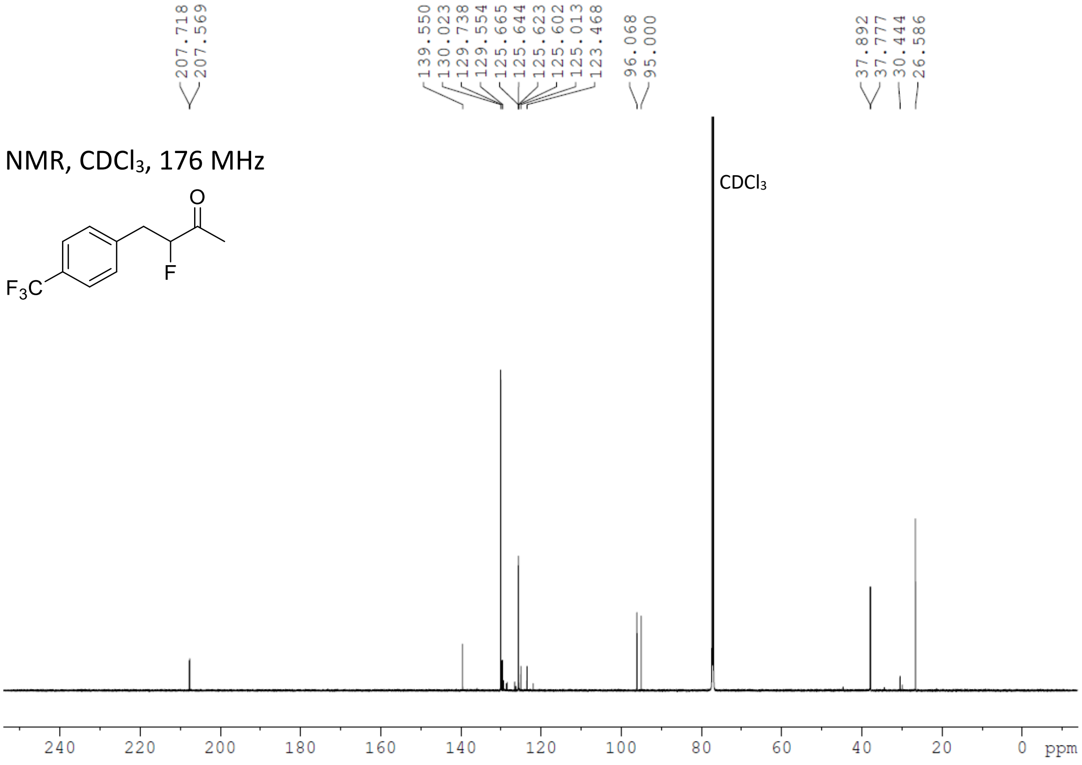

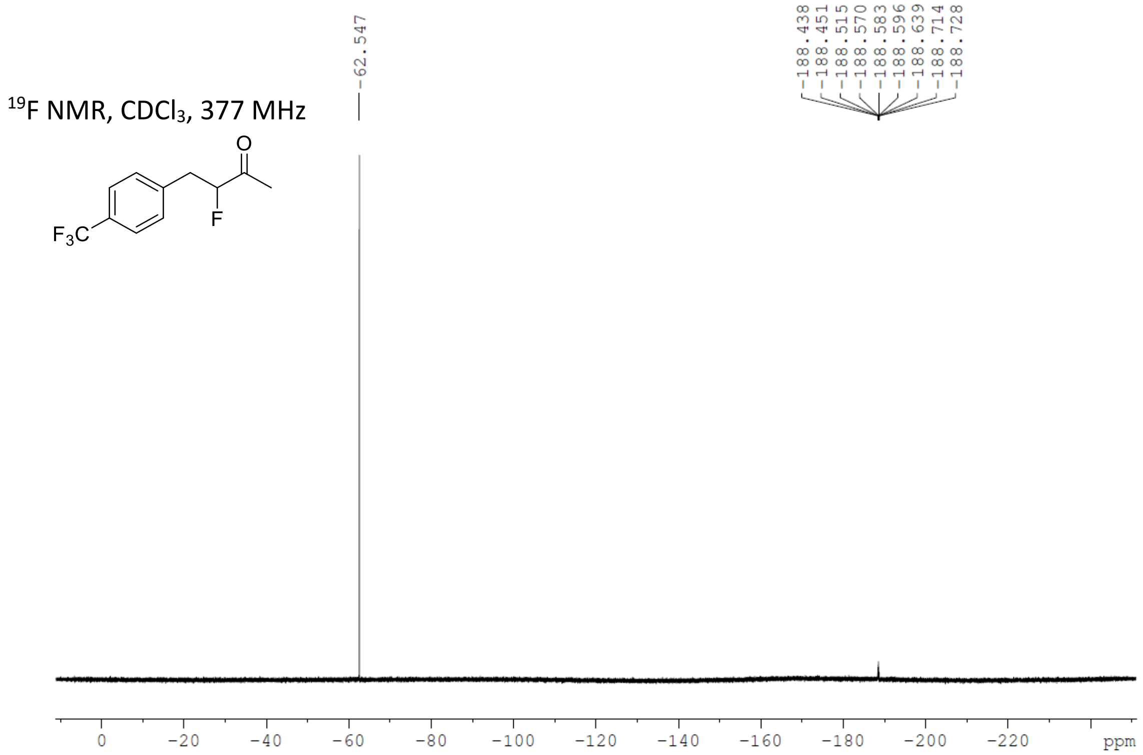


## 3-Fluoro-4-(4-methoxyphenyl)-butan-2-one (7c)

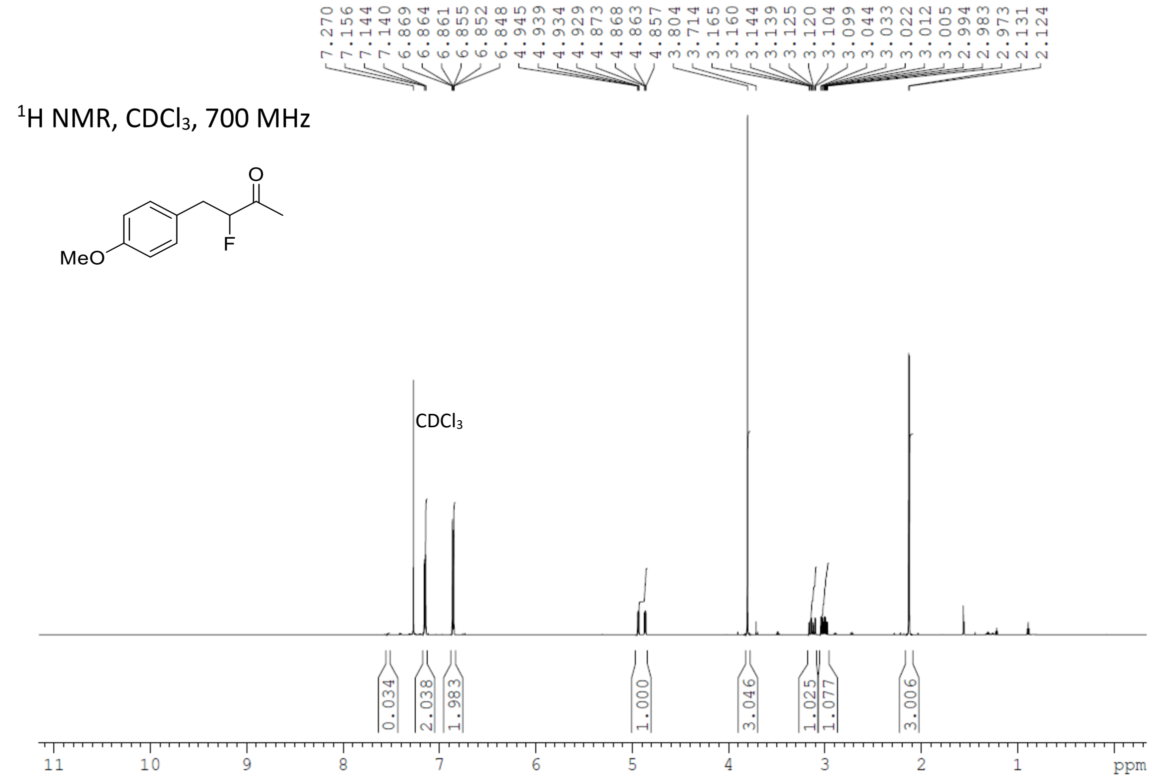

$^{13}C$ NMR, $CDCl_3$, 176 MHz

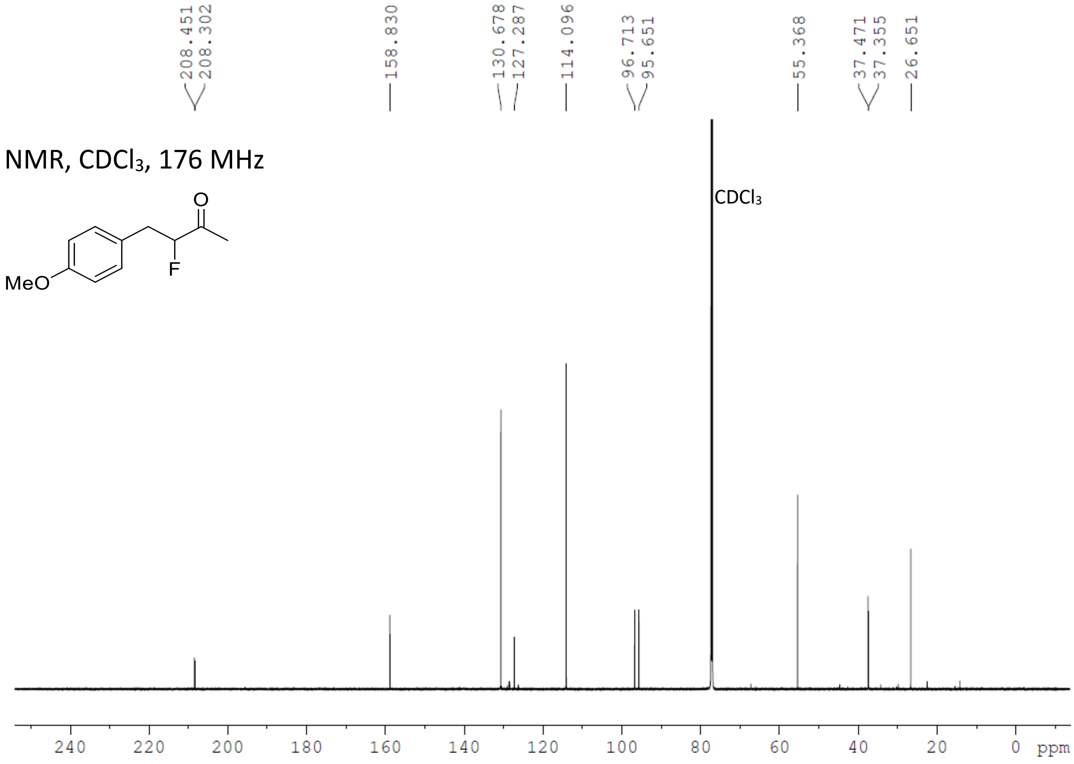


$^{19}F$ NMR, $CDCl_3$, 282 MHz

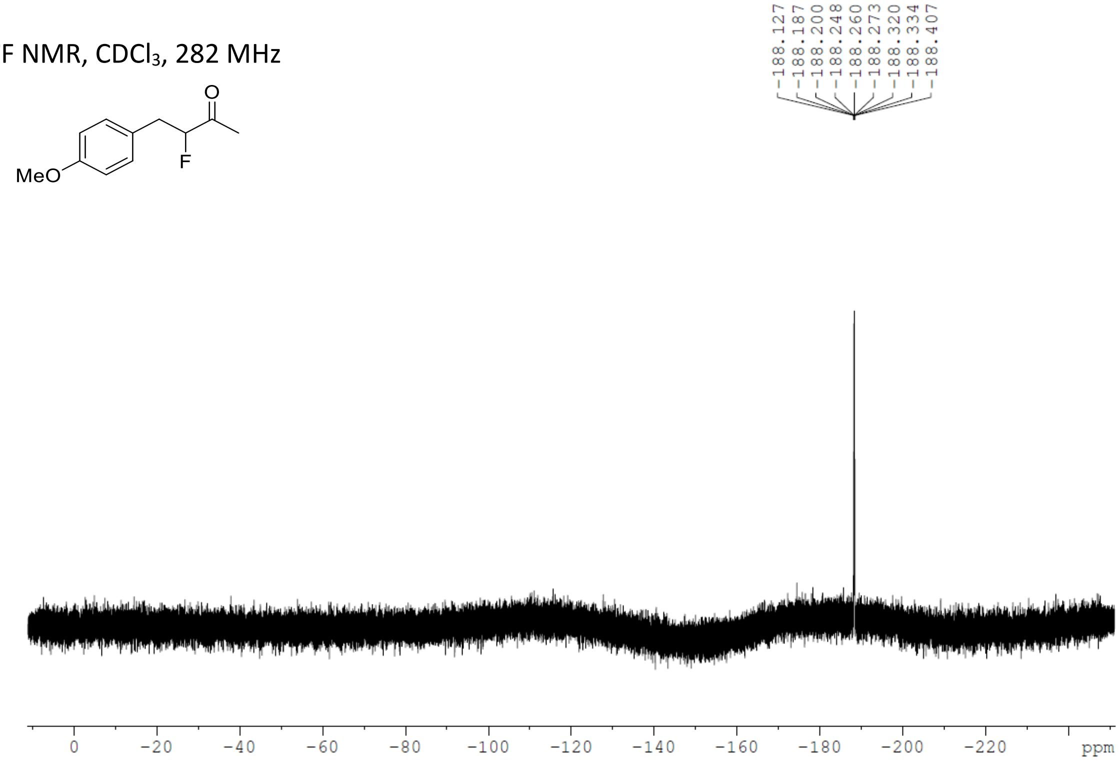

# 3-Fluoro-4-(4-cyanophenyl)-butan-2-one (7d)

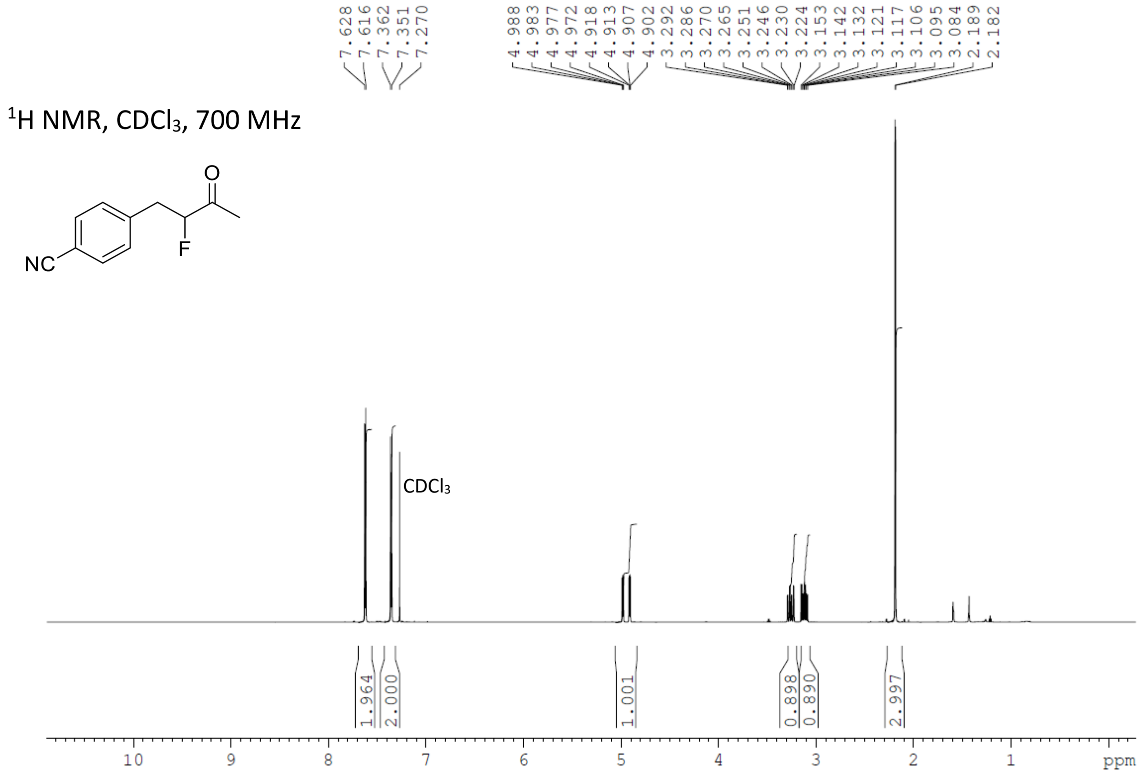


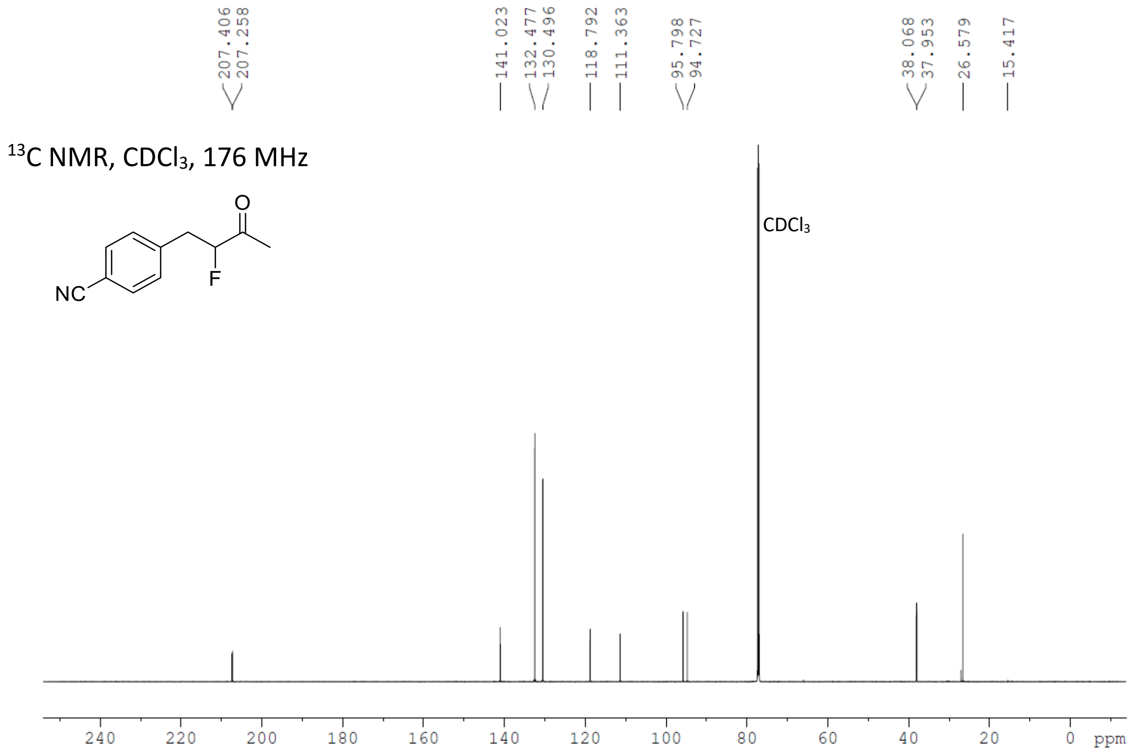

$^{19}F$ NMR, $CDCl_3$, 659 MHz

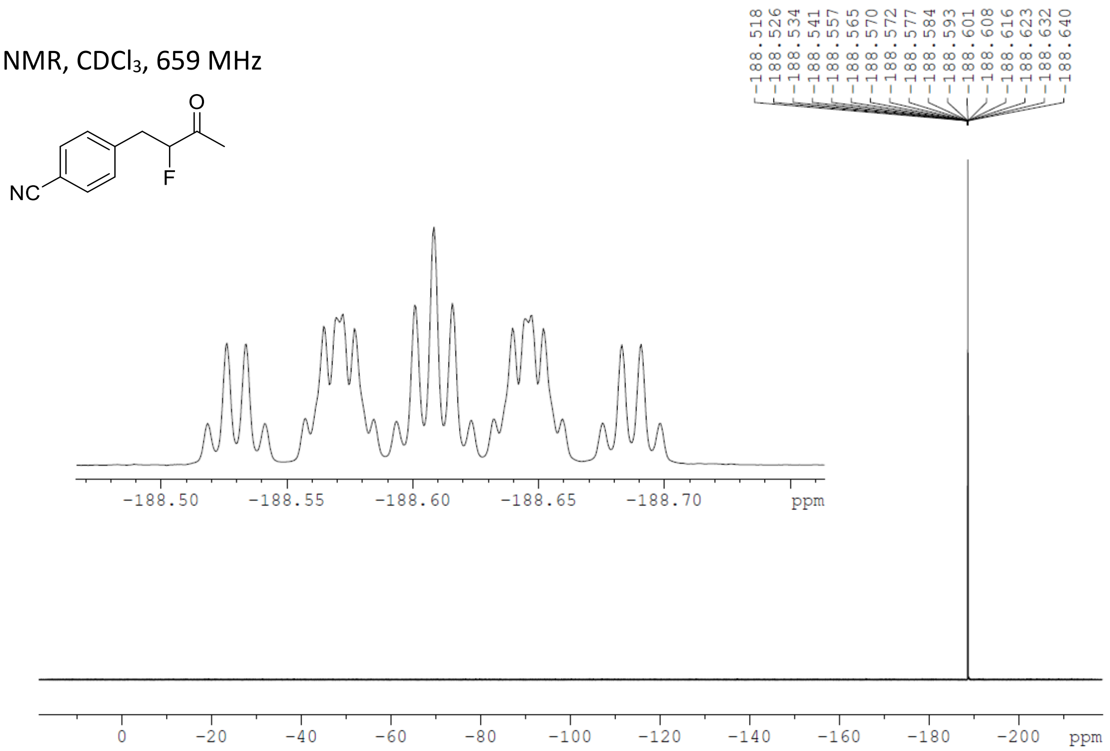


## 3-Fluoro-4-(2-methylphenyl)-butan-2-one (7e)

$^{1}H$ NMR, $CDCl_3$, 400 MHz

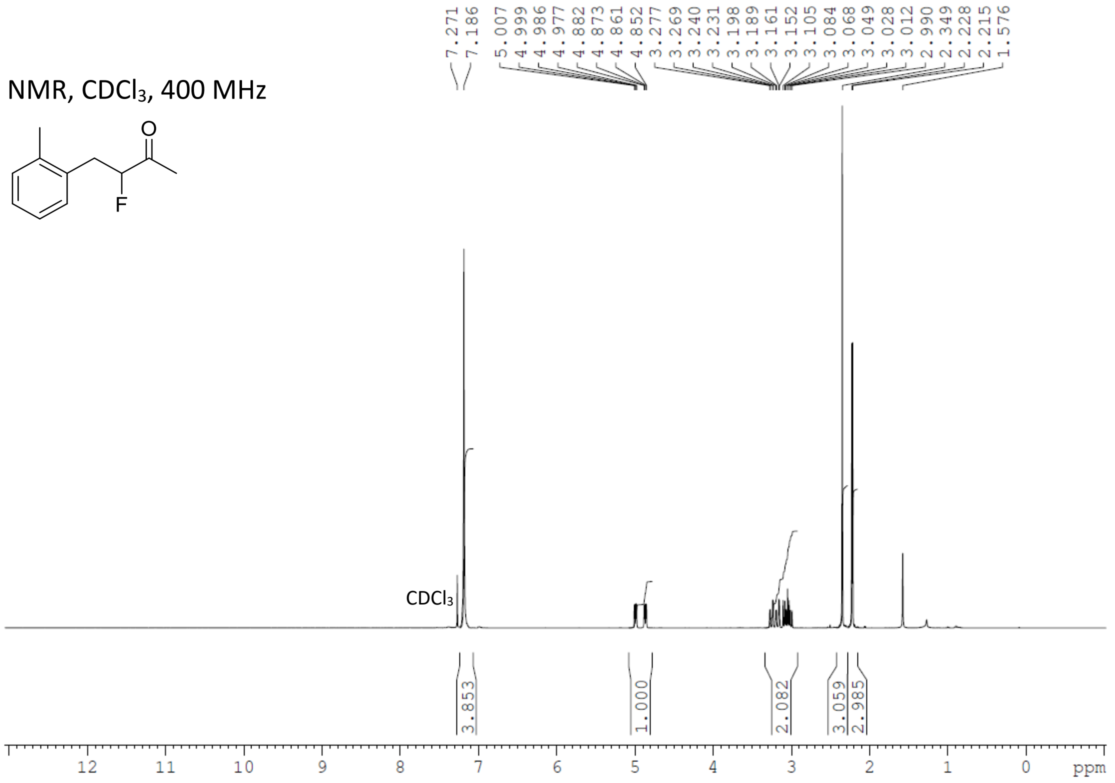

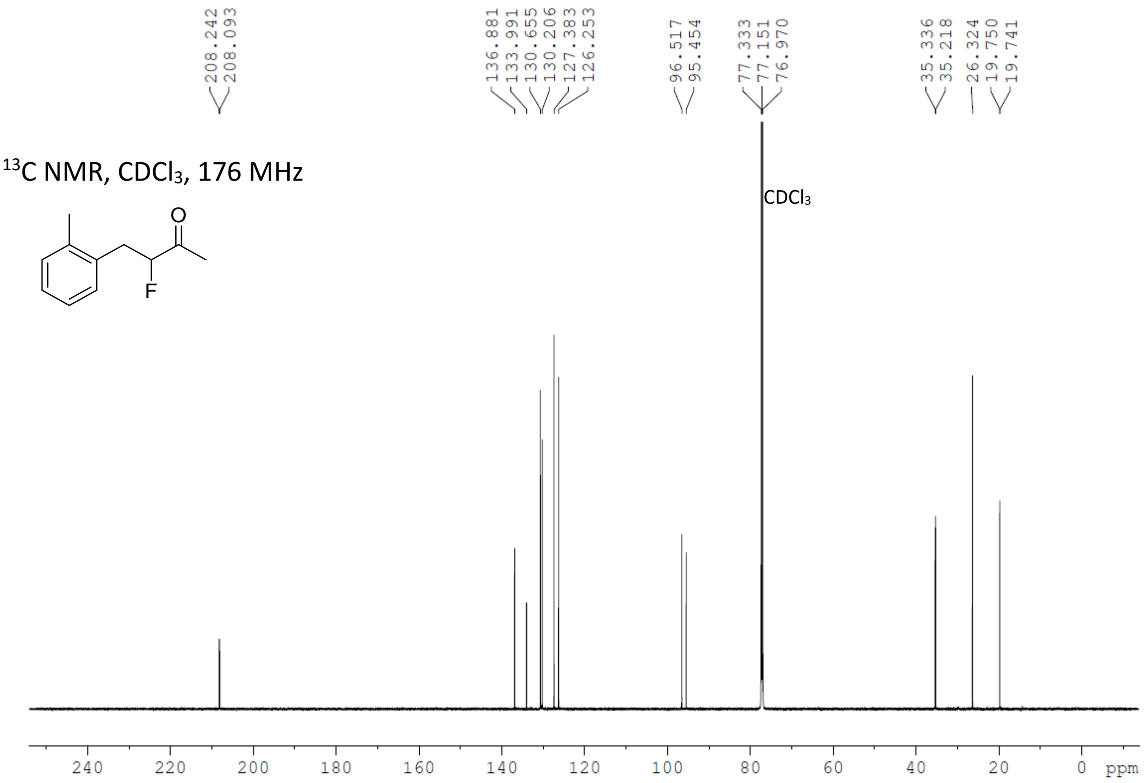
13C NMR, CDCl3, 176 MHz
208.242
208.093
136.881
133.991
130.655
130.206
127.383
126.253
96.517
95.454
77.333
77.151
76.970
35.336
35.218
26.324
19.750
19.741
CDCl3
O
F
240
220
200
180
160
140
120
100
80
60
40
20
0
ppm

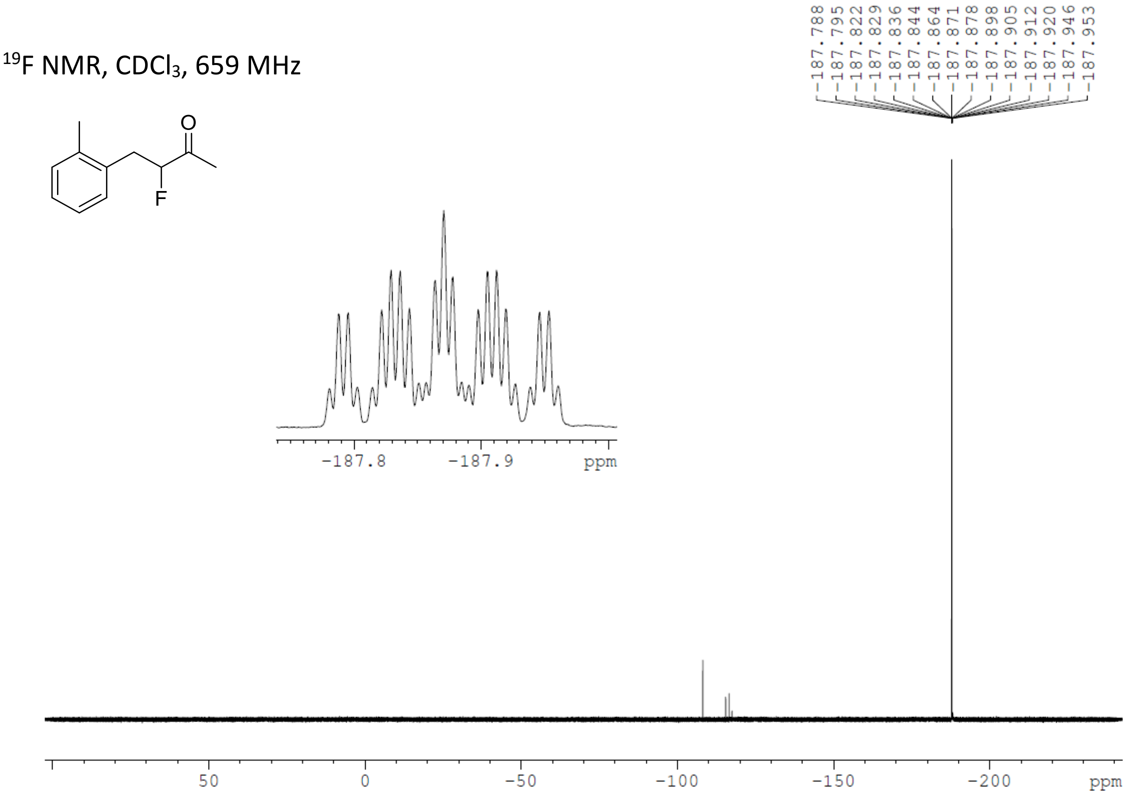
19F NMR, CDCl3, 659 MHz
-187.788
-187.795
-187.822
-187.829
-187.836
-187.844
-187.864
-187.871
-187.878
-187.898
-187.905
-187.912
-187.920
-187.946
-187.953
O
F
-187.8
-187.9
ppm
50
0
-50
-100
-150
-200
ppm

# 3-Fluoro-4-(3-trifluoromethylphenyl)-butan-2-one (7f)

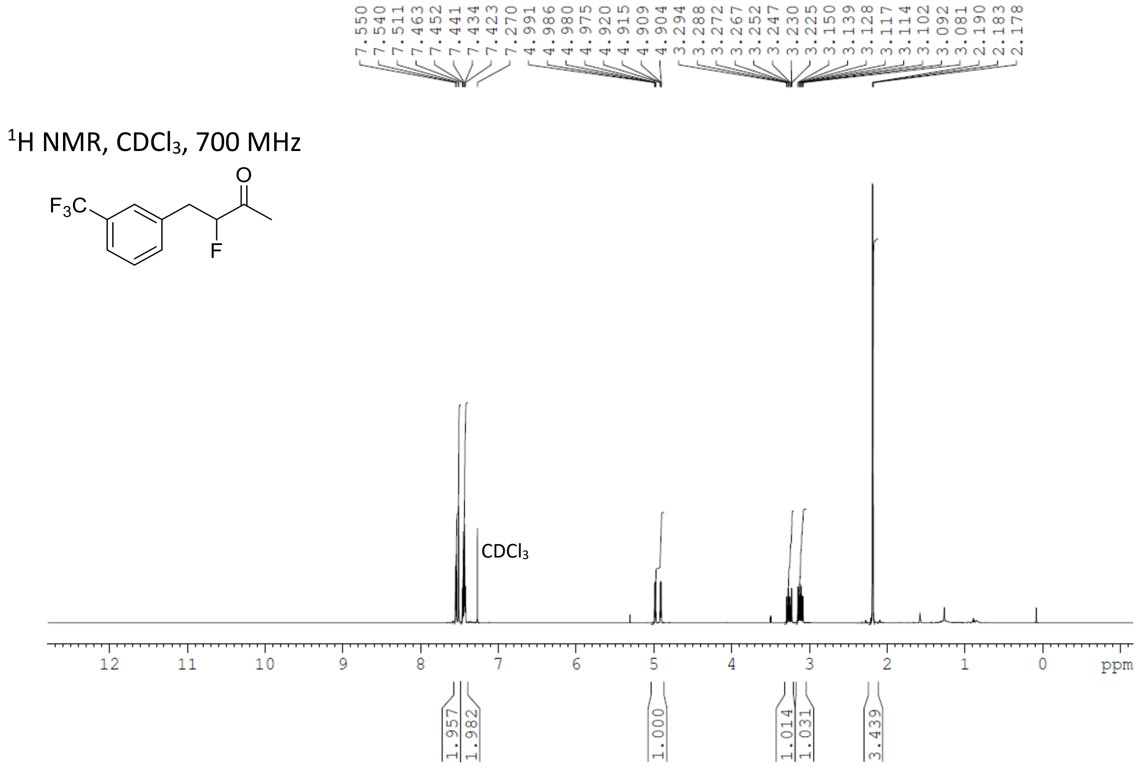


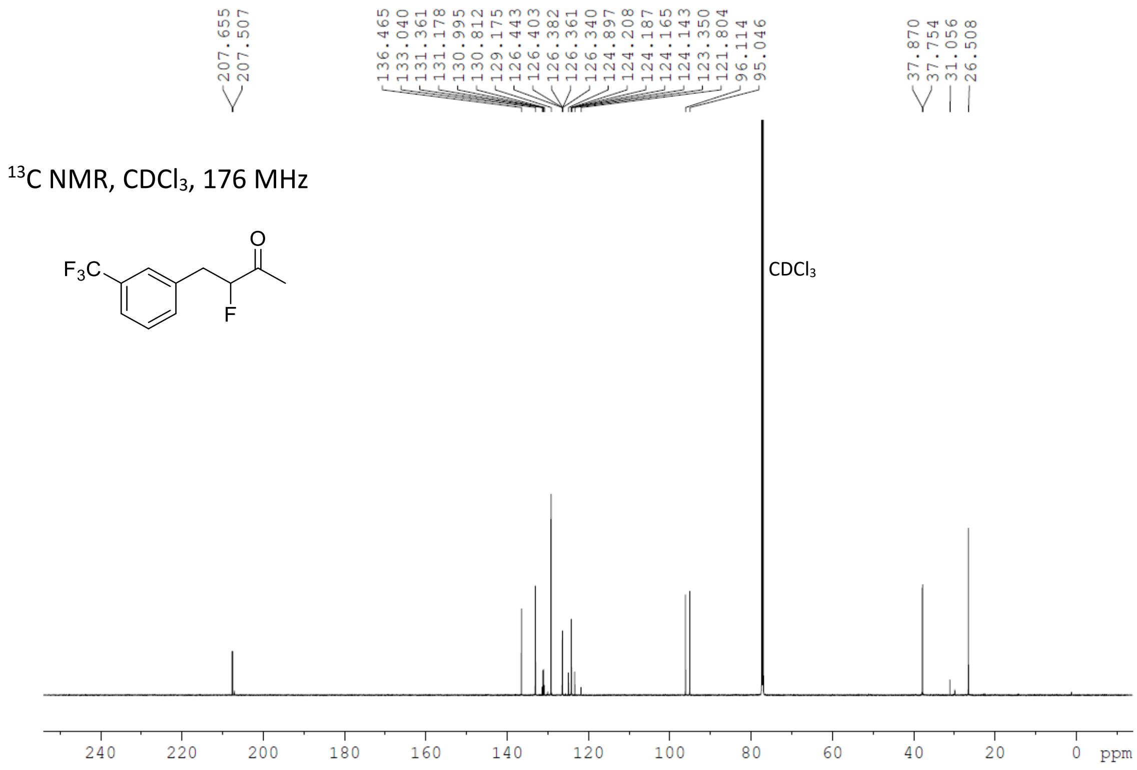

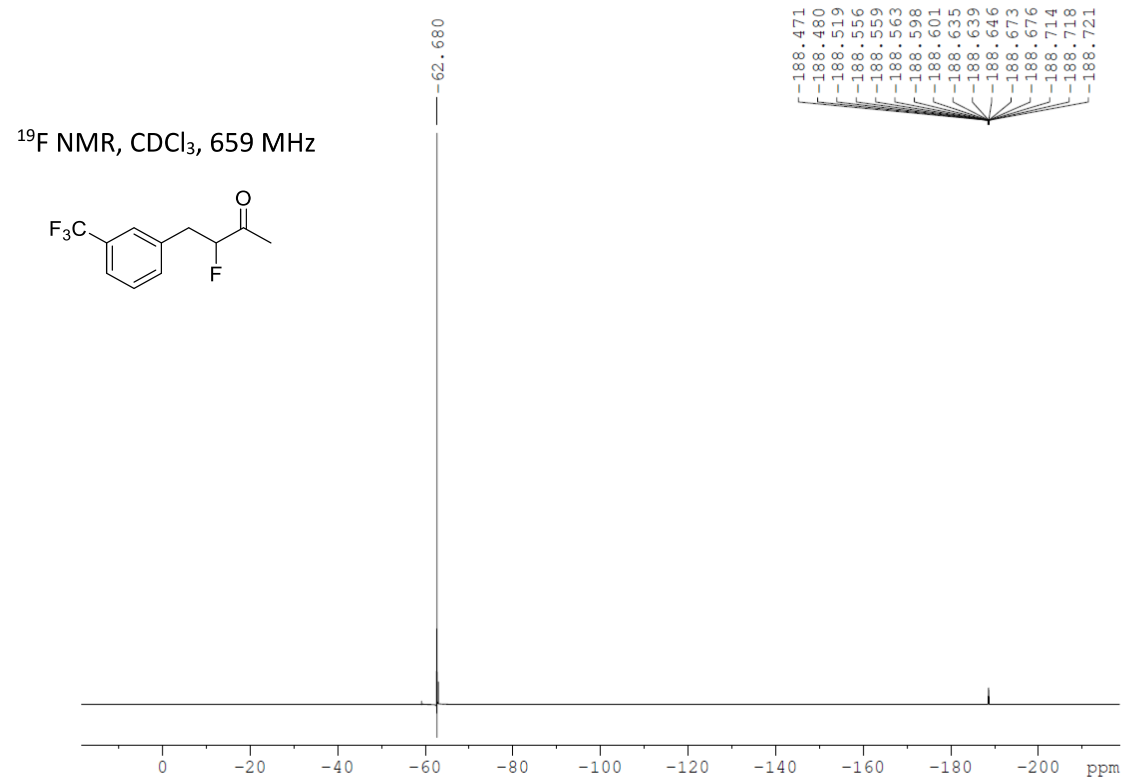


# 3-Fluoro-4-(pyridin-3-yl)butan-2-one (8a)

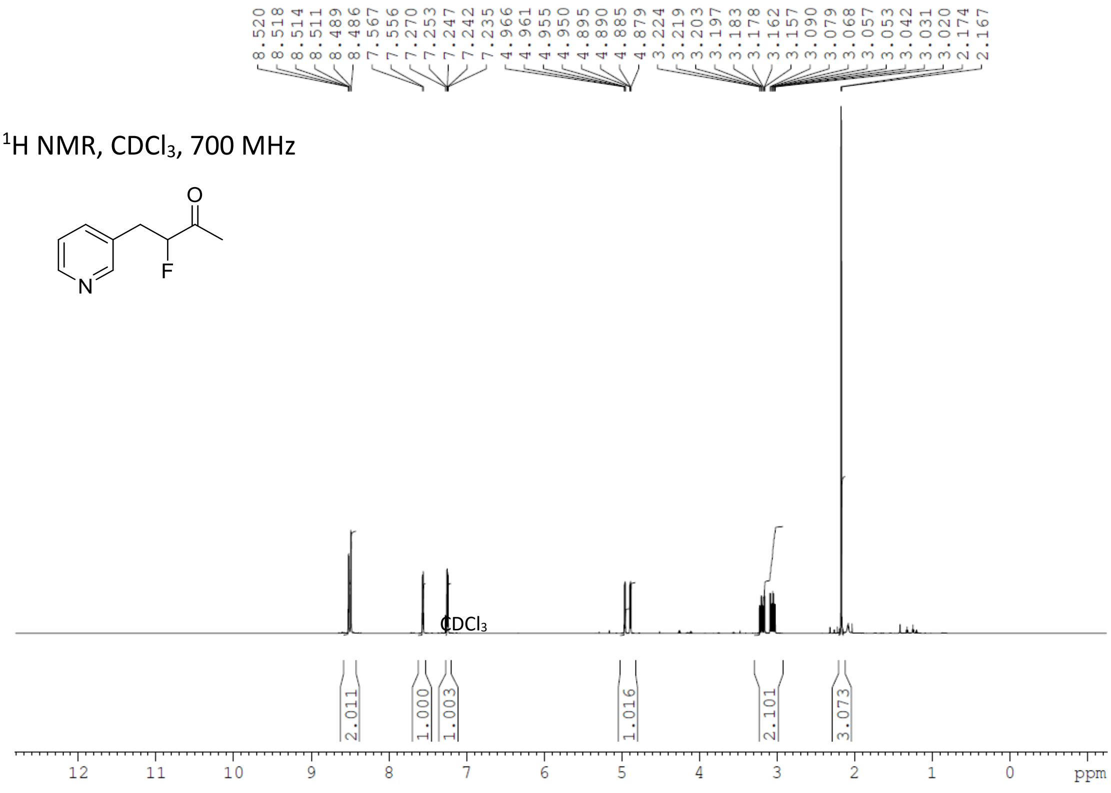

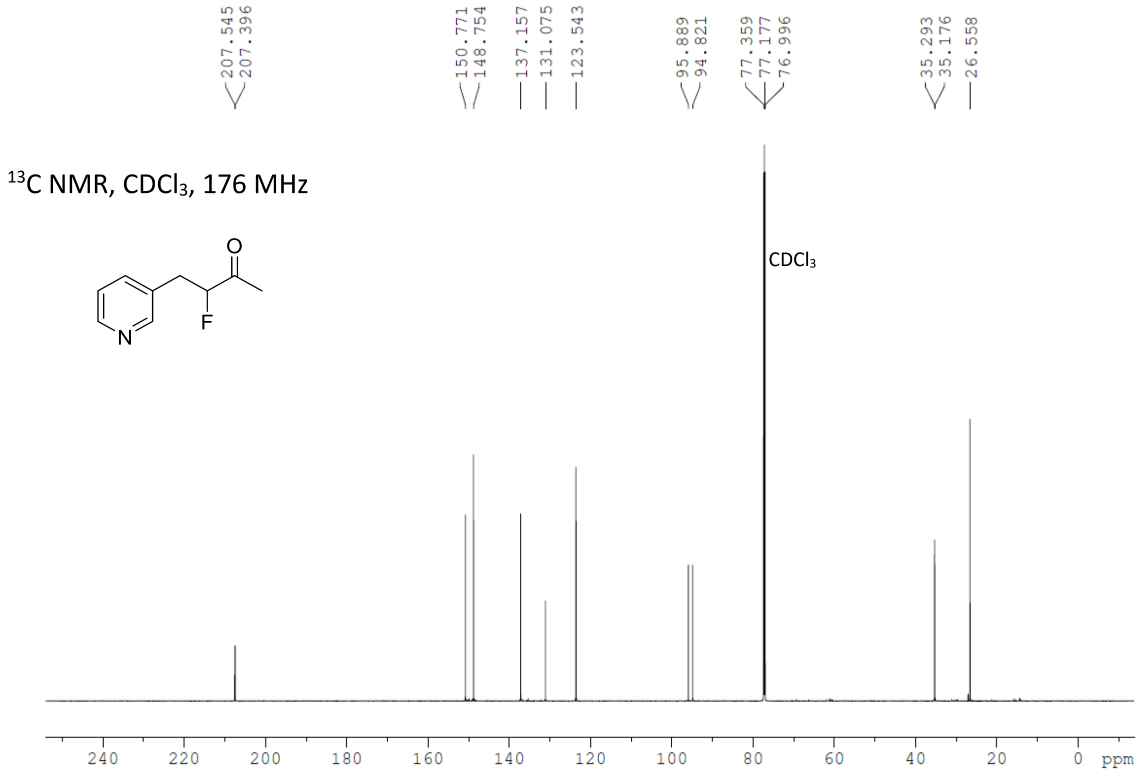
207.545
207.396
150.771
148.754
137.157
131.075
123.543
95.889
94.821
77.359
77.177
76.996
35.293
35.176
26.558
13C NMR, CDCl3, 176 MHz
CDCl3
240 220 200 180 160 140 120 100 80 60 40 20 0 ppm

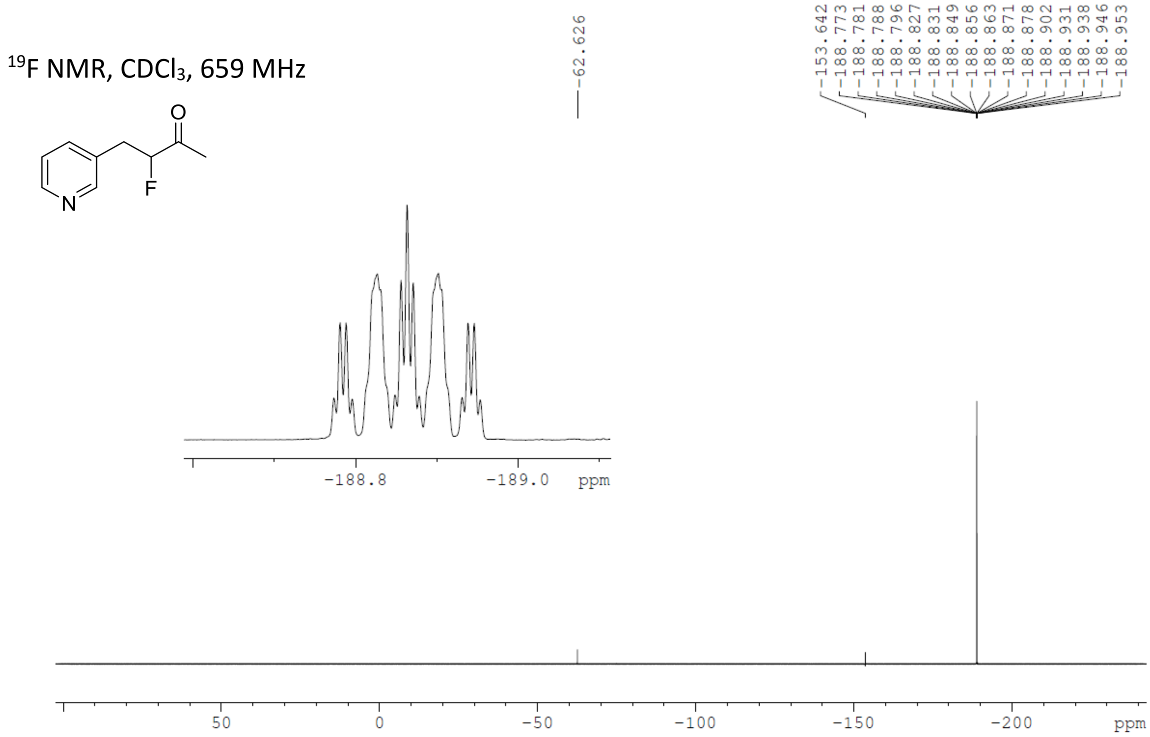
19F NMR, CDCl3, 659 MHz
-62.626
-153.642
-188.773
-188.781
-188.788
-188.796
-188.827
-188.831
-188.849
-188.856
-188.863
-188.871
-188.878
-188.902
-188.931
-188.938
-188.946
-188.953
-188.8 -189.0 ppm
50 0 -50 -100 -150 -200 ppm

## 3-Fluoro-4-(thiophen-2-yl)butan-2-one (8b)

$^{1}$H NMR, $CDCl_3$, 600 MHz

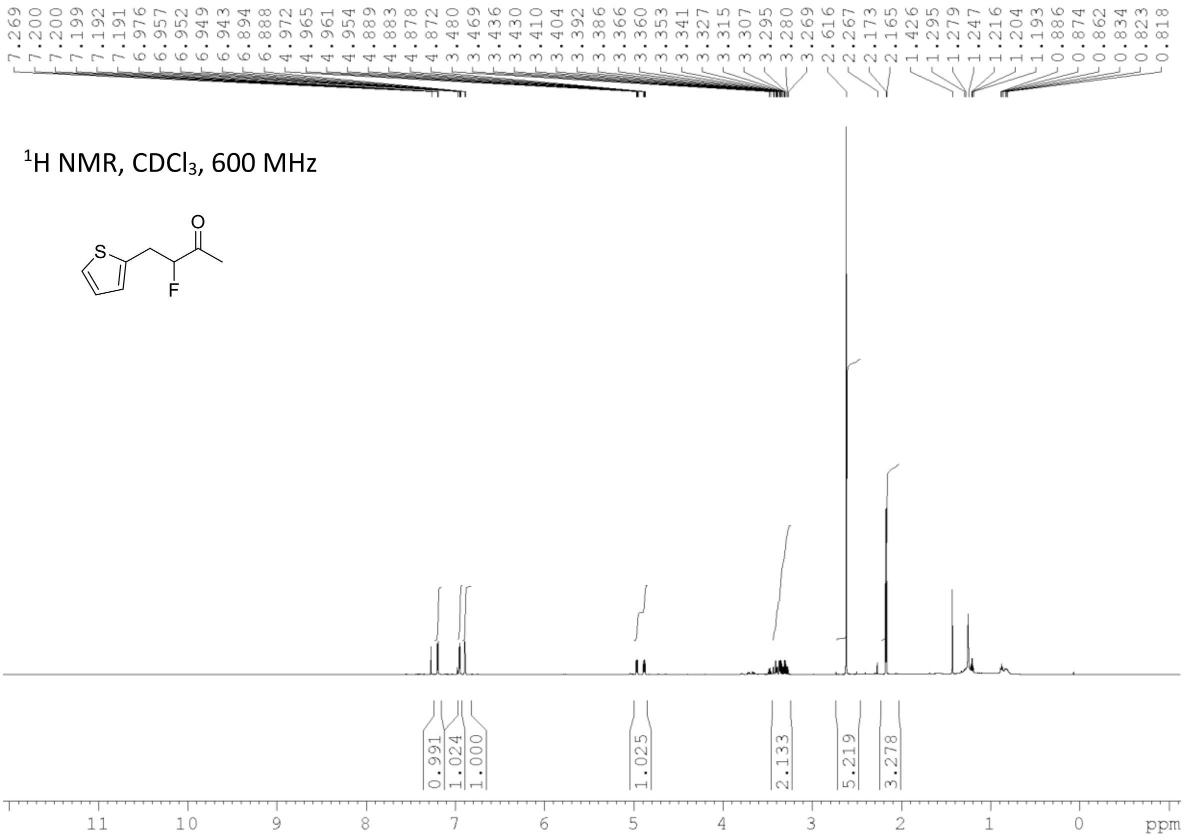


$^{13}$C NMR, $CDCl_3$, 151 MHz

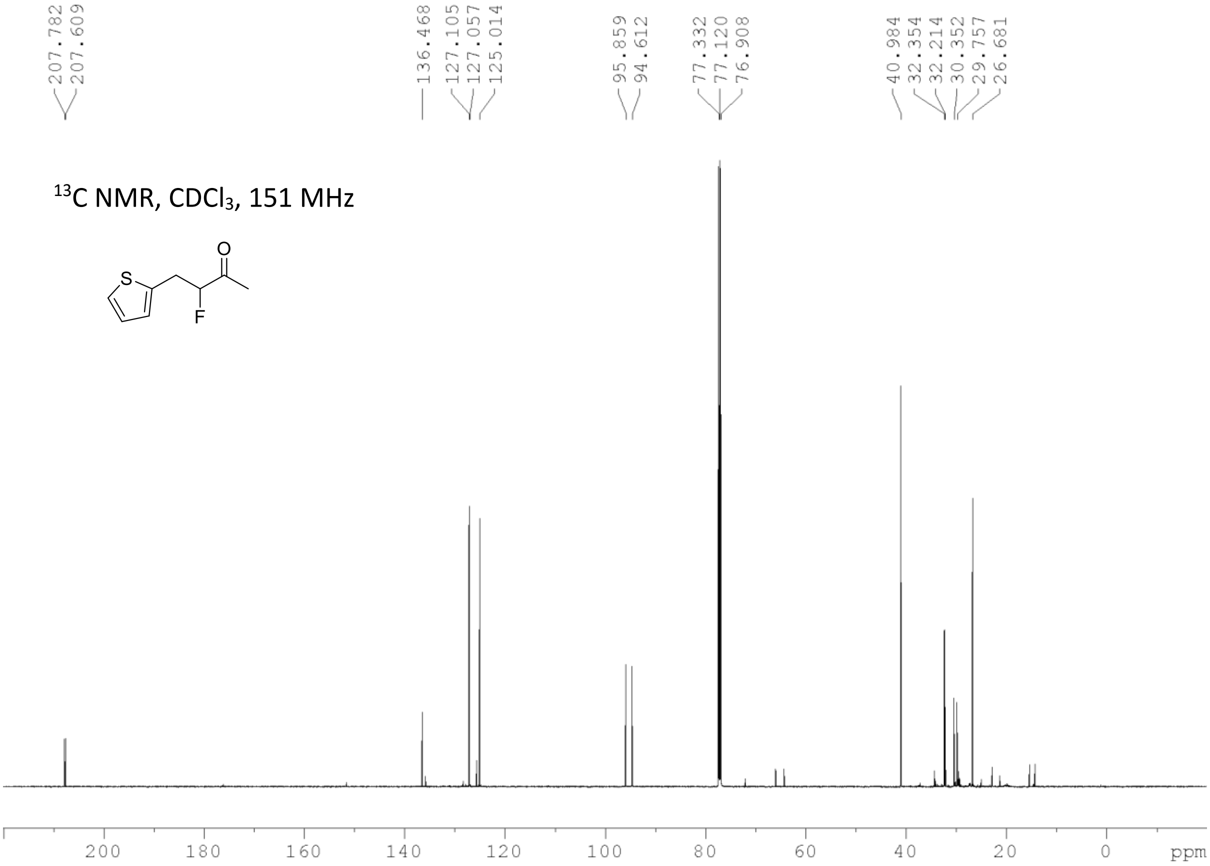




$^{19}$F NMR, $CDCl_3$, 379 MHz

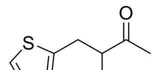

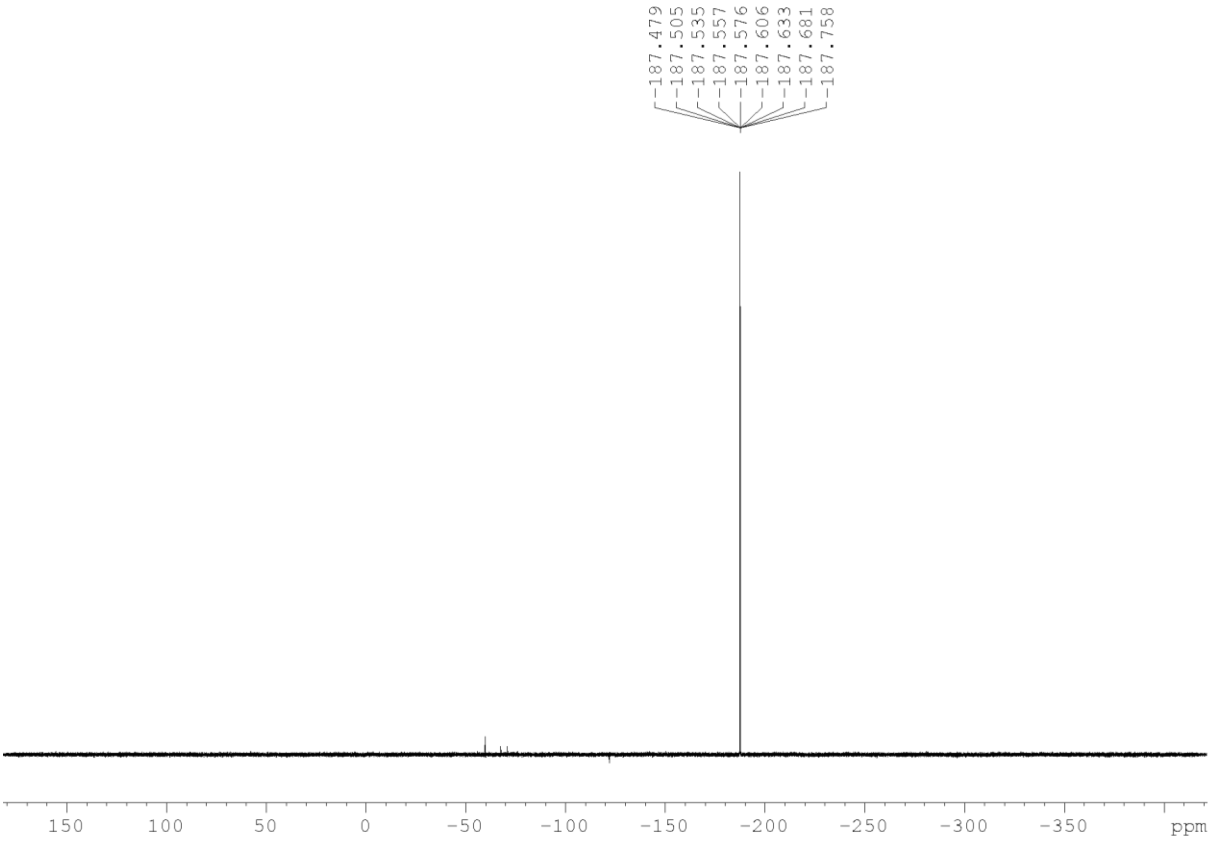


## 4-Cyclohexyl-3-fluorobutan-2-one (9a)

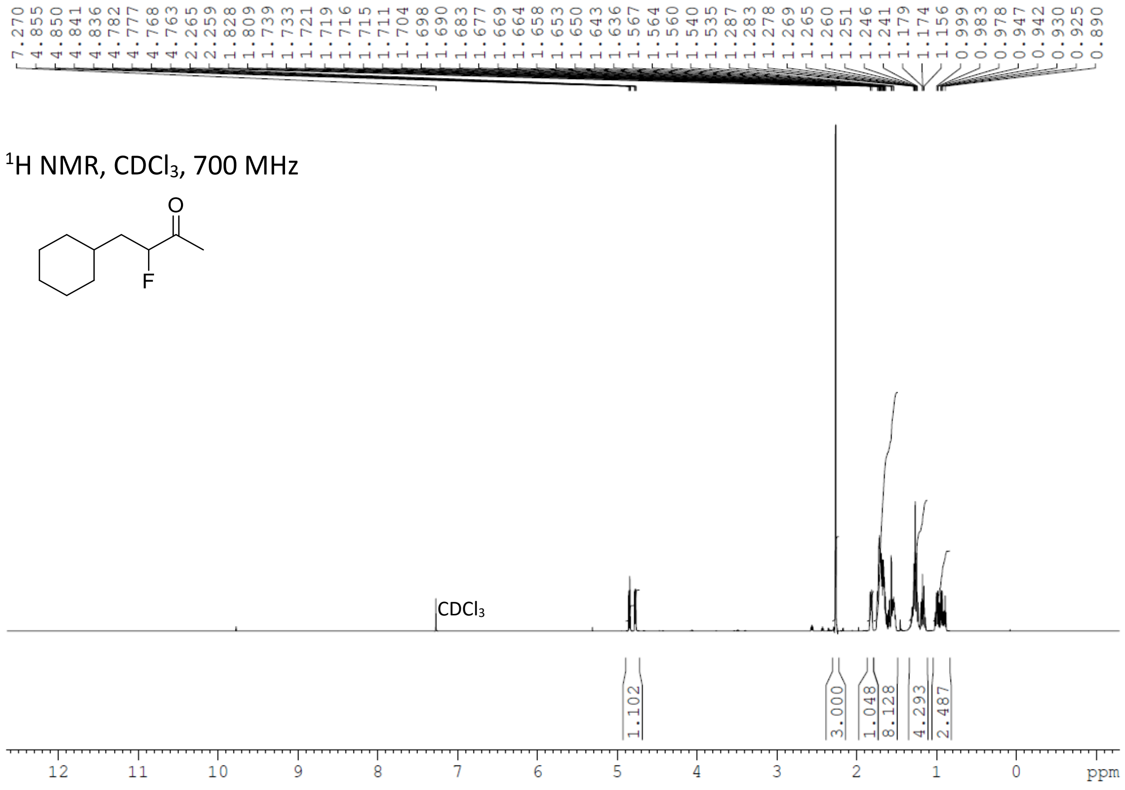

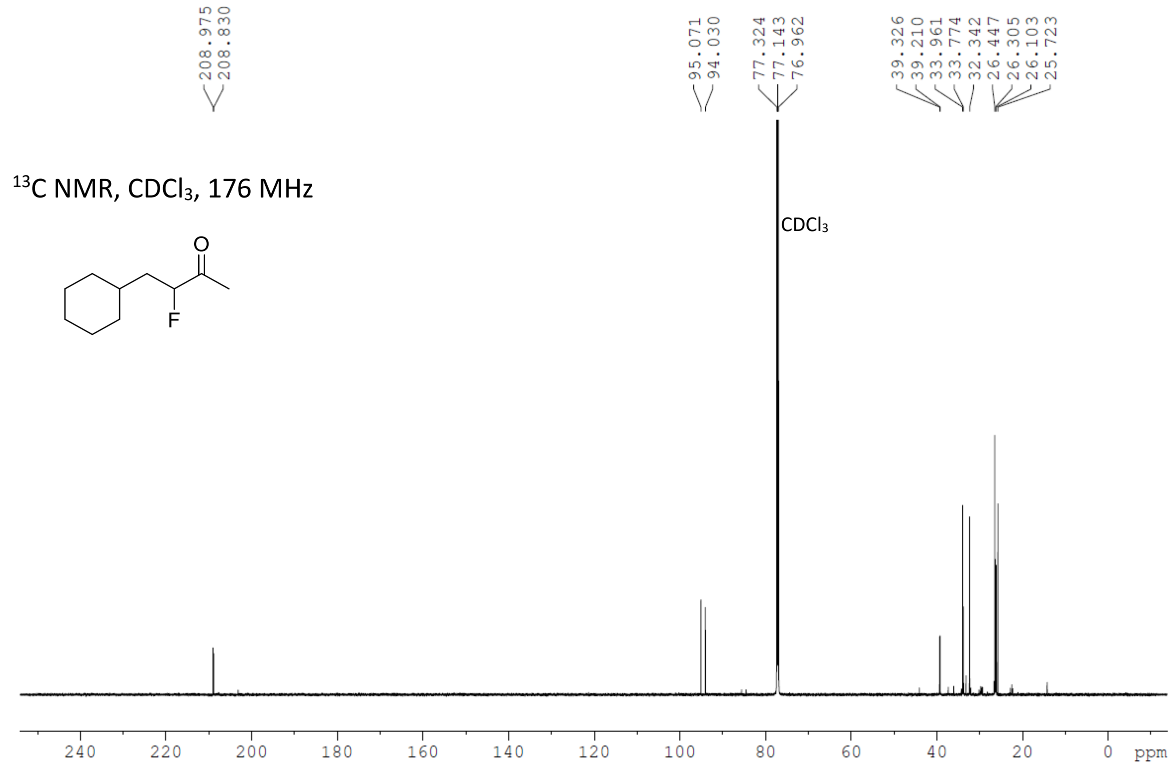

$^{13}$C NMR, $CDCl_3$, 176 MHz
208.975
208.830
95.071
94.030
77.324
77.143
76.962
39.326
39.210
33.961
33.774
32.342
26.447
26.305
26.103
25.723
$CDCl_3$
O
F
240 220 200 180 160 140 120 100 80 60 40 20 0 ppm


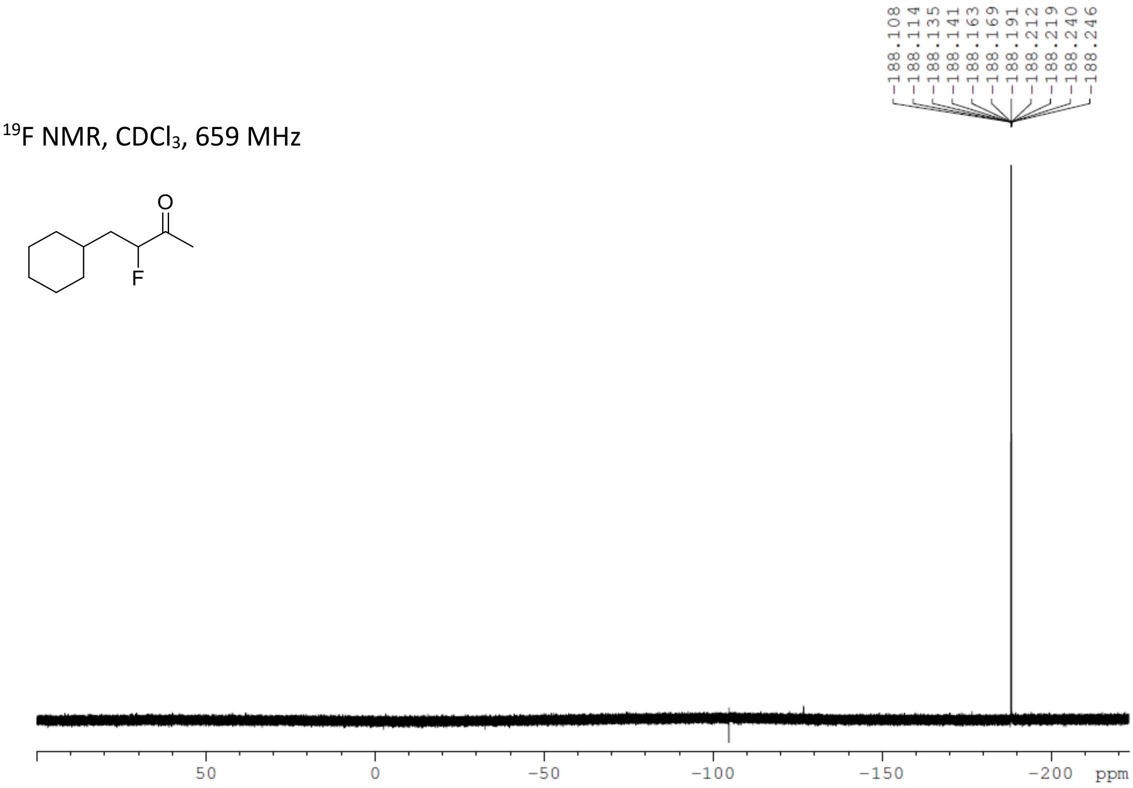

$^{19}$F NMR, $CDCl_3$, 659 MHz
-188.108
-188.114
-188.135
-188.141
-188.163
-188.169
-188.191
-188.212
-188.219
-188.240
-188.246
O
F
50 0 -50 -100 -150 -200 ppm

# 3-Fluoro-4-phenylbutan-2-one (9b)

$^{1}H$ NMR, $CDCl_3$, 700 MHz

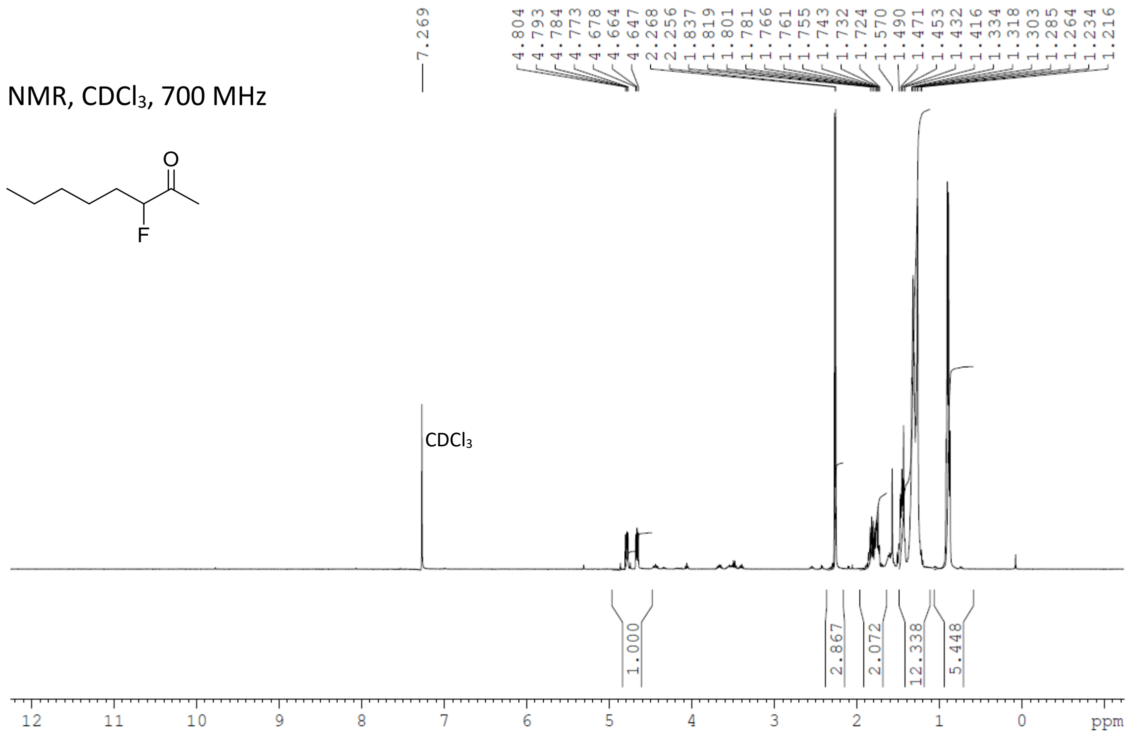


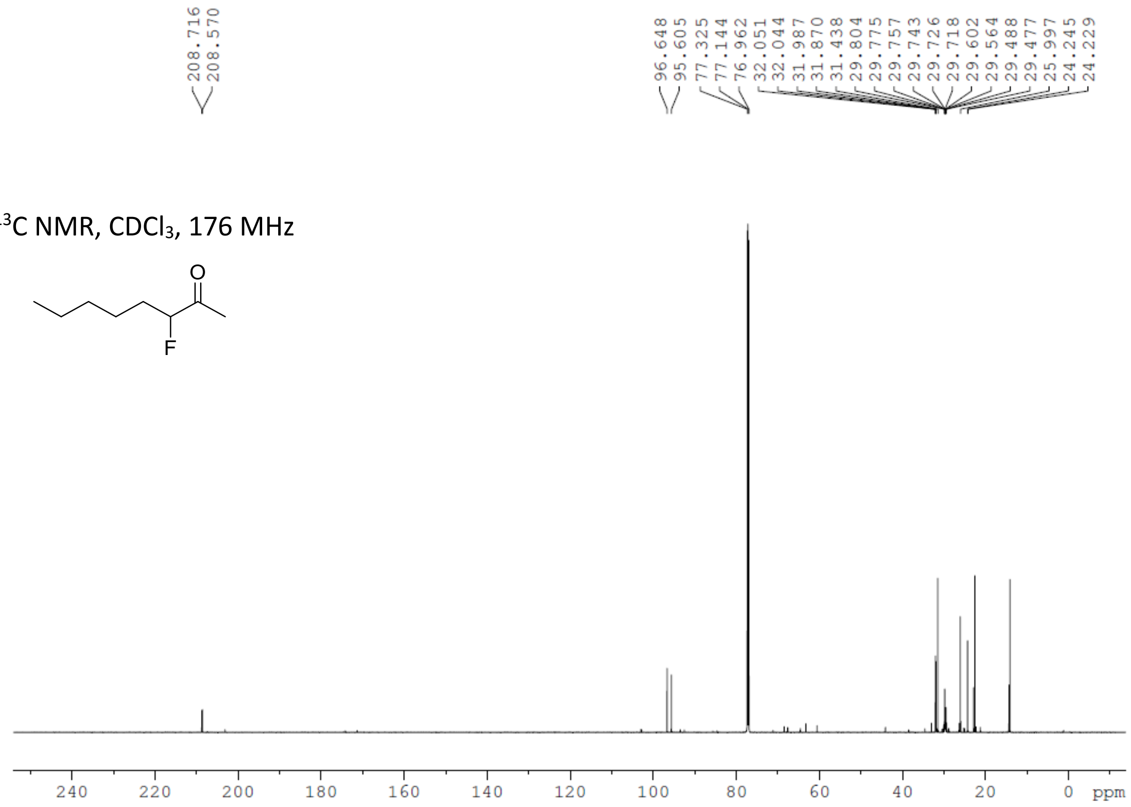


$^{13}C$ NMR, $CDCl_3$, 176 MHz

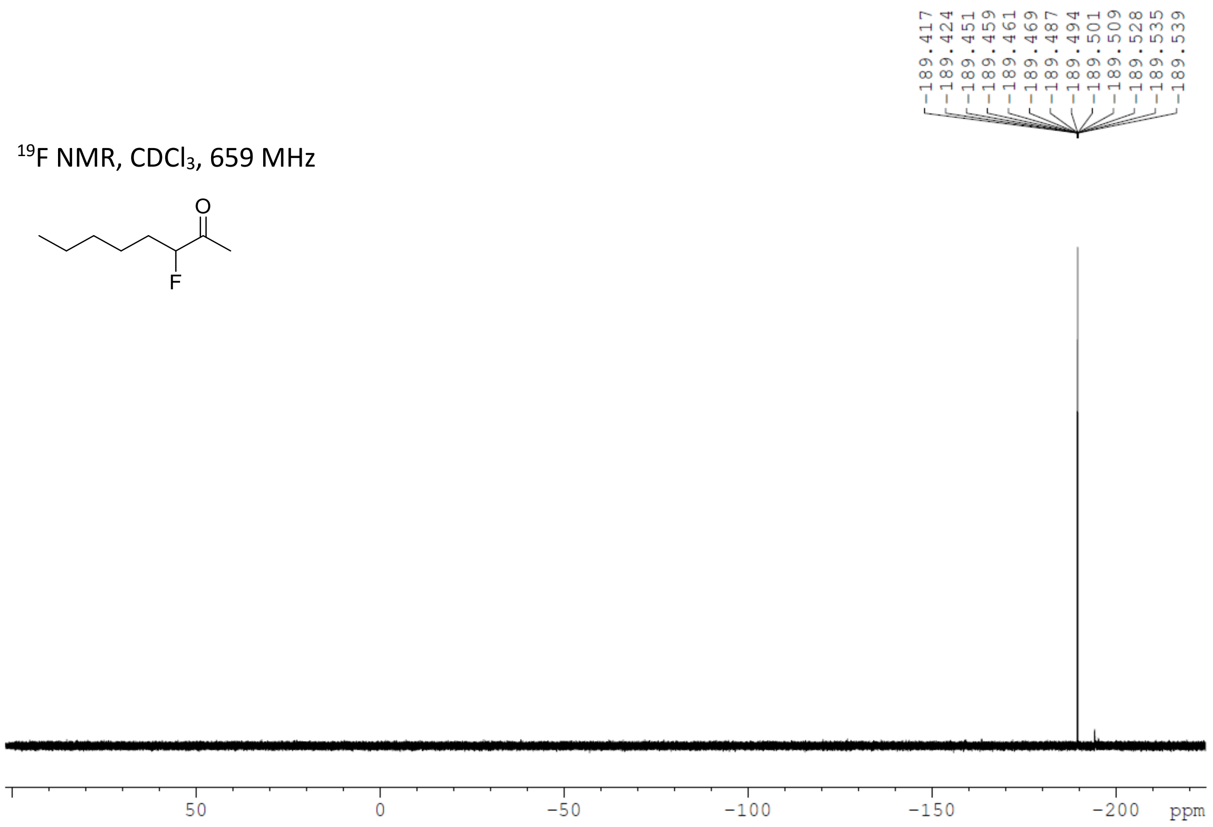


## 3-Fluoro-5-phenylpentan-2-one (9c)

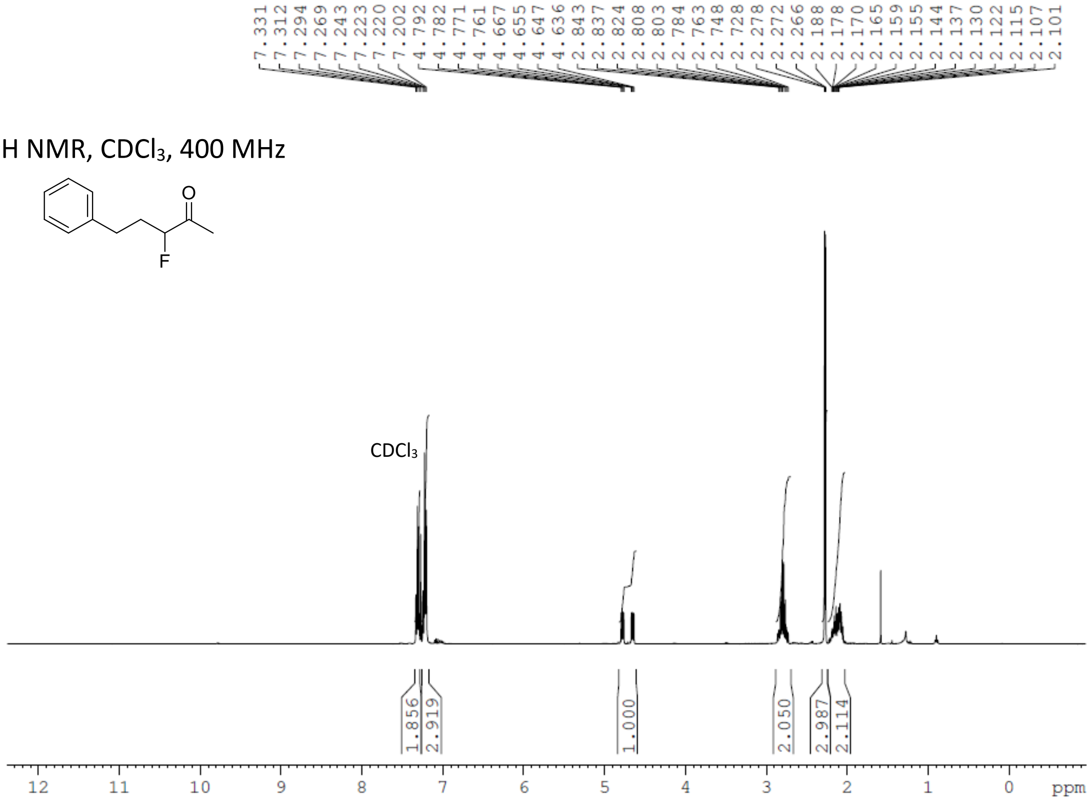

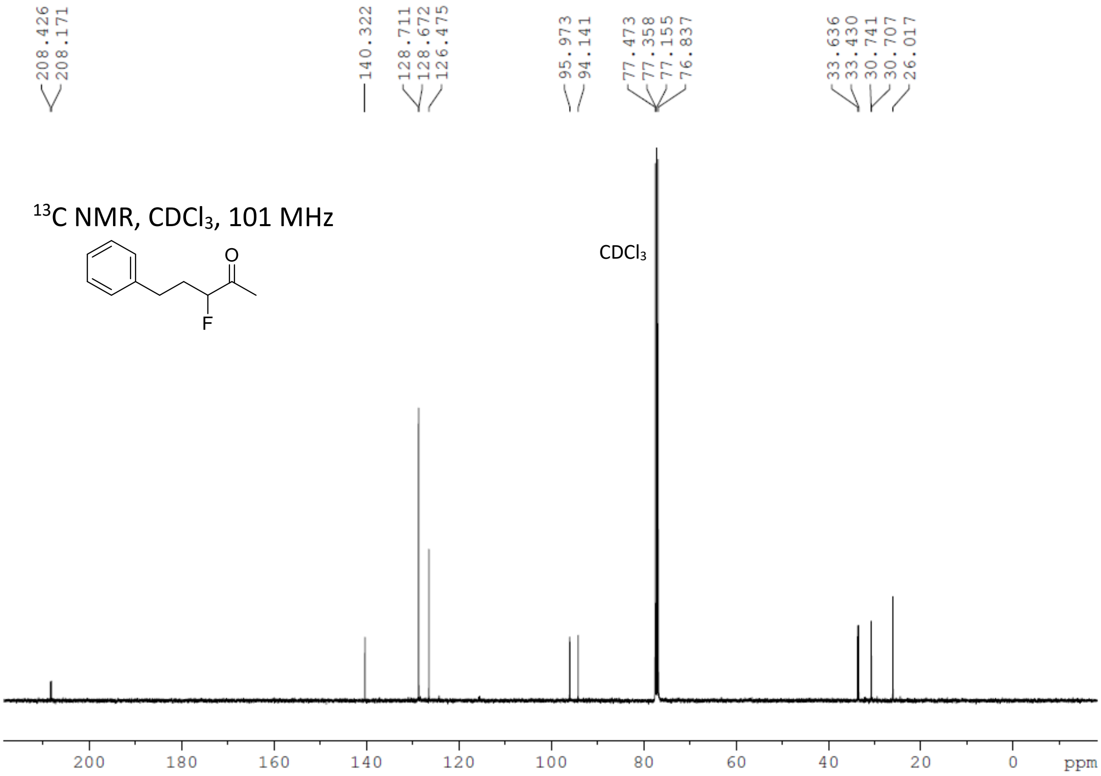
208.426
208.171
140.322
128.711
128.672
126.475
95.973
94.141
77.473
77.358
77.155
76.837
33.636
33.430
30.741
30.707
26.017
13C NMR, CDCl3, 101 MHz
O
F
CDCl3
200
180
160
140
120
100
80
60
40
20
0
ppm

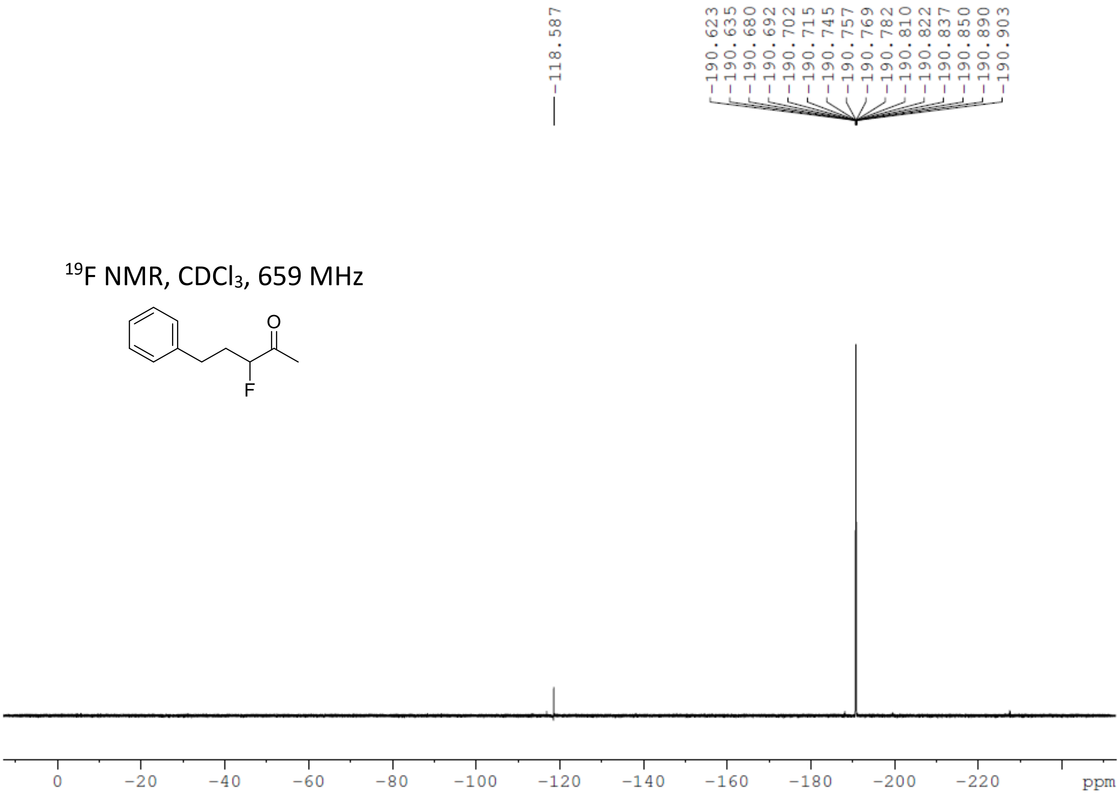
-118.587
-190.623
-190.635
-190.680
-190.692
-190.702
-190.715
-190.745
-190.757
-190.769
-190.782
-190.810
-190.822
-190.837
-190.850
-190.890
-190.903
19F NMR, CDCl3, 659 MHz
O
F
0
-20
-40
-60
-80
-100
-120
-140
-160
-180
-200
-220
ppm

# 3-Fluoro-6-phenylhexan-2-one (9d)

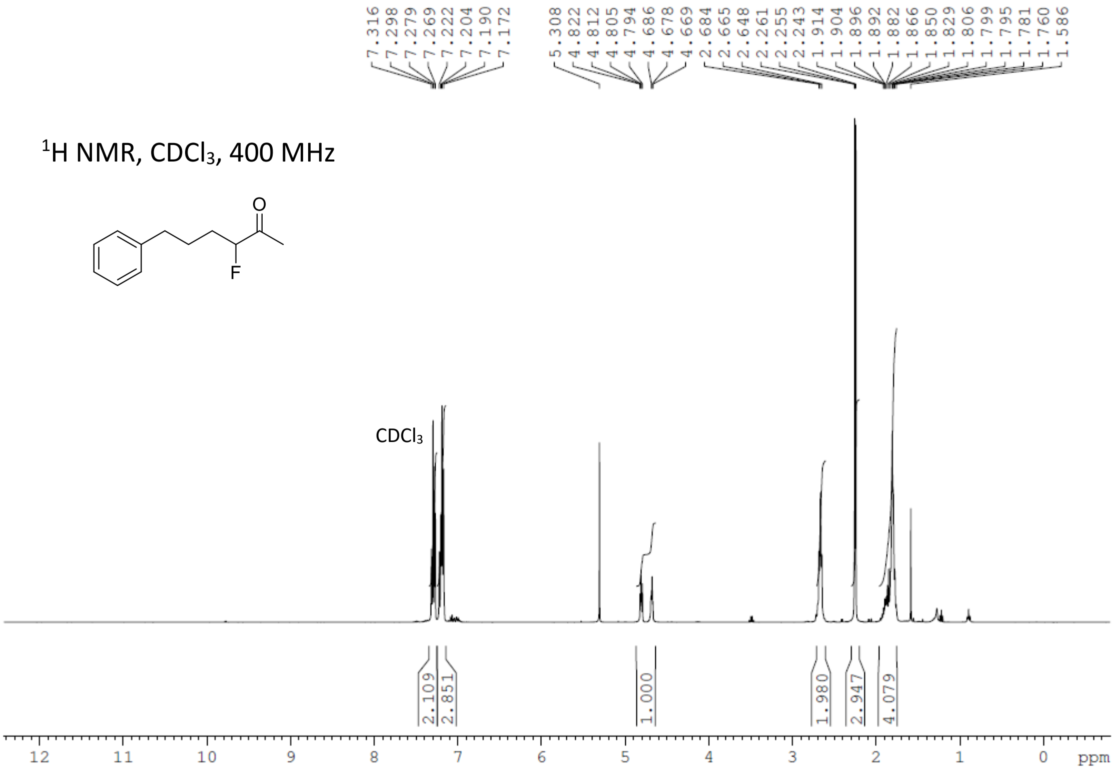


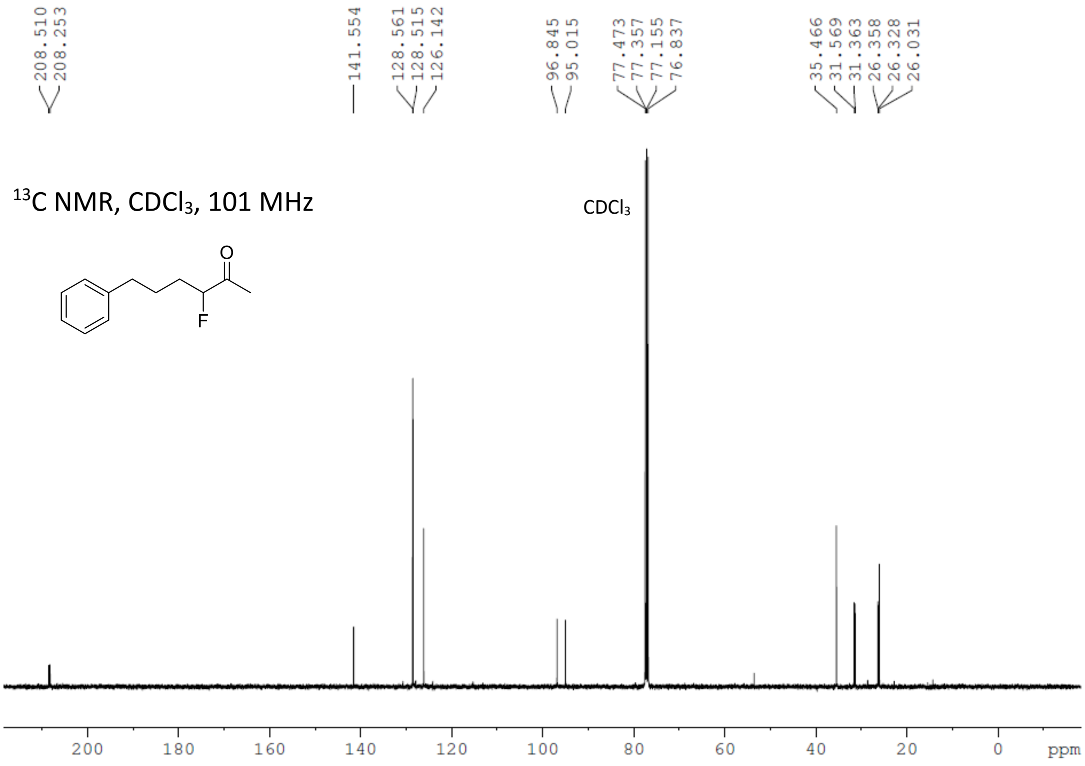

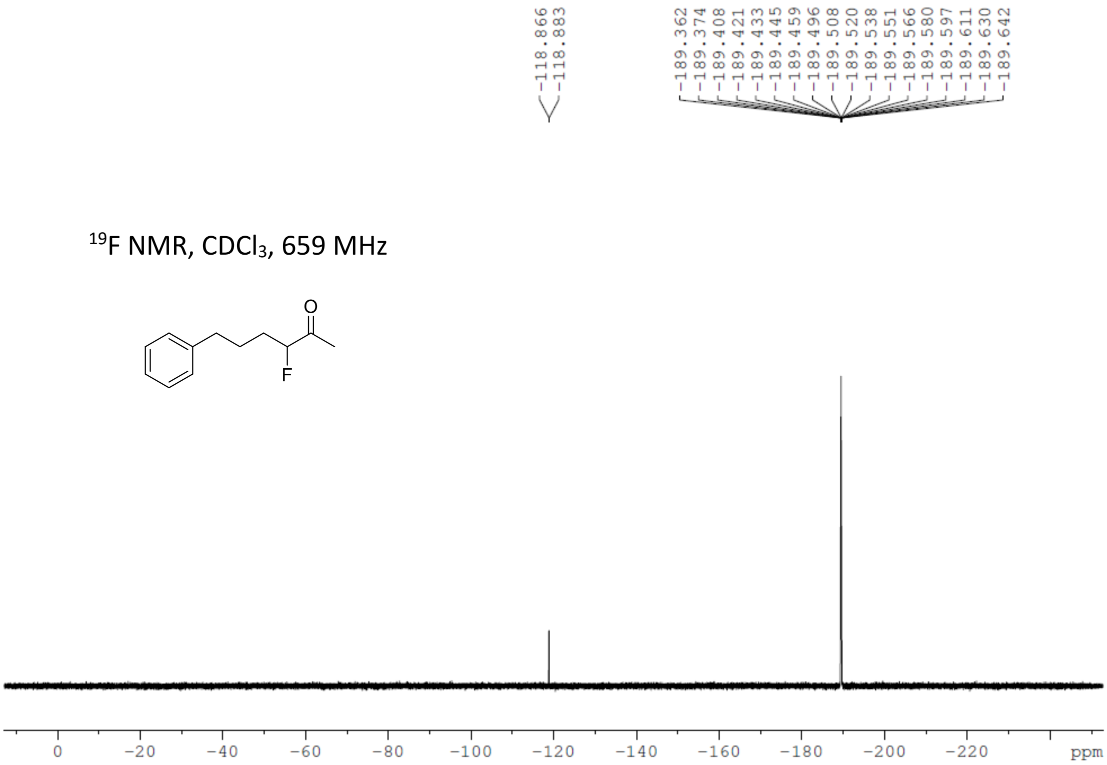


## 2-Fluorocyclohexan-1-one (10a)

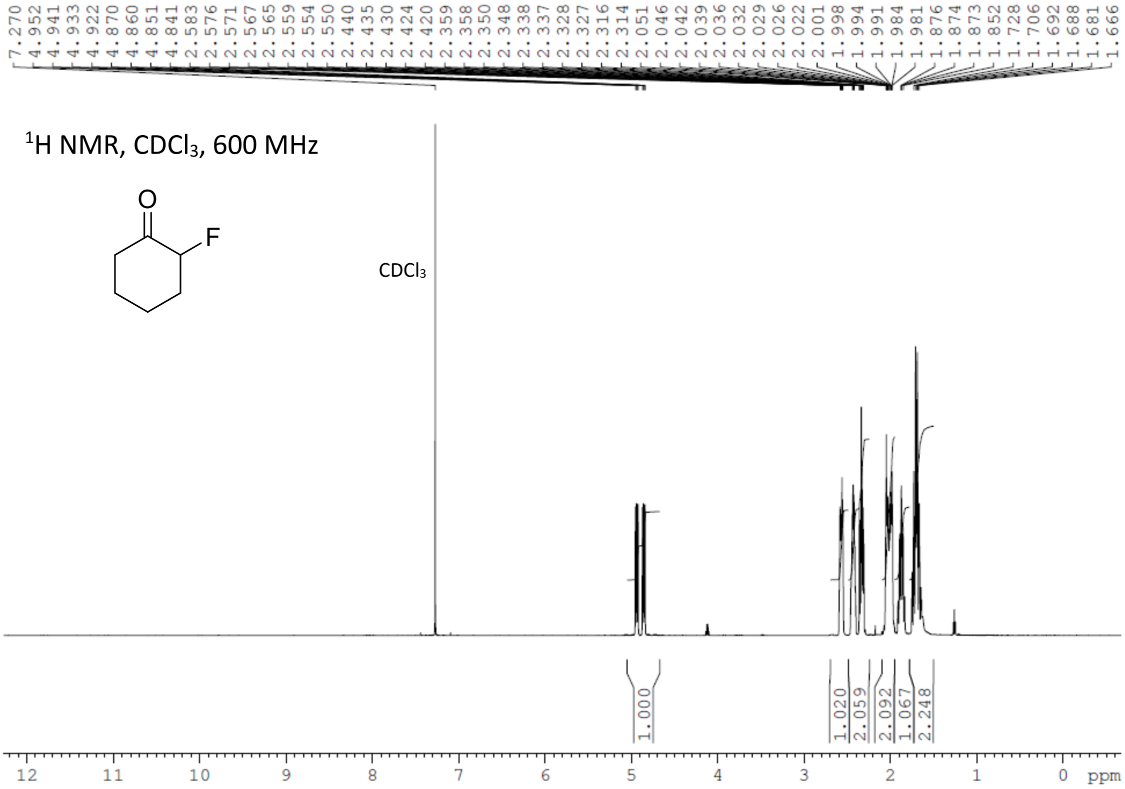

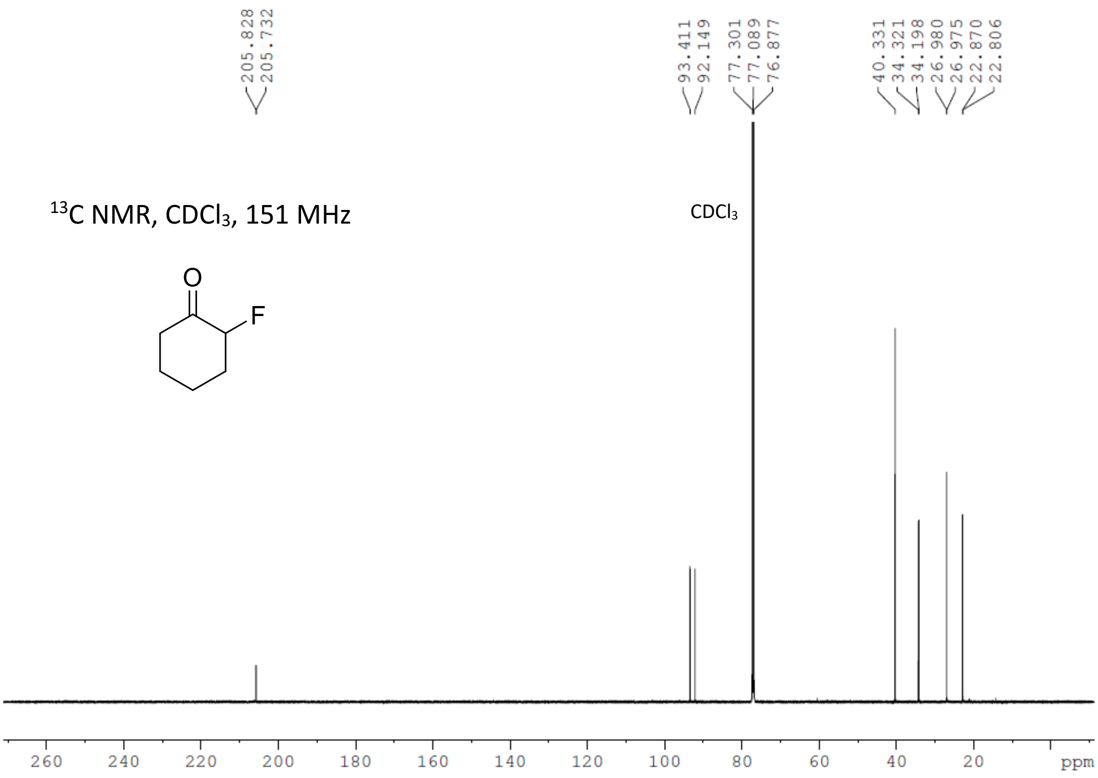
205.828
205.732
93.411
92.149
77.301
77.089
76.877
40.331
34.321
34.198
26.980
26.975
22.870
22.806
13C NMR, CDCl3, 151 MHz
O
F
CDCl3
260 240 220 200 180 160 140 120 100 80 60 40 20 ppm

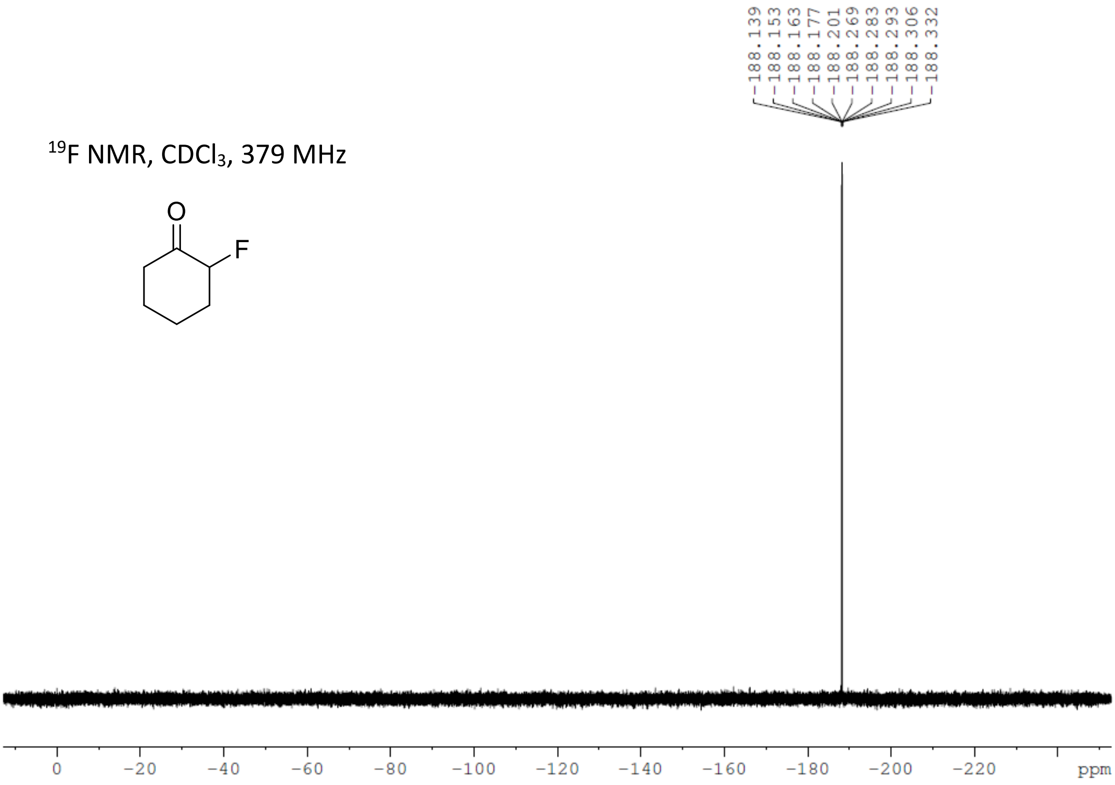
-188.139
-188.153
-188.163
-188.177
-188.201
-188.269
-188.283
-188.293
-188.306
-188.332
19F NMR, CDCl3, 379 MHz
O
F
0 -20 -40 -60 -80 -100 -120 -140 -160 -180 -200 -220 ppm

## 2-Fluorocycloheptan-1-one (10b)

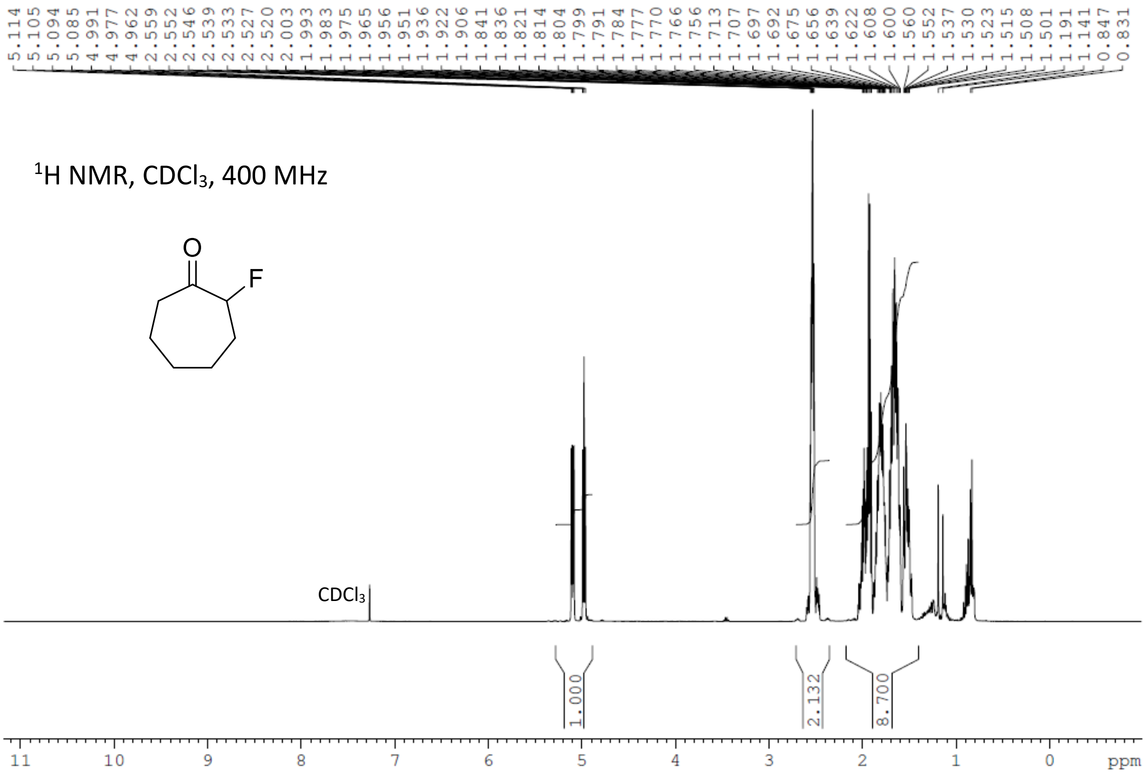


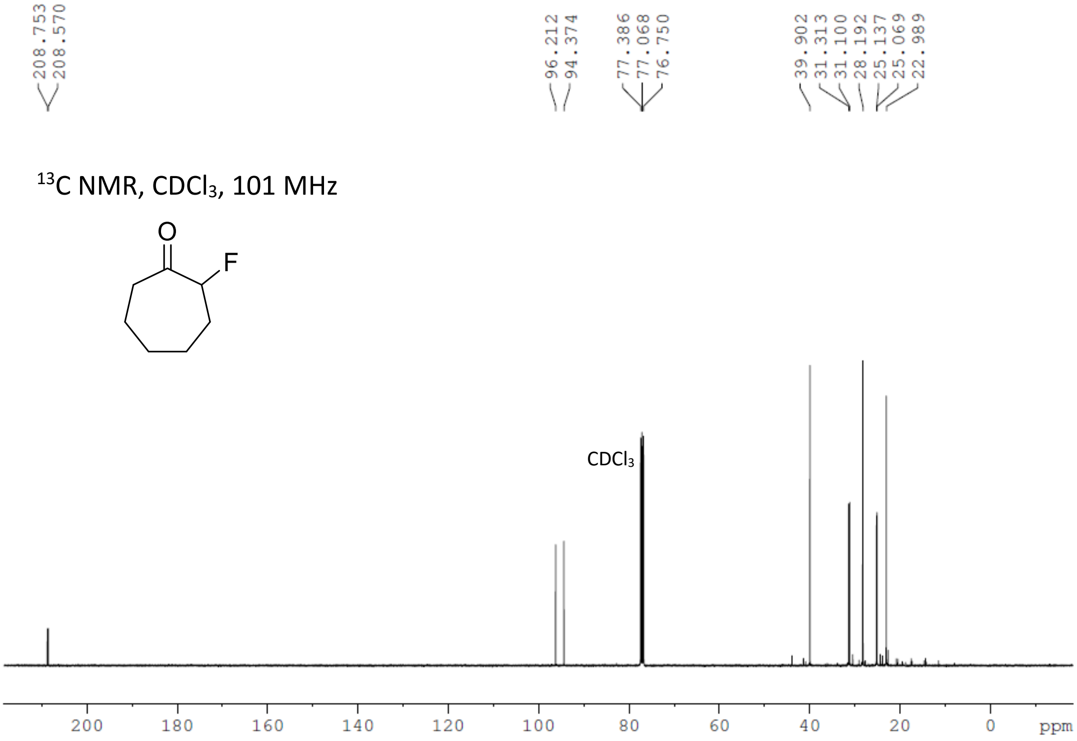

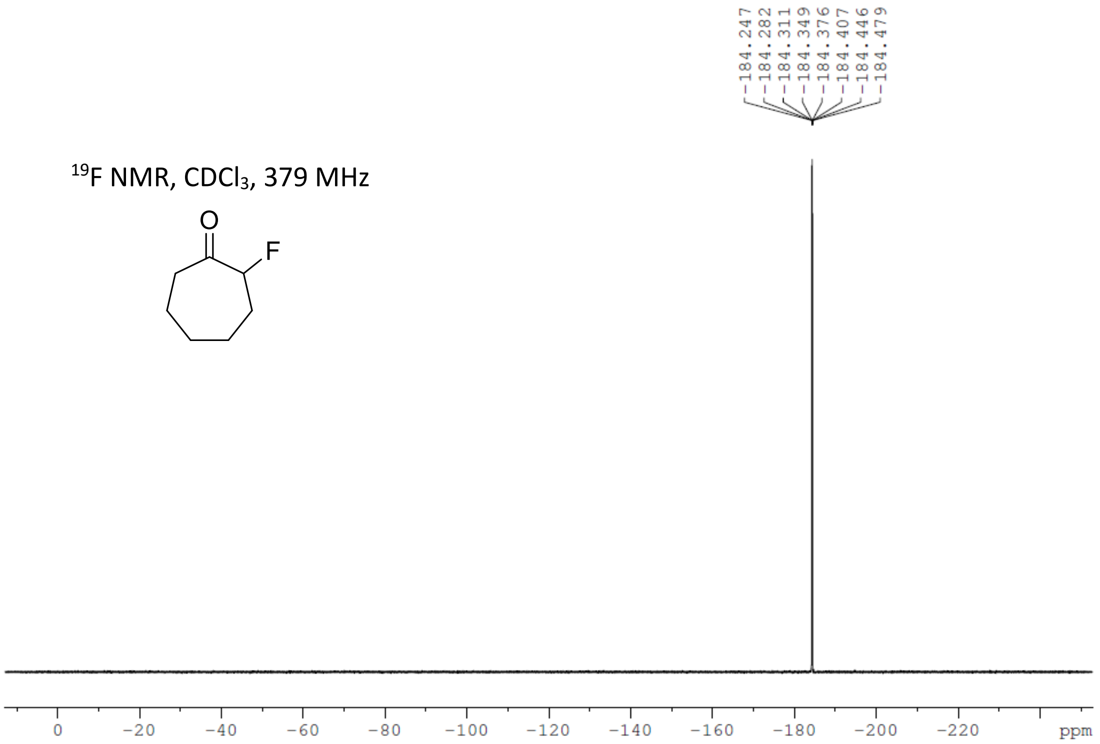


## 2-Fluoro-1-phenylpentan-3-one (11a)

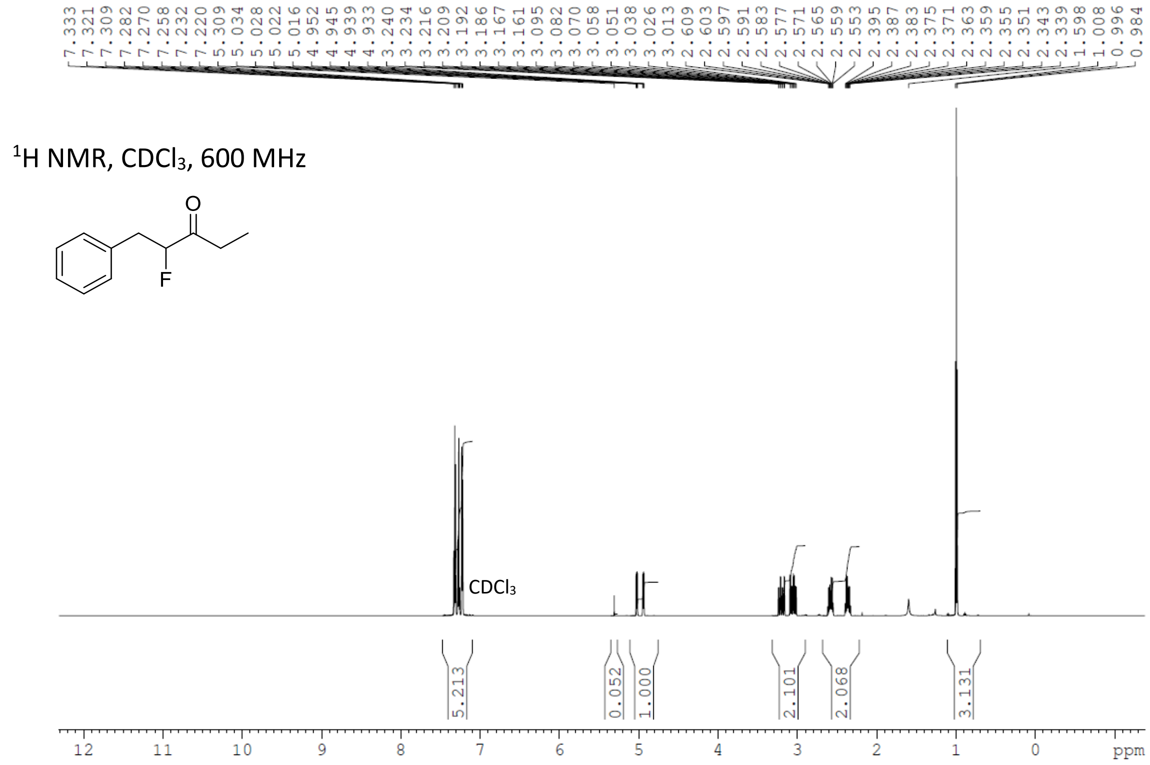

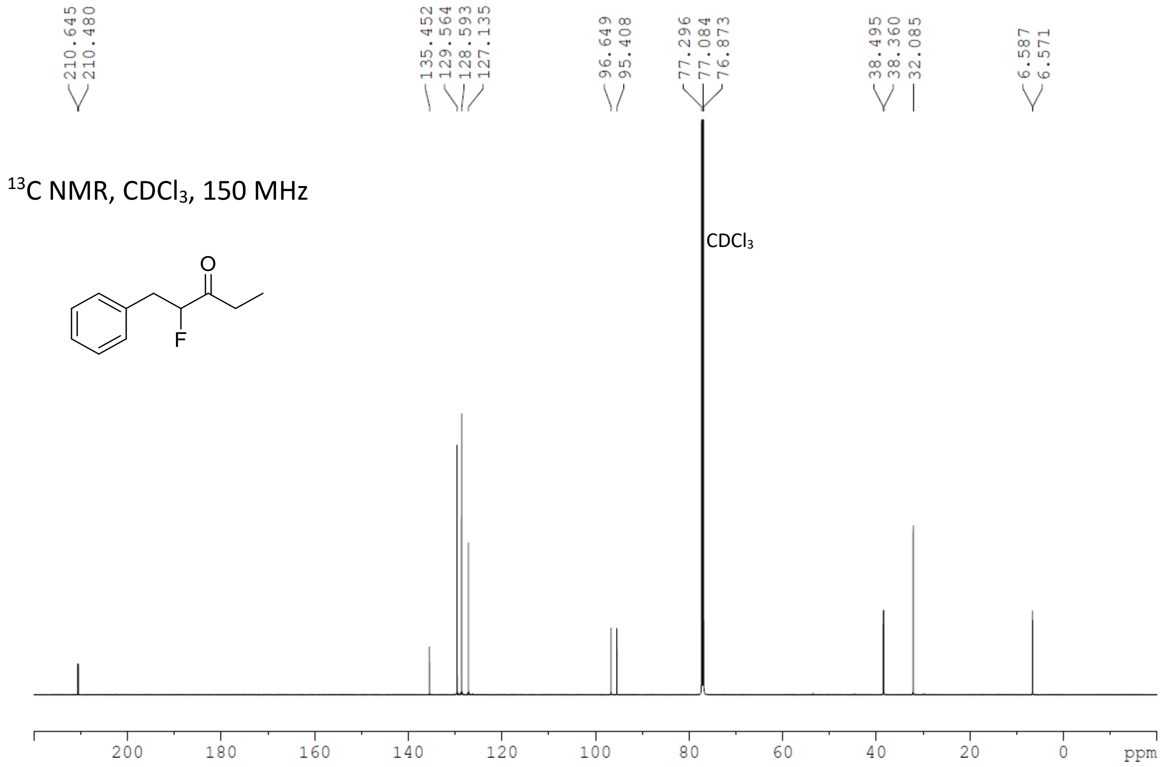
210.645
210.480
135.452
129.564
128.593
127.135
96.649
95.408
77.296
77.084
76.873
38.495
38.360
32.085
6.587
6.571
13C NMR, CDCl3, 150 MHz
O
F
CDCl3
200
180
160
140
120
100
80
60
40
20
0
ppm

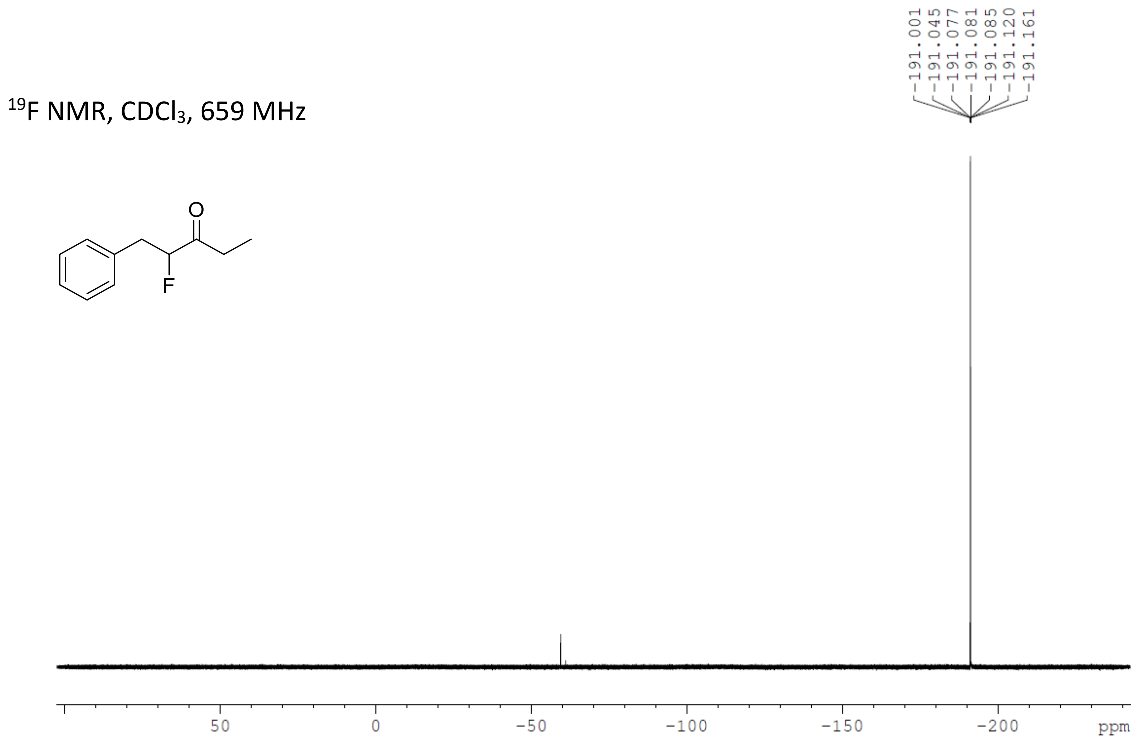
-191.001
-191.045
-191.077
-191.081
-191.085
-191.120
-191.161
19F NMR, CDCl3, 659 MHz
O
F
50
0
-50
-100
-150
-200
ppm

## 2-Fluoro-1-phenylheptan-1-one (11b)

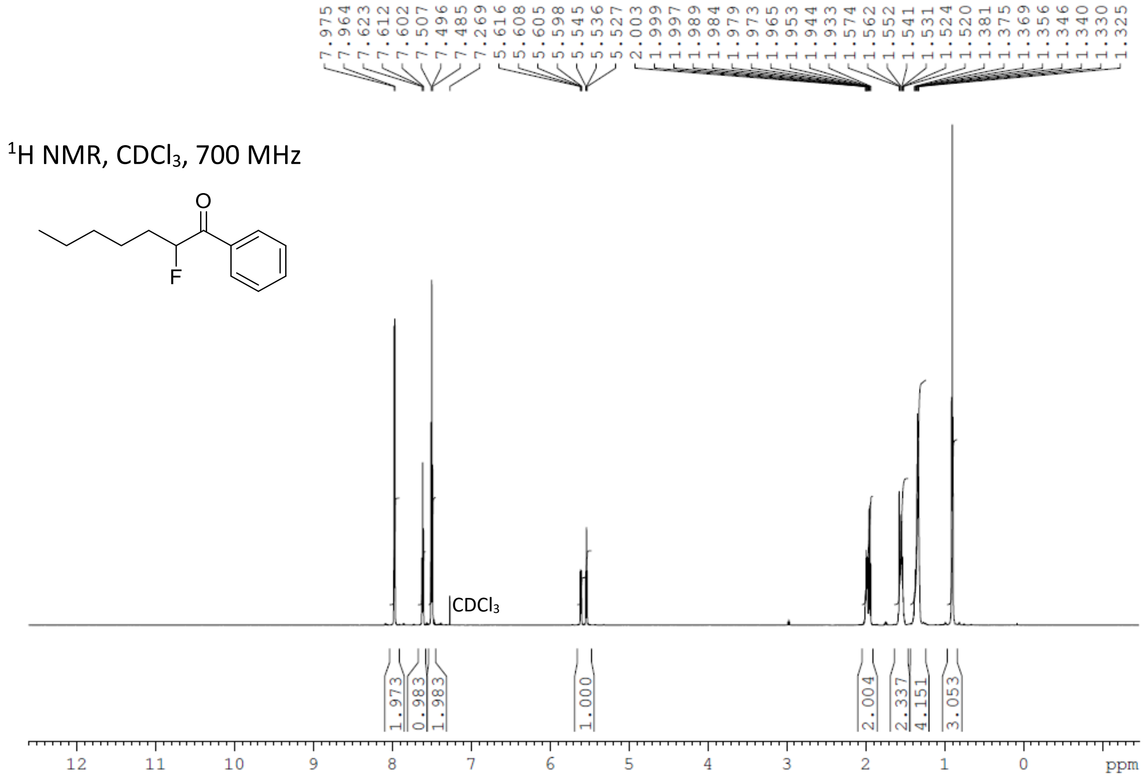


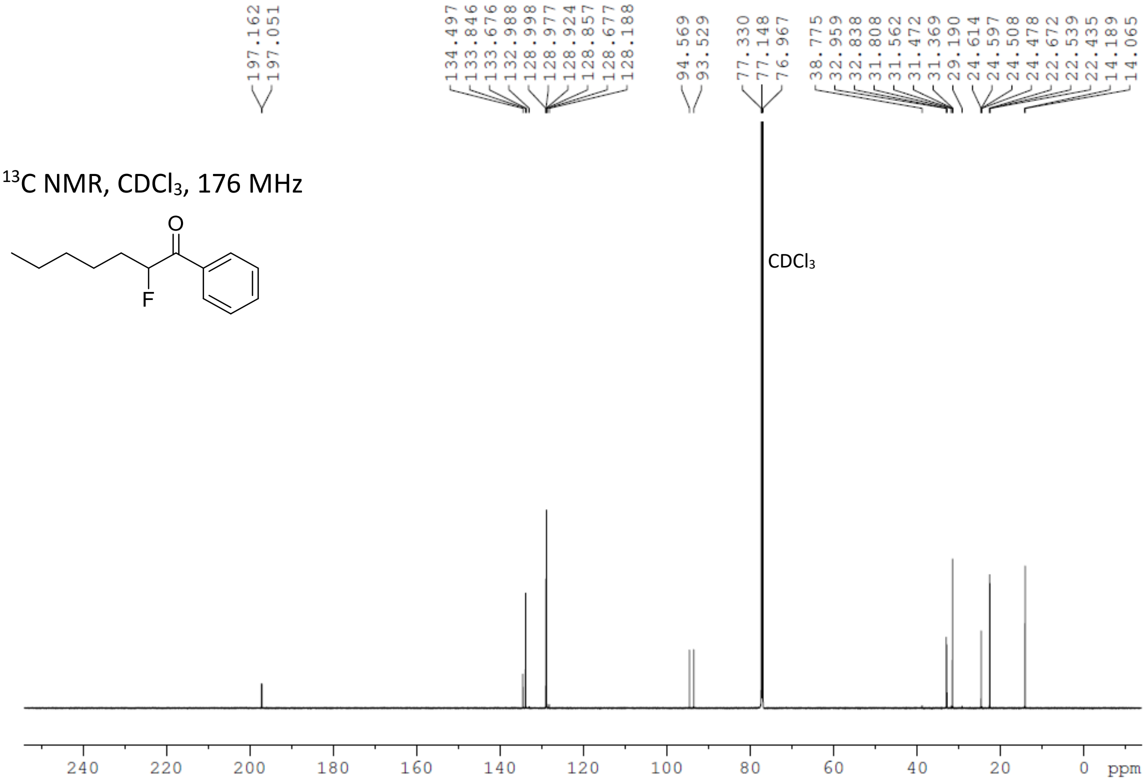

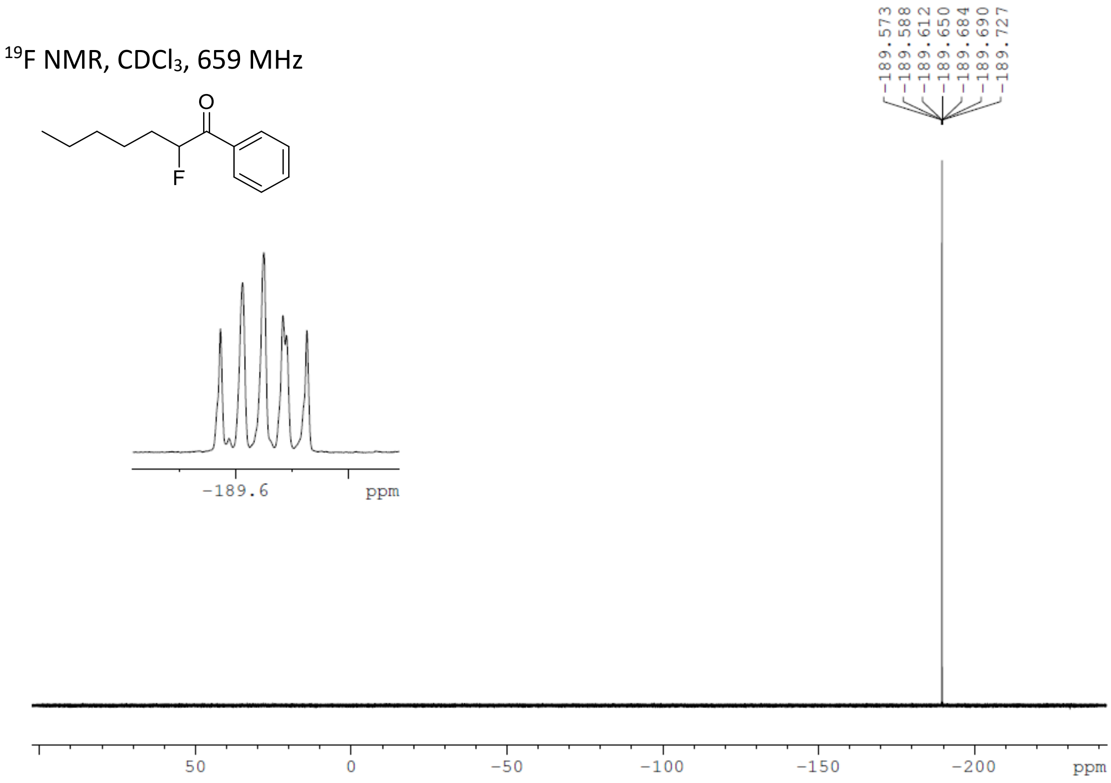


## (3*S*,4*R*)-3-Fluoro-4-phenylbutan-2-one-4-d ((3*R*,4*S*)-7a)

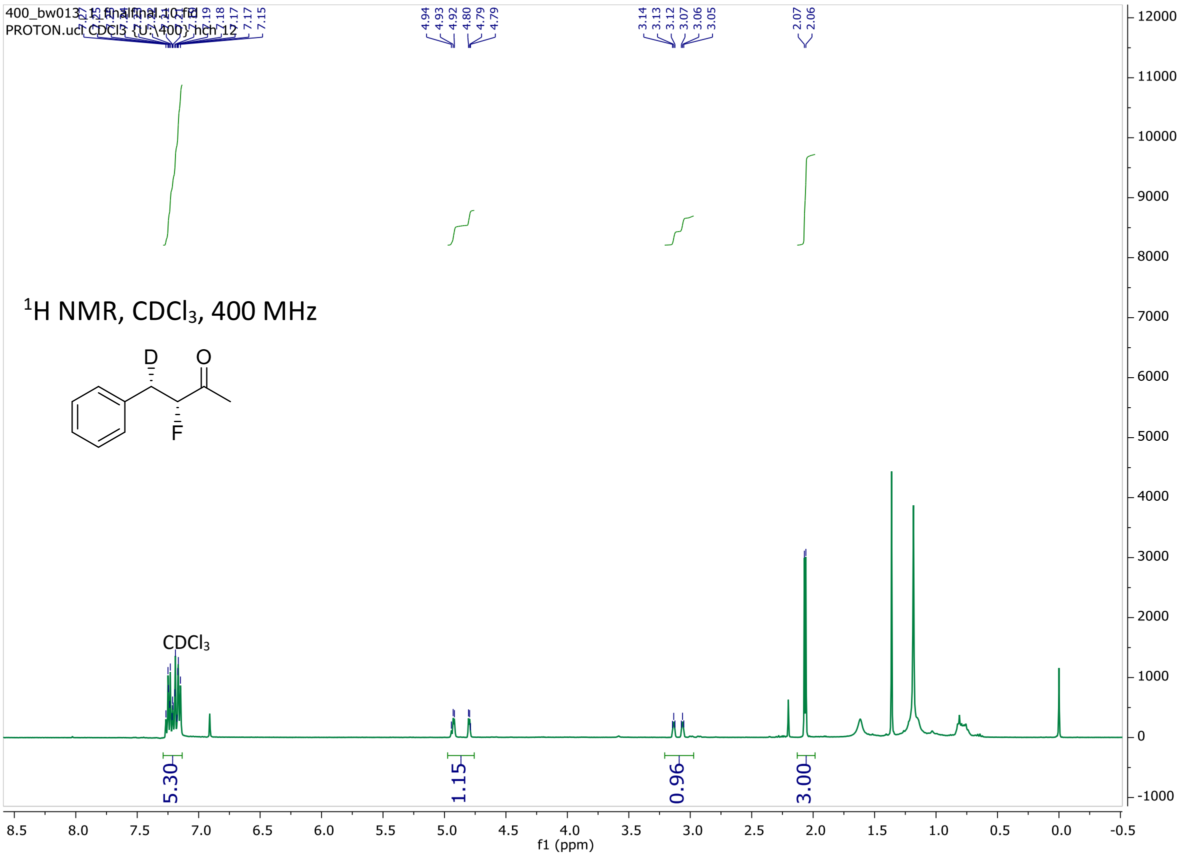

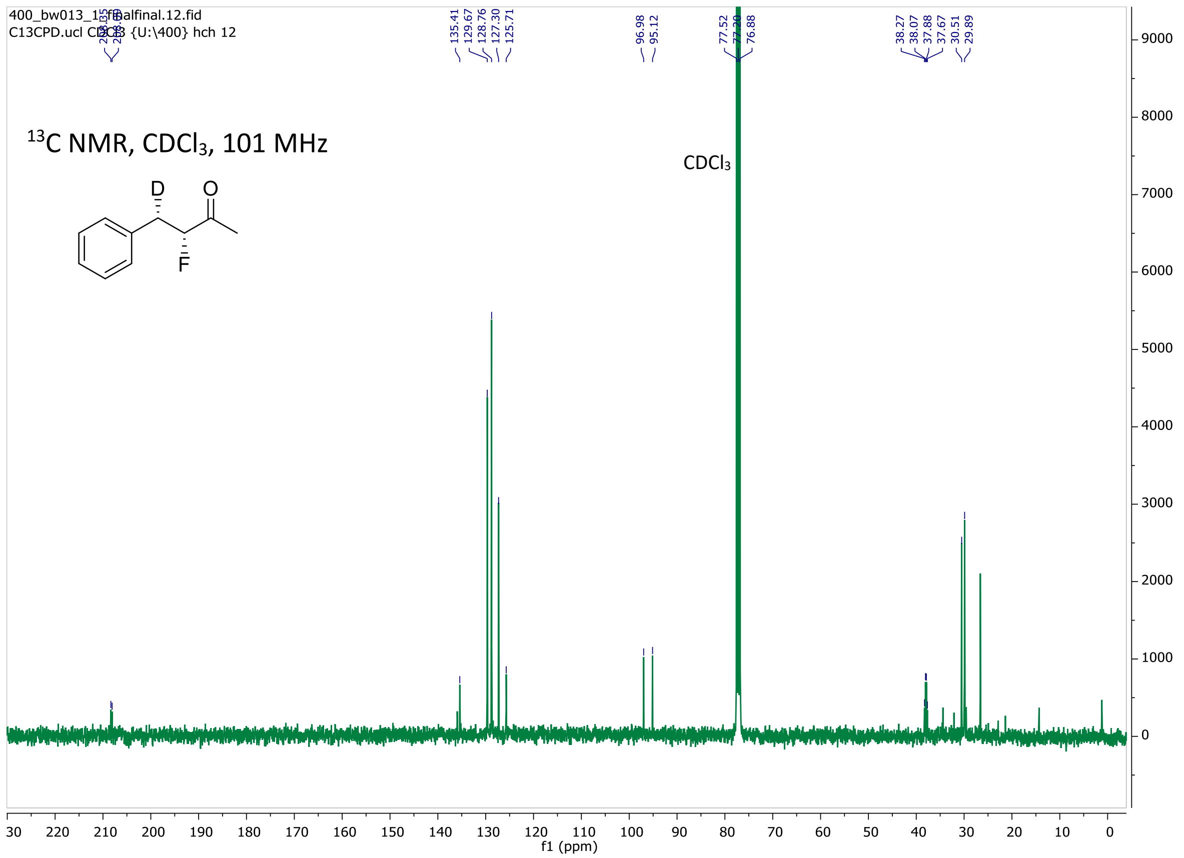
400_bw013_1_finalfinal.12.fid
C13CPD.ucl CDCl3 {U:\400} hch 12
13C NMR, CDCl3, 101 MHz
CDCl3
135.41
129.67
128.76
127.30
125.71
96.98
95.12
77.52
76.88
38.27
38.07
37.88
37.67
30.51
29.89
f1 (ppm)

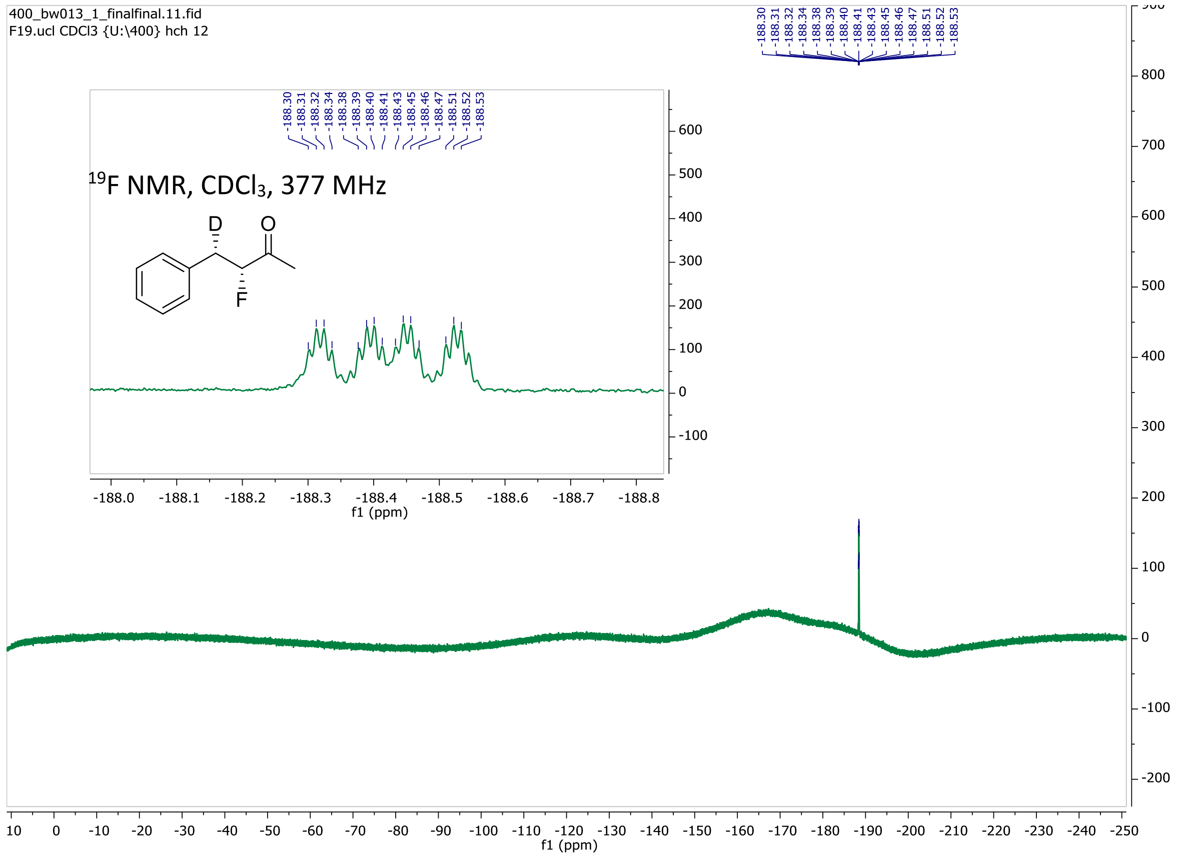
400_bw013_1_finalfinal.11.fid
F19.ucl CDCl3 {U:\400} hch 12
19F NMR, CDCl3, 377 MHz
-188.30
-188.31
-188.32
-188.34
-188.38
-188.39
-188.40
-188.41
-188.43
-188.45
-188.46
-188.47
-188.51
-188.52
-188.53
f1 (ppm)

# (3*S*,4*S*)-3-Fluoro-4-phenylbutan-2-one-4-d ((3*S*,4*S*)-7a)

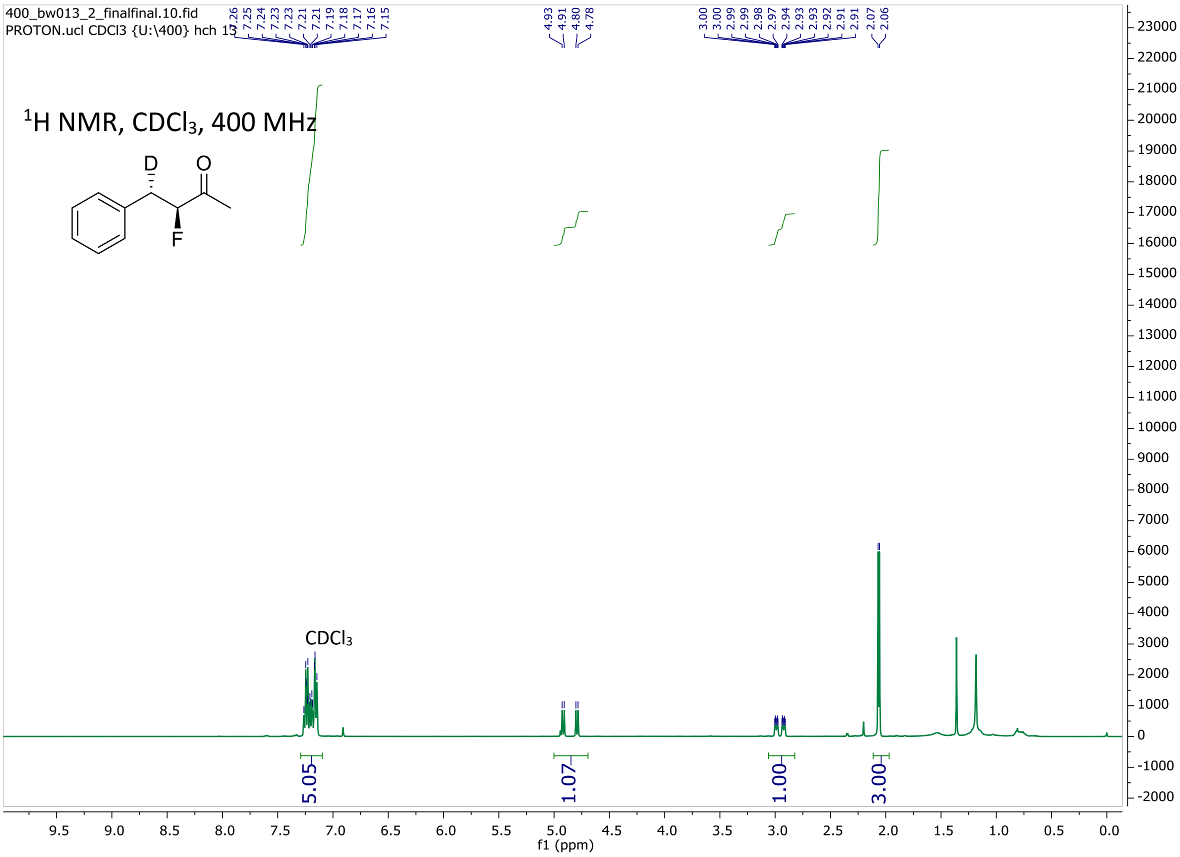

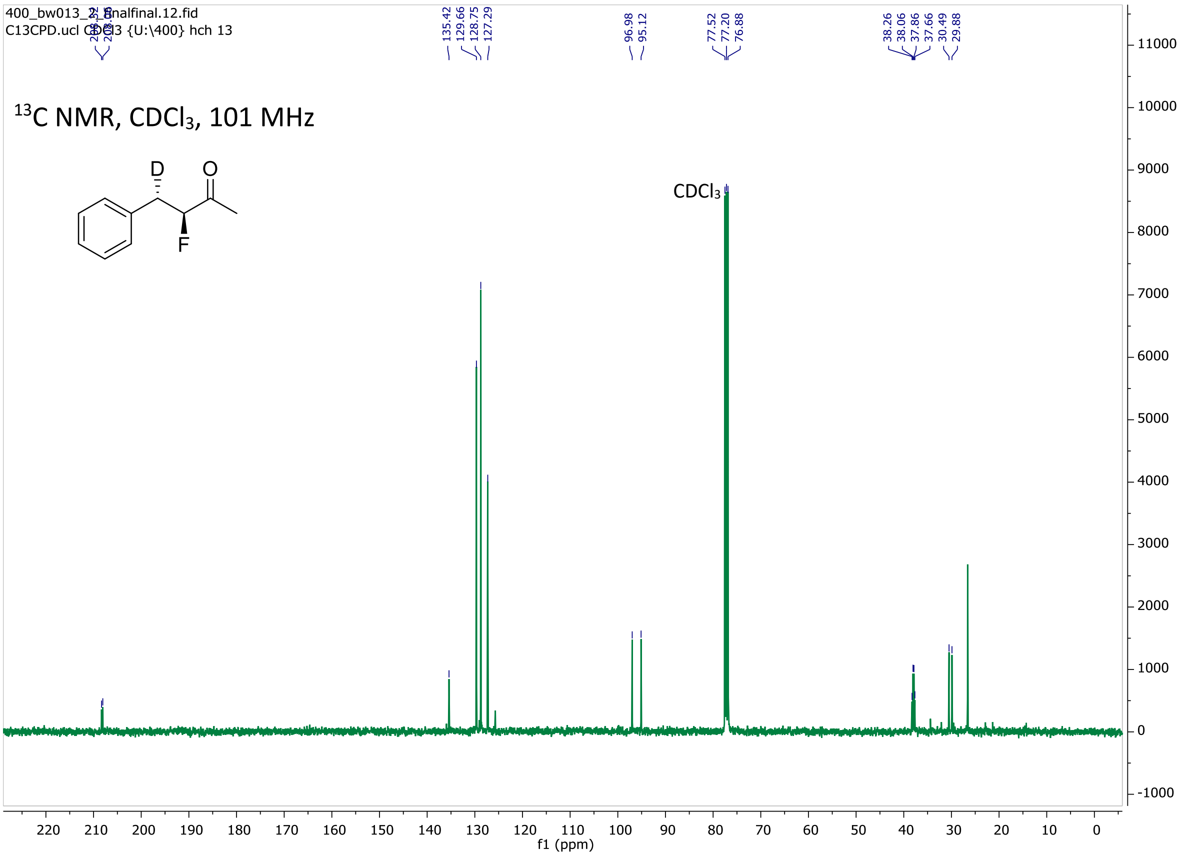
400_bw013_2_finalfinal.12.fid
C13CPD.ucl CDCl3 {U:\400} hch 13
135.42
129.66
128.75
127.29
96.98
95.12
77.52
77.20
76.88
38.26
38.06
37.86
37.66
30.49
29.88
$^{13}$C NMR, $CDCl_3$, 101 MHz
D
O
F
$CDCl_3$
f1 (ppm)

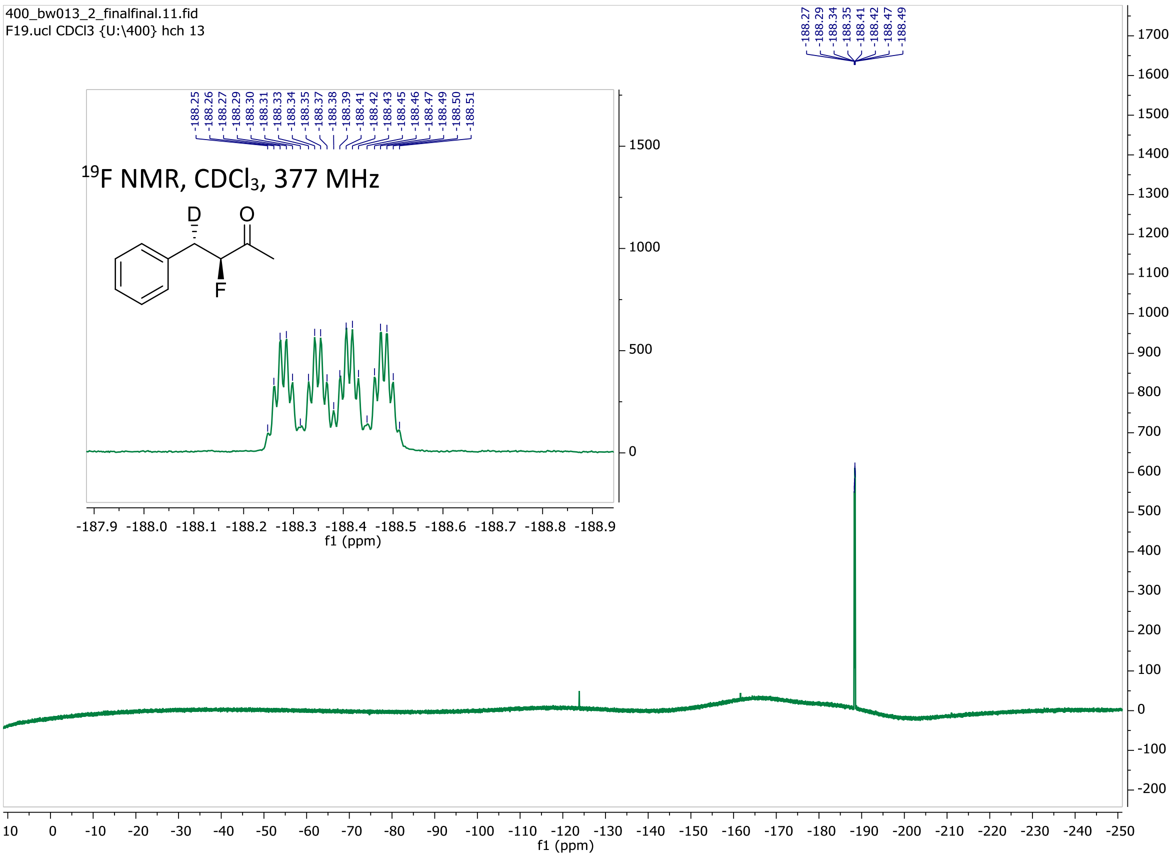
400_bw013_2_finalfinal.11.fid
F19.ucl CDCl3 {U:\400} hch 13
-188.27
-188.29
-188.34
-188.35
-188.41
-188.42
-188.47
-188.49
-188.25
-188.26
-188.27
-188.29
-188.30
-188.31
-188.33
-188.34
-188.35
-188.37
-188.38
-188.39
-188.41
-188.42
-188.43
-188.45
-188.46
-188.47
-188.49
-188.50
-188.51
$^{19}$F NMR, $CDCl_3$, 377 MHz
D
O
F
f1 (ppm)
f1 (ppm)

## Methyl (*Z*)-2-fluoro-3-phenylacrylate ((*Z*)-6a)

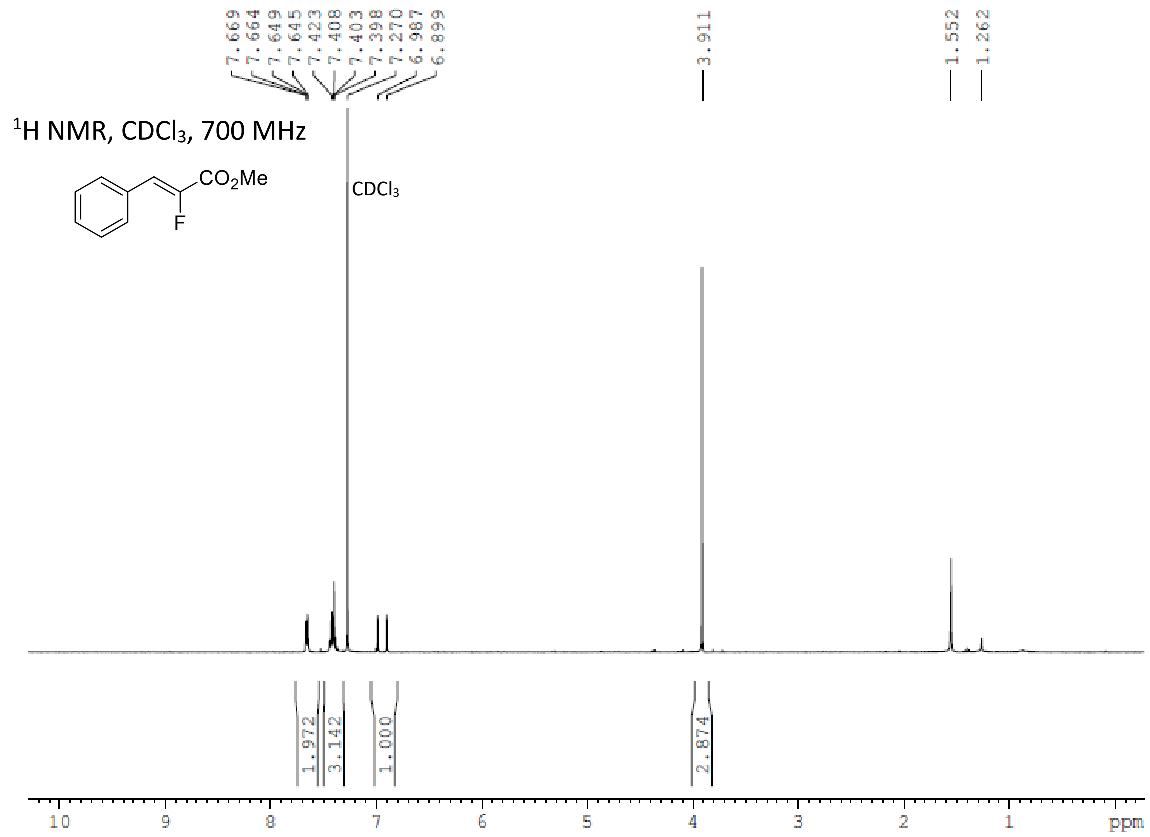


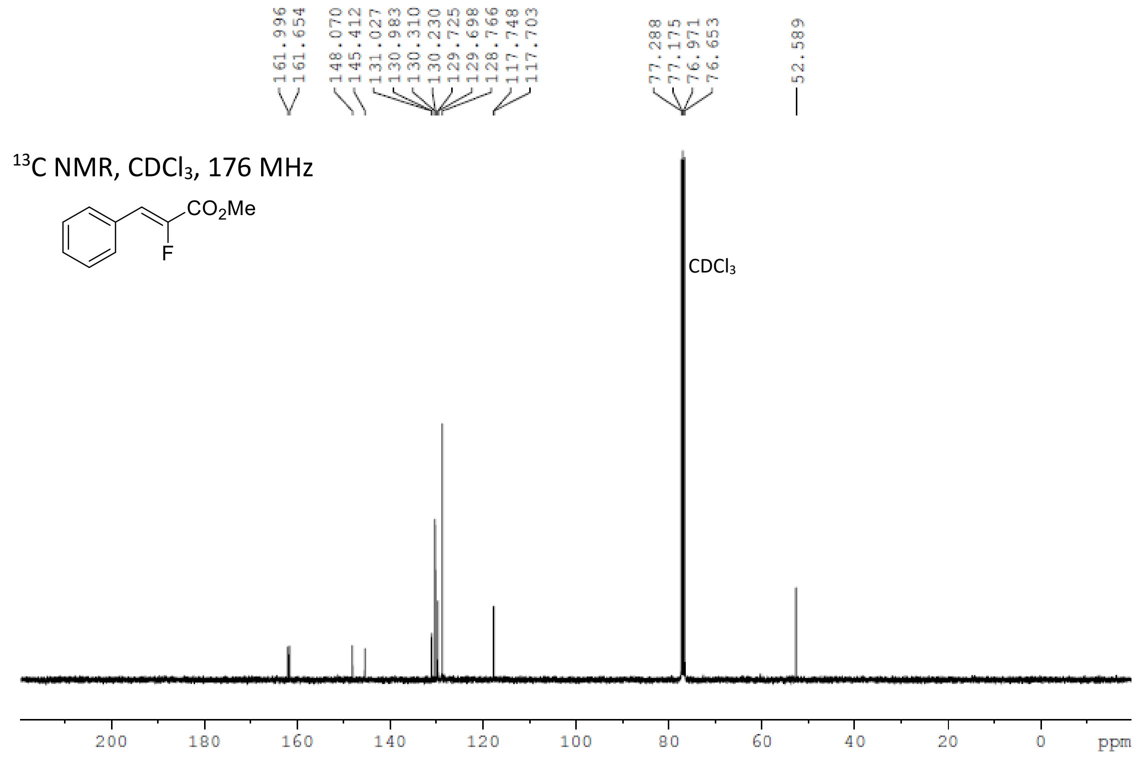

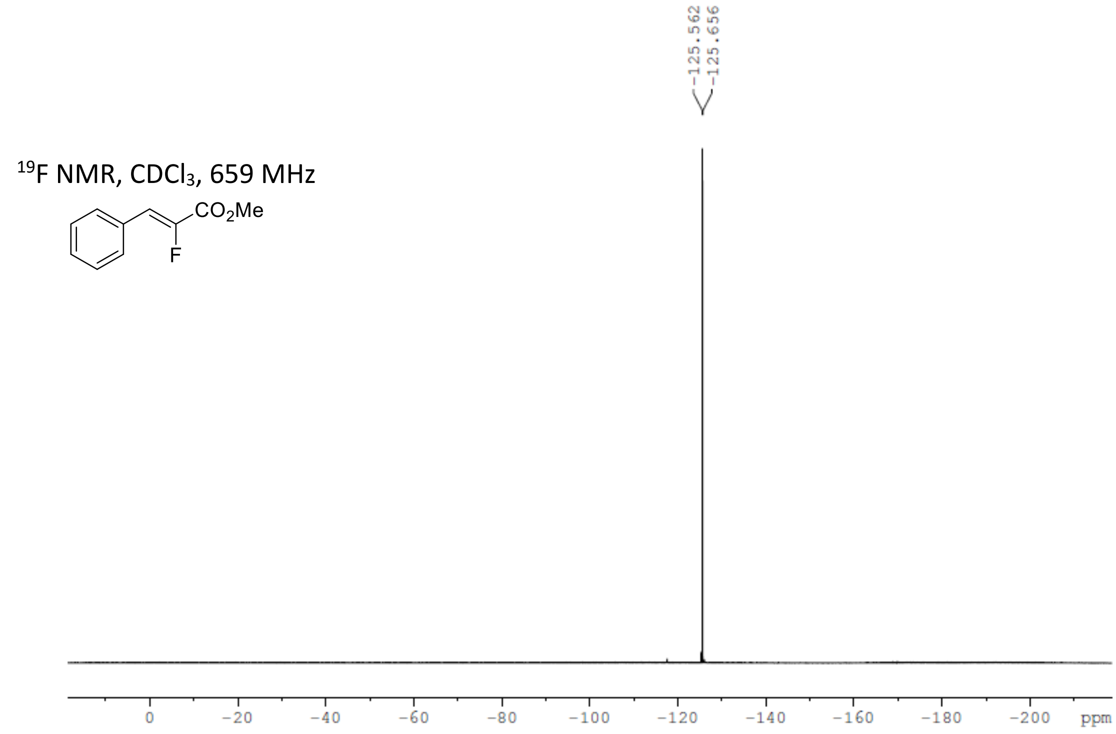


## Methyl (*E*)-2-fluoro-3-phenylacrylate ((*E*)-6a) (8:1 mix with (*Z*)-6a)

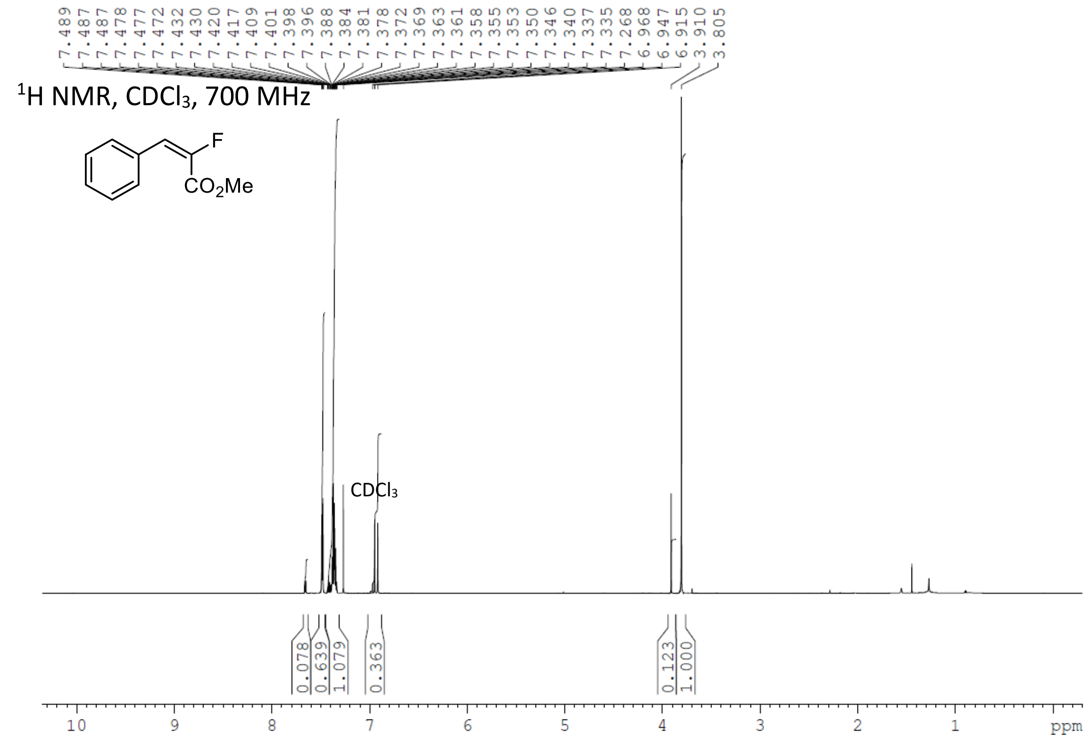

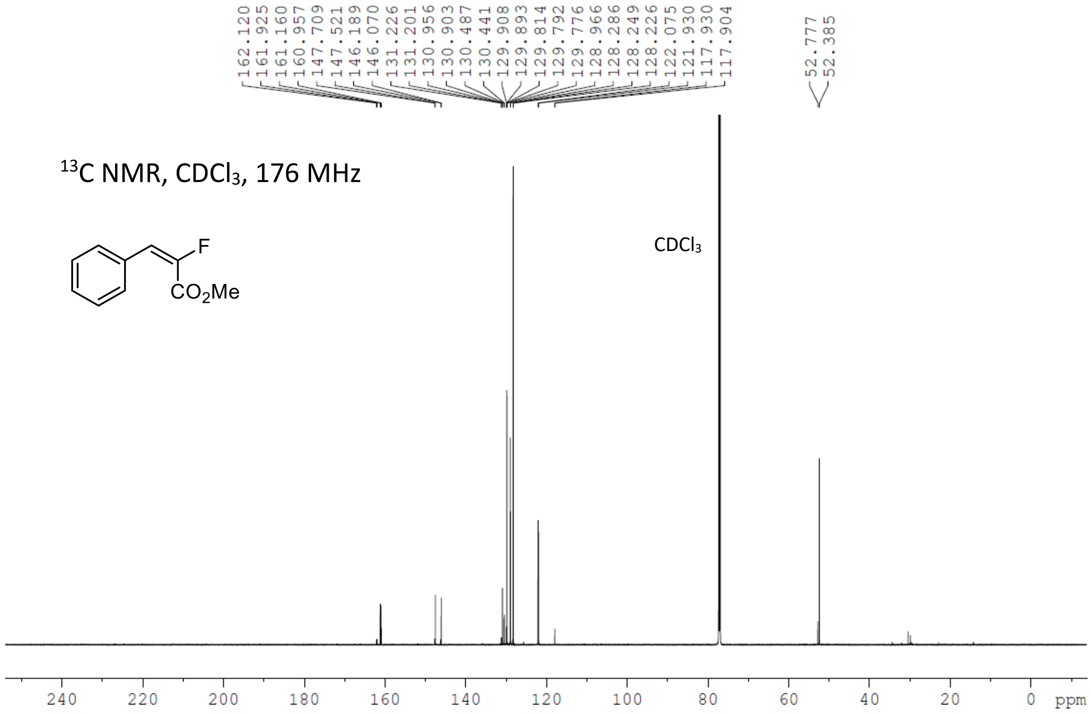
162.120
161.925
161.160
160.957
147.709
147.521
146.189
146.070
131.226
131.201
130.956
130.903
130.487
130.441
129.908
129.893
129.814
129.792
129.776
128.966
128.286
128.249
128.226
122.075
121.930
117.930
117.904
52.777
52.385
13C NMR, CDCl3, 176 MHz
F
CO2Me
CDCl3
240
220
200
180
160
140
120
100
80
60
40
20
0
ppm

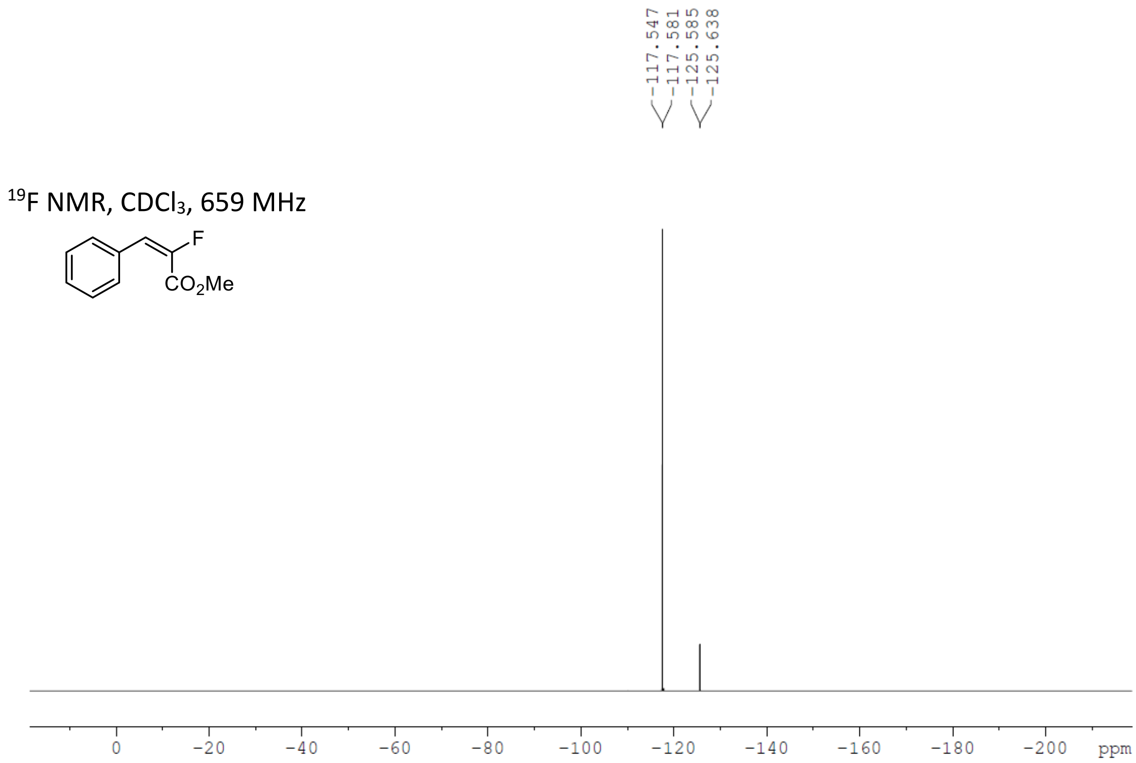
-117.547
-117.581
-125.585
-125.638
19F NMR, CDCl3, 659 MHz
F
CO2Me
0
-20
-40
-60
-80
-100
-120
-140
-160
-180
-200
ppm

Methyl (*Z*)-2-fluoro-3-(4-trifluoromethylphenyl)acrylate ((*Z*)-6b)

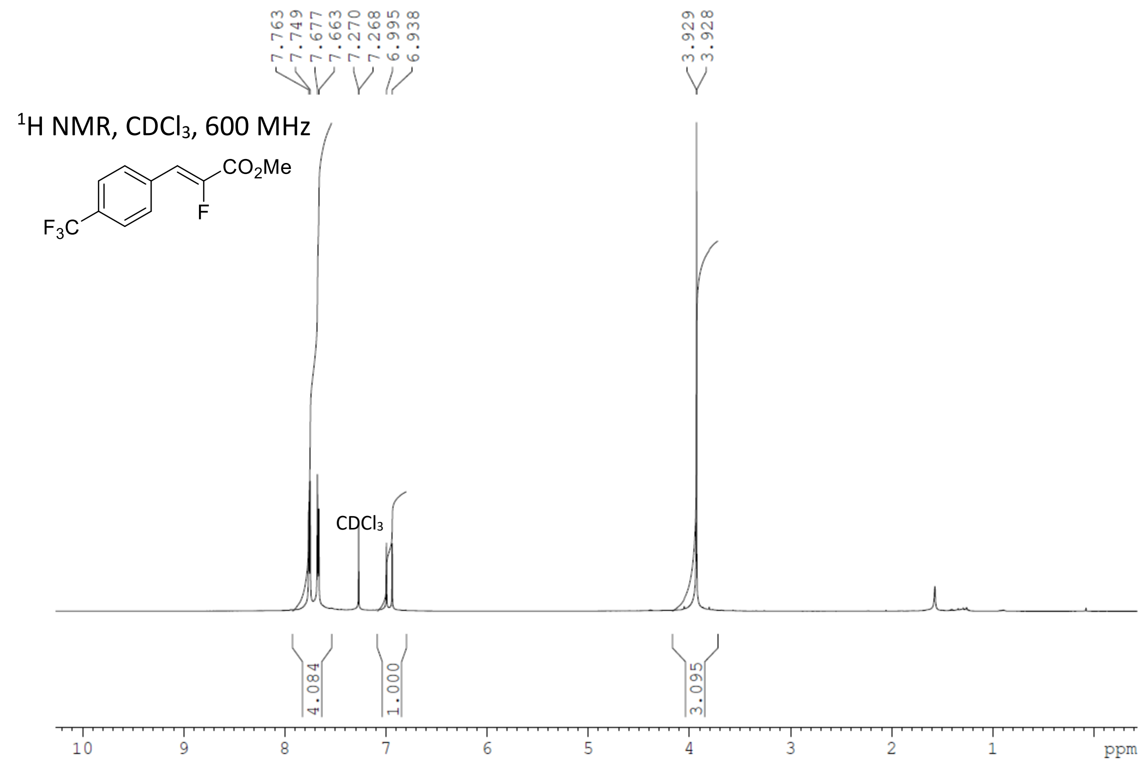


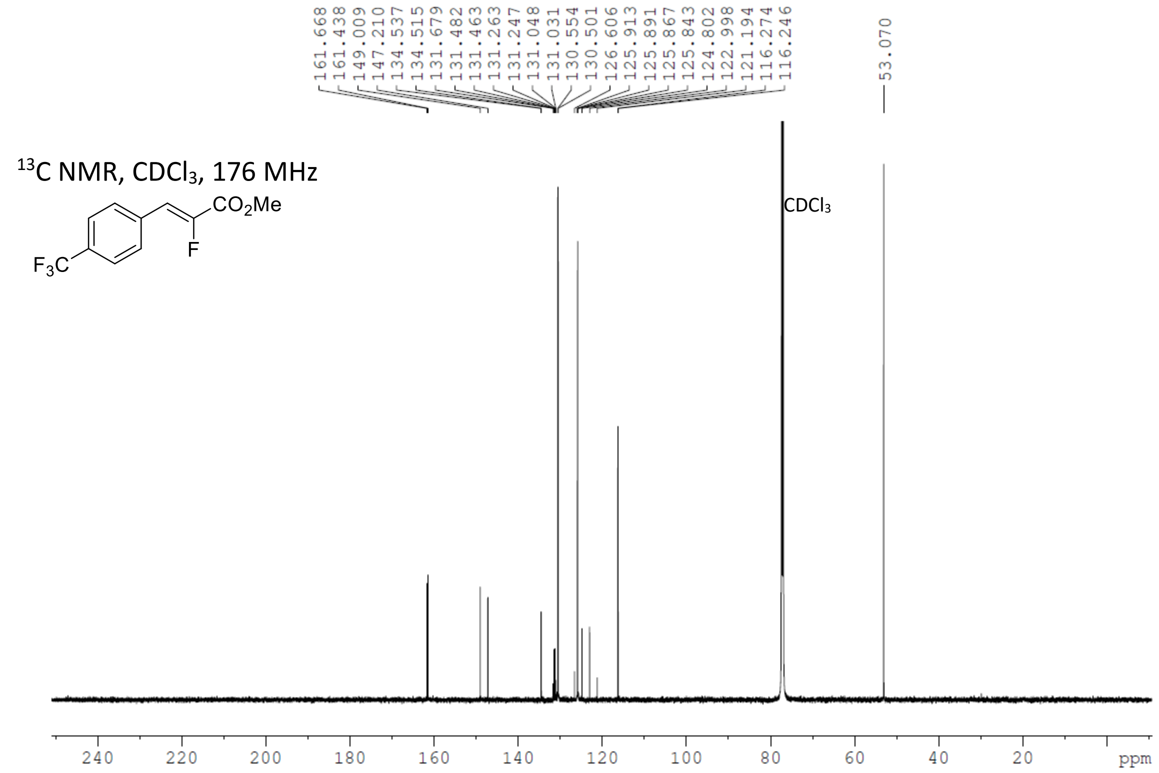

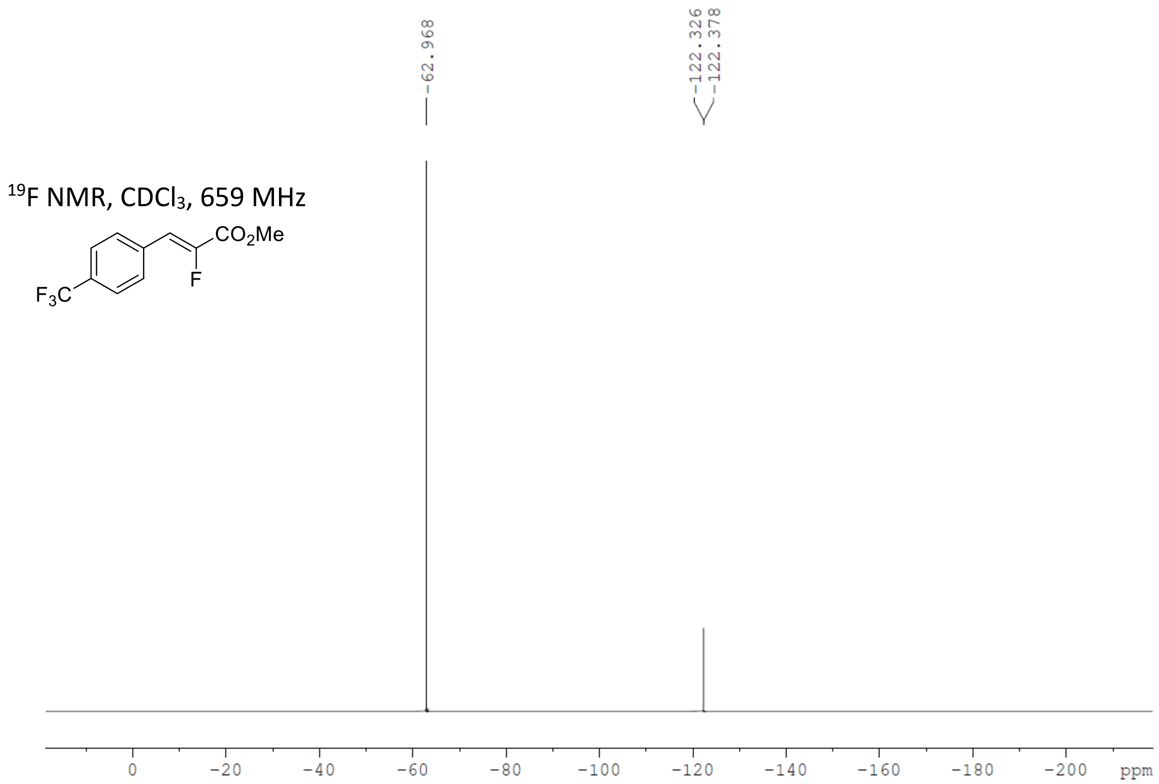


## Methyl (*E*)-2-fluoro-3-(4-trifluoromethylphenyl)acrylate ((*E*)-6b)

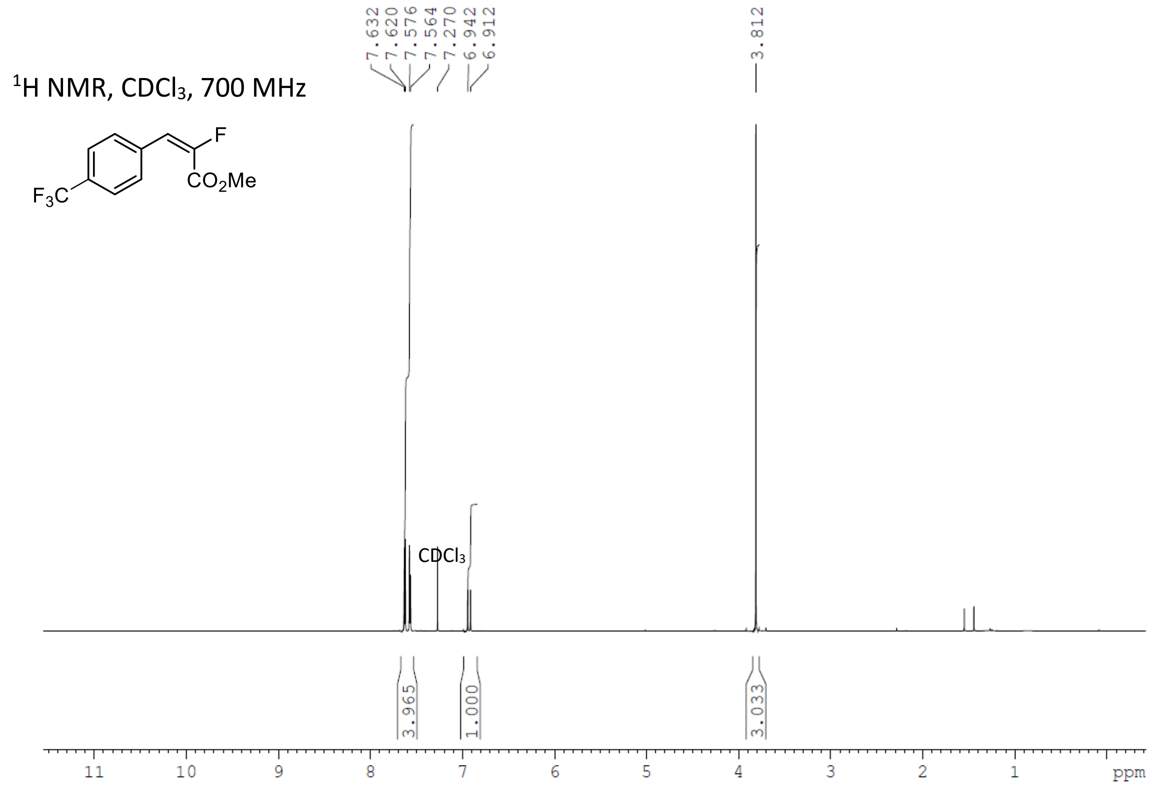

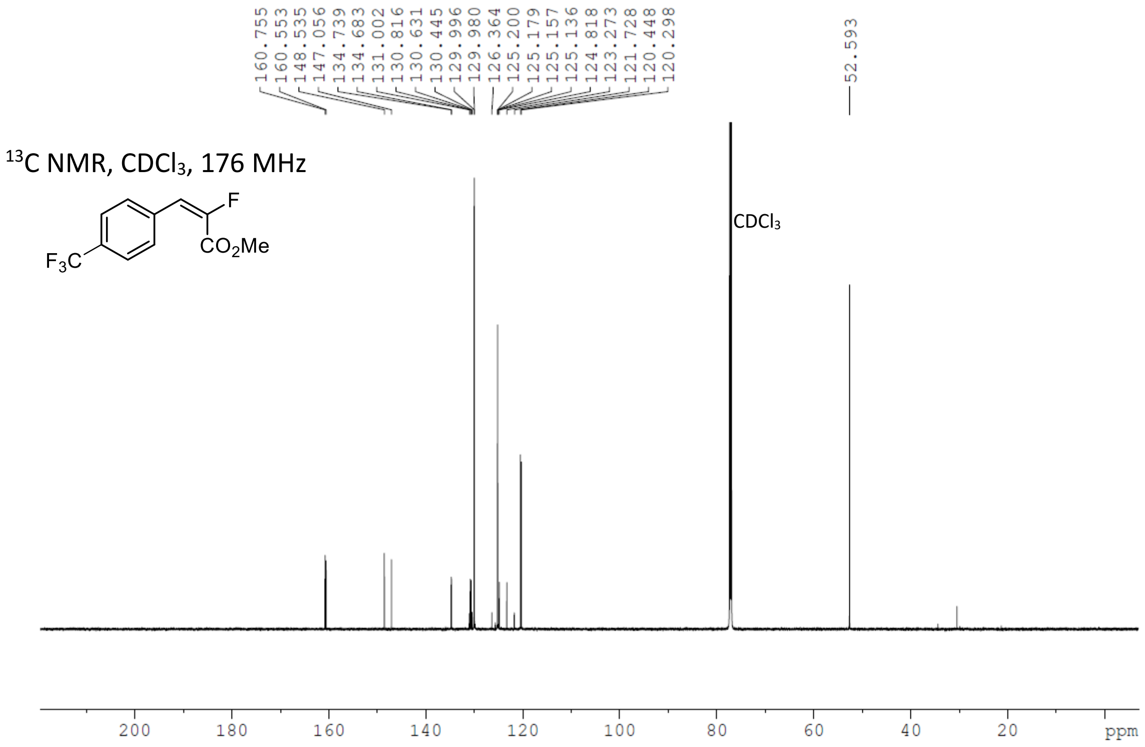
160.755
160.553
148.535
147.056
134.739
134.683
131.002
130.816
130.631
130.445
129.996
129.980
126.364
125.200
125.179
125.157
125.136
124.818
123.273
121.728
120.448
120.298
52.593
13C NMR, CDCl3, 176 MHz
F
F3C
CO2Me
CDCl3
200
180
160
140
120
100
80
60
40
20
ppm

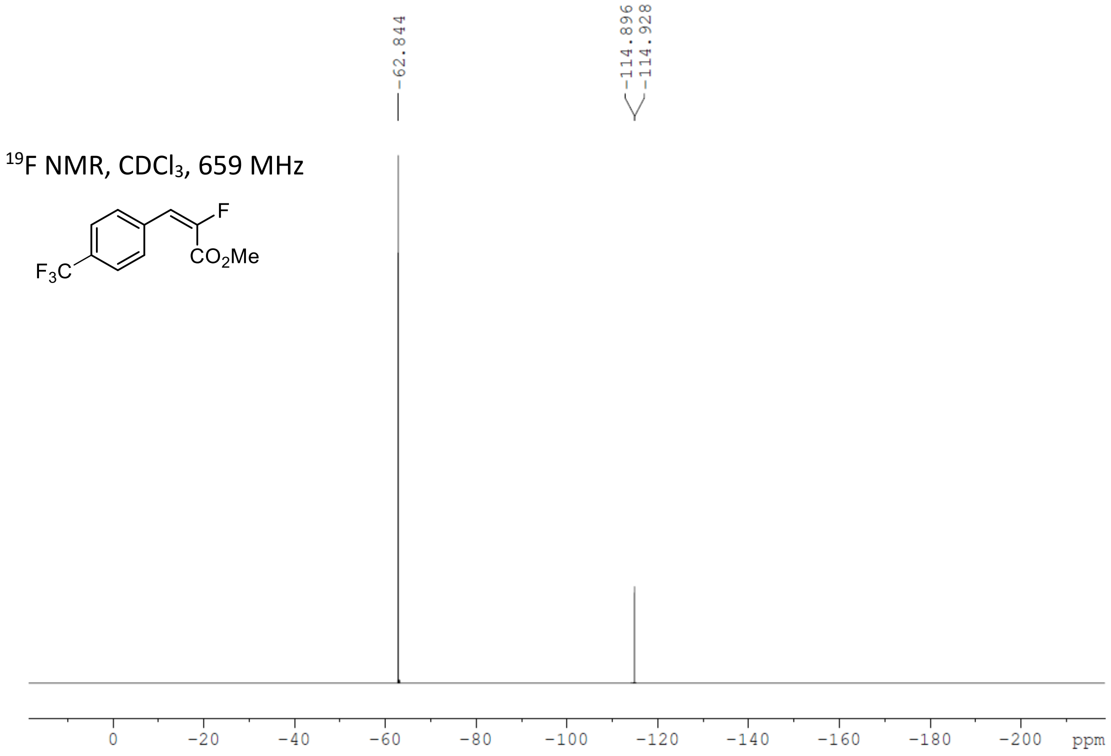
-62.844
-114.896
-114.928
19F NMR, CDCl3, 659 MHz
F
F3C
CO2Me
0
-20
-40
-60
-80
-100
-120
-140
-160
-180
-200
ppm

## Methyl (*Z*)-2-fluoro-3-(4-methoxyphenyl)acrylate ((*Z*)-6c)

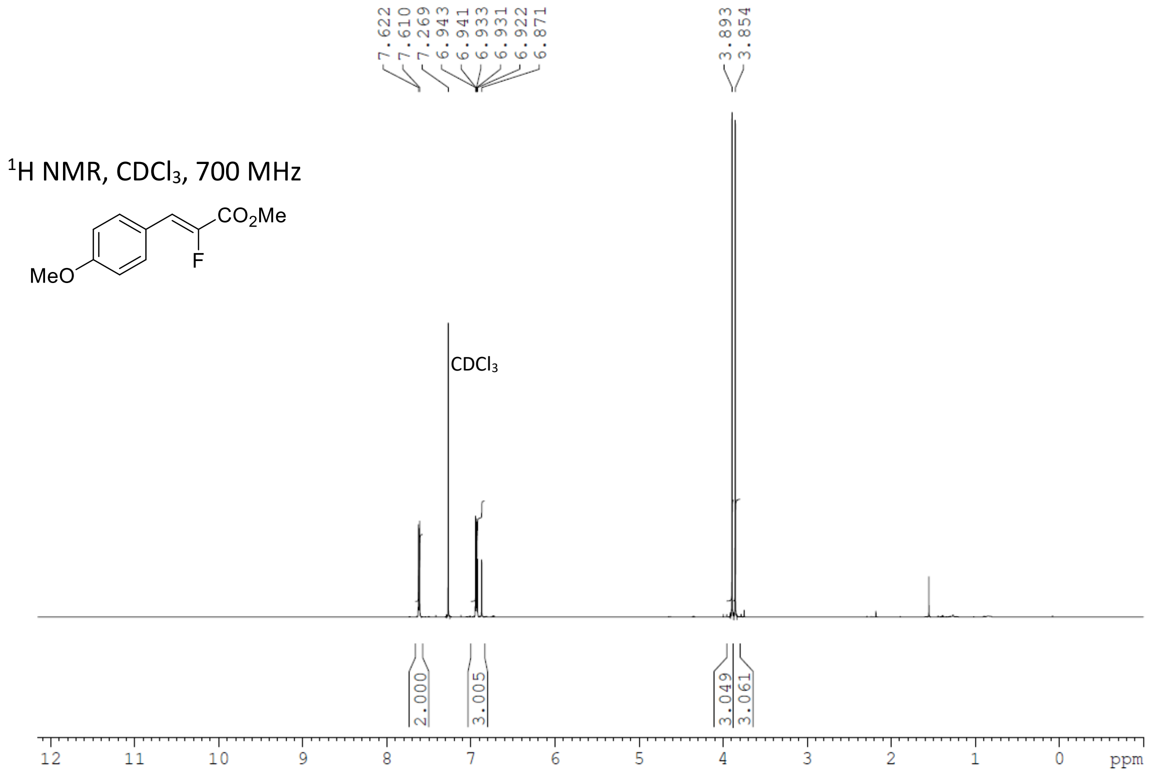


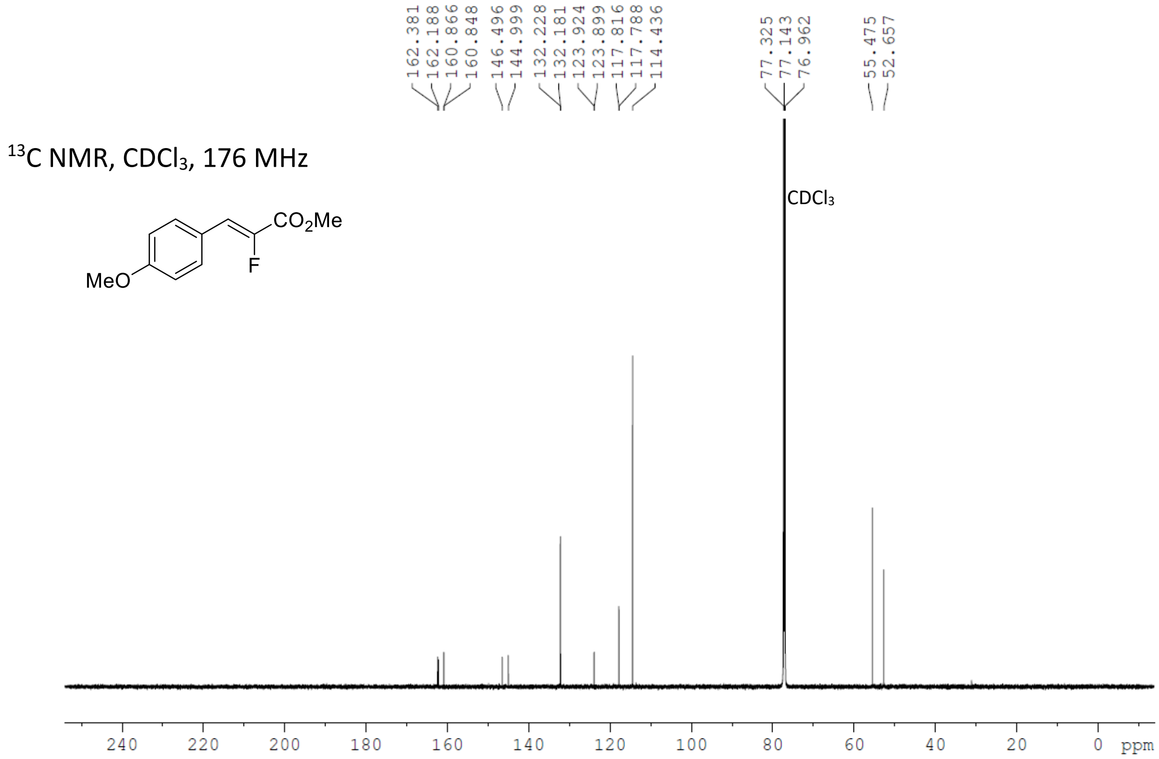

$^{19}$F NMR, $CDCl_3$, 659 MHz

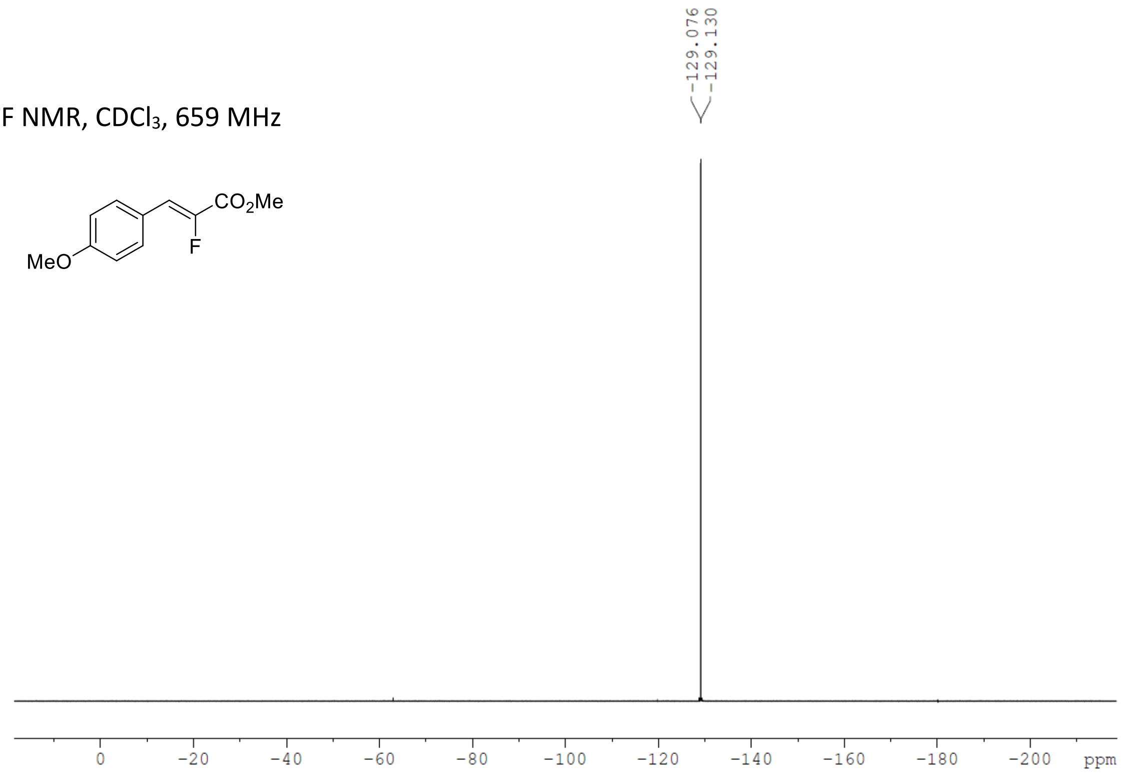

# Methyl (*E*)-2-fluoro-3-(4-methoxyphenyl)acrylate ((*E*)-6c) (5:1 mix with (*Z*)-3c)

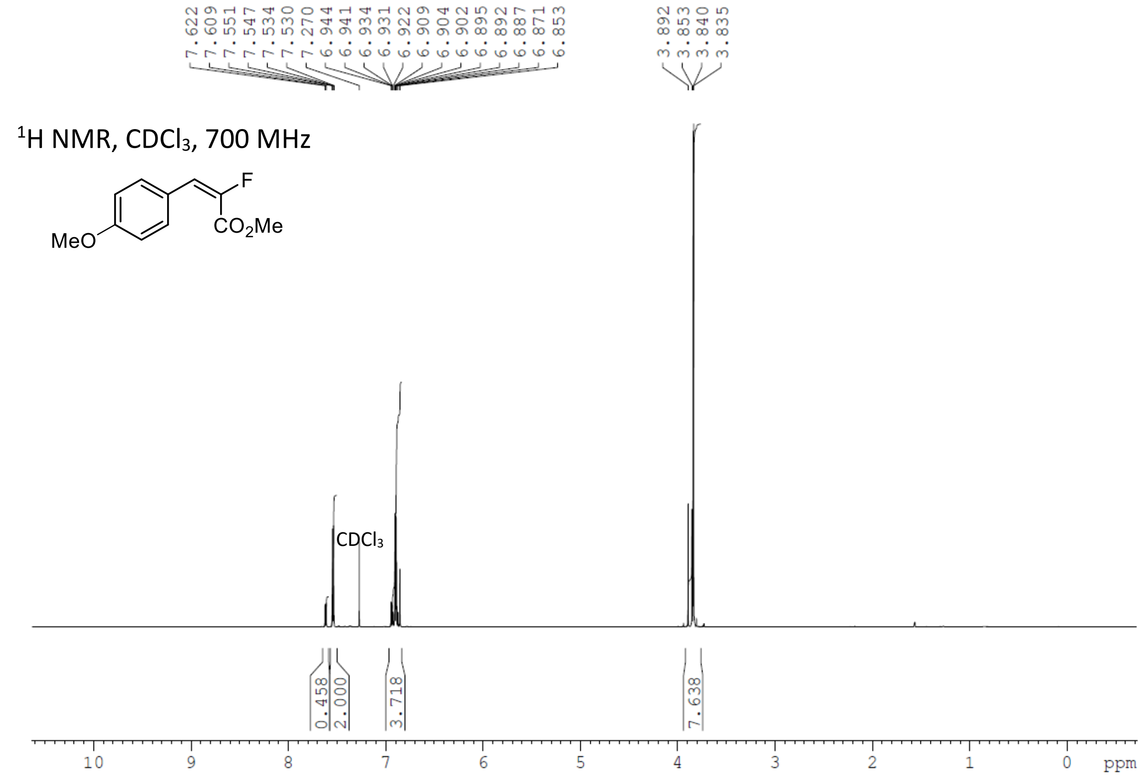


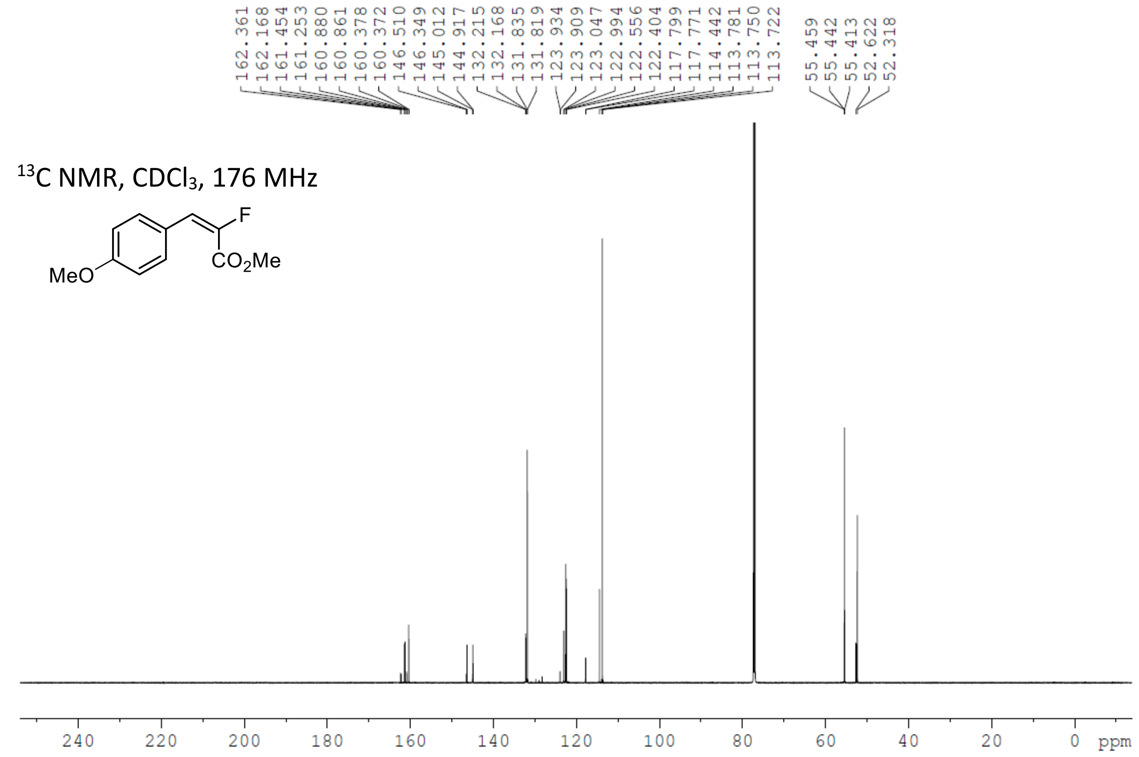

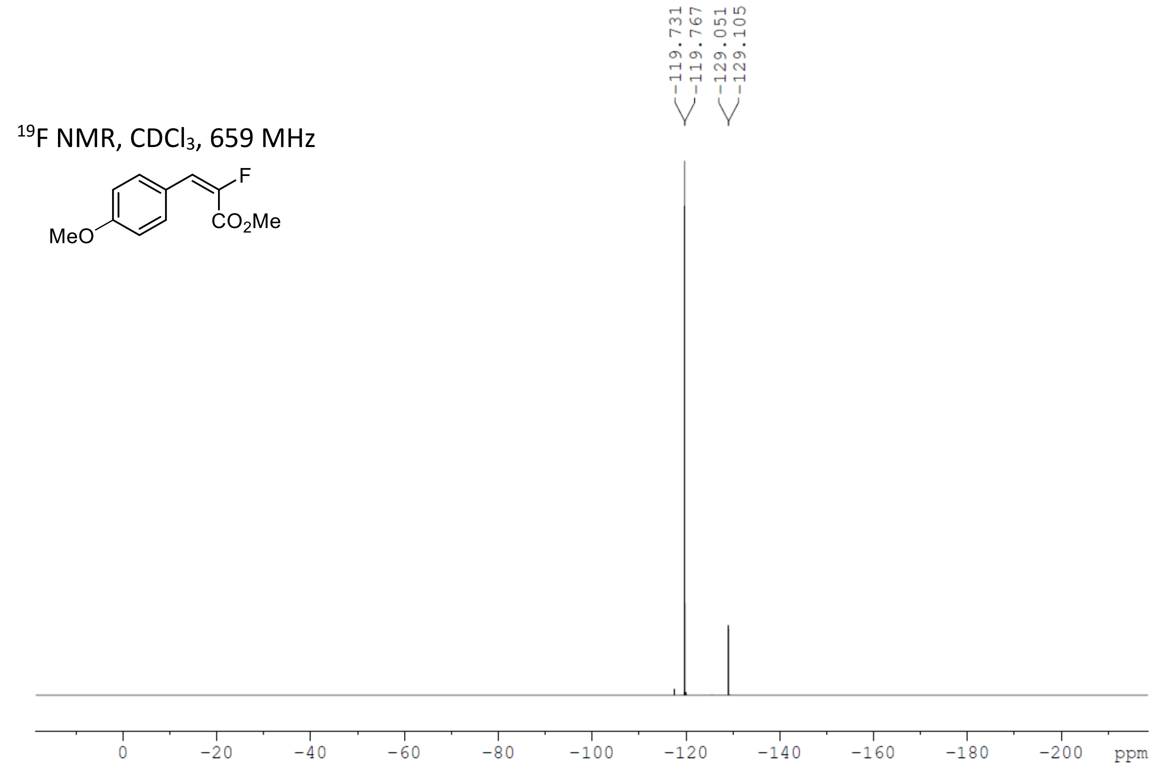


# Ethyl (*E*)-2-fluoro-3-(4-trifluoromethylphenyl)acrylate ((*E*)-6d)

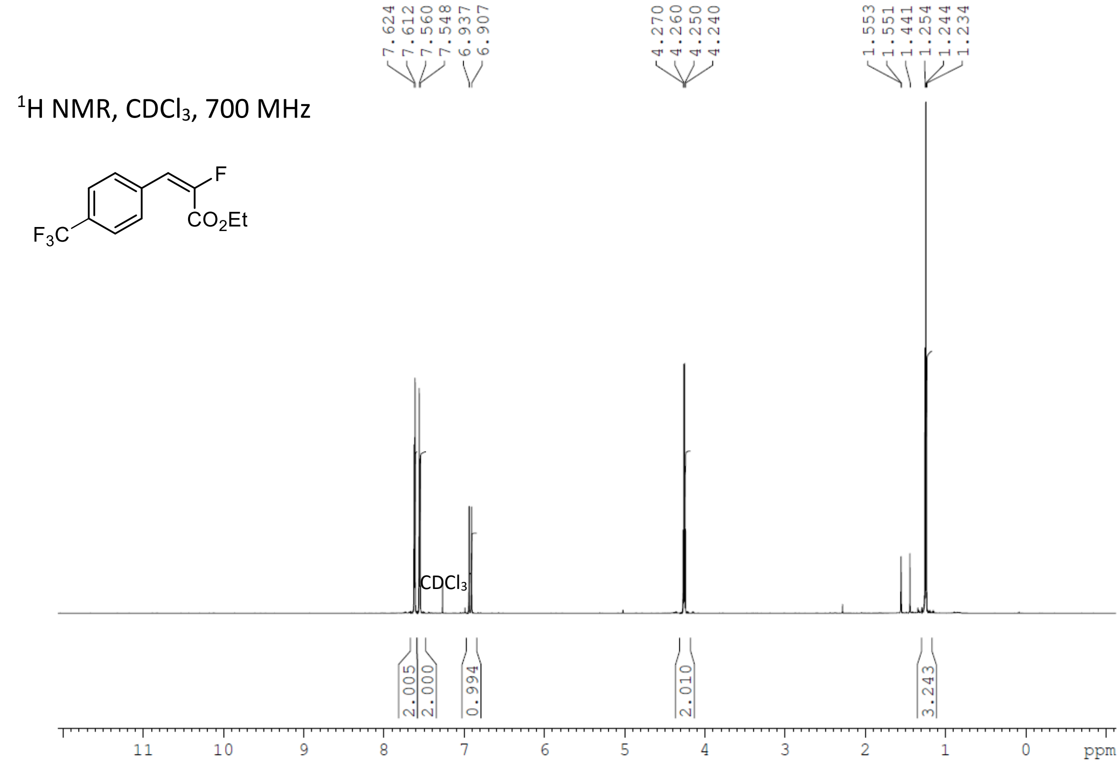

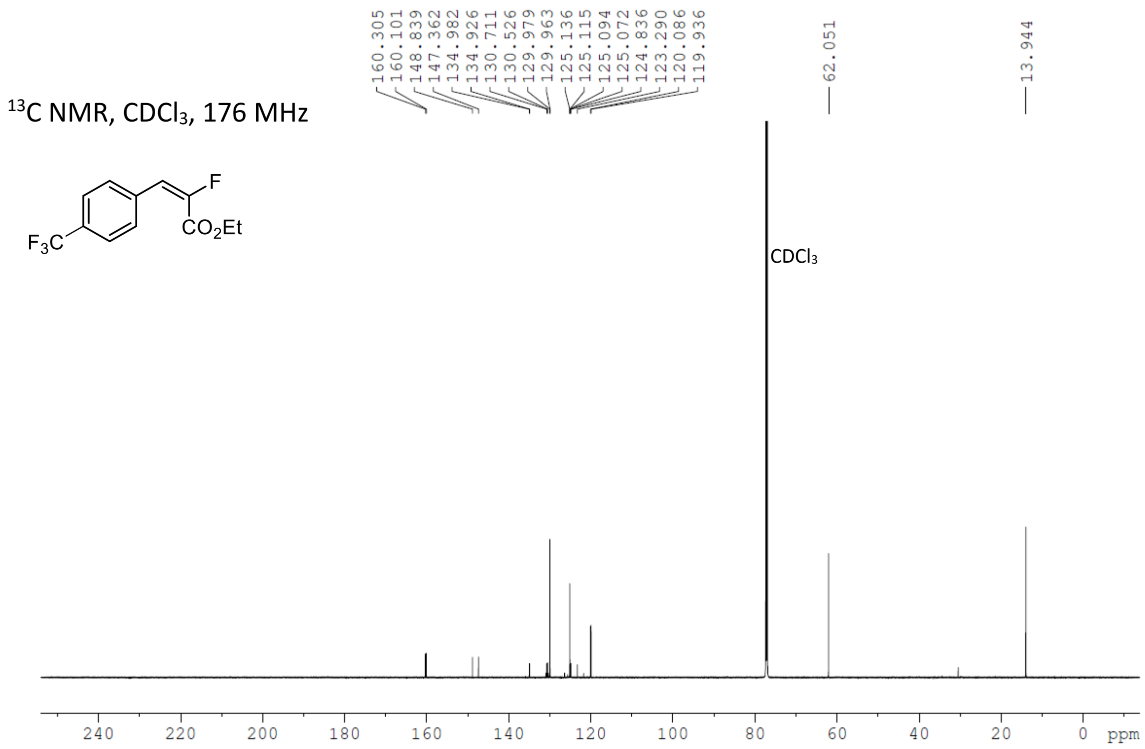
13C NMR, CDCl3, 176 MHz
F
CO2Et
F3C
160.305
160.101
148.839
147.362
134.982
134.926
130.711
130.526
129.979
129.963
125.136
125.115
125.094
125.072
124.836
123.290
120.086
119.936
62.051
13.944
CDCl3
240
220
200
180
160
140
120
100
80
60
40
20
0
ppm

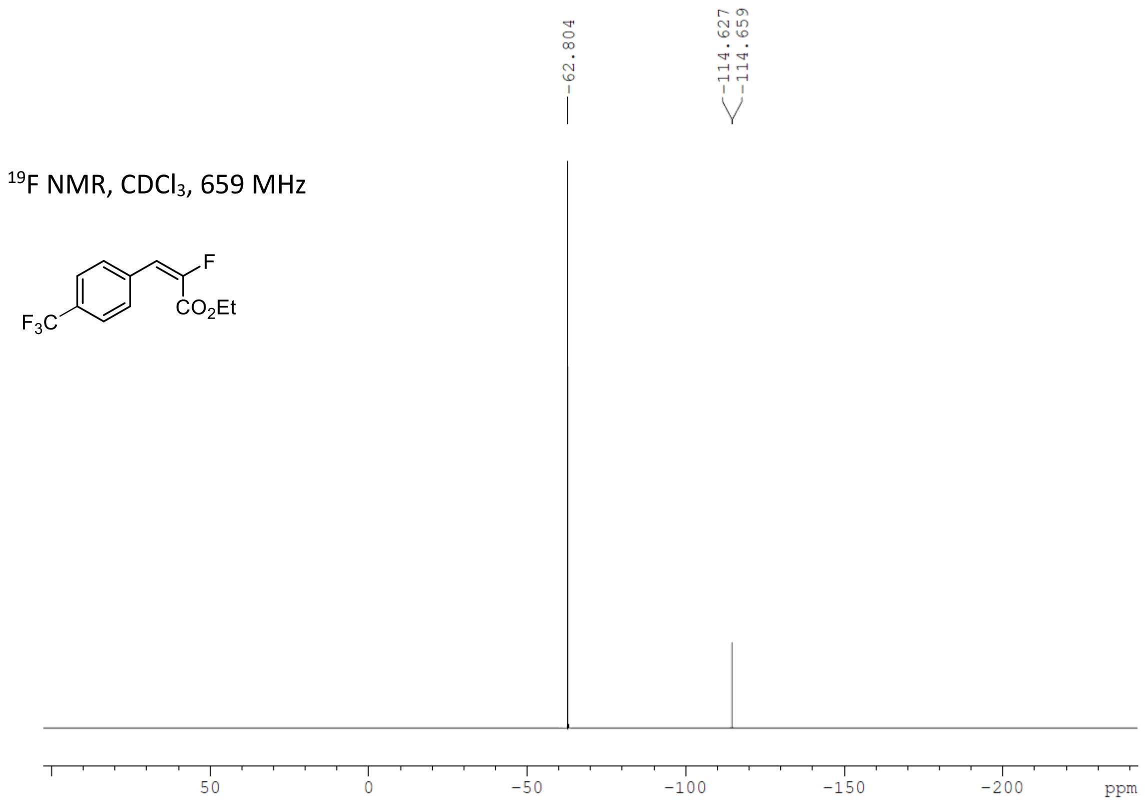
19F NMR, CDCl3, 659 MHz
F
CO2Et
F3C
-62.804
-114.627
-114.659
50
0
-50
-100
-150
-200
ppm

## Methyl (*E*)-2-fluorohept-2-enoate ((*E*)-6e) (13:1 mix with (*Z*)-6e)

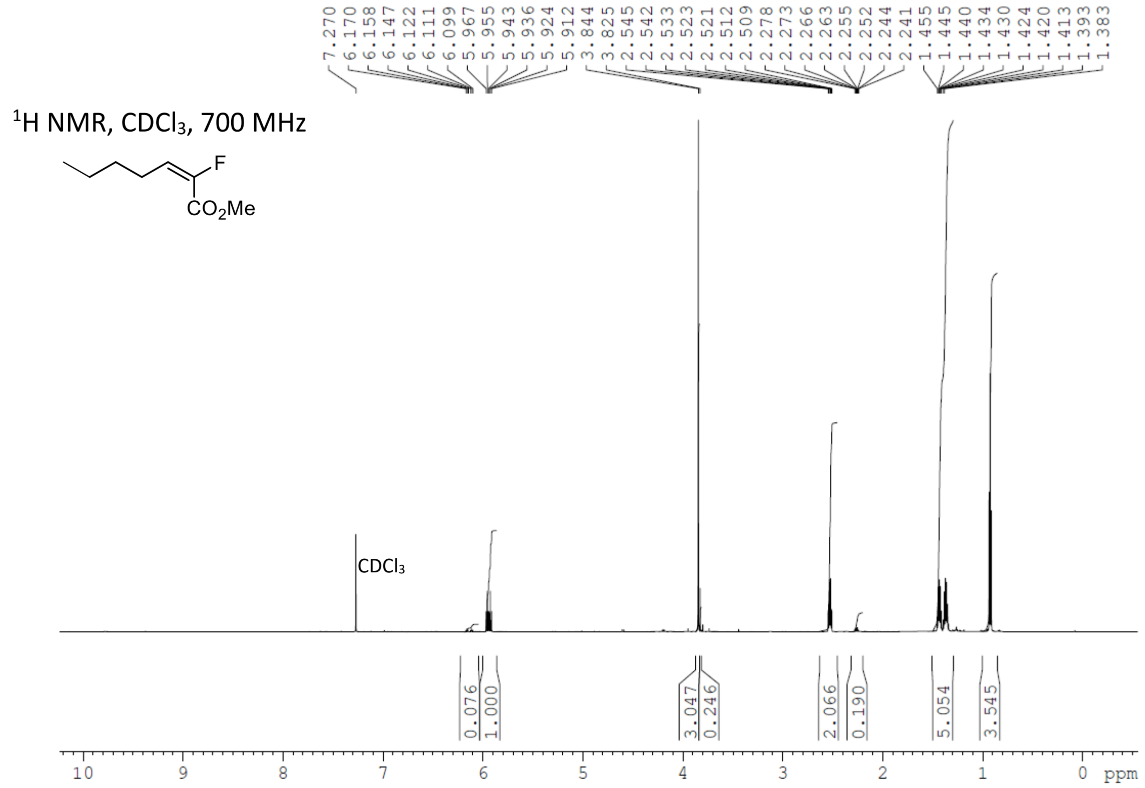


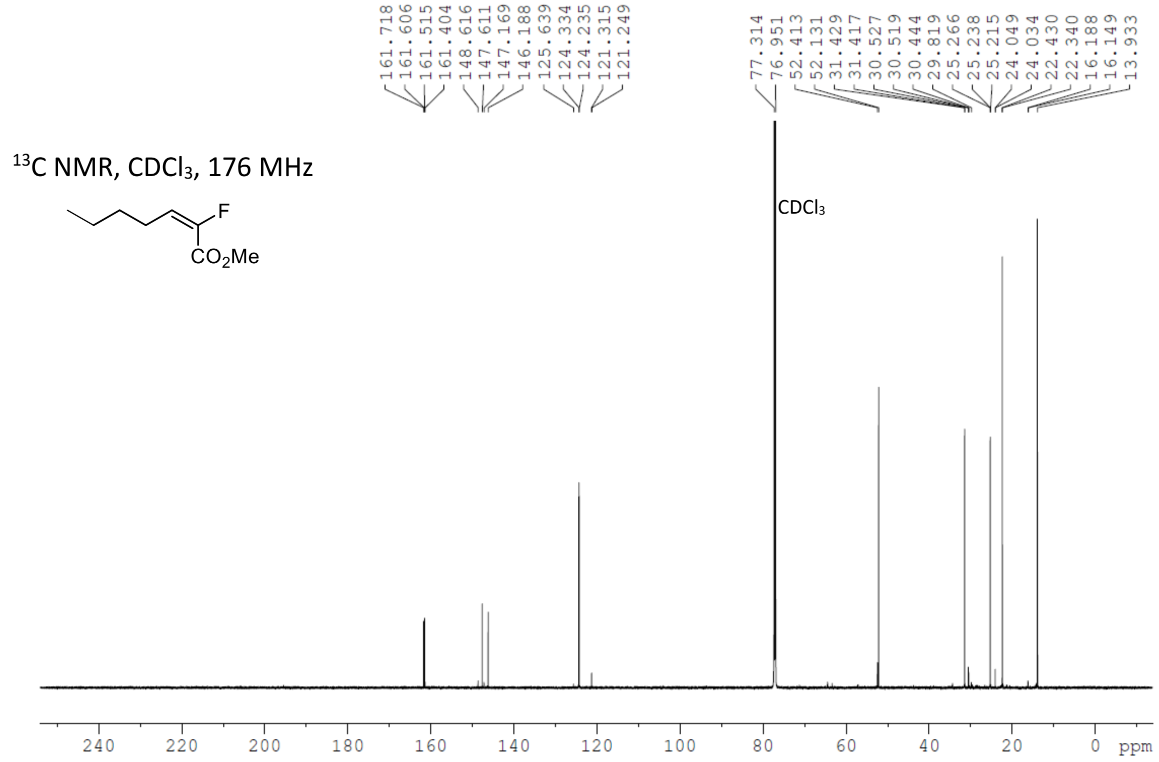

<sup></sup>

$^{19}$F NMR, $CDCl_3$, 659 MHz

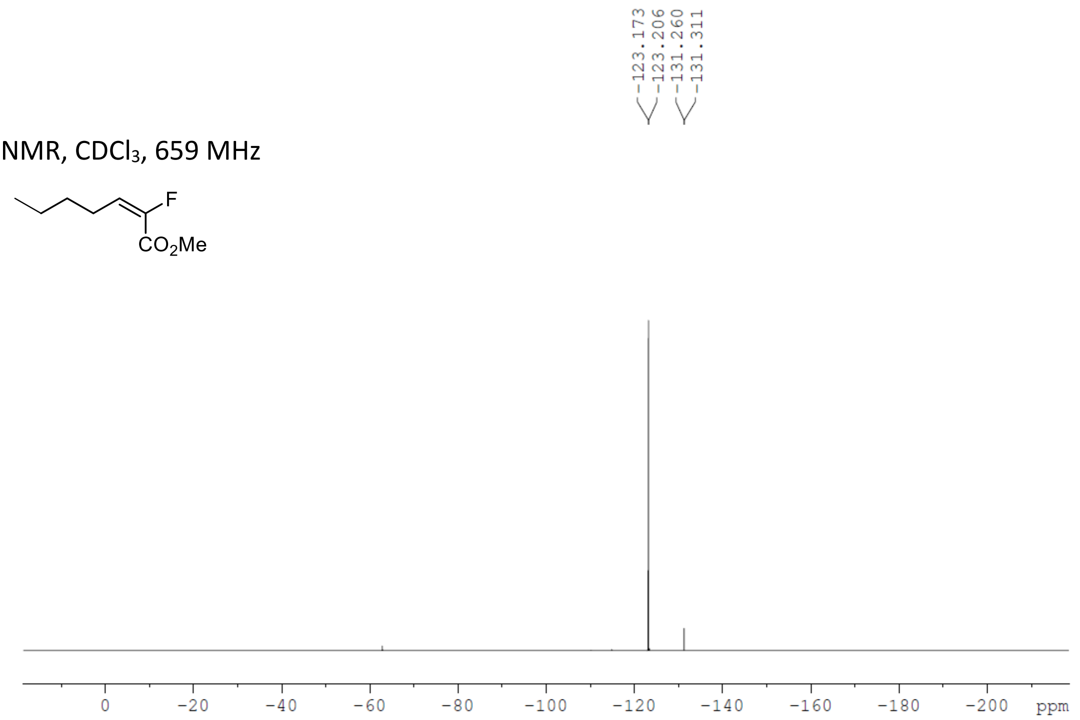


## Methyl 2-fluoro-3-phenylpropanoate (12a)

$^{1}$H NMR, $CDCl_3$, 700 MHz

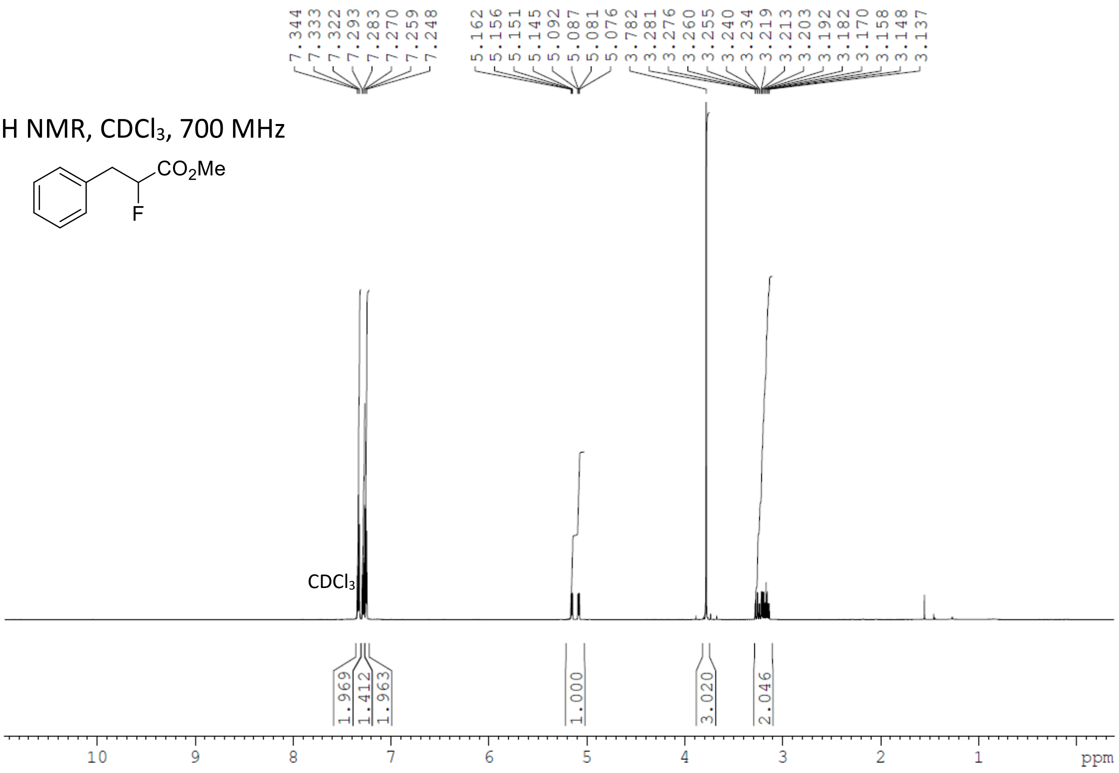

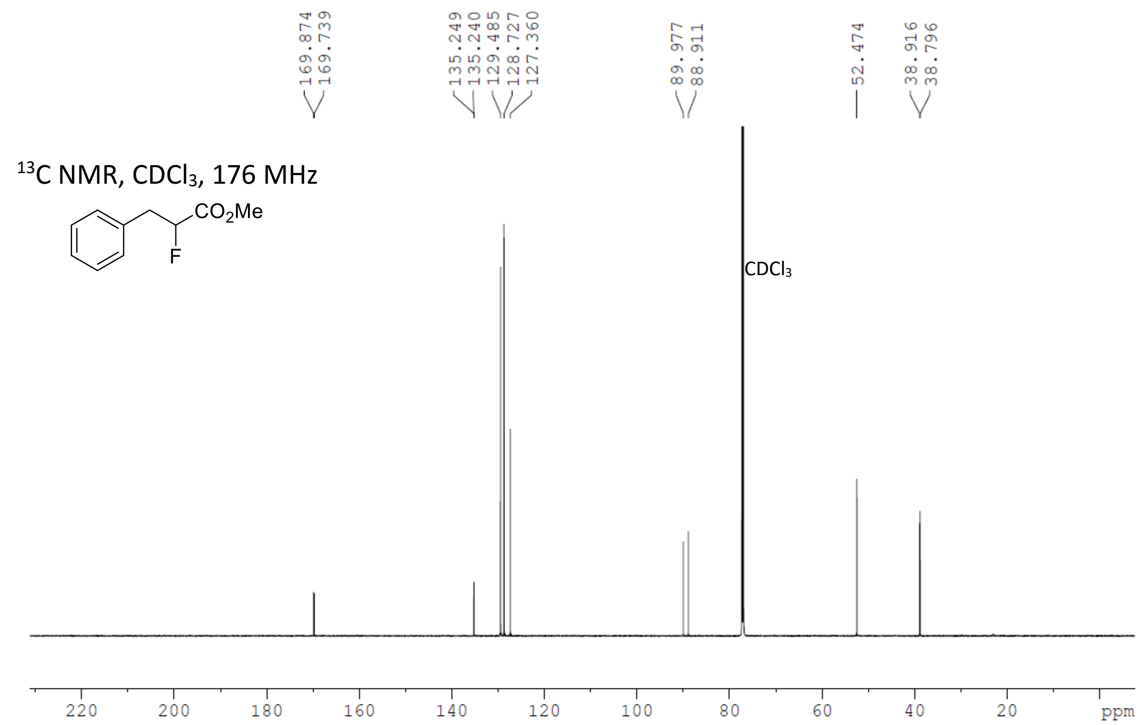

$^{13}C$ NMR, $CDCl_3$, 176 MHz
$CO_2Me$
F
169.874
169.739
135.249
135.240
129.485
128.727
127.360
89.977
88.911
52.474
38.916
38.796
$CDCl_3$
220 200 180 160 140 120 100 80 60 40 20 ppm


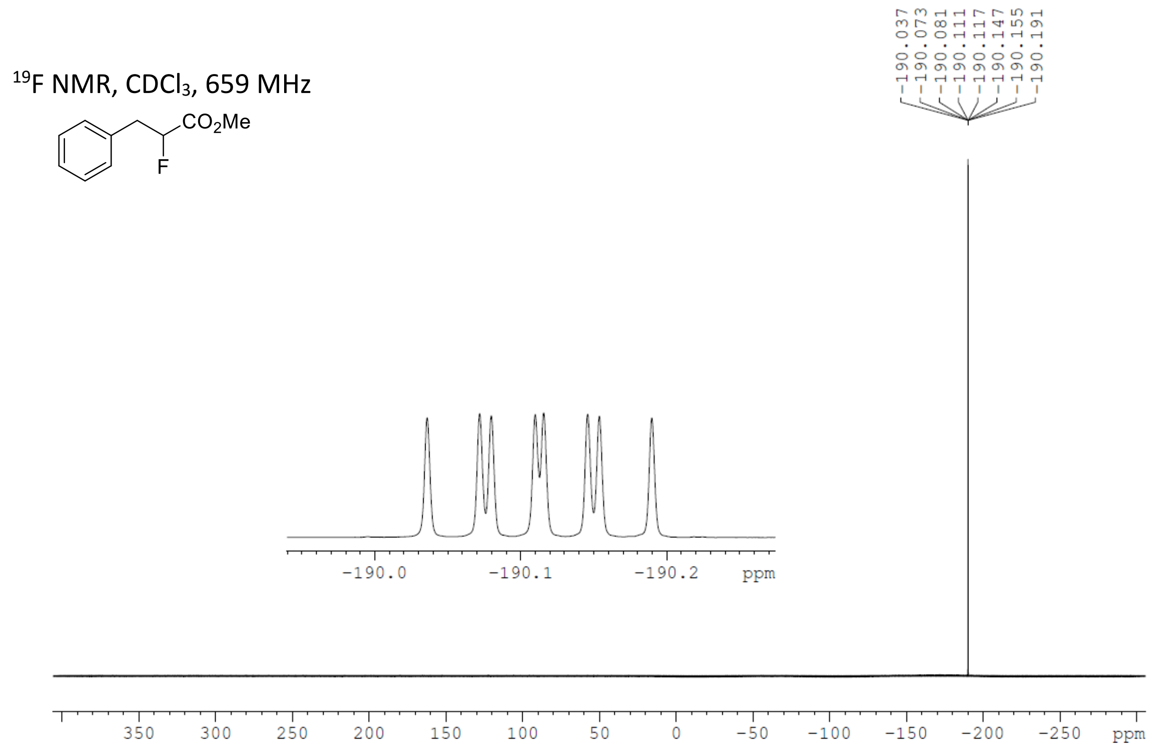

$^{19}F$ NMR, $CDCl_3$, 659 MHz
$CO_2Me$
F
-190.037
-190.073
-190.081
-190.111
-190.117
-190.147
-190.155
-190.191
-190.0 -190.1 -190.2 ppm
350 300 250 200 150 100 50 0 -50 -100 -150 -200 -250 ppm

Methyl 2-fluoro-3-(4-trifluoromethylphenyl)propanoate (12b)

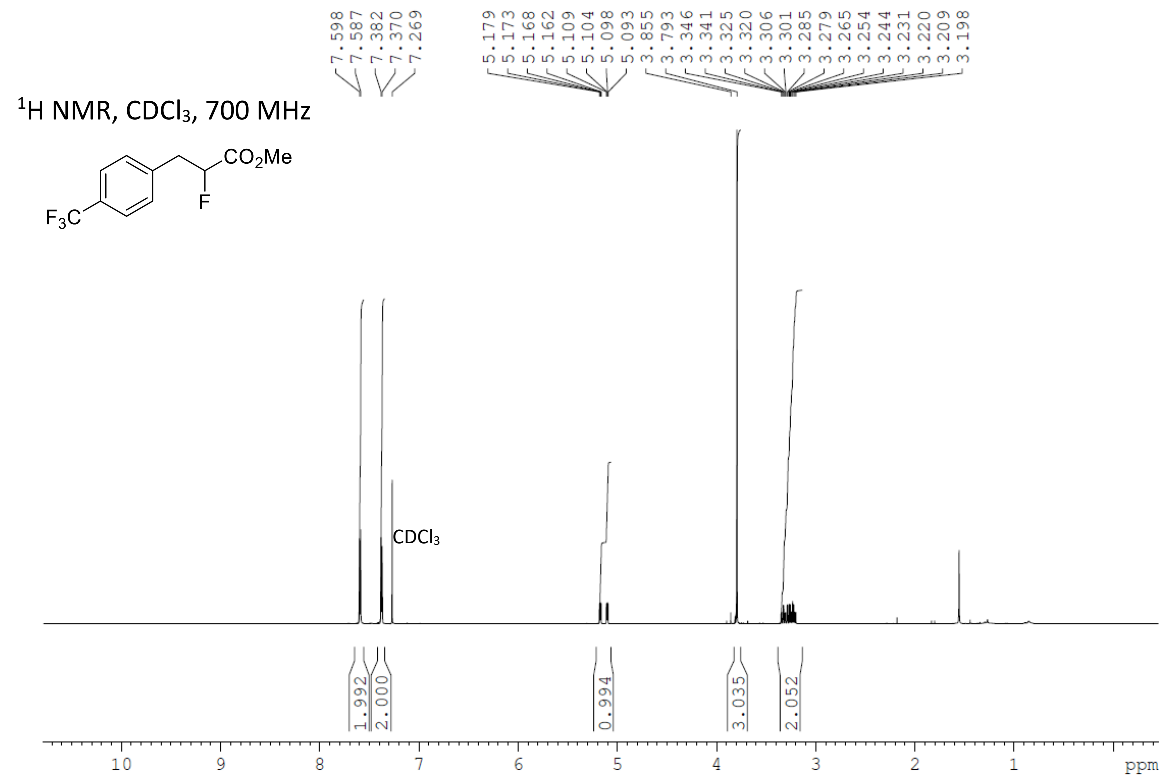


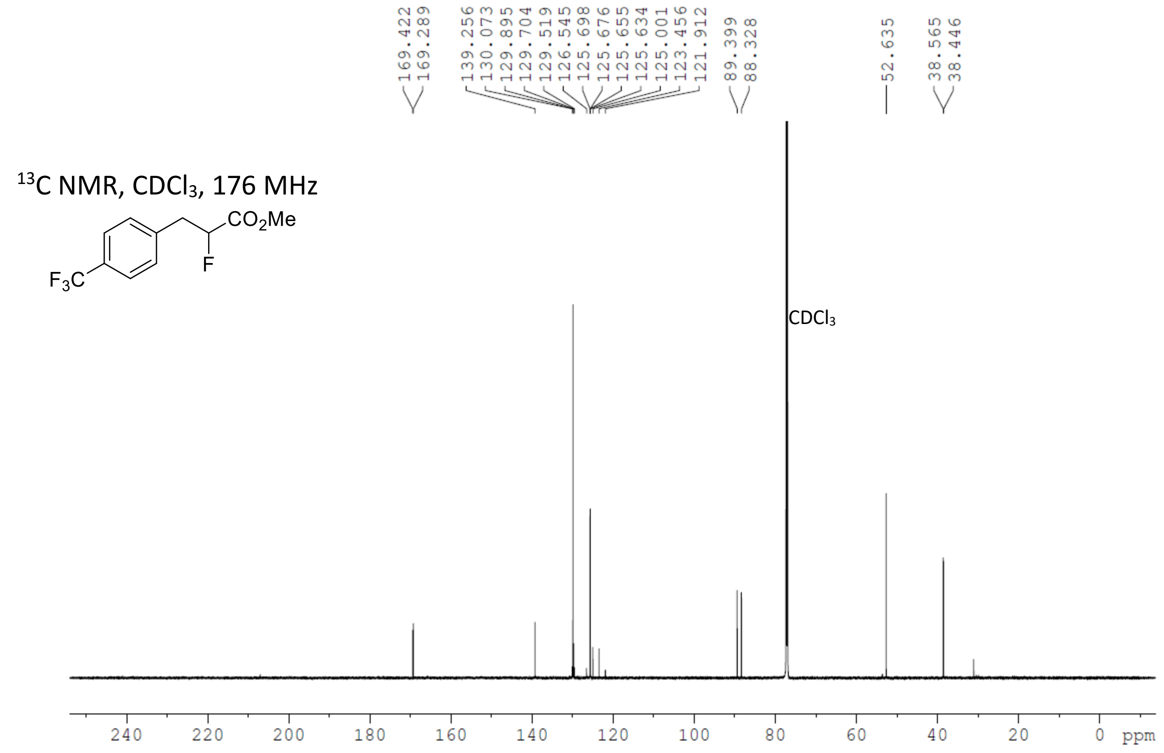

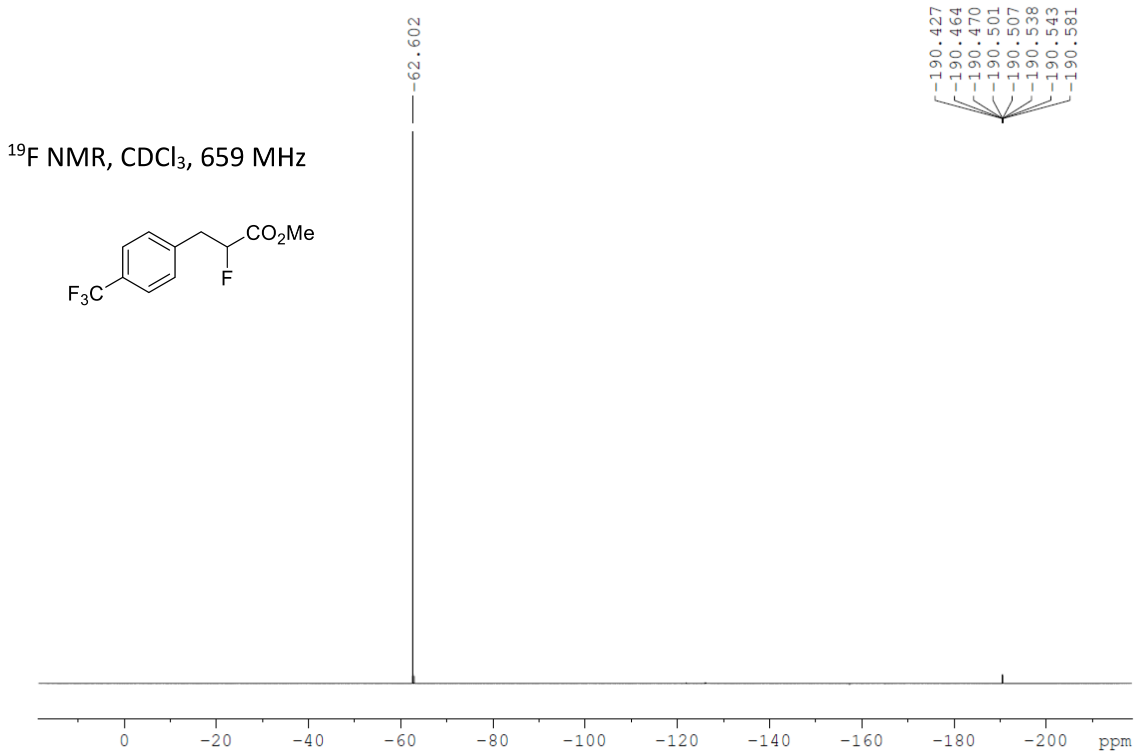


# Methyl 2-fluoro-3-(4-methoxyphenyl)propanoate (12c)

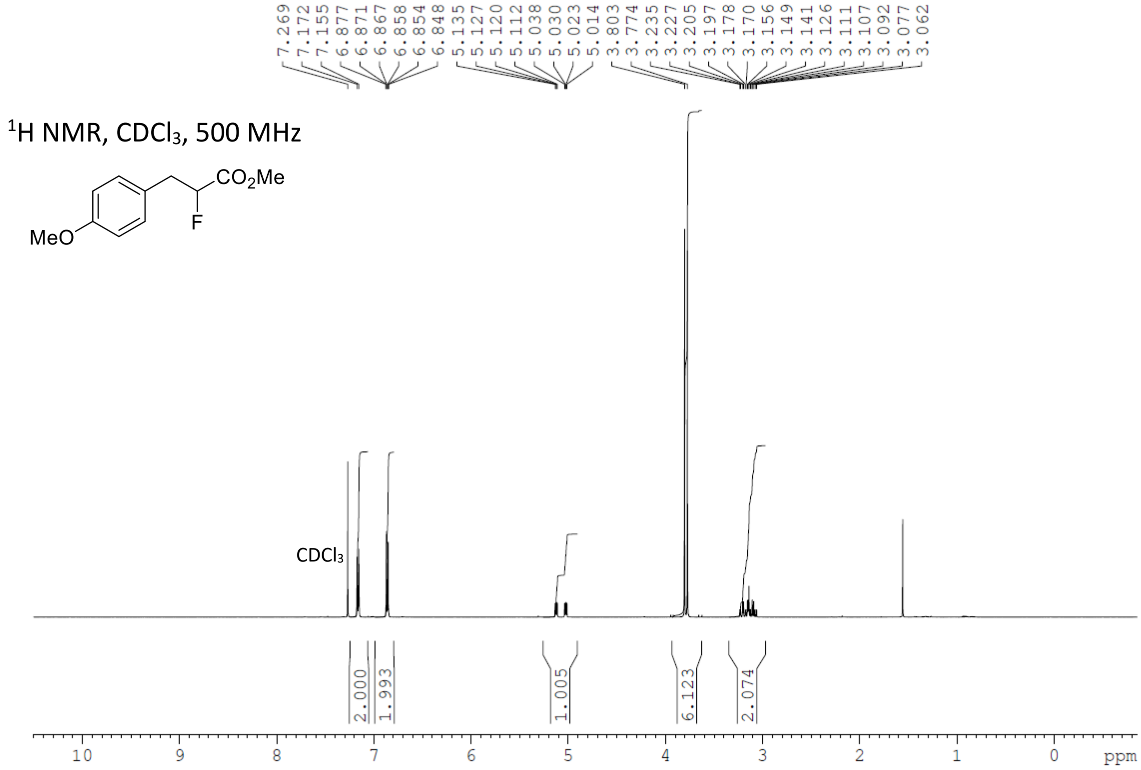

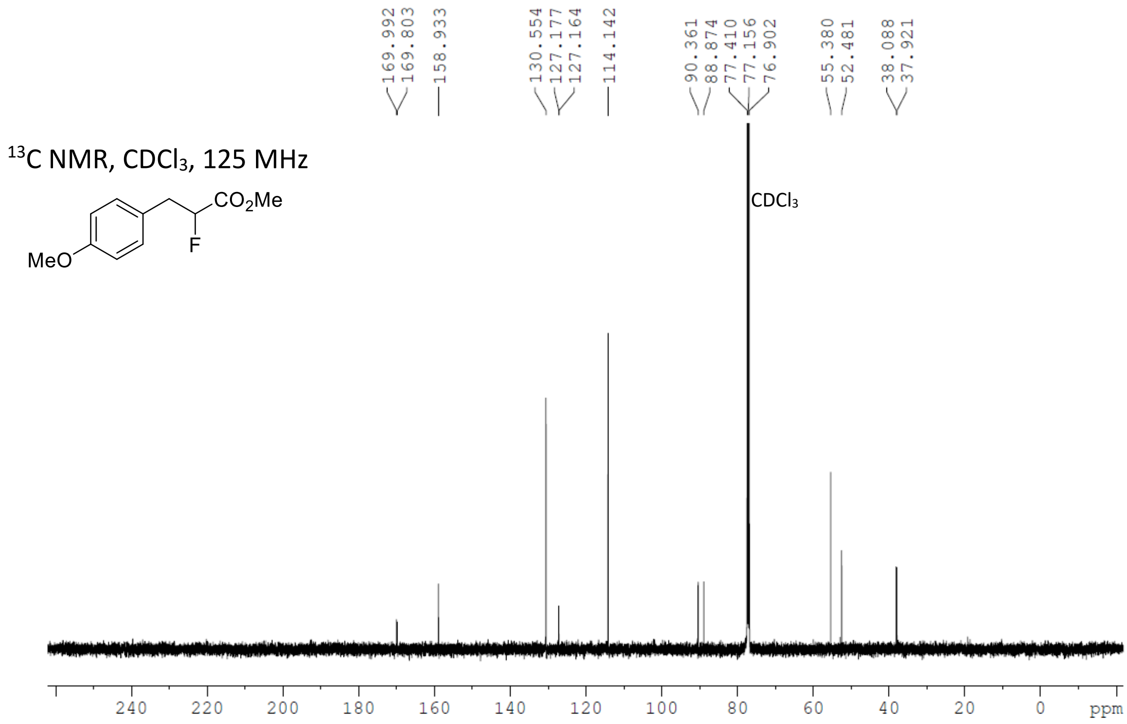
13C NMR, CDCl3, 125 MHz
CO2Me
F
MeO
CDCl3
169.992
169.803
158.933
130.554
127.177
127.164
114.142
90.361
88.874
77.410
77.156
76.902
55.380
52.481
38.088
37.921
240 220 200 180 160 140 120 100 80 60 40 20 0 ppm

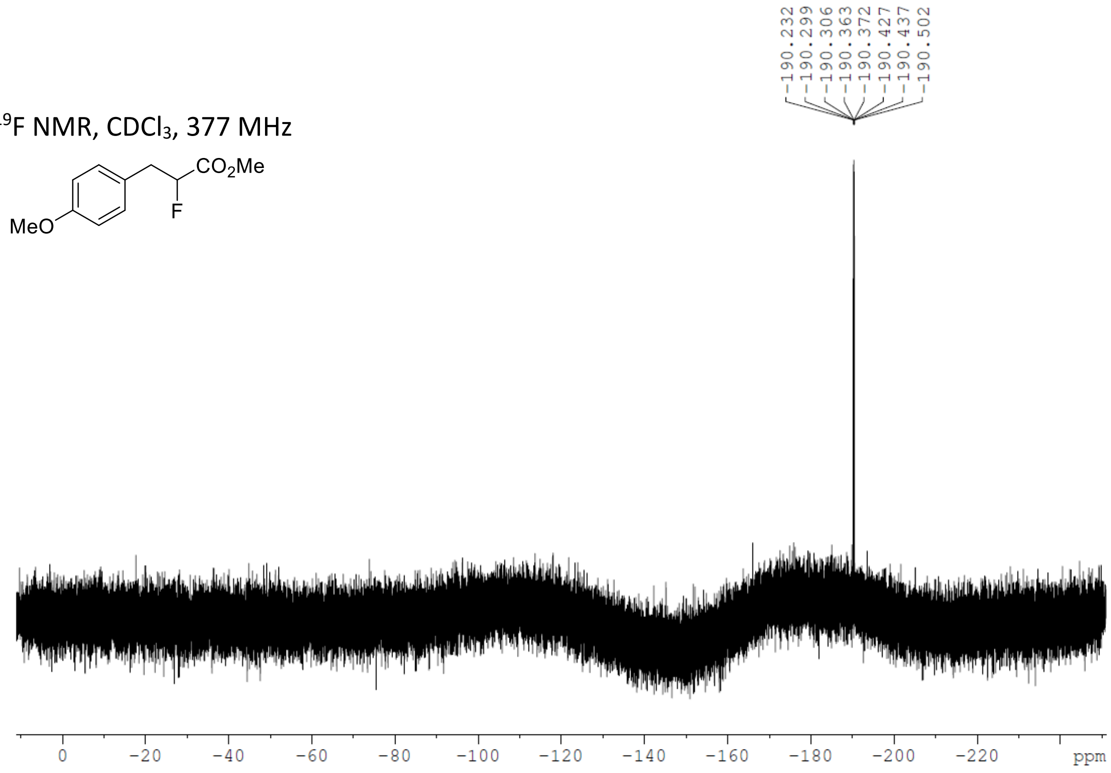
19F NMR, CDCl3, 377 MHz
CO2Me
F
MeO
-190.232
-190.299
-190.306
-190.363
-190.372
-190.427
-190.437
-190.502
0 -20 -40 -60 -80 -100 -120 -140 -160 -180 -200 -220 ppm

# Ethyl 2-fluoro-3-(4-trifluoromethylphenyl)propanoate (12d)

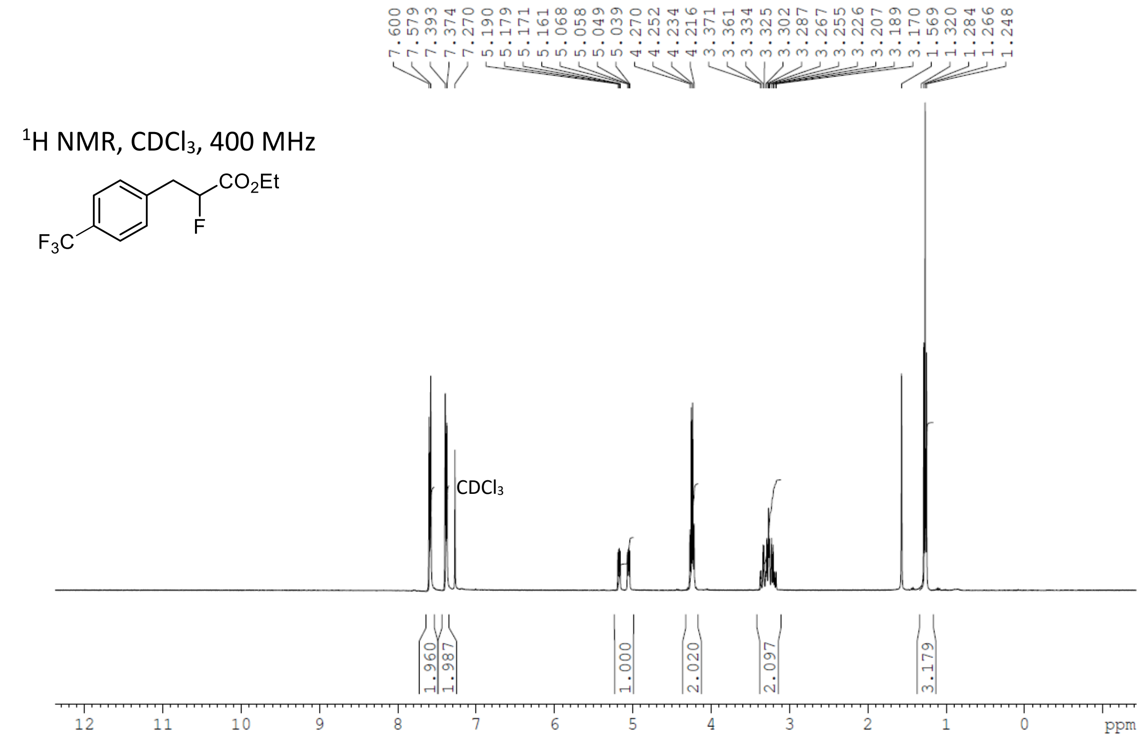


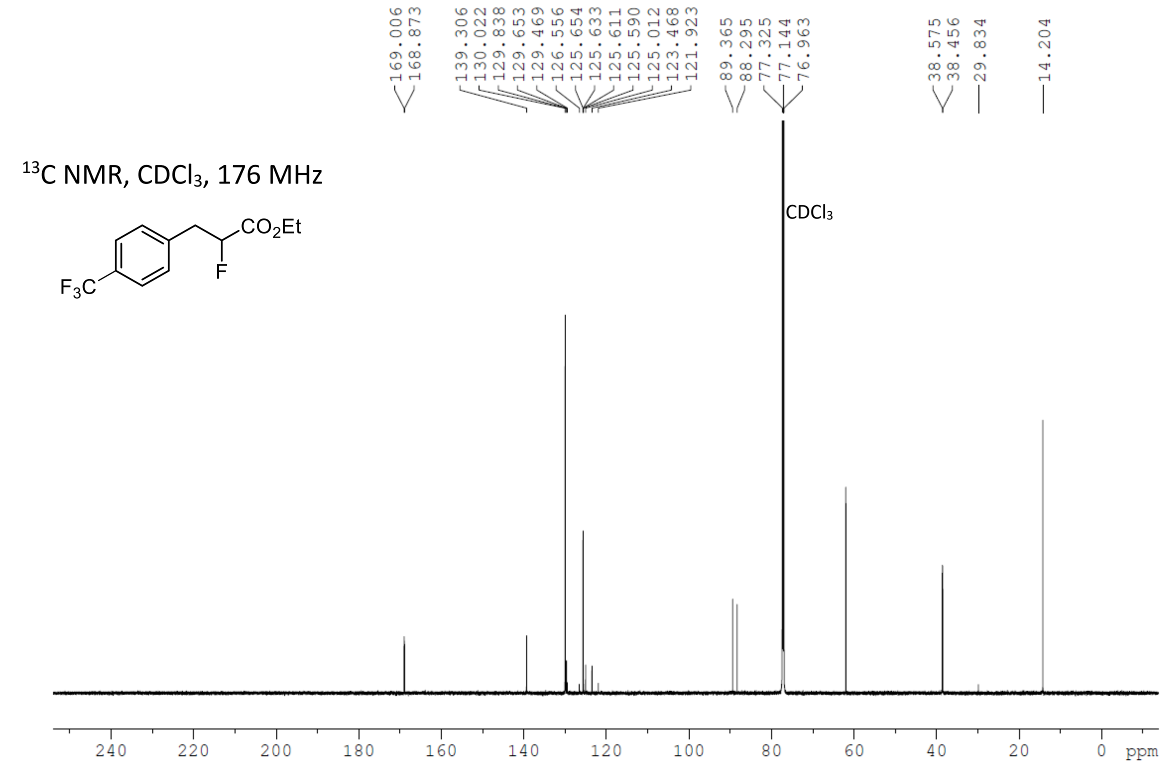

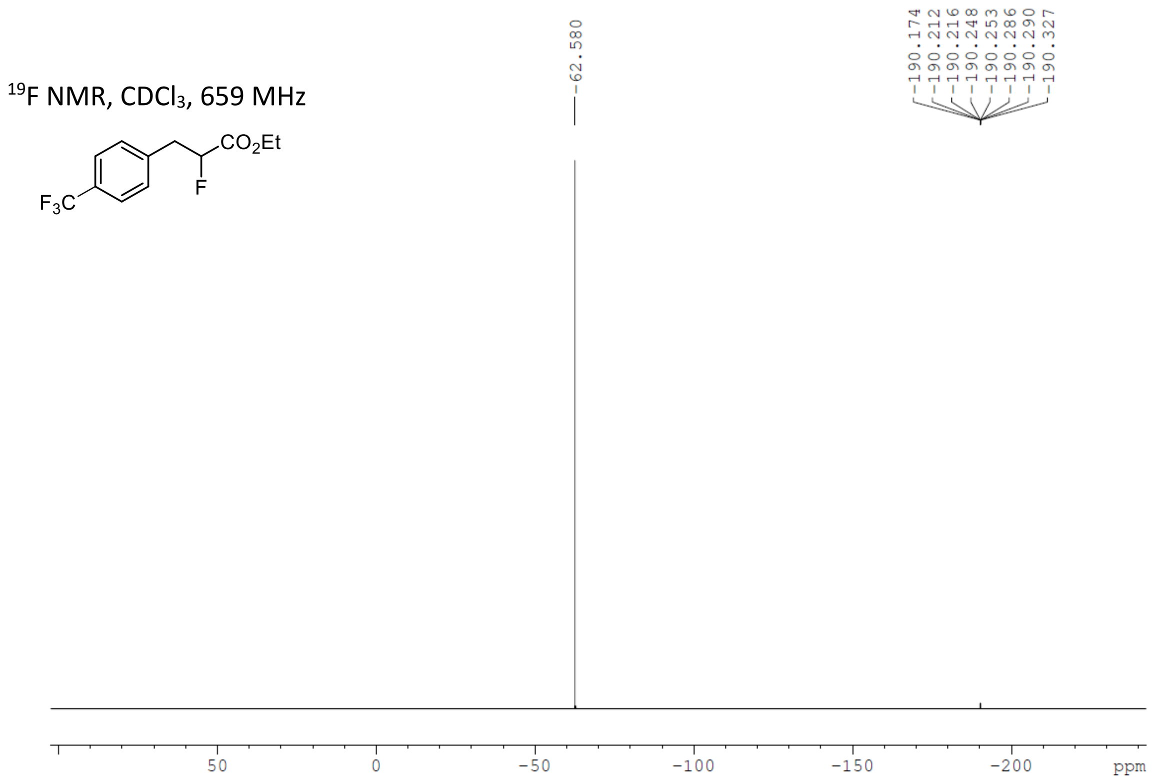


# Methyl 2-fluoroheptanoate (12e)

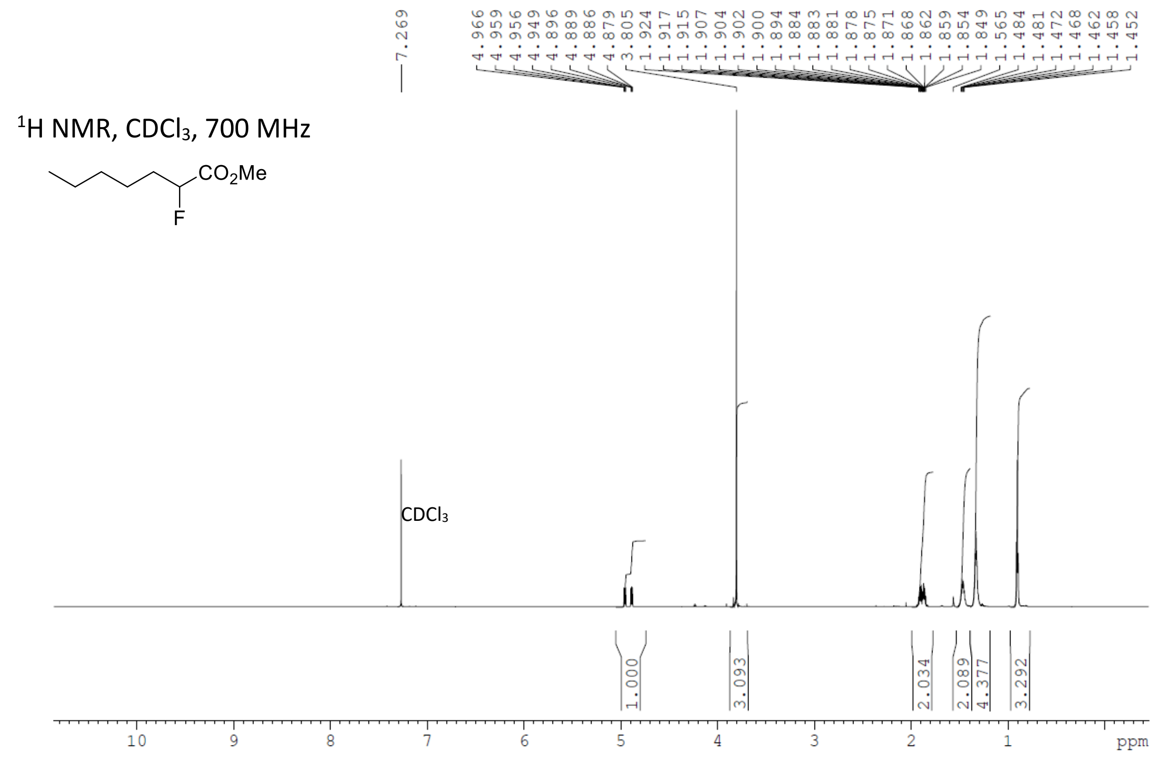

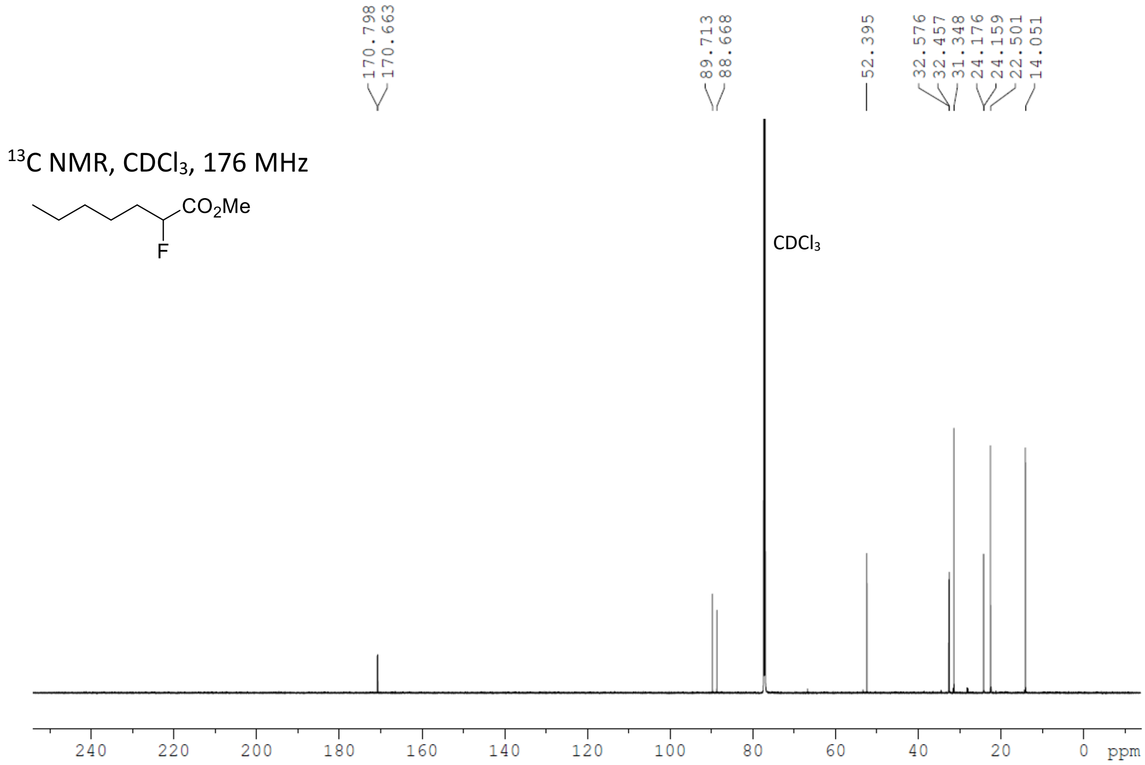
170.798
170.663
89.713
88.668
52.395
32.576
32.457
31.348
24.176
24.159
22.501
14.051
13C NMR, CDCl3, 176 MHz
CO2Me
F
CDCl3
240
220
200
180
160
140
120
100
80
60
40
20
0
ppm

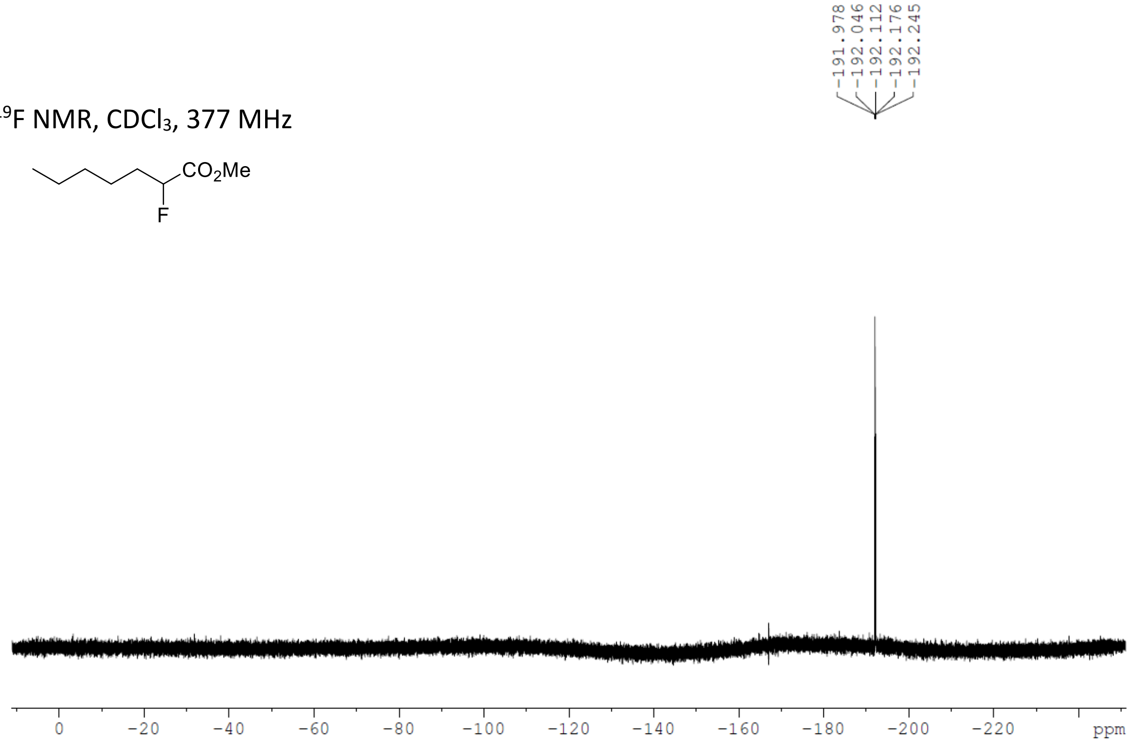
-191.978
-192.046
-192.112
-192.176
-192.245
19F NMR, CDCl3, 377 MHz
CO2Me
F
0
-20
-40
-60
-80
-100
-120
-140
-160
-180
-200
-220
ppm

# (2-Fluoro-3-methylbut-3-en-1-yl)benzene (13)

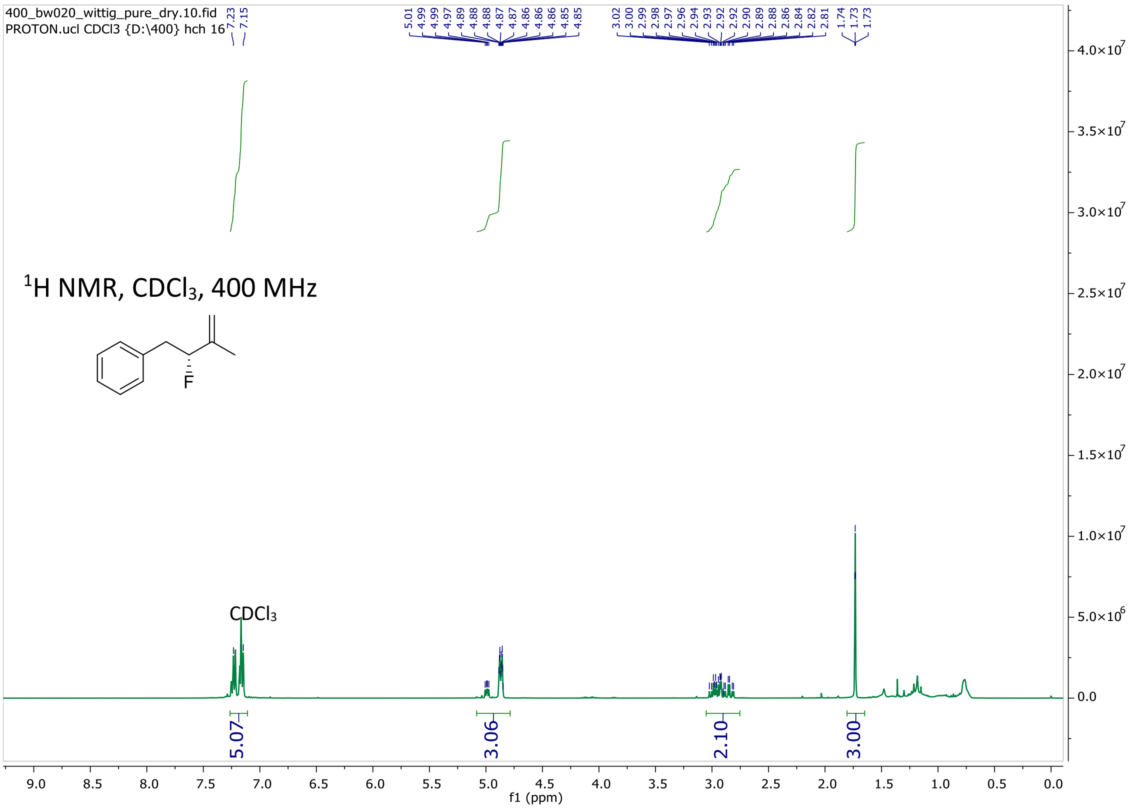


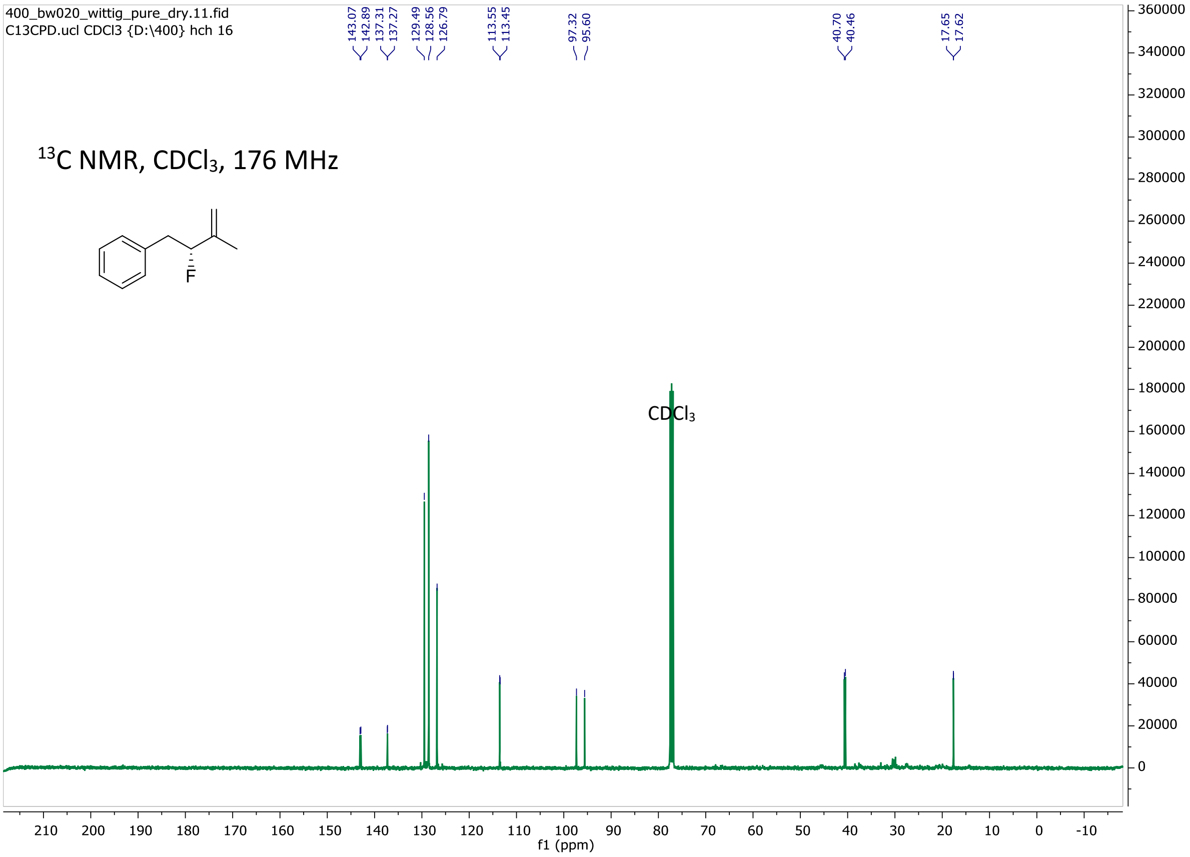

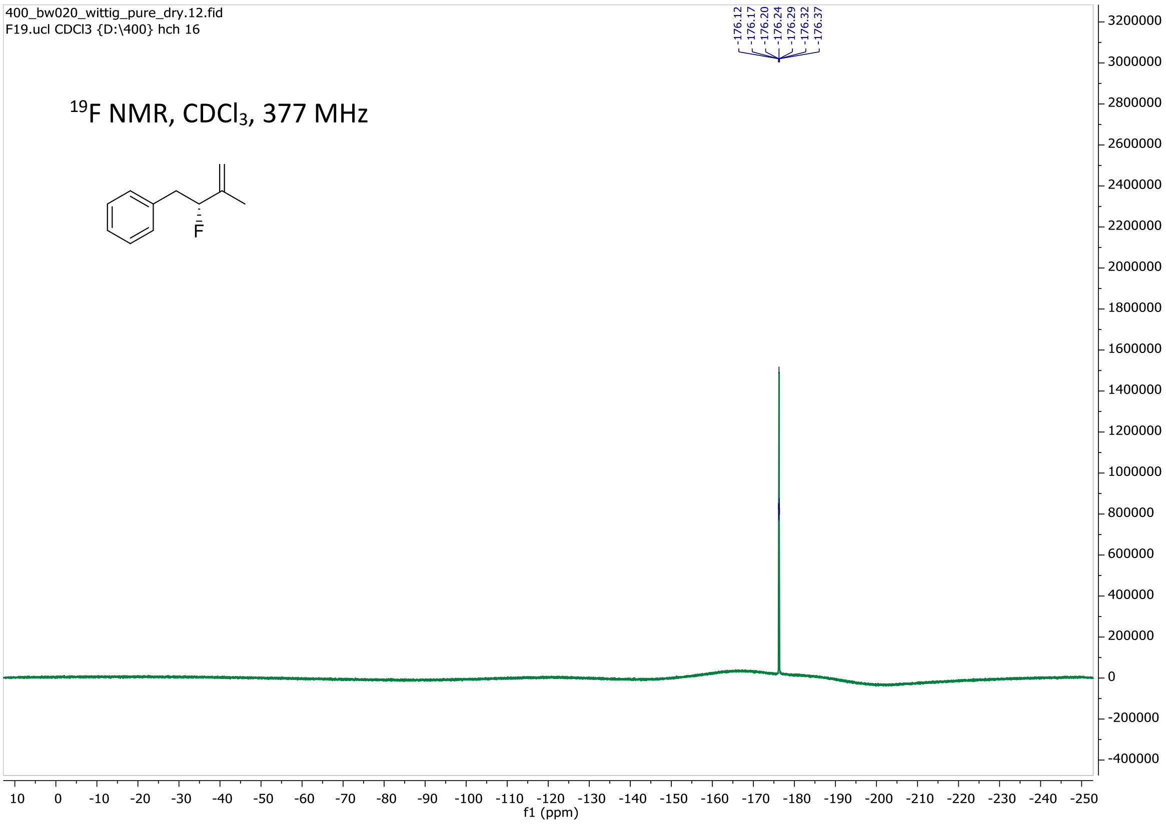


# 3-Fluoro-2-methyl-4-phenylbutan-2-ol (14)

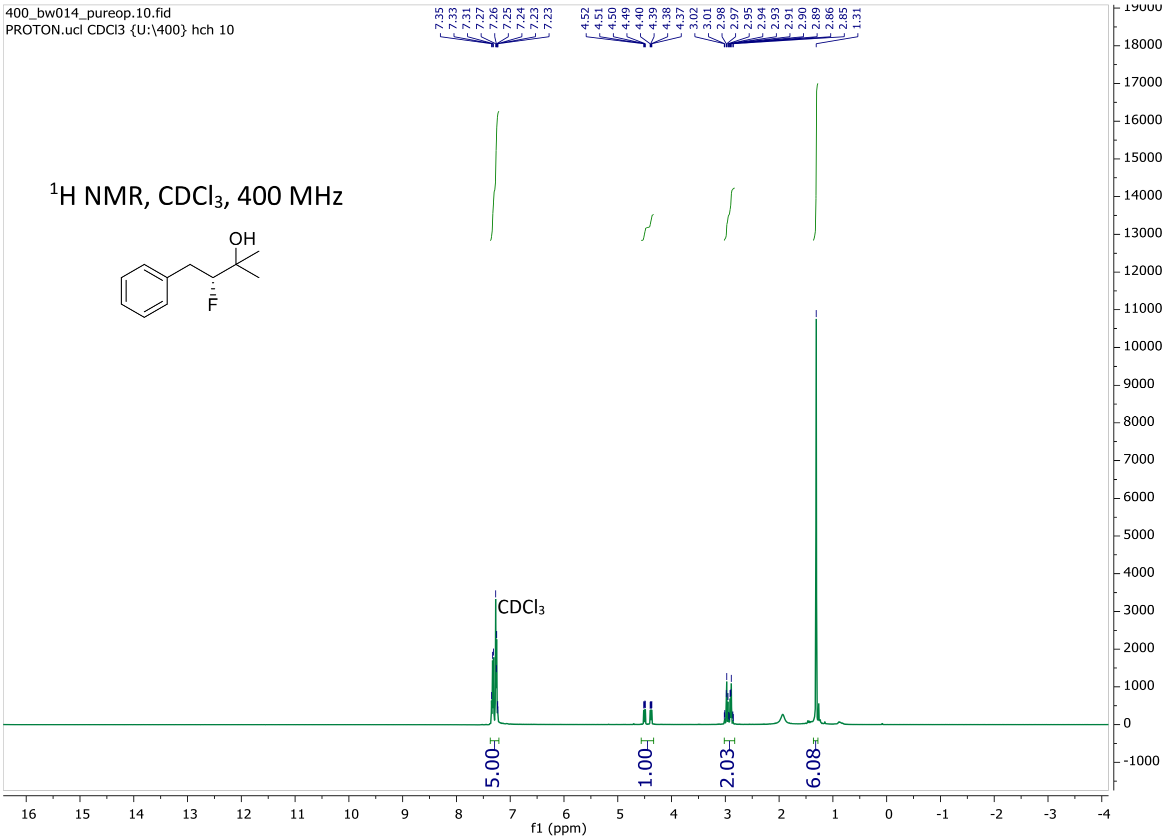

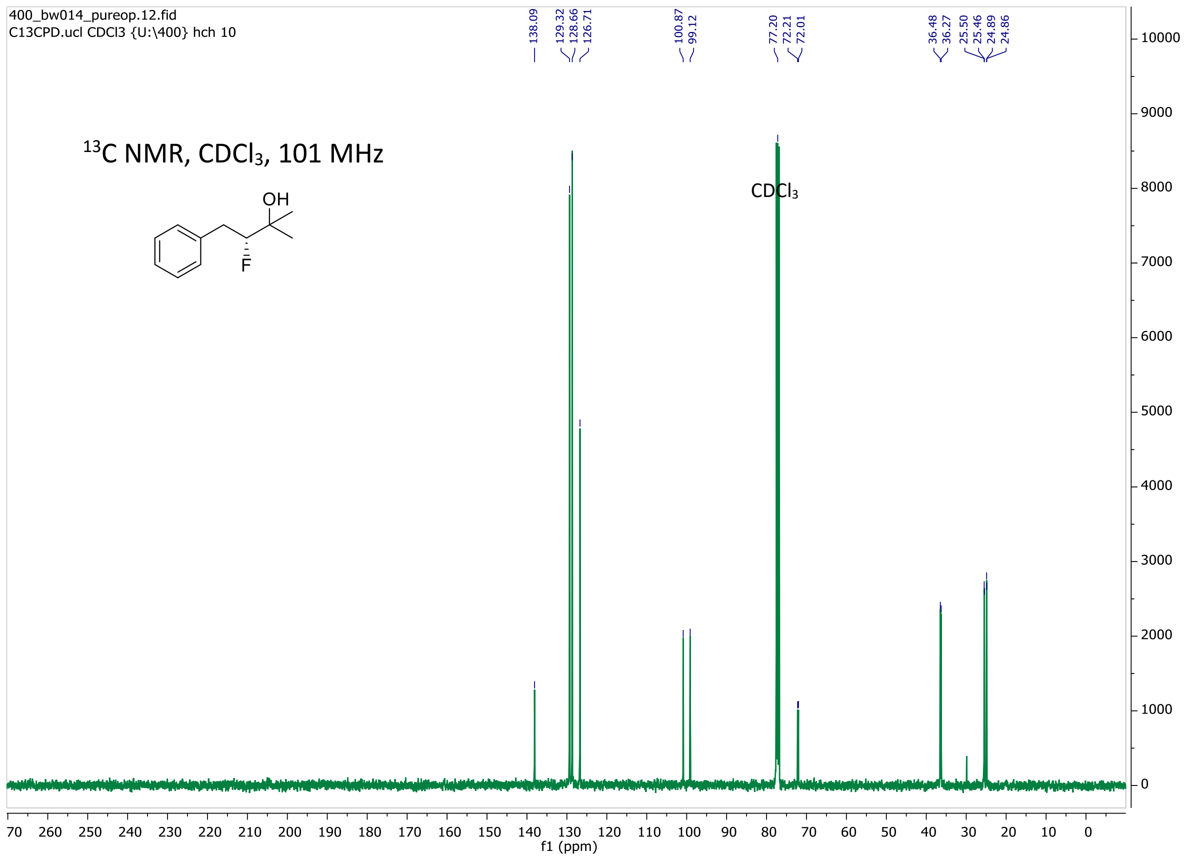
400_bw014_pureop.12.fid
C13CPD.ucl CDCl3 {U:\400} hch 10
138.09
129.32
128.66
126.71
100.87
99.12
77.20
72.21
72.01
36.48
36.27
25.50
25.46
24.89
24.86
13C NMR, CDCl3, 101 MHz
OH
F
CDCl3
f1 (ppm)

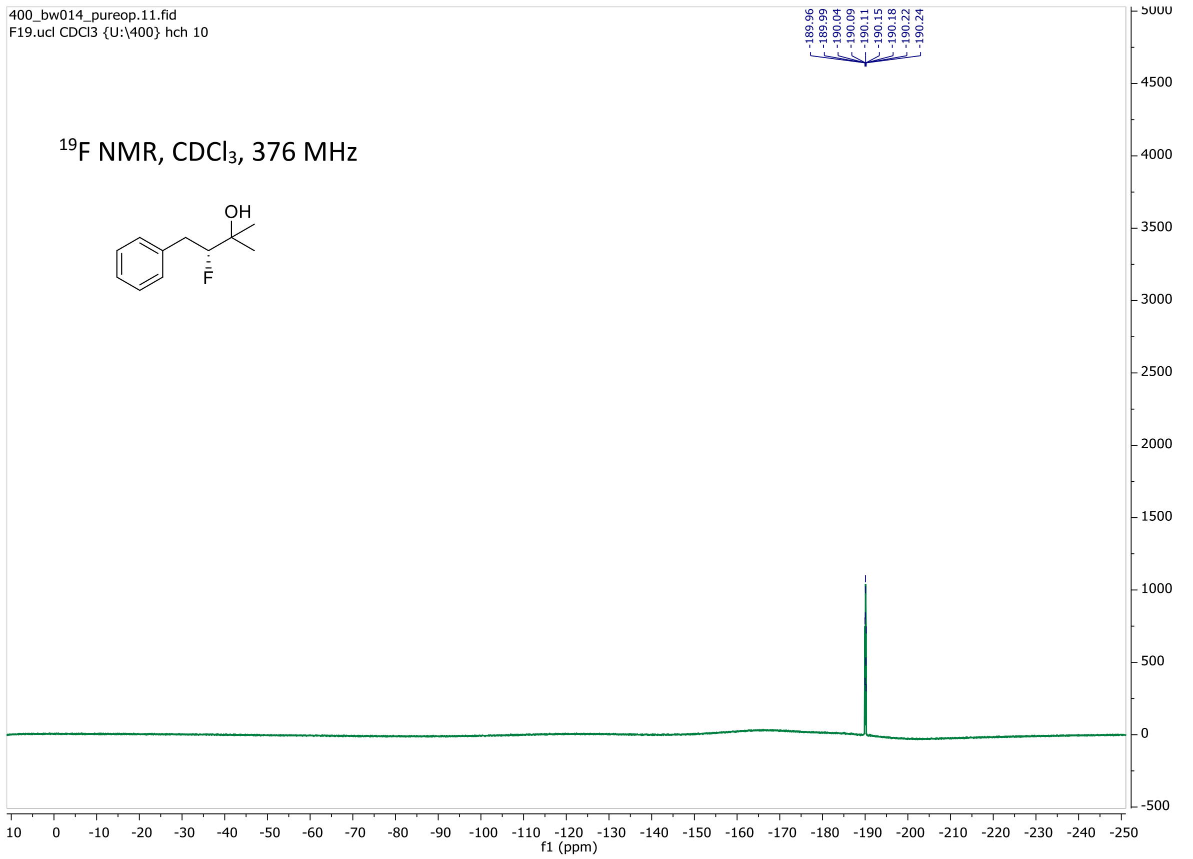
400_bw014_pureop.11.fid
F19.ucl CDCl3 {U:\400} hch 10
-189.96
-189.99
-190.04
-190.09
-190.11
-190.15
-190.18
-190.22
-190.24
19F NMR, CDCl3, 376 MHz
OH
F
f1 (ppm)

(2*S*,3*R*),(2*R*,3*R*)-3-Fluoro-4-phenylbutan-2-ol (15)

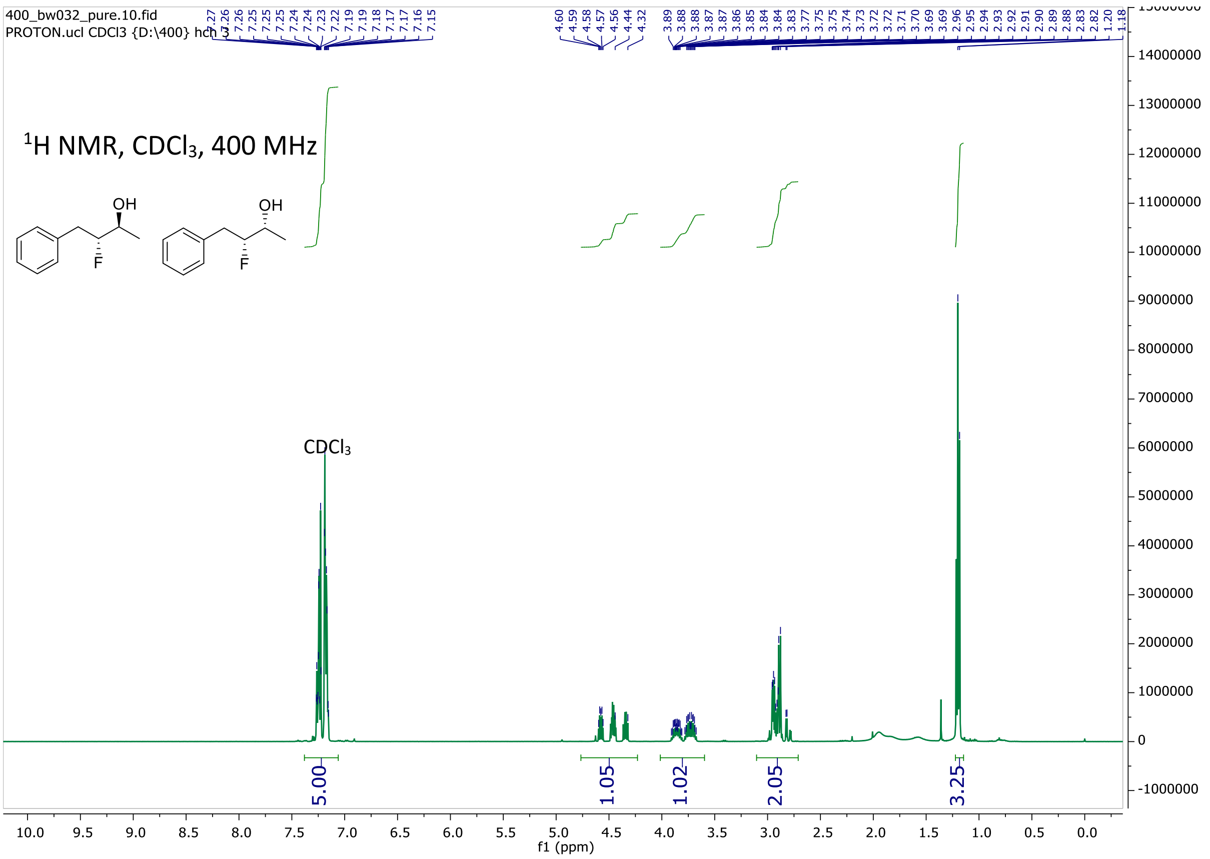


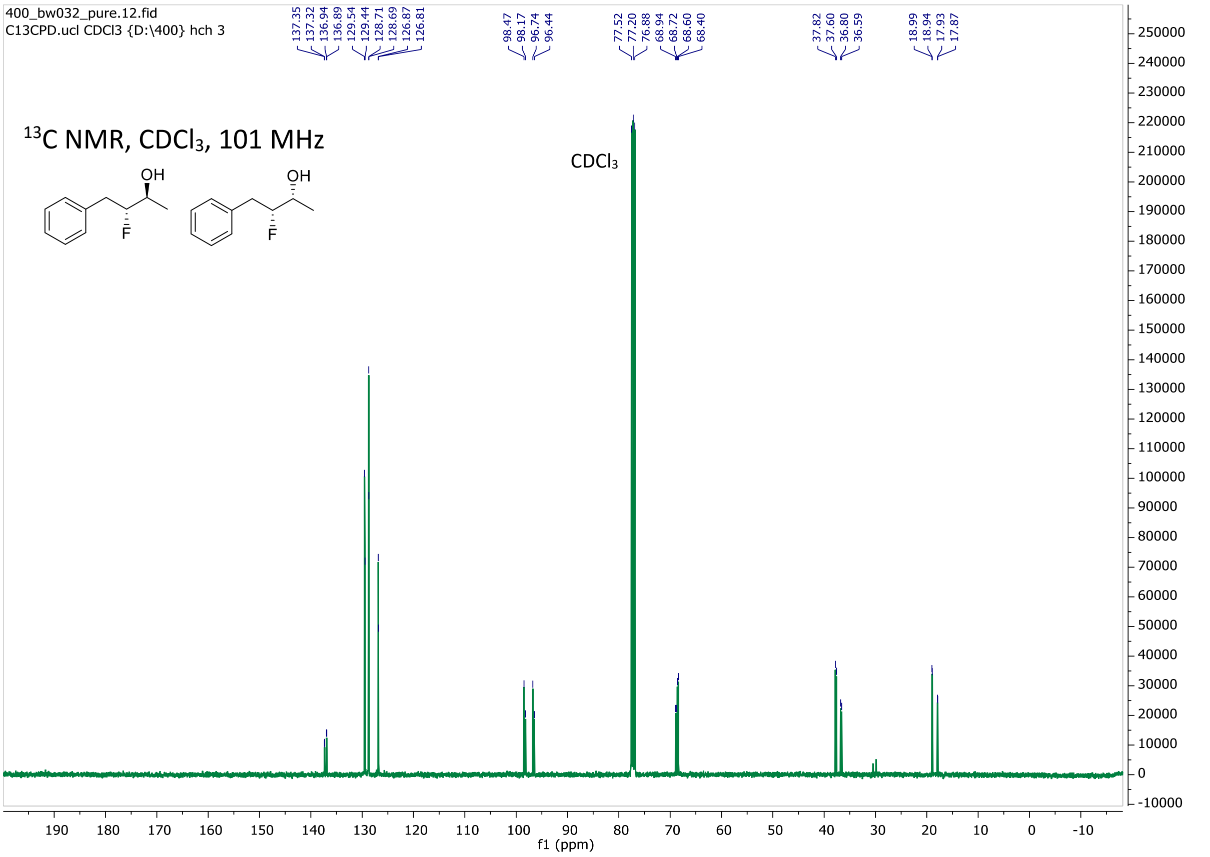

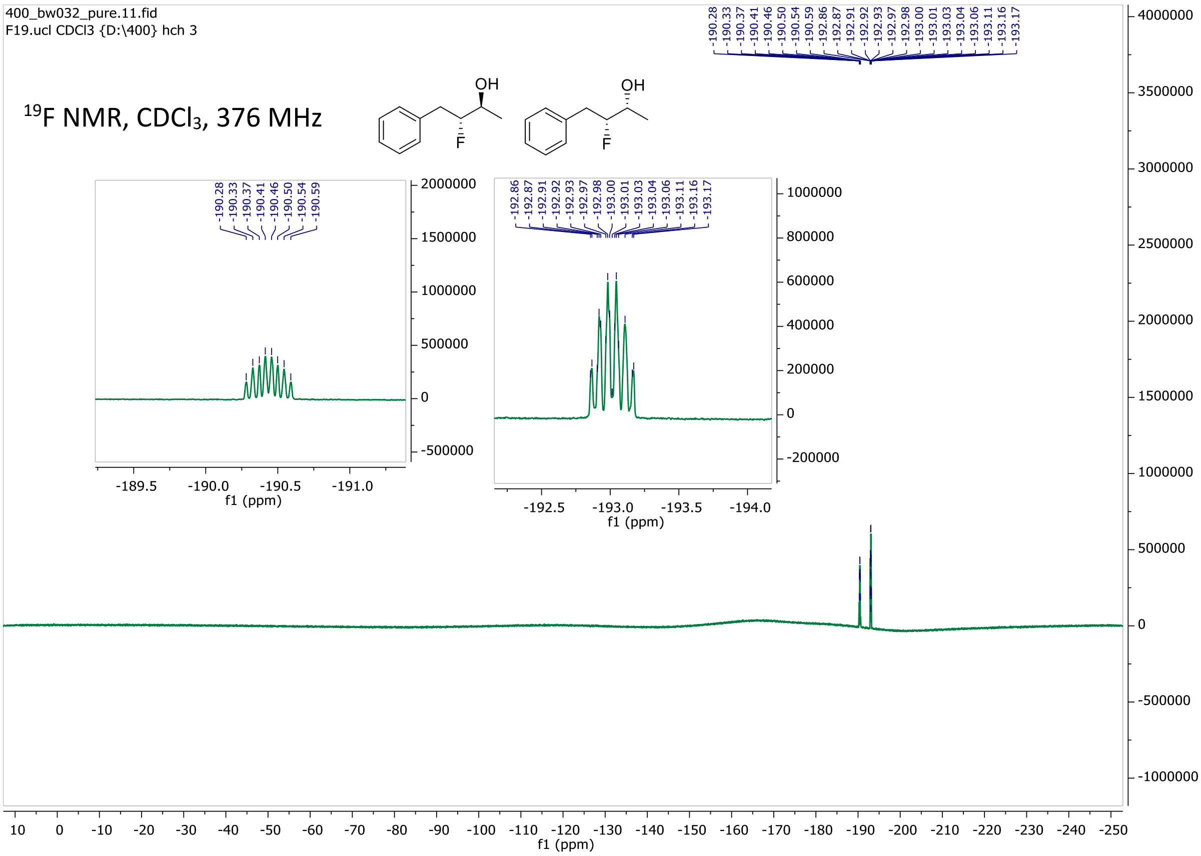
400_bw032_pure.11.fid
F19.ucl CDCl3 {D:\400} hch 3
19F NMR, CDCl3, 376 MHz
OH
F
OH
F
f1 (ppm)
